# The HLA region in ANCA-associated vasculitis: characterisation of genetic associations in a Scandinavian patient population

**DOI:** 10.1136/rmdopen-2023-004039

**Published:** 2024-04-04

**Authors:** Christian Lundtoft, Ann Knight, Jennifer R S Meadows, Åsa Karlsson, Solbritt Rantapää-Dahlqvist, Ewa Berglin, Øyvind Palm, Hilde Haukeland, Iva Gunnarsson, Annette Bruchfeld, Mårten Segelmark, Sophie Ohlsson, Aladdin J Mohammad, Per Eriksson, Peter Söderkvist, Lars Ronnblom, Roald Omdal, Roland Jonsson, Kerstin Lindblad-Toh, Johanna Dahlqvist

**Affiliations:** 1 Department of Medical Sciences, Uppsala University, Uppsala, Sweden; 2 Uppsala University Hospital, Uppsala, Sweden; 3 Department of Medical Biochemistry and Microbiology, Uppsala University, Uppsala, Sweden; 4 Department of Public Health and Clinical Medicine, Umeå University, Umeå, Sweden; 5 Department of Rheumatology, Oslo University Hospital, Oslo, Norway; 6 Department of Rheumatology, Martina Hansens Hospital, Sandvika, Norway; 7 Department of Medicine, Karolinska Institutet, Stockholm, Sweden; 8 Unit of Rheumatology, Karolinska University Hospital, Stockholm, Sweden; 9 Department of Health, Medicine and Caring Sciences, Linköping University, Linköping, Sweden; 10 Department of Renal Medicine, Karolinska University Hospital and CLINTEC Karolinska Institutet, Stockholm, Sweden; 11 Department of Clinical Sciences, Lund University and Skåne University Hospital, Lund, Sweden; 12 Department of Medicine, University of Cambridge, Cambridge, UK; 13 Department of Biomedical and Clinical Sciences, Linköping University, Linköping, Sweden; 14 Research Department, Stavanger University Hospital, Stavanger, Norway; 15 Department of Clinical Science, University of Bergen, Bergen, Norway; 16 Broegelmann Research Laboratory, Department of Clinical Science, University of Bergen, Bergen, Hordaland, Norway; 17 The Broad Institute of MIT and Harvard University, Cambridge, Massachusetts, USA

**Keywords:** Systemic vasculitis, Granulomatosis with polyangiitis, Autoimmune Diseases, Polymorphism, Genetic

## Abstract

**Objective:**

The antineutrophil cytoplasmic antibody (ANCA)-associated vasculitides (AAV) are inflammatory disorders with ANCA autoantibodies recognising either proteinase 3 (PR3-AAV) or myeloperoxidase (MPO-AAV). PR3-AAV and MPO-AAV have been associated with distinct loci in the human leucocyte antigen (HLA) region. While the association between MPO-AAV and HLA has been well characterised in East Asian populations where MPO-AAV is more common, studies in populations of European descent are limited. The aim of this study was to thoroughly characterise associations to the HLA region in Scandinavian patients with PR3-AAV as well as MPO-AAV.

**Methods:**

Genotypes of single-nucleotide polymorphisms (SNPs) located in the HLA region were extracted from a targeted exome-sequencing dataset comprising Scandinavian AAV cases and controls. Classical HLA alleles were called using xHLA. After quality control, association analyses were performed of a joint SNP/classical HLA allele dataset for cases with PR3-AAV (n=411) and MPO-AAV (n=162) versus controls (n=1595). Disease-associated genetic variants were analysed for association with organ involvement, age at diagnosis and relapse, respectively.

**Results:**

PR3-AAV was significantly associated with both HLA-DPB1*04:01 and rs1042335 at the *HLA-DPB1* locus, also after stepwise conditional analysis. MPO-AAV was significantly associated with HLA-DRB1*04:04. Neither carriage of HLA-DPB1*04:01 alleles in PR3-AAV nor of HLA-DRB1*04:04 alleles in MPO-AAV were associated with organ involvement, age at diagnosis or relapse.

**Conclusions:**

The association to the HLA region was distinct in Scandinavian cases with MPO-AAV compared with cases of East Asian descent. In PR3-AAV, the two separate signals of association to the *HLD-DPB1* region mediate potentially different functional effects.

**WHAT IS ALREADY KNOWN ON THIS TOPIC**

- Proteinase 3 (PR3)-antineutrophil cytoplasmic antibody (ANCA) positive and myeloperoxidase (MPO)-ANCA positive ANCA-associated vasculitides (AAV) are associated with distinct genetic loci in the human leucocyte antigen (HLA) region, but the associations have not been thoroughly characterised.

**WHAT THIS STUDY ADDS**

- In Scandinavian patients, the strongest associated allele within the HLA region in MPO-ANCA positive AAV was HLA-DRB1*04:04.
- Two loci in the *HLA-DPB1* gene region were significantly associated with PR3-ANCA positive AAV.

**HOW THIS STUDY MIGHT AFFECT RESEARCH, PRACTICE OR POLICY**

- The results of this study indicate differential genetic susceptibility at the HLA locus for MPO-ANCA positive AAV in populations of East Asian versus Scandinavian descent.
- The results of this study suggest that residues 74 and 86 of the HLA-DRB1 peptide-binding groove are critical in the pathogenesis of MPO-ANCA positive AAV.
- The results of this study suggest different mechanistic effects of the associated HLA loci in PR3-ANCA positive AAV.

## Introduction

Antineutrophil cytoplasmic antibody (ANCA)-associated vasculitides (AAV) comprise a group of chronic inflammatory disorders, clinically categorised as granulomatosis with polyangiitis (GPA), microscopic polyangiitis (MPA) and eosinophilic GPA (EGPA). ANCAs recognising either proteinase 3 (PR3) or myeloperoxidase (MPO) are present in a majority of cases with GPA and MPA and in about 30%–40% of EGPA. Subsequently, the AAV may also be categorised according to ANCA subtype: PR3-ANCA positive AAV (PR3-AAV), mainly associated with GPA, and MPO-ANCA positive AAV (MPO-AAV), mainly associated with MPA. The AAV are rare, worldwide, and the annual incidence in AAV is approximately 15–30 cases/million person years.^1–3^ Several studies have demonstrated that there is a geographical difference in the distribution of specific AAV diagnoses, with MPA being more common in East Asia (79%–83% of AAV) compared with, for example, Northern Europe (30%–35% of AAV), whereas the opposite is true for GPA (~20% of AAV in East Asia and 55%–70% in Northern Europe, respectively).^2 4 5^ Likewise, MPO-AAV is the dominant form in East Asia (~80%–90% of AAV) while that subgroup constitutes approximately 50%–60% of AAV in Northern Europe.^2 5 6^ The reason for these differences is not completely understood, but an effect of both genetic and environmental factors is plausible.

The pathogenesis of the necrotising inflammation in small-sized and medium-sized blood vessels seen in AAV is yet poorly understood, but both environmental and genetic factors with impact on disease risk have been identified.^7 8^ Importantly, the genetic predisposition differs between GPA and MPA, and, additionally, the genetic susceptibility loci show a stronger association with ANCA subtype (PR3-ANCA and MPO-ANCA) than with the clinical diagnoses GPA and MPA.^9^ Genome-wide association studies (GWAS) of single-nucleotide polymorphisms (SNPs) and a large-scale DNA sequencing study have identified the human leucocyte antigen-(HLA)-DP and HLA-DQ regions to have the strongest genetic impact on PR3-AAV and MPO-AAV, respectively.^9–11^

The HLA class II genomic region is highly polymorphic and characterised by complex patterns of linkage disequilibrium (LD). Apart from SNP analyses, the genetic variability of this region has been thoroughly investigated in autoimmune diseases based on classical HLA alleles, where, at four-digit resolution, specific combinations of amino acids in the coding region of the HLA molecule can be distinguished.^12^ Strikingly, many traits are associated with different HLA alleles in different ethnic populations, plausibly explained by a difference in frequencies of the HLA alleles in different populations. GPA and PR3-AAV have been associated with HLA-DPB1*04:01 in populations of European descent in multiple studies,^13–17^ with HLA-DRB1*12:02 in a Chinese population^18^ and with HLA-DRB1*15:01 in African Americans in North America.^19^ In contrast, MPA and MPO-AAV have been associated with HLA-DRB1*09:01 and HLA-DQB1*03:03 in Japanese populations^20 21^ and with the haplotype HLA-DQA1*03:02-DQB1*03:03 and with HLA-DRB1*11:01, respectively, in different Chinese populations.^18 22^ Studies of HLA classical alleles in patients with MPO-AAV of European descent are, however, limited.

In this study, we aimed to characterise the associations between the HLA region and PR3-AAV and MPO-AAV, respectively, in an AAV population of Scandinavian descent. By integrating data of classical HLA alleles with that of SNP alleles, we were able to isolate signals of association specific for the AAV subgroups.

## Material and methods

### Subjects

Patients diagnosed with GPA or MPA (n=679) were recruited to the study at clinics of rheumatology or nephrology at university hospitals in Oslo (Norway), Uppsala, Umeå, Stockholm, Linköping, Lund and Malmö (Sweden) at diagnosis or at follow-up visits to the outpatient clinics, between 2008 and 2014. All patients met the corresponding classification criteria according to the European Medicines Agency algorithm^23^ and were included after informed and written consent. Clinical data, including sex, age, ANCA status, cumulative disease involvement of kidneys, ear–nose–throat (ENT) or lungs and the occurrence of relapse, were collected from patient medical records at time of inclusion in the study. Control samples (n=1706) were collected from population controls in Linköping/Jönköping (Southern Sweden) and Umeå (the biobank of Northern Sweden Health and Disease Study Cohort) and from healthy blood donors at Uppsala University Hospital (central Sweden), Haukeland University Hospital, Bergen and Stavanger University Hospital (both Norway). The study population was included in a previous genetic analysis.^11^

### DNA library preparation, sequencing and SNP calling

DNA samples were collected from all individuals and prepared and sequenced as described elsewhere.^11^ Briefly, exons and conserved regions within 100 000 base pairs of 1853 immune-related genes^24^ were selected in the DNA samples using a SeqCap EZ Choice XL library (Roche NimbleGen, Basel, Switzerland) and sequenced using Illumina HiSeq 2500 (San Diego, California). A standard pipeline for raw data processing was applied.^25^ Raw reads were mapped to the hg19 human reference genome using the Burrows-Wheeler aligner V.0.7.12^26^ and duplicate reads were marked by Picard V.1.92. GATK V.3.3.0^25^ was applied for realignment around indels, base quality score recalibration, SNP and indel discovery and genotyping. Alignment quality was evaluated by Samtools flagstat and Picard tools CalculateHSMetrics; samples with mean target coverage less than 10× were excluded. Indels were removed. SNP quality scores were recalibrated using GATK V.3.3.0 VariantRecalibrator and filtered at tranche level V.99.0. Using VCFtools V.0.1.14,^27^ genotype calls with depth less than 8 reads and genotype Phred quality score less than 20 were excluded. Sequence data were quality assessed concerning read depth, allelic imbalance, Hardy-Weinberg equilibrium and unequal missingness between cases and controls for variants and population stratification (LASER^28 29^) and cryptic relatedness (KING^30^) for samples, as previously described.^11^ The average sample call rate for the targeted regions was 98% and the average SNP call rate was 98%.

### Calling of HLA alleles and genetic association analyses

After quality control and exclusion of ANCA-negative cases and cases positive for both PR3-ANCA and MPO-ANCA, 573 (411 PR3-AAV, 162 MPO-AAV) cases and 1595 controls remained, forming the study population for analysis (table 1). xHLA,^31^ an algorithm capable of calling classical alleles of HLA-A, HLA-B, HLA-C, HLA-DPB1, HLA-DQB1 and HLA-DRB1 with 99%–100% 4-digit typing accuracy, was used to call HLA alleles from indexed bam files of all individuals of the study population. The average call rate was 97.3% (online supplemental table 1). Association analysis of a merged dataset of classical HLA alleles and SNPs (minor allele frequencies (MAFs) ≥0.01) located within the HLA region (chr6:29 600 000–33 500 000, hg19) was performed separately for PR3-AAV, MPO-AAV, GPA and MPA using logistic regression analyses, with adjustment for sex and genetic structure (principal component (PC)1–PC4). Association analyses for organ involvements (pulmonary, ENT and renal) were performed for the entire AAV population with organ involvement as response variable (present=1, absent=0). Conditional analyses were performed for PR3-AAV and MPO-AAV by reanalysis of the data using the lead HLA allele/SNP as covariate, adding any remaining significantly associated variant(s) to the list of covariates, and iterating the procedure until no additional significantly associated variants remained (Plink V.1.9^32^). A standard GWAS significance threshold of p <5 × 10^–8^ was applied to account for multiple testing. At this significance level and for a MAF of 0.16 (median of all variants), the power was >80% to detect an OR of >1.8 for PR3-AAV and >2.4 for MPO-AAV. LDlink^33^ (CEU population) was used for pairwise calculations of LD between SNPs, whereas Pearson correlation was used for calculations of LD (r^2^) between HLA-DRB1*04:04 and rs35874654.

10.1136/rmdopen-2023-004039.supp1 Supplementary data

**Table 1:** Clinical characteristics of cases and controls included in association analyses after data quality control

	Cases	Controls
Total	573	1595
Females, n (%)	290 (51)	1169 (73)
Age at diagnosis, mean (SD)	54 (18)	–
Age at sampling, mean (SD)	59 (17)	58 (14)
GPA, n (%)	434 (76)	–
MPA, n (%)	139 (24)	–
PR3-ANCA+, n (%)	411 (72)	–
MPO-ANCA+, n (%)	162 (28)	–
ENT involvement,* n (%)	380 (66)	–
Kidney involvement,* n (%)	383 (67)	–
Pulmonary involvement,* n (%)	303 (53)	–
Relapse†, n (%)	217 (54)	–

*Defined as glomerulonephritis (kidneys) or any kind of pulmonary/ENT involvement, respectively.

†Missing data, n=172.

ANCA, antineutrophil cytoplasmic autoantibody; ENT, ear–nose–throat; GPA, granulomatosis with polyangiitis; MPA, microscopic polyangiitis; MPO, myeloperoxidase; PR3, proteinase 3.

The number of risk alleles of the lead HLA variants HLA-DPB1*04:01 (in PR3-AAV) and HLA-DRB1*04:04 (in MPO-AAV) carried by each individual was compared between patients with specific organ involvements (pulmonary, renal, ENT) and patients without these involvements, in PR3-AAV and MPO-AAV, separately, using Fisher’s exact test. Additionally, each increase in the number of HLA risk alleles (HLA-DPB1*04:01 alleles for PR3-AAV and HLA-DRB1*04:04 alleles for MPO-AAV) was analysed for association with age at diagnosis of PR3-AAV and MPO-AAV, respectively, using Cox proportional hazards analysis with sex and PC1–PC4 as covariates. The age at diagnosis was plotted in relation to the number of risk HLA alleles using Kaplan-Meier curves (R V.4.0.4). Associations between the risk HLA alleles and risk of relapse were analysed using logistic regression, with sex as covariate (R V.4.0.4).

### HLA allele alignment and modelling

The IPD-IMGT/HLA database^34^ was used for alignment of HLA-DRB1 alleles. The RCSB protein data bank^35^ was used for 3D modelling of HLA-DRB1, antigen and T cell receptor; ID 2IAM.^36^

## Results

### Genetic analyses of the HLA region in PR3-AAV

**Figure 1:**
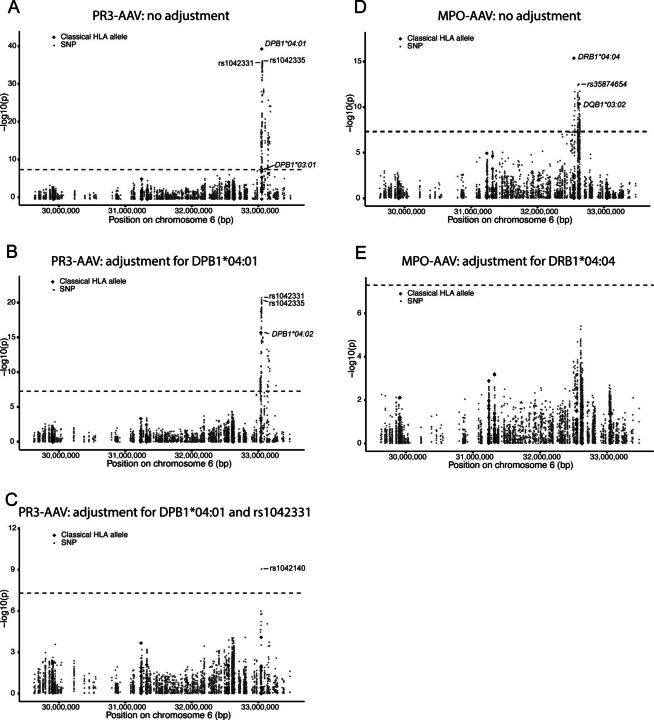
Genetic association analyses of the human leucocyte antigen (HLA) region in antineutrophil cytoplasmic antibody-associated vasculitides (AAV). Manhattan plots depicting signals of association between a combined dataset of HLA alleles and single-nucleotide polymorphisms (SNPs) and proteinase 3 (PR3)-AAV (A–C) and myeloperoxidase (MPO)-AAV (D–E), respectively. Plots show results without conditioning on any genetic variant (A, D) and conditioning on lead variants: HLA-DPB1*04:01 (B), HLA-DPB1*04:01 and rs1042331 (C) in PR3-AAV and HLA-DRB1*04:04 (E) in MPO-AAV. Significantly associated classical HLA alleles and lead SNPs are marked with ID. The −log10 of the p value of each HLA allele/SNP is plotted against the physical location of the variant in the HLA region on chromosome 6. Dashed black line corresponds to the p value threshold for significance (p <5 × 10^−8^). Diamonds=classical HLA alleles; dots=SNPs.

In order to characterise the genetic association between the HLA region and PR3-AAV in Scandinavian cases, we analysed an integrated dataset comprising classical HLA alleles and SNPs. The strongest association was identified for HLA-DPB1*04:01 (p=7.1×10^−38^, OR=3.3 (95% CI 2.8 to 4.0)), followed by SNP rs1042335 (p=6.3×10^−35^, OR=0.089 (95% CI 0.059 to 0.13); figure 1A, table 2). HLA-DPB1*03:01 (p=4.1×10^−8^, OR=0.23 (95% CI 0.13 to 0.37)) and 324 additional SNPs were also significantly associated with PR3-AAV (online supplemental table 2). After conditioning on HLA-DPB1*04:01, the significant association with rs1042335 persisted, although the strongest association was seen for rs1042331, a SNP in complete LD with rs1042335 (r^2^ 1.0, D’ 1.0), with p values 1.8×10^−21^ and 3.7×10^−21^, respectively (table 2). The association with HLA-DPB1*03:01 was no longer significant, instead a significant association with HLA-DPB1*04:02 was revealed (p=1.9×10^−16^, OR=3.7 (95% CI 2.7 to 5.1); figure 1B, table 2). The significant associations with rs1042331/rs1042335 remained after conditioning on both HLA-DPB1*04:01 and *04:02 (p=3.0×10^−15^, OR=0.17 (95% CI 0.10 to 0.26)), and, additionally, an association with HLA-DPB1*02:01 was exposed (p=3.6×10^−8^, OR=3.5 (95% CI 2.2 to 5.4); table 2). Conditioning on HLA-DPB1*04:01 and rs1042331 revealed one association of statistical significance with SNP rs1042140 (p=8.8×10^−10^, OR=0.26 (95% CI 0.17 to 0.39); figure 1C, table 2). Adding this variant to the list of covariates did not result in any additional significant associations.

**Table 2:** Top genetic associations in the HLA region in PR3-AAV and MPO-AAV, respectively

Trait	Conditioned variant(s)*	Allele†	SNP ID (minor allele)	MAF cases	MAF controls	P value	OR	95% CI
PR3-AAV	0	HLA-DPB1*04:01	–	0.74	0.42	7.1×10^−38^	3.3	2.8 to 4.0
		chr6:33 052 958	rs1042335 (T)	0.038	0.27	6.3×10^−35^	0.089	0.059 to 0.13
		chr6:33 052 950	rs1042331 (C)	0.035	0.27	7.3×10^−35^	0.083	0.055 to 0.12
		HLA-DPB1*03:01	–	0.02	0.076	4.1×10^−8^	0.23	0.13 to 0.37
	HLA-DPB1*04:01	chr6:33 052 950	rs1042331 (C)	0.035	0.27	1.8×10^−21^	0.13	0.082 to 0.19
		chr6:33 052 958	rs1042335 (T)	0.038	0.27	3.7×10^−21^	0.14	0.090 to 0.20
		HLA-DPB1*04:02	–	0.12	0.11	1.9×10^−16^	3.7	2.7 to 5.1
	HLA-DPB1*04:01, HLA-DPB1*04:02	chr6:33 052 950	rs1042331 (C)	0.035	0.27	3.0×10^−15^	0.17	0.10 to 0.26
		chr6:33 052 958	rs1042335 (T)	0.038	0.27	7.8×10^−15^	0.18	0.11 to 0.27
		HLA-DPB1*02:01	–	0.056	0.12	3.6×10^−8^	3.5	2.2 to 5.4
	HLA-DPB1*04:01, chr6:33 052 950	chr6:33 048 640	rs1042140 (G)	0.057	0.21	8.8×10^−10^	0.26	0.17 to 0.39
	HLA-DPB1*04:01, chr6:33052950, chr6:33 048 640	–						
MPO-AAV	0	HLA-DRB1*04:04	–	0.22	0.06	4.5×10^−16^	4.5	3.1 to 6.4
		chr6:32 609 479	rs35874654 (A)	0.44	0.20	4.2×10^−13^	3.8	2.7 to 5.5
		chr6:32 635 954	rs9274619 (A)	0.35	0.14	1.9×10^−12^	3.6	2.5 to 5.1
		HLA-DQB1*03:02	–	0.36	0.14	4.8×10^−11^	3.4	2.3 to 4.8
	HLA-DRB1*04:04	–						

*Variant(s) conditioned for in the association analysis.

†Top two HLA classical alleles and SNPs, respectively, associated with PR3-AAV and MPO-AAV (unadjusted p <5.0 × 10^–8^) after conditioning on the indicated variant(s). SNPs are indicated by genomic position (hg19).

ANCA, antineutrophil cytoplasmic antibody; HLA, human leucocyte antigen; MAF, minor allele frequency; MPO, myeloperoxidase; MPO-AAV, MPO-ANCA positive AAV; PR3, proteinase 3; PR3-AAV, PR3-ANCA positive ANCA-associated vasculitis; SNP, single nucleotide polymorphism.

For comparison, HLA alleles and SNPs were analysed in all patients with GPA regardless of ANCA subtype, against controls. HLA-DPB1*04:01 was the strongest associated allele (p=3.5×10^−35^, OR=3.0 (95% CI 2.5 to 3.5)), followed by the SNPs rs1042331 and rs1042335, but associations were weaker with GPA than with PR3-AAV (online supplemental table 3).

### Genetic analyses of the HLA region in MPO-AAV

Next, classical HLA alleles and SNPs in the HLA region were jointly analysed in MPO-AAV compared with healthy controls. The strongest association was identified for HLA-DRB1*04:04 (p=4.5×10^−16^, OR=4.5 (95% CI 3.1 to 6.4)), followed by rs35874654 (p=4.2×10^−13^, OR=3.8 (95% CI 2.7 to 5.5); figure 1D, table 2). In addition, there were significant associations with HLA-DQB1*03:02 (p=4.8×10^−11^, OR=3.4 (95% CI 2.3 to 4.8)) and 114 additional SNPs (online supplemental table 4). After conditioning on HLA-DRB1*04:04, no significant associations remained (figure 1E, table 2).

When analysing all patients with MPA, HLA-DRB1*04:04 was the strongest associated allele (p=8.6×10^−12^, OR=3.9 (95% CI 2.6 to 5.8)), followed by the SNPs rs34784936 and rs35874654, but with weaker signals of association compared with MPO-AAV (online supplemental table 5).

**Figure 2:**
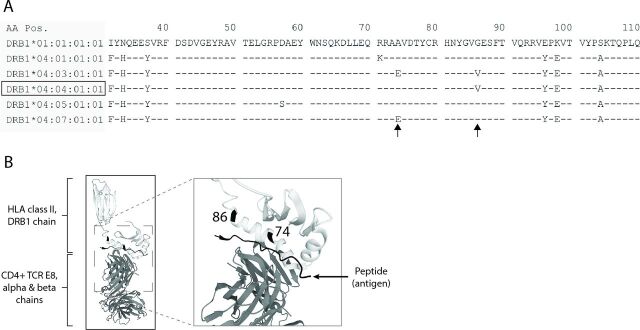
Amino acid residues defining HLA-DRB1*04:04. (A) Alignment of myeloperoxidase (MPO)-antineutrophil cytoplasmic antibody-associated vasculitides (AAV)-associated human leucocyte antigen (HLA) allele DRB1*04:04 against alleles DRB1*04:01, DRB1*04:03, DRB1*04:05, DRB1*04:07 and against DRB1*01:01 (reference), covering amino acids 31-110 of the mature HLA-DRB1 peptide. Amino acids are represented by one-letter abbreviations. Dash sign denotes amino acid equal to reference; deviation from reference is assigned by alternative amino acid. Arrows point to residues distinguishing DRB1*04:04 from the other alleles. AA pos=amino acid position in DRB1 peptide. (B) 3D structures of the HLA-DRB1 chain (bright grey) and CD4+ T cell receptor E8 alpha and beta chains (medium grey), with antigen (triosephosphate isomerase; dark grey) binding to the peptide binding groove. Locations of amino acid residues 74 and 86, distinguishing the HLA-DRB1*04:04 allele, are marked in black. Images are adapted from RCSB Protein data bank, ID 2IAM.

The HLA-DRB1*04:04 allele differs from the three most similar HLA-DRB1*04 alleles DRB1*04:03, *04:05 and *04:07 at one and two amino acid positions in the HLA-DRB1 peptide binding groove, respectively (figure 2A). While DRB1*04:04 and DRB1*04:03 differ at position 74, with alanine (reference) and glutamic acid, respectively, they are both distinct from the other alleles at position 86, with a valine in place of the common glycine (figure 2A,B).

### Analyses of HLA risk alleles in respect to clinical characteristics of AAV

**Figure 3:**
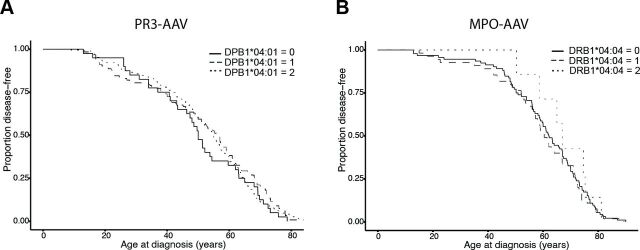
Age at diagnosis and risk of relapse in antineutrophil cytoplasmic antibody-associated vasculitides (AAV), in relation to lead human leucocyte antigen (HLA) variants. The number of risk alleles per patient of HLA-DPB1*04:01 and HLA-DRB1*04:04 was analysed for associations with age at diagnosis of proteinase 3 (PR3)-AAV (A) and myeloperoxidase (MPO)-AAV (B), respectively, and were plotted using Kaplan-Meier curves. Full, dashed and dotted lines correspond to the presence of 0, 1 or 2 risk alleles per patient.

Pulmonary, renal and ENT involvements in the whole AAV population were analysed for associations with HLA alleles, but no significant associations were identified (online supplemental tables 6–8). The strongest associated alleles for ENT involvement were SNPs located in the *HLA-DPB1* region, while the strongest allele for renal involvement was HLA-DRB1*04:04 and for pulmonary involvement SNPs located in the HLA-DM region, between *HLA-DRB1* and *HLA-DPB1*. There were no significant differences in the number of risk alleles of HLA-DPB1*04:01 (in PR3-AAV) or HLA-DRB1*04:04 (in MPO-AAV) carried by patients with versus without each organ involvement, within PR3-AAV and MPO-AAV, respectively (p>0.24; data not shown). Furthermore, there were no significant associations between age at diagnosis and number of HLA-DPB1*04:01 alleles (in PR3-AAV) or HLA-DRB1*04:04 alleles (in MPO-AAV) (p=0.98, HR=0.88, (95% CI 0.84 to 1.2) and p=0.70, HR=0.95 (95% CI 0.72 to 1.2), respectively; figure 3A,B). Likewise, there were no significant associations between HLA-DPB1*04:01/HLA-DRB1*04:04 and relapse in PR3-AAV and MPO-AAV, respectively, although there was a borderline association between a homozygous state of HLA-DPB1*04:01 and relapse in PR3-AAV (p=0.054, OR=2.3 (95% CI 0.99 to 5.3); table 3).

**Table 3:** Association analysis between risk human leucocyte antigen (HLA) alleles and relapse in proteinase 3 (PR3)-antineutrophil cytoplasmic antibody-associated vasculitides (AAV) and myeloperoxidase (MPO)-AAV, respectively

Phenotype	HLA allele	No. of alleles versus 0 alleles	P value	OR	95% CI
PR3-AAV	DPB1*04:01	1	0.16	1.9	0.79 to 4.7
		2	0.054	2.3	0.99 to 5.3
MPO-AAV	DRB1*04:04	1	0.56	1.2	0.60 to 2.6
		2	0.49	0.53	0.071 to 2.9

## Discussion

In this study of a Scandinavian AAV patient population, we identified a novel association between MPO-AAV and HLA-DRB1*04:04 and confirmed a strong association between PR3-AAV and HLA-DPB1*04:01. In addition, we demonstrated that HLA-DPB1*04:01 and the lead *HLA-DPB1* SNP rs1042335 were both associated with PR3-AAV after stepwise conditional analysis.

Associations between PR3-AAV and MPO-AAV and distinct loci in the HLA region have previously been established through SNP analyses, using array-based genotyping and DNA sequencing, respectively.^9–11^ However, as SNPs included on arrays are selected based on their frequency (common), type and ability to tag a region, causality, in terms of functional consequences of disease-associated genetic variation, can rarely be inferred from these associations. In the current study, we analysed classical HLA alleles, providing us the opportunity to identify the HLA genes of relevance and to gain insights into residues of importance for disease in the peptide binding grooves of the HLA molecules.

MPA and MPO-AAV are more prevalent in East Asian populations, as compared with populations of European descent.^1 2^ Accordingly, classical HLA alleles have been investigated in Japanese and Chinese cases with these subtypes of AAV, revealing a genetic predisposition tagged by the HLA-DRB1*09:01-HLA-DQB1*03:03/03:02 haplotypes.^18 20–22^ In contrast, studies of classical HLA alleles in European cases with MPA/MPO-AAV are lacking. In the present study, we identified a strong association between MPO-AAV and HLA-DRB1*04:04, with an allele frequency of 22% in cases and 6% in controls. There is a higher allele frequency of DRB1*04:04 in Caucasian populations (up to 6%) compared with Han Chinese and Japanese populations (<2%; Allele frequency Net Database).^37^ In our study, as suggested by the stepwise conditional analysis, the association with DRB1*04:04 was linked to an association with HLA-DQB1*03:02. The DRB1*04:04-DQB1*03:02 haplotype has a frequency of less than 1% in Han Chinese and Japanese populations and approximately 2.7%–3.9% in populations of European descent, where it is the most common haplotype for DRB1*04:04.^38^ These results emphasise the disparities in genetic predisposition to disease dependent on differences in allele frequencies between populations of different ancestry.

The mechanistic effects of the HLA-DRB1*04:04 allele in relation to disease remain obscure. Two key residues distinguishes the HLA-DRB1*04:04 allele, 74 and 86. Residue 74 is part of the ‘shared epitope’, a five-amino-acid sequence located at the centre of the peptide binding groove, previously associated with rheumatoid arthritis (RA).^39^ Despite extensive studies, the mechanistic role of the shared epitope in RA remains elusive.^40^ At residue 86, a valine is present in HLA-DRB1*04:04, as compared with the reference glycine (present in, for example, HLA-DRB1*04:01), both non-polar amino acids that differ in the size of side chains. Although not located in the centre of the peptide binding groove, this switch in amino acids has been shown to influence peptide binding specificity and affinity of HLA-DRB1.^41 42^

The lead SNP associated with MPO-AAV in the present study, rs35874654, is located in *HLA-DQA1*. Although calculations would suggest a relatively low degree of LD between rs35874654 and HLA-DRB1*04:04 (r^2^ 0.23), this estimation is affected by differences in allele frequencies between the two variants. Instead, stepwise conditional association analysis indicated that rs35874654 and HLA-DRB1*04:04 are linked. The strong LD spanning the HLA-DRB1 – HLA-DQA1 – HLA-DQB1 region and the high rate of genetic variability make it extremely challenging to pin-point functional alleles and key gene(s) affected in MPO-AAV. In an effort to map the effect of genetic variation on gene expression in the HLA region, Kang *et al* 43 performed an extensive expression quantitative trait loci study at single-cell resolution in T cells, B cells and myeloid cells. According to these results, rs35874654 is in strong LD (r^2^>0.8) with 12 SNPs significantly associated with the expression of *HLA-DQA1* in all three cell types (p<1.3 × 10^−34^). Taken together, the association between MPO-AAV and HLA-DRB1*04:04 strongly suggests that the selectivity or affinity of peptide and T cell receptor binding to this particular HLA molecule may contribute to disease development, possibly in combination with perturbed regulation of *HLA-DQA1* expression inflicted by linked genetic variants.

HLA-DRB1*04:04 has previously been associated with other chronic inflammatory disorders. HLA-DRB1*04:04 has been associated with RA, where a particularly strong association with residue 74 was revealed.^44^ An overlap syndrome of AAV and RA has been described, mainly featuring AAV with MPO-ANCA.^45 46^ In light of the current findings, this overlap syndrome may plausibly be an effect of shared genetic predisposition between RA and MPO-AAV. In giant cell arteritis, a vasculitis characterised by inflammation in the wall of medium-sized and large-sized vessels, and with an increased incidence in Northern Europe,^47^ a GWAS identified HLA-DRB1*04:04 as the lead associated genetic variant.^48^ Moreover, Addison’s disease, an organ-specific autoimmune disorder, has also been associated with HLA-DRB1*04:04.^49 50^ Interestingly, both Addison’s disease and MPO-AAV have been associated with SNPs of potentially regulatory function in the *BACH2* gene,^11 24 49^ demonstrating that these two rare autoimmune disorders share at least two genetic susceptibility loci.

We found a strong association between PR3-AAV and HLA-DPB1*04:01, in accordance with previous studies of populations of European descent.^13–17^ When adding DPB1*04:01 as a covariate to the analysis, a significant association with DPB1*04:02 was revealed. Gregersen *et al* 15 demonstrated in a Danish patient cohort that the association between PR3-AAV and *HLA-DPB1* increased when the DPB1*04:01 and DPB1*04:02 alleles were combined (‘HLA-DPB1*04’), compared with an analysis of DPB1*04:01 alone. Furthermore, Gregersen *et al* showed that amino acid residues 69 and 84–87 of HLA-DPB1, shared between DPB1*04:01 and DPB1*04:02, predict PR3-AAV.^15^ These residues constitute essential parts of the HLA-DPB1 peptide binding groove. Interestingly, HLA-DPB1*02:01 shares residues 84–87 with DPB1*04:01, but has a negatively charged glutamic acid at residue 69, instead of the positively charged lysine seen in DPB1*04:01.^15^ In the present study, conditioning on HLA-DPB1*04:01 and *04:02 revealed a significant signal of association with HLA-DPB1*02:01. These findings support a central role for HLA-DPB1 residues 69 and 84–87 for antigen-binding specificity and subsequent T cell activation in the disease development of PR3-AAV.

Gregersen *et al* identified a weak association between PR3-AAV and HLA-DRB1*15,^15^ a finding that was not replicated in the present study. However, similar to Gregersen *et al*,^15^ we could not replicate the significant association between HLA-DPB1*04:01 and relapse rate found in two previous studies.^13 16^ Furthermore, in line with a previous GWAS of AAV,^10^ we did not find significant associations between variants in the HLA region and distinct organ involvements in patients with AAV. In our study, the HLA alleles with strongest suggestive association with organ involvements mirror the prevalence of these organ involvements in PR3-AAV and MPO-AAV, respectively. For instance, ENT involvement, occurring in ~89% of patients with PR3-AAV and ~52% of patients with MPO-AAV,^51^ showed suggestive association with the *HLA-DPB1* locus, implying that the signal of association is linked with the category PR3-ANCA positive AAV rather than the organ involvement itself. Preferably, for a complete dissection of genetic associations with organ involvements in AAV, analyses would be performed in PR3-AAV and MPO-AAV separately. This approach has, however, in this and in previous studies been hampered by limited statistical power when the AAV are divided into subgroups. Combined, these results emphasise the need for deeper studies of plausible associations between genetic risk loci and clinical outcome in AAV in larger cohorts of cases.

In addition to the association with HLA-DPB1*04:01, we detected a significant association between the lead SNPs rs1042331/rs1042335 and PR3-AAV. These SNPs were also the lead SNPs associated with PR3-AAV in our previous SNP analysis of the current dataset.^11^ rs1042331 and rs1042335 are a synonymous and a non-synonymous variant, respectively, located in *HLA-DPB1*. The minor alleles exert a protective effect against PR3-AAV. The SNPs are in strong LD with rs1042169 (r^2^ 0.83, D’ 0.92), one of the lead SNPs in a previous GWAS of PR3-AAV and also located in *HLA-DPB1*.^10^ The rs1042169 risk allele was shown to be significantly associated with lower *HLA-DPB1* gene expression in blood cells and lower HLA-DP protein expression on B cells and monocytes.^10^ Likewise, rs9277534, located in *HLA-DPB1* and in LD with rs1042331/rs1042335 (r^2^ 1.0, D’ 1.0), has been associated with graft-versus-host disease, where the risk allele (linked to the protective allele of rs1042335) was shown to be strongly associated with increased *HLA-DPB1* transcript levels.^52^ In addition, 32 SNPs in perfect LD with rs1042331/rs1042335 (r^2^ 1.0, D’ 1.0) were identified as significant eQTL SNPs for *HLA-DPB1* in T cells and B cells in the dataset by Kang *et al*.^53^ In contrast, Chen *et al* could not detect an association between HLA-DPB1*04:01 and *HLA-DPB1* gene or protein expression in leukocytes.^16^ Taken together, our results indicate that PR3-AAV is associated with two distinct genetic elements in the HLD-DP region, with two different modes of action: one associated with specific amino acid residues affecting the affinity of peptide binding of HLA-DP, and one associated with reduced expression levels of *HLA-DPB1*.

There are limitations to this study. The AAV are rare disorders, and hence, the sample size of the AAV population of this study is modest. Moreover, the novel findings of the HLA allele associations with MPO-AAV were not replicated in an independent cohort in the current study and thus require further validation. While previous GWAS of AAV, as well as our previous targeted sequencing study of AAV, have identified disease-associated variants also in the *SERPINA1, PRTN3* and *BACH2* genes,^9–11^ the current study focused on the HLA region. This approach provided novel insights into the role of HLA genes in AAV heritability, but did not enable the identification of additional AAV-associated genes.

In conclusion, through a combined analysis of SNPs and classical HLA alleles, we have identified a novel association for MPO-AAV with HLA-DRB1*04:04 and have shed further light on two signals of association within the HLA-DP locus in PR3-AAV.

### Web resources

IPD-IMGT/HLA: https://www.ebi.ac.uk/ipd/imgt/hla/

RCSB Protein data bank: https://www.rcsb.org/

LDlink: https://ldlink.nci.nih.gov/

Allele frequency Net Database: https://allelefrequencies.net/

## Data Availability

Data are available upon reasonable request. Requests for data should be directed to the senior author but will be conditioned on the legal premises under which they were collected.
