# Supplementary material for: The HLA region in ANCA-associated vasculitis: characterisation of genetic associations in a Scandinavian patient population
Source: RMD Open. 2024 Apr 4;10(2):e004039. doi: 10.1136/rmdopen-2023-004039 (PMC11002376; doi:10.1136/rmdopen-2023-004039)
Supplement: Supplementary data [file rmdopen-2023-004039supp001.pdf]

Supplementary table 1. Call rates of HLA alleles per gene, using xHLA, in controls and in cases with PR3-ANCA and MPO-ANCA, respectively.

| | | | Controls | | PR3-AAV | | MPO-AAV | | Call rate | | | |
|---|---|---|---|---|---|---|---|---|---|---|---|---|
| **HLA gene** | **Total no. of alleles** | **No. of called alleles** | **Total no. of alleles** | **No. of called alleles** | **Total no. of alleles** | **No. of called alleles** | **Total no. of alleles** | **No. of called alleles** | **All** | **Controls** | **PR3-AAV** | **MPO-AAV** |
| A | 2168 | 2083 | 1595 | 1580 | 411 | 356 | 162 | 147 | 96.1% | 99.1% | 86.6% | 90.7% |
| B | 2168 | 2134 | 1595 | 1580 | 411 | 395 | 162 | 159 | 98.4% | 99.1% | 96.1% | 98.1% |
| C | 2168 | 2124 | 1595 | 1580 | 411 | 391 | 162 | 153 | 98.0% | 99.1% | 95.1% | 94.4% |
| DPB1 | 2168 | 2139 | 1595 | 1580 | 411 | 399 | 162 | 160 | 98.7% | 99.1% | 97.1% | 98.8% |
| DQB1 | 2168 | 2057 | 1595 | 1580 | 411 | 341 | 162 | 136 | 94.9% | 99.1% | 83.0% | 84.0% |
| DRB1 | 2168 | 2121 | 1595 | 1580 | 411 | 386 | 162 | 155 | 97.8% | 99.1% | 93.9% | 95.7% |

PR3 = proteinase 3; MPO = myeloperoxidase; ANCA = anti-neutrophil cytoplasmic antibody; PR3-AAV = PR3-ANCA positive ANCA-associated vasculitis; MPO-AAV = MPO-ANCA positive AAV.

**Supplemtary Table 2**. Association analysis of PR3-ANCA+ AAV patients versus controls, adjusting for sex and genetic structure (principal components 1-4).

| Variant | position | OR | CI_lower | CI_upper | p | MAF | MAF_cases | MAF_controls | Type (SNP, HLA (xHLA)) |
|---|---|---|---|---|---|---|---|---|---|
| xHLA_2field_DPB1_04_01 | 33043767 | 3.32 | 2.78 | 4.00 | 7.07E-38 | 0.48 | 0.74 | 0.42 | HLA (xHLA) |
| chr6_33052958_C_T | 33052958 | 0.09 | 0.06 | 0.13 | 6.30E-35 | 0.23 | 0.04 | 0.27 | SNP |
| chr6_33052950_T_C | 33052950 | 0.08 | 0.05 | 0.12 | 7.29E-35 | 0.22 | 0.04 | 0.27 | SNP |
| chr6_33052986_T_C | 33052986 | 0.09 | 0.06 | 0.13 | 1.29E-34 | 0.23 | 0.04 | 0.28 | SNP |
| chr6_33053455_C_T | 33053455 | 0.09 | 0.06 | 0.13 | 1.48E-34 | 0.23 | 0.04 | 0.28 | SNP |
| chr6_33054046_T_G | 33054046 | 0.09 | 0.06 | 0.13 | 1.77E-34 | 0.23 | 0.04 | 0.28 | SNP |
| chr6_33054071_G_C | 33054071 | 0.09 | 0.06 | 0.13 | 1.90E-34 | 0.23 | 0.04 | 0.28 | SNP |
| chr6_33053877_G_A | 33053877 | 0.09 | 0.06 | 0.13 | 1.91E-34 | 0.23 | 0.04 | 0.28 | SNP |
| chr6_33053887_A_G | 33053887 | 0.09 | 0.06 | 0.13 | 2.09E-34 | 0.23 | 0.04 | 0.28 | SNP |
| chr6_33053682_G_A | 33053682 | 0.09 | 0.06 | 0.13 | 2.09E-34 | 0.23 | 0.04 | 0.28 | SNP |
| chr6_33054057_G_A | 33054057 | 0.09 | 0.06 | 0.13 | 2.10E-34 | 0.23 | 0.04 | 0.28 | SNP |
| chr6_33053871_A_G | 33053871 | 0.09 | 0.06 | 0.13 | 2.14E-34 | 0.23 | 0.04 | 0.28 | SNP |
| chr6_33053890_G_A | 33053890 | 0.09 | 0.06 | 0.13 | 2.19E-34 | 0.23 | 0.04 | 0.28 | SNP |
| chr6_33054152_A_G | 33054152 | 0.09 | 0.06 | 0.13 | 2.22E-34 | 0.23 | 0.04 | 0.28 | SNP |
| chr6_33053968_T_A | 33053968 | 0.09 | 0.06 | 0.13 | 2.29E-34 | 0.23 | 0.04 | 0.28 | SNP |
| chr6_33053971_T_C | 33053971 | 0.09 | 0.06 | 0.13 | 2.29E-34 | 0.23 | 0.04 | 0.28 | SNP |
| chr6_33053728_C_T | 33053728 | 0.09 | 0.06 | 0.13 | 2.29E-34 | 0.23 | 0.04 | 0.28 | SNP |
| chr6_33054149_T_C | 33054149 | 0.09 | 0.06 | 0.13 | 2.31E-34 | 0.23 | 0.04 | 0.28 | SNP |
| chr6_33053477_G_C | 33053477 | 0.09 | 0.06 | 0.13 | 2.34E-34 | 0.23 | 0.04 | 0.28 | SNP |
| chr6_33053468_G_T | 33053468 | 0.09 | 0.06 | 0.13 | 2.35E-34 | 0.23 | 0.04 | 0.28 | SNP |
| chr6_33055009_C_A | 33055009 | 0.09 | 0.06 | 0.13 | 2.36E-34 | 0.23 | 0.04 | 0.28 | SNP |
| chr6_33054235_T_G | 33054235 | 0.09 | 0.06 | 0.13 | 2.40E-34 | 0.23 | 0.04 | 0.28 | SNP |
| chr6_33053772_G_A | 33053772 | 0.09 | 0.06 | 0.13 | 2.40E-34 | 0.23 | 0.04 | 0.28 | SNP |
| chr6_33053982_G_C | 33053982 | 0.09 | 0.06 | 0.13 | 2.41E-34 | 0.23 | 0.04 | 0.28 | SNP |
| chr6_33054353_G_A | 33054353 | 0.09 | 0.06 | 0.13 | 2.43E-34 | 0.23 | 0.04 | 0.28 | SNP |
| chr6_33053868_A_G | 33053868 | 0.09 | 0.06 | 0.13 | 2.44E-34 | 0.23 | 0.04 | 0.28 | SNP |
| chr6_33054148_C_T | 33054148 | 0.09 | 0.06 | 0.13 | 2.45E-34 | 0.23 | 0.04 | 0.28 | SNP |
| chr6_33054142_G_C | 33054142 | 0.09 | 0.06 | 0.13 | 2.48E-34 | 0.23 | 0.04 | 0.28 | SNP |
| chr6_33054656_A_C | 33054656 | 0.09 | 0.06 | 0.13 | 2.50E-34 | 0.23 | 0.04 | 0.28 | SNP |
| chr6_33054675_C_G | 33054675 | 0.09 | 0.06 | 0.13 | 2.50E-34 | 0.23 | 0.04 | 0.28 | SNP |
| chr6_33054683_A_G | 33054683 | 0.09 | 0.06 | 0.13 | 2.50E-34 | 0.23 | 0.04 | 0.28 | SNP |
| chr6_33054687_G_A | 33054687 | 0.09 | 0.06 | 0.13 | 2.50E-34 | 0.23 | 0.04 | 0.28 | SNP |
| chr6_33054091_C_T | 33054091 | 0.09 | 0.06 | 0.13 | 2.50E-34 | 0.23 | 0.04 | 0.28 | SNP |
| chr6_33053780_T_A | 33053780 | 0.09 | 0.06 | 0.13 | 2.51E-34 | 0.23 | 0.04 | 0.28 | SNP |
| chr6_33053723_T_C | 33053723 | 0.09 | 0.06 | 0.13 | 2.53E-34 | 0.23 | 0.04 | 0.28 | SNP |
| chr6_33054711_G_T | 33054711 | 0.09 | 0.06 | 0.13 | 2.57E-34 | 0.23 | 0.04 | 0.28 | SNP |
| chr6_33054721_C_T | 33054721 | 0.09 | 0.06 | 0.13 | 2.57E-34 | 0.23 | 0.04 | 0.28 | SNP |
| chr6_33054141_T_C | 33054141 | 0.09 | 0.06 | 0.13 | 2.58E-34 | 0.23 | 0.04 | 0.28 | SNP |
| chr6_33053789_A_G | 33053789 | 0.09 | 0.06 | 0.13 | 2.61E-34 | 0.23 | 0.04 | 0.28 | SNP |
| chr6_33054457_A_G | 33054457 | 0.09 | 0.06 | 0.13 | 2.61E-34 | 0.23 | 0.04 | 0.28 | SNP |
| chr6_33054579_C_T | 33054579 | 0.09 | 0.06 | 0.13 | 2.61E-34 | 0.23 | 0.04 | 0.28 | SNP |
| chr6_33054595_C_T | 33054595 | 0.09 | 0.06 | 0.13 | 2.61E-34 | 0.23 | 0.04 | 0.28 | SNP |
| chr6_33054619_G_A | 33054619 | 0.09 | 0.06 | 0.13 | 2.61E-34 | 0.23 | 0.04 | 0.28 | SNP |
| chr6_33054890_C_T | 33054890 | 0.09 | 0.06 | 0.13 | 2.61E-34 | 0.23 | 0.04 | 0.28 | SNP |
| chr6_33054342_T_A | 33054342 | 0.09 | 0.06 | 0.13 | 2.63E-34 | 0.23 | 0.04 | 0.28 | SNP |
| chr6_33053902_A_G | 33053902 | 0.09 | 0.06 | 0.13 | 2.63E-34 | 0.23 | 0.04 | 0.28 | SNP |
| chr6_33054355_A_C | 33054355 | 0.09 | 0.06 | 0.13 | 2.64E-34 | 0.23 | 0.04 | 0.28 | SNP |
| chr6_33054586_A_G | 33054586 | 0.09 | 0.06 | 0.13 | 2.65E-34 | 0.23 | 0.04 | 0.28 | SNP |
| chr6_33054155_C_T | 33054155 | 0.09 | 0.06 | 0.13 | 2.67E-34 | 0.23 | 0.04 | 0.28 | SNP |
| chr6_33054156_A_G | 33054156 | 0.09 | 0.06 | 0.13 | 2.67E-34 | 0.23 | 0.04 | 0.28 | SNP |
| chr6_33054400_T_C | 33054400 | 0.09 | 0.06 | 0.13 | 2.67E-34 | 0.23 | 0.04 | 0.28 | SNP |
| chr6_33054404_A_G | 33054404 | 0.09 | 0.06 | 0.13 | 2.67E-34 | 0.23 | 0.04 | 0.28 | SNP |
| chr6_33054410_A_C | 33054410 | 0.09 | 0.06 | 0.13 | 2.67E-34 | 0.23 | 0.04 | 0.28 | SNP |
| chr6_33054331_T_C | 33054331 | 0.09 | 0.06 | 0.13 | 2.68E-34 | 0.23 | 0.04 | 0.28 | SNP |
| chr6_33054550_G_A | 33054550 | 0.09 | 0.06 | 0.13 | 2.68E-34 | 0.23 | 0.04 | 0.28 | SNP |
| chr6_33054386_C_T | 33054386 | 0.09 | 0.06 | 0.13 | 2.75E-34 | 0.23 | 0.04 | 0.28 | SNP |
| chr6_33053906_G_T | 33053906 | 0.09 | 0.06 | 0.13 | 2.77E-34 | 0.23 | 0.04 | 0.28 | SNP |
| chr6_33054381_T_C | 33054381 | 0.09 | 0.06 | 0.13 | 2.80E-34 | 0.23 | 0.04 | 0.28 | SNP |
| chr6_33054807_A_G | 33054807 | 0.09 | 0.06 | 0.13 | 2.86E-34 | 0.23 | 0.04 | 0.28 | SNP |
| chr6_33054373_C_T | 33054373 | 0.09 | 0.06 | 0.13 | 2.88E-34 | 0.23 | 0.04 | 0.28 | SNP |
| chr6_33054325_G_C | 33054325 | 0.09 | 0.06 | 0.13 | 2.91E-34 | 0.23 | 0.04 | 0.28 | SNP |
| chr6_33054302_T_C | 33054302 | 0.09 | 0.06 | 0.13 | 2.95E-34 | 0.23 | 0.04 | 0.28 | SNP |
| chr6_33054413_T_G | 33054413 | 0.09 | 0.06 | 0.13 | 3.04E-34 | 0.23 | 0.04 | 0.28 | SNP |
| chr6_33054414_T_A | 33054414 | 0.09 | 0.06 | 0.13 | 3.04E-34 | 0.23 | 0.04 | 0.28 | SNP |
| chr6_33054231_C_T | 33054231 | 0.09 | 0.06 | 0.13 | 3.16E-34 | 0.23 | 0.04 | 0.28 | SNP |
| chr6_33053399_T_C | 33053399 | 0.09 | 0.06 | 0.13 | 3.19E-34 | 0.23 | 0.04 | 0.28 | SNP |
| chr6_33054268_C_A | 33054268 | 0.09 | 0.06 | 0.13 | 3.20E-34 | 0.23 | 0.04 | 0.28 | SNP |
| chr6_33047432_A_C | 33047432 | 0.10 | 0.07 | 0.14 | 3.30E-34 | 0.23 | 0.04 | 0.28 | SNP |
| chr6_33054280_T_C | 33054280 | 0.09 | 0.06 | 0.13 | 4.03E-34 | 0.23 | 0.04 | 0.28 | SNP |
| chr6_33054281_G_A | 33054281 | 0.09 | 0.06 | 0.13 | 4.03E-34 | 0.23 | 0.04 | 0.28 | SNP |
| chr6_33049211_C_T | 33049211 | 0.10 | 0.07 | 0.15 | 7.80E-34 | 0.23 | 0.04 | 0.28 | SNP |
| chr6_33055047_A_G | 33055047 | 0.09 | 0.06 | 0.13 | 9.98E-34 | 0.23 | 0.04 | 0.28 | SNP |
| chr6_33053167_A_C | 33053167 | 0.09 | 0.06 | 0.13 | 1.31E-33 | 0.23 | 0.04 | 0.28 | SNP |
| chr6_33050125_G_A | 33050125 | 0.09 | 0.06 | 0.14 | 1.43E-33 | 0.22 | 0.04 | 0.27 | SNP |
| chr6_33050078_A_G | 33050078 | 0.09 | 0.06 | 0.14 | 1.48E-33 | 0.22 | 0.04 | 0.27 | SNP |
| chr6_33050089_G_A | 33050089 | 0.09 | 0.06 | 0.14 | 1.54E-33 | 0.22 | 0.04 | 0.27 | SNP |
| chr6_33050107_G_A | 33050107 | 0.09 | 0.06 | 0.14 | 1.58E-33 | 0.22 | 0.04 | 0.27 | SNP |
| chr6_33050079_C_G | 33050079 | 0.09 | 0.06 | 0.14 | 1.60E-33 | 0.22 | 0.04 | 0.27 | SNP |
| chr6_33050118_C_T | 33050118 | 0.09 | 0.06 | 0.14 | 1.85E-33 | 0.22 | 0.04 | 0.27 | SNP |
| chr6_33050148_T_C | 33050148 | 0.09 | 0.06 | 0.14 | 1.97E-33 | 0.22 | 0.04 | 0.27 | SNP |
| chr6_33050168_C_A | 33050168 | 0.10 | 0.07 | 0.14 | 2.47E-33 | 0.22 | 0.04 | 0.27 | SNP |
| chr6_33050024_C_A | 33050024 | 0.10 | 0.07 | 0.14 | 2.62E-33 | 0.22 | 0.04 | 0.27 | SNP |
| chr6_33050179_A_G | 33050179 | 0.10 | 0.07 | 0.14 | 2.69E-33 | 0.22 | 0.04 | 0.27 | SNP |
| chr6_33050203_C_G | 33050203 | 0.10 | 0.07 | 0.15 | 2.76E-33 | 0.22 | 0.04 | 0.27 | SNP |
| chr6_33050196_G_C | 33050196 | 0.10 | 0.07 | 0.15 | 2.86E-33 | 0.22 | 0.04 | 0.27 | SNP |
| chr6_33050045_T_C | 33050045 | 0.10 | 0.07 | 0.14 | 2.88E-33 | 0.22 | 0.04 | 0.27 | SNP |
| chr6_33050185_C_A | 33050185 | 0.10 | 0.07 | 0.14 | 2.99E-33 | 0.22 | 0.04 | 0.27 | SNP |
| chr6_33050186_T_C | 33050186 | 0.10 | 0.07 | 0.14 | 2.99E-33 | 0.22 | 0.04 | 0.27 | SNP |
| chr6_33050279_A_G | 33050279 | 0.10 | 0.07 | 0.14 | 3.72E-33 | 0.23 | 0.04 | 0.27 | SNP |
| chr6_33050473_A_G | 33050473 | 0.10 | 0.06 | 0.14 | 4.51E-33 | 0.22 | 0.04 | 0.27 | SNP |
| chr6_33049979_A_G | 33049979 | 0.11 | 0.07 | 0.15 | 4.70E-33 | 0.23 | 0.04 | 0.27 | SNP |
| chr6_33049983_C_T | 33049983 | 0.11 | 0.07 | 0.15 | 5.20E-33 | 0.23 | 0.04 | 0.27 | SNP |
| chr6_33050499_A_G | 33050499 | 0.10 | 0.06 | 0.14 | 5.22E-33 | 0.22 | 0.04 | 0.27 | SNP |
| chr6_33053307_C_T | 33053307 | 0.09 | 0.06 | 0.14 | 1.27E-32 | 0.23 | 0.04 | 0.28 | SNP |
| chr6_33050525_T_C | 33050525 | 0.10 | 0.07 | 0.14 | 1.40E-32 | 0.22 | 0.04 | 0.27 | SNP |
| chr6_33050526_C_T | 33050526 | 0.10 | 0.07 | 0.14 | 1.60E-32 | 0.22 | 0.04 | 0.27 | SNP |
| chr6_33053270_T_A | 33053270 | 0.08 | 0.05 | 0.12 | 1.78E-32 | 0.23 | 0.03 | 0.28 | SNP |
| chr6_33055123_A_G | 33055123 | 0.09 | 0.06 | 0.13 | 1.96E-32 | 0.23 | 0.04 | 0.28 | SNP |
| chr6_33050521_G_T | 33050521 | 0.10 | 0.07 | 0.14 | 2.16E-32 | 0.22 | 0.04 | 0.27 | SNP |
| chr6_33053271_T_C | 33053271 | 0.08 | 0.05 | 0.12 | 2.59E-32 | 0.23 | 0.03 | 0.28 | SNP |
| chr6_33053252_G_A | 33053252 | 0.08 | 0.05 | 0.12 | 3.24E-32 | 0.23 | 0.03 | 0.28 | SNP |
| chr6_33047031_T_C | 33047031 | 0.10 | 0.07 | 0.15 | 3.39E-32 | 0.22 | 0.04 | 0.27 | SNP |
| chr6_33054659_A_G | 33054659 | 0.10 | 0.07 | 0.14 | 3.49E-32 | 0.22 | 0.04 | 0.26 | SNP |
| chr6_33053248_A_G | 33053248 | 0.08 | 0.05 | 0.12 | 3.59E-32 | 0.23 | 0.03 | 0.28 | SNP |
| chr6_33055079_A_G | 33055079 | 0.10 | 0.07 | 0.15 | 2.07E-31 | 0.21 | 0.04 | 0.26 | SNP |
| chr6_33050232_T_G | 33050232 | 0.11 | 0.07 | 0.15 | 8.33E-31 | 0.21 | 0.04 | 0.25 | SNP |
| chr6_33050223_G_A | 33050223 | 0.11 | 0.08 | 0.16 | 8.55E-31 | 0.21 | 0.04 | 0.25 | SNP |
| chr6_33048459_T_G | 33048459 | 0.11 | 0.07 | 0.15 | 2.68E-30 | 0.19 | 0.04 | 0.23 | SNP |
| chr6_33048461_T_A | 33048461 | 0.11 | 0.07 | 0.15 | 3.07E-30 | 0.19 | 0.04 | 0.23 | SNP |
| chr6_33048457_C_G | 33048457 | 0.11 | 0.07 | 0.16 | 4.31E-30 | 0.19 | 0.04 | 0.23 | SNP |
| chr6_33071754_C_A | 33071754 | 0.10 | 0.06 | 0.14 | 6.51E-28 | 0.19 | 0.03 | 0.23 | SNP |
| chr6_33071777_A_G | 33071777 | 0.10 | 0.06 | 0.15 | 1.51E-27 | 0.19 | 0.04 | 0.23 | SNP |
| chr6_33039625_T_C | 33039625 | 0.20 | 0.15 | 0.27 | 2.40E-27 | 0.24 | 0.07 | 0.28 | SNP |
| chr6_33071708_G_A | 33071708 | 0.10 | 0.06 | 0.15 | 2.86E-27 | 0.19 | 0.03 | 0.23 | SNP |
| chr6_33071600_C_A | 33071600 | 0.10 | 0.06 | 0.15 | 4.48E-27 | 0.19 | 0.03 | 0.23 | SNP |
| chr6_33054861_A_G | 33054861 | 0.09 | 0.06 | 0.14 | 1.21E-26 | 0.18 | 0.03 | 0.22 | SNP |
| chr6_33053942_T_C | 33053942 | 0.09 | 0.06 | 0.14 | 1.28E-26 | 0.18 | 0.03 | 0.22 | SNP |
| chr6_33053812_A_G | 33053812 | 0.09 | 0.06 | 0.14 | 1.31E-26 | 0.18 | 0.03 | 0.22 | SNP |
| chr6_33053396_A_G | 33053396 | 0.09 | 0.06 | 0.14 | 1.84E-26 | 0.18 | 0.03 | 0.22 | SNP |
| chr6_33048542_C_T | 33048542 | 0.33 | 0.27 | 0.41 | 2.28E-26 | 0.41 | 0.22 | 0.46 | SNP |
| chr6_33053352_C_T | 33053352 | 0.09 | 0.06 | 0.14 | 2.57E-26 | 0.18 | 0.03 | 0.22 | SNP |
| chr6_33143756_A_G | 33143756 | 0.16 | 0.11 | 0.22 | 3.30E-25 | 0.18 | 0.05 | 0.22 | SNP |
| chr6_33054074_C_T | 33054074 | 0.10 | 0.06 | 0.15 | 7.86E-25 | 0.16 | 0.03 | 0.20 | SNP |
| chr6_33047466_T_C | 33047466 | 0.10 | 0.06 | 0.15 | 8.07E-25 | 0.17 | 0.03 | 0.20 | SNP |
| chr6_33166034_A_G | 33166034 | 0.16 | 0.11 | 0.22 | 8.31E-24 | 0.18 | 0.05 | 0.22 | SNP |
| chr6_33178010_G_T | 33178010 | 0.17 | 0.12 | 0.23 | 1.03E-23 | 0.19 | 0.05 | 0.22 | SNP |
| chr6_33160425_G_T | 33160425 | 0.15 | 0.11 | 0.22 | 1.03E-23 | 0.17 | 0.04 | 0.20 | SNP |
| chr6_33174783_A_G | 33174783 | 0.26 | 0.19 | 0.34 | 2.28E-22 | 0.22 | 0.09 | 0.25 | SNP |
| chr6_33048599_C_A | 33048599 | 0.34 | 0.27 | 0.42 | 5.37E-22 | 0.40 | 0.22 | 0.44 | SNP |
| chr6_33048466_G_T | 33048466 | 0.11 | 0.06 | 0.16 | 9.68E-22 | 0.14 | 0.03 | 0.17 | SNP |
| chr6_33048467_G_T | 33048467 | 0.11 | 0.07 | 0.16 | 1.08E-21 | 0.14 | 0.03 | 0.17 | SNP |
| chr6_33046742_T_C | 33046742 | 0.11 | 0.07 | 0.17 | 2.40E-21 | 0.15 | 0.03 | 0.18 | SNP |
| chr6_33048663_G_A | 33048663 | 0.13 | 0.08 | 0.20 | 5.19E-21 | 0.18 | 0.04 | 0.21 | SNP |
| chr6_33095615_T_C | 33095615 | 0.36 | 0.29 | 0.45 | 1.58E-20 | 0.32 | 0.18 | 0.36 | SNP |
| chr6_33050084_C_T | 33050084 | 0.11 | 0.06 | 0.17 | 7.01E-20 | 0.13 | 0.02 | 0.16 | SNP |
| chr6_33050144_C_T | 33050144 | 0.11 | 0.06 | 0.17 | 8.19E-20 | 0.13 | 0.02 | 0.16 | SNP |
| chr6_33050394_G_A | 33050394 | 0.11 | 0.07 | 0.17 | 9.57E-20 | 0.13 | 0.02 | 0.16 | SNP |
| chr6_33050325_A_C | 33050325 | 0.11 | 0.07 | 0.17 | 9.98E-20 | 0.13 | 0.02 | 0.16 | SNP |
| chr6_33050463_C_G | 33050463 | 0.10 | 0.06 | 0.16 | 1.11E-19 | 0.13 | 0.02 | 0.16 | SNP |
| chr6_33050036_T_C | 33050036 | 0.11 | 0.07 | 0.18 | 1.11E-19 | 0.13 | 0.02 | 0.16 | SNP |
| chr6_33054145_A_G | 33054145 | 0.11 | 0.07 | 0.17 | 1.27E-19 | 0.13 | 0.02 | 0.16 | SNP |
| chr6_33095636_A_G | 33095636 | 0.38 | 0.31 | 0.47 | 1.33E-19 | 0.32 | 0.19 | 0.36 | SNP |
| chr6_33095801_C_T | 33095801 | 0.37 | 0.30 | 0.46 | 1.37E-19 | 0.32 | 0.18 | 0.35 | SNP |
| chr6_33095684_A_G | 33095684 | 0.38 | 0.31 | 0.47 | 1.38E-19 | 0.32 | 0.19 | 0.36 | SNP |
| chr6_33045208_T_C | 33045208 | 0.40 | 0.32 | 0.48 | 1.71E-19 | 0.36 | 0.19 | 0.40 | SNP |
| chr6_33046915_T_A | 33046915 | 0.11 | 0.07 | 0.18 | 3.91E-19 | 0.13 | 0.02 | 0.16 | SNP |
| chr6_33046726_T_C | 33046726 | 0.11 | 0.07 | 0.18 | 4.23E-19 | 0.13 | 0.02 | 0.16 | SNP |
| chr6_33095737_G_A | 33095737 | 0.39 | 0.31 | 0.47 | 6.19E-19 | 0.32 | 0.19 | 0.36 | SNP |
| chr6_33047612_G_C | 33047612 | 0.11 | 0.07 | 0.18 | 8.22E-19 | 0.13 | 0.02 | 0.15 | SNP |
| chr6_33095836_G_A | 33095836 | 0.38 | 0.31 | 0.47 | 1.10E-18 | 0.32 | 0.19 | 0.35 | SNP |

| | | | | | | | | | |
|---|---|---|---|---|---|---|---|---|---|
| chr6_33048661_A_G | 33048661 | 0.15 | 0.09 | 0.22 | 1.15E-18 | 0.16 | 0.04 | 0.19 | SNP |
| chr6_33045255_T_C | 33045255 | 0.41 | 0.33 | 0.50 | 1.18E-18 | 0.36 | 0.20 | 0.40 | SNP |
| chr6_33095748_C_G | 33095748 | 0.39 | 0.32 | 0.48 | 2.49E-18 | 0.32 | 0.19 | 0.35 | SNP |
| chr6_33037419_G_A | 33037419 | 0.11 | 0.06 | 0.17 | 6.66E-18 | 0.12 | 0.02 | 0.14 | SNP |
| chr6_33037424_T_C | 33037424 | 0.11 | 0.06 | 0.17 | 7.03E-18 | 0.12 | 0.02 | 0.14 | SNP |
| chr6_33095516_G_A | 33095516 | 0.37 | 0.29 | 0.46 | 1.23E-17 | 0.32 | 0.18 | 0.36 | SNP |
| chr6_33037304_G_T | 33037304 | 0.11 | 0.06 | 0.18 | 1.65E-17 | 0.12 | 0.02 | 0.15 | SNP |
| chr6_33037279_A_G | 33037279 | 0.11 | 0.07 | 0.18 | 1.91E-17 | 0.12 | 0.02 | 0.15 | SNP |
| chr6_33037294_T_C | 33037294 | 0.11 | 0.06 | 0.18 | 1.98E-17 | 0.12 | 0.02 | 0.15 | SNP |
| chr6_33037187_C_G | 33037187 | 0.12 | 0.07 | 0.19 | 2.10E-17 | 0.13 | 0.02 | 0.15 | SNP |
| chr6_33033058_T_C | 33033058 | 0.11 | 0.07 | 0.18 | 2.15E-17 | 0.12 | 0.02 | 0.15 | SNP |
| chr6_33048640_A_G | 33048640 | 0.22 | 0.15 | 0.31 | 2.33E-17 | 0.18 | 0.06 | 0.21 | SNP |
| chr6_33036951_A_G | 33036951 | 0.12 | 0.07 | 0.19 | 2.38E-17 | 0.12 | 0.02 | 0.14 | SNP |
| chr6_33036959_G_A | 33036959 | 0.12 | 0.07 | 0.19 | 2.43E-17 | 0.12 | 0.02 | 0.14 | SNP |
| chr6_33036312_A_G | 33036312 | 0.12 | 0.07 | 0.19 | 2.47E-17 | 0.12 | 0.02 | 0.15 | SNP |
| chr6_33036287_G_A | 33036287 | 0.12 | 0.07 | 0.19 | 2.49E-17 | 0.12 | 0.02 | 0.15 | SNP |
| chr6_33036505_G_C | 33036505 | 0.12 | 0.07 | 0.19 | 2.75E-17 | 0.13 | 0.02 | 0.15 | SNP |
| chr6_33036288_G_A | 33036288 | 0.12 | 0.07 | 0.19 | 2.82E-17 | 0.12 | 0.02 | 0.15 | SNP |
| chr6_33036233_A_G | 33036233 | 0.12 | 0.07 | 0.19 | 2.84E-17 | 0.12 | 0.02 | 0.15 | SNP |
| chr6_33036242_T_C | 33036242 | 0.12 | 0.07 | 0.19 | 2.86E-17 | 0.12 | 0.02 | 0.15 | SNP |
| chr6_33036617_C_T | 33036617 | 0.12 | 0.07 | 0.19 | 2.91E-17 | 0.13 | 0.02 | 0.15 | SNP |
| chr6_33036630_G_T | 33036630 | 0.12 | 0.07 | 0.19 | 2.95E-17 | 0.13 | 0.02 | 0.15 | SNP |
| chr6_33036631_A_C | 33036631 | 0.12 | 0.07 | 0.19 | 2.95E-17 | 0.13 | 0.02 | 0.15 | SNP |
| chr6_33036177_A_G | 33036177 | 0.12 | 0.07 | 0.19 | 2.98E-17 | 0.12 | 0.02 | 0.15 | SNP |
| chr6_33036401_C_T | 33036401 | 0.13 | 0.08 | 0.20 | 3.01E-17 | 0.13 | 0.02 | 0.15 | SNP |
| chr6_33036210_G_T | 33036210 | 0.12 | 0.07 | 0.19 | 3.03E-17 | 0.12 | 0.02 | 0.15 | SNP |
| chr6_33033170_T_C | 33033170 | 0.12 | 0.07 | 0.19 | 3.06E-17 | 0.12 | 0.02 | 0.15 | SNP |
| chr6_33033202_C_T | 33033202 | 0.12 | 0.07 | 0.19 | 3.09E-17 | 0.12 | 0.02 | 0.15 | SNP |
| chr6_33036717_C_G | 33036717 | 0.12 | 0.07 | 0.19 | 3.22E-17 | 0.12 | 0.02 | 0.15 | SNP |
| chr6_33036394_G_A | 33036394 | 0.12 | 0.07 | 0.19 | 3.31E-17 | 0.12 | 0.02 | 0.15 | SNP |
| chr6_33033146_T_C | 33033146 | 0.12 | 0.07 | 0.19 | 3.33E-17 | 0.12 | 0.02 | 0.15 | SNP |
| chr6_33033235_G_C | 33033235 | 0.12 | 0.07 | 0.19 | 3.35E-17 | 0.13 | 0.02 | 0.15 | SNP |
| chr6_33037522_T_C | 33037522 | 0.12 | 0.07 | 0.18 | 3.44E-17 | 0.12 | 0.02 | 0.15 | SNP |
| chr6_33037142_A_G | 33037142 | 0.12 | 0.07 | 0.19 | 3.46E-17 | 0.13 | 0.02 | 0.15 | SNP |
| chr6_33037675_C_A | 33037675 | 0.12 | 0.07 | 0.18 | 3.46E-17 | 0.12 | 0.02 | 0.15 | SNP |
| chr6_33036388_C_T | 33036388 | 0.12 | 0.07 | 0.19 | 3.51E-17 | 0.12 | 0.02 | 0.15 | SNP |
| chr6_33037690_C_T | 33037690 | 0.12 | 0.07 | 0.18 | 3.57E-17 | 0.12 | 0.02 | 0.15 | SNP |
| chr6_33036853_A_C | 33036853 | 0.12 | 0.07 | 0.19 | 3.57E-17 | 0.12 | 0.02 | 0.15 | SNP |
| chr6_33033178_G_A | 33033178 | 0.12 | 0.07 | 0.19 | 3.58E-17 | 0.12 | 0.02 | 0.15 | SNP |
| chr6_33033188_C_T | 33033188 | 0.12 | 0.07 | 0.19 | 3.59E-17 | 0.12 | 0.02 | 0.15 | SNP |
| chr6_33037579_A_T | 33037579 | 0.12 | 0.07 | 0.18 | 3.61E-17 | 0.12 | 0.02 | 0.15 | SNP |
| chr6_33037580_T_G | 33037580 | 0.12 | 0.07 | 0.18 | 3.61E-17 | 0.12 | 0.02 | 0.15 | SNP |
| chr6_33036971_C_T | 33036971 | 0.12 | 0.07 | 0.19 | 3.62E-17 | 0.12 | 0.02 | 0.15 | SNP |
| chr6_33142162_C_A | 33142162 | 0.19 | 0.13 | 0.28 | 3.64E-17 | 0.13 | 0.04 | 0.16 | SNP |
| chr6_33037080_G_A | 33037080 | 0.12 | 0.07 | 0.19 | 3.66E-17 | 0.12 | 0.02 | 0.15 | SNP |
| chr6_33037082_A_G | 33037082 | 0.12 | 0.07 | 0.19 | 3.66E-17 | 0.12 | 0.02 | 0.15 | SNP |
| chr6_33033177_G_A | 33033177 | 0.12 | 0.07 | 0.19 | 3.68E-17 | 0.12 | 0.02 | 0.15 | SNP |
| chr6_33048602_C_A | 33048602 | 0.38 | 0.31 | 0.48 | 3.72E-17 | 0.37 | 0.21 | 0.40 | SNP |
| chr6_33036999_T_C | 33036999 | 0.12 | 0.07 | 0.19 | 3.79E-17 | 0.12 | 0.02 | 0.15 | SNP |
| chr6_33036708_C_T | 33036708 | 0.12 | 0.07 | 0.19 | 3.88E-17 | 0.12 | 0.02 | 0.15 | SNP |
| chr6_33036709_A_G | 33036709 | 0.12 | 0.07 | 0.19 | 3.88E-17 | 0.12 | 0.02 | 0.15 | SNP |
| chr6_33037104_C_T | 33037104 | 0.12 | 0.07 | 0.19 | 3.95E-17 | 0.12 | 0.02 | 0.15 | SNP |
| chr6_33036132_A_G | 33036132 | 0.12 | 0.07 | 0.19 | 4.11E-17 | 0.12 | 0.02 | 0.15 | SNP |
| chr6_33036133_G_A | 33036133 | 0.12 | 0.07 | 0.19 | 4.11E-17 | 0.12 | 0.02 | 0.15 | SNP |
| chr6_33032822_G_A | 33032822 | 0.12 | 0.07 | 0.19 | 4.14E-17 | 0.12 | 0.02 | 0.15 | SNP |
| chr6_33032865_A_C | 33032865 | 0.12 | 0.07 | 0.19 | 4.48E-17 | 0.12 | 0.02 | 0.15 | SNP |
| chr6_33040715_G_A | 33040715 | 0.10 | 0.06 | 0.17 | 4.70E-17 | 0.12 | 0.02 | 0.15 | SNP |
| chr6_33040736_T_C | 33040736 | 0.10 | 0.06 | 0.17 | 4.75E-17 | 0.12 | 0.02 | 0.15 | SNP |
| chr6_33040596_A_G | 33040596 | 0.12 | 0.07 | 0.19 | 4.91E-17 | 0.12 | 0.02 | 0.15 | SNP |
| chr6_33040744_G_C | 33040744 | 0.12 | 0.07 | 0.19 | 4.92E-17 | 0.12 | 0.02 | 0.15 | SNP |
| chr6_33040781_A_G | 33040781 | 0.12 | 0.07 | 0.19 | 4.95E-17 | 0.12 | 0.02 | 0.15 | SNP |
| chr6_33040798_T_C | 33040798 | 0.12 | 0.07 | 0.19 | 4.95E-17 | 0.12 | 0.02 | 0.15 | SNP |
| chr6_33040835_A_G | 33040835 | 0.12 | 0.07 | 0.19 | 4.95E-17 | 0.12 | 0.02 | 0.15 | SNP |
| chr6_33040877_T_C | 33040877 | 0.12 | 0.07 | 0.19 | 4.95E-17 | 0.12 | 0.02 | 0.15 | SNP |
| chr6_33041034_A_G | 33041034 | 0.12 | 0.07 | 0.19 | 4.95E-17 | 0.12 | 0.02 | 0.15 | SNP |
| chr6_33041226_C_T | 33041226 | 0.12 | 0.07 | 0.19 | 4.95E-17 | 0.12 | 0.02 | 0.15 | SNP |
| chr6_33039606_C_T | 33039606 | 0.12 | 0.07 | 0.19 | 4.96E-17 | 0.12 | 0.02 | 0.15 | SNP |
| chr6_33039980_A_G | 33039980 | 0.12 | 0.07 | 0.19 | 5.08E-17 | 0.12 | 0.02 | 0.15 | SNP |
| chr6_33043930_A_G | 33043930 | 0.12 | 0.07 | 0.19 | 5.39E-17 | 0.12 | 0.02 | 0.15 | SNP |
| chr6_33033266_T_A | 33033266 | 0.12 | 0.07 | 0.19 | 5.51E-17 | 0.12 | 0.02 | 0.15 | SNP |
| chr6_33036085_C_G | 33036085 | 0.12 | 0.07 | 0.20 | 5.56E-17 | 0.12 | 0.02 | 0.15 | SNP |
| chr6_33039405_C_G | 33039405 | 0.11 | 0.06 | 0.18 | 5.65E-17 | 0.12 | 0.02 | 0.15 | SNP |
| chr6_33036116_T_G | 33036116 | 0.12 | 0.07 | 0.20 | 5.84E-17 | 0.12 | 0.02 | 0.15 | SNP |
| chr6_33039837_G_A | 33039837 | 0.12 | 0.07 | 0.19 | 5.95E-17 | 0.12 | 0.02 | 0.15 | SNP |
| chr6_33039767_A_G | 33039767 | 0.12 | 0.07 | 0.19 | 6.03E-17 | 0.12 | 0.02 | 0.15 | SNP |
| chr6_33039484_A_G | 33039484 | 0.10 | 0.06 | 0.17 | 6.04E-17 | 0.12 | 0.02 | 0.15 | SNP |
| chr6_33037768_G_A | 33037768 | 0.12 | 0.07 | 0.19 | 6.07E-17 | 0.12 | 0.02 | 0.15 | SNP |
| chr6_33041176_C_T | 33041176 | 0.11 | 0.06 | 0.18 | 6.12E-17 | 0.12 | 0.02 | 0.15 | SNP |
| chr6_33033287_G_C | 33033287 | 0.12 | 0.07 | 0.20 | 6.29E-17 | 0.12 | 0.02 | 0.15 | SNP |
| chr6_33039686_T_C | 33039686 | 0.12 | 0.07 | 0.19 | 6.52E-17 | 0.12 | 0.02 | 0.15 | SNP |
| chr6_33033267_G_A | 33033267 | 0.12 | 0.07 | 0.20 | 7.04E-17 | 0.12 | 0.02 | 0.15 | SNP |
| chr6_33039719_A_G | 33039719 | 0.12 | 0.07 | 0.19 | 7.08E-17 | 0.12 | 0.02 | 0.15 | SNP |
| chr6_33039503_C_T | 33039503 | 0.10 | 0.06 | 0.17 | 7.10E-17 | 0.12 | 0.02 | 0.15 | SNP |
| chr6_33039295_T_A | 33039295 | 0.12 | 0.07 | 0.19 | 7.62E-17 | 0.12 | 0.02 | 0.15 | SNP |
| chr6_33039729_T_G | 33039729 | 0.12 | 0.07 | 0.19 | 8.21E-17 | 0.12 | 0.02 | 0.15 | SNP |
| chr6_33041186_G_A | 33041186 | 0.11 | 0.06 | 0.18 | 1.13E-16 | 0.12 | 0.02 | 0.15 | SNP |
| chr6_33141161_G_A | 33141161 | 2.08 | 1.75 | 2.48 | 1.38E-16 | 0.35 | 0.50 | 0.32 | SNP |
| chr6_33033351_G_T | 33033351 | 0.12 | 0.07 | 0.19 | 2.71E-16 | 0.13 | 0.02 | 0.15 | SNP |
| chr6_33044257_T_C | 33044257 | 0.12 | 0.07 | 0.20 | 3.74E-16 | 0.12 | 0.02 | 0.14 | SNP |
| chr6_33158189_C_A | 33158189 | 2.06 | 1.73 | 2.46 | 4.31E-16 | 0.36 | 0.50 | 0.32 | SNP |
| chr6_33033357_C_G | 33033357 | 0.12 | 0.07 | 0.19 | 4.84E-16 | 0.12 | 0.02 | 0.15 | SNP |
| chr6_33145710_G_A | 33145710 | 2.03 | 1.71 | 2.41 | 5.18E-16 | 0.35 | 0.49 | 0.32 | SNP |
| chr6_33141280_G_A | 33141280 | 0.20 | 0.13 | 0.29 | 5.94E-16 | 0.14 | 0.04 | 0.16 | SNP |
| chr6_33154656_G_A | 33154656 | 0.19 | 0.12 | 0.28 | 6.76E-16 | 0.12 | 0.03 | 0.15 | SNP |
| chr6_33037866_A_G | 33037866 | 0.12 | 0.07 | 0.20 | 7.60E-16 | 0.12 | 0.02 | 0.14 | SNP |
| chr6_33146053_C_G | 33146053 | 0.19 | 0.12 | 0.28 | 7.76E-16 | 0.12 | 0.03 | 0.15 | SNP |
| chr6_33148878_C_T | 33148878 | 0.19 | 0.13 | 0.28 | 8.10E-16 | 0.12 | 0.03 | 0.15 | SNP |
| chr6_33037865_C_T | 33037865 | 0.13 | 0.07 | 0.20 | 9.04E-16 | 0.12 | 0.02 | 0.14 | SNP |
| chr6_33140983_G_A | 33140983 | 2.05 | 1.72 | 2.44 | 9.86E-16 | 0.36 | 0.49 | 0.32 | SNP |
| chr6_33137403_T_C | 33137403 | 2.02 | 1.70 | 2.40 | 1.12E-15 | 0.36 | 0.50 | 0.32 | SNP |
| chr6_33142253_G_C | 33142253 | 2.01 | 1.69 | 2.39 | 2.53E-15 | 0.36 | 0.50 | 0.33 | SNP |
| chr6_33048628_A_C | 33048628 | 0.15 | 0.09 | 0.23 | 2.78E-15 | 0.13 | 0.03 | 0.15 | SNP |
| chr6_33095762_G_A | 33095762 | 0.12 | 0.07 | 0.20 | 3.68E-15 | 0.10 | 0.02 | 0.12 | SNP |
| chr6_33048606_G_C | 33048606 | 0.16 | 0.10 | 0.24 | 4.35E-15 | 0.12 | 0.03 | 0.14 | SNP |
| chr6_33135392_G_A | 33135392 | 2.20 | 1.81 | 2.69 | 5.28E-15 | 0.38 | 0.52 | 0.34 | SNP |
| chr6_33139475_G_T | 33139475 | 2.03 | 1.70 | 2.42 | 5.58E-15 | 0.36 | 0.49 | 0.32 | SNP |
| chr6_33036003_G_C | 33036003 | 0.12 | 0.07 | 0.20 | 5.72E-15 | 0.11 | 0.02 | 0.13 | SNP |
| chr6_33162215_C_A | 33162215 | 1.97 | 1.67 | 2.34 | 5.82E-15 | 0.36 | 0.49 | 0.33 | SNP |
| chr6_33037878_G_A | 33037878 | 0.13 | 0.07 | 0.21 | 6.89E-15 | 0.12 | 0.02 | 0.14 | SNP |
| chr6_33035986_T_C | 33035986 | 0.12 | 0.07 | 0.20 | 7.11E-15 | 0.11 | 0.02 | 0.13 | SNP |
| chr6_33143136_G_A | 33143136 | 0.19 | 0.12 | 0.28 | 7.79E-15 | 0.14 | 0.04 | 0.16 | SNP |
| chr6_33161661_G_A | 33161661 | 1.97 | 1.66 | 2.34 | 8.03E-15 | 0.36 | 0.49 | 0.33 | SNP |
| chr6_33149152_C_G | 33149152 | 0.20 | 0.13 | 0.29 | 8.16E-15 | 0.12 | 0.03 | 0.15 | SNP |
| chr6_33144373_A_G | 33144373 | 1.96 | 1.65 | 2.33 | 8.45E-15 | 0.36 | 0.49 | 0.33 | SNP |
| chr6_33035980_T_A | 33035980 | 0.12 | 0.07 | 0.20 | 9.31E-15 | 0.11 | 0.02 | 0.13 | SNP |
| chr6_33041064_C_A | 33041064 | 0.14 | 0.08 | 0.22 | 1.02E-14 | 0.10 | 0.02 | 0.12 | SNP |
| chr6_33141920_C_G | 33141920 | 1.96 | 1.65 | 2.33 | 1.06E-14 | 0.36 | 0.49 | 0.33 | SNP |
| chr6_33139662_G_A | 33139662 | 1.96 | 1.65 | 2.33 | 1.08E-14 | 0.36 | 0.49 | 0.33 | SNP |
| chr6_33048380_G_A | 33048380 | 0.14 | 0.08 | 0.23 | 1.15E-14 | 0.10 | 0.02 | 0.12 | SNP |
| chr6_33046752_G_C | 33046752 | 0.14 | 0.08 | 0.23 | 1.18E-14 | 0.10 | 0.02 | 0.12 | SNP |
| chr6_33048102_C_T | 33048102 | 0.14 | 0.08 | 0.23 | 1.23E-14 | 0.10 | 0.02 | 0.12 | SNP |
| chr6_33095555_T_A | 33095555 | 0.13 | 0.07 | 0.21 | 1.27E-14 | 0.10 | 0.02 | 0.12 | SNP |
| chr6_33035982_G_A | 33035982 | 0.12 | 0.07 | 0.20 | 1.30E-14 | 0.11 | 0.02 | 0.13 | SNP |
| chr6_33037560_G_A | 33037560 | 0.10 | 0.05 | 0.17 | 1.30E-14 | 0.10 | 0.01 | 0.12 | SNP |
| chr6_33033388_A_C | 33033388 | 0.11 | 0.06 | 0.19 | 1.34E-14 | 0.13 | 0.02 | 0.15 | SNP |
| chr6_33163451_A_G | 33163451 | 1.99 | 1.67 | 2.38 | 1.90E-14 | 0.36 | 0.49 | 0.33 | SNP |
| chr6_33033390_A_T | 33033390 | 0.10 | 0.05 | 0.17 | 1.95E-14 | 0.13 | 0.02 | 0.15 | SNP |
| chr6_33033313_G_T | 33033313 | 0.11 | 0.06 | 0.18 | 2.03E-14 | 0.10 | 0.02 | 0.12 | SNP |
| chr6_33045823_A_G | 33045823 | 0.08 | 0.04 | 0.15 | 2.52E-14 | 0.10 | 0.01 | 0.13 | SNP |
| chr6_33035974_A_G | 33035974 | 0.13 | 0.07 | 0.21 | 2.76E-14 | 0.11 | 0.02 | 0.13 | SNP |
| chr6_33138498_C_T | 33138498 | 1.97 | 1.65 | 2.34 | 2.95E-14 | 0.37 | 0.50 | 0.34 | SNP |
| chr6_33138839_A_T | 33138839 | 0.19 | 0.12 | 0.29 | 4.00E-14 | 0.13 | 0.03 | 0.15 | SNP |
| chr6_33037891_C_T | 33037891 | 0.12 | 0.06 | 0.20 | 4.35E-14 | 0.11 | 0.02 | 0.14 | SNP |
| chr6_33037557_C_T | 33037557 | 0.10 | 0.05 | 0.18 | 7.72E-14 | 0.09 | 0.01 | 0.11 | SNP |
| chr6_33048921_A_G | 33048921 | 0.12 | 0.07 | 0.20 | 8.43E-14 | 0.10 | 0.02 | 0.12 | SNP |
| chr6_33035969_A_G | 33035969 | 0.13 | 0.07 | 0.21 | 9.59E-14 | 0.10 | 0.02 | 0.13 | SNP |
| chr6_33041073_A_C | 33041073 | 0.13 | 0.07 | 0.22 | 1.15E-13 | 0.10 | 0.02 | 0.12 | SNP |
| chr6_33044258_G_A | 33044258 | 0.10 | 0.05 | 0.18 | 1.38E-13 | 0.09 | 0.01 | 0.11 | SNP |
| chr6_33044388_T_C | 33044388 | 0.11 | 0.06 | 0.18 | 1.54E-13 | 0.09 | 0.01 | 0.11 | SNP |
| chr6_33042368_A_G | 33042368 | 0.11 | 0.06 | 0.18 | 1.54E-13 | 0.09 | 0.01 | 0.11 | SNP |
| chr6_33039694_G_T | 33039694 | 0.11 | 0.06 | 0.18 | 1.59E-13 | 0.09 | 0.01 | 0.11 | SNP |
| chr6_33042406_C_G | 33042406 | 0.11 | 0.06 | 0.18 | 1.59E-13 | 0.09 | 0.01 | 0.11 | SNP |
| chr6_33040654_A_G | 33040654 | 0.11 | 0.06 | 0.18 | 1.60E-13 | 0.09 | 0.01 | 0.11 | SNP |
| chr6_33042492_T_C | 33042492 | 0.11 | 0.06 | 0.18 | 1.60E-13 | 0.09 | 0.01 | 0.11 | SNP |
| chr6_33042880_A_G | 33042880 | 0.11 | 0.06 | 0.18 | 1.60E-13 | 0.09 | 0.01 | 0.11 | SNP |
| chr6_33044188_T_A | 33044188 | 0.11 | 0.06 | 0.18 | 1.62E-13 | 0.09 | 0.01 | 0.11 | SNP |
| chr6_33039681_A_G | 33039681 | 0.11 | 0.06 | 0.18 | 1.64E-13 | 0.09 | 0.01 | 0.11 | SNP |
| chr6_33039697_T_G | 33039697 | 0.11 | 0.06 | 0.18 | 1.64E-13 | 0.09 | 0.01 | 0.11 | SNP |
| chr6_33040044_A_G | 33040044 | 0.11 | 0.06 | 0.18 | 1.65E-13 | 0.09 | 0.01 | 0.11 | SNP |
| chr6_33138955_A_G | 33138955 | 1.87 | 1.58 | 2.22 | 3.94E-13 | 0.37 | 0.50 | 0.34 | SNP |
| chr6_33143948_C_G | 33143948 | 0.50 | 0.41 | 0.60 | 5.73E-13 | 0.34 | 0.22 | 0.37 | SNP |
| chr6_33144243_T_A | 33144243 | 0.50 | 0.42 | 0.61 | 8.14E-13 | 0.34 | 0.22 | 0.37 | SNP |
| chr6_33040478_C_A | 33040478 | 0.11 | 0.06 | 0.20 | 1.02E-12 | 0.09 | 0.01 | 0.11 | SNP |
| chr6_33048937_C_G | 33048937 | 0.10 | 0.05 | 0.19 | 6.10E-12 | 0.08 | 0.01 | 0.10 | SNP |
| chr6_33136638_A_G | 33136638 | 1.81 | 1.53 | 2.15 | 1.10E-11 | 0.37 | 0.47 | 0.34 | SNP |

| | | | | | | | | | |
|---|---|---|---|---|---|---|---|---|---|
| chr6_33036290_G_A | 33036290 | 1.93 | 1.59 | 2.34 | 2.83E-11 | 0.18 | 0.27 | 0.16 | SNP |
| chr6_33048348_G_A | 33048348 | 0.14 | 0.08 | 0.25 | 5.04E-11 | 0.08 | 0.02 | 0.09 | SNP |
| chr6_33045147_A_T | 33045147 | 0.49 | 0.39 | 0.61 | 9.52E-11 | 0.30 | 0.18 | 0.33 | SNP |
| chr6_33045500_T_C | 33045500 | 0.13 | 0.07 | 0.23 | 1.28E-10 | 0.08 | 0.01 | 0.09 | SNP |
| chr6_33048539_T_A | 33048539 | 0.11 | 0.05 | 0.20 | 2.40E-10 | 0.07 | 0.01 | 0.09 | SNP |
| chr6_33139328_C_T | 33139328 | 0.56 | 0.47 | 0.67 | 3.20E-10 | 0.43 | 0.33 | 0.46 | SNP |
| chr6_33049726_G_T | 33049726 | 0.11 | 0.05 | 0.21 | 5.50E-10 | 0.07 | 0.01 | 0.08 | SNP |
| chr6_33049727_A_T | 33049727 | 0.11 | 0.05 | 0.21 | 5.50E-10 | 0.07 | 0.01 | 0.08 | SNP |
| chr6_33047898_C_A | 33047898 | 0.16 | 0.09 | 0.28 | 1.06E-09 | 0.07 | 0.02 | 0.08 | SNP |
| chr6_33134392_A_C | 33134392 | 1.67 | 1.41 | 1.98 | 3.82E-09 | 0.40 | 0.50 | 0.38 | SNP |
| chr6_33132931_T_C | 33132931 | 1.80 | 1.48 | 2.21 | 6.11E-09 | 0.42 | 0.52 | 0.39 | SNP |
| chr6_33043520_C_G | 33043520 | 0.37 | 0.26 | 0.51 | 7.75E-09 | 0.12 | 0.05 | 0.13 | SNP |
| chr6_33037199_G_C | 33037199 | 0.37 | 0.26 | 0.51 | 9.31E-09 | 0.12 | 0.05 | 0.13 | SNP |
| chr6_33032851_T_C | 33032851 | 0.37 | 0.26 | 0.51 | 9.43E-09 | 0.12 | 0.05 | 0.13 | SNP |
| chr6_33134102_C_T | 33134102 | 1.65 | 1.39 | 1.96 | 1.01E-08 | 0.41 | 0.51 | 0.38 | SNP |
| chr6_33033274_T_C | 33033274 | 0.37 | 0.26 | 0.52 | 1.39E-08 | 0.12 | 0.05 | 0.13 | SNP |
| chr6_33136575_T_C | 33136575 | 1.63 | 1.38 | 1.93 | 1.45E-08 | 0.44 | 0.53 | 0.41 | SNP |
| chr6_33041734_T_C | 33041734 | 0.37 | 0.25 | 0.51 | 1.73E-08 | 0.11 | 0.05 | 0.13 | SNP |
| chr6_33131734_G_A | 33131734 | 1.77 | 1.45 | 2.17 | 2.52E-08 | 0.41 | 0.52 | 0.39 | SNP |
| chr6_33096003_G_C | 33096003 | 0.58 | 0.48 | 0.70 | 2.66E-08 | 0.43 | 0.34 | 0.45 | SNP |
| xHLA_2field_DPB1_03_01 | 33043767 | 0.23 | 0.13 | 0.37 | 4.11E-08 | 0.06 | 0.02 | 0.08 | HLA (xHLA) |
| chr6_33049902_T_A | 33049902 | 0.17 | 0.09 | 0.31 | 5.15E-08 | 0.06 | 0.01 | 0.07 | SNP |
| chr6_33050178_C_T | 33050178 | 0.17 | 0.09 | 0.31 | 5.15E-08 | 0.06 | 0.01 | 0.07 | SNP |
| chr6_33048354_A_T | 33048354 | 0.15 | 0.07 | 0.28 | 6.92E-08 | 0.05 | 0.01 | 0.06 | SNP |
| chr6_33047173_T_C | 33047173 | 0.15 | 0.07 | 0.28 | 7.25E-08 | 0.05 | 0.01 | 0.06 | SNP |
| chr6_32978587_C_A | 32978587 | 0.24 | 0.14 | 0.40 | 1.13E-07 | 0.06 | 0.02 | 0.07 | SNP |
| chr6_33096009_A_C | 33096009 | 0.59 | 0.49 | 0.72 | 1.31E-07 | 0.42 | 0.34 | 0.44 | SNP |
| chr6_33137727_T_C | 33137727 | 1.53 | 1.30 | 1.81 | 6.42E-07 | 0.42 | 0.51 | 0.40 | SNP |
| chr6_33132365_T_C | 33132365 | 0.14 | 0.06 | 0.28 | 7.67E-07 | 0.05 | 0.01 | 0.06 | SNP |
| chr6_32555317_C_T | 32555317 | 0.40 | 0.27 | 0.57 | 9.54E-07 | 0.09 | 0.05 | 0.10 | SNP |
| chr6_33160906_G_T | 33160906 | 0.16 | 0.07 | 0.31 | 1.18E-06 | 0.05 | 0.01 | 0.06 | SNP |
| chr6_32376019_G_T | 32376019 | 0.44 | 0.31 | 0.61 | 1.55E-06 | 0.10 | 0.05 | 0.11 | SNP |
| chr6_33079626_T_C | 33079626 | 0.17 | 0.07 | 0.33 | 2.32E-06 | 0.05 | 0.01 | 0.06 | SNP |
| chr6_33239869_C_T | 33239869 | 1.90 | 1.45 | 2.49 | 3.12E-06 | 0.09 | 0.13 | 0.08 | SNP |
| chr6_33039804_C_T | 33039804 | 1.55 | 1.29 | 1.86 | 3.40E-06 | 0.21 | 0.28 | 0.20 | SNP |
| chr6_33045272_C_A | 33045272 | 0.59 | 0.47 | 0.74 | 3.47E-06 | 0.23 | 0.15 | 0.25 | SNP |
| xHLA_2field_DPB1_02_01 | 33043767 | 0.46 | 0.33 | 0.63 | 4.03E-06 | 0.11 | 0.06 | 0.12 | HLA (xHLA) |
| chr6_33132338_A_G | 33132338 | 1.57 | 1.30 | 1.91 | 4.29E-06 | 0.41 | 0.50 | 0.39 | SNP |
| chr6_32410691_G_A | 32410691 | 0.50 | 0.37 | 0.67 | 5.84E-06 | 0.22 | 0.17 | 0.23 | SNP |
| chr6_33044795_C_T | 33044795 | 2.31 | 1.60 | 3.32 | 7.12E-06 | 0.04 | 0.08 | 0.03 | SNP |
| chr6_32412809_T_A | 32412809 | 0.51 | 0.38 | 0.68 | 7.56E-06 | 0.22 | 0.17 | 0.23 | SNP |
| chr6_32409060_G_A | 32409060 | 0.50 | 0.37 | 0.67 | 7.62E-06 | 0.22 | 0.17 | 0.23 | SNP |
| chr6_32412592_G_A | 32412592 | 0.51 | 0.38 | 0.68 | 7.67E-06 | 0.22 | 0.17 | 0.23 | SNP |
| chr6_32632777_C_T | 32632777 | 0.47 | 0.34 | 0.65 | 7.71E-06 | 0.14 | 0.08 | 0.15 | SNP |
| chr6_33384473_C_T | 33384473 | 2.01 | 1.48 | 2.73 | 7.99E-06 | 0.06 | 0.11 | 0.05 | SNP |
| chr6_33095584_G_C | 33095584 | 1.74 | 1.36 | 2.21 | 8.09E-06 | 0.11 | 0.16 | 0.10 | SNP |
| chr6_32187202_T_C | 32187202 | 1.61 | 1.31 | 1.99 | 8.20E-06 | 0.43 | 0.49 | 0.42 | SNP |
| chr6_32406611_C_A | 32406611 | 0.50 | 0.37 | 0.68 | 8.35E-06 | 0.22 | 0.17 | 0.23 | SNP |
| chr6_33623509_T_C | 33623509 | 0.22 | 0.10 | 0.41 | 8.46E-06 | 0.04 | 0.01 | 0.05 | SNP |
| chr6_32411307_C_T | 32411307 | 0.51 | 0.38 | 0.68 | 8.62E-06 | 0.22 | 0.17 | 0.23 | SNP |
| chr6_32412566_T_A | 32412566 | 0.51 | 0.38 | 0.68 | 8.62E-06 | 0.22 | 0.17 | 0.23 | SNP |
| chr6_32408842_A_G | 32408842 | 0.51 | 0.37 | 0.68 | 9.59E-06 | 0.22 | 0.17 | 0.23 | SNP |
| chr6_33048119_C_T | 33048119 | 2.21 | 1.55 | 3.14 | 1.01E-05 | 0.04 | 0.08 | 0.03 | SNP |
| xHLA_2field_C_07_01 | 31236526 | 2.02 | 1.48 | 2.76 | 1.01E-05 | 0.15 | 0.17 | 0.14 | HLA (xHLA) |
| chr6_32411376_T_G | 32411376 | 0.51 | 0.38 | 0.68 | 1.03E-05 | 0.22 | 0.17 | 0.23 | SNP |
| chr6_32410215_T_C | 32410215 | 0.51 | 0.38 | 0.69 | 1.10E-05 | 0.22 | 0.17 | 0.23 | SNP |
| chr6_32410210_T_C | 32410210 | 0.51 | 0.38 | 0.69 | 1.24E-05 | 0.22 | 0.17 | 0.23 | SNP |
| chr6_32973263_T_A | 32973263 | 1.43 | 1.22 | 1.69 | 1.71E-05 | 0.46 | 0.52 | 0.44 | SNP |
| chr6_32609216_T_G | 32609216 | 0.61 | 0.48 | 0.76 | 1.85E-05 | 0.50 | 0.42 | 0.52 | SNP |
| chr6_32190390_T_G | 32190390 | 1.61 | 1.29 | 2.01 | 1.97E-05 | 0.34 | 0.43 | 0.32 | SNP |
| chr6_33095865_C_T | 33095865 | 1.68 | 1.32 | 2.13 | 2.38E-05 | 0.11 | 0.15 | 0.10 | SNP |
| chr6_31235505_G_A | 31235505 | 1.76 | 1.35 | 2.28 | 2.44E-05 | 0.21 | 0.24 | 0.21 | SNP |
| chr6_32609207_T_C | 32609207 | 0.62 | 0.49 | 0.77 | 2.69E-05 | 0.50 | 0.42 | 0.52 | SNP |
| chr6_31325090_T_C | 31325090 | 1.69 | 1.32 | 2.17 | 2.75E-05 | 0.37 | 0.41 | 0.36 | SNP |
| chr6_31235869_T_C | 31235869 | 1.73 | 1.34 | 2.25 | 3.34E-05 | 0.22 | 0.24 | 0.21 | SNP |
| chr6_32628953_G_C | 32628953 | 0.62 | 0.49 | 0.78 | 3.76E-05 | 0.48 | 0.41 | 0.50 | SNP |
| chr6_32501522_A_G | 32501522 | 1.70 | 1.32 | 2.20 | 4.35E-05 | 0.24 | 0.29 | 0.23 | SNP |
| chr6_32610769_C_A | 32610769 | 0.61 | 0.48 | 0.77 | 4.39E-05 | 0.33 | 0.28 | 0.34 | SNP |
| chr6_33647936_C_T | 33647936 | 0.21 | 0.09 | 0.42 | 4.92E-05 | 0.04 | 0.01 | 0.05 | SNP |
| chr6_32408917_C_T | 32408917 | 1.56 | 1.26 | 1.94 | 5.46E-05 | 0.26 | 0.30 | 0.25 | SNP |
| chr6_32631029_T_C | 32631029 | 0.36 | 0.21 | 0.58 | 5.53E-05 | 0.06 | 0.03 | 0.07 | SNP |
| chr6_32611968_T_G | 32611968 | 0.62 | 0.49 | 0.78 | 6.03E-05 | 0.47 | 0.40 | 0.49 | SNP |
| xHLA_2field_DRB1_13_02 | 32546552 | 0.36 | 0.21 | 0.57 | 6.33E-05 | 0.05 | 0.02 | 0.06 | HLA (xHLA) |
| chr6_33049881_T_G | 33049881 | 0.54 | 0.40 | 0.73 | 6.79E-05 | 0.11 | 0.07 | 0.12 | SNP |
| chr6_32973303_T_C | 32973303 | 0.50 | 0.35 | 0.70 | 7.27E-05 | 0.10 | 0.06 | 0.11 | SNP |
| chr6_32556491_A_G | 32556491 | 0.47 | 0.33 | 0.68 | 7.64E-05 | 0.09 | 0.07 | 0.10 | SNP |
| chr6_32559709_C_T | 32559709 | 0.66 | 0.53 | 0.81 | 7.67E-05 | 0.44 | 0.38 | 0.46 | SNP |
| chr6_32610704_C_T | 32610704 | 0.62 | 0.48 | 0.78 | 7.98E-05 | 0.32 | 0.28 | 0.34 | SNP |
| chr6_32628967_T_C | 32628967 | 0.64 | 0.51 | 0.80 | 9.89E-05 | 0.49 | 0.41 | 0.51 | SNP |
| chr6_32606701_A_G | 32606701 | 0.62 | 0.48 | 0.79 | 0.000116 | 0.17 | 0.12 | 0.18 | SNP |
| chr6_32439696_T_G | 32439696 | 0.39 | 0.23 | 0.61 | 0.000122 | 0.05 | 0.03 | 0.06 | SNP |
| chr6_31112737_C_A | 31112737 | 1.82 | 1.34 | 2.47 | 0.000126 | 0.20 | 0.23 | 0.19 | SNP |
| chr6_32976909_C_A | 32976909 | 0.72 | 0.61 | 0.85 | 0.000126 | 0.48 | 0.43 | 0.49 | SNP |
| chr6_32629297_C_T | 32629297 | 0.65 | 0.52 | 0.81 | 0.000135 | 0.44 | 0.37 | 0.46 | SNP |
| chr6_32408735_A_C | 32408735 | 0.65 | 0.52 | 0.81 | 0.000135 | 0.33 | 0.26 | 0.35 | SNP |
| chr6_32192436_A_G | 32192436 | 0.44 | 0.28 | 0.66 | 0.000137 | 0.06 | 0.03 | 0.07 | SNP |
| chr6_32611723_T_G | 32611723 | 0.64 | 0.51 | 0.80 | 0.000137 | 0.47 | 0.40 | 0.49 | SNP |
| chr6_32608880_G_A | 32608880 | 1.56 | 1.24 | 1.95 | 0.000141 | 0.33 | 0.40 | 0.31 | SNP |
| chr6_32603277_G_T | 32603277 | 0.40 | 0.24 | 0.63 | 0.000145 | 0.05 | 0.03 | 0.06 | SNP |
| chr6_32609009_T_G | 32609009 | 0.40 | 0.24 | 0.63 | 0.000147 | 0.05 | 0.03 | 0.06 | SNP |
| chr6_32606376_T_C | 32606376 | 0.39 | 0.24 | 0.62 | 0.000152 | 0.05 | 0.02 | 0.06 | SNP |
| chr6_31243828_A_G | 31243828 | 0.67 | 0.54 | 0.82 | 0.000155 | 0.32 | 0.28 | 0.33 | SNP |
| chr6_32606682_C_G | 32606682 | 0.40 | 0.24 | 0.63 | 0.000158 | 0.05 | 0.03 | 0.06 | SNP |
| chr6_32609047_C_T | 32609047 | 0.40 | 0.24 | 0.63 | 0.000159 | 0.05 | 0.03 | 0.06 | SNP |
| chr6_32603896_T_C | 32603896 | 0.40 | 0.24 | 0.63 | 0.000160 | 0.05 | 0.03 | 0.06 | SNP |
| chr6_32604166_A_G | 32604166 | 0.40 | 0.24 | 0.63 | 0.000161 | 0.05 | 0.03 | 0.06 | SNP |
| chr6_32606691_T_G | 32606691 | 0.40 | 0.24 | 0.63 | 0.000162 | 0.05 | 0.03 | 0.06 | SNP |
| chr6_32441129_G_A | 32441129 | 0.40 | 0.24 | 0.63 | 0.000166 | 0.05 | 0.03 | 0.06 | SNP |
| chr6_32188383_T_C | 32188383 | 1.46 | 1.20 | 1.78 | 0.000167 | 0.42 | 0.48 | 0.41 | SNP |
| chr6_32608435_T_C | 32608435 | 0.40 | 0.24 | 0.63 | 0.000171 | 0.05 | 0.03 | 0.06 | SNP |
| chr6_32441046_G_A | 32441046 | 0.40 | 0.24 | 0.63 | 0.000172 | 0.05 | 0.03 | 0.06 | SNP |
| chr6_32628606_C_A | 32628606 | 0.40 | 0.24 | 0.63 | 0.000180 | 0.05 | 0.03 | 0.06 | SNP |
| chr6_32553418_T_G | 32553418 | 0.66 | 0.53 | 0.82 | 0.000181 | 0.41 | 0.35 | 0.42 | SNP |
| chr6_32610947_A_C | 32610947 | 0.42 | 0.26 | 0.64 | 0.000185 | 0.06 | 0.03 | 0.06 | SNP |
| chr6_32501459_T_C | 32501459 | 0.68 | 0.55 | 0.83 | 0.000192 | 0.39 | 0.33 | 0.41 | SNP |
| chr6_31248382_C_T | 31248382 | 0.51 | 0.36 | 0.72 | 0.000193 | 0.10 | 0.06 | 0.11 | SNP |
| chr6_32407302_A_G | 32407302 | 0.65 | 0.52 | 0.81 | 0.000202 | 0.33 | 0.26 | 0.34 | SNP |
| chr6_32442703_G_A | 32442703 | 0.39 | 0.23 | 0.63 | 0.000207 | 0.05 | 0.02 | 0.06 | SNP |
| chr6_32972903_G_A | 32972903 | 1.49 | 1.21 | 1.84 | 0.000209 | 0.14 | 0.19 | 0.13 | SNP |
| chr6_32636040_C_T | 32636040 | 0.63 | 0.49 | 0.80 | 0.000217 | 0.31 | 0.26 | 0.32 | SNP |
| chr6_32917042_A_C | 32917042 | 1.81 | 1.32 | 2.48 | 0.000235 | 0.06 | 0.09 | 0.05 | SNP |
| chr6_31325494_C_T | 31325494 | 1.65 | 1.26 | 2.15 | 0.000238 | 0.33 | 0.36 | 0.32 | SNP |
| chr6_32804687_T_C | 32804687 | 1.77 | 1.30 | 2.39 | 0.000240 | 0.07 | 0.10 | 0.06 | SNP |
| chr6_32406887_C_T | 32406887 | 0.64 | 0.50 | 0.81 | 0.000240 | 0.31 | 0.23 | 0.33 | SNP |
| chr6_32407310_T_G | 32407310 | 0.65 | 0.52 | 0.82 | 0.000244 | 0.33 | 0.26 | 0.34 | SNP |
| chr6_32609425_C_G | 32609425 | 0.43 | 0.26 | 0.66 | 0.000246 | 0.05 | 0.03 | 0.06 | SNP |
| chr6_32628843_C_T | 32628843 | 0.41 | 0.25 | 0.65 | 0.000247 | 0.05 | 0.03 | 0.06 | SNP |
| chr6_32607193_T_C | 32607193 | 1.53 | 1.22 | 1.92 | 0.000249 | 0.34 | 0.42 | 0.32 | SNP |
| chr6_32407404_G_C | 32407404 | 0.66 | 0.52 | 0.82 | 0.000249 | 0.33 | 0.26 | 0.35 | SNP |
| chr6_32407468_T_C | 32407468 | 0.66 | 0.52 | 0.82 | 0.000249 | 0.33 | 0.26 | 0.35 | SNP |
| chr6_32629545_T_C | 32629545 | 0.41 | 0.25 | 0.65 | 0.000249 | 0.05 | 0.03 | 0.06 | SNP |
| chr6_32612003_T_C | 32612003 | 0.65 | 0.52 | 0.82 | 0.000260 | 0.47 | 0.41 | 0.49 | SNP |
| chr6_32559714_G_A | 32559714 | 0.68 | 0.55 | 0.83 | 0.000264 | 0.45 | 0.39 | 0.47 | SNP |
| chr6_32559708_G_A | 32559708 | 1.56 | 1.23 | 1.98 | 0.000268 | 0.27 | 0.34 | 0.25 | SNP |
| chr6_32603029_T_C | 32603029 | 0.67 | 0.54 | 0.83 | 0.000277 | 0.27 | 0.20 | 0.29 | SNP |
| chr6_32184066_G_T | 32184066 | 1.43 | 1.18 | 1.73 | 0.000277 | 0.42 | 0.47 | 0.41 | SNP |
| chr6_32634485_C_A | 32634485 | 0.64 | 0.50 | 0.81 | 0.000278 | 0.32 | 0.27 | 0.33 | SNP |
| chr6_32633273_T_G | 32633273 | 0.67 | 0.53 | 0.83 | 0.000279 | 0.35 | 0.30 | 0.37 | SNP |
| chr6_33556605_G_C | 33556605 | 0.66 | 0.52 | 0.82 | 0.000280 | 0.21 | 0.15 | 0.22 | SNP |
| chr6_31325513_G_C | 31325513 | 1.63 | 1.25 | 2.13 | 0.000284 | 0.33 | 0.36 | 0.32 | SNP |
| chr6_32636591_G_A | 32636591 | 0.62 | 0.48 | 0.80 | 0.000299 | 0.45 | 0.38 | 0.47 | SNP |
| chr6_33169895_C_G | 33169895 | 1.57 | 1.22 | 1.99 | 0.000301 | 0.11 | 0.14 | 0.10 | SNP |
| chr6_31125978_A_T | 31125978 | 1.76 | 1.29 | 2.39 | 0.000303 | 0.20 | 0.23 | 0.19 | SNP |
| chr6_31325504_C_T | 31325504 | 1.63 | 1.25 | 2.13 | 0.000304 | 0.33 | 0.36 | 0.32 | SNP |
| chr6_32407153_A_T | 32407153 | 0.66 | 0.53 | 0.83 | 0.000305 | 0.33 | 0.26 | 0.34 | SNP |
| chr6_32804570_G_A | 32804570 | 1.42 | 1.17 | 1.72 | 0.000315 | 0.37 | 0.44 | 0.35 | SNP |
| chr6_32785275_G_A | 32785275 | 0.55 | 0.39 | 0.76 | 0.000317 | 0.09 | 0.06 | 0.10 | SNP |
| chr6_31325341_C_G | 31325341 | 1.63 | 1.25 | 2.13 | 0.000324 | 0.33 | 0.36 | 0.32 | SNP |
| chr6_31236051_A_G | 31236051 | 1.41 | 1.17 | 1.71 | 0.000332 | 0.49 | 0.52 | 0.48 | SNP |
| chr6_32781121_G_C | 32781121 | 0.55 | 0.39 | 0.76 | 0.000341 | 0.09 | 0.06 | 0.10 | SNP |
| chr6_32336299_A_G | 32336299 | 0.38 | 0.22 | 0.63 | 0.000353 | 0.04 | 0.02 | 0.05 | SNP |
| chr6_32406412_C_G | 32406412 | 0.66 | 0.53 | 0.83 | 0.000353 | 0.33 | 0.26 | 0.34 | SNP |
| chr6_32189032_T_C | 32189032 | 1.43 | 1.18 | 1.74 | 0.000356 | 0.29 | 0.35 | 0.28 | SNP |
| chr6_32603355_T_C | 32603355 | 0.68 | 0.55 | 0.84 | 0.000358 | 0.27 | 0.20 | 0.29 | SNP |
| chr6_33137689_C_T | 33137689 | 1.55 | 1.22 | 1.97 | 0.000361 | 0.11 | 0.14 | 0.10 | SNP |
| chr6_32629320_G_A | 32629320 | 0.64 | 0.50 | 0.82 | 0.000362 | 0.32 | 0.28 | 0.34 | SNP |
| chr6_32813214_G_A | 32813214 | 1.77 | 1.29 | 2.43 | 0.000368 | 0.06 | 0.09 | 0.06 | SNP |
| chr6_32612050_T_A | 32612050 | 0.66 | 0.53 | 0.83 | 0.000372 | 0.47 | 0.40 | 0.49 | SNP |
| chr6_32627561_C_T | 32627561 | 0.64 | 0.50 | 0.82 | 0.000377 | 0.32 | 0.27 | 0.33 | SNP |
| chr6_33130430_G_A | 33130430 | 0.66 | 0.52 | 0.83 | 0.000377 | 0.18 | 0.13 | 0.20 | SNP |
| chr6_32409484_C_T | 32409484 | 0.66 | 0.53 | 0.83 | 0.000377 | 0.33 | 0.26 | 0.34 | SNP |
| chr6_31325532_A_G | 31325532 | 1.61 | 1.24 | 2.10 | 0.000382 | 0.33 | 0.36 | 0.32 | SNP |
| chr6_31325380_T_C | 31325380 | 1.62 | 1.24 | 2.11 | 0.000388 | 0.33 | 0.36 | 0.32 | SNP |
| chr6_32785284_T_C | 32785284 | 0.56 | 0.40 | 0.76 | 0.000390 | 0.09 | 0.06 | 0.10 | SNP |
| chr6_32151934_A_G | 32151934 | 1.79 | 1.29 | 2.46 | 0.000395 | 0.16 | 0.19 | 0.16 | SNP |

| | | | | | | | | | |
|---|---|---|---|---|---|---|---|---|---|
| chr6_32500957_A_T | 32500957 | 0.54 | 0.39 | 0.76 | 0.000395 | 0.19 | 0.15 | 0.20 | SNP |
| chr6_29855331_G_A | 29855331 | 1.49 | 1.19 | 1.86 | 0.000397 | 0.19 | 0.22 | 0.19 | SNP |
| chr6_32407440_T_C | 32407440 | 0.67 | 0.53 | 0.83 | 0.000407 | 0.32 | 0.26 | 0.34 | SNP |
| chr6_32631039_G_A | 32631039 | 0.63 | 0.49 | 0.81 | 0.000408 | 0.36 | 0.31 | 0.38 | SNP |
| chr6_32782018_C_T | 32782018 | 0.55 | 0.39 | 0.76 | 0.000409 | 0.10 | 0.06 | 0.10 | SNP |
| chr6_32188603_C_T | 32188603 | 1.43 | 1.17 | 1.75 | 0.000409 | 0.29 | 0.34 | 0.28 | SNP |
| chr6_31325526_A_G | 31325526 | 1.61 | 1.24 | 2.10 | 0.000412 | 0.33 | 0.36 | 0.32 | SNP |
| chr6_31127562_C_T | 31127562 | 1.66 | 1.25 | 2.19 | 0.000415 | 0.20 | 0.22 | 0.19 | SNP |
| chr6_32627747_G_A | 32627747 | 0.64 | 0.50 | 0.82 | 0.000421 | 0.30 | 0.26 | 0.32 | SNP |
| chr6_32521117_T_C | 32521117 | 0.68 | 0.55 | 0.84 | 0.000437 | 0.49 | 0.41 | 0.51 | SNP |
| chr6_32185796_C_T | 32185796 | 2.16 | 1.40 | 3.30 | 0.000438 | 0.03 | 0.05 | 0.03 | SNP |
| chr6_32611725_G_A | 32611725 | 0.66 | 0.53 | 0.83 | 0.000441 | 0.47 | 0.40 | 0.49 | SNP |
| chr6_31122500_G_A | 31122500 | 1.78 | 1.29 | 2.46 | 0.000443 | 0.19 | 0.23 | 0.18 | SNP |
| chr6_32407433_G_C | 32407433 | 0.67 | 0.53 | 0.83 | 0.000447 | 0.32 | 0.26 | 0.34 | SNP |
| chr6_32521951_A_C | 32521951 | 0.69 | 0.56 | 0.85 | 0.000449 | 0.37 | 0.31 | 0.39 | SNP |
| chr6_32603488_A_G | 32603488 | 0.68 | 0.55 | 0.84 | 0.000450 | 0.27 | 0.20 | 0.29 | SNP |
| chr6_32164587_C_T | 32164587 | 0.39 | 0.22 | 0.64 | 0.000464 | 0.04 | 0.02 | 0.05 | SNP |
| chr6_32633665_T_C | 32633665 | 0.65 | 0.51 | 0.82 | 0.000470 | 0.31 | 0.27 | 0.32 | SNP |
| chr6_32408597_G_A | 32408597 | 0.67 | 0.53 | 0.84 | 0.000476 | 0.32 | 0.26 | 0.34 | SNP |
| chr6_32339498_C_G | 32339498 | 0.40 | 0.24 | 0.65 | 0.000479 | 0.04 | 0.02 | 0.05 | SNP |
| chr6_32408012_G_A | 32408012 | 0.67 | 0.53 | 0.84 | 0.000487 | 0.33 | 0.26 | 0.34 | SNP |
| chr6_32636494_C_T | 32636494 | 0.67 | 0.54 | 0.84 | 0.000492 | 0.42 | 0.35 | 0.44 | SNP |
| chr6_32184094_G_A | 32184094 | 1.40 | 1.16 | 1.69 | 0.000497 | 0.43 | 0.48 | 0.41 | SNP |
| chr6_32410137_C_T | 32410137 | 1.45 | 1.17 | 1.78 | 0.000501 | 0.29 | 0.32 | 0.28 | SNP |
| chr6_32374595_A_G | 32374595 | 0.54 | 0.38 | 0.76 | 0.000503 | 0.18 | 0.15 | 0.19 | SNP |
| chr6_32375796_A_T | 32375796 | 0.54 | 0.38 | 0.76 | 0.000503 | 0.18 | 0.15 | 0.19 | SNP |
| chr6_31325529_C_T | 31325529 | 1.60 | 1.23 | 2.08 | 0.000507 | 0.33 | 0.36 | 0.32 | SNP |
| chr6_31325521_C_G | 31325521 | 1.60 | 1.23 | 2.08 | 0.000509 | 0.33 | 0.36 | 0.32 | SNP |
| chr6_32710407_T_C | 32710407 | 1.86 | 1.31 | 2.64 | 0.000511 | 0.06 | 0.10 | 0.05 | SNP |
| chr6_32501296_G_A | 32501296 | 0.70 | 0.57 | 0.85 | 0.000513 | 0.39 | 0.33 | 0.40 | SNP |
| xHLA_2field_DRB1_04_01 | 32546552 | 1.61 | 1.23 | 2.11 | 0.000513 | 0.13 | 0.20 | 0.11 | HLA (xHLA) |
| chr6_31373445_T_C | 31373445 | 2.33 | 1.43 | 3.73 | 0.000525 | 0.03 | 0.04 | 0.02 | SNP |
| chr6_31326629_T_C | 31326629 | 1.56 | 1.21 | 2.01 | 0.000530 | 0.35 | 0.38 | 0.34 | SNP |
| chr6_32634010_A_G | 32634010 | 0.65 | 0.51 | 0.83 | 0.000540 | 0.31 | 0.26 | 0.32 | SNP |
| chr6_32184221_G_A | 32184221 | 1.40 | 1.16 | 1.69 | 0.000559 | 0.43 | 0.48 | 0.41 | SNP |
| chr6_33045558_G_A | 33045558 | 0.70 | 0.57 | 0.85 | 0.000568 | 0.25 | 0.18 | 0.27 | SNP |
| chr6_32192617_T_C | 32192617 | 0.44 | 0.27 | 0.69 | 0.000570 | 0.05 | 0.03 | 0.06 | SNP |
| chr6_32501301_G_C | 32501301 | 0.70 | 0.57 | 0.85 | 0.000576 | 0.39 | 0.33 | 0.40 | SNP |
| chr6_32780914_G_A | 32780914 | 0.57 | 0.41 | 0.78 | 0.000578 | 0.09 | 0.06 | 0.10 | SNP |
| chr6_32604968_A_C | 32604968 | 0.62 | 0.48 | 0.81 | 0.000592 | 0.25 | 0.21 | 0.26 | SNP |
| chr6_32409056_G_A | 32409056 | 1.44 | 1.17 | 1.77 | 0.000602 | 0.29 | 0.32 | 0.28 | SNP |
| chr6_32188297_G_C | 32188297 | 1.42 | 1.16 | 1.73 | 0.000608 | 0.29 | 0.34 | 0.28 | SNP |
| chr6_32633909_T_A | 32633909 | 0.66 | 0.52 | 0.83 | 0.000612 | 0.33 | 0.28 | 0.34 | SNP |
| chr6_32604538_G_A | 32604538 | 1.51 | 1.19 | 1.91 | 0.000624 | 0.33 | 0.40 | 0.31 | SNP |
| chr6_32149816_T_C | 32149816 | 1.76 | 1.27 | 2.43 | 0.000639 | 0.16 | 0.18 | 0.16 | SNP |
| chr6_32781076_C_A | 32781076 | 0.57 | 0.41 | 0.78 | 0.000642 | 0.09 | 0.06 | 0.10 | SNP |
| chr6_32149537_C_G | 32149537 | 1.74 | 1.26 | 2.40 | 0.000643 | 0.17 | 0.19 | 0.16 | SNP |
| chr6_32627494_T_C | 32627494 | 1.48 | 1.18 | 1.85 | 0.000645 | 0.25 | 0.31 | 0.23 | SNP |
| chr6_32611931_T_A | 32611931 | 0.67 | 0.54 | 0.84 | 0.000650 | 0.46 | 0.40 | 0.48 | SNP |
| chr6_32709964_T_C | 32709964 | 0.69 | 0.55 | 0.85 | 0.000660 | 0.31 | 0.26 | 0.33 | SNP |
| chr6_32550591_G_A | 32550591 | 0.44 | 0.27 | 0.69 | 0.000677 | 0.06 | 0.03 | 0.06 | SNP |
| chr6_32520595_G_A | 32520595 | 0.70 | 0.57 | 0.86 | 0.000680 | 0.38 | 0.32 | 0.39 | SNP |
| chr6_32559045_T_C | 32559045 | 0.71 | 0.58 | 0.86 | 0.000733 | 0.43 | 0.37 | 0.44 | SNP |
| chr6_32609514_T_C | 32609514 | 0.68 | 0.54 | 0.85 | 0.000756 | 0.46 | 0.39 | 0.47 | SNP |
| chr6_31321267_A_C | 31321267 | 2.26 | 1.40 | 3.64 | 0.000766 | 0.12 | 0.13 | 0.12 | SNP |
| chr6_32152442_A_G | 32152442 | 1.73 | 1.25 | 2.38 | 0.000768 | 0.17 | 0.19 | 0.16 | SNP |
| chr6_32633906_A_C | 32633906 | 0.66 | 0.52 | 0.84 | 0.000784 | 0.33 | 0.28 | 0.34 | SNP |
| chr6_29910189_T_A | 29910189 | 1.38 | 1.14 | 1.67 | 0.000794 | 0.37 | 0.40 | 0.36 | SNP |
| chr6_32439753_T_A | 32439753 | 0.66 | 0.51 | 0.84 | 0.000796 | 0.31 | 0.26 | 0.32 | SNP |
| chr6_32628784_A_C | 32628784 | 0.68 | 0.54 | 0.85 | 0.000800 | 0.47 | 0.41 | 0.49 | SNP |
| chr6_29891850_A_G | 29891850 | 1.34 | 1.13 | 1.60 | 0.000827 | 0.43 | 0.46 | 0.42 | SNP |
| chr6_32709990_C_T | 32709990 | 0.69 | 0.56 | 0.86 | 0.000837 | 0.31 | 0.26 | 0.33 | SNP |
| chr6_32441696_C_T | 32441696 | 0.52 | 0.34 | 0.75 | 0.000844 | 0.07 | 0.04 | 0.07 | SNP |
| chr6_32487677_T_C | 32487677 | 1.66 | 1.23 | 2.23 | 0.000857 | 0.16 | 0.22 | 0.14 | SNP |
| chr6_32636221_G_T | 32636221 | 0.68 | 0.54 | 0.85 | 0.000870 | 0.45 | 0.39 | 0.47 | SNP |
| chr6_32261940_G_C | 32261940 | 0.42 | 0.24 | 0.68 | 0.000883 | 0.04 | 0.02 | 0.05 | SNP |
| chr6_32406122_G_A | 32406122 | 1.99 | 1.31 | 2.96 | 0.000908 | 0.03 | 0.05 | 0.03 | SNP |
| chr6_32501311_C_T | 32501311 | 0.71 | 0.58 | 0.87 | 0.000930 | 0.38 | 0.33 | 0.40 | SNP |
| chr6_32408932_G_A | 32408932 | 1.98 | 1.31 | 2.96 | 0.000946 | 0.03 | 0.05 | 0.03 | SNP |
| chr6_32411573_C_T | 32411573 | 1.98 | 1.31 | 2.96 | 0.000946 | 0.03 | 0.05 | 0.03 | SNP |
| chr6_32628098_G_C | 32628098 | 0.44 | 0.27 | 0.70 | 0.000949 | 0.05 | 0.03 | 0.05 | SNP |
| chr6_32628751_T_C | 32628751 | 0.68 | 0.55 | 0.86 | 0.000953 | 0.47 | 0.41 | 0.49 | SNP |
| chr6_29910358_G_C | 29910358 | 1.39 | 1.14 | 1.70 | 0.000967 | 0.46 | 0.49 | 0.45 | SNP |
| chr6_32147696_G_A | 32147696 | 1.72 | 1.24 | 2.37 | 0.000975 | 0.17 | 0.18 | 0.16 | SNP |
| chr6_31323116_C_T | 31323116 | 2.04 | 1.33 | 3.11 | 0.000984 | 0.03 | 0.05 | 0.03 | SNP |
| chr6_32136029_A_G | 32136029 | 2.15 | 1.35 | 3.37 | 0.000992 | 0.03 | 0.05 | 0.03 | SNP |
| chr6_32270222_C_A | 32270222 | 0.43 | 0.25 | 0.69 | 0.000998 | 0.04 | 0.02 | 0.05 | SNP |
| chr6_32783365_T_C | 32783365 | 0.59 | 0.43 | 0.80 | 0.001020 | 0.09 | 0.06 | 0.10 | SNP |
| chr6_32629409_G_A | 32629409 | 0.67 | 0.52 | 0.85 | 0.001030 | 0.32 | 0.27 | 0.33 | SNP |
| chr6_32148031_A_G | 32148031 | 1.71 | 1.24 | 2.35 | 0.001078 | 0.17 | 0.18 | 0.16 | SNP |
| chr6_31322108_T_G | 31322108 | 2.11 | 1.33 | 3.27 | 0.001097 | 0.03 | 0.04 | 0.02 | SNP |
| chr6_32634387_T_C | 32634387 | 0.67 | 0.52 | 0.85 | 0.001113 | 0.31 | 0.27 | 0.32 | SNP |
| chr6_32629582_T_C | 32629582 | 0.67 | 0.52 | 0.85 | 0.001124 | 0.31 | 0.26 | 0.32 | SNP |
| chr6_32628737_G_A | 32628737 | 0.69 | 0.55 | 0.86 | 0.001129 | 0.47 | 0.41 | 0.49 | SNP |
| chr6_32628776_A_C | 32628776 | 0.69 | 0.55 | 0.86 | 0.001131 | 0.47 | 0.41 | 0.49 | SNP |
| chr6_32785029_T_C | 32785029 | 0.59 | 0.43 | 0.80 | 0.001146 | 0.09 | 0.06 | 0.10 | SNP |
| chr6_32322071_G_A | 32322071 | 2.09 | 1.33 | 3.23 | 0.001153 | 0.03 | 0.05 | 0.03 | SNP |
| chr6_32710010_G_T | 32710010 | 0.70 | 0.56 | 0.87 | 0.001159 | 0.31 | 0.26 | 0.33 | SNP |
| chr6_32785035_A_G | 32785035 | 0.59 | 0.43 | 0.81 | 0.001203 | 0.09 | 0.06 | 0.10 | SNP |
| chr6_31248256_C_T | 31248256 | 0.43 | 0.25 | 0.70 | 0.001207 | 0.04 | 0.02 | 0.05 | SNP |
| chr6_29893293_A_G | 29893293 | 1.34 | 1.12 | 1.60 | 0.001209 | 0.48 | 0.51 | 0.47 | SNP |
| chr6_32786516_C_T | 32786516 | 0.55 | 0.38 | 0.78 | 0.001226 | 0.07 | 0.05 | 0.08 | SNP |
| chr6_32550947_A_G | 32550947 | 1.49 | 1.17 | 1.89 | 0.001229 | 0.22 | 0.29 | 0.20 | SNP |
| chr6_32975578_T_C | 32975578 | 1.40 | 1.14 | 1.72 | 0.001245 | 0.16 | 0.21 | 0.15 | SNP |
| chr6_31322611_C_T | 31322611 | 2.09 | 1.32 | 3.24 | 0.001245 | 0.03 | 0.04 | 0.02 | SNP |
| chr6_31323455_A_G | 31323455 | 2.09 | 1.32 | 3.24 | 0.001245 | 0.03 | 0.04 | 0.02 | SNP |
| chr6_32489960_A_G | 32489960 | 1.70 | 1.23 | 2.34 | 0.001248 | 0.06 | 0.09 | 0.06 | SNP |
| chr6_32783406_G_A | 32783406 | 0.55 | 0.38 | 0.78 | 0.001251 | 0.07 | 0.05 | 0.08 | SNP |
| chr6_32783450_C_G | 32783450 | 0.55 | 0.38 | 0.78 | 0.001251 | 0.07 | 0.05 | 0.08 | SNP |
| chr6_31378257_G_A | 31378257 | 2.09 | 1.32 | 3.24 | 0.001256 | 0.03 | 0.04 | 0.02 | SNP |
| chr6_31322987_C_T | 31322987 | 2.09 | 1.32 | 3.24 | 0.001256 | 0.03 | 0.04 | 0.02 | SNP |
| chr6_32171075_A_G | 32171075 | 1.62 | 1.21 | 2.17 | 0.001285 | 0.18 | 0.20 | 0.18 | SNP |
| chr6_31122482_G_A | 31122482 | 1.70 | 1.23 | 2.34 | 0.001308 | 0.19 | 0.23 | 0.19 | SNP |
| xHLA_2field_DQB1_06_04 | 32627244 | 0.42 | 0.24 | 0.70 | 0.001329 | 0.05 | 0.02 | 0.05 | HLA (xHLA) |
| chr6_32303160_G_T | 32303160 | 0.44 | 0.26 | 0.71 | 0.001342 | 0.04 | 0.02 | 0.05 | SNP |
| chr6_32551568_C_G | 32551568 | 0.71 | 0.58 | 0.88 | 0.001350 | 0.38 | 0.33 | 0.40 | SNP |
| chr6_32611971_G_C | 32611971 | 1.46 | 1.16 | 1.85 | 0.001353 | 0.28 | 0.35 | 0.26 | SNP |
| chr6_31430060_G_T | 31430060 | 2.10 | 1.32 | 3.28 | 0.001354 | 0.03 | 0.04 | 0.02 | SNP |
| chr6_32154998_C_T | 32154998 | 1.64 | 1.21 | 2.21 | 0.001369 | 0.18 | 0.21 | 0.18 | SNP |
| chr6_32634834_T_C | 32634834 | 0.69 | 0.55 | 0.87 | 0.001392 | 0.45 | 0.39 | 0.47 | SNP |
| chr6_29891617_A_G | 29891617 | 1.35 | 1.12 | 1.63 | 0.001415 | 0.48 | 0.51 | 0.48 | SNP |
| chr6_32782112_C_T | 32782112 | 2.14 | 1.33 | 3.39 | 0.001418 | 0.02 | 0.04 | 0.02 | SNP |
| chr6_32406473_G_A | 32406473 | 1.51 | 1.17 | 1.94 | 0.001425 | 0.21 | 0.24 | 0.21 | SNP |
| chr6_31125705_C_G | 31125705 | 1.59 | 1.19 | 2.10 | 0.001427 | 0.20 | 0.21 | 0.19 | SNP |
| chr6_32410576_C_T | 32410576 | 1.40 | 1.14 | 1.72 | 0.001463 | 0.29 | 0.32 | 0.28 | SNP |
| chr6_32628765_C_T | 32628765 | 1.39 | 1.13 | 1.70 | 0.001503 | 0.40 | 0.45 | 0.38 | SNP |
| chr6_31325792_G_A | 31325792 | 1.48 | 1.16 | 1.89 | 0.001538 | 0.36 | 0.39 | 0.35 | SNP |
| chr6_32973281_C_T | 32973281 | 1.30 | 1.11 | 1.53 | 0.001545 | 0.48 | 0.52 | 0.47 | SNP |
| chr6_32782149_G_A | 32782149 | 0.55 | 0.38 | 0.79 | 0.001560 | 0.07 | 0.05 | 0.08 | SNP |
| chr6_32145205_C_T | 32145205 | 1.69 | 1.22 | 2.33 | 0.001572 | 0.18 | 0.21 | 0.17 | SNP |
| chr6_29910083_G_A | 29910083 | 1.44 | 1.15 | 1.80 | 0.001579 | 0.18 | 0.20 | 0.17 | SNP |
| chr6_30878857_G_A | 30878857 | 1.68 | 1.21 | 2.30 | 0.001594 | 0.07 | 0.11 | 0.06 | SNP |
| chr6_32190620_T_C | 32190620 | 0.73 | 0.59 | 0.88 | 0.001604 | 0.26 | 0.21 | 0.27 | SNP |
| chr6_33382288_A_G | 33382288 | 1.53 | 1.17 | 1.98 | 0.001637 | 0.10 | 0.13 | 0.09 | SNP |
| chr6_29895697_T_C | 29895697 | 0.76 | 0.64 | 0.90 | 0.001665 | 0.50 | 0.47 | 0.50 | SNP |
| chr6_32607224_C_T | 32607224 | 0.56 | 0.39 | 0.80 | 0.001677 | 0.10 | 0.06 | 0.11 | SNP |
| chr6_31903121_G_A | 31903121 | 0.49 | 0.31 | 0.75 | 0.001678 | 0.06 | 0.03 | 0.06 | SNP |
| chr6_32611450_T_G | 32611450 | 0.70 | 0.55 | 0.87 | 0.001679 | 0.48 | 0.41 | 0.49 | SNP |
| chr6_32606214_C_T | 32606214 | 1.79 | 1.24 | 2.56 | 0.001682 | 0.04 | 0.06 | 0.04 | SNP |
| chr6_32611964_C_T | 32611964 | 1.45 | 1.15 | 1.84 | 0.001701 | 0.28 | 0.35 | 0.27 | SNP |
| chr6_32605295_C_T | 32605295 | 1.41 | 1.14 | 1.75 | 0.001709 | 0.32 | 0.39 | 0.30 | SNP |
| chr6_32636434_G_A | 32636434 | 0.70 | 0.56 | 0.87 | 0.001727 | 0.46 | 0.39 | 0.47 | SNP |
| chr6_31320569_G_T | 31320569 | 0.39 | 0.20 | 0.68 | 0.001748 | 0.04 | 0.02 | 0.04 | SNP |
| chr6_32609312_C_A | 32609312 | 0.69 | 0.55 | 0.87 | 0.001763 | 0.46 | 0.39 | 0.48 | SNP |
| chr6_32557301_T_C | 32557301 | 0.40 | 0.21 | 0.69 | 0.001773 | 0.03 | 0.02 | 0.04 | SNP |
| chr6_32611873_C_T | 32611873 | 0.70 | 0.56 | 0.87 | 0.001780 | 0.47 | 0.40 | 0.48 | SNP |
| chr6_32611874_T_G | 32611874 | 0.70 | 0.56 | 0.87 | 0.001780 | 0.47 | 0.40 | 0.48 | SNP |
| chr6_32628030_G_A | 32628030 | 1.38 | 1.13 | 1.68 | 0.001784 | 0.42 | 0.48 | 0.41 | SNP |
| chr6_33381459_A_G | 33381459 | 0.39 | 0.21 | 0.68 | 0.001786 | 0.04 | 0.02 | 0.05 | SNP |
| chr6_32634838_C_A | 32634838 | 0.70 | 0.56 | 0.87 | 0.001793 | 0.45 | 0.39 | 0.47 | SNP |
| chr6_31243660_G_T | 31243660 | 2.31 | 1.35 | 3.89 | 0.001813 | 0.02 | 0.04 | 0.02 | SNP |
| chr6_32633467_A_G | 32633467 | 0.58 | 0.40 | 0.81 | 0.001821 | 0.09 | 0.05 | 0.10 | SNP |
| chr6_32439915_T_C | 32439915 | 0.67 | 0.52 | 0.86 | 0.001825 | 0.29 | 0.25 | 0.30 | SNP |
| chr6_32604372_A_G | 32604372 | 0.70 | 0.56 | 0.87 | 0.001846 | 0.46 | 0.40 | 0.48 | SNP |
| chr6_33052981_C_A | 33052981 | 1.49 | 1.16 | 1.91 | 0.001847 | 0.11 | 0.13 | 0.11 | SNP |
| chr6_32974843_C_T | 32974843 | 1.38 | 1.12 | 1.69 | 0.001864 | 0.16 | 0.21 | 0.15 | SNP |
| chr6_32627833_A_G | 32627833 | 1.37 | 1.12 | 1.67 | 0.001883 | 0.41 | 0.47 | 0.40 | SNP |
| chr6_32489964_G_A | 32489964 | 1.66 | 1.20 | 2.27 | 0.001902 | 0.07 | 0.09 | 0.06 | SNP |
| chr6_32789400_A_G | 32789400 | 0.44 | 0.25 | 0.72 | 0.001907 | 0.04 | 0.02 | 0.05 | SNP |
| chr6_32611504_C_T | 32611504 | 0.70 | 0.56 | 0.88 | 0.001929 | 0.47 | 0.41 | 0.49 | SNP |
| chr6_31116210_G_A | 31116210 | 1.50 | 1.16 | 1.93 | 0.001932 | 0.24 | 0.26 | 0.24 | SNP |
| chr6_32973599_C_T | 32973599 | 1.38 | 1.12 | 1.69 | 0.001954 | 0.16 | 0.21 | 0.15 | SNP |
| chr6_31378864_A_G | 31378864 | 2.06 | 1.29 | 3.24 | 0.001966 | 0.03 | 0.04 | 0.02 | SNP |
| chr6_32975699_G_C | 32975699 | 1.39 | 1.13 | 1.71 | 0.002022 | 0.16 | 0.21 | 0.15 | SNP |
| chr6_29909988_T_C | 29909988 | 1.34 | 1.11 | 1.61 | 0.002027 | 0.36 | 0.39 | 0.36 | SNP |

| | | | | | | | | | |
|---|---|---|---|---|---|---|---|---|---|
| chr6_32973794_C_T | 32973794 | 1.37 | 1.12 | 1.68 | 0.002033 | 0.16 | 0.21 | 0.15 | SNP |
| chr6_32629802_G_A | 32629802 | 0.70 | 0.56 | 0.88 | 0.002033 | 0.45 | 0.39 | 0.47 | SNP |
| chr6_31324375_G_T | 31324375 | 0.55 | 0.37 | 0.80 | 0.002044 | 0.09 | 0.06 | 0.09 | SNP |
| chr6_32607174_C_T | 32607174 | 1.42 | 1.14 | 1.77 | 0.002046 | 0.35 | 0.41 | 0.33 | SNP |
| chr6_31237918_G_A | 31237918 | 0.54 | 0.36 | 0.79 | 0.002048 | 0.08 | 0.04 | 0.09 | SNP |
| chr6_32612397_A_G | 32612397 | 1.41 | 1.13 | 1.76 | 0.002049 | 0.47 | 0.54 | 0.45 | SNP |
| chr6_32627489_G_A | 32627489 | 0.68 | 0.54 | 0.87 | 0.002052 | 0.43 | 0.37 | 0.45 | SNP |
| chr6_32186050_G_A | 32186050 | 1.41 | 1.13 | 1.76 | 0.002071 | 0.41 | 0.46 | 0.40 | SNP |
| chr6_32628439_G_A | 32628439 | 1.37 | 1.12 | 1.68 | 0.002075 | 0.40 | 0.46 | 0.39 | SNP |
| chr6_32628736_T_C | 32628736 | 0.70 | 0.56 | 0.88 | 0.002089 | 0.47 | 0.41 | 0.49 | SNP |
| chr6_31321776_T_C | 31321776 | 2.03 | 1.28 | 3.17 | 0.002096 | 0.03 | 0.04 | 0.02 | SNP |
| chr6_29893131_T_C | 29893131 | 0.36 | 0.17 | 0.65 | 0.002131 | 0.03 | 0.01 | 0.04 | SNP |
| chr6_32520774_C_T | 32520774 | 1.42 | 1.14 | 1.78 | 0.002140 | 0.30 | 0.37 | 0.29 | SNP |
| chr6_33590009_A_T | 33590009 | 0.51 | 0.33 | 0.77 | 0.002145 | 0.06 | 0.03 | 0.06 | SNP |
| chr6_32605705_T_G | 32605705 | 0.71 | 0.57 | 0.88 | 0.002186 | 0.46 | 0.40 | 0.47 | SNP |
| chr6_32607031_G_T | 32607031 | 1.44 | 1.14 | 1.82 | 0.002189 | 0.32 | 0.40 | 0.30 | SNP |
| chr6_32605708_A_G | 32605708 | 0.71 | 0.57 | 0.88 | 0.002219 | 0.46 | 0.40 | 0.47 | SNP |
| chr6_32607221_C_G | 32607221 | 0.57 | 0.40 | 0.81 | 0.002235 | 0.09 | 0.06 | 0.10 | SNP |
| chr6_33542661_G_T | 33542661 | 0.74 | 0.60 | 0.89 | 0.002261 | 0.25 | 0.21 | 0.27 | SNP |
| chr6_29895973_G_C | 29895973 | 1.42 | 1.13 | 1.79 | 0.002277 | 0.18 | 0.20 | 0.17 | SNP |
| chr6_31322690_A_G | 31322690 | 2.02 | 1.27 | 3.15 | 0.002291 | 0.03 | 0.04 | 0.02 | SNP |
| chr6_29944670_G_C | 29944670 | 1.37 | 1.12 | 1.69 | 0.002304 | 0.47 | 0.50 | 0.46 | SNP |
| chr6_32605274_C_T | 32605274 | 0.71 | 0.57 | 0.88 | 0.002311 | 0.45 | 0.39 | 0.47 | SNP |
| chr6_31324750_G_A | 31324750 | 0.56 | 0.39 | 0.81 | 0.002318 | 0.09 | 0.06 | 0.10 | SNP |
| chr6_32633285_T_C | 32633285 | 0.72 | 0.58 | 0.89 | 0.002330 | 0.36 | 0.31 | 0.37 | SNP |
| chr6_32608467_G_A | 32608467 | 1.42 | 1.13 | 1.78 | 0.002331 | 0.29 | 0.36 | 0.27 | SNP |
| chr6_32604000_T_G | 32604000 | 0.71 | 0.56 | 0.88 | 0.002358 | 0.46 | 0.39 | 0.47 | SNP |
| chr6_32817774_T_G | 32817774 | 0.68 | 0.53 | 0.87 | 0.002389 | 0.17 | 0.14 | 0.18 | SNP |
| chr6_32603098_T_C | 32603098 | 1.36 | 1.12 | 1.66 | 0.002414 | 0.48 | 0.55 | 0.46 | SNP |
| chr6_33548493_C_T | 33548493 | 0.73 | 0.60 | 0.89 | 0.002424 | 0.33 | 0.27 | 0.35 | SNP |
| chr6_32406704_G_T | 32406704 | 1.49 | 1.15 | 1.92 | 0.002467 | 0.21 | 0.24 | 0.21 | SNP |
| chr6_29944706_G_A | 29944706 | 1.51 | 1.15 | 1.97 | 0.002484 | 0.15 | 0.17 | 0.14 | SNP |
| chr6_33365640_C_T | 33365640 | 1.44 | 1.14 | 1.82 | 0.002484 | 0.13 | 0.16 | 0.12 | SNP |
| chr6_33639760_C_G | 33639760 | 0.75 | 0.62 | 0.90 | 0.002493 | 0.33 | 0.28 | 0.35 | SNP |
| chr6_32606085_T_A | 32606085 | 0.71 | 0.57 | 0.89 | 0.002512 | 0.45 | 0.39 | 0.47 | SNP |
| chr6_32604564_C_T | 32604564 | 1.43 | 1.13 | 1.81 | 0.002514 | 0.33 | 0.40 | 0.31 | SNP |
| chr6_31371071_C_A | 31371071 | 2.09 | 1.28 | 3.36 | 0.002531 | 0.03 | 0.05 | 0.02 | SNP |
| chr6_32635226_G_T | 32635226 | 0.71 | 0.56 | 0.89 | 0.002609 | 0.46 | 0.40 | 0.48 | SNP |
| chr6_31326703_T_C | 31326703 | 2.09 | 1.29 | 3.38 | 0.002621 | 0.12 | 0.13 | 0.12 | SNP |
| chr6_31566507_G_A | 31566507 | 0.50 | 0.31 | 0.77 | 0.002648 | 0.05 | 0.03 | 0.05 | SNP |
| chr6_32627535_T_G | 32627535 | 0.70 | 0.56 | 0.88 | 0.002659 | 0.45 | 0.39 | 0.47 | SNP |
| chr6_29908838_C_G | 29908838 | 1.33 | 1.10 | 1.59 | 0.002672 | 0.37 | 0.40 | 0.37 | SNP |
| chr6_32603412_T_C | 32603412 | 1.36 | 1.11 | 1.66 | 0.002686 | 0.48 | 0.55 | 0.46 | SNP |
| chr6_31910938_G_T | 31910938 | 0.59 | 0.41 | 0.82 | 0.002688 | 0.08 | 0.05 | 0.09 | SNP |
| chr6_32627714_G_T | 32627714 | 0.71 | 0.57 | 0.89 | 0.002701 | 0.45 | 0.39 | 0.47 | SNP |
| chr6_31326074_A_T | 31326074 | 2.09 | 1.29 | 3.37 | 0.002750 | 0.12 | 0.13 | 0.11 | SNP |
| chr6_32629314_A_G | 32629314 | 0.71 | 0.56 | 0.89 | 0.002756 | 0.47 | 0.41 | 0.49 | SNP |
| chr6_32520465_A_G | 32520465 | 0.73 | 0.59 | 0.90 | 0.002765 | 0.38 | 0.33 | 0.39 | SNP |
| chr6_32609105_A_G | 32609105 | 0.71 | 0.57 | 0.89 | 0.002767 | 0.46 | 0.40 | 0.48 | SNP |
| chr6_32634990_G_T | 32634990 | 0.59 | 0.42 | 0.83 | 0.002768 | 0.08 | 0.05 | 0.09 | SNP |
| chr6_32362447_C_T | 32362447 | 1.89 | 1.23 | 2.85 | 0.002776 | 0.03 | 0.05 | 0.03 | SNP |
| chr6_32373796_A_G | 32373796 | 1.89 | 1.23 | 2.85 | 0.002776 | 0.03 | 0.05 | 0.03 | SNP |
| chr6_29912368_T_C | 29912368 | 1.33 | 1.10 | 1.60 | 0.002794 | 0.38 | 0.40 | 0.37 | SNP |
| chr6_32603968_G_A | 32603968 | 0.71 | 0.57 | 0.89 | 0.002796 | 0.45 | 0.39 | 0.47 | SNP |
| chr6_29908954_A_G | 29908954 | 1.33 | 1.10 | 1.60 | 0.002805 | 0.37 | 0.40 | 0.37 | SNP |
| chr6_31325116_T_G | 31325116 | 1.51 | 1.15 | 1.97 | 0.002847 | 0.34 | 0.37 | 0.33 | SNP |
| chr6_29909807_G_T | 29909807 | 1.41 | 1.12 | 1.76 | 0.002886 | 0.18 | 0.20 | 0.17 | SNP |
| chr6_29909840_A_G | 29909840 | 1.31 | 1.10 | 1.57 | 0.002889 | 0.46 | 0.48 | 0.45 | SNP |
| chr6_32634103_C_T | 32634103 | 0.71 | 0.57 | 0.89 | 0.002895 | 0.45 | 0.39 | 0.47 | SNP |
| chr6_29909153_T_G | 29909153 | 1.32 | 1.10 | 1.59 | 0.002908 | 0.37 | 0.40 | 0.37 | SNP |
| chr6_31935464_C_G | 31935464 | 0.43 | 0.23 | 0.72 | 0.002915 | 0.04 | 0.02 | 0.04 | SNP |
| chr6_32611958_C_G | 32611958 | 1.43 | 1.13 | 1.81 | 0.002927 | 0.28 | 0.34 | 0.27 | SNP |
| chr6_33256471_C_T | 33256471 | 1.43 | 1.13 | 1.81 | 0.002975 | 0.13 | 0.16 | 0.12 | SNP |
| chr6_29909745_G_A | 29909745 | 1.32 | 1.10 | 1.59 | 0.002978 | 0.37 | 0.40 | 0.37 | SNP |
| chr6_32605754_A_T | 32605754 | 0.71 | 0.57 | 0.89 | 0.002982 | 0.46 | 0.40 | 0.47 | SNP |
| chr6_31118019_T_C | 31118019 | 1.44 | 1.13 | 1.84 | 0.002993 | 0.26 | 0.27 | 0.26 | SNP |
| chr6_29913542_G_T | 29913542 | 1.40 | 1.12 | 1.75 | 0.003021 | 0.18 | 0.20 | 0.18 | SNP |
| chr6_31910929_G_A | 31910929 | 0.59 | 0.41 | 0.83 | 0.003023 | 0.08 | 0.05 | 0.09 | SNP |
| chr6_32713267_A_G | 32713267 | 1.52 | 1.15 | 1.99 | 0.003040 | 0.09 | 0.11 | 0.08 | SNP |
| chr6_29912280_C_T | 29912280 | 1.33 | 1.10 | 1.60 | 0.003080 | 0.38 | 0.40 | 0.37 | SNP |
| chr6_31248324_C_T | 31248324 | 0.45 | 0.26 | 0.75 | 0.003091 | 0.04 | 0.02 | 0.05 | SNP |
| chr6_32611243_C_T | 32611243 | 1.41 | 1.12 | 1.77 | 0.003092 | 0.28 | 0.35 | 0.27 | SNP |
| chr6_29912830_C_T | 29912830 | 1.39 | 1.12 | 1.73 | 0.003110 | 0.19 | 0.21 | 0.18 | SNP |
| chr6_32607008_T_G | 32607008 | 0.72 | 0.57 | 0.89 | 0.003122 | 0.45 | 0.39 | 0.47 | SNP |
| chr6_31244332_C_T | 31244332 | 0.48 | 0.29 | 0.76 | 0.003127 | 0.04 | 0.02 | 0.05 | SNP |
| chr6_32627600_T_G | 32627600 | 0.71 | 0.57 | 0.89 | 0.003132 | 0.45 | 0.39 | 0.47 | SNP |
| chr6_32636289_T_C | 32636289 | 1.40 | 1.12 | 1.75 | 0.003191 | 0.23 | 0.29 | 0.22 | SNP |
| chr6_31326080_T_C | 31326080 | 0.61 | 0.44 | 0.84 | 0.003199 | 0.09 | 0.07 | 0.09 | SNP |
| chr6_33545340_G_A | 33545340 | 0.72 | 0.57 | 0.89 | 0.003214 | 0.26 | 0.20 | 0.27 | SNP |
| chr6_32520665_A_C | 32520665 | 0.73 | 0.60 | 0.90 | 0.003220 | 0.37 | 0.32 | 0.39 | SNP |
| chr6_29909616_C_T | 29909616 | 1.32 | 1.10 | 1.59 | 0.003223 | 0.37 | 0.40 | 0.37 | SNP |
| chr6_31248091_G_A | 31248091 | 0.45 | 0.25 | 0.74 | 0.003235 | 0.04 | 0.02 | 0.05 | SNP |
| chr6_29909033_G_A | 29909033 | 1.32 | 1.10 | 1.59 | 0.003241 | 0.37 | 0.39 | 0.36 | SNP |
| chr6_32163274_G_A | 32163274 | 1.83 | 1.21 | 2.72 | 0.003260 | 0.03 | 0.05 | 0.03 | SNP |
| chr6_32166380_C_G | 32166380 | 1.83 | 1.21 | 2.72 | 0.003260 | 0.03 | 0.05 | 0.03 | SNP |
| chr6_32134828_C_T | 32134828 | 0.42 | 0.22 | 0.72 | 0.003264 | 0.04 | 0.02 | 0.05 | SNP |
| chr6_31321211_C_T | 31321211 | 0.76 | 0.63 | 0.91 | 0.003275 | 0.41 | 0.37 | 0.42 | SNP |
| chr6_33039535_A_T | 33039535 | 1.45 | 1.13 | 1.85 | 0.003298 | 0.12 | 0.13 | 0.11 | SNP |
| chr6_33543409_A_C | 33543409 | 1.30 | 1.09 | 1.55 | 0.003314 | 0.40 | 0.47 | 0.39 | SNP |
| chr6_32627517_G_T | 32627517 | 0.71 | 0.56 | 0.89 | 0.003327 | 0.45 | 0.39 | 0.47 | SNP |
| chr6_29892615_C_T | 29892615 | 0.78 | 0.66 | 0.92 | 0.003336 | 0.50 | 0.47 | 0.50 | SNP |
| chr6_32628941_G_C | 32628941 | 1.39 | 1.12 | 1.74 | 0.003344 | 0.23 | 0.29 | 0.22 | SNP |
| chr6_32442296_C_T | 32442296 | 0.69 | 0.54 | 0.88 | 0.003356 | 0.29 | 0.25 | 0.30 | SNP |
| chr6_31241933_A_C | 31241933 | 1.56 | 1.16 | 2.10 | 0.003386 | 0.08 | 0.11 | 0.07 | SNP |
| chr6_32611373_A_G | 32611373 | 0.71 | 0.56 | 0.89 | 0.003388 | 0.49 | 0.43 | 0.51 | SNP |
| chr6_32605943_A_G | 32605943 | 0.72 | 0.57 | 0.89 | 0.003392 | 0.45 | 0.39 | 0.47 | SNP |
| chr6_29798610_T_C | 29798610 | 1.40 | 1.12 | 1.75 | 0.003399 | 0.18 | 0.20 | 0.17 | SNP |
| chr6_31325115_G_A | 31325115 | 1.50 | 1.14 | 1.96 | 0.003418 | 0.34 | 0.37 | 0.34 | SNP |
| chr6_33382943_T_G | 33382943 | 1.52 | 1.14 | 2.00 | 0.003434 | 0.10 | 0.12 | 0.09 | SNP |
| chr6_31236201_G_A | 31236201 | 1.48 | 1.13 | 1.91 | 0.003440 | 0.10 | 0.12 | 0.09 | SNP |
| chr6_29909472_C_G | 29909472 | 1.32 | 1.10 | 1.58 | 0.003449 | 0.37 | 0.40 | 0.37 | SNP |
| chr6_33136076_G_A | 33136076 | 1.49 | 1.14 | 1.95 | 0.003466 | 0.11 | 0.12 | 0.10 | SNP |
| chr6_32490732_G_A | 32490732 | 2.14 | 1.27 | 3.54 | 0.003479 | 0.02 | 0.04 | 0.02 | SNP |
| chr6_32610059_G_T | 32610059 | 0.72 | 0.57 | 0.90 | 0.003485 | 0.45 | 0.39 | 0.47 | SNP |
| chr6_29909751_C_T | 29909751 | 1.30 | 1.09 | 1.56 | 0.003512 | 0.46 | 0.48 | 0.45 | SNP |
| chr6_31116142_G_A | 31116142 | 1.45 | 1.13 | 1.86 | 0.003538 | 0.26 | 0.27 | 0.26 | SNP |
| chr6_32526540_A_G | 32526540 | 0.74 | 0.61 | 0.91 | 0.003551 | 0.41 | 0.36 | 0.42 | SNP |
| chr6_31241207_C_T | 31241207 | 0.74 | 0.60 | 0.90 | 0.003560 | 0.29 | 0.26 | 0.29 | SNP |
| chr6_31430065_C_T | 31430065 | 2.09 | 1.27 | 3.44 | 0.003604 | 0.12 | 0.13 | 0.11 | SNP |
| chr6_32627485_C_T | 32627485 | 0.70 | 0.55 | 0.89 | 0.003609 | 0.43 | 0.37 | 0.45 | SNP |
| chr6_32977044_G_A | 32977044 | 1.49 | 1.14 | 1.95 | 0.003619 | 0.09 | 0.10 | 0.09 | SNP |
| chr6_29892196_T_A | 29892196 | 1.31 | 1.09 | 1.56 | 0.003691 | 0.47 | 0.49 | 0.47 | SNP |
| chr6_29913171_A_G | 29913171 | 1.31 | 1.09 | 1.58 | 0.003701 | 0.38 | 0.40 | 0.37 | SNP |
| chr6_33281843_A_T | 33281843 | 1.31 | 1.09 | 1.58 | 0.003716 | 0.34 | 0.39 | 0.32 | SNP |
| chr6_31902645_A_G | 31902645 | 0.53 | 0.34 | 0.80 | 0.003720 | 0.05 | 0.03 | 0.06 | SNP |
| chr6_32603487_C_T | 32603487 | 0.72 | 0.58 | 0.90 | 0.003734 | 0.45 | 0.39 | 0.47 | SNP |
| chr6_31118942_T_C | 31118942 | 1.44 | 1.12 | 1.84 | 0.003747 | 0.26 | 0.27 | 0.26 | SNP |
| chr6_32306922_T_C | 32306922 | 1.85 | 1.21 | 2.79 | 0.003752 | 0.03 | 0.05 | 0.03 | SNP |
| chr6_29892709_A_G | 29892709 | 0.78 | 0.65 | 0.92 | 0.003786 | 0.50 | 0.48 | 0.50 | SNP |
| chr6_31325092_G_A | 31325092 | 1.49 | 1.14 | 1.94 | 0.003809 | 0.34 | 0.37 | 0.33 | SNP |
| chr6_29857730_C_G | 29857730 | 1.37 | 1.11 | 1.70 | 0.003810 | 0.18 | 0.21 | 0.18 | SNP |
| chr6_29908667_G_A | 29908667 | 1.38 | 1.11 | 1.72 | 0.003850 | 0.19 | 0.21 | 0.18 | SNP |
| chr6_32826985_C_T | 32826985 | 0.77 | 0.64 | 0.92 | 0.003876 | 0.43 | 0.39 | 0.44 | SNP |
| chr6_31915532_C_T | 31915532 | 0.45 | 0.25 | 0.75 | 0.003909 | 0.04 | 0.02 | 0.04 | SNP |
| chr6_31239999_A_T | 31239999 | 0.57 | 0.38 | 0.83 | 0.003969 | 0.08 | 0.05 | 0.09 | SNP |
| chr6_29909181_C_A | 29909181 | 1.38 | 1.11 | 1.72 | 0.004001 | 0.19 | 0.21 | 0.18 | SNP |
| chr6_29909559_A_G | 29909559 | 1.30 | 1.09 | 1.55 | 0.004051 | 0.46 | 0.48 | 0.45 | SNP |
| chr6_33281842_G_T | 33281842 | 1.31 | 1.09 | 1.58 | 0.004063 | 0.34 | 0.39 | 0.32 | SNP |
| chr6_32405671_C_A | 32405671 | 1.34 | 1.10 | 1.64 | 0.004071 | 0.32 | 0.35 | 0.31 | SNP |
| chr6_29893461_A_G | 29893461 | 1.37 | 1.10 | 1.71 | 0.004083 | 0.19 | 0.21 | 0.18 | SNP |
| chr6_32076499_G_A | 32076499 | 1.35 | 1.10 | 1.66 | 0.004091 | 0.37 | 0.43 | 0.36 | SNP |
| chr6_32612111_G_A | 32612111 | 1.39 | 1.11 | 1.75 | 0.004104 | 0.29 | 0.36 | 0.27 | SNP |
| chr6_29909197_C_G | 29909197 | 1.39 | 1.11 | 1.73 | 0.004156 | 0.18 | 0.20 | 0.18 | SNP |
| chr6_33542523_T_C | 33542523 | 1.31 | 1.09 | 1.58 | 0.004171 | 0.22 | 0.26 | 0.21 | SNP |
| chr6_32976654_G_A | 32976654 | 1.48 | 1.13 | 1.94 | 0.004176 | 0.09 | 0.10 | 0.09 | SNP |
| chr6_32187605_G_A | 32187605 | 0.74 | 0.60 | 0.91 | 0.004189 | 0.48 | 0.42 | 0.49 | SNP |
| chr6_29799103_G_A | 29799103 | 1.39 | 1.11 | 1.74 | 0.004193 | 0.18 | 0.20 | 0.17 | SNP |
| chr6_32627646_G_A | 32627646 | 0.72 | 0.57 | 0.90 | 0.004209 | 0.45 | 0.39 | 0.47 | SNP |
| chr6_29893040_T_C | 29893040 | 1.37 | 1.10 | 1.71 | 0.004241 | 0.20 | 0.22 | 0.19 | SNP |
| chr6_29913672_A_G | 29913672 | 1.38 | 1.10 | 1.72 | 0.004259 | 0.19 | 0.21 | 0.19 | SNP |
| chr6_32803576_A_G | 32803576 | 1.54 | 1.14 | 2.07 | 0.004263 | 0.07 | 0.11 | 0.06 | SNP |
| chr6_32490746_C_A | 32490746 | 2.07 | 1.24 | 3.38 | 0.004269 | 0.02 | 0.04 | 0.02 | SNP |
| chr6_29909447_C_G | 29909447 | 1.30 | 1.08 | 1.55 | 0.004288 | 0.46 | 0.48 | 0.45 | SNP |
| chr6_32975381_G_A | 32975381 | 0.77 | 0.64 | 0.92 | 0.004297 | 0.40 | 0.35 | 0.41 | SNP |
| chr6_31383130_T_A | 31383130 | 0.66 | 0.49 | 0.87 | 0.004298 | 0.12 | 0.09 | 0.12 | SNP |
| chr6_32609195_G_A | 32609195 | 0.61 | 0.43 | 0.85 | 0.004305 | 0.09 | 0.06 | 0.09 | SNP |
| chr6_32520639_G_A | 32520639 | 1.38 | 1.11 | 1.72 | 0.004312 | 0.31 | 0.38 | 0.29 | SNP |
| chr6_33636660_C_G | 33636660 | 1.33 | 1.09 | 1.63 | 0.004323 | 0.33 | 0.38 | 0.32 | SNP |
| chr6_32612104_C_T | 32612104 | 1.39 | 1.11 | 1.74 | 0.004326 | 0.29 | 0.36 | 0.27 | SNP |
| chr6_32608473_G_A | 32608473 | 1.39 | 1.11 | 1.74 | 0.004351 | 0.29 | 0.36 | 0.28 | SNP |
| chr6_32611472_G_A | 32611472 | 0.72 | 0.57 | 0.90 | 0.004373 | 0.47 | 0.41 | 0.48 | SNP |
| chr6_29913042_T_C | 29913042 | 1.38 | 1.11 | 1.73 | 0.004375 | 0.18 | 0.20 | 0.18 | SNP |
| chr6_32490747_T_A | 32490747 | 2.06 | 1.24 | 3.37 | 0.004377 | 0.02 | 0.04 | 0.02 | SNP |
| chr6_32609453_C_T | 32609453 | 1.39 | 1.11 | 1.75 | 0.004445 | 0.33 | 0.40 | 0.32 | SNP |
| chr6_29796768_C_T | 29796768 | 1.35 | 1.10 | 1.66 | 0.004462 | 0.22 | 0.24 | 0.21 | SNP |

| | | | | | | | | | |
|---|---|---|---|---|---|---|---|---|---|
| chr6_31507160_A_G | 31507160 | 0.40 | 0.20 | 0.72 | 0.004484 | 0.03 | 0.02 | 0.04 | SNP |
| chr6_32151420_G_C | 32151420 | 0.58 | 0.39 | 0.83 | 0.004490 | 0.06 | 0.04 | 0.07 | SNP |
| chr6_31902289_C_G | 31902289 | 0.54 | 0.34 | 0.81 | 0.004546 | 0.05 | 0.03 | 0.06 | SNP |
| chr6_32628916_G_A | 32628916 | 1.38 | 1.10 | 1.71 | 0.004548 | 0.23 | 0.29 | 0.22 | SNP |
| chr6_32608967_A_G | 32608967 | 0.61 | 0.43 | 0.85 | 0.004558 | 0.09 | 0.06 | 0.10 | SNP |
| chr6_32636280_G_T | 32636280 | 1.38 | 1.10 | 1.73 | 0.004573 | 0.23 | 0.29 | 0.22 | SNP |
| chr6_32612161_C_A | 32612161 | 1.39 | 1.11 | 1.74 | 0.004593 | 0.29 | 0.36 | 0.27 | SNP |
| chr6_32627777_G_C | 32627777 | 1.37 | 1.10 | 1.70 | 0.004633 | 0.24 | 0.29 | 0.22 | SNP |
| chr6_32605825_G_A | 32605825 | 1.51 | 1.13 | 1.99 | 0.004637 | 0.08 | 0.10 | 0.07 | SNP |
| chr6_32605920_A_G | 32605920 | 0.72 | 0.58 | 0.90 | 0.004677 | 0.45 | 0.39 | 0.47 | SNP |
| chr6_32628571_C_T | 32628571 | 1.39 | 1.11 | 1.75 | 0.004686 | 0.23 | 0.28 | 0.21 | SNP |
| chr6_32606176_T_A | 32606176 | 0.72 | 0.58 | 0.90 | 0.004692 | 0.45 | 0.39 | 0.47 | SNP |
| chr6_32976185_G_A | 32976185 | 0.39 | 0.20 | 0.72 | 0.004702 | 0.03 | 0.01 | 0.03 | SNP |
| chr6_31248114_C_T | 31248114 | 0.48 | 0.28 | 0.78 | 0.004709 | 0.04 | 0.03 | 0.05 | SNP |
| xHLA_2field_B_40_01 | 31321652 | 0.63 | 0.45 | 0.86 | 0.004740 | 0.09 | 0.07 | 0.09 | HLA (xHLA) |
| chr6_32604394_T_C | 32604394 | 1.39 | 1.11 | 1.74 | 0.004742 | 0.29 | 0.36 | 0.28 | SNP |
| chr6_32611234_C_T | 32611234 | 1.38 | 1.10 | 1.74 | 0.004761 | 0.28 | 0.35 | 0.27 | SNP |
| chr6_32612166_C_T | 32612166 | 1.39 | 1.10 | 1.74 | 0.004773 | 0.29 | 0.35 | 0.27 | SNP |
| chr6_29912935_T_C | 29912935 | 1.39 | 1.10 | 1.74 | 0.004798 | 0.17 | 0.19 | 0.17 | SNP |
| chr6_32526702_C_T | 32526702 | 0.60 | 0.42 | 0.85 | 0.004865 | 0.08 | 0.05 | 0.09 | SNP |
| chr6_32628944_C_G | 32628944 | 1.39 | 1.10 | 1.75 | 0.004869 | 0.36 | 0.42 | 0.34 | SNP |
| chr6_32605067_C_G | 32605067 | 1.50 | 1.13 | 1.98 | 0.004874 | 0.08 | 0.10 | 0.08 | SNP |
| chr6_31323506_C_T | 31323506 | 1.94 | 1.22 | 3.08 | 0.004894 | 0.12 | 0.13 | 0.12 | SNP |
| chr6_32409530_A_G | 32409530 | 1.31 | 1.08 | 1.57 | 0.004957 | 0.40 | 0.43 | 0.39 | SNP |
| chr6_32604395_A_G | 32604395 | 1.50 | 1.13 | 1.99 | 0.004957 | 0.08 | 0.10 | 0.07 | SNP |
| chr6_29852855_T_C | 29852855 | 1.39 | 1.10 | 1.75 | 0.004991 | 0.18 | 0.20 | 0.17 | SNP |
| chr6_33596664_A_C | 33596664 | 0.55 | 0.35 | 0.82 | 0.005004 | 0.05 | 0.03 | 0.06 | SNP |
| chr6_32604431_A_T | 32604431 | 1.50 | 1.13 | 1.99 | 0.005029 | 0.08 | 0.10 | 0.07 | SNP |
| chr6_32611759_T_G | 32611759 | 0.62 | 0.44 | 0.86 | 0.005073 | 0.09 | 0.06 | 0.09 | SNP |
| chr6_32605326_A_G | 32605326 | 1.50 | 1.12 | 1.98 | 0.005128 | 0.08 | 0.10 | 0.08 | SNP |
| chr6_31321919_G_T | 31321919 | 1.72 | 1.17 | 2.49 | 0.005130 | 0.04 | 0.06 | 0.04 | SNP |
| chr6_32604974_C_T | 32604974 | 1.50 | 1.12 | 1.99 | 0.005153 | 0.08 | 0.10 | 0.07 | SNP |
| chr6_32608417_G_A | 32608417 | 1.38 | 1.10 | 1.73 | 0.005190 | 0.29 | 0.36 | 0.27 | SNP |
| chr6_29913638_C_G | 29913638 | 1.37 | 1.10 | 1.71 | 0.005215 | 0.19 | 0.21 | 0.18 | SNP |
| chr6_32609126_C_T | 32609126 | 0.73 | 0.58 | 0.91 | 0.005224 | 0.46 | 0.40 | 0.48 | SNP |
| chr6_29913727_C_T | 29913727 | 1.37 | 1.10 | 1.70 | 0.005251 | 0.20 | 0.21 | 0.19 | SNP |
| chr6_32793721_G_C | 32793721 | 1.63 | 1.15 | 2.29 | 0.005273 | 0.06 | 0.10 | 0.05 | SNP |
| chr6_32609476_T_C | 32609476 | 1.50 | 1.12 | 1.99 | 0.005289 | 0.08 | 0.10 | 0.07 | SNP |
| chr6_29758726_G_C | 29758726 | 1.37 | 1.10 | 1.71 | 0.005292 | 0.19 | 0.21 | 0.18 | SNP |
| chr6_32611510_C_G | 32611510 | 0.73 | 0.58 | 0.91 | 0.005342 | 0.47 | 0.41 | 0.48 | SNP |
| chr6_32629755_G_A | 32629755 | 1.36 | 1.10 | 1.70 | 0.005343 | 0.24 | 0.29 | 0.22 | SNP |
| chr6_31112602_G_C | 31112602 | 1.41 | 1.11 | 1.80 | 0.005418 | 0.26 | 0.27 | 0.26 | SNP |
| chr6_32784676_C_T | 32784676 | 1.59 | 1.14 | 2.21 | 0.005426 | 0.06 | 0.10 | 0.05 | SNP |
| chr6_32628506_C_A | 32628506 | 1.38 | 1.10 | 1.73 | 0.005430 | 0.23 | 0.28 | 0.21 | SNP |
| chr6_32793534_T_C | 32793534 | 1.62 | 1.15 | 2.28 | 0.005434 | 0.06 | 0.10 | 0.05 | SNP |
| chr6_32520621_G_A | 32520621 | 1.36 | 1.10 | 1.69 | 0.005472 | 0.31 | 0.38 | 0.29 | SNP |
| chr6_31924880_G_A | 31924880 | 0.61 | 0.43 | 0.86 | 0.005474 | 0.09 | 0.06 | 0.09 | SNP |
| chr6_33219142_A_C | 33219142 | 0.76 | 0.63 | 0.92 | 0.005501 | 0.34 | 0.28 | 0.35 | SNP |
| chr6_31836827_G_T | 31836827 | 1.48 | 1.12 | 1.95 | 0.005513 | 0.33 | 0.35 | 0.33 | SNP |
| chr6_32609427_C_T | 32609427 | 0.73 | 0.58 | 0.91 | 0.005606 | 0.46 | 0.39 | 0.47 | SNP |
| chr6_32628760_C_A | 32628760 | 1.37 | 1.10 | 1.71 | 0.005609 | 0.23 | 0.29 | 0.22 | SNP |
| chr6_32634007_A_G | 32634007 | 1.36 | 1.09 | 1.70 | 0.005629 | 0.24 | 0.29 | 0.22 | SNP |
| chr6_32376788_C_T | 32376788 | 1.36 | 1.09 | 1.68 | 0.005657 | 0.45 | 0.49 | 0.44 | SNP |
| chr6_31245534_A_G | 31245534 | 1.45 | 1.11 | 1.88 | 0.005668 | 0.10 | 0.12 | 0.09 | SNP |
| chr6_32559674_T_A | 32559674 | 1.52 | 1.12 | 2.03 | 0.005681 | 0.08 | 0.10 | 0.07 | SNP |
| chr6_29758117_G_A | 29758117 | 1.37 | 1.09 | 1.71 | 0.005692 | 0.19 | 0.21 | 0.19 | SNP |
| chr6_31606756_A_T | 31606756 | 0.66 | 0.48 | 0.88 | 0.005702 | 0.10 | 0.08 | 0.11 | SNP |
| chr6_32610300_C_T | 32610300 | 0.62 | 0.44 | 0.86 | 0.005708 | 0.08 | 0.06 | 0.09 | SNP |
| chr6_31321360_G_A | 31321360 | 1.58 | 1.14 | 2.18 | 0.005742 | 0.19 | 0.21 | 0.18 | SNP |
| chr6_29897759_A_G | 29897759 | 1.37 | 1.09 | 1.71 | 0.005744 | 0.20 | 0.22 | 0.19 | SNP |
| chr6_32559124_T_C | 32559124 | 1.39 | 1.10 | 1.75 | 0.005762 | 0.28 | 0.35 | 0.26 | SNP |
| chr6_29896268_C_T | 29896268 | 1.29 | 1.08 | 1.54 | 0.005789 | 0.44 | 0.46 | 0.43 | SNP |
| chr6_29910286_T_C | 29910286 | 1.37 | 1.10 | 1.72 | 0.005797 | 0.19 | 0.21 | 0.19 | SNP |
| chr6_32608483_T_C | 32608483 | 1.37 | 1.10 | 1.72 | 0.005798 | 0.29 | 0.36 | 0.27 | SNP |
| chr6_29910176_T_C | 29910176 | 1.31 | 1.08 | 1.58 | 0.005807 | 0.39 | 0.41 | 0.38 | SNP |
| chr6_32612241_A_T | 32612241 | 1.37 | 1.10 | 1.72 | 0.005811 | 0.29 | 0.35 | 0.27 | SNP |
| chr6_31326948_C_A | 31326948 | 0.66 | 0.49 | 0.88 | 0.005813 | 0.11 | 0.08 | 0.11 | SNP |
| chr6_32605759_A_T | 32605759 | 0.73 | 0.59 | 0.91 | 0.005825 | 0.46 | 0.40 | 0.47 | SNP |
| chr6_32605689_C_A | 32605689 | 1.49 | 1.12 | 1.97 | 0.005851 | 0.08 | 0.10 | 0.07 | SNP |
| chr6_32825379_A_G | 32825379 | 0.78 | 0.65 | 0.93 | 0.005859 | 0.45 | 0.41 | 0.46 | SNP |
| chr6_32520664_G_A | 32520664 | 1.36 | 1.09 | 1.69 | 0.005863 | 0.31 | 0.38 | 0.29 | SNP |
| chr6_32636351_T_C | 32636351 | 1.36 | 1.09 | 1.70 | 0.005867 | 0.24 | 0.29 | 0.22 | SNP |
| chr6_32611769_G_C | 32611769 | 1.40 | 1.10 | 1.77 | 0.005873 | 0.31 | 0.38 | 0.29 | SNP |
| chr6_32629639_T_C | 32629639 | 1.36 | 1.09 | 1.69 | 0.005879 | 0.24 | 0.29 | 0.22 | SNP |
| chr6_31110391_G_C | 31110391 | 1.40 | 1.10 | 1.79 | 0.005884 | 0.26 | 0.27 | 0.26 | SNP |
| chr6_32412580_C_T | 32412580 | 1.41 | 1.10 | 1.79 | 0.005890 | 0.23 | 0.25 | 0.22 | SNP |
| chr6_32606960_A_T | 32606960 | 0.62 | 0.43 | 0.86 | 0.005907 | 0.08 | 0.05 | 0.09 | SNP |
| chr6_32636217_G_A | 32636217 | 1.36 | 1.09 | 1.70 | 0.006017 | 0.23 | 0.29 | 0.22 | SNP |
| chr6_32605861_A_G | 32605861 | 0.62 | 0.44 | 0.86 | 0.006018 | 0.08 | 0.05 | 0.09 | SNP |
| chr6_29896680_G_A | 29896680 | 1.28 | 1.07 | 1.53 | 0.006022 | 0.49 | 0.51 | 0.49 | SNP |
| chr6_32611265_C_T | 32611265 | 1.37 | 1.09 | 1.72 | 0.006044 | 0.29 | 0.35 | 0.27 | SNP |
| chr6_31326683_G_A | 31326683 | 0.67 | 0.50 | 0.89 | 0.006064 | 0.11 | 0.08 | 0.11 | SNP |
| chr6_32085278_C_T | 32085278 | 1.92 | 1.19 | 3.03 | 0.006069 | 0.03 | 0.04 | 0.03 | SNP |
| chr6_32635230_G_A | 32635230 | 1.37 | 1.09 | 1.73 | 0.006100 | 0.23 | 0.28 | 0.22 | SNP |
| chr6_32976147_T_A | 32976147 | 0.39 | 0.19 | 0.73 | 0.006103 | 0.03 | 0.01 | 0.03 | SNP |
| chr6_32633415_C_T | 32633415 | 1.48 | 1.12 | 1.95 | 0.006119 | 0.16 | 0.18 | 0.16 | SNP |
| chr6_31323416_G_C | 31323416 | 1.91 | 1.20 | 3.02 | 0.006143 | 0.12 | 0.13 | 0.12 | SNP |
| chr6_32612096_A_G | 32612096 | 0.62 | 0.44 | 0.87 | 0.006158 | 0.08 | 0.06 | 0.09 | SNP |
| chr6_32550819_C_A | 32550819 | 1.37 | 1.09 | 1.72 | 0.006201 | 0.29 | 0.35 | 0.27 | SNP |
| chr6_32441150_C_T | 32441150 | 1.37 | 1.09 | 1.72 | 0.006211 | 0.30 | 0.36 | 0.28 | SNP |
| chr6_32605950_C_T | 32605950 | 0.62 | 0.44 | 0.86 | 0.006228 | 0.08 | 0.05 | 0.09 | SNP |
| chr6_32781112_C_T | 32781112 | 1.62 | 1.14 | 2.28 | 0.006237 | 0.06 | 0.09 | 0.05 | SNP |
| chr6_29855120_T_G | 29855120 | 1.30 | 1.08 | 1.57 | 0.006252 | 0.31 | 0.33 | 0.31 | SNP |
| chr6_32559212_A_G | 32559212 | 1.38 | 1.10 | 1.74 | 0.006260 | 0.29 | 0.35 | 0.27 | SNP |
| chr6_32817130_A_G | 32817130 | 0.73 | 0.58 | 0.91 | 0.006278 | 0.18 | 0.15 | 0.19 | SNP |
| chr6_31473247_C_T | 31473247 | 0.46 | 0.26 | 0.78 | 0.006313 | 0.03 | 0.02 | 0.04 | SNP |
| chr6_31932209_G_A | 31932209 | 0.43 | 0.23 | 0.76 | 0.006316 | 0.04 | 0.02 | 0.04 | SNP |
| chr6_32633819_A_G | 32633819 | 0.73 | 0.58 | 0.91 | 0.006322 | 0.50 | 0.54 | 0.49 | SNP |
| chr6_32610105_A_G | 32610105 | 1.50 | 1.12 | 1.99 | 0.006322 | 0.08 | 0.10 | 0.07 | SNP |
| chr6_32608946_G_A | 32608946 | 0.63 | 0.44 | 0.87 | 0.006340 | 0.08 | 0.06 | 0.09 | SNP |
| chr6_31237605_G_A | 31237605 | 1.32 | 1.08 | 1.60 | 0.006385 | 0.21 | 0.25 | 0.20 | SNP |
| chr6_31934372_C_T | 31934372 | 0.64 | 0.46 | 0.88 | 0.006404 | 0.12 | 0.08 | 0.12 | SNP |
| chr6_32610330_A_T | 32610330 | 1.48 | 1.11 | 1.96 | 0.006416 | 0.08 | 0.10 | 0.07 | SNP |
| chr6_32611215_C_T | 32611215 | 0.63 | 0.45 | 0.87 | 0.006422 | 0.08 | 0.06 | 0.09 | SNP |
| chr6_31244082_C_T | 31244082 | 1.45 | 1.10 | 1.88 | 0.006520 | 0.10 | 0.12 | 0.09 | SNP |
| chr6_31540957_T_C | 31540957 | 0.46 | 0.26 | 0.78 | 0.006525 | 0.03 | 0.02 | 0.04 | SNP |
| chr6_31538277_G_A | 31538277 | 0.46 | 0.26 | 0.78 | 0.006565 | 0.03 | 0.02 | 0.04 | SNP |
| chr6_31919956_T_C | 31919956 | 0.62 | 0.44 | 0.87 | 0.006565 | 0.08 | 0.06 | 0.09 | SNP |
| chr6_32611650_G_A | 32611650 | 1.38 | 1.09 | 1.74 | 0.006573 | 0.28 | 0.34 | 0.27 | SNP |
| chr6_32171279_C_T | 32171279 | 1.74 | 1.16 | 2.58 | 0.006582 | 0.03 | 0.05 | 0.03 | SNP |
| chr6_32171288_C_T | 32171288 | 1.74 | 1.16 | 2.58 | 0.006582 | 0.03 | 0.05 | 0.03 | SNP |
| xHLA_2field_B_08_01 | 31321652 | 1.96 | 1.20 | 3.17 | 0.006587 | 0.12 | 0.13 | 0.11 | HLA (xHLA) |
| chr6_31326929_A_C | 31326929 | 0.67 | 0.50 | 0.89 | 0.006606 | 0.11 | 0.08 | 0.11 | SNP |
| chr6_32606742_A_G | 32606742 | 0.62 | 0.44 | 0.87 | 0.006614 | 0.08 | 0.05 | 0.09 | SNP |
| xHLA_2field_DQB1_03_03 | 32627244 | 1.74 | 1.15 | 2.58 | 0.006626 | 0.04 | 0.06 | 0.04 | HLA (xHLA) |
| chr6_33625696_T_C | 33625696 | 1.35 | 1.09 | 1.67 | 0.006632 | 0.26 | 0.31 | 0.25 | SNP |
| chr6_31916951_T_C | 31916951 | 0.63 | 0.44 | 0.87 | 0.006633 | 0.08 | 0.06 | 0.09 | SNP |
| chr6_31919917_T_C | 31919917 | 0.63 | 0.44 | 0.87 | 0.006633 | 0.08 | 0.06 | 0.09 | SNP |
| chr6_32604931_C_A | 32604931 | 1.36 | 1.09 | 1.70 | 0.006642 | 0.30 | 0.37 | 0.28 | SNP |
| chr6_32607017_T_A | 32607017 | 0.62 | 0.44 | 0.87 | 0.006677 | 0.08 | 0.06 | 0.09 | SNP |
| chr6_32629543_T_C | 32629543 | 1.35 | 1.09 | 1.67 | 0.006744 | 0.24 | 0.29 | 0.23 | SNP |
| chr6_31325745_C_T | 31325745 | 1.30 | 1.07 | 1.56 | 0.006752 | 0.47 | 0.52 | 0.45 | SNP |
| chr6_32154285_A_G | 32154285 | 1.51 | 1.12 | 2.04 | 0.006755 | 0.19 | 0.20 | 0.18 | SNP |
| chr6_31248995_C_T | 31248995 | 0.50 | 0.29 | 0.80 | 0.006762 | 0.05 | 0.03 | 0.05 | SNP |
| chr6_32303474_G_T | 32303474 | 1.82 | 1.17 | 2.79 | 0.006772 | 0.03 | 0.04 | 0.03 | SNP |
| chr6_32611466_A_G | 32611466 | 1.38 | 1.09 | 1.74 | 0.006778 | 0.27 | 0.34 | 0.25 | SNP |
| chr6_32611474_G_A | 32611474 | 1.38 | 1.09 | 1.74 | 0.006783 | 0.27 | 0.34 | 0.25 | SNP |
| chr6_29909916_T_A | 29909916 | 1.30 | 1.07 | 1.57 | 0.006790 | 0.39 | 0.41 | 0.39 | SNP |
| chr6_29945949_G_A | 29945949 | 1.55 | 1.13 | 2.12 | 0.006792 | 0.11 | 0.13 | 0.11 | SNP |
| chr6_32809848_G_A | 32809848 | 1.38 | 1.09 | 1.75 | 0.006796 | 0.14 | 0.19 | 0.13 | SNP |
| chr6_32812528_C_T | 32812528 | 1.33 | 1.08 | 1.63 | 0.006810 | 0.16 | 0.20 | 0.16 | SNP |
| chr6_32603405_C_T | 32603405 | 0.62 | 0.44 | 0.87 | 0.006815 | 0.08 | 0.06 | 0.09 | SNP |
| chr6_32819865_T_C | 32819865 | 0.70 | 0.54 | 0.90 | 0.006821 | 0.13 | 0.10 | 0.13 | SNP |
| chr6_32628605_T_C | 32628605 | 1.37 | 1.09 | 1.72 | 0.006835 | 0.23 | 0.28 | 0.22 | SNP |
| chr6_32634091_G_C | 32634091 | 1.35 | 1.09 | 1.68 | 0.006845 | 0.24 | 0.29 | 0.22 | SNP |
| chr6_31543758_G_A | 31543758 | 1.86 | 1.17 | 2.90 | 0.006849 | 0.03 | 0.04 | 0.03 | SNP |
| chr6_32608934_A_T | 32608934 | 0.63 | 0.44 | 0.87 | 0.006860 | 0.08 | 0.06 | 0.09 | SNP |
| chr6_30864279_C_T | 30864279 | 1.75 | 1.16 | 2.63 | 0.006907 | 0.12 | 0.13 | 0.11 | SNP |
| chr6_29892543_C_A | 29892543 | 1.27 | 1.07 | 1.51 | 0.006963 | 0.46 | 0.48 | 0.46 | SNP |
| chr6_31238851_C_T | 31238851 | 1.49 | 1.12 | 2.00 | 0.006971 | 0.32 | 0.34 | 0.31 | SNP |
| chr6_29856178_T_C | 29856178 | 1.42 | 1.10 | 1.83 | 0.006985 | 0.18 | 0.20 | 0.18 | SNP |
| chr6_31932177_C_T | 31932177 | 0.65 | 0.47 | 0.88 | 0.006989 | 0.12 | 0.08 | 0.12 | SNP |
| chr6_29902373_G_A | 29902373 | 1.30 | 1.07 | 1.57 | 0.007011 | 0.40 | 0.42 | 0.39 | SNP |
| chr6_32606676_T_C | 32606676 | 0.62 | 0.44 | 0.87 | 0.007017 | 0.08 | 0.05 | 0.09 | SNP |
| chr6_32634090_T_C | 32634090 | 1.35 | 1.09 | 1.68 | 0.007021 | 0.24 | 0.29 | 0.22 | SNP |
| chr6_32608425_C_A | 32608425 | 0.62 | 0.44 | 0.87 | 0.007040 | 0.08 | 0.06 | 0.09 | SNP |
| chr6_32609874_T_G | 32609874 | 0.63 | 0.45 | 0.87 | 0.007100 | 0.08 | 0.06 | 0.09 | SNP |
| chr6_32610075_A_G | 32610075 | 0.63 | 0.45 | 0.87 | 0.007100 | 0.08 | 0.06 | 0.09 | SNP |
| chr6_32610313_C_G | 32610313 | 0.63 | 0.45 | 0.87 | 0.007100 | 0.08 | 0.06 | 0.09 | SNP |
| chr6_29911538_A_G | 29911538 | 1.29 | 1.07 | 1.56 | 0.007114 | 0.37 | 0.38 | 0.36 | SNP |
| chr6_33548394_G_T | 33548394 | 1.32 | 1.08 | 1.62 | 0.007114 | 0.22 | 0.26 | 0.21 | SNP |
| chr6_32610688_G_C | 32610688 | 0.63 | 0.45 | 0.87 | 0.007135 | 0.08 | 0.06 | 0.09 | SNP |
| chr6_29857852_G_A | 29857852 | 1.33 | 1.08 | 1.64 | 0.007151 | 0.19 | 0.21 | 0.18 | SNP |
| chr6_32634425_C_T | 32634425 | 1.35 | 1.08 | 1.68 | 0.007152 | 0.24 | 0.29 | 0.22 | SNP |
| chr6_32629422_T_C | 32629422 | 1.35 | 1.08 | 1.68 | 0.007187 | 0.24 | 0.29 | 0.22 | SNP |

| | | | | | | | | | |
|---|---|---|---|---|---|---|---|---|---|
| chr6_32612279_T_C | 32612279 | 1.36 | 1.09 | 1.71 | 0.007200 | 0.29 | 0.35 | 0.27 | SNP |
| chr6_32608537_A_C | 32608537 | 1.36 | 1.09 | 1.71 | 0.007222 | 0.29 | 0.36 | 0.27 | SNP |
| xHLA_2field_C_06_02 | 31236526 | 1.54 | 1.12 | 2.11 | 0.007240 | 0.06 | 0.08 | 0.06 | HLA (xHLA) |
| chr6_32604384_G_A | 32604384 | 0.62 | 0.44 | 0.87 | 0.007265 | 0.08 | 0.06 | 0.09 | SNP |
| chr6_32610696_G_A | 32610696 | 0.63 | 0.45 | 0.88 | 0.007272 | 0.08 | 0.06 | 0.09 | SNP |
| xHLA_2field_A_03_01 | 29910309 | 1.39 | 1.09 | 1.76 | 0.007285 | 0.18 | 0.19 | 0.17 | HLA (xHLA) |
| chr6_32635033_T_G | 32635033 | 1.35 | 1.08 | 1.67 | 0.007310 | 0.24 | 0.29 | 0.22 | SNP |
| chr6_32633899_T_C | 32633899 | 1.34 | 1.08 | 1.66 | 0.007354 | 0.24 | 0.29 | 0.23 | SNP |
| chr6_31930351_A_G | 31930351 | 0.54 | 0.33 | 0.83 | 0.007368 | 0.05 | 0.03 | 0.05 | SNP |
| chr6_31237449_C_T | 31237449 | 1.45 | 1.10 | 1.90 | 0.007397 | 0.32 | 0.35 | 0.31 | SNP |
| chr6_32559444_C_T | 32559444 | 0.65 | 0.47 | 0.88 | 0.007430 | 0.09 | 0.06 | 0.10 | SNP |
| chr6_32634094_T_C | 32634094 | 1.35 | 1.08 | 1.68 | 0.007431 | 0.24 | 0.29 | 0.22 | SNP |
| chr6_31901773_G_A | 31901773 | 0.47 | 0.26 | 0.80 | 0.007438 | 0.03 | 0.02 | 0.04 | SNP |
| chr6_29913074_G_A | 29913074 | 1.35 | 1.08 | 1.68 | 0.007479 | 0.19 | 0.21 | 0.19 | SNP |
| chr6_32629398_G_C | 32629398 | 1.35 | 1.08 | 1.68 | 0.007491 | 0.24 | 0.29 | 0.23 | SNP |
| chr6_32611403_G_A | 32611403 | 1.37 | 1.09 | 1.71 | 0.007514 | 0.27 | 0.33 | 0.25 | SNP |
| chr6_32635949_G_A | 32635949 | 1.32 | 1.08 | 1.61 | 0.007608 | 0.29 | 0.34 | 0.27 | SNP |
| chr6_32633636_G_A | 32633636 | 1.34 | 1.08 | 1.67 | 0.007629 | 0.24 | 0.29 | 0.23 | SNP |
| chr6_31325317_C_T | 31325317 | 0.67 | 0.50 | 0.89 | 0.007634 | 0.10 | 0.08 | 0.11 | SNP |
| chr6_32550931_C_A | 32550931 | 1.38 | 1.09 | 1.74 | 0.007703 | 0.23 | 0.29 | 0.21 | SNP |
| chr6_30879987_C_T | 30879987 | 1.74 | 1.16 | 2.62 | 0.007815 | 0.12 | 0.13 | 0.11 | SNP |
| chr6_32785142_G_A | 32785142 | 1.58 | 1.12 | 2.20 | 0.007836 | 0.06 | 0.10 | 0.05 | SNP |
| chr6_32633781_C_T | 32633781 | 1.34 | 1.08 | 1.67 | 0.007880 | 0.24 | 0.29 | 0.23 | SNP |
| chr6_33546498_C_T | 33546498 | 1.29 | 1.07 | 1.56 | 0.007890 | 0.22 | 0.25 | 0.21 | SNP |
| chr6_32823257_T_C | 32823257 | 0.79 | 0.67 | 0.94 | 0.007907 | 0.45 | 0.41 | 0.46 | SNP |
| chr6_32606779_G_A | 32606779 | 1.47 | 1.10 | 1.95 | 0.007962 | 0.08 | 0.10 | 0.08 | SNP |
| chr6_29913078_G_A | 29913078 | 1.35 | 1.08 | 1.68 | 0.007983 | 0.19 | 0.21 | 0.19 | SNP |
| chr6_32629530_C_T | 32629530 | 1.34 | 1.08 | 1.67 | 0.007986 | 0.24 | 0.29 | 0.23 | SNP |
| chr6_33365724_A_G | 33365724 | 0.62 | 0.43 | 0.87 | 0.007987 | 0.07 | 0.05 | 0.08 | SNP |
| chr6_31321407_C_T | 31321407 | 0.68 | 0.51 | 0.90 | 0.008000 | 0.11 | 0.08 | 0.12 | SNP |
| chr6_32804381_A_T | 32804381 | 0.57 | 0.37 | 0.85 | 0.008012 | 0.06 | 0.04 | 0.06 | SNP |
| chr6_32822975_T_A | 32822975 | 0.79 | 0.66 | 0.94 | 0.008013 | 0.43 | 0.40 | 0.44 | SNP |
| chr6_32633767_G_T | 32633767 | 1.34 | 1.08 | 1.67 | 0.008015 | 0.24 | 0.29 | 0.22 | SNP |
| chr6_32605724_A_G | 32605724 | 1.36 | 1.08 | 1.70 | 0.008023 | 0.29 | 0.35 | 0.27 | SNP |
| chr6_32340596_T_A | 32340596 | 0.53 | 0.32 | 0.83 | 0.008097 | 0.04 | 0.03 | 0.05 | SNP |
| chr6_32634098_C_G | 32634098 | 1.34 | 1.08 | 1.67 | 0.008132 | 0.24 | 0.29 | 0.23 | SNP |
| chr6_32611709_T_C | 32611709 | 0.64 | 0.45 | 0.88 | 0.008141 | 0.08 | 0.06 | 0.09 | SNP |
| chr6_32944094_G_C | 32944094 | 1.43 | 1.09 | 1.87 | 0.008163 | 0.09 | 0.12 | 0.08 | SNP |
| chr6_31546495_G_C | 31546495 | 1.83 | 1.16 | 2.85 | 0.008175 | 0.03 | 0.04 | 0.03 | SNP |
| chr6_32612251_T_G | 32612251 | 1.36 | 1.08 | 1.70 | 0.008178 | 0.29 | 0.35 | 0.27 | SNP |
| chr6_31560565_C_T | 31560565 | 0.47 | 0.26 | 0.80 | 0.008271 | 0.03 | 0.02 | 0.04 | SNP |
| chr6_31324064_T_A | 31324064 | 0.61 | 0.42 | 0.87 | 0.008278 | 0.09 | 0.07 | 0.09 | SNP |
| chr6_30229694_A_C | 30229694 | 0.72 | 0.56 | 0.92 | 0.008405 | 0.19 | 0.14 | 0.20 | SNP |
| chr6_29854703_G_A | 29854703 | 1.33 | 1.07 | 1.63 | 0.008421 | 0.19 | 0.21 | 0.18 | SNP |
| chr6_32612326_C_T | 32612326 | 1.36 | 1.08 | 1.70 | 0.008451 | 0.29 | 0.35 | 0.27 | SNP |
| chr6_32611760_G_T | 32611760 | 1.36 | 1.08 | 1.71 | 0.008496 | 0.29 | 0.35 | 0.27 | SNP |
| chr6_32627635_G_T | 32627635 | 0.74 | 0.59 | 0.92 | 0.008514 | 0.45 | 0.39 | 0.46 | SNP |
| chr6_32610968_A_G | 32610968 | 1.36 | 1.08 | 1.71 | 0.008519 | 0.27 | 0.32 | 0.25 | SNP |
| chr6_31928306_A_G | 31928306 | 0.63 | 0.45 | 0.88 | 0.008539 | 0.08 | 0.06 | 0.09 | SNP |
| chr6_31509980_G_T | 31509980 | 1.88 | 1.16 | 2.98 | 0.008549 | 0.03 | 0.04 | 0.03 | SNP |
| chr6_32822907_C_T | 32822907 | 0.79 | 0.67 | 0.94 | 0.008557 | 0.45 | 0.41 | 0.46 | SNP |
| chr6_30857894_G_A | 30857894 | 1.72 | 1.15 | 2.58 | 0.008583 | 0.12 | 0.13 | 0.11 | SNP |
| chr6_30867527_G_A | 30867527 | 1.72 | 1.15 | 2.58 | 0.008583 | 0.12 | 0.13 | 0.11 | SNP |
| chr6_32611260_A_G | 32611260 | 1.36 | 1.08 | 1.70 | 0.008586 | 0.29 | 0.35 | 0.27 | SNP |
| chr6_32804798_G_A | 32804798 | 1.31 | 1.07 | 1.59 | 0.008592 | 0.27 | 0.34 | 0.26 | SNP |
| chr6_32603729_A_G | 32603729 | 1.47 | 1.10 | 1.94 | 0.008620 | 0.08 | 0.10 | 0.08 | SNP |
| chr6_32628787_C_T | 32628787 | 1.35 | 1.08 | 1.69 | 0.008628 | 0.23 | 0.29 | 0.22 | SNP |
| chr6_32607270_A_G | 32607270 | 0.63 | 0.44 | 0.88 | 0.008690 | 0.08 | 0.06 | 0.09 | SNP |
| chr6_31248693_C_G | 31248693 | 0.47 | 0.26 | 0.81 | 0.008738 | 0.04 | 0.02 | 0.04 | SNP |
| chr6_32608820_T_C | 32608820 | 0.74 | 0.59 | 0.93 | 0.008787 | 0.46 | 0.40 | 0.48 | SNP |
| chr6_32635172_C_T | 32635172 | 1.34 | 1.08 | 1.66 | 0.008799 | 0.24 | 0.29 | 0.23 | SNP |
| chr6_32635266_T_G | 32635266 | 1.35 | 1.08 | 1.70 | 0.008808 | 0.23 | 0.28 | 0.21 | SNP |
| chr6_32822858_G_T | 32822858 | 0.79 | 0.67 | 0.94 | 0.008908 | 0.43 | 0.40 | 0.44 | SNP |
| chr6_29913948_T_G | 29913948 | 1.29 | 1.07 | 1.55 | 0.008921 | 0.38 | 0.40 | 0.38 | SNP |
| chr6_31830593_A_C | 31830593 | 0.47 | 0.26 | 0.80 | 0.008935 | 0.04 | 0.03 | 0.05 | SNP |
| chr6_29913944_C_T | 29913944 | 1.29 | 1.06 | 1.55 | 0.008938 | 0.38 | 0.40 | 0.38 | SNP |
| chr6_32609341_T_C | 32609341 | 0.74 | 0.58 | 0.93 | 0.009074 | 0.48 | 0.43 | 0.49 | SNP |
| chr6_31930462_G_A | 31930462 | 0.70 | 0.54 | 0.91 | 0.009077 | 0.14 | 0.11 | 0.14 | SNP |
| chr6_32634556_G_A | 32634556 | 1.35 | 1.08 | 1.68 | 0.009078 | 0.24 | 0.29 | 0.23 | SNP |
| chr6_32376471_A_C | 32376471 | 1.37 | 1.08 | 1.72 | 0.009083 | 0.19 | 0.20 | 0.19 | SNP |
| chr6_31238522_C_T | 31238522 | 1.31 | 1.07 | 1.61 | 0.009084 | 0.21 | 0.24 | 0.20 | SNP |
| chr6_32629859_G_A | 32629859 | 0.74 | 0.59 | 0.93 | 0.009089 | 0.49 | 0.44 | 0.50 | SNP |
| chr6_32607047_T_G | 32607047 | 1.36 | 1.08 | 1.71 | 0.009096 | 0.31 | 0.38 | 0.30 | SNP |
| xHLA_2field_B_57_01 | 31321652 | 1.87 | 1.16 | 2.98 | 0.009110 | 0.03 | 0.04 | 0.02 | HLA (xHLA) |
| chr6_32606266_T_C | 32606266 | 1.46 | 1.09 | 1.94 | 0.009144 | 0.08 | 0.10 | 0.08 | SNP |
| chr6_30879636_C_T | 30879636 | 1.75 | 1.15 | 2.66 | 0.009151 | 0.11 | 0.13 | 0.11 | SNP |
| chr6_32522698_G_T | 32522698 | 0.75 | 0.61 | 0.93 | 0.009159 | 0.41 | 0.37 | 0.42 | SNP |
| chr6_31751071_C_T | 31751071 | 1.84 | 1.15 | 2.89 | 0.009162 | 0.03 | 0.04 | 0.03 | SNP |
| chr6_31084684_G_A | 31084684 | 0.77 | 0.63 | 0.94 | 0.009181 | 0.49 | 0.45 | 0.50 | SNP |
| chr6_32634057_C_T | 32634057 | 1.34 | 1.07 | 1.67 | 0.009326 | 0.24 | 0.29 | 0.22 | SNP |
| chr6_31325764_C_G | 31325764 | 0.69 | 0.51 | 0.91 | 0.009341 | 0.11 | 0.09 | 0.12 | SNP |
| chr6_32013500_T_C | 32013500 | 0.64 | 0.46 | 0.89 | 0.009438 | 0.09 | 0.07 | 0.10 | SNP |
| chr6_32604059_A_G | 32604059 | 1.34 | 1.07 | 1.68 | 0.009442 | 0.29 | 0.36 | 0.28 | SNP |
| chr6_32634593_G_C | 32634593 | 1.34 | 1.07 | 1.66 | 0.009486 | 0.24 | 0.28 | 0.22 | SNP |
| chr6_32407537_C_T | 32407537 | 0.53 | 0.32 | 0.84 | 0.009508 | 0.04 | 0.03 | 0.05 | SNP |
| chr6_32798647_A_T | 32798647 | 0.50 | 0.29 | 0.82 | 0.009527 | 0.04 | 0.02 | 0.04 | SNP |
| chr6_31528130_C_T | 31528130 | 1.51 | 1.10 | 2.05 | 0.009708 | 0.08 | 0.09 | 0.08 | SNP |
| chr6_32712666_T_C | 32712666 | 1.49 | 1.10 | 2.02 | 0.009712 | 0.14 | 0.16 | 0.14 | SNP |
| chr6_32191457_G_A | 32191457 | 0.74 | 0.59 | 0.93 | 0.009809 | 0.18 | 0.15 | 0.19 | SNP |
| chr6_32634355_G_A | 32634355 | 1.34 | 1.07 | 1.67 | 0.009901 | 0.24 | 0.29 | 0.22 | SNP |
| chr6_33046662_T_C | 33046662 | 0.76 | 0.62 | 0.93 | 0.009914 | 0.23 | 0.18 | 0.25 | SNP |
| chr6_32634646_T_C | 32634646 | 1.33 | 1.07 | 1.66 | 0.010012 | 0.24 | 0.29 | 0.22 | SNP |
| chr6_32783405_C_T | 32783405 | 1.55 | 1.11 | 2.15 | 0.010109 | 0.06 | 0.10 | 0.05 | SNP |
| chr6_31707526_G_A | 31707526 | 1.82 | 1.14 | 2.85 | 0.010124 | 0.03 | 0.04 | 0.02 | SNP |
| chr6_31240060_G_A | 31240060 | 1.43 | 1.09 | 1.88 | 0.010177 | 0.33 | 0.35 | 0.32 | SNP |
| chr6_29857890_C_T | 29857890 | 1.29 | 1.06 | 1.56 | 0.010197 | 0.24 | 0.26 | 0.23 | SNP |
| chr6_32612041_A_G | 32612041 | 1.35 | 1.07 | 1.70 | 0.010204 | 0.29 | 0.35 | 0.27 | SNP |
| chr6_32603939_G_T | 32603939 | 1.33 | 1.07 | 1.64 | 0.010217 | 0.33 | 0.35 | 0.32 | SNP |
| chr6_32629066_T_C | 32629066 | 1.34 | 1.07 | 1.68 | 0.010227 | 0.23 | 0.28 | 0.22 | SNP |
| chr6_32604259_C_T | 32604259 | 1.35 | 1.07 | 1.69 | 0.010272 | 0.29 | 0.36 | 0.28 | SNP |
| chr6_32604544_C_T | 32604544 | 1.35 | 1.07 | 1.69 | 0.010307 | 0.32 | 0.34 | 0.31 | SNP |
| chr6_32635713_C_T | 32635713 | 0.75 | 0.60 | 0.93 | 0.010334 | 0.48 | 0.42 | 0.49 | SNP |
| chr6_32612008_T_C | 32612008 | 1.35 | 1.07 | 1.71 | 0.010360 | 0.28 | 0.34 | 0.27 | SNP |
| chr6_32610976_A_C | 32610976 | 0.65 | 0.46 | 0.89 | 0.010455 | 0.08 | 0.06 | 0.09 | SNP |
| chr6_29914034_A_G | 29914034 | 1.29 | 1.06 | 1.57 | 0.010501 | 0.39 | 0.41 | 0.39 | SNP |
| chr6_32785841_C_T | 32785841 | 1.54 | 1.10 | 2.15 | 0.010574 | 0.06 | 0.10 | 0.05 | SNP |
| chr6_31244230_C_A | 31244230 | 1.57 | 1.11 | 2.21 | 0.010617 | 0.18 | 0.20 | 0.17 | SNP |
| chr6_32633617_A_G | 32633617 | 0.75 | 0.60 | 0.93 | 0.010635 | 0.45 | 0.39 | 0.46 | SNP |
| chr6_32604108_T_C | 32604108 | 1.34 | 1.07 | 1.68 | 0.010723 | 0.29 | 0.36 | 0.28 | SNP |
| chr6_32490708_A_C | 32490708 | 2.21 | 1.18 | 4.02 | 0.010748 | 0.02 | 0.03 | 0.01 | SNP |
| chr6_32155581_A_G | 32155581 | 1.37 | 1.07 | 1.75 | 0.010839 | 0.23 | 0.27 | 0.22 | SNP |
| chr6_31894355_C_T | 31894355 | 0.68 | 0.50 | 0.91 | 0.010848 | 0.10 | 0.08 | 0.10 | SNP |
| chr6_32628556_C_T | 32628556 | 1.35 | 1.07 | 1.71 | 0.010934 | 0.20 | 0.25 | 0.19 | SNP |
| chr6_29892104_A_G | 29892104 | 1.25 | 1.05 | 1.49 | 0.010936 | 0.49 | 0.50 | 0.48 | SNP |
| chr6_32635248_A_T | 32635248 | 1.35 | 1.07 | 1.69 | 0.010952 | 0.23 | 0.27 | 0.21 | SNP |
| chr6_29897379_C_G | 29897379 | 1.25 | 1.05 | 1.48 | 0.010991 | 0.47 | 0.49 | 0.46 | SNP |
| chr6_29942983_A_G | 29942983 | 1.27 | 1.06 | 1.52 | 0.011049 | 0.46 | 0.48 | 0.46 | SNP |
| chr6_32634139_C_T | 32634139 | 1.33 | 1.07 | 1.65 | 0.011067 | 0.24 | 0.29 | 0.23 | SNP |
| chr6_29855063_C_G | 29855063 | 1.28 | 1.06 | 1.56 | 0.011125 | 0.32 | 0.33 | 0.31 | SNP |
| chr6_30855211_T_G | 30855211 | 1.70 | 1.13 | 2.55 | 0.011131 | 0.12 | 0.13 | 0.11 | SNP |
| chr6_32634137_A_C | 32634137 | 1.33 | 1.07 | 1.65 | 0.011233 | 0.24 | 0.29 | 0.22 | SNP |
| chr6_32559216_G_A | 32559216 | 1.31 | 1.06 | 1.62 | 0.011263 | 0.38 | 0.38 | 0.38 | SNP |
| chr6_31914180_G_A | 31914180 | 0.64 | 0.45 | 0.90 | 0.011319 | 0.08 | 0.06 | 0.09 | SNP |
| chr6_32796793_G_A | 32796793 | 0.65 | 0.47 | 0.90 | 0.011368 | 0.08 | 0.06 | 0.09 | SNP |
| chr6_32627446_C_T | 32627446 | 1.67 | 1.11 | 2.46 | 0.011373 | 0.04 | 0.06 | 0.04 | SNP |
| chr6_31241002_A_G | 31241002 | 1.40 | 1.08 | 1.81 | 0.011384 | 0.10 | 0.12 | 0.10 | SNP |
| chr6_29692305_G_A | 29692305 | 1.32 | 1.06 | 1.64 | 0.011397 | 0.20 | 0.22 | 0.20 | SNP |
| chr6_31947086_G_A | 31947086 | 0.68 | 0.50 | 0.91 | 0.011415 | 0.11 | 0.08 | 0.12 | SNP |
| chr6_32635293_C_G | 32635293 | 1.33 | 1.07 | 1.66 | 0.011432 | 0.23 | 0.29 | 0.22 | SNP |
| chr6_31925921_G_A | 31925921 | 0.52 | 0.30 | 0.84 | 0.011448 | 0.04 | 0.02 | 0.04 | SNP |
| chr6_31929808_C_T | 31929808 | 1.80 | 1.13 | 2.82 | 0.011496 | 0.03 | 0.04 | 0.03 | SNP |
| chr6_32190217_T_C | 32190217 | 1.33 | 1.07 | 1.67 | 0.011512 | 0.27 | 0.30 | 0.26 | SNP |
| chr6_32610008_C_T | 32610008 | 1.34 | 1.07 | 1.67 | 0.011520 | 0.29 | 0.36 | 0.28 | SNP |
| chr6_32633959_T_C | 32633959 | 1.32 | 1.06 | 1.64 | 0.011525 | 0.24 | 0.29 | 0.23 | SNP |
| chr6_33546825_T_C | 33546825 | 1.25 | 1.05 | 1.49 | 0.011534 | 0.40 | 0.46 | 0.39 | SNP |
| chr6_29913099_G_A | 29913099 | 0.49 | 0.27 | 0.82 | 0.011570 | 0.03 | 0.02 | 0.04 | SNP |
| chr6_31110671_G_C | 31110671 | 0.77 | 0.62 | 0.94 | 0.011581 | 0.25 | 0.23 | 0.26 | SNP |
| chr6_31429927_T_C | 31429927 | 1.50 | 1.09 | 2.06 | 0.011631 | 0.17 | 0.18 | 0.16 | SNP |
| chr6_31935311_C_T | 31935311 | 0.67 | 0.49 | 0.91 | 0.011650 | 0.11 | 0.08 | 0.12 | SNP |
| chr6_31237087_C_A | 31237087 | 1.40 | 1.07 | 1.81 | 0.011657 | 0.10 | 0.12 | 0.10 | SNP |
| chr6_32633811_C_T | 32633811 | 1.62 | 1.10 | 2.34 | 0.011687 | 0.04 | 0.06 | 0.04 | SNP |
| chr6_32709204_G_C | 32709204 | 0.71 | 0.54 | 0.92 | 0.011741 | 0.21 | 0.18 | 0.22 | SNP |
| chr6_32629467_G_A | 32629467 | 1.33 | 1.07 | 1.67 | 0.011759 | 0.37 | 0.43 | 0.35 | SNP |
| chr6_32604924_C_T | 32604924 | 1.44 | 1.08 | 1.90 | 0.011887 | 0.08 | 0.10 | 0.07 | SNP |
| chr6_31113083_G_A | 31113083 | 1.36 | 1.07 | 1.73 | 0.011896 | 0.26 | 0.27 | 0.26 | SNP |
| chr6_31084288_T_C | 31084288 | 0.77 | 0.63 | 0.94 | 0.011911 | 0.48 | 0.45 | 0.49 | SNP |
| chr6_29692562_G_A | 29692562 | 1.32 | 1.06 | 1.63 | 0.011959 | 0.20 | 0.22 | 0.20 | SNP |
| chr6_29891825_T_C | 29891825 | 1.25 | 1.05 | 1.48 | 0.011963 | 0.48 | 0.50 | 0.48 | SNP |
| chr6_32603897_A_G | 32603897 | 1.44 | 1.08 | 1.91 | 0.011966 | 0.08 | 0.10 | 0.07 | SNP |
| chr6_32826450_T_C | 32826450 | 0.79 | 0.66 | 0.95 | 0.011985 | 0.45 | 0.42 | 0.46 | SNP |
| chr6_29854539_G_A | 29854539 | 1.30 | 1.06 | 1.60 | 0.012015 | 0.19 | 0.21 | 0.19 | SNP |
| chr6_32190484_G_A | 32190484 | 0.66 | 0.47 | 0.90 | 0.012019 | 0.09 | 0.06 | 0.09 | SNP |
| chr6_32634506_C_T | 32634506 | 1.32 | 1.06 | 1.64 | 0.012064 | 0.24 | 0.29 | 0.23 | SNP |
| chr6_32604660_C_T | 32604660 | 0.66 | 0.47 | 0.90 | 0.012075 | 0.09 | 0.06 | 0.10 | SNP |
| chr6_31240771_T_C | 31240771 | 1.40 | 1.07 | 1.81 | 0.012129 | 0.10 | 0.12 | 0.10 | SNP |

| | | | | | | | | | |
|---|---|---|---|---|---|---|---|---|---|
| chr6_31240932_C_A | 31240932 | 1.40 | 1.07 | 1.81 | 0.012129 | 0.10 | 0.12 | 0.10 | SNP |
| xHLA_2field_C_02_02 | 31236526 | 0.57 | 0.36 | 0.87 | 0.012242 | 0.06 | 0.03 | 0.06 | HLA (xHLA) |
| chr6_31324051_C_A | 31324051 | 0.63 | 0.43 | 0.90 | 0.012290 | 0.09 | 0.07 | 0.09 | SNP |
| chr6_32782387_G_A | 32782387 | 1.32 | 1.06 | 1.63 | 0.012310 | 0.21 | 0.22 | 0.21 | SNP |
| chr6_32634150_A_C | 32634150 | 1.32 | 1.06 | 1.64 | 0.012346 | 0.24 | 0.29 | 0.23 | SNP |
| chr6_32822296_C_G | 32822296 | 0.80 | 0.66 | 0.95 | 0.012435 | 0.43 | 0.39 | 0.44 | SNP |
| chr6_32609520_C_A | 32609520 | 1.44 | 1.08 | 1.92 | 0.012459 | 0.08 | 0.10 | 0.08 | SNP |
| chr6_29911154_C_A | 29911154 | 0.77 | 0.63 | 0.95 | 0.012557 | 0.49 | 0.47 | 0.50 | SNP |
| chr6_32634755_C_T | 32634755 | 1.32 | 1.06 | 1.64 | 0.012601 | 0.24 | 0.29 | 0.23 | SNP |
| chr6_31600106_C_A | 31600106 | 0.46 | 0.24 | 0.82 | 0.012621 | 0.03 | 0.02 | 0.03 | SNP |
| chr6_32606942_G_A | 32606942 | 1.33 | 1.06 | 1.66 | 0.012711 | 0.30 | 0.36 | 0.28 | SNP |
| chr6_32629531_A_G | 32629531 | 1.32 | 1.06 | 1.64 | 0.012720 | 0.24 | 0.29 | 0.23 | SNP |
| chr6_33169839_C_A | 33169839 | 1.39 | 1.07 | 1.80 | 0.012728 | 0.10 | 0.11 | 0.10 | SNP |
| chr6_33272626_C_T | 33272626 | 0.75 | 0.60 | 0.94 | 0.012777 | 0.18 | 0.15 | 0.18 | SNP |
| chr6_29712866_C_T | 29712866 | 1.39 | 1.07 | 1.79 | 0.012883 | 0.19 | 0.20 | 0.18 | SNP |
| chr6_32611229_T_G | 32611229 | 0.75 | 0.60 | 0.94 | 0.012899 | 0.46 | 0.41 | 0.48 | SNP |
| chr6_31113214_C_A | 31113214 | 0.77 | 0.62 | 0.94 | 0.012959 | 0.25 | 0.23 | 0.26 | SNP |
| chr6_32611418_A_C | 32611418 | 1.35 | 1.06 | 1.71 | 0.012974 | 0.27 | 0.33 | 0.26 | SNP |
| chr6_32635974_A_G | 32635974 | 1.32 | 1.06 | 1.64 | 0.012982 | 0.24 | 0.29 | 0.22 | SNP |
| xHLA_2field_DPB1_04_02 | 33043767 | 1.38 | 1.07 | 1.77 | 0.013032 | 0.11 | 0.12 | 0.11 | HLA (xHLA) |
| chr6_32634967_T_A | 32634967 | 1.32 | 1.06 | 1.64 | 0.013038 | 0.24 | 0.29 | 0.23 | SNP |
| chr6_32340593_G_C | 32340593 | 0.55 | 0.33 | 0.86 | 0.013049 | 0.04 | 0.03 | 0.05 | SNP |
| chr6_31465047_G_T | 31465047 | 1.87 | 1.14 | 3.05 | 0.013063 | 0.12 | 0.13 | 0.12 | SNP |
| chr6_32634545_C_A | 32634545 | 1.32 | 1.06 | 1.64 | 0.013095 | 0.24 | 0.29 | 0.22 | SNP |
| chr6_31795345_G_A | 31795345 | 0.52 | 0.30 | 0.85 | 0.013100 | 0.04 | 0.02 | 0.04 | SNP |
| chr6_31275511_T_A | 31275511 | 0.39 | 0.17 | 0.77 | 0.013159 | 0.02 | 0.01 | 0.03 | SNP |
| chr6_32374382_C_A | 32374382 | 0.58 | 0.36 | 0.88 | 0.013184 | 0.05 | 0.03 | 0.05 | SNP |
| chr6_32374437_A_G | 32374437 | 0.58 | 0.36 | 0.88 | 0.013184 | 0.05 | 0.03 | 0.05 | SNP |
| chr6_32374640_G_A | 32374640 | 0.58 | 0.36 | 0.88 | 0.013184 | 0.05 | 0.03 | 0.05 | SNP |
| chr6_32606732_G_A | 32606732 | 1.32 | 1.06 | 1.66 | 0.013337 | 0.30 | 0.36 | 0.28 | SNP |
| chr6_31514247_A_G | 31514247 | 1.88 | 1.14 | 3.10 | 0.013362 | 0.12 | 0.13 | 0.12 | SNP |
| chr6_32812858_A_G | 32812858 | 0.74 | 0.58 | 0.94 | 0.013411 | 0.15 | 0.13 | 0.16 | SNP |
| chr6_31239577_C_A | 31239577 | 1.36 | 1.06 | 1.73 | 0.013552 | 0.48 | 0.51 | 0.47 | SNP |
| chr6_32007625_C_T | 32007625 | 0.67 | 0.49 | 0.92 | 0.013639 | 0.10 | 0.07 | 0.10 | SNP |
| chr6_32604151_C_T | 32604151 | 1.33 | 1.06 | 1.66 | 0.013657 | 0.30 | 0.36 | 0.28 | SNP |
| chr6_31974328_G_A | 31974328 | 1.56 | 1.09 | 2.22 | 0.013707 | 0.05 | 0.07 | 0.05 | SNP |
| chr6_32629549_G_A | 32629549 | 1.32 | 1.06 | 1.64 | 0.013721 | 0.24 | 0.29 | 0.23 | SNP |
| chr6_32707585_G_A | 32707585 | 0.71 | 0.54 | 0.93 | 0.013722 | 0.21 | 0.18 | 0.22 | SNP |
| chr6_31275691_A_C | 31275691 | 0.62 | 0.42 | 0.90 | 0.013730 | 0.07 | 0.05 | 0.07 | SNP |
| chr6_32012755_G_A | 32012755 | 0.63 | 0.43 | 0.90 | 0.013745 | 0.08 | 0.06 | 0.09 | SNP |
| chr6_32188713_C_G | 32188713 | 0.53 | 0.31 | 0.86 | 0.013819 | 0.04 | 0.02 | 0.04 | SNP |
| chr6_32017524_A_G | 32017524 | 0.50 | 0.28 | 0.84 | 0.013917 | 0.04 | 0.02 | 0.04 | SNP |
| chr6_32628617_A_C | 32628617 | 1.32 | 1.06 | 1.66 | 0.013962 | 0.23 | 0.28 | 0.22 | SNP |
| chr6_32628283_G_A | 32628283 | 1.34 | 1.06 | 1.70 | 0.014053 | 0.22 | 0.28 | 0.21 | SNP |
| chr6_32077690_G_A | 32077690 | 0.53 | 0.31 | 0.86 | 0.014076 | 0.04 | 0.02 | 0.04 | SNP |
| chr6_32818430_C_G | 32818430 | 0.56 | 0.35 | 0.87 | 0.014142 | 0.04 | 0.03 | 0.05 | SNP |
| chr6_30229267_G_C | 30229267 | 1.59 | 1.10 | 2.29 | 0.014205 | 0.10 | 0.12 | 0.10 | SNP |
| chr6_29897018_G_A | 29897018 | 1.33 | 1.06 | 1.68 | 0.014232 | 0.18 | 0.20 | 0.18 | SNP |
| chr6_33553580_T_C | 33553580 | 1.27 | 1.05 | 1.54 | 0.014238 | 0.49 | 0.53 | 0.48 | SNP |
| chr6_31919578_G_A | 31919578 | 0.75 | 0.60 | 0.94 | 0.014248 | 0.48 | 0.44 | 0.49 | SNP |
| chr6_29909440_A_G | 29909440 | 0.68 | 0.49 | 0.92 | 0.014263 | 0.09 | 0.07 | 0.10 | SNP |
| chr6_32036357_G_A | 32036357 | 0.67 | 0.48 | 0.92 | 0.014267 | 0.09 | 0.07 | 0.10 | SNP |
| chr6_29635928_C_T | 29635928 | 1.31 | 1.05 | 1.62 | 0.014293 | 0.22 | 0.23 | 0.21 | SNP |
| chr6_32605336_G_A | 32605336 | 1.32 | 1.06 | 1.65 | 0.014295 | 0.30 | 0.36 | 0.28 | SNP |
| chr6_32550587_T_G | 32550587 | 0.57 | 0.35 | 0.88 | 0.014412 | 0.05 | 0.03 | 0.05 | SNP |
| chr6_29941597_G_A | 29941597 | 1.35 | 1.06 | 1.71 | 0.014516 | 0.14 | 0.17 | 0.14 | SNP |
| chr6_32609380_C_T | 32609380 | 1.37 | 1.06 | 1.77 | 0.014574 | 0.35 | 0.42 | 0.34 | SNP |
| chr6_29909454_T_C | 29909454 | 0.68 | 0.49 | 0.92 | 0.014628 | 0.09 | 0.07 | 0.10 | SNP |
| chr6_32817008_C_T | 32817008 | 0.53 | 0.31 | 0.86 | 0.014668 | 0.04 | 0.02 | 0.04 | SNP |
| chr6_32610403_C_A | 32610403 | 1.33 | 1.06 | 1.67 | 0.014706 | 0.29 | 0.35 | 0.28 | SNP |
| chr6_32605418_G_A | 32605418 | 1.32 | 1.06 | 1.65 | 0.014805 | 0.30 | 0.36 | 0.28 | SNP |
| chr6_32633816_C_G | 32633816 | 1.28 | 1.05 | 1.56 | 0.014830 | 0.37 | 0.40 | 0.36 | SNP |
| chr6_32825348_T_C | 32825348 | 0.80 | 0.67 | 0.96 | 0.014889 | 0.43 | 0.40 | 0.44 | SNP |
| chr6_32814244_A_T | 32814244 | 0.71 | 0.53 | 0.93 | 0.014914 | 0.10 | 0.08 | 0.11 | SNP |
| chr6_32605101_A_G | 32605101 | 1.32 | 1.06 | 1.65 | 0.014959 | 0.30 | 0.36 | 0.28 | SNP |
| chr6_31236853_A_G | 31236853 | 1.52 | 1.08 | 2.12 | 0.014974 | 0.18 | 0.20 | 0.17 | SNP |
| chr6_32017522_G_A | 32017522 | 0.50 | 0.28 | 0.85 | 0.014981 | 0.04 | 0.02 | 0.04 | SNP |
| chr6_32628420_C_T | 32628420 | 1.33 | 1.06 | 1.66 | 0.015035 | 0.36 | 0.42 | 0.34 | SNP |
| chr6_32608350_G_A | 32608350 | 0.65 | 0.45 | 0.91 | 0.015144 | 0.10 | 0.07 | 0.10 | SNP |
| chr6_32375347_T_G | 32375347 | 0.55 | 0.33 | 0.87 | 0.015392 | 0.04 | 0.03 | 0.04 | SNP |
| xHLA_2field_DRB1_04_04 | 32546552 | 0.60 | 0.39 | 0.90 | 0.015495 | 0.06 | 0.04 | 0.06 | HLA (xHLA) |
| chr6_31837491_G_A | 31837491 | 1.35 | 1.06 | 1.72 | 0.015565 | 0.49 | 0.50 | 0.49 | SNP |
| chr6_33547287_C_G | 33547287 | 0.49 | 0.26 | 0.84 | 0.015589 | 0.03 | 0.02 | 0.03 | SNP |
| chr6_32498205_G_T | 32498205 | 1.32 | 1.05 | 1.66 | 0.015702 | 0.30 | 0.37 | 0.28 | SNP |
| chr6_32005649_A_G | 32005649 | 0.70 | 0.53 | 0.93 | 0.015705 | 0.11 | 0.08 | 0.12 | SNP |
| chr6_31906865_C_T | 31906865 | 0.66 | 0.47 | 0.92 | 0.015791 | 0.08 | 0.06 | 0.09 | SNP |
| chr6_32052444_C_T | 32052444 | 0.54 | 0.32 | 0.87 | 0.015805 | 0.04 | 0.02 | 0.04 | SNP |
| chr6_32605578_G_A | 32605578 | 1.30 | 1.05 | 1.62 | 0.015821 | 0.33 | 0.35 | 0.33 | SNP |
| chr6_31905130_G_A | 31905130 | 0.66 | 0.47 | 0.92 | 0.015909 | 0.08 | 0.06 | 0.09 | SNP |
| chr6_31905328_G_T | 31905328 | 0.66 | 0.47 | 0.92 | 0.015909 | 0.08 | 0.06 | 0.09 | SNP |
| chr6_31907147_T_C | 31907147 | 0.66 | 0.47 | 0.92 | 0.015909 | 0.08 | 0.06 | 0.09 | SNP |
| chr6_31907168_C_T | 31907168 | 0.66 | 0.47 | 0.92 | 0.015909 | 0.08 | 0.06 | 0.09 | SNP |
| chr6_31248432_A_G | 31248432 | 0.77 | 0.62 | 0.95 | 0.015942 | 0.47 | 0.46 | 0.47 | SNP |
| chr6_31465917_G_T | 31465917 | 1.74 | 1.10 | 2.72 | 0.015943 | 0.03 | 0.04 | 0.03 | SNP |
| chr6_32629764_C_T | 32629764 | 1.31 | 1.05 | 1.64 | 0.016040 | 0.37 | 0.43 | 0.36 | SNP |
| chr6_31430010_G_A | 31430010 | 1.83 | 1.12 | 2.98 | 0.016096 | 0.12 | 0.13 | 0.12 | SNP |
| chr6_30037990_T_C | 30037990 | 1.35 | 1.05 | 1.71 | 0.016099 | 0.15 | 0.17 | 0.14 | SNP |
| chr6_32611427_T_A | 32611427 | 1.33 | 1.05 | 1.69 | 0.016119 | 0.27 | 0.33 | 0.26 | SNP |
| chr6_29689164_T_A | 29689164 | 1.30 | 1.05 | 1.61 | 0.016145 | 0.20 | 0.22 | 0.20 | SNP |
| chr6_32005048_C_G | 32005048 | 0.54 | 0.32 | 0.87 | 0.016183 | 0.04 | 0.02 | 0.04 | SNP |
| chr6_32816998_T_C | 32816998 | 0.76 | 0.61 | 0.95 | 0.016214 | 0.18 | 0.15 | 0.19 | SNP |
| chr6_32052983_C_T | 32052983 | 0.68 | 0.49 | 0.92 | 0.016239 | 0.09 | 0.07 | 0.10 | SNP |
| chr6_31243971_G_A | 31243971 | 1.41 | 1.06 | 1.86 | 0.016296 | 0.32 | 0.34 | 0.32 | SNP |
| chr6_32369601_T_C | 32369601 | 0.55 | 0.33 | 0.88 | 0.016414 | 0.04 | 0.03 | 0.04 | SNP |
| chr6_31248819_C_T | 31248819 | 1.42 | 1.06 | 1.88 | 0.016510 | 0.10 | 0.12 | 0.10 | SNP |
| chr6_32610860_G_T | 32610860 | 1.32 | 1.05 | 1.66 | 0.016533 | 0.29 | 0.34 | 0.27 | SNP |
| chr6_29692634_G_T | 29692634 | 1.30 | 1.05 | 1.61 | 0.016739 | 0.20 | 0.22 | 0.20 | SNP |
| chr6_32080383_C_T | 32080383 | 0.66 | 0.46 | 0.92 | 0.016787 | 0.08 | 0.06 | 0.09 | SNP |
| chr6_31244021_A_G | 31244021 | 1.40 | 1.06 | 1.86 | 0.017008 | 0.32 | 0.33 | 0.31 | SNP |
| chr6_32813279_C_A | 32813279 | 0.71 | 0.53 | 0.93 | 0.017080 | 0.10 | 0.08 | 0.11 | SNP |
| chr6_32006791_G_A | 32006791 | 0.54 | 0.32 | 0.88 | 0.017125 | 0.04 | 0.02 | 0.04 | SNP |
| chr6_32304466_T_A | 32304466 | 0.56 | 0.33 | 0.88 | 0.017134 | 0.04 | 0.03 | 0.05 | SNP |
| chr6_31383848_G_A | 31383848 | 0.68 | 0.49 | 0.93 | 0.017302 | 0.09 | 0.07 | 0.09 | SNP |
| chr6_31508069_T_G | 31508069 | 0.37 | 0.15 | 0.79 | 0.017352 | 0.02 | 0.01 | 0.02 | SNP |
| chr6_32498206_A_T | 32498206 | 1.32 | 1.05 | 1.66 | 0.017430 | 0.30 | 0.37 | 0.28 | SNP |
| chr6_31473746_G_A | 31473746 | 1.83 | 1.11 | 3.02 | 0.017595 | 0.12 | 0.13 | 0.12 | SNP |
| chr6_31322790_T_A | 31322790 | 1.74 | 1.10 | 2.76 | 0.017756 | 0.12 | 0.13 | 0.12 | SNP |
| chr6_32605419_G_C | 32605419 | 1.31 | 1.05 | 1.64 | 0.017837 | 0.30 | 0.36 | 0.28 | SNP |
| chr6_29864562_G_A | 29864562 | 1.27 | 1.04 | 1.56 | 0.017909 | 0.28 | 0.30 | 0.28 | SNP |
| chr6_32707040_G_A | 32707040 | 0.71 | 0.53 | 0.94 | 0.017966 | 0.21 | 0.17 | 0.22 | SNP |
| chr6_32609286_T_C | 32609286 | 0.77 | 0.62 | 0.96 | 0.017999 | 0.49 | 0.54 | 0.47 | SNP |
| chr6_29910721_G_C | 29910721 | 0.66 | 0.47 | 0.92 | 0.018092 | 0.11 | 0.08 | 0.11 | SNP |
| chr6_32376882_T_G | 32376882 | 1.30 | 1.05 | 1.62 | 0.018153 | 0.48 | 0.52 | 0.47 | SNP |
| chr6_31544189_A_G | 31544189 | 1.58 | 1.07 | 2.30 | 0.018206 | 0.04 | 0.05 | 0.04 | SNP |
| xHLA_2field_B_07_02 | 31321652 | 1.52 | 1.07 | 2.15 | 0.018229 | 0.17 | 0.19 | 0.17 | HLA (xHLA) |
| chr6_31321238_G_C | 31321238 | 1.59 | 1.07 | 2.32 | 0.018244 | 0.04 | 0.05 | 0.04 | SNP |
| chr6_32634261_C_T | 32634261 | 1.30 | 1.05 | 1.62 | 0.018286 | 0.24 | 0.28 | 0.23 | SNP |
| chr6_32818774_T_C | 32818774 | 0.77 | 0.62 | 0.95 | 0.018474 | 0.18 | 0.15 | 0.18 | SNP |
| chr6_33290872_G_A | 33290872 | 1.24 | 1.04 | 1.49 | 0.018562 | 0.35 | 0.40 | 0.34 | SNP |
| chr6_32634262_T_G | 32634262 | 1.30 | 1.04 | 1.62 | 0.018572 | 0.24 | 0.28 | 0.23 | SNP |
| chr6_32636364_G_A | 32636364 | 1.26 | 1.04 | 1.54 | 0.018676 | 0.37 | 0.40 | 0.37 | SNP |
| chr6_32606052_T_G | 32606052 | 1.31 | 1.05 | 1.64 | 0.018694 | 0.30 | 0.36 | 0.29 | SNP |
| chr6_32633287_C_T | 32633287 | 1.31 | 1.05 | 1.64 | 0.018730 | 0.31 | 0.37 | 0.30 | SNP |
| chr6_32375745_G_A | 32375745 | 1.30 | 1.05 | 1.61 | 0.018757 | 0.48 | 0.52 | 0.47 | SNP |
| chr6_32376618_C_T | 32376618 | 1.30 | 1.05 | 1.61 | 0.018757 | 0.48 | 0.52 | 0.47 | SNP |
| chr6_31895973_C_G | 31895973 | 1.35 | 1.05 | 1.72 | 0.018768 | 0.13 | 0.14 | 0.13 | SNP |
| chr6_31851354_G_A | 31851354 | 0.54 | 0.32 | 0.88 | 0.018832 | 0.05 | 0.03 | 0.05 | SNP |
| chr6_32707023_T_C | 32707023 | 0.71 | 0.53 | 0.94 | 0.018936 | 0.21 | 0.17 | 0.22 | SNP |
| chr6_32628965_A_G | 32628965 | 1.35 | 1.05 | 1.74 | 0.019043 | 0.21 | 0.23 | 0.21 | SNP |
| chr6_32323905_C_A | 32323905 | 0.56 | 0.34 | 0.89 | 0.019080 | 0.04 | 0.03 | 0.05 | SNP |
| chr6_29897102_A_G | 29897102 | 1.29 | 1.04 | 1.59 | 0.019086 | 0.20 | 0.22 | 0.20 | SNP |
| chr6_31247067_G_T | 31247067 | 1.54 | 1.07 | 2.21 | 0.019121 | 0.17 | 0.19 | 0.17 | SNP |
| chr6_29892815_A_G | 29892815 | 1.39 | 1.05 | 1.82 | 0.019135 | 0.15 | 0.17 | 0.14 | SNP |
| chr6_32085317_C_G | 32085317 | 0.54 | 0.32 | 0.88 | 0.019169 | 0.04 | 0.02 | 0.04 | SNP |
| chr6_32077380_C_T | 32077380 | 0.67 | 0.47 | 0.93 | 0.019197 | 0.08 | 0.06 | 0.09 | SNP |
| chr6_32823186_T_G | 32823186 | 0.54 | 0.31 | 0.88 | 0.019199 | 0.04 | 0.02 | 0.04 | SNP |
| chr6_32362416_G_T | 32362416 | 1.36 | 1.05 | 1.76 | 0.019273 | 0.24 | 0.26 | 0.23 | SNP |
| chr6_32412395_A_C | 32412395 | 1.31 | 1.04 | 1.64 | 0.019293 | 0.25 | 0.27 | 0.24 | SNP |
| chr6_32710692_G_T | 32710692 | 1.25 | 1.04 | 1.50 | 0.019316 | 0.38 | 0.40 | 0.37 | SNP |
| chr6_29925636_T_G | 29925636 | 1.35 | 1.05 | 1.74 | 0.019446 | 0.14 | 0.16 | 0.13 | SNP |
| chr6_31236998_T_C | 31236998 | 1.50 | 1.07 | 2.10 | 0.019457 | 0.18 | 0.20 | 0.17 | SNP |
| chr6_31118511_T_G | 31118511 | 0.78 | 0.63 | 0.96 | 0.019487 | 0.25 | 0.23 | 0.25 | SNP |
| chr6_32636172_C_T | 32636172 | 1.30 | 1.04 | 1.62 | 0.019512 | 0.23 | 0.28 | 0.22 | SNP |
| chr6_32803408_T_A | 32803408 | 1.74 | 1.08 | 2.75 | 0.019523 | 0.03 | 0.04 | 0.02 | SNP |
| chr6_32710466_G_T | 32710466 | 1.25 | 1.04 | 1.51 | 0.019542 | 0.37 | 0.39 | 0.36 | SNP |
| chr6_31244300_A_G | 31244300 | 1.51 | 1.07 | 2.14 | 0.019565 | 0.18 | 0.20 | 0.17 | SNP |
| chr6_32376932_C_T | 32376932 | 1.30 | 1.04 | 1.61 | 0.019589 | 0.48 | 0.52 | 0.47 | SNP |
| chr6_31245620_T_C | 31245620 | 1.49 | 1.07 | 2.09 | 0.019682 | 0.18 | 0.20 | 0.17 | SNP |
| chr6_32612410_T_C | 32612410 | 0.75 | 0.59 | 0.95 | 0.019695 | 0.45 | 0.38 | 0.46 | SNP |
| chr6_32711050_C_T | 32711050 | 0.72 | 0.55 | 0.95 | 0.019709 | 0.21 | 0.18 | 0.21 | SNP |
| chr6_32821447_C_T | 32821447 | 1.39 | 1.05 | 1.83 | 0.019784 | 0.13 | 0.18 | 0.12 | SNP |
| chr6_32522703_T_G | 32522703 | 0.77 | 0.62 | 0.96 | 0.019785 | 0.40 | 0.37 | 0.41 | SNP |
| chr6_31243979_A_T | 31243979 | 1.39 | 1.05 | 1.83 | 0.019813 | 0.32 | 0.34 | 0.32 | SNP |
| chr6_31464091_G_A | 31464091 | 1.71 | 1.08 | 2.67 | 0.019864 | 0.03 | 0.04 | 0.03 | SNP |
| chr6_31474820_C_T | 31474820 | 1.71 | 1.08 | 2.67 | 0.019864 | 0.03 | 0.04 | 0.03 | SNP |

| | | | | | | | | | |
|---|---|---|---|---|---|---|---|---|---|
| chr6_29720604_G_A | 29720604 | 1.35 | 1.05 | 1.75 | 0.019866 | 0.20 | 0.21 | 0.20 | SNP |
| chr6_31275560_T_C | 31275560 | 0.64 | 0.43 | 0.92 | 0.019906 | 0.07 | 0.05 | 0.07 | SNP |
| chr6_31778272_A_G | 31778272 | 1.40 | 1.05 | 1.86 | 0.019910 | 0.22 | 0.23 | 0.21 | SNP |
| chr6_31241092_G_C | 31241092 | 1.30 | 1.04 | 1.61 | 0.019992 | 0.38 | 0.40 | 0.38 | SNP |
| chr6_30030114_G_A | 30030114 | 1.33 | 1.04 | 1.69 | 0.020007 | 0.15 | 0.17 | 0.14 | SNP |
| chr6_32490766_G_T | 32490766 | 1.86 | 1.09 | 3.12 | 0.020174 | 0.02 | 0.03 | 0.02 | SNP |
| chr6_29895913_G_A | 29895913 | 0.81 | 0.68 | 0.97 | 0.020272 | 0.44 | 0.43 | 0.45 | SNP |
| chr6_32183175_G_A | 32183175 | 1.25 | 1.03 | 1.50 | 0.020310 | 0.39 | 0.43 | 0.38 | SNP |
| chr6_32785714_T_C | 32785714 | 1.29 | 1.04 | 1.60 | 0.020329 | 0.21 | 0.22 | 0.21 | SNP |
| chr6_30796545_T_C | 30796545 | 1.56 | 1.07 | 2.28 | 0.020356 | 0.12 | 0.13 | 0.12 | SNP |
| chr6_31948400_T_G | 31948400 | 0.55 | 0.33 | 0.89 | 0.020511 | 0.04 | 0.02 | 0.04 | SNP |
| chr6_31935567_G_A | 31935567 | 0.58 | 0.36 | 0.90 | 0.020754 | 0.04 | 0.03 | 0.05 | SNP |
| chr6_32707551_A_G | 32707551 | 1.24 | 1.03 | 1.49 | 0.020767 | 0.38 | 0.41 | 0.38 | SNP |
| chr6_32628845_G_A | 32628845 | 1.31 | 1.04 | 1.64 | 0.020859 | 0.36 | 0.42 | 0.35 | SNP |
| chr6_31127037_C_T | 31127037 | 0.60 | 0.38 | 0.91 | 0.020902 | 0.05 | 0.03 | 0.06 | SNP |
| chr6_32610314_T_C | 32610314 | 1.29 | 1.04 | 1.60 | 0.020938 | 0.33 | 0.35 | 0.32 | SNP |
| chr6_32707558_G_A | 32707558 | 1.24 | 1.03 | 1.49 | 0.020944 | 0.38 | 0.41 | 0.38 | SNP |
| chr6_29768705_C_A | 29768705 | 1.29 | 1.04 | 1.59 | 0.020944 | 0.25 | 0.26 | 0.25 | SNP |
| chr6_32006337_A_C | 32006337 | 0.74 | 0.58 | 0.95 | 0.020957 | 0.33 | 0.30 | 0.34 | SNP |
| chr6_32187722_T_C | 32187722 | 0.79 | 0.64 | 0.96 | 0.021023 | 0.45 | 0.40 | 0.46 | SNP |
| chr6_32377061_C_T | 32377061 | 1.30 | 1.04 | 1.62 | 0.021162 | 0.48 | 0.52 | 0.47 | SNP |
| chr6_32707565_C_T | 32707565 | 1.24 | 1.03 | 1.48 | 0.021224 | 0.38 | 0.41 | 0.38 | SNP |
| chr6_32707566_A_G | 32707566 | 1.24 | 1.03 | 1.48 | 0.021224 | 0.38 | 0.41 | 0.38 | SNP |
| chr6_31629995_G_A | 31629995 | 0.64 | 0.43 | 0.92 | 0.021263 | 0.06 | 0.04 | 0.07 | SNP |
| chr6_32521591_C_T | 32521591 | 1.31 | 1.04 | 1.64 | 0.021306 | 0.32 | 0.38 | 0.30 | SNP |
| chr6_31239896_T_C | 31239896 | 0.76 | 0.60 | 0.96 | 0.021622 | 0.21 | 0.21 | 0.21 | SNP |
| chr6_32636385_A_T | 32636385 | 1.26 | 1.03 | 1.53 | 0.021788 | 0.37 | 0.40 | 0.36 | SNP |
| chr6_32629328_G_A | 32629328 | 1.30 | 1.04 | 1.64 | 0.021864 | 0.23 | 0.28 | 0.22 | SNP |
| xHLA_2field_C_07_04 | 31236526 | 0.41 | 0.18 | 0.83 | 0.021876 | 0.02 | 0.01 | 0.02 | HLA (xHLA) |
| chr6_32559702_A_G | 32559702 | 1.39 | 1.05 | 1.84 | 0.021929 | 0.21 | 0.27 | 0.19 | SNP |
| xHLA_2field_DRB1_13_01 | 32546552 | 0.67 | 0.47 | 0.93 | 0.022054 | 0.08 | 0.06 | 0.09 | HLA (xHLA) |
| chr6_32608856_T_C | 32608856 | 1.31 | 1.04 | 1.65 | 0.022504 | 0.28 | 0.34 | 0.27 | SNP |
| chr6_32797466_G_A | 32797466 | 1.45 | 1.05 | 2.00 | 0.022543 | 0.08 | 0.11 | 0.07 | SNP |
| chr6_29795720_G_A | 29795720 | 1.35 | 1.04 | 1.74 | 0.022588 | 0.19 | 0.20 | 0.18 | SNP |
| chr6_29693011_T_C | 29693011 | 1.29 | 1.03 | 1.60 | 0.022887 | 0.20 | 0.21 | 0.20 | SNP |
| chr6_32628633_T_C | 32628633 | 1.29 | 1.04 | 1.62 | 0.022932 | 0.23 | 0.28 | 0.22 | SNP |
| chr6_29893403_C_G | 29893403 | 1.37 | 1.04 | 1.78 | 0.022954 | 0.15 | 0.17 | 0.15 | SNP |
| chr6_32627652_C_G | 32627652 | 1.30 | 1.04 | 1.62 | 0.023014 | 0.37 | 0.43 | 0.36 | SNP |
| chr6_29913668_G_T | 29913668 | 1.28 | 1.03 | 1.59 | 0.023109 | 0.23 | 0.24 | 0.22 | SNP |
| chr6_32636138_A_G | 32636138 | 1.29 | 1.03 | 1.61 | 0.023494 | 0.23 | 0.28 | 0.22 | SNP |
| chr6_29897736_C_T | 29897736 | 1.28 | 1.03 | 1.58 | 0.023590 | 0.21 | 0.22 | 0.20 | SNP |
| chr6_31324531_T_C | 31324531 | 1.35 | 1.04 | 1.75 | 0.023656 | 0.31 | 0.33 | 0.31 | SNP |
| chr6_32334258_G_T | 32334258 | 0.77 | 0.61 | 0.96 | 0.023657 | 0.40 | 0.40 | 0.40 | SNP |
| chr6_32411726_A_G | 32411726 | 1.30 | 1.03 | 1.63 | 0.023686 | 0.25 | 0.27 | 0.24 | SNP |
| chr6_29944314_C_T | 29944314 | 1.23 | 1.03 | 1.47 | 0.023797 | 0.46 | 0.47 | 0.46 | SNP |
| chr6_32629535_C_A | 32629535 | 1.29 | 1.03 | 1.62 | 0.023889 | 0.37 | 0.43 | 0.35 | SNP |
| chr6_31558702_C_T | 31558702 | 1.79 | 1.08 | 2.96 | 0.023902 | 0.12 | 0.13 | 0.12 | SNP |
| chr6_33280505_C_A | 33280505 | 1.21 | 1.03 | 1.43 | 0.024091 | 0.35 | 0.40 | 0.34 | SNP |
| chr6_32629400_A_G | 32629400 | 1.29 | 1.03 | 1.62 | 0.024102 | 0.37 | 0.43 | 0.35 | SNP |
| chr6_32608809_T_C | 32608809 | 1.31 | 1.04 | 1.65 | 0.024103 | 0.27 | 0.33 | 0.25 | SNP |
| chr6_29893235_C_T | 29893235 | 0.82 | 0.69 | 0.97 | 0.024105 | 0.45 | 0.44 | 0.45 | SNP |
| chr6_29895944_G_A | 29895944 | 0.82 | 0.68 | 0.97 | 0.024147 | 0.45 | 0.44 | 0.45 | SNP |
| chr6_32610994_C_A | 32610994 | 1.31 | 1.04 | 1.66 | 0.024152 | 0.26 | 0.31 | 0.25 | SNP |
| chr6_32609456_C_T | 32609456 | 1.30 | 1.03 | 1.63 | 0.024178 | 0.29 | 0.35 | 0.28 | SNP |
| chr6_32555432_G_A | 32555432 | 1.42 | 1.04 | 1.91 | 0.024297 | 0.08 | 0.09 | 0.07 | SNP |
| chr6_32713867_C_T | 32713867 | 0.73 | 0.55 | 0.96 | 0.024306 | 0.20 | 0.17 | 0.20 | SNP |
| chr6_31085382_C_A | 31085382 | 0.81 | 0.67 | 0.97 | 0.024408 | 0.48 | 0.46 | 0.49 | SNP |
| chr6_33257162_C_T | 33257162 | 1.32 | 1.03 | 1.69 | 0.024408 | 0.13 | 0.16 | 0.13 | SNP |
| chr6_29893195_G_A | 29893195 | 1.22 | 1.03 | 1.45 | 0.024428 | 0.48 | 0.49 | 0.47 | SNP |
| chr6_32814659_A_G | 32814659 | 0.77 | 0.61 | 0.96 | 0.024453 | 0.16 | 0.13 | 0.16 | SNP |
| chr6_32498208_G_T | 32498208 | 1.30 | 1.03 | 1.64 | 0.024508 | 0.30 | 0.37 | 0.28 | SNP |
| chr6_31601344_C_T | 31601344 | 0.73 | 0.55 | 0.96 | 0.024515 | 0.29 | 0.26 | 0.30 | SNP |
| chr6_32806576_C_T | 32806576 | 1.26 | 1.03 | 1.55 | 0.024581 | 0.42 | 0.48 | 0.41 | SNP |
| chr6_32629347_T_C | 32629347 | 1.30 | 1.03 | 1.63 | 0.024583 | 0.36 | 0.42 | 0.35 | SNP |
| chr6_32709965_G_T | 32709965 | 0.73 | 0.55 | 0.96 | 0.024668 | 0.20 | 0.17 | 0.21 | SNP |
| chr6_29857410_C_T | 29857410 | 1.23 | 1.03 | 1.47 | 0.024811 | 0.29 | 0.30 | 0.29 | SNP |
| chr6_32410987_G_A | 32410987 | 1.46 | 1.05 | 2.02 | 0.025100 | 0.17 | 0.19 | 0.17 | SNP |
| chr6_32707806_T_C | 32707806 | 1.23 | 1.03 | 1.47 | 0.025269 | 0.38 | 0.41 | 0.38 | SNP |
| chr6_32611860_G_A | 32611860 | 1.43 | 1.04 | 1.96 | 0.025381 | 0.18 | 0.20 | 0.18 | SNP |
| chr6_31240353_C_A | 31240353 | 1.47 | 1.05 | 2.05 | 0.025392 | 0.18 | 0.20 | 0.17 | SNP |
| chr6_31240663_A_T | 31240663 | 1.47 | 1.05 | 2.05 | 0.025392 | 0.18 | 0.20 | 0.17 | SNP |
| chr6_32131667_G_A | 32131667 | 0.56 | 0.33 | 0.91 | 0.025430 | 0.04 | 0.02 | 0.04 | SNP |
| chr6_32491581_A_C | 32491581 | 1.33 | 1.03 | 1.70 | 0.025441 | 0.26 | 0.32 | 0.25 | SNP |
| chr6_31275657_T_C | 31275657 | 0.44 | 0.20 | 0.86 | 0.025629 | 0.02 | 0.01 | 0.03 | SNP |
| chr6_32975014_T_C | 32975014 | 1.22 | 1.02 | 1.45 | 0.025701 | 0.48 | 0.53 | 0.47 | SNP |
| chr6_29895945_A_T | 29895945 | 2.05 | 1.06 | 3.79 | 0.025741 | 0.01 | 0.02 | 0.01 | SNP |
| chr6_29854082_T_A | 29854082 | 1.24 | 1.03 | 1.50 | 0.025764 | 0.24 | 0.25 | 0.24 | SNP |
| chr6_32636443_A_C | 32636443 | 1.29 | 1.03 | 1.61 | 0.026152 | 0.23 | 0.28 | 0.22 | SNP |
| chr6_33627367_A_G | 33627367 | 0.58 | 0.36 | 0.92 | 0.026326 | 0.05 | 0.03 | 0.05 | SNP |
| chr6_32707282_A_G | 32707282 | 1.23 | 1.02 | 1.47 | 0.026355 | 0.38 | 0.41 | 0.38 | SNP |
| chr6_31112108_G_T | 31112108 | 1.32 | 1.03 | 1.68 | 0.026372 | 0.27 | 0.27 | 0.27 | SNP |
| chr6_31239681_T_A | 31239681 | 1.34 | 1.03 | 1.74 | 0.026382 | 0.41 | 0.44 | 0.40 | SNP |
| chr6_32006317_T_C | 32006317 | 0.71 | 0.53 | 0.96 | 0.026441 | 0.26 | 0.23 | 0.27 | SNP |
| chr6_32610997_C_G | 32610997 | 1.30 | 1.03 | 1.65 | 0.026466 | 0.26 | 0.31 | 0.25 | SNP |
| chr6_32636154_C_G | 32636154 | 1.28 | 1.03 | 1.60 | 0.026586 | 0.23 | 0.28 | 0.22 | SNP |
| chr6_31325056_C_T | 31325056 | 1.47 | 1.04 | 2.05 | 0.026587 | 0.15 | 0.16 | 0.15 | SNP |
| chr6_32193945_G_T | 32193945 | 0.77 | 0.61 | 0.97 | 0.026662 | 0.16 | 0.14 | 0.17 | SNP |
| chr6_31702710_C_T | 31702710 | 0.65 | 0.44 | 0.94 | 0.026785 | 0.06 | 0.04 | 0.06 | SNP |
| chr6_32636063_G_A | 32636063 | 1.28 | 1.03 | 1.59 | 0.026977 | 0.24 | 0.29 | 0.22 | SNP |
| chr6_32806673_T_C | 32806673 | 0.58 | 0.35 | 0.92 | 0.027296 | 0.04 | 0.02 | 0.04 | SNP |
| chr6_32709346_T_C | 32709346 | 1.23 | 1.02 | 1.47 | 0.027411 | 0.38 | 0.41 | 0.38 | SNP |
| chr6_30228416_T_C | 30228416 | 0.76 | 0.59 | 0.97 | 0.027434 | 0.20 | 0.15 | 0.21 | SNP |
| chr6_32731313_T_A | 32731313 | 0.74 | 0.56 | 0.96 | 0.027635 | 0.20 | 0.17 | 0.21 | SNP |
| chr6_30515043_T_C | 30515043 | 1.51 | 1.04 | 2.17 | 0.027702 | 0.11 | 0.12 | 0.11 | SNP |
| chr6_31906828_G_A | 31906828 | 0.58 | 0.35 | 0.92 | 0.027861 | 0.04 | 0.03 | 0.04 | SNP |
| chr6_32500941_C_A | 32500941 | 1.41 | 1.04 | 1.91 | 0.027915 | 0.18 | 0.20 | 0.18 | SNP |
| chr6_31241127_T_C | 31241127 | 1.35 | 1.03 | 1.76 | 0.027978 | 0.10 | 0.11 | 0.09 | SNP |
| chr6_32825507_C_T | 32825507 | 0.81 | 0.66 | 0.98 | 0.028002 | 0.36 | 0.32 | 0.37 | SNP |
| chr6_32029183_C_T | 32029183 | 0.70 | 0.51 | 0.96 | 0.028110 | 0.09 | 0.07 | 0.10 | SNP |
| chr6_31549556_T_G | 31549556 | 1.54 | 1.04 | 2.24 | 0.028217 | 0.04 | 0.05 | 0.04 | SNP |
| chr6_33162228_A_T | 33162228 | 1.31 | 1.03 | 1.67 | 0.028241 | 0.12 | 0.13 | 0.12 | SNP |
| chr6_29895469_G_A | 29895469 | 1.37 | 1.03 | 1.81 | 0.028273 | 0.14 | 0.16 | 0.14 | SNP |
| chr6_31367882_G_A | 31367882 | 1.56 | 1.05 | 2.32 | 0.028274 | 0.17 | 0.19 | 0.17 | SNP |
| chr6_32063896_G_A | 32063896 | 0.53 | 0.29 | 0.91 | 0.028406 | 0.04 | 0.02 | 0.04 | SNP |
| xHLA_2field_DRB1_07_01 | 32546552 | 1.39 | 1.03 | 1.86 | 0.028839 | 0.08 | 0.10 | 0.08 | HLA (xHLA) |
| chr6_31275663_C_A | 31275663 | 0.74 | 0.56 | 0.97 | 0.029038 | 0.19 | 0.14 | 0.20 | SNP |
| chr6_32629509_A_C | 32629509 | 1.28 | 1.03 | 1.60 | 0.029208 | 0.37 | 0.42 | 0.36 | SNP |
| chr6_31129310_C_T | 31129310 | 1.66 | 1.05 | 2.63 | 0.029274 | 0.12 | 0.13 | 0.12 | SNP |
| chr6_32604642_A_G | 32604642 | 1.30 | 1.03 | 1.66 | 0.029374 | 0.28 | 0.34 | 0.26 | SNP |
| chr6_29896114_G_A | 29896114 | 0.82 | 0.69 | 0.98 | 0.029410 | 0.48 | 0.47 | 0.48 | SNP |
| chr6_31236679_T_C | 31236679 | 0.78 | 0.62 | 0.97 | 0.029411 | 0.41 | 0.39 | 0.41 | SNP |
| chr6_32520460_C_T | 32520460 | 1.28 | 1.02 | 1.59 | 0.029452 | 0.31 | 0.37 | 0.30 | SNP |
| chr6_33280309_C_T | 33280309 | 1.20 | 1.02 | 1.42 | 0.029548 | 0.35 | 0.40 | 0.34 | SNP |
| chr6_31708463_C_A | 31708463 | 1.79 | 1.06 | 3.02 | 0.029607 | 0.12 | 0.13 | 0.12 | SNP |
| chr6_31243895_T_C | 31243895 | 1.28 | 1.02 | 1.60 | 0.029611 | 0.39 | 0.41 | 0.38 | SNP |
| chr6_32629331_A_G | 32629331 | 1.29 | 1.03 | 1.61 | 0.029675 | 0.36 | 0.42 | 0.34 | SNP |
| chr6_31085340_G_A | 31085340 | 0.79 | 0.63 | 0.98 | 0.029850 | 0.23 | 0.22 | 0.24 | SNP |
| chr6_31241916_T_C | 31241916 | 0.75 | 0.57 | 0.97 | 0.029908 | 0.17 | 0.16 | 0.17 | SNP |
| chr6_32803999_A_T | 32803999 | 0.50 | 0.26 | 0.90 | 0.029928 | 0.03 | 0.01 | 0.03 | SNP |
| chr6_31803130_C_T | 31803130 | 0.58 | 0.35 | 0.93 | 0.029950 | 0.04 | 0.03 | 0.04 | SNP |
| chr6_32193449_T_C | 32193449 | 0.78 | 0.62 | 0.97 | 0.029957 | 0.16 | 0.14 | 0.17 | SNP |
| chr6_31473957_C_G | 31473957 | 1.71 | 1.05 | 2.77 | 0.030128 | 0.12 | 0.13 | 0.12 | SNP |
| chr6_29769521_G_A | 29769521 | 1.26 | 1.02 | 1.56 | 0.030173 | 0.30 | 0.31 | 0.30 | SNP |
| chr6_32559710_C_T | 32559710 | 1.36 | 1.03 | 1.81 | 0.030184 | 0.20 | 0.26 | 0.19 | SNP |
| chr6_31243830_A_G | 31243830 | 1.28 | 1.02 | 1.60 | 0.030282 | 0.39 | 0.41 | 0.38 | SNP |
| chr6_29943039_G_T | 29943039 | 0.58 | 0.35 | 0.93 | 0.030345 | 0.04 | 0.03 | 0.04 | SNP |
| chr6_32558284_T_C | 32558284 | 0.69 | 0.49 | 0.96 | 0.030357 | 0.08 | 0.06 | 0.08 | SNP |
| chr6_32629269_C_T | 32629269 | 1.28 | 1.02 | 1.61 | 0.030447 | 0.23 | 0.27 | 0.22 | SNP |
| chr6_31325430_A_G | 31325430 | 1.24 | 1.02 | 1.51 | 0.030463 | 0.48 | 0.51 | 0.47 | SNP |
| chr6_32549259_A_G | 32549259 | 1.41 | 1.03 | 1.93 | 0.030510 | 0.18 | 0.20 | 0.18 | SNP |
| chr6_29853750_G_T | 29853750 | 1.36 | 1.03 | 1.78 | 0.030583 | 0.14 | 0.16 | 0.14 | SNP |
| chr6_32024234_G_A | 32024234 | 2.19 | 1.04 | 4.36 | 0.030686 | 0.01 | 0.02 | 0.01 | SNP |
| chr6_31324953_T_C | 31324953 | 1.33 | 1.03 | 1.72 | 0.030732 | 0.37 | 0.40 | 0.37 | SNP |
| chr6_31733650_G_A | 31733650 | 0.64 | 0.43 | 0.95 | 0.030782 | 0.06 | 0.04 | 0.06 | SNP |
| chr6_31247097_T_C | 31247097 | 0.45 | 0.21 | 0.88 | 0.030832 | 0.02 | 0.01 | 0.03 | SNP |
| chr6_32782605_C_T | 32782605 | 1.24 | 1.02 | 1.51 | 0.030938 | 0.37 | 0.39 | 0.36 | SNP |
| chr6_33359142_A_G | 33359142 | 1.23 | 1.02 | 1.49 | 0.031031 | 0.40 | 0.45 | 0.39 | SNP |
| chr6_31241109_C_T | 31241109 | 1.35 | 1.02 | 1.76 | 0.031044 | 0.10 | 0.11 | 0.09 | SNP |
| chr6_32725832_A_G | 32725832 | 0.74 | 0.56 | 0.97 | 0.031065 | 0.20 | 0.17 | 0.21 | SNP |
| chr6_32636528_A_G | 32636528 | 1.26 | 1.02 | 1.55 | 0.031100 | 0.41 | 0.44 | 0.40 | SNP |
| chr6_31237354_G_A | 31237354 | 0.63 | 0.41 | 0.94 | 0.031133 | 0.06 | 0.04 | 0.06 | SNP |
| chr6_32713018_C_T | 32713018 | 0.73 | 0.55 | 0.97 | 0.031203 | 0.11 | 0.08 | 0.12 | SNP |
| chr6_32710307_G_A | 32710307 | 0.74 | 0.55 | 0.97 | 0.031232 | 0.11 | 0.08 | 0.12 | SNP |
| chr6_32408044_C_A | 32408044 | 0.79 | 0.63 | 0.98 | 0.031264 | 0.50 | 0.55 | 0.49 | SNP |
| chr6_31478960_G_A | 31478960 | 1.66 | 1.03 | 2.62 | 0.031349 | 0.03 | 0.04 | 0.03 | SNP |
| chr6_29909970_T_A | 29909970 | 0.61 | 0.38 | 0.94 | 0.031445 | 0.05 | 0.03 | 0.05 | SNP |
| chr6_31125407_G_A | 31125407 | 0.70 | 0.50 | 0.96 | 0.031555 | 0.08 | 0.06 | 0.09 | SNP |
| chr6_32627456_A_G | 32627456 | 0.78 | 0.62 | 0.98 | 0.031592 | 0.36 | 0.31 | 0.37 | SNP |
| chr6_32338283_G_T | 32338283 | 1.28 | 1.02 | 1.60 | 0.031728 | 0.26 | 0.28 | 0.26 | SNP |
| chr6_29908789_T_C | 29908789 | 0.61 | 0.38 | 0.94 | 0.031756 | 0.05 | 0.03 | 0.05 | SNP |
| chr6_32559648_G_T | 32559648 | 1.35 | 1.03 | 1.77 | 0.031832 | 0.19 | 0.25 | 0.18 | SNP |
| chr6_32406579_C_T | 32406579 | 1.43 | 1.03 | 1.98 | 0.031907 | 0.18 | 0.19 | 0.17 | SNP |
| chr6_33633527_A_G | 33633527 | 1.22 | 1.02 | 1.47 | 0.031921 | 0.30 | 0.34 | 0.29 | SNP |
| chr6_32811629_G_T | 32811629 | 1.32 | 1.02 | 1.70 | 0.031926 | 0.14 | 0.18 | 0.13 | SNP |
| chr6_33152865_G_A | 33152865 | 1.27 | 1.02 | 1.58 | 0.031927 | 0.15 | 0.17 | 0.15 | SNP |

| | | | | | | | | | |
|---|---|---|---|---|---|---|---|---|---|
| chr6_31326960_G_A | 31326960 | 0.81 | 0.67 | 0.98 | 0.032073 | 0.38 | 0.34 | 0.39 | SNP |
| chr6_33268737_C_A | 33268737 | 1.20 | 1.01 | 1.42 | 0.032630 | 0.35 | 0.40 | 0.34 | SNP |
| chr6_32409058_C_T | 32409058 | 1.43 | 1.03 | 1.97 | 0.032671 | 0.18 | 0.19 | 0.17 | SNP |
| chr6_29895604_C_G | 29895604 | 0.83 | 0.70 | 0.98 | 0.032799 | 0.45 | 0.44 | 0.45 | SNP |
| chr6_32163799_T_C | 32163799 | 1.63 | 1.03 | 2.52 | 0.032926 | 0.03 | 0.05 | 0.03 | SNP |
| chr6_29896084_A_G | 29896084 | 0.83 | 0.69 | 0.98 | 0.033017 | 0.46 | 0.45 | 0.46 | SNP |
| chr6_31730568_A_G | 31730568 | 0.68 | 0.48 | 0.96 | 0.033018 | 0.07 | 0.05 | 0.08 | SNP |
| chr6_31543101_G_A | 31543101 | 1.57 | 1.03 | 2.37 | 0.033208 | 0.04 | 0.05 | 0.03 | SNP |
| chr6_33288075_C_A | 33288075 | 1.20 | 1.01 | 1.41 | 0.033684 | 0.35 | 0.40 | 0.34 | SNP |
| chr6_33545125_A_G | 33545125 | 1.25 | 1.02 | 1.53 | 0.033760 | 0.21 | 0.24 | 0.20 | SNP |
| chr6_32605646_T_G | 32605646 | 1.27 | 1.02 | 1.59 | 0.033804 | 0.30 | 0.36 | 0.29 | SNP |
| chr6_32526994_G_A | 32526994 | 1.69 | 1.03 | 2.72 | 0.033898 | 0.02 | 0.03 | 0.02 | SNP |
| chr6_31084792_C_T | 31084792 | 0.81 | 0.67 | 0.98 | 0.034131 | 0.48 | 0.45 | 0.48 | SNP |
| chr6_33382241_T_C | 33382241 | 1.19 | 1.01 | 1.40 | 0.034350 | 0.41 | 0.45 | 0.40 | SNP |
| chr6_33288271_A_G | 33288271 | 1.20 | 1.01 | 1.41 | 0.034849 | 0.35 | 0.40 | 0.34 | SNP |
| chr6_32189192_G_A | 32189192 | 0.59 | 0.36 | 0.94 | 0.035050 | 0.04 | 0.03 | 0.04 | SNP |
| chr6_32441008_C_T | 32441008 | 1.28 | 1.02 | 1.61 | 0.035110 | 0.31 | 0.36 | 0.29 | SNP |
| chr6_32627700_A_G | 32627700 | 0.77 | 0.61 | 0.98 | 0.035172 | 0.19 | 0.15 | 0.20 | SNP |
| chr6_32134510_G_A | 32134510 | 1.44 | 1.02 | 2.01 | 0.035326 | 0.19 | 0.21 | 0.18 | SNP |
| chr6_31133677_G_A | 31133677 | 0.51 | 0.26 | 0.92 | 0.035352 | 0.02 | 0.01 | 0.03 | SNP |
| chr6_32725271_G_A | 32725271 | 0.75 | 0.57 | 0.98 | 0.035398 | 0.21 | 0.18 | 0.22 | SNP |
| chr6_29897129_T_C | 29897129 | 1.24 | 1.01 | 1.52 | 0.035477 | 0.22 | 0.23 | 0.22 | SNP |
| chr6_29854399_G_A | 29854399 | 1.24 | 1.01 | 1.52 | 0.035498 | 0.23 | 0.24 | 0.23 | SNP |
| chr6_32975257_C_T | 32975257 | 1.20 | 1.01 | 1.43 | 0.035528 | 0.47 | 0.52 | 0.46 | SNP |
| chr6_32606217_G_A | 32606217 | 1.27 | 1.02 | 1.59 | 0.035601 | 0.30 | 0.36 | 0.29 | SNP |
| chr6_29924889_C_A | 29924889 | 0.81 | 0.67 | 0.99 | 0.035647 | 0.40 | 0.39 | 0.40 | SNP |
| chr6_31241537_T_C | 31241537 | 1.25 | 1.01 | 1.54 | 0.035672 | 0.29 | 0.33 | 0.29 | SNP |
| chr6_32814975_T_C | 32814975 | 0.78 | 0.61 | 0.98 | 0.035700 | 0.15 | 0.13 | 0.16 | SNP |
| chr6_31321948_T_G | 31321948 | 1.40 | 1.02 | 1.91 | 0.035765 | 0.09 | 0.09 | 0.09 | SNP |
| chr6_29892732_G_A | 29892732 | 1.27 | 1.01 | 1.59 | 0.035824 | 0.20 | 0.22 | 0.20 | SNP |
| chr6_29855706_G_C | 29855706 | 1.28 | 1.01 | 1.62 | 0.035971 | 0.24 | 0.24 | 0.23 | SNP |
| chr6_32335657_A_G | 32335657 | 1.50 | 1.02 | 2.17 | 0.036030 | 0.04 | 0.05 | 0.03 | SNP |
| chr6_32629330_C_T | 32629330 | 1.27 | 1.01 | 1.60 | 0.036436 | 0.23 | 0.27 | 0.22 | SNP |
| chr6_31837277_A_C | 31837277 | 1.25 | 1.01 | 1.55 | 0.036450 | 0.49 | 0.49 | 0.49 | SNP |
| chr6_31477324_A_C | 31477324 | 0.45 | 0.19 | 0.90 | 0.036457 | 0.02 | 0.01 | 0.03 | SNP |
| chr6_31325030_C_T | 31325030 | 1.43 | 1.02 | 2.01 | 0.036988 | 0.19 | 0.20 | 0.18 | SNP |
| chr6_30228230_A_C | 30228230 | 0.75 | 0.57 | 0.98 | 0.037202 | 0.20 | 0.15 | 0.21 | SNP |
| chr6_32377053_C_T | 32377053 | 1.69 | 1.01 | 2.74 | 0.037666 | 0.02 | 0.03 | 0.02 | SNP |
| chr6_29943490_G_A | 29943490 | 1.24 | 1.01 | 1.52 | 0.038108 | 0.21 | 0.23 | 0.21 | SNP |
| chr6_31238029_C_T | 31238029 | 1.31 | 1.01 | 1.69 | 0.038137 | 0.36 | 0.39 | 0.35 | SNP |
| chr6_31238632_C_T | 31238632 | 0.78 | 0.62 | 0.99 | 0.038292 | 0.21 | 0.21 | 0.21 | SNP |
| chr6_29896773_C_T | 29896773 | 1.26 | 1.01 | 1.57 | 0.038366 | 0.23 | 0.25 | 0.23 | SNP |
| chr6_31240614_C_T | 31240614 | 0.50 | 0.25 | 0.92 | 0.038610 | 0.02 | 0.01 | 0.02 | SNP |
| chr6_31236862_C_T | 31236862 | 0.65 | 0.42 | 0.96 | 0.038710 | 0.06 | 0.04 | 0.06 | SNP |
| chr6_31237040_A_T | 31237040 | 0.65 | 0.42 | 0.96 | 0.038710 | 0.06 | 0.04 | 0.06 | SNP |
| chr6_31237254_C_T | 31237254 | 0.65 | 0.42 | 0.96 | 0.038710 | 0.06 | 0.04 | 0.06 | SNP |
| chr6_32014456_C_T | 32014456 | 1.43 | 1.01 | 2.00 | 0.038965 | 0.05 | 0.07 | 0.05 | SNP |
| chr6_29911207_G_A | 29911207 | 0.80 | 0.65 | 0.99 | 0.039427 | 0.41 | 0.40 | 0.41 | SNP |
| chr6_32032421_C_T | 32032421 | 0.72 | 0.52 | 0.98 | 0.039537 | 0.11 | 0.08 | 0.12 | SNP |
| chr6_33282338_G_A | 33282338 | 0.79 | 0.62 | 0.99 | 0.039541 | 0.17 | 0.15 | 0.18 | SNP |
| chr6_32603177_A_G | 32603177 | 1.28 | 1.01 | 1.62 | 0.039594 | 0.19 | 0.19 | 0.19 | SNP |
| chr6_29864478_G_A | 29864478 | 1.22 | 1.01 | 1.48 | 0.039613 | 0.29 | 0.31 | 0.29 | SNP |
| chr6_31118903_C_T | 31118903 | 0.52 | 0.26 | 0.93 | 0.039710 | 0.03 | 0.01 | 0.03 | SNP |
| chr6_32710695_T_G | 32710695 | 0.74 | 0.55 | 0.98 | 0.039804 | 0.11 | 0.08 | 0.11 | SNP |
| chr6_30226123_C_T | 30226123 | 1.37 | 1.01 | 1.84 | 0.040458 | 0.13 | 0.14 | 0.12 | SNP |
| chr6_33641194_C_T | 33641194 | 1.23 | 1.01 | 1.49 | 0.040493 | 0.29 | 0.33 | 0.28 | SNP |
| chr6_31134033_A_T | 31134033 | 0.51 | 0.26 | 0.94 | 0.040591 | 0.02 | 0.02 | 0.03 | SNP |
| chr6_31860156_C_T | 31860156 | 1.43 | 1.01 | 2.00 | 0.040596 | 0.08 | 0.07 | 0.08 | SNP |
| chr6_31864304_A_G | 31864304 | 1.30 | 1.01 | 1.66 | 0.040676 | 0.37 | 0.39 | 0.37 | SNP |
| chr6_32520338_G_A | 32520338 | 1.44 | 1.01 | 2.05 | 0.040766 | 0.17 | 0.18 | 0.16 | SNP |
| chr6_31244262_A_G | 31244262 | 0.51 | 0.25 | 0.93 | 0.040836 | 0.02 | 0.01 | 0.03 | SNP |
| chr6_33138677_G_A | 33138677 | 1.21 | 1.01 | 1.45 | 0.041083 | 0.25 | 0.28 | 0.25 | SNP |
| chr6_31320833_C_T | 31320833 | 0.53 | 0.28 | 0.94 | 0.041160 | 0.03 | 0.02 | 0.03 | SNP |
| chr6_32029849_G_A | 32029849 | 0.71 | 0.51 | 0.98 | 0.041379 | 0.09 | 0.07 | 0.10 | SNP |
| chr6_32806391_C_T | 32806391 | 1.25 | 1.01 | 1.54 | 0.041527 | 0.45 | 0.50 | 0.43 | SNP |
| chr6_32945843_T_A | 32945843 | 1.83 | 1.00 | 3.21 | 0.041586 | 0.02 | 0.02 | 0.01 | SNP |
| chr6_29694427_T_C | 29694427 | 1.25 | 1.01 | 1.55 | 0.041883 | 0.20 | 0.21 | 0.20 | SNP |
| chr6_31631531_G_A | 31631531 | 1.97 | 1.00 | 3.71 | 0.041978 | 0.02 | 0.02 | 0.02 | SNP |
| chr6_29858253_G_A | 29858253 | 1.33 | 1.01 | 1.75 | 0.042224 | 0.15 | 0.16 | 0.14 | SNP |
| chr6_32193564_C_T | 32193564 | 0.80 | 0.64 | 0.99 | 0.042247 | 0.21 | 0.17 | 0.22 | SNP |
| chr6_32340176_C_T | 32340176 | 0.77 | 0.59 | 0.99 | 0.042664 | 0.24 | 0.21 | 0.24 | SNP |
| chr6_32608844_A_G | 32608844 | 1.27 | 1.01 | 1.60 | 0.042824 | 0.28 | 0.33 | 0.26 | SNP |
| chr6_31127745_C_A | 31127745 | 0.64 | 0.40 | 0.97 | 0.042927 | 0.05 | 0.03 | 0.05 | SNP |
| chr6_31246969_A_G | 31246969 | 0.72 | 0.52 | 0.98 | 0.043043 | 0.11 | 0.08 | 0.12 | SNP |
| chr6_31113149_T_A | 31113149 | 0.64 | 0.41 | 0.97 | 0.043176 | 0.05 | 0.03 | 0.06 | SNP |
| chr6_32411846_G_C | 32411846 | 1.27 | 1.01 | 1.59 | 0.043377 | 0.25 | 0.27 | 0.25 | SNP |
| chr6_32707508_T_C | 32707508 | 0.75 | 0.56 | 0.99 | 0.043616 | 0.10 | 0.08 | 0.11 | SNP |
| chr6_32707569_G_C | 32707569 | 0.75 | 0.56 | 0.99 | 0.043616 | 0.10 | 0.08 | 0.11 | SNP |
| chr6_32603660_T_C | 32603660 | 0.82 | 0.67 | 0.99 | 0.043631 | 0.38 | 0.37 | 0.38 | SNP |
| chr6_32708890_A_G | 32708890 | 0.75 | 0.56 | 0.99 | 0.043641 | 0.10 | 0.08 | 0.11 | SNP |
| chr6_32708413_G_A | 32708413 | 0.75 | 0.56 | 0.99 | 0.043688 | 0.11 | 0.08 | 0.11 | SNP |
| chr6_32035427_T_C | 32035427 | 0.72 | 0.52 | 0.98 | 0.043743 | 0.09 | 0.07 | 0.10 | SNP |
| chr6_32412571_T_A | 32412571 | 1.26 | 1.00 | 1.58 | 0.043756 | 0.25 | 0.27 | 0.25 | SNP |
| chr6_29896659_C_T | 29896659 | 0.83 | 0.70 | 0.99 | 0.043782 | 0.45 | 0.44 | 0.45 | SNP |
| chr6_29911069_A_T | 29911069 | 0.69 | 0.48 | 0.98 | 0.043874 | 0.09 | 0.06 | 0.09 | SNP |
| chr6_30864927_C_T | 30864927 | 1.45 | 1.00 | 2.06 | 0.043990 | 0.05 | 0.06 | 0.05 | SNP |
| chr6_32707614_A_G | 32707614 | 0.75 | 0.56 | 0.99 | 0.044013 | 0.10 | 0.08 | 0.11 | SNP |
| chr6_32707638_C_T | 32707638 | 0.75 | 0.56 | 0.99 | 0.044013 | 0.10 | 0.08 | 0.11 | SNP |
| chr6_32707641_C_A | 32707641 | 0.75 | 0.56 | 0.99 | 0.044013 | 0.10 | 0.08 | 0.11 | SNP |
| chr6_32707740_T_C | 32707740 | 0.75 | 0.56 | 0.99 | 0.044013 | 0.10 | 0.08 | 0.11 | SNP |
| chr6_32707783_A_G | 32707783 | 0.75 | 0.56 | 0.99 | 0.044013 | 0.10 | 0.08 | 0.11 | SNP |
| chr6_32708798_T_C | 32708798 | 0.75 | 0.56 | 0.99 | 0.044013 | 0.10 | 0.08 | 0.11 | SNP |
| chr6_32709179_A_G | 32709179 | 0.75 | 0.56 | 0.99 | 0.044013 | 0.10 | 0.08 | 0.11 | SNP |
| chr6_32709586_G_A | 32709586 | 0.75 | 0.56 | 0.99 | 0.044013 | 0.10 | 0.08 | 0.11 | SNP |
| chr6_32709738_A_G | 32709738 | 0.75 | 0.56 | 0.99 | 0.044013 | 0.10 | 0.08 | 0.11 | SNP |
| chr6_31245911_G_A | 31245911 | 0.66 | 0.43 | 0.97 | 0.044234 | 0.06 | 0.04 | 0.06 | SNP |
| chr6_29972984_G_A | 29972984 | 1.23 | 1.00 | 1.51 | 0.044415 | 0.28 | 0.28 | 0.28 | SNP |
| chr6_30226811_C_G | 30226811 | 0.79 | 0.62 | 0.99 | 0.044440 | 0.19 | 0.15 | 0.20 | SNP |
| chr6_32321554_G_T | 32321554 | 1.48 | 1.00 | 2.16 | 0.044524 | 0.04 | 0.05 | 0.03 | SNP |
| chr6_32781954_A_G | 32781954 | 0.47 | 0.21 | 0.93 | 0.044565 | 0.02 | 0.01 | 0.02 | SNP |
| chr6_32553662_A_G | 32553662 | 0.81 | 0.65 | 0.99 | 0.044810 | 0.39 | 0.35 | 0.40 | SNP |
| chr6_32974081_C_T | 32974081 | 1.19 | 1.00 | 1.41 | 0.044820 | 0.48 | 0.53 | 0.47 | SNP |
| chr6_32411362_A_C | 32411362 | 1.31 | 1.00 | 1.71 | 0.044869 | 0.14 | 0.14 | 0.14 | SNP |
| chr6_32412435_G_A | 32412435 | 1.31 | 1.00 | 1.71 | 0.044869 | 0.14 | 0.14 | 0.14 | SNP |
| chr6_32707360_G_A | 32707360 | 0.75 | 0.56 | 0.99 | 0.045113 | 0.10 | 0.08 | 0.11 | SNP |
| xHLA_2field_DQB1_06_03 | 32627244 | 0.69 | 0.48 | 0.98 | 0.045192 | 0.08 | 0.06 | 0.09 | HLA (xHLA) |
| chr6_30228879_C_T | 30228879 | 0.78 | 0.62 | 0.99 | 0.045265 | 0.19 | 0.15 | 0.20 | SNP |
| chr6_32373378_A_T | 32373378 | 1.41 | 1.00 | 1.97 | 0.045343 | 0.07 | 0.10 | 0.06 | SNP |
| chr6_29942324_T_C | 29942324 | 0.83 | 0.69 | 1.00 | 0.045405 | 0.49 | 0.48 | 0.50 | SNP |
| chr6_31727897_C_T | 31727897 | 1.69 | 1.01 | 2.83 | 0.045470 | 0.12 | 0.12 | 0.12 | SNP |
| chr6_32710221_C_T | 32710221 | 1.20 | 1.00 | 1.44 | 0.045662 | 0.39 | 0.41 | 0.39 | SNP |
| chr6_29910947_T_A | 29910947 | 0.69 | 0.47 | 0.98 | 0.045786 | 0.09 | 0.06 | 0.09 | SNP |
| chr6_32707893_A_C | 32707893 | 0.75 | 0.56 | 0.99 | 0.045929 | 0.10 | 0.08 | 0.11 | SNP |
| chr6_32797309_T_G | 32797309 | 1.37 | 1.00 | 1.85 | 0.045943 | 0.08 | 0.11 | 0.07 | SNP |
| chr6_29895929_C_G | 29895929 | 0.84 | 0.70 | 1.00 | 0.046013 | 0.47 | 0.47 | 0.48 | SNP |
| chr6_32707427_G_C | 32707427 | 0.75 | 0.56 | 0.99 | 0.046054 | 0.10 | 0.08 | 0.11 | SNP |
| chr6_32377116_C_G | 32377116 | 1.27 | 1.01 | 1.60 | 0.046087 | 0.48 | 0.51 | 0.47 | SNP |
| chr6_29910725_C_G | 29910725 | 0.68 | 0.46 | 0.98 | 0.046121 | 0.09 | 0.06 | 0.09 | SNP |
| chr6_32707071_A_G | 32707071 | 0.74 | 0.55 | 0.99 | 0.046261 | 0.11 | 0.08 | 0.11 | SNP |
| chr6_31244154_A_C | 31244154 | 0.66 | 0.43 | 0.98 | 0.046465 | 0.06 | 0.04 | 0.06 | SNP |
| chr6_31238179_C_T | 31238179 | 0.66 | 0.43 | 0.98 | 0.046530 | 0.06 | 0.04 | 0.06 | SNP |
| chr6_31238334_C_T | 31238334 | 0.66 | 0.43 | 0.98 | 0.046530 | 0.06 | 0.04 | 0.06 | SNP |
| chr6_32556677_C_A | 32556677 | 1.35 | 1.00 | 1.81 | 0.046587 | 0.10 | 0.13 | 0.10 | SNP |
| chr6_32942277_G_A | 32942277 | 1.79 | 0.99 | 3.15 | 0.047062 | 0.02 | 0.02 | 0.01 | SNP |
| chr6_32192978_T_A | 32192978 | 0.83 | 0.69 | 1.00 | 0.047129 | 0.30 | 0.27 | 0.30 | SNP |
| chr6_32056580_A_C | 32056580 | 0.72 | 0.52 | 0.99 | 0.047190 | 0.09 | 0.07 | 0.10 | SNP |
| chr6_31244181_T_C | 31244181 | 1.42 | 1.00 | 2.01 | 0.047216 | 0.18 | 0.19 | 0.17 | SNP |
| chr6_29911198_T_C | 29911198 | 0.81 | 0.66 | 1.00 | 0.047348 | 0.41 | 0.41 | 0.41 | SNP |
| chr6_32609358_C_T | 32609358 | 1.27 | 1.00 | 1.60 | 0.047355 | 0.29 | 0.34 | 0.27 | SNP |
| chr6_32818678_G_A | 32818678 | 1.22 | 1.00 | 1.48 | 0.047546 | 0.24 | 0.26 | 0.24 | SNP |
| chr6_32707124_T_A | 32707124 | 0.75 | 0.56 | 0.99 | 0.047682 | 0.10 | 0.08 | 0.11 | SNP |
| chr6_29895626_T_A | 29895626 | 1.32 | 1.00 | 1.72 | 0.047875 | 0.15 | 0.16 | 0.15 | SNP |
| chr6_31325270_C_G | 31325270 | 1.22 | 1.00 | 1.48 | 0.047928 | 0.48 | 0.51 | 0.48 | SNP |
| chr6_32974401_G_T | 32974401 | 1.19 | 1.00 | 1.40 | 0.048000 | 0.48 | 0.53 | 0.47 | SNP |
| chr6_29911545_G_A | 29911545 | 0.73 | 0.53 | 0.99 | 0.048170 | 0.09 | 0.07 | 0.09 | SNP |
| chr6_29943340_C_G | 29943340 | 1.22 | 1.00 | 1.49 | 0.048210 | 0.29 | 0.30 | 0.28 | SNP |
| chr6_33272677_C_T | 33272677 | 1.34 | 1.00 | 1.78 | 0.048361 | 0.08 | 0.09 | 0.07 | SNP |
| chr6_31237950_C_T | 31237950 | 1.29 | 1.00 | 1.67 | 0.048364 | 0.36 | 0.38 | 0.36 | SNP |
| xHLA_2field_DQB1_06_02 | 32627244 | 1.43 | 1.00 | 2.03 | 0.048461 | 0.17 | 0.18 | 0.17 | HLA (xHLA) |
| chr6_30228881_T_C | 30228881 | 0.79 | 0.62 | 1.00 | 0.048755 | 0.19 | 0.15 | 0.20 | SNP |
| xHLA_2field_C_03_04 | 31236526 | 0.77 | 0.60 | 1.00 | 0.048831 | 0.15 | 0.15 | 0.15 | HLA (xHLA) |
| chr6_29893837_G_A | 29893837 | 1.21 | 1.00 | 1.46 | 0.048885 | 0.46 | 0.47 | 0.46 | SNP |
| chr6_32083175_C_T | 32083175 | 1.24 | 1.00 | 1.54 | 0.048969 | 0.30 | 0.35 | 0.29 | SNP |
| chr6_33152966_A_C | 33152966 | 1.28 | 1.00 | 1.64 | 0.049270 | 0.12 | 0.13 | 0.12 | SNP |
| chr6_30851909_C_A | 30851909 | 1.58 | 1.00 | 2.48 | 0.049354 | 0.12 | 0.15 | 0.12 | SNP |
| chr6_31244068_A_G | 31244068 | 1.41 | 1.00 | 1.98 | 0.049684 | 0.18 | 0.20 | 0.18 | SNP |
| chr6_32340872_C_G | 32340872 | 0.78 | 0.60 | 1.00 | 0.049837 | 0.24 | 0.21 | 0.24 | SNP |
| chr6_31607050_T_C | 31607050 | 1.41 | 1.00 | 2.00 | 0.049890 | 0.19 | 0.20 | 0.19 | SNP |
| chr6_31323020_T_C | 31323020 | 0.75 | 0.56 | 1.00 | 0.049967 | 0.14 | 0.11 | 0.14 | SNP |

ANCA = anitneutrophil cytoplasmic antibody; PR3-ANCA+ = proteinase 3 ANCA positive; AAV = ANCA-associated vasculitis; SNP = single nucleotide polymorphism; OR = Odds ratio; CI = confidence interval; MAF = minor allele frequency. SNPs and HLA alleles with P < 0.05 included in the table.

**Supplemtary Table 3**. Association analysis of patients with GPA versus controls, adjusting for sex and genetic structure (principal components 1-4).

| Variant | position | OR | CI_lower | CI_upper | p | MAF | MAF_cases | MAF_controls | Type (SNP, HLA (xHLA)) |
|---|---|---|---|---|---|---|---|---|---|
| xHLA_2field_DPB1_04_01 | 33043767 | 2.97 | 2.51 | 3.54 | 3.47E-35 | 0.48 | 0.72 | 0.42 | HLA (xHLA) |
| chr6_33052950_T_C | 33052950 | 0.13 | 0.09 | 0.18 | 1.36E-34 | 0.23 | 0.05 | 0.27 | SNP |
| chr6_33052958_C_T | 33052958 | 0.14 | 0.10 | 0.18 | 2.26E-34 | 0.23 | 0.05 | 0.27 | SNP |
| chr6_33052986_T_C | 33052986 | 0.13 | 0.10 | 0.18 | 2.37E-34 | 0.23 | 0.05 | 0.28 | SNP |
| chr6_33053455_C_T | 33053455 | 0.14 | 0.10 | 0.19 | 3.42E-34 | 0.23 | 0.05 | 0.28 | SNP |
| chr6_33053728_C_T | 33053728 | 0.14 | 0.10 | 0.18 | 3.99E-34 | 0.23 | 0.05 | 0.28 | SNP |
| chr6_33053723_T_C | 33053723 | 0.14 | 0.10 | 0.19 | 4.64E-34 | 0.23 | 0.05 | 0.28 | SNP |
| chr6_33053468_G_T | 33053468 | 0.14 | 0.10 | 0.19 | 5.81E-34 | 0.23 | 0.05 | 0.28 | SNP |
| chr6_33053477_G_C | 33053477 | 0.14 | 0.10 | 0.19 | 6.00E-34 | 0.23 | 0.05 | 0.28 | SNP |
| chr6_33053399_T_C | 33053399 | 0.14 | 0.10 | 0.19 | 6.58E-34 | 0.23 | 0.05 | 0.28 | SNP |
| chr6_33053877_G_A | 33053877 | 0.14 | 0.10 | 0.19 | 8.21E-34 | 0.23 | 0.06 | 0.28 | SNP |
| chr6_33053682_G_A | 33053682 | 0.14 | 0.10 | 0.19 | 8.84E-34 | 0.23 | 0.06 | 0.28 | SNP |
| chr6_33054046_T_G | 33054046 | 0.14 | 0.10 | 0.19 | 9.06E-34 | 0.23 | 0.06 | 0.28 | SNP |
| chr6_33053887_A_G | 33053887 | 0.14 | 0.10 | 0.19 | 9.27E-34 | 0.23 | 0.06 | 0.28 | SNP |
| chr6_33053982_G_C | 33053982 | 0.14 | 0.10 | 0.19 | 9.27E-34 | 0.23 | 0.06 | 0.28 | SNP |
| chr6_33053871_A_G | 33053871 | 0.14 | 0.10 | 0.19 | 9.33E-34 | 0.23 | 0.06 | 0.28 | SNP |
| chr6_33054353_G_A | 33054353 | 0.14 | 0.10 | 0.19 | 9.63E-34 | 0.23 | 0.06 | 0.28 | SNP |
| chr6_33053772_G_A | 33053772 | 0.14 | 0.10 | 0.19 | 9.68E-34 | 0.23 | 0.06 | 0.28 | SNP |
| chr6_33054656_A_C | 33054656 | 0.14 | 0.10 | 0.19 | 9.72E-34 | 0.23 | 0.06 | 0.28 | SNP |
| chr6_33054675_C_G | 33054675 | 0.14 | 0.10 | 0.19 | 9.72E-34 | 0.23 | 0.06 | 0.28 | SNP |
| chr6_33054683_A_G | 33054683 | 0.14 | 0.10 | 0.19 | 9.72E-34 | 0.23 | 0.06 | 0.28 | SNP |
| chr6_33054687_G_A | 33054687 | 0.14 | 0.10 | 0.19 | 9.72E-34 | 0.23 | 0.06 | 0.28 | SNP |
| chr6_33053890_G_A | 33053890 | 0.14 | 0.10 | 0.19 | 9.81E-34 | 0.23 | 0.06 | 0.28 | SNP |
| chr6_33054152_A_G | 33054152 | 0.14 | 0.10 | 0.19 | 9.81E-34 | 0.23 | 0.06 | 0.28 | SNP |
| chr6_33055009_C_A | 33055009 | 0.14 | 0.10 | 0.19 | 9.97E-34 | 0.23 | 0.06 | 0.28 | SNP |
| chr6_33054711_G_T | 33054711 | 0.14 | 0.10 | 0.19 | 1.02E-33 | 0.23 | 0.06 | 0.28 | SNP |
| chr6_33054721_C_T | 33054721 | 0.14 | 0.10 | 0.19 | 1.02E-33 | 0.23 | 0.06 | 0.28 | SNP |
| chr6_33054149_T_C | 33054149 | 0.14 | 0.10 | 0.19 | 1.02E-33 | 0.23 | 0.06 | 0.28 | SNP |
| chr6_33053780_T_A | 33053780 | 0.14 | 0.10 | 0.19 | 1.02E-33 | 0.23 | 0.06 | 0.28 | SNP |
| chr6_33054457_A_G | 33054457 | 0.14 | 0.10 | 0.19 | 1.03E-33 | 0.23 | 0.06 | 0.28 | SNP |
| chr6_33054579_C_T | 33054579 | 0.14 | 0.10 | 0.19 | 1.03E-33 | 0.23 | 0.06 | 0.28 | SNP |
| chr6_33054595_C_T | 33054595 | 0.14 | 0.10 | 0.19 | 1.03E-33 | 0.23 | 0.06 | 0.28 | SNP |
| chr6_33054619_G_A | 33054619 | 0.14 | 0.10 | 0.19 | 1.03E-33 | 0.23 | 0.06 | 0.28 | SNP |
| chr6_33054890_C_T | 33054890 | 0.14 | 0.10 | 0.19 | 1.03E-33 | 0.23 | 0.06 | 0.28 | SNP |
| chr6_33054091_C_T | 33054091 | 0.14 | 0.10 | 0.19 | 1.04E-33 | 0.23 | 0.06 | 0.28 | SNP |
| chr6_33054235_T_G | 33054235 | 0.14 | 0.10 | 0.19 | 1.04E-33 | 0.23 | 0.06 | 0.28 | SNP |
| chr6_33054586_A_G | 33054586 | 0.14 | 0.10 | 0.19 | 1.06E-33 | 0.23 | 0.06 | 0.28 | SNP |
| chr6_33054342_T_A | 33054342 | 0.14 | 0.10 | 0.19 | 1.07E-33 | 0.23 | 0.06 | 0.28 | SNP |
| chr6_33054355_A_C | 33054355 | 0.14 | 0.10 | 0.19 | 1.07E-33 | 0.23 | 0.06 | 0.28 | SNP |
| chr6_33053868_A_G | 33053868 | 0.14 | 0.10 | 0.19 | 1.07E-33 | 0.23 | 0.06 | 0.28 | SNP |
| chr6_33053789_A_G | 33053789 | 0.14 | 0.10 | 0.19 | 1.07E-33 | 0.23 | 0.06 | 0.28 | SNP |
| chr6_33054331_T_C | 33054331 | 0.14 | 0.10 | 0.19 | 1.08E-33 | 0.23 | 0.06 | 0.28 | SNP |
| chr6_33054550_G_A | 33054550 | 0.14 | 0.10 | 0.19 | 1.08E-33 | 0.23 | 0.06 | 0.28 | SNP |
| chr6_33054142_G_C | 33054142 | 0.14 | 0.10 | 0.19 | 1.08E-33 | 0.23 | 0.06 | 0.28 | SNP |
| chr6_33053968_T_A | 33053968 | 0.14 | 0.10 | 0.19 | 1.08E-33 | 0.23 | 0.06 | 0.28 | SNP |
| chr6_33053971_T_C | 33053971 | 0.14 | 0.10 | 0.19 | 1.08E-33 | 0.23 | 0.06 | 0.28 | SNP |
| chr6_33054141_T_C | 33054141 | 0.14 | 0.10 | 0.19 | 1.11E-33 | 0.23 | 0.06 | 0.28 | SNP |
| chr6_33054148_C_T | 33054148 | 0.14 | 0.10 | 0.19 | 1.11E-33 | 0.23 | 0.06 | 0.28 | SNP |
| chr6_33054400_T_C | 33054400 | 0.14 | 0.10 | 0.19 | 1.12E-33 | 0.23 | 0.06 | 0.28 | SNP |
| chr6_33054404_A_G | 33054404 | 0.14 | 0.10 | 0.19 | 1.12E-33 | 0.23 | 0.06 | 0.28 | SNP |
| chr6_33054410_A_C | 33054410 | 0.14 | 0.10 | 0.19 | 1.12E-33 | 0.23 | 0.06 | 0.28 | SNP |
| chr6_33054057_G_A | 33054057 | 0.14 | 0.10 | 0.19 | 1.12E-33 | 0.23 | 0.06 | 0.28 | SNP |
| chr6_33054386_C_T | 33054386 | 0.14 | 0.10 | 0.19 | 1.14E-33 | 0.23 | 0.06 | 0.28 | SNP |
| chr6_33054302_T_C | 33054302 | 0.14 | 0.10 | 0.19 | 1.15E-33 | 0.23 | 0.06 | 0.28 | SNP |
| chr6_33054807_A_G | 33054807 | 0.14 | 0.10 | 0.19 | 1.15E-33 | 0.23 | 0.06 | 0.28 | SNP |
| chr6_33054381_T_C | 33054381 | 0.14 | 0.10 | 0.19 | 1.16E-33 | 0.23 | 0.06 | 0.28 | SNP |
| chr6_33054071_G_C | 33054071 | 0.14 | 0.10 | 0.19 | 1.17E-33 | 0.23 | 0.06 | 0.28 | SNP |
| chr6_33053902_A_G | 33053902 | 0.14 | 0.10 | 0.19 | 1.18E-33 | 0.23 | 0.06 | 0.28 | SNP |
| chr6_33054373_C_T | 33054373 | 0.14 | 0.10 | 0.19 | 1.22E-33 | 0.23 | 0.06 | 0.28 | SNP |
| chr6_33054325_G_C | 33054325 | 0.14 | 0.10 | 0.19 | 1.23E-33 | 0.23 | 0.06 | 0.28 | SNP |
| chr6_33054155_C_T | 33054155 | 0.14 | 0.10 | 0.19 | 1.26E-33 | 0.23 | 0.06 | 0.28 | SNP |
| chr6_33054156_A_G | 33054156 | 0.14 | 0.10 | 0.19 | 1.26E-33 | 0.23 | 0.06 | 0.28 | SNP |
| chr6_33054268_C_A | 33054268 | 0.14 | 0.10 | 0.19 | 1.28E-33 | 0.23 | 0.06 | 0.28 | SNP |
| chr6_33053906_G_T | 33053906 | 0.14 | 0.10 | 0.19 | 1.29E-33 | 0.23 | 0.06 | 0.28 | SNP |
| chr6_33054413_T_G | 33054413 | 0.14 | 0.10 | 0.19 | 1.36E-33 | 0.23 | 0.06 | 0.28 | SNP |
| chr6_33054414_T_A | 33054414 | 0.14 | 0.10 | 0.19 | 1.36E-33 | 0.23 | 0.06 | 0.28 | SNP |
| chr6_33054231_C_T | 33054231 | 0.14 | 0.10 | 0.19 | 1.45E-33 | 0.23 | 0.06 | 0.28 | SNP |
| chr6_33054280_T_C | 33054280 | 0.14 | 0.10 | 0.20 | 1.85E-33 | 0.23 | 0.06 | 0.28 | SNP |
| chr6_33054281_G_A | 33054281 | 0.14 | 0.10 | 0.20 | 1.85E-33 | 0.23 | 0.06 | 0.28 | SNP |
| chr6_33047432_A_C | 33047432 | 0.15 | 0.11 | 0.21 | 3.66E-33 | 0.23 | 0.06 | 0.28 | SNP |
| chr6_33050125_G_A | 33050125 | 0.14 | 0.10 | 0.19 | 4.64E-33 | 0.22 | 0.05 | 0.27 | SNP |
| chr6_33055047_A_G | 33055047 | 0.15 | 0.10 | 0.20 | 5.67E-33 | 0.23 | 0.06 | 0.28 | SNP |
| chr6_33050118_C_T | 33050118 | 0.14 | 0.10 | 0.19 | 6.11E-33 | 0.22 | 0.05 | 0.27 | SNP |
| chr6_33050148_T_C | 33050148 | 0.14 | 0.10 | 0.19 | 6.99E-33 | 0.22 | 0.05 | 0.27 | SNP |
| chr6_33049211_C_T | 33049211 | 0.15 | 0.11 | 0.21 | 8.70E-33 | 0.23 | 0.06 | 0.28 | SNP |
| chr6_33053167_A_C | 33053167 | 0.15 | 0.11 | 0.20 | 1.22E-32 | 0.23 | 0.06 | 0.28 | SNP |
| chr6_33050078_A_G | 33050078 | 0.15 | 0.11 | 0.20 | 1.43E-32 | 0.22 | 0.06 | 0.27 | SNP |
| chr6_33050089_G_A | 33050089 | 0.15 | 0.11 | 0.20 | 1.48E-32 | 0.22 | 0.06 | 0.27 | SNP |
| chr6_33050079_C_G | 33050079 | 0.15 | 0.11 | 0.20 | 1.50E-32 | 0.22 | 0.06 | 0.27 | SNP |
| chr6_33050107_G_A | 33050107 | 0.15 | 0.11 | 0.20 | 1.58E-32 | 0.22 | 0.06 | 0.27 | SNP |
| chr6_33050499_A_G | 33050499 | 0.14 | 0.10 | 0.19 | 1.79E-32 | 0.23 | 0.05 | 0.27 | SNP |
| chr6_33050473_A_G | 33050473 | 0.15 | 0.10 | 0.20 | 1.98E-32 | 0.22 | 0.05 | 0.27 | SNP |
| chr6_33053270_T_A | 33053270 | 0.13 | 0.09 | 0.18 | 2.37E-32 | 0.23 | 0.05 | 0.28 | SNP |
| chr6_33050024_C_A | 33050024 | 0.15 | 0.11 | 0.20 | 2.73E-32 | 0.23 | 0.06 | 0.27 | SNP |
| chr6_33050168_C_A | 33050168 | 0.15 | 0.11 | 0.20 | 3.10E-32 | 0.22 | 0.06 | 0.27 | SNP |
| chr6_33050045_T_C | 33050045 | 0.15 | 0.11 | 0.20 | 3.23E-32 | 0.22 | 0.06 | 0.27 | SNP |
| chr6_33050279_A_G | 33050279 | 0.15 | 0.11 | 0.21 | 3.26E-32 | 0.23 | 0.06 | 0.27 | SNP |
| chr6_33050179_A_G | 33050179 | 0.15 | 0.11 | 0.20 | 3.48E-32 | 0.22 | 0.06 | 0.27 | SNP |
| chr6_33053271_T_C | 33053271 | 0.13 | 0.09 | 0.18 | 3.81E-32 | 0.23 | 0.05 | 0.28 | SNP |
| chr6_33050185_C_A | 33050185 | 0.15 | 0.11 | 0.20 | 3.97E-32 | 0.22 | 0.06 | 0.27 | SNP |
| chr6_33050186_T_C | 33050186 | 0.15 | 0.11 | 0.20 | 3.97E-32 | 0.22 | 0.06 | 0.27 | SNP |
| chr6_33053307_C_T | 33053307 | 0.14 | 0.10 | 0.19 | 4.24E-32 | 0.23 | 0.05 | 0.28 | SNP |
| chr6_33050525_T_C | 33050525 | 0.14 | 0.10 | 0.20 | 5.34E-32 | 0.22 | 0.05 | 0.27 | SNP |
| chr6_33050203_C_G | 33050203 | 0.16 | 0.11 | 0.21 | 5.97E-32 | 0.22 | 0.06 | 0.27 | SNP |
| chr6_33050526_C_T | 33050526 | 0.14 | 0.10 | 0.20 | 6.29E-32 | 0.22 | 0.05 | 0.27 | SNP |
| chr6_33050196_G_C | 33050196 | 0.16 | 0.11 | 0.21 | 6.36E-32 | 0.22 | 0.06 | 0.27 | SNP |
| chr6_33049979_A_G | 33049979 | 0.16 | 0.12 | 0.21 | 8.29E-32 | 0.23 | 0.06 | 0.27 | SNP |
| chr6_33055123_A_G | 33055123 | 0.14 | 0.10 | 0.20 | 8.49E-32 | 0.23 | 0.05 | 0.28 | SNP |
| chr6_33050521_G_T | 33050521 | 0.15 | 0.10 | 0.20 | 9.38E-32 | 0.22 | 0.05 | 0.27 | SNP |
| chr6_33049983_C_T | 33049983 | 0.16 | 0.12 | 0.21 | 9.52E-32 | 0.23 | 0.06 | 0.27 | SNP |
| chr6_33053248_A_G | 33053248 | 0.13 | 0.09 | 0.18 | 9.54E-32 | 0.23 | 0.05 | 0.28 | SNP |
| chr6_33053252_G_A | 33053252 | 0.13 | 0.09 | 0.18 | 9.83E-32 | 0.23 | 0.05 | 0.28 | SNP |
| chr6_33054659_A_G | 33054659 | 0.15 | 0.11 | 0.21 | 4.33E-31 | 0.22 | 0.06 | 0.26 | SNP |
| chr6_33047031_T_C | 33047031 | 0.16 | 0.11 | 0.21 | 4.91E-31 | 0.22 | 0.06 | 0.27 | SNP |
| chr6_33055079_A_G | 33055079 | 0.16 | 0.11 | 0.21 | 4.61E-30 | 0.22 | 0.06 | 0.26 | SNP |
| chr6_33050232_T_G | 33050232 | 0.17 | 0.12 | 0.22 | 3.52E-29 | 0.21 | 0.06 | 0.25 | SNP |
| chr6_33050223_G_A | 33050223 | 0.17 | 0.12 | 0.23 | 5.44E-29 | 0.21 | 0.06 | 0.25 | SNP |
| chr6_33048459_T_G | 33048459 | 0.16 | 0.11 | 0.22 | 1.23E-28 | 0.19 | 0.05 | 0.23 | SNP |
| chr6_33048461_T_A | 33048461 | 0.16 | 0.11 | 0.22 | 1.46E-28 | 0.19 | 0.05 | 0.23 | SNP |
| chr6_33048457_C_G | 33048457 | 0.16 | 0.11 | 0.22 | 2.03E-28 | 0.19 | 0.05 | 0.23 | SNP |
| chr6_33071754_C_A | 33071754 | 0.15 | 0.10 | 0.21 | 1.10E-26 | 0.19 | 0.05 | 0.23 | SNP |
| chr6_33039625_T_C | 33039625 | 0.24 | 0.18 | 0.30 | 1.63E-26 | 0.24 | 0.08 | 0.28 | SNP |
| chr6_33053396_A_G | 33053396 | 0.14 | 0.10 | 0.20 | 2.92E-26 | 0.18 | 0.04 | 0.22 | SNP |
| chr6_33054861_A_G | 33054861 | 0.15 | 0.11 | 0.21 | 3.58E-26 | 0.18 | 0.04 | 0.22 | SNP |
| chr6_33071777_A_G | 33071777 | 0.15 | 0.10 | 0.21 | 3.65E-26 | 0.19 | 0.05 | 0.23 | SNP |
| chr6_33053942_T_C | 33053942 | 0.15 | 0.11 | 0.21 | 4.00E-26 | 0.18 | 0.04 | 0.22 | SNP |
| chr6_33053812_A_G | 33053812 | 0.15 | 0.11 | 0.21 | 4.17E-26 | 0.18 | 0.04 | 0.22 | SNP |
| chr6_33071708_G_A | 33071708 | 0.15 | 0.11 | 0.22 | 4.93E-26 | 0.19 | 0.05 | 0.23 | SNP |
| chr6_33071600_C_A | 33071600 | 0.16 | 0.11 | 0.22 | 1.03E-25 | 0.19 | 0.05 | 0.23 | SNP |
| chr6_33053352_C_T | 33053352 | 0.15 | 0.11 | 0.22 | 1.36E-25 | 0.18 | 0.05 | 0.22 | SNP |
| chr6_33047466_T_C | 33047466 | 0.17 | 0.12 | 0.23 | 7.57E-24 | 0.17 | 0.04 | 0.20 | SNP |
| chr6_33054074_C_T | 33054074 | 0.17 | 0.11 | 0.23 | 1.12E-23 | 0.16 | 0.04 | 0.20 | SNP |
| chr6_33048542_C_T | 33048542 | 0.38 | 0.32 | 0.46 | 3.80E-23 | 0.41 | 0.24 | 0.46 | SNP |
| chr6_33143756_A_G | 33143756 | 0.23 | 0.17 | 0.31 | 4.85E-22 | 0.19 | 0.07 | 0.22 | SNP |
| chr6_33166034_A_G | 33166034 | 0.22 | 0.16 | 0.30 | 3.85E-21 | 0.19 | 0.07 | 0.22 | SNP |
| chr6_33160425_G_T | 33160425 | 0.23 | 0.17 | 0.31 | 6.33E-21 | 0.17 | 0.06 | 0.20 | SNP |
| chr6_33048466_G_T | 33048466 | 0.17 | 0.12 | 0.25 | 1.72E-20 | 0.14 | 0.04 | 0.17 | SNP |
| chr6_33048467_G_T | 33048467 | 0.17 | 0.12 | 0.25 | 2.04E-20 | 0.14 | 0.04 | 0.17 | SNP |
| chr6_33046742_T_C | 33046742 | 0.18 | 0.12 | 0.25 | 2.37E-20 | 0.15 | 0.04 | 0.18 | SNP |
| chr6_33178010_G_T | 33178010 | 0.25 | 0.18 | 0.33 | 3.03E-20 | 0.19 | 0.07 | 0.22 | SNP |
| chr6_33048663_G_A | 33048663 | 0.19 | 0.13 | 0.27 | 1.52E-19 | 0.18 | 0.05 | 0.21 | SNP |
| chr6_33048599_C_A | 33048599 | 0.38 | 0.31 | 0.47 | 2.17E-19 | 0.40 | 0.24 | 0.44 | SNP |
| chr6_33050144_C_T | 33050144 | 0.17 | 0.11 | 0.24 | 3.45E-19 | 0.13 | 0.03 | 0.16 | SNP |
| chr6_33050394_G_A | 33050394 | 0.17 | 0.11 | 0.25 | 3.85E-19 | 0.14 | 0.03 | 0.16 | SNP |
| chr6_33045208_T_C | 33045208 | 0.41 | 0.34 | 0.50 | 4.49E-19 | 0.36 | 0.20 | 0.40 | SNP |
| chr6_33050463_C_G | 33050463 | 0.17 | 0.11 | 0.25 | 4.75E-19 | 0.13 | 0.03 | 0.16 | SNP |
| chr6_33174783_A_G | 33174783 | 0.33 | 0.26 | 0.42 | 8.66E-19 | 0.22 | 0.11 | 0.25 | SNP |
| chr6_33050084_C_T | 33050084 | 0.18 | 0.12 | 0.26 | 9.22E-19 | 0.13 | 0.04 | 0.16 | SNP |
| chr6_33050325_A_C | 33050325 | 0.18 | 0.12 | 0.26 | 1.17E-18 | 0.13 | 0.04 | 0.16 | SNP |
| chr6_33054145_A_G | 33054145 | 0.18 | 0.12 | 0.26 | 1.44E-18 | 0.14 | 0.04 | 0.16 | SNP |
| chr6_33050036_T_C | 33050036 | 0.19 | 0.13 | 0.27 | 1.99E-18 | 0.13 | 0.04 | 0.16 | SNP |
| chr6_33045255_T_C | 33045255 | 0.43 | 0.35 | 0.51 | 2.84E-18 | 0.36 | 0.20 | 0.40 | SNP |
| chr6_33037419_G_A | 33037419 | 0.13 | 0.08 | 0.20 | 3.22E-18 | 0.12 | 0.02 | 0.14 | SNP |
| chr6_33037424_T_C | 33037424 | 0.13 | 0.08 | 0.20 | 3.44E-18 | 0.12 | 0.02 | 0.14 | SNP |
| chr6_33095615_T_C | 33095615 | 0.41 | 0.33 | 0.50 | 4.79E-18 | 0.32 | 0.20 | 0.36 | SNP |
| chr6_33046915_T_A | 33046915 | 0.19 | 0.13 | 0.27 | 7.16E-18 | 0.13 | 0.04 | 0.16 | SNP |
| chr6_33037304_G_T | 33037304 | 0.13 | 0.08 | 0.21 | 7.80E-18 | 0.12 | 0.02 | 0.15 | SNP |
| chr6_33037279_A_G | 33037279 | 0.14 | 0.08 | 0.21 | 8.08E-18 | 0.12 | 0.02 | 0.15 | SNP |
| chr6_33046726_T_C | 33046726 | 0.19 | 0.13 | 0.27 | 8.20E-18 | 0.13 | 0.04 | 0.16 | SNP |
| chr6_33033058_T_C | 33033058 | 0.14 | 0.08 | 0.21 | 9.68E-18 | 0.12 | 0.02 | 0.15 | SNP |
| chr6_33037294_T_C | 33037294 | 0.13 | 0.08 | 0.21 | 9.74E-18 | 0.12 | 0.02 | 0.15 | SNP |
| chr6_33037187_C_G | 33037187 | 0.14 | 0.09 | 0.22 | 9.87E-18 | 0.13 | 0.03 | 0.15 | SNP |
| chr6_33036312_A_G | 33036312 | 0.14 | 0.09 | 0.22 | 1.22E-17 | 0.12 | 0.03 | 0.15 | SNP |
| chr6_33036287_G_A | 33036287 | 0.14 | 0.09 | 0.22 | 1.24E-17 | 0.12 | 0.03 | 0.15 | SNP |
| chr6_33036505_G_C | 33036505 | 0.14 | 0.09 | 0.22 | 1.33E-17 | 0.13 | 0.03 | 0.15 | SNP |
| chr6_33036951_A_G | 33036951 | 0.14 | 0.09 | 0.22 | 1.38E-17 | 0.12 | 0.03 | 0.14 | SNP |
| chr6_33036242_T_C | 33036242 | 0.14 | 0.09 | 0.22 | 1.40E-17 | 0.12 | 0.03 | 0.15 | SNP |
| chr6_33036288_G_A | 33036288 | 0.14 | 0.09 | 0.22 | 1.41E-17 | 0.12 | 0.03 | 0.15 | SNP |

| | | | | | | | | | |
|---|---|---|---|---|---|---|---|---|---|
| chr6_33036617_C_T | 33036617 | 0.14 | 0.09 | 0.22 | 1.41E-17 | 0.13 | 0.03 | 0.15 | SNP |
| chr6_33036233_A_G | 33036233 | 0.14 | 0.09 | 0.22 | 1.43E-17 | 0.12 | 0.03 | 0.15 | SNP |
| chr6_33036630_G_T | 33036630 | 0.14 | 0.09 | 0.22 | 1.44E-17 | 0.12 | 0.03 | 0.15 | SNP |
| chr6_33036631_A_C | 33036631 | 0.14 | 0.09 | 0.22 | 1.44E-17 | 0.12 | 0.03 | 0.15 | SNP |
| chr6_33036959_G_A | 33036959 | 0.14 | 0.09 | 0.22 | 1.45E-17 | 0.12 | 0.03 | 0.14 | SNP |
| chr6_33036177_A_G | 33036177 | 0.14 | 0.09 | 0.22 | 1.48E-17 | 0.12 | 0.03 | 0.15 | SNP |
| chr6_33037522_T_C | 33037522 | 0.14 | 0.09 | 0.21 | 1.49E-17 | 0.12 | 0.02 | 0.15 | SNP |
| chr6_33040715_G_A | 33040715 | 0.13 | 0.08 | 0.20 | 1.49E-17 | 0.12 | 0.02 | 0.15 | SNP |
| chr6_33037675_C_A | 33037675 | 0.14 | 0.09 | 0.21 | 1.51E-17 | 0.12 | 0.02 | 0.15 | SNP |
| chr6_33036210_G_T | 33036210 | 0.14 | 0.09 | 0.22 | 1.52E-17 | 0.12 | 0.03 | 0.15 | SNP |
| chr6_33033202_C_T | 33033202 | 0.14 | 0.09 | 0.22 | 1.52E-17 | 0.12 | 0.03 | 0.15 | SNP |
| chr6_33040736_T_C | 33040736 | 0.13 | 0.08 | 0.20 | 1.52E-17 | 0.12 | 0.02 | 0.15 | SNP |
| chr6_33033170_T_C | 33033170 | 0.14 | 0.09 | 0.22 | 1.53E-17 | 0.12 | 0.03 | 0.15 | SNP |
| chr6_33037690_C_T | 33037690 | 0.14 | 0.09 | 0.21 | 1.57E-17 | 0.12 | 0.02 | 0.15 | SNP |
| chr6_33036401_C_T | 33036401 | 0.15 | 0.09 | 0.23 | 1.60E-17 | 0.12 | 0.03 | 0.15 | SNP |
| chr6_33037579_A_T | 33037579 | 0.14 | 0.09 | 0.21 | 1.61E-17 | 0.12 | 0.02 | 0.15 | SNP |
| chr6_33037580_T_G | 33037580 | 0.14 | 0.09 | 0.21 | 1.61E-17 | 0.12 | 0.02 | 0.15 | SNP |
| chr6_33033235_G_C | 33033235 | 0.14 | 0.09 | 0.22 | 1.64E-17 | 0.12 | 0.03 | 0.15 | SNP |
| chr6_33036717_C_G | 33036717 | 0.14 | 0.09 | 0.22 | 1.64E-17 | 0.12 | 0.03 | 0.15 | SNP |
| chr6_33036394_G_A | 33036394 | 0.14 | 0.09 | 0.22 | 1.66E-17 | 0.12 | 0.03 | 0.15 | SNP |
| chr6_33047612_G_C | 33047612 | 0.19 | 0.13 | 0.28 | 1.68E-17 | 0.13 | 0.04 | 0.15 | SNP |
| chr6_33033146_T_C | 33033146 | 0.14 | 0.09 | 0.22 | 1.68E-17 | 0.12 | 0.03 | 0.15 | SNP |
| chr6_33037142_A_G | 33037142 | 0.14 | 0.09 | 0.22 | 1.69E-17 | 0.13 | 0.03 | 0.15 | SNP |
| chr6_33033188_C_T | 33033188 | 0.14 | 0.09 | 0.22 | 1.78E-17 | 0.12 | 0.03 | 0.15 | SNP |
| chr6_33036388_C_T | 33036388 | 0.14 | 0.09 | 0.22 | 1.79E-17 | 0.12 | 0.03 | 0.15 | SNP |
| chr6_33033178_G_A | 33033178 | 0.14 | 0.09 | 0.22 | 1.80E-17 | 0.12 | 0.03 | 0.15 | SNP |
| chr6_33036853_A_C | 33036853 | 0.14 | 0.09 | 0.22 | 1.80E-17 | 0.12 | 0.03 | 0.15 | SNP |
| chr6_33033177_G_A | 33033177 | 0.14 | 0.09 | 0.22 | 1.87E-17 | 0.12 | 0.03 | 0.15 | SNP |
| chr6_33036971_C_T | 33036971 | 0.14 | 0.09 | 0.22 | 1.92E-17 | 0.12 | 0.03 | 0.15 | SNP |
| chr6_33036999_T_C | 33036999 | 0.14 | 0.09 | 0.22 | 1.92E-17 | 0.12 | 0.03 | 0.15 | SNP |
| chr6_33036708_C_T | 33036708 | 0.14 | 0.09 | 0.22 | 1.94E-17 | 0.12 | 0.03 | 0.15 | SNP |
| chr6_33036709_A_G | 33036709 | 0.14 | 0.09 | 0.22 | 1.94E-17 | 0.12 | 0.03 | 0.15 | SNP |
| chr6_33037080_G_A | 33037080 | 0.14 | 0.09 | 0.22 | 1.99E-17 | 0.12 | 0.03 | 0.15 | SNP |
| chr6_33037082_A_G | 33037082 | 0.14 | 0.09 | 0.22 | 1.99E-17 | 0.12 | 0.03 | 0.15 | SNP |
| chr6_33039484_A_G | 33039484 | 0.13 | 0.08 | 0.20 | 2.02E-17 | 0.12 | 0.02 | 0.15 | SNP |
| chr6_33037104_C_T | 33037104 | 0.14 | 0.09 | 0.22 | 2.04E-17 | 0.12 | 0.03 | 0.15 | SNP |
| chr6_33095801_C_T | 33095801 | 0.41 | 0.34 | 0.51 | 2.05E-17 | 0.32 | 0.20 | 0.35 | SNP |
| chr6_33040596_A_G | 33040596 | 0.14 | 0.09 | 0.21 | 2.16E-17 | 0.12 | 0.02 | 0.15 | SNP |
| chr6_33036132_A_G | 33036132 | 0.14 | 0.09 | 0.22 | 2.16E-17 | 0.12 | 0.03 | 0.15 | SNP |
| chr6_33036133_G_A | 33036133 | 0.14 | 0.09 | 0.22 | 2.16E-17 | 0.12 | 0.03 | 0.15 | SNP |
| chr6_33040781_A_G | 33040781 | 0.14 | 0.09 | 0.21 | 2.18E-17 | 0.12 | 0.02 | 0.15 | SNP |
| chr6_33040798_T_C | 33040798 | 0.14 | 0.09 | 0.21 | 2.18E-17 | 0.12 | 0.02 | 0.15 | SNP |
| chr6_33040835_A_G | 33040835 | 0.14 | 0.09 | 0.21 | 2.18E-17 | 0.12 | 0.02 | 0.15 | SNP |
| chr6_33040877_T_C | 33040877 | 0.14 | 0.09 | 0.21 | 2.18E-17 | 0.12 | 0.02 | 0.15 | SNP |
| chr6_33041034_A_G | 33041034 | 0.14 | 0.09 | 0.21 | 2.18E-17 | 0.12 | 0.02 | 0.15 | SNP |
| chr6_33041226_C_T | 33041226 | 0.14 | 0.09 | 0.21 | 2.18E-17 | 0.12 | 0.02 | 0.15 | SNP |
| chr6_33040744_G_C | 33040744 | 0.14 | 0.09 | 0.21 | 2.19E-17 | 0.12 | 0.02 | 0.15 | SNP |
| chr6_33032822_G_A | 33032822 | 0.14 | 0.09 | 0.22 | 2.21E-17 | 0.12 | 0.03 | 0.15 | SNP |
| chr6_33039606_C_T | 33039606 | 0.14 | 0.09 | 0.21 | 2.24E-17 | 0.12 | 0.02 | 0.15 | SNP |
| chr6_33039405_C_G | 33039405 | 0.13 | 0.08 | 0.21 | 2.24E-17 | 0.12 | 0.02 | 0.15 | SNP |
| chr6_33039980_A_G | 33039980 | 0.14 | 0.09 | 0.21 | 2.26E-17 | 0.12 | 0.02 | 0.15 | SNP |
| chr6_33032865_A_C | 33032865 | 0.14 | 0.09 | 0.22 | 2.38E-17 | 0.12 | 0.03 | 0.15 | SNP |
| chr6_33043930_A_G | 33043930 | 0.14 | 0.09 | 0.21 | 2.42E-17 | 0.12 | 0.02 | 0.15 | SNP |
| chr6_33039503_C_T | 33039503 | 0.13 | 0.08 | 0.20 | 2.47E-17 | 0.12 | 0.02 | 0.15 | SNP |
| chr6_33041176_C_T | 33041176 | 0.13 | 0.08 | 0.21 | 2.65E-17 | 0.12 | 0.02 | 0.15 | SNP |
| chr6_33039837_G_A | 33039837 | 0.14 | 0.09 | 0.22 | 2.80E-17 | 0.12 | 0.02 | 0.15 | SNP |
| chr6_33036085_C_G | 33036085 | 0.15 | 0.09 | 0.22 | 2.81E-17 | 0.12 | 0.03 | 0.15 | SNP |
| chr6_33039767_A_G | 33039767 | 0.14 | 0.09 | 0.22 | 2.82E-17 | 0.12 | 0.02 | 0.15 | SNP |
| chr6_33033266_T_A | 33033266 | 0.15 | 0.09 | 0.22 | 2.89E-17 | 0.12 | 0.03 | 0.15 | SNP |
| chr6_33036116_T_G | 33036116 | 0.15 | 0.09 | 0.22 | 2.94E-17 | 0.12 | 0.03 | 0.15 | SNP |
| chr6_33037768_G_A | 33037768 | 0.14 | 0.09 | 0.22 | 2.97E-17 | 0.12 | 0.02 | 0.15 | SNP |
| chr6_33095636_A_G | 33095636 | 0.42 | 0.35 | 0.52 | 3.04E-17 | 0.33 | 0.20 | 0.36 | SNP |
| chr6_33039686_T_C | 33039686 | 0.14 | 0.09 | 0.22 | 3.12E-17 | 0.12 | 0.02 | 0.15 | SNP |
| chr6_33033287_G_C | 33033287 | 0.15 | 0.09 | 0.22 | 3.27E-17 | 0.12 | 0.03 | 0.15 | SNP |
| chr6_33039719_A_G | 33039719 | 0.14 | 0.09 | 0.22 | 3.49E-17 | 0.12 | 0.02 | 0.15 | SNP |
| chr6_33039295_T_A | 33039295 | 0.14 | 0.09 | 0.22 | 3.63E-17 | 0.12 | 0.02 | 0.15 | SNP |
| chr6_33033267_G_A | 33033267 | 0.15 | 0.09 | 0.22 | 3.76E-17 | 0.12 | 0.03 | 0.15 | SNP |
| chr6_33095836_G_A | 33095836 | 0.42 | 0.34 | 0.51 | 3.77E-17 | 0.32 | 0.20 | 0.35 | SNP |
| chr6_33039729_T_G | 33039729 | 0.14 | 0.09 | 0.22 | 4.07E-17 | 0.12 | 0.02 | 0.15 | SNP |
| chr6_33041186_G_A | 33041186 | 0.14 | 0.08 | 0.21 | 4.90E-17 | 0.12 | 0.02 | 0.15 | SNP |
| chr6_33048661_A_G | 33048661 | 0.21 | 0.14 | 0.30 | 6.26E-17 | 0.16 | 0.05 | 0.19 | SNP |
| chr6_33095684_A_G | 33095684 | 0.43 | 0.35 | 0.53 | 8.66E-17 | 0.33 | 0.21 | 0.36 | SNP |
| chr6_33095737_G_A | 33095737 | 0.44 | 0.36 | 0.53 | 1.90E-16 | 0.32 | 0.20 | 0.36 | SNP |
| chr6_33033351_G_T | 33033351 | 0.15 | 0.09 | 0.22 | 2.05E-16 | 0.13 | 0.03 | 0.15 | SNP |
| chr6_33044257_T_C | 33044257 | 0.15 | 0.09 | 0.23 | 2.20E-16 | 0.12 | 0.02 | 0.14 | SNP |
| chr6_33033357_C_G | 33033357 | 0.14 | 0.09 | 0.22 | 3.32E-16 | 0.12 | 0.03 | 0.15 | SNP |
| chr6_33037866_A_G | 33037866 | 0.14 | 0.08 | 0.21 | 3.49E-16 | 0.12 | 0.02 | 0.14 | SNP |
| chr6_33037865_C_T | 33037865 | 0.14 | 0.08 | 0.21 | 3.68E-16 | 0.12 | 0.02 | 0.14 | SNP |
| chr6_33048640_A_G | 33048640 | 0.27 | 0.20 | 0.37 | 5.62E-16 | 0.18 | 0.07 | 0.21 | SNP |
| chr6_33095748_C_G | 33095748 | 0.44 | 0.36 | 0.54 | 7.56E-16 | 0.32 | 0.21 | 0.35 | SNP |
| chr6_33036003_G_C | 33036003 | 0.13 | 0.08 | 0.21 | 2.11E-15 | 0.11 | 0.02 | 0.13 | SNP |
| chr6_33035986_T_C | 33035986 | 0.13 | 0.08 | 0.21 | 2.76E-15 | 0.11 | 0.02 | 0.13 | SNP |
| chr6_33048602_C_A | 33048602 | 0.43 | 0.34 | 0.53 | 2.95E-15 | 0.37 | 0.23 | 0.40 | SNP |
| chr6_33037878_G_A | 33037878 | 0.14 | 0.08 | 0.22 | 3.50E-15 | 0.12 | 0.02 | 0.14 | SNP |
| chr6_33035980_T_A | 33035980 | 0.13 | 0.08 | 0.21 | 3.72E-15 | 0.11 | 0.02 | 0.13 | SNP |
| chr6_33037560_G_A | 33037560 | 0.11 | 0.06 | 0.18 | 3.78E-15 | 0.10 | 0.02 | 0.12 | SNP |
| chr6_33095516_G_A | 33095516 | 0.43 | 0.34 | 0.53 | 4.81E-15 | 0.32 | 0.20 | 0.36 | SNP |
| chr6_33035982_G_A | 33035982 | 0.13 | 0.08 | 0.22 | 5.18E-15 | 0.11 | 0.02 | 0.13 | SNP |
| chr6_33033313_G_T | 33033313 | 0.12 | 0.07 | 0.20 | 6.74E-15 | 0.10 | 0.02 | 0.12 | SNP |
| chr6_33045823_A_G | 33045823 | 0.13 | 0.07 | 0.21 | 7.49E-15 | 0.10 | 0.02 | 0.13 | SNP |
| chr6_33033388_A_C | 33033388 | 0.13 | 0.07 | 0.21 | 8.35E-15 | 0.13 | 0.02 | 0.15 | SNP |
| chr6_33141161_G_A | 33141161 | 1.95 | 1.65 | 2.30 | 8.66E-15 | 0.35 | 0.48 | 0.32 | SNP |
| chr6_33033390_A_T | 33033390 | 0.11 | 0.06 | 0.19 | 9.47E-15 | 0.13 | 0.02 | 0.15 | SNP |
| chr6_33142162_C_A | 33142162 | 0.28 | 0.20 | 0.38 | 1.22E-14 | 0.14 | 0.05 | 0.16 | SNP |
| chr6_33035974_A_G | 33035974 | 0.14 | 0.08 | 0.22 | 1.27E-14 | 0.11 | 0.02 | 0.13 | SNP |
| chr6_33037891_C_T | 33037891 | 0.12 | 0.07 | 0.20 | 2.05E-14 | 0.11 | 0.02 | 0.14 | SNP |
| chr6_33145710_G_A | 33145710 | 1.90 | 1.61 | 2.25 | 2.64E-14 | 0.35 | 0.48 | 0.32 | SNP |
| chr6_33037557_C_T | 33037557 | 0.12 | 0.06 | 0.19 | 2.83E-14 | 0.09 | 0.02 | 0.11 | SNP |
| chr6_33035969_A_G | 33035969 | 0.14 | 0.08 | 0.23 | 5.16E-14 | 0.10 | 0.02 | 0.13 | SNP |
| chr6_33044258_G_A | 33044258 | 0.12 | 0.07 | 0.20 | 5.21E-14 | 0.09 | 0.02 | 0.11 | SNP |
| chr6_33158189_C_A | 33158189 | 1.91 | 1.62 | 2.27 | 5.23E-14 | 0.36 | 0.48 | 0.32 | SNP |
| chr6_33044388_T_C | 33044388 | 0.12 | 0.07 | 0.20 | 5.83E-14 | 0.09 | 0.02 | 0.11 | SNP |
| chr6_33042368_A_G | 33042368 | 0.12 | 0.07 | 0.20 | 5.85E-14 | 0.09 | 0.02 | 0.11 | SNP |
| chr6_33042406_C_G | 33042406 | 0.12 | 0.07 | 0.20 | 6.04E-14 | 0.09 | 0.02 | 0.11 | SNP |
| chr6_33039694_G_T | 33039694 | 0.12 | 0.07 | 0.20 | 6.06E-14 | 0.09 | 0.02 | 0.11 | SNP |
| chr6_33140983_G_A | 33140983 | 1.91 | 1.62 | 2.27 | 6.08E-14 | 0.36 | 0.48 | 0.32 | SNP |
| chr6_33040654_A_G | 33040654 | 0.12 | 0.07 | 0.20 | 6.09E-14 | 0.09 | 0.02 | 0.11 | SNP |
| chr6_33042492_T_C | 33042492 | 0.12 | 0.07 | 0.20 | 6.09E-14 | 0.09 | 0.02 | 0.11 | SNP |
| chr6_33042880_A_G | 33042880 | 0.12 | 0.07 | 0.20 | 6.09E-14 | 0.09 | 0.02 | 0.11 | SNP |
| chr6_33044188_T_A | 33044188 | 0.12 | 0.07 | 0.20 | 6.18E-14 | 0.09 | 0.02 | 0.11 | SNP |
| chr6_33040044_A_G | 33040044 | 0.12 | 0.07 | 0.20 | 6.29E-14 | 0.09 | 0.02 | 0.11 | SNP |
| chr6_33039681_A_G | 33039681 | 0.12 | 0.07 | 0.20 | 6.33E-14 | 0.09 | 0.02 | 0.11 | SNP |
| chr6_33039697_T_G | 33039697 | 0.12 | 0.07 | 0.20 | 6.33E-14 | 0.09 | 0.02 | 0.11 | SNP |
| chr6_33048921_A_G | 33048921 | 0.15 | 0.09 | 0.24 | 6.46E-14 | 0.10 | 0.02 | 0.12 | SNP |
| chr6_33141280_G_A | 33141280 | 0.28 | 0.19 | 0.38 | 6.55E-14 | 0.14 | 0.05 | 0.16 | SNP |
| chr6_33137403_T_C | 33137403 | 1.89 | 1.60 | 2.23 | 7.01E-14 | 0.36 | 0.48 | 0.32 | SNP |
| chr6_33041073_A_C | 33041073 | 0.15 | 0.09 | 0.24 | 7.05E-14 | 0.10 | 0.02 | 0.12 | SNP |
| chr6_33095762_G_A | 33095762 | 0.21 | 0.13 | 0.31 | 7.30E-14 | 0.10 | 0.03 | 0.12 | SNP |
| chr6_33154656_G_A | 33154656 | 0.27 | 0.19 | 0.38 | 8.36E-14 | 0.13 | 0.05 | 0.15 | SNP |
| chr6_33048628_A_C | 33048628 | 0.23 | 0.15 | 0.33 | 9.05E-14 | 0.13 | 0.04 | 0.15 | SNP |
| chr6_33146053_C_G | 33146053 | 0.27 | 0.19 | 0.38 | 1.02E-13 | 0.12 | 0.05 | 0.15 | SNP |
| chr6_33148878_C_T | 33148878 | 0.27 | 0.19 | 0.38 | 1.06E-13 | 0.12 | 0.05 | 0.15 | SNP |
| chr6_33048606_G_C | 33048606 | 0.23 | 0.15 | 0.33 | 1.16E-13 | 0.12 | 0.04 | 0.14 | SNP |
| chr6_33142253_G_C | 33142253 | 1.88 | 1.59 | 2.23 | 1.41E-13 | 0.36 | 0.48 | 0.33 | SNP |
| chr6_33095555_T_A | 33095555 | 0.21 | 0.13 | 0.31 | 1.72E-13 | 0.10 | 0.03 | 0.12 | SNP |
| chr6_33048102_C_T | 33048102 | 0.22 | 0.14 | 0.32 | 2.39E-13 | 0.10 | 0.03 | 0.12 | SNP |
| chr6_33162215_C_A | 33162215 | 1.85 | 1.57 | 2.18 | 3.21E-13 | 0.36 | 0.48 | 0.33 | SNP |
| chr6_33161661_G_A | 33161661 | 1.85 | 1.56 | 2.18 | 4.06E-13 | 0.36 | 0.48 | 0.33 | SNP |
| chr6_33135392_G_A | 33135392 | 2.03 | 1.68 | 2.47 | 4.22E-13 | 0.38 | 0.50 | 0.34 | SNP |
| chr6_33144373_A_G | 33144373 | 1.84 | 1.56 | 2.17 | 4.38E-13 | 0.36 | 0.48 | 0.33 | SNP |
| chr6_33040478_C_A | 33040478 | 0.13 | 0.07 | 0.22 | 4.93E-13 | 0.09 | 0.02 | 0.11 | SNP |
| chr6_33141920_C_G | 33141920 | 1.84 | 1.56 | 2.17 | 5.40E-13 | 0.36 | 0.48 | 0.33 | SNP |
| chr6_33139662_G_A | 33139662 | 1.83 | 1.56 | 2.17 | 5.81E-13 | 0.36 | 0.48 | 0.33 | SNP |
| chr6_33048380_G_A | 33048380 | 0.24 | 0.16 | 0.35 | 6.92E-13 | 0.10 | 0.03 | 0.12 | SNP |
| chr6_33046752_G_C | 33046752 | 0.24 | 0.16 | 0.35 | 7.31E-13 | 0.10 | 0.03 | 0.12 | SNP |
| chr6_33139475_G_T | 33139475 | 1.87 | 1.58 | 2.23 | 7.62E-13 | 0.35 | 0.48 | 0.32 | SNP |
| chr6_33163451_A_G | 33163451 | 1.87 | 1.58 | 2.22 | 7.80E-13 | 0.36 | 0.47 | 0.33 | SNP |
| chr6_33041064_C_A | 33041064 | 0.24 | 0.16 | 0.35 | 1.01E-12 | 0.10 | 0.03 | 0.12 | SNP |
| chr6_33149152_C_G | 33149152 | 0.28 | 0.20 | 0.40 | 1.02E-12 | 0.12 | 0.05 | 0.15 | SNP |
| chr6_33138839_A_T | 33138839 | 0.26 | 0.18 | 0.38 | 1.06E-12 | 0.13 | 0.05 | 0.15 | SNP |
| chr6_33143136_G_A | 33143136 | 0.28 | 0.19 | 0.39 | 1.53E-12 | 0.14 | 0.05 | 0.16 | SNP |
| chr6_33138498_C_T | 33138498 | 1.84 | 1.56 | 2.18 | 1.54E-12 | 0.37 | 0.49 | 0.34 | SNP |
| chr6_33048937_C_G | 33048937 | 0.14 | 0.08 | 0.24 | 4.76E-12 | 0.08 | 0.02 | 0.10 | SNP |
| chr6_33138955_A_G | 33138955 | 1.77 | 1.50 | 2.09 | 9.01E-12 | 0.37 | 0.48 | 0.34 | SNP |
| chr6_33048348_G_A | 33048348 | 0.16 | 0.09 | 0.27 | 3.44E-11 | 0.08 | 0.02 | 0.09 | SNP |
| chr6_33143948_C_G | 33143948 | 0.55 | 0.46 | 0.65 | 5.26E-11 | 0.34 | 0.23 | 0.37 | SNP |
| chr6_33045147_A_T | 33045147 | 0.50 | 0.40 | 0.61 | 6.09E-11 | 0.30 | 0.19 | 0.33 | SNP |
| chr6_33144243_T_A | 33144243 | 0.55 | 0.46 | 0.66 | 7.08E-11 | 0.34 | 0.23 | 0.37 | SNP |
| chr6_33048539_T_A | 33048539 | 0.13 | 0.06 | 0.23 | 1.34E-10 | 0.07 | 0.01 | 0.09 | SNP |
| chr6_33045500_T_C | 33045500 | 0.17 | 0.10 | 0.29 | 1.79E-10 | 0.08 | 0.02 | 0.09 | SNP |
| chr6_33036290_G_A | 33036290 | 1.84 | 1.52 | 2.22 | 2.74E-10 | 0.18 | 0.27 | 0.16 | SNP |
| chr6_33049726_G_T | 33049726 | 0.13 | 0.07 | 0.24 | 3.50E-10 | 0.07 | 0.01 | 0.08 | SNP |
| chr6_33049727_A_T | 33049727 | 0.13 | 0.07 | 0.24 | 3.50E-10 | 0.07 | 0.01 | 0.08 | SNP |
| chr6_33047898_C_A | 33047898 | 0.18 | 0.10 | 0.30 | 9.34E-10 | 0.07 | 0.02 | 0.08 | SNP |
| chr6_33136638_A_G | 33136638 | 1.67 | 1.42 | 1.98 | 1.44E-09 | 0.36 | 0.46 | 0.34 | SNP |
| chr6_33139328_C_T | 33139328 | 0.60 | 0.50 | 0.71 | 7.99E-09 | 0.43 | 0.34 | 0.46 | SNP |
| chr6_33049965_G_A | 33049965 | 0.14 | 0.07 | 0.27 | 3.99E-08 | 0.05 | 0.01 | 0.06 | SNP |
| chr6_33043520_C_G | 33043520 | 0.41 | 0.30 | 0.56 | 4.78E-08 | 0.12 | 0.06 | 0.13 | SNP |
| chr6_33037199_G_C | 33037199 | 0.42 | 0.30 | 0.57 | 5.80E-08 | 0.12 | 0.06 | 0.13 | SNP |
| chr6_33032851_T_C | 33032851 | 0.42 | 0.30 | 0.57 | 5.88E-08 | 0.12 | 0.06 | 0.13 | SNP |
| chr6_33049902_T_A | 33049902 | 0.20 | 0.10 | 0.34 | 6.02E-08 | 0.06 | 0.01 | 0.07 | SNP |
| chr6_33050178_C_T | 33050178 | 0.20 | 0.10 | 0.34 | 6.02E-08 | 0.06 | 0.01 | 0.07 | SNP |

| | | | | | | | | | |
|---|---|---|---|---|---|---|---|---|---|
| chr6_33048975_G_A | 33048975 | 0.16 | 0.08 | 0.30 | 7.33E-08 | 0.05 | 0.01 | 0.06 | SNP |
| chr6_32978587_C_A | 32978587 | 0.25 | 0.14 | 0.40 | 7.74E-08 | 0.06 | 0.02 | 0.07 | SNP |
| chr6_33053577_G_A | 33053577 | 0.16 | 0.08 | 0.30 | 7.86E-08 | 0.05 | 0.01 | 0.06 | SNP |
| chr6_33054378_G_A | 33054378 | 0.16 | 0.08 | 0.30 | 7.90E-08 | 0.05 | 0.01 | 0.06 | SNP |
| chr6_33054559_T_C | 33054559 | 0.16 | 0.08 | 0.30 | 7.90E-08 | 0.05 | 0.01 | 0.06 | SNP |
| chr6_33134392_A_C | 33134392 | 1.57 | 1.33 | 1.85 | 7.92E-08 | 0.40 | 0.49 | 0.38 | SNP |
| chr6_33048354_A_T | 33048354 | 0.18 | 0.09 | 0.32 | 8.72E-08 | 0.05 | 0.01 | 0.06 | SNP |
| chr6_33047173_T_C | 33047173 | 0.18 | 0.09 | 0.32 | 9.06E-08 | 0.05 | 0.01 | 0.06 | SNP |
| chr6_33033274_T_C | 33033274 | 0.42 | 0.30 | 0.57 | 9.55E-08 | 0.12 | 0.06 | 0.13 | SNP |
| chr6_33041734_T_C | 33041734 | 0.42 | 0.30 | 0.57 | 1.20E-07 | 0.11 | 0.05 | 0.13 | SNP |
| xHLA_2field_DPB1_01_01 | 33043767 | 0.15 | 0.07 | 0.29 | 1.33E-07 | 0.05 | 0.01 | 0.06 | HLA (xHLA) |
| chr6_33045699_T_C | 33045699 | 0.15 | 0.07 | 0.29 | 1.41E-07 | 0.05 | 0.01 | 0.06 | SNP |
| chr6_33050083_C_A | 33050083 | 0.15 | 0.07 | 0.29 | 1.41E-07 | 0.05 | 0.01 | 0.06 | SNP |
| chr6_32555317_C_T | 32555317 | 0.37 | 0.25 | 0.53 | 1.47E-07 | 0.09 | 0.05 | 0.10 | SNP |
| chr6_32376019_G_T | 32376019 | 0.41 | 0.29 | 0.57 | 1.86E-07 | 0.10 | 0.05 | 0.11 | SNP |
| chr6_33144466_C_A | 33144466 | 0.16 | 0.07 | 0.30 | 2.20E-07 | 0.05 | 0.01 | 0.06 | SNP |
| chr6_33158912_G_A | 33158912 | 0.16 | 0.07 | 0.30 | 2.20E-07 | 0.05 | 0.01 | 0.06 | SNP |
| chr6_33134102_C_T | 33134102 | 1.55 | 1.31 | 1.83 | 2.21E-07 | 0.41 | 0.49 | 0.38 | SNP |
| chr6_33138015_T_C | 33138015 | 0.16 | 0.07 | 0.30 | 2.34E-07 | 0.05 | 0.01 | 0.06 | SNP |
| chr6_33151880_C_A | 33151880 | 0.16 | 0.07 | 0.30 | 2.36E-07 | 0.05 | 0.01 | 0.06 | SNP |
| chr6_33136575_T_C | 33136575 | 1.52 | 1.29 | 1.79 | 4.43E-07 | 0.43 | 0.51 | 0.41 | SNP |
| chr6_33132931_T_C | 33132931 | 1.64 | 1.35 | 1.99 | 4.68E-07 | 0.41 | 0.50 | 0.39 | SNP |
| chr6_32609216_T_G | 32609216 | 0.56 | 0.45 | 0.70 | 4.73E-07 | 0.49 | 0.40 | 0.52 | SNP |
| chr6_33132314_G_A | 33132314 | 0.15 | 0.07 | 0.29 | 4.99E-07 | 0.05 | 0.01 | 0.06 | SNP |
| chr6_32632777_C_T | 32632777 | 0.43 | 0.31 | 0.59 | 5.17E-07 | 0.14 | 0.07 | 0.15 | SNP |
| chr6_33096003_G_C | 33096003 | 0.62 | 0.51 | 0.75 | 6.25E-07 | 0.43 | 0.35 | 0.45 | SNP |
| chr6_33131734_G_A | 33131734 | 1.64 | 1.35 | 1.99 | 6.92E-07 | 0.41 | 0.51 | 0.39 | SNP |
| chr6_32609207_T_C | 32609207 | 0.57 | 0.46 | 0.71 | 6.99E-07 | 0.50 | 0.41 | 0.52 | SNP |
| chr6_33132365_T_C | 33132365 | 0.15 | 0.07 | 0.30 | 7.26E-07 | 0.05 | 0.01 | 0.06 | SNP |
| chr6_33137570_C_T | 33137570 | 0.21 | 0.10 | 0.37 | 8.15E-07 | 0.05 | 0.01 | 0.06 | SNP |
| chr6_33158898_C_T | 33158898 | 0.21 | 0.11 | 0.37 | 8.37E-07 | 0.05 | 0.01 | 0.06 | SNP |
| chr6_33138021_G_A | 33138021 | 0.21 | 0.11 | 0.37 | 9.33E-07 | 0.05 | 0.01 | 0.06 | SNP |
| chr6_32628953_G_C | 32628953 | 0.57 | 0.46 | 0.72 | 1.06E-06 | 0.48 | 0.39 | 0.50 | SNP |
| chr6_33160906_G_T | 33160906 | 0.17 | 0.08 | 0.33 | 1.21E-06 | 0.05 | 0.01 | 0.06 | SNP |
| chr6_33096009_A_C | 33096009 | 0.63 | 0.52 | 0.76 | 1.68E-06 | 0.43 | 0.35 | 0.44 | SNP |
| chr6_33079626_T_C | 33079626 | 0.18 | 0.08 | 0.35 | 2.20E-06 | 0.05 | 0.01 | 0.06 | SNP |
| chr6_33045272_C_A | 33045272 | 0.60 | 0.48 | 0.74 | 2.32E-06 | 0.22 | 0.15 | 0.25 | SNP |
| chr6_32627494_T_C | 32627494 | 1.67 | 1.35 | 2.08 | 3.04E-06 | 0.25 | 0.34 | 0.23 | SNP |
| chr6_32608880_G_A | 32608880 | 1.69 | 1.35 | 2.11 | 3.35E-06 | 0.34 | 0.42 | 0.31 | SNP |
| chr6_32410691_G_A | 32410691 | 0.50 | 0.37 | 0.66 | 3.37E-06 | 0.22 | 0.17 | 0.23 | SNP |
| chr6_32628967_T_C | 32628967 | 0.59 | 0.47 | 0.74 | 3.60E-06 | 0.48 | 0.40 | 0.51 | SNP |
| chr6_32607193_T_C | 32607193 | 1.68 | 1.35 | 2.10 | 3.79E-06 | 0.35 | 0.43 | 0.32 | SNP |
| xHLA_2field_DPB1_03_01 | 33043767 | 0.37 | 0.23 | 0.55 | 4.18E-06 | 0.07 | 0.03 | 0.08 | HLA (xHLA) |
| chr6_32412809_T_A | 32412809 | 0.51 | 0.38 | 0.68 | 4.93E-06 | 0.22 | 0.17 | 0.23 | SNP |
| chr6_32412592_G_A | 32412592 | 0.51 | 0.38 | 0.68 | 5.01E-06 | 0.22 | 0.17 | 0.23 | SNP |
| chr6_32409060_G_A | 32409060 | 0.50 | 0.37 | 0.67 | 5.10E-06 | 0.21 | 0.17 | 0.23 | SNP |
| chr6_32611968_T_G | 32611968 | 0.59 | 0.47 | 0.74 | 5.30E-06 | 0.47 | 0.39 | 0.49 | SNP |
| chr6_32411307_C_T | 32411307 | 0.51 | 0.38 | 0.68 | 5.60E-06 | 0.22 | 0.17 | 0.23 | SNP |
| chr6_32412566_T_A | 32412566 | 0.51 | 0.38 | 0.68 | 5.60E-06 | 0.22 | 0.17 | 0.23 | SNP |
| chr6_32606701_A_G | 32606701 | 0.57 | 0.44 | 0.72 | 5.67E-06 | 0.17 | 0.11 | 0.18 | SNP |
| chr6_33137727_T_C | 33137727 | 1.46 | 1.24 | 1.71 | 5.86E-06 | 0.42 | 0.50 | 0.40 | SNP |
| chr6_32406611_C_A | 32406611 | 0.51 | 0.38 | 0.68 | 6.07E-06 | 0.22 | 0.17 | 0.23 | SNP |
| chr6_32408842_A_G | 32408842 | 0.51 | 0.38 | 0.68 | 6.40E-06 | 0.22 | 0.17 | 0.23 | SNP |
| chr6_32411376_T_G | 32411376 | 0.51 | 0.38 | 0.68 | 6.86E-06 | 0.22 | 0.17 | 0.23 | SNP |
| chr6_32611723_T_G | 32611723 | 0.60 | 0.48 | 0.75 | 7.16E-06 | 0.47 | 0.39 | 0.49 | SNP |
| chr6_32501459_T_C | 32501459 | 0.63 | 0.51 | 0.77 | 7.24E-06 | 0.39 | 0.32 | 0.41 | SNP |
| chr6_32410215_T_C | 32410215 | 0.51 | 0.38 | 0.68 | 7.32E-06 | 0.22 | 0.17 | 0.23 | SNP |
| chr6_32501522_A_G | 32501522 | 1.76 | 1.37 | 2.25 | 7.78E-06 | 0.24 | 0.29 | 0.23 | SNP |
| chr6_33384473_C_T | 33384473 | 1.97 | 1.46 | 2.66 | 7.85E-06 | 0.06 | 0.11 | 0.05 | SNP |
| chr6_32410210_T_C | 32410210 | 0.52 | 0.38 | 0.69 | 8.32E-06 | 0.22 | 0.17 | 0.23 | SNP |
| chr6_32521117_T_C | 32521117 | 0.63 | 0.51 | 0.77 | 8.52E-06 | 0.48 | 0.40 | 0.51 | SNP |
| chr6_32559709_C_T | 32559709 | 0.63 | 0.51 | 0.77 | 8.57E-06 | 0.44 | 0.37 | 0.46 | SNP |
| chr6_32603029_T_C | 32603029 | 0.62 | 0.50 | 0.76 | 8.61E-06 | 0.27 | 0.19 | 0.29 | SNP |
| xHLA_2field_C_07_01 | 31236526 | 2.00 | 1.47 | 2.71 | 8.98E-06 | 0.14 | 0.17 | 0.14 | HLA (xHLA) |
| chr6_32610769_C_A | 32610769 | 0.59 | 0.46 | 0.74 | 9.19E-06 | 0.33 | 0.27 | 0.34 | SNP |
| chr6_31325090_T_C | 31325090 | 1.71 | 1.35 | 2.17 | 1.02E-05 | 0.37 | 0.40 | 0.36 | SNP |
| chr6_33039804_C_T | 33039804 | 1.50 | 1.25 | 1.79 | 1.05E-05 | 0.21 | 0.28 | 0.20 | SNP |
| chr6_32559708_G_A | 32559708 | 1.68 | 1.34 | 2.12 | 1.06E-05 | 0.28 | 0.36 | 0.25 | SNP |
| chr6_32603355_T_C | 32603355 | 0.63 | 0.51 | 0.77 | 1.11E-05 | 0.27 | 0.19 | 0.29 | SNP |
| chr6_32628030_G_A | 32628030 | 1.55 | 1.27 | 1.88 | 1.11E-05 | 0.43 | 0.50 | 0.41 | SNP |
| chr6_33623509_T_C | 33623509 | 0.25 | 0.13 | 0.45 | 1.42E-05 | 0.04 | 0.01 | 0.05 | SNP |
| chr6_32603488_A_G | 32603488 | 0.63 | 0.51 | 0.77 | 1.44E-05 | 0.27 | 0.19 | 0.29 | SNP |
| chr6_32627833_A_G | 32627833 | 1.53 | 1.26 | 1.86 | 1.53E-05 | 0.42 | 0.49 | 0.40 | SNP |
| chr6_33095890_A_T | 33095890 | 0.22 | 0.10 | 0.42 | 1.75E-05 | 0.04 | 0.01 | 0.05 | SNP |
| chr6_32612003_T_C | 32612003 | 0.61 | 0.49 | 0.77 | 1.89E-05 | 0.47 | 0.39 | 0.49 | SNP |
| chr6_33239869_C_T | 33239869 | 1.78 | 1.36 | 2.32 | 2.02E-05 | 0.09 | 0.12 | 0.08 | SNP |
| chr6_32604538_G_A | 32604538 | 1.64 | 1.31 | 2.06 | 2.08E-05 | 0.33 | 0.41 | 0.31 | SNP |
| chr6_32501296_G_A | 32501296 | 0.65 | 0.53 | 0.79 | 2.09E-05 | 0.38 | 0.31 | 0.40 | SNP |
| chr6_33044795_C_T | 33044795 | 2.17 | 1.51 | 3.10 | 2.22E-05 | 0.04 | 0.08 | 0.03 | SNP |
| chr6_32628765_C_T | 32628765 | 1.53 | 1.26 | 1.86 | 2.33E-05 | 0.40 | 0.47 | 0.38 | SNP |
| chr6_32611725_G_A | 32611725 | 0.62 | 0.49 | 0.77 | 2.37E-05 | 0.46 | 0.39 | 0.49 | SNP |
| chr6_32501301_G_C | 32501301 | 0.65 | 0.53 | 0.79 | 2.37E-05 | 0.38 | 0.32 | 0.40 | SNP |
| chr6_33095584_G_C | 33095584 | 1.67 | 1.31 | 2.11 | 2.46E-05 | 0.11 | 0.15 | 0.10 | SNP |
| chr6_32610704_C_T | 32610704 | 0.60 | 0.48 | 0.76 | 2.49E-05 | 0.32 | 0.27 | 0.34 | SNP |
| chr6_31235505_G_A | 31235505 | 1.72 | 1.34 | 2.22 | 2.58E-05 | 0.21 | 0.24 | 0.21 | SNP |
| chr6_32628439_G_A | 32628439 | 1.52 | 1.25 | 1.85 | 2.61E-05 | 0.40 | 0.48 | 0.39 | SNP |
| xHLA_2field_DPB1_02_01 | 33043767 | 0.52 | 0.38 | 0.70 | 2.64E-05 | 0.11 | 0.06 | 0.12 | HLA (xHLA) |
| chr6_32628571_C_T | 32628571 | 1.61 | 1.29 | 2.00 | 2.75E-05 | 0.23 | 0.30 | 0.21 | SNP |
| chr6_31235869_T_C | 31235869 | 1.71 | 1.33 | 2.20 | 2.77E-05 | 0.22 | 0.24 | 0.21 | SNP |
| chr6_32521951_A_C | 32521951 | 0.64 | 0.52 | 0.79 | 2.82E-05 | 0.37 | 0.30 | 0.39 | SNP |
| chr6_32628916_G_A | 32628916 | 1.57 | 1.27 | 1.94 | 2.85E-05 | 0.24 | 0.31 | 0.22 | SNP |
| chr6_32553418_T_G | 32553418 | 0.64 | 0.51 | 0.79 | 2.87E-05 | 0.40 | 0.34 | 0.42 | SNP |
| chr6_32612050_T_A | 32612050 | 0.63 | 0.50 | 0.78 | 2.94E-05 | 0.46 | 0.39 | 0.49 | SNP |
| chr6_32636040_C_T | 32636040 | 0.60 | 0.47 | 0.76 | 2.99E-05 | 0.31 | 0.25 | 0.32 | SNP |
| chr6_32628941_G_C | 32628941 | 1.58 | 1.27 | 1.95 | 3.00E-05 | 0.24 | 0.31 | 0.22 | SNP |
| chr6_32627777_G_C | 32627777 | 1.56 | 1.27 | 1.92 | 3.08E-05 | 0.24 | 0.32 | 0.22 | SNP |
| chr6_32520595_G_A | 32520595 | 0.66 | 0.54 | 0.80 | 3.19E-05 | 0.38 | 0.31 | 0.39 | SNP |
| chr6_32636289_T_C | 32636289 | 1.58 | 1.27 | 1.96 | 3.50E-05 | 0.24 | 0.31 | 0.22 | SNP |
| chr6_32408735_A_C | 32408735 | 0.63 | 0.51 | 0.78 | 3.52E-05 | 0.33 | 0.26 | 0.35 | SNP |
| chr6_32628605_T_C | 32628605 | 1.59 | 1.27 | 1.98 | 3.63E-05 | 0.23 | 0.30 | 0.22 | SNP |
| chr6_32559714_G_A | 32559714 | 0.65 | 0.52 | 0.79 | 3.77E-05 | 0.45 | 0.38 | 0.47 | SNP |
| chr6_32628506_C_A | 32628506 | 1.58 | 1.27 | 1.97 | 3.96E-05 | 0.23 | 0.30 | 0.21 | SNP |
| chr6_32634007_A_G | 32634007 | 1.55 | 1.26 | 1.92 | 4.18E-05 | 0.24 | 0.31 | 0.22 | SNP |
| xHLA_2field_DRB1_13_02 | 32546552 | 0.36 | 0.21 | 0.57 | 4.27E-05 | 0.05 | 0.02 | 0.06 | HLA (xHLA) |
| chr6_32629639_T_C | 32629639 | 1.55 | 1.26 | 1.91 | 4.28E-05 | 0.24 | 0.32 | 0.22 | SNP |
| chr6_32501311_C_T | 32501311 | 0.66 | 0.54 | 0.80 | 4.29E-05 | 0.38 | 0.32 | 0.40 | SNP |
| chr6_32187202_T_C | 32187202 | 1.52 | 1.25 | 1.87 | 4.35E-05 | 0.43 | 0.49 | 0.42 | SNP |
| chr6_32629398_G_C | 32629398 | 1.55 | 1.25 | 1.91 | 4.49E-05 | 0.24 | 0.32 | 0.23 | SNP |
| chr6_33048119_C_T | 33048119 | 2.05 | 1.45 | 2.90 | 4.62E-05 | 0.04 | 0.07 | 0.03 | SNP |
| chr6_32607031_G_T | 32607031 | 1.60 | 1.27 | 2.00 | 4.67E-05 | 0.32 | 0.42 | 0.30 | SNP |
| chr6_32634556_G_A | 32634556 | 1.57 | 1.26 | 1.94 | 4.68E-05 | 0.25 | 0.32 | 0.23 | SNP |
| chr6_32631029_T_C | 32631029 | 0.37 | 0.22 | 0.58 | 4.70E-05 | 0.06 | 0.03 | 0.07 | SNP |
| chr6_32607174_C_T | 32607174 | 1.57 | 1.26 | 1.94 | 4.70E-05 | 0.35 | 0.43 | 0.33 | SNP |
| chr6_31325494_C_T | 31325494 | 1.71 | 1.32 | 2.21 | 4.83E-05 | 0.33 | 0.36 | 0.32 | SNP |
| chr6_32629297_C_T | 32629297 | 0.64 | 0.52 | 0.80 | 4.86E-05 | 0.44 | 0.36 | 0.46 | SNP |
| chr6_33132338_A_G | 33132338 | 1.47 | 1.22 | 1.77 | 4.90E-05 | 0.41 | 0.49 | 0.39 | SNP |
| chr6_32636591_G_A | 32636591 | 0.60 | 0.46 | 0.76 | 5.06E-05 | 0.45 | 0.37 | 0.47 | SNP |
| chr6_32603412_T_C | 32603412 | 1.49 | 1.23 | 1.81 | 5.10E-05 | 0.49 | 0.57 | 0.46 | SNP |
| chr6_32609514_T_C | 32609514 | 0.64 | 0.51 | 0.79 | 5.38E-05 | 0.45 | 0.38 | 0.47 | SNP |
| chr6_33049881_T_G | 33049881 | 0.55 | 0.40 | 0.73 | 5.43E-05 | 0.11 | 0.07 | 0.12 | SNP |
| chr6_32636221_G_T | 32636221 | 0.64 | 0.51 | 0.79 | 5.49E-05 | 0.45 | 0.37 | 0.47 | SNP |
| chr6_32629422_T_C | 32629422 | 1.54 | 1.25 | 1.90 | 5.64E-05 | 0.24 | 0.31 | 0.22 | SNP |
| chr6_31325513_G_C | 31325513 | 1.70 | 1.31 | 2.20 | 5.79E-05 | 0.33 | 0.36 | 0.32 | SNP |
| chr6_33647936_C_T | 33647936 | 0.23 | 0.11 | 0.45 | 5.81E-05 | 0.04 | 0.01 | 0.05 | SNP |
| chr6_32634425_C_T | 32634425 | 1.54 | 1.25 | 1.90 | 5.92E-05 | 0.24 | 0.31 | 0.22 | SNP |
| chr6_32407302_A_G | 32407302 | 0.64 | 0.51 | 0.79 | 5.96E-05 | 0.32 | 0.25 | 0.34 | SNP |
| chr6_32611931_T_A | 32611931 | 0.63 | 0.51 | 0.79 | 5.98E-05 | 0.46 | 0.39 | 0.48 | SNP |
| chr6_32629755_G_A | 32629755 | 1.54 | 1.25 | 1.90 | 6.04E-05 | 0.24 | 0.31 | 0.22 | SNP |
| chr6_32628760_C_A | 32628760 | 1.55 | 1.25 | 1.92 | 6.07E-05 | 0.24 | 0.31 | 0.22 | SNP |
| chr6_32408917_C_T | 32408917 | 1.54 | 1.25 | 1.90 | 6.15E-05 | 0.26 | 0.30 | 0.25 | SNP |
| chr6_31325504_C_T | 31325504 | 1.69 | 1.31 | 2.19 | 6.27E-05 | 0.33 | 0.36 | 0.32 | SNP |
| chr6_32635033_T_G | 32635033 | 1.53 | 1.24 | 1.89 | 6.29E-05 | 0.24 | 0.31 | 0.22 | SNP |
| chr6_32603098_T_C | 32603098 | 1.48 | 1.22 | 1.80 | 6.29E-05 | 0.48 | 0.57 | 0.46 | SNP |
| chr6_32633665_T_C | 32633665 | 0.61 | 0.48 | 0.78 | 6.31E-05 | 0.31 | 0.26 | 0.32 | SNP |
| chr6_31325341_C_G | 31325341 | 1.70 | 1.31 | 2.20 | 6.40E-05 | 0.33 | 0.36 | 0.32 | SNP |
| chr6_32636351_T_C | 32636351 | 1.54 | 1.25 | 1.90 | 6.46E-05 | 0.24 | 0.31 | 0.22 | SNP |
| chr6_32636280_G_T | 32636280 | 1.55 | 1.25 | 1.93 | 6.72E-05 | 0.24 | 0.30 | 0.22 | SNP |
| chr6_32629543_T_C | 32629543 | 1.53 | 1.24 | 1.88 | 6.96E-05 | 0.25 | 0.32 | 0.23 | SNP |
| chr6_32627747_G_A | 32627747 | 0.61 | 0.48 | 0.78 | 6.97E-05 | 0.30 | 0.25 | 0.32 | SNP |
| chr6_32406887_C_T | 32406887 | 0.63 | 0.49 | 0.79 | 7.01E-05 | 0.31 | 0.23 | 0.33 | SNP |
| chr6_32633781_C_T | 32633781 | 1.53 | 1.24 | 1.88 | 7.01E-05 | 0.24 | 0.32 | 0.23 | SNP |
| chr6_32633636_G_A | 32633636 | 1.53 | 1.24 | 1.88 | 7.13E-05 | 0.24 | 0.31 | 0.23 | SNP |
| chr6_33095865_C_T | 33095865 | 1.61 | 1.27 | 2.04 | 7.17E-05 | 0.11 | 0.15 | 0.10 | SNP |
| chr6_32627561_C_T | 32627561 | 0.61 | 0.48 | 0.78 | 7.19E-05 | 0.31 | 0.26 | 0.33 | SNP |
| chr6_32407404_G_C | 32407404 | 0.64 | 0.51 | 0.80 | 7.20E-05 | 0.33 | 0.25 | 0.35 | SNP |
| chr6_32407468_T_C | 32407468 | 0.64 | 0.51 | 0.80 | 7.20E-05 | 0.33 | 0.25 | 0.35 | SNP |
| chr6_32407310_T_G | 32407310 | 0.64 | 0.51 | 0.80 | 7.28E-05 | 0.32 | 0.25 | 0.34 | SNP |
| chr6_32551568_C_G | 32551568 | 0.66 | 0.54 | 0.81 | 7.38E-05 | 0.38 | 0.32 | 0.40 | SNP |
| chr6_31325380_T_C | 31325380 | 1.68 | 1.30 | 2.18 | 7.96E-05 | 0.33 | 0.36 | 0.32 | SNP |
| chr6_32556491_A_G | 32556491 | 0.49 | 0.34 | 0.69 | 7.98E-05 | 0.09 | 0.07 | 0.10 | SNP |
| chr6_31325532_A_G | 31325532 | 1.68 | 1.30 | 2.17 | 8.06E-05 | 0.33 | 0.36 | 0.32 | SNP |
| chr6_32635172_C_T | 32635172 | 1.52 | 1.23 | 1.88 | 8.19E-05 | 0.24 | 0.31 | 0.23 | SNP |
| chr6_32439696_T_G | 32439696 | 0.39 | 0.24 | 0.61 | 8.43E-05 | 0.05 | 0.03 | 0.06 | SNP |
| chr6_32634355_G_A | 32634355 | 1.53 | 1.24 | 1.89 | 8.49E-05 | 0.24 | 0.31 | 0.22 | SNP |
| chr6_32611971_G_C | 32611971 | 1.58 | 1.26 | 1.98 | 8.52E-05 | 0.29 | 0.36 | 0.26 | SNP |
| chr6_31325526_A_G | 31325526 | 1.67 | 1.29 | 2.16 | 8.67E-05 | 0.33 | 0.36 | 0.32 | SNP |
| chr6_32633899_T_C | 32633899 | 1.51 | 1.23 | 1.86 | 8.78E-05 | 0.25 | 0.32 | 0.23 | SNP |
| chr6_32612397_A_G | 32612397 | 1.53 | 1.24 | 1.90 | 8.86E-05 | 0.47 | 0.56 | 0.45 | SNP |
| chr6_32636217_G_A | 32636217 | 1.53 | 1.24 | 1.89 | 8.90E-05 | 0.24 | 0.31 | 0.22 | SNP |
| chr6_32629530_C_T | 32629530 | 1.52 | 1.23 | 1.87 | 8.97E-05 | 0.25 | 0.32 | 0.23 | SNP |
| chr6_32628784_A_C | 32628784 | 0.64 | 0.51 | 0.80 | 8.97E-05 | 0.47 | 0.40 | 0.49 | SNP |
| chr6_32407153_A_T | 32407153 | 0.64 | 0.52 | 0.80 | 9.10E-05 | 0.32 | 0.25 | 0.34 | SNP |

| | | | | | | | | | |
|---|---|---|---|---|---|---|---|---|---|
| chr6_32603277_G_T | 32603277 | 0.40 | 0.24 | 0.62 | 9.11E-05 | 0.05 | 0.03 | 0.06 | SNP |
| chr6_32636494_C_T | 32636494 | 0.65 | 0.52 | 0.80 | 9.35E-05 | 0.42 | 0.34 | 0.44 | SNP |
| chr6_32634646_T_C | 32634646 | 1.52 | 1.23 | 1.87 | 9.39E-05 | 0.24 | 0.31 | 0.22 | SNP |
| chr6_32606376_T_C | 32606376 | 0.39 | 0.24 | 0.61 | 9.47E-05 | 0.05 | 0.02 | 0.06 | SNP |
| chr6_32635230_G_A | 32635230 | 1.55 | 1.24 | 1.93 | 9.52E-05 | 0.23 | 0.30 | 0.22 | SNP |
| chr6_32628787_C_T | 32628787 | 1.54 | 1.24 | 1.91 | 9.65E-05 | 0.24 | 0.31 | 0.22 | SNP |
| chr6_32609009_T_G | 32609009 | 0.40 | 0.25 | 0.62 | 9.66E-05 | 0.05 | 0.03 | 0.06 | SNP |
| chr6_32606682_C_G | 32606682 | 0.40 | 0.25 | 0.62 | 9.96E-05 | 0.05 | 0.03 | 0.06 | SNP |
| chr6_32804570_G_A | 32804570 | 1.44 | 1.20 | 1.74 | 0.00010 | 0.37 | 0.45 | 0.35 | SNP |
| chr6_32628617_A_C | 32628617 | 1.53 | 1.24 | 1.90 | 0.00010 | 0.24 | 0.31 | 0.22 | SNP |
| chr6_32606691_T_G | 32606691 | 0.40 | 0.25 | 0.62 | 0.00010 | 0.05 | 0.03 | 0.06 | SNP |
| chr6_32609047_C_T | 32609047 | 0.40 | 0.25 | 0.62 | 0.00010 | 0.05 | 0.03 | 0.06 | SNP |
| chr6_32603896_T_C | 32603896 | 0.40 | 0.25 | 0.62 | 0.00010 | 0.05 | 0.03 | 0.06 | SNP |
| chr6_32604166_A_G | 32604166 | 0.40 | 0.25 | 0.62 | 0.00011 | 0.05 | 0.03 | 0.06 | SNP |
| chr6_32406412_C_G | 32406412 | 0.65 | 0.52 | 0.80 | 0.00011 | 0.32 | 0.25 | 0.34 | SNP |
| chr6_32609312_C_A | 32609312 | 0.64 | 0.51 | 0.80 | 0.00011 | 0.46 | 0.38 | 0.48 | SNP |
| chr6_32441129_G_A | 32441129 | 0.40 | 0.25 | 0.62 | 0.00011 | 0.05 | 0.03 | 0.06 | SNP |
| chr6_31325529_C_T | 31325529 | 1.66 | 1.28 | 2.14 | 0.00011 | 0.33 | 0.36 | 0.32 | SNP |
| chr6_31325521_C_G | 31325521 | 1.66 | 1.28 | 2.14 | 0.00011 | 0.33 | 0.36 | 0.32 | SNP |
| chr6_32441046_G_A | 32441046 | 0.40 | 0.25 | 0.62 | 0.00011 | 0.05 | 0.03 | 0.06 | SNP |
| chr6_32520774_C_T | 32520774 | 1.53 | 1.24 | 1.91 | 0.00011 | 0.31 | 0.39 | 0.29 | SNP |
| chr6_32611964_C_T | 32611964 | 1.56 | 1.25 | 1.96 | 0.00011 | 0.29 | 0.36 | 0.27 | SNP |
| chr6_32609453_C_T | 32609453 | 1.55 | 1.24 | 1.93 | 0.00011 | 0.34 | 0.42 | 0.32 | SNP |
| chr6_32608435_T_C | 32608435 | 0.40 | 0.25 | 0.63 | 0.00012 | 0.05 | 0.03 | 0.06 | SNP |
| chr6_32559045_T_C | 32559045 | 0.68 | 0.55 | 0.82 | 0.00012 | 0.43 | 0.37 | 0.44 | SNP |
| chr6_32409484_C_T | 32409484 | 0.65 | 0.52 | 0.81 | 0.00012 | 0.32 | 0.25 | 0.34 | SNP |
| chr6_32633767_G_T | 32633767 | 1.51 | 1.22 | 1.86 | 0.00012 | 0.24 | 0.31 | 0.22 | SNP |
| chr6_32628606_C_A | 32628606 | 0.40 | 0.25 | 0.63 | 0.00012 | 0.05 | 0.03 | 0.06 | SNP |
| chr6_32190390_T_G | 32190390 | 1.52 | 1.23 | 1.88 | 0.00012 | 0.34 | 0.42 | 0.32 | SNP |
| chr6_31321267_A_C | 31321267 | 2.47 | 1.56 | 3.92 | 0.00012 | 0.12 | 0.13 | 0.12 | SNP |
| chr6_32634834_T_C | 32634834 | 0.65 | 0.52 | 0.81 | 0.00012 | 0.45 | 0.37 | 0.47 | SNP |
| chr6_32611873_C_T | 32611873 | 0.65 | 0.52 | 0.81 | 0.00012 | 0.46 | 0.39 | 0.48 | SNP |
| chr6_32611874_T_G | 32611874 | 0.65 | 0.52 | 0.81 | 0.00012 | 0.46 | 0.39 | 0.48 | SNP |
| chr6_32634838_C_A | 32634838 | 0.65 | 0.52 | 0.81 | 0.00012 | 0.45 | 0.37 | 0.47 | SNP |
| chr6_32629531_A_G | 32629531 | 1.51 | 1.22 | 1.86 | 0.00013 | 0.24 | 0.31 | 0.23 | SNP |
| chr6_32407440_T_C | 32407440 | 0.65 | 0.52 | 0.81 | 0.00013 | 0.32 | 0.25 | 0.34 | SNP |
| chr6_32635974_A_G | 32635974 | 1.51 | 1.22 | 1.86 | 0.00013 | 0.24 | 0.31 | 0.22 | SNP |
| chr6_32629320_G_A | 32629320 | 0.63 | 0.50 | 0.80 | 0.00013 | 0.32 | 0.27 | 0.34 | SNP |
| chr6_32634545_C_A | 32634545 | 1.51 | 1.22 | 1.86 | 0.00013 | 0.24 | 0.31 | 0.22 | SNP |
| chr6_32629549_G_A | 32629549 | 1.51 | 1.22 | 1.86 | 0.00013 | 0.24 | 0.31 | 0.23 | SNP |
| chr6_32611450_T_G | 32611450 | 0.65 | 0.52 | 0.81 | 0.00013 | 0.47 | 0.40 | 0.49 | SNP |
| chr6_32628737_G_A | 32628737 | 0.65 | 0.52 | 0.81 | 0.00013 | 0.47 | 0.39 | 0.49 | SNP |
| chr6_32628776_A_C | 32628776 | 0.65 | 0.52 | 0.81 | 0.00013 | 0.47 | 0.40 | 0.49 | SNP |
| chr6_32634091_G_C | 32634091 | 1.51 | 1.22 | 1.87 | 0.00013 | 0.24 | 0.31 | 0.22 | SNP |
| chr6_32442703_G_A | 32442703 | 0.39 | 0.24 | 0.62 | 0.00013 | 0.05 | 0.02 | 0.06 | SNP |
| chr6_32605705_T_G | 32605705 | 0.66 | 0.53 | 0.81 | 0.00014 | 0.45 | 0.38 | 0.47 | SNP |
| chr6_32634967_T_A | 32634967 | 1.50 | 1.22 | 1.85 | 0.00014 | 0.24 | 0.31 | 0.23 | SNP |
| chr6_32634090_T_C | 32634090 | 1.51 | 1.22 | 1.86 | 0.00014 | 0.24 | 0.31 | 0.22 | SNP |
| chr6_32634057_C_T | 32634057 | 1.51 | 1.22 | 1.86 | 0.00014 | 0.24 | 0.31 | 0.22 | SNP |
| chr6_32605708_A_G | 32605708 | 0.66 | 0.53 | 0.81 | 0.00014 | 0.45 | 0.38 | 0.47 | SNP |
| chr6_32407433_G_C | 32407433 | 0.65 | 0.52 | 0.81 | 0.00014 | 0.32 | 0.25 | 0.34 | SNP |
| chr6_32634485_C_A | 32634485 | 0.63 | 0.50 | 0.80 | 0.00014 | 0.32 | 0.27 | 0.33 | SNP |
| chr6_32634593_G_C | 32634593 | 1.50 | 1.22 | 1.86 | 0.00014 | 0.24 | 0.31 | 0.22 | SNP |
| chr6_32604564_C_T | 32604564 | 1.55 | 1.24 | 1.94 | 0.00014 | 0.33 | 0.41 | 0.31 | SNP |
| chr6_32636434_G_A | 32636434 | 0.65 | 0.53 | 0.81 | 0.00014 | 0.45 | 0.38 | 0.47 | SNP |
| chr6_32610947_A_C | 32610947 | 0.42 | 0.26 | 0.64 | 0.00014 | 0.06 | 0.03 | 0.06 | SNP |
| chr6_32520665_A_C | 32520665 | 0.68 | 0.55 | 0.82 | 0.00014 | 0.37 | 0.31 | 0.39 | SNP |
| chr6_32408012_G_A | 32408012 | 0.65 | 0.52 | 0.81 | 0.00014 | 0.32 | 0.25 | 0.34 | SNP |
| chr6_31243828_A_G | 31243828 | 0.68 | 0.55 | 0.83 | 0.00015 | 0.32 | 0.29 | 0.33 | SNP |
| chr6_32604372_A_G | 32604372 | 0.65 | 0.52 | 0.81 | 0.00015 | 0.46 | 0.38 | 0.48 | SNP |
| chr6_32633959_T_C | 32633959 | 1.50 | 1.22 | 1.85 | 0.00015 | 0.24 | 0.31 | 0.23 | SNP |
| chr6_32408597_G_A | 32408597 | 0.65 | 0.52 | 0.81 | 0.00015 | 0.32 | 0.25 | 0.34 | SNP |
| chr6_32634094_T_C | 32634094 | 1.50 | 1.22 | 1.86 | 0.00015 | 0.24 | 0.31 | 0.22 | SNP |
| chr6_32629802_G_A | 32629802 | 0.66 | 0.53 | 0.82 | 0.00015 | 0.45 | 0.37 | 0.47 | SNP |
| chr6_32629066_T_C | 32629066 | 1.52 | 1.22 | 1.89 | 0.00015 | 0.23 | 0.30 | 0.22 | SNP |
| chr6_32629328_G_A | 32629328 | 1.52 | 1.22 | 1.89 | 0.00015 | 0.23 | 0.30 | 0.22 | SNP |
| chr6_32520465_A_G | 32520465 | 0.68 | 0.55 | 0.83 | 0.00016 | 0.38 | 0.31 | 0.39 | SNP |
| chr6_32609425_C_G | 32609425 | 0.43 | 0.27 | 0.65 | 0.00016 | 0.05 | 0.03 | 0.06 | SNP |
| chr6_32627489_G_A | 32627489 | 0.64 | 0.50 | 0.80 | 0.00016 | 0.43 | 0.36 | 0.45 | SNP |
| chr6_32628751_T_C | 32628751 | 0.66 | 0.53 | 0.82 | 0.00016 | 0.47 | 0.40 | 0.49 | SNP |
| chr6_32604931_C_A | 32604931 | 1.51 | 1.22 | 1.87 | 0.00016 | 0.30 | 0.39 | 0.28 | SNP |
| chr6_32604968_A_C | 32604968 | 0.60 | 0.46 | 0.78 | 0.00016 | 0.25 | 0.20 | 0.26 | SNP |
| chr6_32604000_T_G | 32604000 | 0.66 | 0.53 | 0.82 | 0.00016 | 0.45 | 0.38 | 0.47 | SNP |
| chr6_32629545_T_C | 32629545 | 0.41 | 0.25 | 0.64 | 0.00016 | 0.05 | 0.03 | 0.06 | SNP |
| chr6_32634506_C_T | 32634506 | 1.49 | 1.21 | 1.84 | 0.00017 | 0.25 | 0.31 | 0.23 | SNP |
| chr6_32633273_T_G | 32633273 | 0.66 | 0.53 | 0.82 | 0.00017 | 0.35 | 0.29 | 0.37 | SNP |
| chr6_32609105_A_G | 32609105 | 0.66 | 0.53 | 0.82 | 0.00017 | 0.46 | 0.39 | 0.48 | SNP |
| chr6_32605274_C_T | 32605274 | 0.66 | 0.53 | 0.82 | 0.00017 | 0.45 | 0.38 | 0.47 | SNP |
| chr6_32628843_C_T | 32628843 | 0.41 | 0.25 | 0.64 | 0.00017 | 0.05 | 0.03 | 0.06 | SNP |
| chr6_32633909_T_A | 32633909 | 0.64 | 0.51 | 0.81 | 0.00017 | 0.33 | 0.27 | 0.34 | SNP |
| chr6_32634098_C_G | 32634098 | 1.50 | 1.21 | 1.85 | 0.00017 | 0.24 | 0.31 | 0.23 | SNP |
| chr6_32627714_G_T | 32627714 | 0.66 | 0.53 | 0.82 | 0.00017 | 0.45 | 0.37 | 0.47 | SNP |
| chr6_32629409_G_A | 32629409 | 0.63 | 0.50 | 0.80 | 0.00017 | 0.31 | 0.26 | 0.33 | SNP |
| chr6_32604394_T_C | 32604394 | 1.52 | 1.22 | 1.90 | 0.00017 | 0.30 | 0.38 | 0.28 | SNP |
| chr6_32635293_C_G | 32635293 | 1.50 | 1.21 | 1.86 | 0.00018 | 0.24 | 0.31 | 0.22 | SNP |
| chr6_32606085_T_A | 32606085 | 0.66 | 0.53 | 0.82 | 0.00018 | 0.45 | 0.38 | 0.47 | SNP |
| chr6_32611243_C_T | 32611243 | 1.52 | 1.22 | 1.90 | 0.00019 | 0.29 | 0.36 | 0.27 | SNP |
| chr6_32605295_C_T | 32605295 | 1.48 | 1.21 | 1.83 | 0.00019 | 0.33 | 0.41 | 0.30 | SNP |
| chr6_32629582_T_C | 32629582 | 0.64 | 0.50 | 0.80 | 0.00019 | 0.30 | 0.25 | 0.32 | SNP |
| chr6_32635949_G_A | 32635949 | 1.45 | 1.19 | 1.77 | 0.00019 | 0.29 | 0.36 | 0.27 | SNP |
| chr6_32973303_T_C | 32973303 | 0.54 | 0.38 | 0.74 | 0.00019 | 0.10 | 0.06 | 0.11 | SNP |
| chr6_32709964_T_C | 32709964 | 0.67 | 0.54 | 0.83 | 0.00020 | 0.31 | 0.25 | 0.33 | SNP |
| chr6_32607008_T_G | 32607008 | 0.66 | 0.53 | 0.82 | 0.00020 | 0.45 | 0.38 | 0.47 | SNP |
| chr6_32603968_G_A | 32603968 | 0.66 | 0.53 | 0.82 | 0.00020 | 0.45 | 0.38 | 0.47 | SNP |
| chr6_32605754_A_T | 32605754 | 0.66 | 0.53 | 0.82 | 0.00020 | 0.45 | 0.38 | 0.47 | SNP |
| chr6_32192436_A_G | 32192436 | 0.46 | 0.30 | 0.69 | 0.00020 | 0.06 | 0.03 | 0.07 | SNP |
| chr6_32611650_G_A | 32611650 | 1.53 | 1.22 | 1.91 | 0.00020 | 0.29 | 0.36 | 0.27 | SNP |
| chr6_32608467_G_A | 32608467 | 1.52 | 1.22 | 1.89 | 0.00020 | 0.29 | 0.37 | 0.27 | SNP |
| chr6_32631039_G_A | 32631039 | 0.63 | 0.49 | 0.80 | 0.00020 | 0.36 | 0.31 | 0.38 | SNP |
| chr6_32634261_C_T | 32634261 | 1.49 | 1.21 | 1.84 | 0.00021 | 0.24 | 0.31 | 0.23 | SNP |
| chr6_32634262_T_G | 32634262 | 1.49 | 1.21 | 1.84 | 0.00021 | 0.24 | 0.31 | 0.23 | SNP |
| chr6_32633467_A_G | 32633467 | 0.52 | 0.36 | 0.72 | 0.00021 | 0.09 | 0.05 | 0.10 | SNP |
| chr6_32634387_T_C | 32634387 | 0.64 | 0.50 | 0.81 | 0.00021 | 0.31 | 0.26 | 0.32 | SNP |
| chr6_31836827_G_T | 31836827 | 1.66 | 1.27 | 2.17 | 0.00021 | 0.33 | 0.35 | 0.33 | SNP |
| chr6_32634010_A_G | 32634010 | 0.64 | 0.50 | 0.81 | 0.00022 | 0.31 | 0.26 | 0.32 | SNP |
| chr6_32634755_C_T | 32634755 | 1.48 | 1.20 | 1.83 | 0.00022 | 0.24 | 0.31 | 0.23 | SNP |
| chr6_32628633_T_C | 32628633 | 1.50 | 1.21 | 1.85 | 0.00022 | 0.24 | 0.30 | 0.22 | SNP |
| chr6_32635266_T_G | 32635266 | 1.51 | 1.21 | 1.88 | 0.00022 | 0.23 | 0.30 | 0.21 | SNP |
| chr6_32633906_A_C | 32633906 | 0.64 | 0.51 | 0.81 | 0.00022 | 0.33 | 0.28 | 0.34 | SNP |
| chr6_32610059_G_T | 32610059 | 0.67 | 0.53 | 0.83 | 0.00023 | 0.45 | 0.38 | 0.47 | SNP |
| chr6_32188383_T_C | 32188383 | 1.44 | 1.19 | 1.74 | 0.00023 | 0.42 | 0.48 | 0.41 | SNP |
| chr6_31326629_T_C | 31326629 | 1.58 | 1.24 | 2.03 | 0.00024 | 0.35 | 0.37 | 0.34 | SNP |
| chr6_32611504_C_T | 32611504 | 0.66 | 0.53 | 0.83 | 0.00025 | 0.47 | 0.40 | 0.49 | SNP |
| chr6_32520639_G_A | 32520639 | 1.49 | 1.20 | 1.85 | 0.00025 | 0.31 | 0.40 | 0.29 | SNP |
| chr6_32634139_C_T | 32634139 | 1.48 | 1.20 | 1.83 | 0.00025 | 0.24 | 0.31 | 0.23 | SNP |
| chr6_32709990_C_T | 32709990 | 0.67 | 0.55 | 0.83 | 0.00025 | 0.31 | 0.25 | 0.33 | SNP |
| chr6_32634137_A_C | 32634137 | 1.48 | 1.20 | 1.83 | 0.00025 | 0.24 | 0.31 | 0.22 | SNP |
| chr6_32605943_A_G | 32605943 | 0.67 | 0.54 | 0.83 | 0.00026 | 0.45 | 0.38 | 0.47 | SNP |
| chr6_32628736_T_C | 32628736 | 0.67 | 0.54 | 0.83 | 0.00026 | 0.47 | 0.39 | 0.49 | SNP |
| chr6_32611474_G_A | 32611474 | 1.52 | 1.21 | 1.90 | 0.00026 | 0.27 | 0.35 | 0.25 | SNP |
| chr6_32611234_C_T | 32611234 | 1.50 | 1.21 | 1.87 | 0.00026 | 0.29 | 0.36 | 0.27 | SNP |
| chr6_32608473_G_A | 32608473 | 1.50 | 1.21 | 1.87 | 0.00026 | 0.30 | 0.37 | 0.28 | SNP |
| chr6_32628283_G_A | 32628283 | 1.53 | 1.22 | 1.92 | 0.00027 | 0.23 | 0.30 | 0.21 | SNP |
| chr6_32336299_A_G | 32336299 | 0.38 | 0.22 | 0.63 | 0.00027 | 0.04 | 0.02 | 0.05 | SNP |
| chr6_32804687_T_C | 32804687 | 1.74 | 1.29 | 2.33 | 0.00027 | 0.07 | 0.10 | 0.06 | SNP |
| chr6_32151934_A_G | 32151934 | 1.79 | 1.30 | 2.45 | 0.00028 | 0.16 | 0.18 | 0.16 | SNP |
| chr6_32611466_A_G | 32611466 | 1.52 | 1.21 | 1.90 | 0.00028 | 0.27 | 0.35 | 0.25 | SNP |
| chr6_32441696_C_T | 32441696 | 0.49 | 0.33 | 0.71 | 0.00029 | 0.07 | 0.04 | 0.07 | SNP |
| chr6_32526540_A_G | 32526540 | 0.70 | 0.57 | 0.85 | 0.00029 | 0.41 | 0.35 | 0.42 | SNP |
| chr6_32627535_T_G | 32627535 | 0.66 | 0.53 | 0.83 | 0.00029 | 0.45 | 0.38 | 0.47 | SNP |
| chr6_32634150_A_C | 32634150 | 1.48 | 1.20 | 1.82 | 0.00029 | 0.24 | 0.31 | 0.23 | SNP |
| chr6_32635248_A_T | 32635248 | 1.51 | 1.21 | 1.88 | 0.00030 | 0.23 | 0.29 | 0.21 | SNP |
| chr6_32550947_A_G | 32550947 | 1.54 | 1.22 | 1.94 | 0.00030 | 0.22 | 0.30 | 0.20 | SNP |
| chr6_32629314_A_G | 32629314 | 0.67 | 0.53 | 0.83 | 0.00030 | 0.47 | 0.40 | 0.49 | SNP |
| chr6_32635226_G_T | 32635226 | 0.67 | 0.54 | 0.83 | 0.00030 | 0.46 | 0.39 | 0.48 | SNP |
| chr6_32520664_G_A | 32520664 | 1.48 | 1.20 | 1.83 | 0.00030 | 0.31 | 0.40 | 0.29 | SNP |
| chr6_32608537_A_C | 32608537 | 1.49 | 1.20 | 1.86 | 0.00031 | 0.30 | 0.37 | 0.27 | SNP |
| chr6_32605724_A_G | 32605724 | 1.49 | 1.20 | 1.85 | 0.00031 | 0.29 | 0.37 | 0.27 | SNP |
| chr6_32627600_T_G | 32627600 | 0.67 | 0.54 | 0.83 | 0.00031 | 0.45 | 0.38 | 0.47 | SNP |
| chr6_32629330_C_T | 32629330 | 1.49 | 1.20 | 1.85 | 0.00032 | 0.23 | 0.30 | 0.22 | SNP |
| chr6_32339498_C_G | 32339498 | 0.40 | 0.24 | 0.64 | 0.00032 | 0.04 | 0.02 | 0.05 | SNP |
| chr6_32612111_G_A | 32612111 | 1.50 | 1.20 | 1.87 | 0.00032 | 0.29 | 0.37 | 0.27 | SNP |
| chr6_32149816_T_C | 32149816 | 1.78 | 1.30 | 2.44 | 0.00032 | 0.16 | 0.18 | 0.16 | SNP |
| chr6_32627646_G_A | 32627646 | 0.67 | 0.54 | 0.83 | 0.00033 | 0.45 | 0.38 | 0.47 | SNP |
| chr6_31910938_G_T | 31910938 | 0.52 | 0.36 | 0.74 | 0.00033 | 0.08 | 0.05 | 0.09 | SNP |
| chr6_32611958_C_G | 32611958 | 1.52 | 1.21 | 1.91 | 0.00033 | 0.28 | 0.36 | 0.27 | SNP |
| chr6_32607047_T_G | 32607047 | 1.50 | 1.20 | 1.87 | 0.00033 | 0.32 | 0.40 | 0.30 | SNP |
| chr6_32487677_T_C | 32487677 | 1.69 | 1.27 | 2.26 | 0.00033 | 0.16 | 0.23 | 0.14 | SNP |
| chr6_32627517_G_T | 32627517 | 0.66 | 0.53 | 0.83 | 0.00034 | 0.45 | 0.37 | 0.47 | SNP |
| chr6_32813214_G_A | 32813214 | 1.76 | 1.29 | 2.39 | 0.00034 | 0.06 | 0.09 | 0.06 | SNP |
| chr6_32607224_C_T | 32607224 | 0.52 | 0.36 | 0.74 | 0.00034 | 0.09 | 0.06 | 0.11 | SNP |
| chr6_32612104_C_T | 32612104 | 1.49 | 1.20 | 1.86 | 0.00034 | 0.29 | 0.37 | 0.27 | SNP |
| chr6_32608417_G_A | 32608417 | 1.49 | 1.20 | 1.85 | 0.00034 | 0.29 | 0.37 | 0.27 | SNP |
| chr6_32636443_A_C | 32636443 | 1.48 | 1.19 | 1.84 | 0.00035 | 0.24 | 0.30 | 0.22 | SNP |
| chr6_32609126_C_T | 32609126 | 0.67 | 0.54 | 0.83 | 0.00035 | 0.46 | 0.39 | 0.48 | SNP |
| chr6_32520621_G_A | 32520621 | 1.47 | 1.19 | 1.82 | 0.00035 | 0.32 | 0.40 | 0.29 | SNP |
| chr6_32627485_C_T | 32627485 | 0.65 | 0.51 | 0.82 | 0.00035 | 0.43 | 0.36 | 0.45 | SNP |
| chr6_32149537_C_G | 32149537 | 1.76 | 1.29 | 2.40 | 0.00035 | 0.17 | 0.19 | 0.16 | SNP |
| chr6_32603487_C_T | 32603487 | 0.67 | 0.54 | 0.84 | 0.00035 | 0.45 | 0.38 | 0.47 | SNP |
| chr6_32710010_G_T | 32710010 | 0.68 | 0.55 | 0.84 | 0.00036 | 0.31 | 0.25 | 0.33 | SNP |
| chr6_32605920_A_G | 32605920 | 0.67 | 0.54 | 0.84 | 0.00036 | 0.45 | 0.38 | 0.47 | SNP |
| chr6_32608483_T_C | 32608483 | 1.49 | 1.19 | 1.85 | 0.00037 | 0.30 | 0.37 | 0.27 | SNP |

| | | | | | | | | | |
|---|---|---|---|---|---|---|---|---|---|
| chr6_31910929_G_A | 31910929 | 0.53 | 0.37 | 0.74 | 0.00038 | 0.08 | 0.05 | 0.09 | SNP |
| chr6_32606176_T_A | 32606176 | 0.67 | 0.54 | 0.84 | 0.00038 | 0.45 | 0.38 | 0.47 | SNP |
| chr6_31903121_G_A | 31903121 | 0.44 | 0.28 | 0.68 | 0.00039 | 0.06 | 0.03 | 0.06 | SNP |
| chr6_32441150_C_T | 32441150 | 1.48 | 1.19 | 1.85 | 0.00039 | 0.30 | 0.38 | 0.28 | SNP |
| chr6_32634990_G_T | 32634990 | 0.53 | 0.37 | 0.75 | 0.00039 | 0.08 | 0.05 | 0.09 | SNP |
| chr6_32559124_T_C | 32559124 | 1.50 | 1.20 | 1.88 | 0.00040 | 0.28 | 0.36 | 0.26 | SNP |
| chr6_32917042_A_C | 32917042 | 1.75 | 1.28 | 2.38 | 0.00041 | 0.06 | 0.09 | 0.05 | SNP |
| chr6_32636172_C_T | 32636172 | 1.47 | 1.19 | 1.81 | 0.00041 | 0.24 | 0.30 | 0.22 | SNP |
| chr6_32636138_A_G | 32636138 | 1.47 | 1.19 | 1.82 | 0.00041 | 0.24 | 0.30 | 0.22 | SNP |
| chr6_32559212_A_G | 32559212 | 1.49 | 1.20 | 1.87 | 0.00041 | 0.29 | 0.36 | 0.27 | SNP |
| chr6_32609427_C_T | 32609427 | 0.67 | 0.54 | 0.84 | 0.00041 | 0.45 | 0.38 | 0.47 | SNP |
| chr6_33045558_G_A | 33045558 | 0.70 | 0.57 | 0.85 | 0.00041 | 0.25 | 0.18 | 0.27 | SNP |
| chr6_32152442_A_G | 32152442 | 1.75 | 1.28 | 2.39 | 0.00042 | 0.17 | 0.19 | 0.16 | SNP |
| chr6_32610008_C_T | 32610008 | 1.48 | 1.19 | 1.83 | 0.00043 | 0.30 | 0.38 | 0.28 | SNP |
| chr6_31325792_G_A | 31325792 | 1.53 | 1.21 | 1.94 | 0.00043 | 0.36 | 0.38 | 0.35 | SNP |
| chr6_32628944_C_G | 32628944 | 1.49 | 1.19 | 1.87 | 0.00043 | 0.36 | 0.44 | 0.34 | SNP |
| chr6_32605759_A_T | 32605759 | 0.68 | 0.55 | 0.84 | 0.00044 | 0.45 | 0.38 | 0.47 | SNP |
| chr6_32439753_T_A | 32439753 | 0.65 | 0.51 | 0.82 | 0.00045 | 0.30 | 0.26 | 0.32 | SNP |
| chr6_32550591_G_A | 32550591 | 0.44 | 0.27 | 0.68 | 0.00046 | 0.06 | 0.03 | 0.06 | SNP |
| chr6_32610968_A_G | 32610968 | 1.49 | 1.19 | 1.85 | 0.00047 | 0.27 | 0.34 | 0.25 | SNP |
| chr6_31326703_T_C | 31326703 | 2.30 | 1.44 | 3.66 | 0.00047 | 0.12 | 0.13 | 0.12 | SNP |
| chr6_32607221_C_G | 32607221 | 0.53 | 0.37 | 0.75 | 0.00048 | 0.09 | 0.06 | 0.10 | SNP |
| chr6_32612326_C_T | 32612326 | 1.48 | 1.19 | 1.84 | 0.00049 | 0.29 | 0.37 | 0.27 | SNP |
| chr6_32611373_A_G | 32611373 | 0.67 | 0.53 | 0.84 | 0.00049 | 0.49 | 0.42 | 0.51 | SNP |
| chr6_31326074_A_T | 31326074 | 2.29 | 1.44 | 3.65 | 0.00050 | 0.12 | 0.13 | 0.11 | SNP |
| chr6_32629269_C_T | 32629269 | 1.47 | 1.18 | 1.83 | 0.00051 | 0.23 | 0.29 | 0.22 | SNP |
| chr6_32612161_C_A | 32612161 | 1.48 | 1.19 | 1.84 | 0.00051 | 0.29 | 0.37 | 0.27 | SNP |
| chr6_32147696_G_A | 32147696 | 1.74 | 1.27 | 2.38 | 0.00051 | 0.17 | 0.18 | 0.16 | SNP |
| chr6_32634103_C_T | 32634103 | 0.68 | 0.54 | 0.84 | 0.00052 | 0.45 | 0.38 | 0.47 | SNP |
| chr6_32608967_A_G | 32608967 | 0.54 | 0.38 | 0.76 | 0.00052 | 0.09 | 0.06 | 0.10 | SNP |
| chr6_32604108_T_C | 32604108 | 1.47 | 1.18 | 1.82 | 0.00052 | 0.30 | 0.38 | 0.28 | SNP |
| chr6_32612166_C_T | 32612166 | 1.48 | 1.18 | 1.84 | 0.00053 | 0.29 | 0.37 | 0.27 | SNP |
| chr6_32611769_G_C | 32611769 | 1.50 | 1.19 | 1.89 | 0.00053 | 0.31 | 0.39 | 0.29 | SNP |
| chr6_32609195_G_A | 32609195 | 0.55 | 0.38 | 0.76 | 0.00053 | 0.08 | 0.05 | 0.09 | SNP |
| chr6_32611472_G_A | 32611472 | 0.68 | 0.54 | 0.85 | 0.00056 | 0.46 | 0.39 | 0.48 | SNP |
| chr6_32606732_G_A | 32606732 | 1.46 | 1.18 | 1.81 | 0.00056 | 0.30 | 0.38 | 0.28 | SNP |
| chr6_32611510_C_G | 32611510 | 0.68 | 0.55 | 0.85 | 0.00056 | 0.46 | 0.40 | 0.48 | SNP |
| chr6_31430065_C_T | 31430065 | 2.33 | 1.44 | 3.78 | 0.00057 | 0.12 | 0.13 | 0.11 | SNP |
| chr6_32611265_C_T | 32611265 | 1.47 | 1.18 | 1.83 | 0.00057 | 0.29 | 0.36 | 0.27 | SNP |
| chr6_32184066_G_T | 32184066 | 1.39 | 1.15 | 1.67 | 0.00058 | 0.42 | 0.47 | 0.41 | SNP |
| chr6_32148031_A_G | 32148031 | 1.73 | 1.26 | 2.36 | 0.00058 | 0.17 | 0.18 | 0.16 | SNP |
| chr6_32612279_T_C | 32612279 | 1.47 | 1.18 | 1.82 | 0.00059 | 0.29 | 0.37 | 0.27 | SNP |
| chr6_32261940_G_C | 32261940 | 0.42 | 0.24 | 0.67 | 0.00059 | 0.04 | 0.02 | 0.05 | SNP |
| chr6_32500957_A_T | 32500957 | 0.57 | 0.41 | 0.78 | 0.00060 | 0.19 | 0.15 | 0.20 | SNP |
| chr6_32611403_G_A | 32611403 | 1.47 | 1.18 | 1.83 | 0.00060 | 0.27 | 0.35 | 0.25 | SNP |
| chr6_32710407_T_C | 32710407 | 1.81 | 1.29 | 2.54 | 0.00061 | 0.06 | 0.10 | 0.05 | SNP |
| chr6_32611759_T_G | 32611759 | 0.55 | 0.39 | 0.77 | 0.00061 | 0.08 | 0.05 | 0.09 | SNP |
| chr6_32164587_C_T | 32164587 | 0.42 | 0.25 | 0.67 | 0.00062 | 0.04 | 0.02 | 0.05 | SNP |
| chr6_32604059_A_G | 32604059 | 1.46 | 1.17 | 1.81 | 0.00062 | 0.30 | 0.37 | 0.28 | SNP |
| chr6_32192617_T_C | 32192617 | 0.46 | 0.29 | 0.70 | 0.00063 | 0.05 | 0.03 | 0.06 | SNP |
| chr6_32521591_C_T | 32521591 | 1.47 | 1.18 | 1.82 | 0.00063 | 0.32 | 0.40 | 0.30 | SNP |
| chr6_32610300_C_T | 32610300 | 0.55 | 0.38 | 0.77 | 0.00063 | 0.08 | 0.05 | 0.09 | SNP |
| chr6_31324375_G_T | 31324375 | 0.53 | 0.36 | 0.75 | 0.00066 | 0.09 | 0.06 | 0.09 | SNP |
| chr6_32636063_G_A | 32636063 | 1.44 | 1.17 | 1.78 | 0.00066 | 0.24 | 0.31 | 0.22 | SNP |
| chr6_32612041_A_G | 32612041 | 1.47 | 1.18 | 1.83 | 0.00067 | 0.29 | 0.37 | 0.27 | SNP |
| chr6_31127562_C_T | 31127562 | 1.60 | 1.22 | 2.10 | 0.00069 | 0.20 | 0.21 | 0.19 | SNP |
| chr6_32606942_G_A | 32606942 | 1.45 | 1.17 | 1.80 | 0.00070 | 0.30 | 0.38 | 0.28 | SNP |
| chr6_32973263_T_A | 32973263 | 1.32 | 1.12 | 1.55 | 0.00070 | 0.46 | 0.50 | 0.44 | SNP |
| chr6_31236051_A_G | 31236051 | 1.37 | 1.14 | 1.65 | 0.00071 | 0.49 | 0.51 | 0.48 | SNP |
| chr6_31606756_A_T | 31606756 | 0.60 | 0.44 | 0.80 | 0.00072 | 0.10 | 0.07 | 0.11 | SNP |
| chr6_32604151_C_T | 32604151 | 1.46 | 1.17 | 1.81 | 0.00072 | 0.30 | 0.37 | 0.28 | SNP |
| chr6_32270222_C_A | 32270222 | 0.43 | 0.26 | 0.69 | 0.00072 | 0.04 | 0.02 | 0.05 | SNP |
| chr6_32636154_C_G | 32636154 | 1.44 | 1.17 | 1.79 | 0.00072 | 0.24 | 0.30 | 0.22 | SNP |
| chr6_32611760_G_T | 32611760 | 1.47 | 1.17 | 1.83 | 0.00073 | 0.29 | 0.37 | 0.27 | SNP |
| chr6_32612241_A_T | 32612241 | 1.46 | 1.17 | 1.82 | 0.00073 | 0.29 | 0.37 | 0.27 | SNP |
| chr6_32559702_A_G | 32559702 | 1.60 | 1.22 | 2.10 | 0.00073 | 0.21 | 0.28 | 0.19 | SNP |
| chr6_32550819_C_A | 32550819 | 1.46 | 1.17 | 1.82 | 0.00074 | 0.29 | 0.37 | 0.27 | SNP |
| chr6_31112737_C_A | 31112737 | 1.67 | 1.24 | 2.25 | 0.00074 | 0.19 | 0.22 | 0.19 | SNP |
| chr6_32410137_C_T | 32410137 | 1.42 | 1.16 | 1.73 | 0.00075 | 0.28 | 0.31 | 0.28 | SNP |
| chr6_31902645_A_G | 31902645 | 0.47 | 0.30 | 0.72 | 0.00075 | 0.05 | 0.03 | 0.06 | SNP |
| chr6_31324750_G_A | 31324750 | 0.54 | 0.37 | 0.76 | 0.00075 | 0.09 | 0.06 | 0.10 | SNP |
| chr6_32627635_G_T | 32627635 | 0.68 | 0.55 | 0.85 | 0.00075 | 0.45 | 0.38 | 0.46 | SNP |
| chr6_32972903_G_A | 32972903 | 1.43 | 1.16 | 1.76 | 0.00075 | 0.14 | 0.19 | 0.13 | SNP |
| chr6_31924880_G_A | 31924880 | 0.55 | 0.39 | 0.77 | 0.00076 | 0.08 | 0.05 | 0.09 | SNP |
| chr6_32608946_G_A | 32608946 | 0.56 | 0.39 | 0.77 | 0.00076 | 0.08 | 0.05 | 0.09 | SNP |
| chr6_32633816_C_G | 32633816 | 1.39 | 1.15 | 1.69 | 0.00076 | 0.38 | 0.42 | 0.36 | SNP |
| chr6_32612096_A_G | 32612096 | 0.56 | 0.39 | 0.77 | 0.00076 | 0.08 | 0.05 | 0.09 | SNP |
| chr6_32374595_A_G | 32374595 | 0.57 | 0.40 | 0.78 | 0.00077 | 0.18 | 0.15 | 0.19 | SNP |
| chr6_32375796_A_T | 32375796 | 0.57 | 0.40 | 0.78 | 0.00077 | 0.18 | 0.15 | 0.19 | SNP |
| chr6_32145205_C_T | 32145205 | 1.72 | 1.25 | 2.35 | 0.00077 | 0.18 | 0.21 | 0.17 | SNP |
| chr6_32604259_C_T | 32604259 | 1.46 | 1.17 | 1.81 | 0.00077 | 0.30 | 0.37 | 0.28 | SNP |
| chr6_32628098_G_C | 32628098 | 0.45 | 0.27 | 0.70 | 0.00079 | 0.05 | 0.03 | 0.05 | SNP |
| chr6_31373445_T_C | 31373445 | 2.21 | 1.38 | 3.51 | 0.00079 | 0.03 | 0.04 | 0.02 | SNP |
| chr6_32609874_T_G | 32609874 | 0.56 | 0.39 | 0.78 | 0.00079 | 0.08 | 0.05 | 0.09 | SNP |
| chr6_32610075_A_G | 32610075 | 0.56 | 0.39 | 0.78 | 0.00079 | 0.08 | 0.05 | 0.09 | SNP |
| chr6_32610313_C_G | 32610313 | 0.56 | 0.39 | 0.78 | 0.00079 | 0.08 | 0.05 | 0.09 | SNP |
| chr6_32608934_A_T | 32608934 | 0.56 | 0.39 | 0.78 | 0.00080 | 0.08 | 0.05 | 0.09 | SNP |
| chr6_31248382_C_T | 31248382 | 0.57 | 0.40 | 0.78 | 0.00080 | 0.10 | 0.06 | 0.11 | SNP |
| chr6_32612008_T_C | 32612008 | 1.47 | 1.17 | 1.84 | 0.00081 | 0.29 | 0.36 | 0.27 | SNP |
| chr6_32605336_G_A | 32605336 | 1.44 | 1.16 | 1.79 | 0.00081 | 0.30 | 0.38 | 0.28 | SNP |
| chr6_32605418_G_A | 32605418 | 1.45 | 1.17 | 1.79 | 0.00081 | 0.30 | 0.38 | 0.28 | SNP |
| chr6_32612251_T_G | 32612251 | 1.45 | 1.17 | 1.81 | 0.00082 | 0.29 | 0.37 | 0.27 | SNP |
| chr6_32605101_A_G | 32605101 | 1.44 | 1.16 | 1.79 | 0.00083 | 0.30 | 0.38 | 0.28 | SNP |
| chr6_32611215_C_T | 32611215 | 0.56 | 0.39 | 0.78 | 0.00083 | 0.08 | 0.05 | 0.09 | SNP |
| chr6_32610688_G_C | 32610688 | 0.56 | 0.39 | 0.78 | 0.00083 | 0.08 | 0.05 | 0.09 | SNP |
| chr6_32184094_G_A | 32184094 | 1.37 | 1.14 | 1.64 | 0.00084 | 0.43 | 0.48 | 0.41 | SNP |
| chr6_33556605_G_C | 33556605 | 0.69 | 0.55 | 0.86 | 0.00084 | 0.21 | 0.16 | 0.22 | SNP |
| chr6_32611260_A_G | 32611260 | 1.45 | 1.17 | 1.81 | 0.00085 | 0.29 | 0.36 | 0.27 | SNP |
| chr6_32610696_G_A | 32610696 | 0.56 | 0.39 | 0.78 | 0.00085 | 0.08 | 0.05 | 0.09 | SNP |
| chr6_32609380_C_T | 32609380 | 1.52 | 1.19 | 1.94 | 0.00087 | 0.36 | 0.43 | 0.34 | SNP |
| chr6_32609341_T_C | 32609341 | 0.68 | 0.55 | 0.85 | 0.00089 | 0.47 | 0.41 | 0.49 | SNP |
| chr6_32188297_G_C | 32188297 | 1.38 | 1.14 | 1.68 | 0.00090 | 0.29 | 0.35 | 0.28 | SNP |
| chr6_32606960_A_T | 32606960 | 0.56 | 0.39 | 0.78 | 0.00091 | 0.08 | 0.05 | 0.09 | SNP |
| chr6_32633617_A_G | 32633617 | 0.69 | 0.55 | 0.86 | 0.00091 | 0.44 | 0.38 | 0.46 | SNP |
| chr6_31902289_C_G | 31902289 | 0.48 | 0.30 | 0.73 | 0.00093 | 0.05 | 0.03 | 0.06 | SNP |
| chr6_32184221_G_A | 32184221 | 1.36 | 1.13 | 1.64 | 0.00093 | 0.43 | 0.48 | 0.41 | SNP |
| chr6_31919956_T_C | 31919956 | 0.56 | 0.39 | 0.78 | 0.00094 | 0.08 | 0.05 | 0.09 | SNP |
| chr6_31916951_T_C | 31916951 | 0.56 | 0.40 | 0.78 | 0.00095 | 0.08 | 0.05 | 0.09 | SNP |
| chr6_31919917_T_C | 31919917 | 0.56 | 0.40 | 0.78 | 0.00095 | 0.08 | 0.05 | 0.09 | SNP |
| chr6_32409056_G_A | 32409056 | 1.41 | 1.15 | 1.72 | 0.00095 | 0.29 | 0.32 | 0.28 | SNP |
| chr6_32785275_G_A | 32785275 | 0.59 | 0.43 | 0.80 | 0.00097 | 0.09 | 0.06 | 0.10 | SNP |
| chr6_32605861_A_G | 32605861 | 0.56 | 0.39 | 0.78 | 0.00097 | 0.08 | 0.05 | 0.09 | SNP |
| chr6_32303160_G_T | 32303160 | 0.44 | 0.26 | 0.70 | 0.00097 | 0.04 | 0.02 | 0.05 | SNP |
| chr6_32611418_A_C | 32611418 | 1.47 | 1.17 | 1.84 | 0.00099 | 0.28 | 0.35 | 0.26 | SNP |
| chr6_32605950_C_T | 32605950 | 0.56 | 0.39 | 0.78 | 0.00101 | 0.08 | 0.05 | 0.09 | SNP |
| chr6_32611427_T_A | 32611427 | 1.46 | 1.17 | 1.84 | 0.00102 | 0.28 | 0.35 | 0.26 | SNP |
| chr6_32442296_C_T | 32442296 | 0.67 | 0.52 | 0.85 | 0.00102 | 0.29 | 0.24 | 0.30 | SNP |
| chr6_32606742_A_G | 32606742 | 0.56 | 0.39 | 0.78 | 0.00103 | 0.08 | 0.05 | 0.09 | SNP |
| chr6_32633285_T_C | 32633285 | 0.70 | 0.57 | 0.87 | 0.00104 | 0.35 | 0.30 | 0.37 | SNP |
| chr6_33169895_C_G | 33169895 | 1.49 | 1.17 | 1.89 | 0.00104 | 0.11 | 0.14 | 0.10 | SNP |
| chr6_32611709_T_C | 32611709 | 0.56 | 0.40 | 0.79 | 0.00105 | 0.08 | 0.05 | 0.09 | SNP |
| xHLA_2field_DQB1_06_04 | 32627244 | 0.43 | 0.25 | 0.69 | 0.00105 | 0.05 | 0.02 | 0.05 | HLA (xHLA) |
| chr6_32559710_C_T | 32559710 | 1.57 | 1.20 | 2.06 | 0.00105 | 0.21 | 0.28 | 0.19 | SNP |
| chr6_32629467_G_A | 32629467 | 1.44 | 1.16 | 1.79 | 0.00106 | 0.37 | 0.44 | 0.35 | SNP |
| chr6_32781121_G_C | 32781121 | 0.60 | 0.43 | 0.81 | 0.00106 | 0.09 | 0.06 | 0.10 | SNP |
| chr6_32603405_C_T | 32603405 | 0.56 | 0.39 | 0.79 | 0.00106 | 0.08 | 0.05 | 0.09 | SNP |
| chr6_31323506_C_T | 31323506 | 2.11 | 1.35 | 3.31 | 0.00107 | 0.12 | 0.13 | 0.12 | SNP |
| chr6_33130430_G_A | 33130430 | 0.69 | 0.55 | 0.86 | 0.00108 | 0.18 | 0.14 | 0.20 | SNP |
| chr6_32628420_C_T | 32628420 | 1.45 | 1.16 | 1.80 | 0.00110 | 0.36 | 0.44 | 0.34 | SNP |
| chr6_32607017_T_A | 32607017 | 0.56 | 0.39 | 0.79 | 0.00110 | 0.08 | 0.05 | 0.09 | SNP |
| chr6_32606676_T_C | 32606676 | 0.56 | 0.39 | 0.79 | 0.00111 | 0.08 | 0.05 | 0.09 | SNP |
| chr6_32629500_C_A | 32629500 | 1.45 | 1.16 | 1.82 | 0.00112 | 0.19 | 0.25 | 0.18 | SNP |
| chr6_32610403_C_A | 32610403 | 1.44 | 1.16 | 1.80 | 0.00113 | 0.30 | 0.37 | 0.28 | SNP |
| chr6_32628556_C_T | 32628556 | 1.45 | 1.16 | 1.81 | 0.00113 | 0.20 | 0.26 | 0.19 | SNP |
| chr6_31325116_T_G | 31325116 | 1.54 | 1.19 | 2.01 | 0.00113 | 0.34 | 0.36 | 0.33 | SNP |
| chr6_32606052_T_G | 32606052 | 1.43 | 1.15 | 1.78 | 0.00114 | 0.31 | 0.38 | 0.29 | SNP |
| chr6_31125978_A_T | 31125978 | 1.64 | 1.21 | 2.21 | 0.00116 | 0.20 | 0.22 | 0.19 | SNP |
| chr6_32604384_G_A | 32604384 | 0.56 | 0.39 | 0.79 | 0.00116 | 0.08 | 0.05 | 0.09 | SNP |
| chr6_32636364_G_A | 32636364 | 1.37 | 1.13 | 1.66 | 0.00117 | 0.38 | 0.42 | 0.37 | SNP |
| chr6_32526702_C_T | 32526702 | 0.56 | 0.39 | 0.79 | 0.00118 | 0.08 | 0.05 | 0.09 | SNP |
| chr6_32608820_T_C | 32608820 | 0.70 | 0.56 | 0.87 | 0.00120 | 0.46 | 0.39 | 0.48 | SNP |
| chr6_32605419_G_C | 32605419 | 1.43 | 1.15 | 1.78 | 0.00120 | 0.30 | 0.38 | 0.28 | SNP |
| chr6_32785284_T_C | 32785284 | 0.60 | 0.44 | 0.81 | 0.00120 | 0.09 | 0.06 | 0.10 | SNP |
| chr6_32606214_C_T | 32606214 | 1.78 | 1.25 | 2.52 | 0.00121 | 0.04 | 0.06 | 0.04 | SNP |
| chr6_31935464_C_G | 31935464 | 0.39 | 0.21 | 0.67 | 0.00121 | 0.04 | 0.02 | 0.04 | SNP |
| chr6_29891617_A_G | 29891617 | 1.35 | 1.13 | 1.62 | 0.00123 | 0.48 | 0.51 | 0.48 | SNP |
| chr6_33137689_C_T | 33137689 | 1.48 | 1.16 | 1.87 | 0.00123 | 0.11 | 0.14 | 0.10 | SNP |
| chr6_32608425_C_A | 32608425 | 0.57 | 0.40 | 0.79 | 0.00125 | 0.08 | 0.05 | 0.09 | SNP |
| chr6_31928306_A_G | 31928306 | 0.57 | 0.40 | 0.79 | 0.00126 | 0.08 | 0.05 | 0.09 | SNP |
| chr6_32780914_G_A | 32780914 | 0.60 | 0.44 | 0.81 | 0.00130 | 0.09 | 0.06 | 0.10 | SNP |
| chr6_32610860_G_T | 32610860 | 1.44 | 1.15 | 1.79 | 0.00131 | 0.29 | 0.36 | 0.27 | SNP |
| chr6_32609456_C_T | 32609456 | 1.43 | 1.15 | 1.78 | 0.00133 | 0.30 | 0.37 | 0.28 | SNP |
| chr6_32439915_T_C | 32439915 | 0.67 | 0.52 | 0.85 | 0.00134 | 0.29 | 0.25 | 0.30 | SNP |
| chr6_31383130_T_A | 31383130 | 0.63 | 0.47 | 0.83 | 0.00135 | 0.12 | 0.09 | 0.12 | SNP |
| chr6_32559648_G_T | 32559648 | 1.54 | 1.18 | 2.00 | 0.00135 | 0.20 | 0.27 | 0.18 | SNP |
| chr6_31325115_G_A | 31325115 | 1.53 | 1.18 | 1.99 | 0.00136 | 0.34 | 0.36 | 0.34 | SNP |
| chr6_32171075_A_G | 32171075 | 1.60 | 1.20 | 2.13 | 0.00138 | 0.18 | 0.20 | 0.18 | SNP |
| chr6_32636385_A_T | 32636385 | 1.36 | 1.13 | 1.65 | 0.00138 | 0.38 | 0.42 | 0.36 | SNP |
| chr6_31323416_G_C | 31323416 | 2.08 | 1.32 | 3.24 | 0.00139 | 0.12 | 0.13 | 0.12 | SNP |
| chr6_32154998_C_T | 32154998 | 1.62 | 1.20 | 2.17 | 0.00141 | 0.18 | 0.20 | 0.18 | SNP |
| chr6_32610976_A_C | 32610976 | 0.58 | 0.41 | 0.80 | 0.00141 | 0.08 | 0.05 | 0.09 | SNP |
| chr6_32189032_T_C | 32189032 | 1.36 | 1.13 | 1.65 | 0.00142 | 0.29 | 0.35 | 0.28 | SNP |

| | | | | | | | | | |
|---|---|---|---|---|---|---|---|---|---|
| chr6_32406122_G_A | 32406122 | 1.90 | 1.27 | 2.81 | 0.00146 | 0.03 | 0.05 | 0.03 | SNP |
| chr6_31325092_G_A | 31325092 | 1.53 | 1.18 | 1.98 | 0.00147 | 0.34 | 0.36 | 0.33 | SNP |
| chr6_31915532_C_T | 31915532 | 0.40 | 0.22 | 0.69 | 0.00149 | 0.04 | 0.02 | 0.04 | SNP |
| chr6_32136029_A_G | 32136029 | 2.05 | 1.31 | 3.17 | 0.00149 | 0.03 | 0.05 | 0.03 | SNP |
| chr6_32185796_C_T | 32185796 | 1.98 | 1.29 | 3.00 | 0.00149 | 0.03 | 0.05 | 0.03 | SNP |
| chr6_31238851_C_T | 31238851 | 1.58 | 1.19 | 2.10 | 0.00150 | 0.32 | 0.33 | 0.31 | SNP |
| chr6_31323116_C_T | 31323116 | 1.96 | 1.28 | 2.95 | 0.00150 | 0.03 | 0.05 | 0.03 | SNP |
| chr6_32408932_G_A | 32408932 | 1.90 | 1.27 | 2.80 | 0.00151 | 0.03 | 0.05 | 0.03 | SNP |
| chr6_32411573_C_T | 32411573 | 1.90 | 1.27 | 2.80 | 0.00151 | 0.03 | 0.05 | 0.03 | SNP |
| chr6_32406473_G_A | 32406473 | 1.49 | 1.16 | 1.90 | 0.00153 | 0.21 | 0.24 | 0.21 | SNP |
| chr6_32782018_C_T | 32782018 | 0.60 | 0.44 | 0.82 | 0.00155 | 0.10 | 0.07 | 0.10 | SNP |
| chr6_32557301_T_C | 32557301 | 0.40 | 0.22 | 0.69 | 0.00156 | 0.03 | 0.02 | 0.04 | SNP |
| chr6_32610994_C_A | 32610994 | 1.44 | 1.15 | 1.80 | 0.00157 | 0.27 | 0.33 | 0.25 | SNP |
| chr6_31122500_G_A | 31122500 | 1.67 | 1.21 | 2.29 | 0.00157 | 0.19 | 0.22 | 0.18 | SNP |
| chr6_32782112_C_T | 32782112 | 2.07 | 1.31 | 3.24 | 0.00160 | 0.02 | 0.04 | 0.02 | SNP |
| chr6_32633819_A_G | 32633819 | 0.70 | 0.56 | 0.87 | 0.00161 | 0.50 | 0.45 | 0.51 | SNP |
| chr6_32607270_A_G | 32607270 | 0.57 | 0.40 | 0.80 | 0.00162 | 0.08 | 0.05 | 0.09 | SNP |
| chr6_31465047_G_T | 31465047 | 2.15 | 1.33 | 3.46 | 0.00163 | 0.12 | 0.13 | 0.12 | SNP |
| chr6_31116210_G_A | 31116210 | 1.49 | 1.16 | 1.92 | 0.00163 | 0.24 | 0.25 | 0.24 | SNP |
| chr6_32789400_A_G | 32789400 | 0.44 | 0.26 | 0.72 | 0.00168 | 0.04 | 0.02 | 0.05 | SNP |
| chr6_32628913_G_T | 32628913 | 1.55 | 1.18 | 2.04 | 0.00169 | 0.18 | 0.24 | 0.16 | SNP |
| chr6_32188603_C_T | 32188603 | 1.36 | 1.12 | 1.65 | 0.00169 | 0.29 | 0.34 | 0.28 | SNP |
| chr6_32611229_T_G | 32611229 | 0.71 | 0.57 | 0.88 | 0.00175 | 0.46 | 0.39 | 0.48 | SNP |
| chr6_32608809_T_C | 32608809 | 1.43 | 1.14 | 1.79 | 0.00176 | 0.27 | 0.35 | 0.25 | SNP |
| chr6_32610997_C_G | 32610997 | 1.43 | 1.14 | 1.79 | 0.00176 | 0.27 | 0.33 | 0.25 | SNP |
| chr6_31237449_C_T | 31237449 | 1.53 | 1.17 | 1.99 | 0.00177 | 0.32 | 0.34 | 0.31 | SNP |
| chr6_31914180_G_A | 31914180 | 0.58 | 0.41 | 0.81 | 0.00178 | 0.08 | 0.05 | 0.09 | SNP |
| chr6_31326948_C_A | 31326948 | 0.64 | 0.48 | 0.84 | 0.00179 | 0.11 | 0.08 | 0.11 | SNP |
| chr6_31934372_C_T | 31934372 | 0.60 | 0.43 | 0.82 | 0.00182 | 0.11 | 0.07 | 0.12 | SNP |
| chr6_32559444_C_T | 32559444 | 0.61 | 0.44 | 0.82 | 0.00185 | 0.09 | 0.06 | 0.10 | SNP |
| chr6_33281843_A_T | 33281843 | 1.33 | 1.11 | 1.59 | 0.00186 | 0.34 | 0.39 | 0.32 | SNP |
| chr6_31906865_C_T | 31906865 | 0.58 | 0.41 | 0.81 | 0.00187 | 0.08 | 0.05 | 0.09 | SNP |
| chr6_32520460_C_T | 32520460 | 1.40 | 1.13 | 1.73 | 0.00187 | 0.32 | 0.39 | 0.30 | SNP |
| chr6_32629764_C_T | 32629764 | 1.41 | 1.13 | 1.75 | 0.00188 | 0.38 | 0.45 | 0.36 | SNP |
| chr6_31905130_G_A | 31905130 | 0.58 | 0.41 | 0.81 | 0.00188 | 0.08 | 0.05 | 0.09 | SNP |
| chr6_31905328_G_T | 31905328 | 0.58 | 0.41 | 0.81 | 0.00188 | 0.08 | 0.05 | 0.09 | SNP |
| chr6_31907147_T_C | 31907147 | 0.58 | 0.41 | 0.81 | 0.00188 | 0.08 | 0.05 | 0.09 | SNP |
| chr6_31907168_C_T | 31907168 | 0.58 | 0.41 | 0.81 | 0.00188 | 0.08 | 0.05 | 0.09 | SNP |
| chr6_32781076_C_A | 32781076 | 0.61 | 0.45 | 0.83 | 0.00190 | 0.09 | 0.06 | 0.10 | SNP |
| chr6_32633971_A_G | 32633971 | 1.43 | 1.14 | 1.78 | 0.00190 | 0.19 | 0.25 | 0.18 | SNP |
| chr6_31326683_G_A | 31326683 | 0.64 | 0.48 | 0.84 | 0.00191 | 0.10 | 0.08 | 0.11 | SNP |
| chr6_31932177_C_T | 31932177 | 0.61 | 0.44 | 0.83 | 0.00196 | 0.11 | 0.07 | 0.12 | SNP |
| chr6_29893131_T_C | 29893131 | 0.37 | 0.19 | 0.66 | 0.00196 | 0.03 | 0.01 | 0.04 | SNP |
| chr6_31326080_T_C | 31326080 | 0.61 | 0.44 | 0.83 | 0.00205 | 0.09 | 0.07 | 0.09 | SNP |
| chr6_31326929_A_C | 31326929 | 0.64 | 0.48 | 0.85 | 0.00207 | 0.11 | 0.08 | 0.11 | SNP |
| chr6_31321211_C_T | 31321211 | 0.75 | 0.63 | 0.90 | 0.00208 | 0.41 | 0.37 | 0.42 | SNP |
| chr6_32410576_C_T | 32410576 | 1.37 | 1.12 | 1.68 | 0.00209 | 0.28 | 0.31 | 0.28 | SNP |
| chr6_32608856_T_C | 32608856 | 1.42 | 1.13 | 1.77 | 0.00213 | 0.28 | 0.35 | 0.27 | SNP |
| chr6_31947086_G_A | 31947086 | 0.63 | 0.46 | 0.84 | 0.00214 | 0.11 | 0.07 | 0.12 | SNP |
| chr6_31125705_C_G | 31125705 | 1.54 | 1.17 | 2.02 | 0.00215 | 0.20 | 0.21 | 0.19 | SNP |
| chr6_31473746_G_A | 31473746 | 2.12 | 1.31 | 3.43 | 0.00223 | 0.12 | 0.13 | 0.12 | SNP |
| chr6_32629859_G_A | 32629859 | 0.71 | 0.57 | 0.88 | 0.00225 | 0.48 | 0.43 | 0.50 | SNP |
| chr6_31322108_T_G | 31322108 | 1.98 | 1.27 | 3.05 | 0.00226 | 0.03 | 0.04 | 0.02 | SNP |
| chr6_31325745_C_T | 31325745 | 1.33 | 1.11 | 1.59 | 0.00228 | 0.47 | 0.52 | 0.45 | SNP |
| xHLA_2field_B_08_01 | 31321652 | 2.07 | 1.30 | 3.31 | 0.00229 | 0.12 | 0.13 | 0.11 | HLA (xHLA) |
| chr6_32550931_C_A | 32550931 | 1.42 | 1.13 | 1.79 | 0.00237 | 0.23 | 0.30 | 0.21 | SNP |
| chr6_32605646_T_G | 32605646 | 1.40 | 1.13 | 1.73 | 0.00241 | 0.31 | 0.38 | 0.29 | SNP |
| chr6_31325317_C_T | 31325317 | 0.64 | 0.48 | 0.85 | 0.00243 | 0.10 | 0.08 | 0.11 | SNP |
| chr6_32975381_G_A | 32975381 | 0.76 | 0.64 | 0.91 | 0.00243 | 0.40 | 0.35 | 0.41 | SNP |
| chr6_33281842_G_T | 33281842 | 1.32 | 1.10 | 1.58 | 0.00248 | 0.34 | 0.39 | 0.32 | SNP |
| chr6_32804798_G_A | 32804798 | 1.35 | 1.11 | 1.63 | 0.00249 | 0.28 | 0.35 | 0.26 | SNP |
| chr6_31935311_C_T | 31935311 | 0.62 | 0.45 | 0.84 | 0.00252 | 0.11 | 0.07 | 0.12 | SNP |
| chr6_32635713_C_T | 32635713 | 0.72 | 0.58 | 0.89 | 0.00253 | 0.47 | 0.40 | 0.49 | SNP |
| chr6_32604660_C_T | 32604660 | 0.60 | 0.43 | 0.83 | 0.00258 | 0.09 | 0.06 | 0.10 | SNP |
| chr6_31864304_A_G | 31864304 | 1.45 | 1.14 | 1.84 | 0.00260 | 0.37 | 0.39 | 0.37 | SNP |
| chr6_31322611_C_T | 31322611 | 1.96 | 1.25 | 3.02 | 0.00261 | 0.03 | 0.04 | 0.02 | SNP |
| chr6_31323455_A_G | 31323455 | 1.96 | 1.25 | 3.02 | 0.00261 | 0.03 | 0.04 | 0.02 | SNP |
| chr6_32629347_T_C | 32629347 | 1.40 | 1.13 | 1.75 | 0.00262 | 0.37 | 0.44 | 0.35 | SNP |
| chr6_31378257_G_A | 31378257 | 1.96 | 1.25 | 3.02 | 0.00263 | 0.03 | 0.04 | 0.02 | SNP |
| chr6_31322987_C_T | 31322987 | 1.96 | 1.25 | 3.02 | 0.00263 | 0.03 | 0.04 | 0.02 | SNP |
| chr6_32627652_C_G | 32627652 | 1.39 | 1.12 | 1.73 | 0.00267 | 0.38 | 0.45 | 0.36 | SNP |
| chr6_31514247_A_G | 31514247 | 2.10 | 1.29 | 3.42 | 0.00268 | 0.12 | 0.13 | 0.12 | SNP |
| chr6_32629400_A_G | 32629400 | 1.40 | 1.12 | 1.74 | 0.00269 | 0.37 | 0.44 | 0.35 | SNP |
| chr6_32783365_T_C | 32783365 | 0.63 | 0.46 | 0.85 | 0.00269 | 0.09 | 0.07 | 0.10 | SNP |
| chr6_32629535_C_A | 32629535 | 1.40 | 1.12 | 1.73 | 0.00272 | 0.37 | 0.44 | 0.35 | SNP |
| chr6_31932209_G_A | 31932209 | 0.39 | 0.20 | 0.69 | 0.00273 | 0.04 | 0.02 | 0.04 | SNP |
| chr6_31240060_G_A | 31240060 | 1.50 | 1.15 | 1.95 | 0.00278 | 0.33 | 0.34 | 0.32 | SNP |
| chr6_31430010_G_A | 31430010 | 2.06 | 1.28 | 3.32 | 0.00283 | 0.12 | 0.13 | 0.12 | SNP |
| chr6_32803576_A_G | 32803576 | 1.55 | 1.16 | 2.05 | 0.00283 | 0.08 | 0.12 | 0.06 | SNP |
| chr6_31325764_C_G | 31325764 | 0.66 | 0.49 | 0.86 | 0.00289 | 0.11 | 0.09 | 0.12 | SNP |
| chr6_31930462_G_A | 31930462 | 0.67 | 0.52 | 0.87 | 0.00290 | 0.13 | 0.10 | 0.14 | SNP |
| chr6_32406704_G_T | 32406704 | 1.46 | 1.14 | 1.88 | 0.00292 | 0.21 | 0.23 | 0.21 | SNP |
| chr6_32608350_G_A | 32608350 | 0.58 | 0.40 | 0.82 | 0.00292 | 0.10 | 0.06 | 0.10 | SNP |
| chr6_32628845_G_A | 32628845 | 1.40 | 1.12 | 1.74 | 0.00292 | 0.37 | 0.44 | 0.35 | SNP |
| chr6_32809848_G_A | 32809848 | 1.41 | 1.12 | 1.77 | 0.00295 | 0.14 | 0.19 | 0.13 | SNP |
| chr6_31901773_G_A | 31901773 | 0.43 | 0.23 | 0.73 | 0.00295 | 0.03 | 0.02 | 0.04 | SNP |
| chr6_32190620_T_C | 32190620 | 0.75 | 0.61 | 0.90 | 0.00298 | 0.26 | 0.21 | 0.27 | SNP |
| chr6_29891850_A_G | 29891850 | 1.29 | 1.09 | 1.53 | 0.00299 | 0.43 | 0.45 | 0.42 | SNP |
| chr6_32606217_G_A | 32606217 | 1.39 | 1.12 | 1.73 | 0.00299 | 0.31 | 0.38 | 0.29 | SNP |
| chr6_29855331_G_A | 29855331 | 1.39 | 1.12 | 1.73 | 0.00305 | 0.19 | 0.21 | 0.19 | SNP |
| chr6_33365724_A_G | 33365724 | 0.59 | 0.41 | 0.83 | 0.00309 | 0.07 | 0.05 | 0.08 | SNP |
| chr6_32629331_A_G | 32629331 | 1.39 | 1.12 | 1.74 | 0.00311 | 0.36 | 0.44 | 0.34 | SNP |
| chr6_32785029_T_C | 32785029 | 0.63 | 0.47 | 0.85 | 0.00313 | 0.09 | 0.07 | 0.10 | SNP |
| chr6_32340596_T_A | 32340596 | 0.49 | 0.29 | 0.77 | 0.00316 | 0.04 | 0.02 | 0.05 | SNP |
| chr6_32976909_C_A | 32976909 | 0.79 | 0.67 | 0.92 | 0.00317 | 0.48 | 0.45 | 0.49 | SNP |
| chr6_32374382_C_A | 32374382 | 0.51 | 0.32 | 0.78 | 0.00317 | 0.05 | 0.03 | 0.05 | SNP |
| chr6_32374437_A_G | 32374437 | 0.51 | 0.32 | 0.78 | 0.00317 | 0.05 | 0.03 | 0.05 | SNP |
| chr6_32374640_G_A | 32374640 | 0.51 | 0.32 | 0.78 | 0.00317 | 0.05 | 0.03 | 0.05 | SNP |
| chr6_32713267_A_G | 32713267 | 1.49 | 1.14 | 1.94 | 0.00319 | 0.09 | 0.11 | 0.08 | SNP |
| xHLA_2field_B_40_01 | 31321652 | 0.62 | 0.45 | 0.85 | 0.00321 | 0.09 | 0.07 | 0.09 | HLA (xHLA) |
| chr6_32785035_A_G | 32785035 | 0.64 | 0.47 | 0.85 | 0.00328 | 0.09 | 0.07 | 0.10 | SNP |
| chr6_32609286_T_C | 32609286 | 0.73 | 0.59 | 0.90 | 0.00331 | 0.49 | 0.56 | 0.47 | SNP |
| chr6_33219142_A_C | 33219142 | 0.75 | 0.62 | 0.91 | 0.00331 | 0.34 | 0.28 | 0.35 | SNP |
| chr6_32604642_A_G | 32604642 | 1.42 | 1.12 | 1.78 | 0.00333 | 0.28 | 0.35 | 0.26 | SNP |
| chr6_32629509_A_C | 32629509 | 1.39 | 1.11 | 1.72 | 0.00335 | 0.37 | 0.44 | 0.36 | SNP |
| chr6_32793721_G_C | 32793721 | 1.64 | 1.17 | 2.27 | 0.00339 | 0.06 | 0.10 | 0.05 | SNP |
| chr6_32550587_T_G | 32550587 | 0.50 | 0.31 | 0.78 | 0.00341 | 0.05 | 0.03 | 0.05 | SNP |
| xHLA_2field_DRB1_04_01 | 32546552 | 1.48 | 1.14 | 1.92 | 0.00341 | 0.13 | 0.20 | 0.11 | HLA (xHLA) |
| chr6_31122482_G_A | 31122482 | 1.60 | 1.16 | 2.19 | 0.00342 | 0.19 | 0.22 | 0.19 | SNP |
| chr6_32793534_T_C | 32793534 | 1.63 | 1.17 | 2.27 | 0.00349 | 0.06 | 0.10 | 0.05 | SNP |
| chr6_33639760_C_G | 33639760 | 0.76 | 0.63 | 0.91 | 0.00350 | 0.33 | 0.28 | 0.35 | SNP |
| chr6_32627818_C_A | 32627818 | 1.39 | 1.11 | 1.73 | 0.00352 | 0.20 | 0.26 | 0.19 | SNP |
| chr6_31321360_G_A | 31321360 | 1.60 | 1.17 | 2.20 | 0.00354 | 0.19 | 0.21 | 0.18 | SNP |
| chr6_31241207_C_T | 31241207 | 0.74 | 0.61 | 0.91 | 0.00358 | 0.29 | 0.27 | 0.29 | SNP |
| chr6_31239577_C_A | 31239577 | 1.41 | 1.12 | 1.79 | 0.00358 | 0.48 | 0.51 | 0.47 | SNP |
| chr6_29895697_T_C | 29895697 | 0.78 | 0.66 | 0.92 | 0.00361 | 0.50 | 0.48 | 0.50 | SNP |
| chr6_32786516_C_T | 32786516 | 0.60 | 0.42 | 0.84 | 0.00363 | 0.07 | 0.05 | 0.08 | SNP |
| chr6_31241092_G_C | 31241092 | 1.37 | 1.11 | 1.69 | 0.00363 | 0.38 | 0.40 | 0.38 | SNP |
| chr6_31248432_A_G | 31248432 | 0.73 | 0.59 | 0.90 | 0.00363 | 0.47 | 0.46 | 0.47 | SNP |
| chr6_32498205_G_T | 32498205 | 1.38 | 1.11 | 1.71 | 0.00370 | 0.30 | 0.39 | 0.28 | SNP |
| chr6_32376788_C_T | 32376788 | 1.36 | 1.11 | 1.68 | 0.00370 | 0.45 | 0.50 | 0.44 | SNP |
| chr6_32783406_G_A | 32783406 | 0.60 | 0.42 | 0.84 | 0.00370 | 0.07 | 0.05 | 0.08 | SNP |
| chr6_32783450_C_G | 32783450 | 0.60 | 0.42 | 0.84 | 0.00370 | 0.07 | 0.05 | 0.08 | SNP |
| chr6_32322071_G_A | 32322071 | 1.90 | 1.22 | 2.92 | 0.00375 | 0.03 | 0.05 | 0.03 | SNP |
| chr6_30864279_C_T | 30864279 | 1.78 | 1.20 | 2.64 | 0.00377 | 0.12 | 0.13 | 0.11 | SNP |
| chr6_31321407_C_T | 31321407 | 0.66 | 0.50 | 0.87 | 0.00379 | 0.11 | 0.08 | 0.12 | SNP |
| chr6_32784676_C_T | 32784676 | 1.60 | 1.16 | 2.19 | 0.00383 | 0.06 | 0.10 | 0.05 | SNP |
| chr6_32407537_C_T | 32407537 | 0.49 | 0.29 | 0.77 | 0.00387 | 0.04 | 0.02 | 0.05 | SNP |
| chr6_31378864_A_G | 31378864 | 1.94 | 1.23 | 3.02 | 0.00387 | 0.03 | 0.04 | 0.02 | SNP |
| chr6_31244021_A_G | 31244021 | 1.49 | 1.14 | 1.96 | 0.00389 | 0.32 | 0.33 | 0.31 | SNP |
| chr6_31118019_T_C | 31118019 | 1.41 | 1.12 | 1.79 | 0.00394 | 0.26 | 0.27 | 0.26 | SNP |
| chr6_32781112_C_T | 32781112 | 1.63 | 1.17 | 2.26 | 0.00396 | 0.06 | 0.10 | 0.05 | SNP |
| chr6_32498206_A_T | 32498206 | 1.38 | 1.11 | 1.71 | 0.00404 | 0.30 | 0.39 | 0.28 | SNP |
| chr6_33636660_C_G | 33636660 | 1.33 | 1.09 | 1.61 | 0.00406 | 0.33 | 0.38 | 0.32 | SNP |
| chr6_30879636_C_T | 30879636 | 1.81 | 1.21 | 2.71 | 0.00412 | 0.11 | 0.13 | 0.11 | SNP |
| chr6_31321776_T_C | 31321776 | 1.91 | 1.22 | 2.95 | 0.00412 | 0.03 | 0.04 | 0.02 | SNP |
| chr6_31243660_G_T | 31243660 | 2.14 | 1.26 | 3.58 | 0.00417 | 0.02 | 0.03 | 0.02 | SNP |
| chr6_32362447_C_T | 32362447 | 1.81 | 1.20 | 2.70 | 0.00421 | 0.03 | 0.05 | 0.03 | SNP |
| chr6_32373796_A_G | 32373796 | 1.81 | 1.20 | 2.70 | 0.00421 | 0.03 | 0.05 | 0.03 | SNP |
| chr6_32005649_A_G | 32005649 | 0.66 | 0.50 | 0.87 | 0.00428 | 0.11 | 0.08 | 0.12 | SNP |
| chr6_31320569_G_T | 31320569 | 0.45 | 0.25 | 0.76 | 0.00432 | 0.04 | 0.02 | 0.04 | SNP |
| chr6_31129310_C_T | 31129310 | 1.91 | 1.22 | 2.97 | 0.00434 | 0.12 | 0.13 | 0.12 | SNP |
| chr6_31778272_A_G | 31778272 | 1.49 | 1.13 | 1.96 | 0.00438 | 0.22 | 0.23 | 0.21 | SNP |
| chr6_31708463_C_A | 31708463 | 2.10 | 1.26 | 3.49 | 0.00438 | 0.12 | 0.13 | 0.12 | SNP |
| chr6_32612410_T_C | 32612410 | 0.71 | 0.56 | 0.90 | 0.00439 | 0.44 | 0.37 | 0.46 | SNP |
| chr6_32013500_T_C | 32013500 | 0.62 | 0.45 | 0.86 | 0.00442 | 0.09 | 0.06 | 0.10 | SNP |
| chr6_29944670_G_C | 29944670 | 1.33 | 1.09 | 1.63 | 0.00443 | 0.47 | 0.49 | 0.46 | SNP |
| chr6_29893293_A_G | 29893293 | 1.28 | 1.08 | 1.52 | 0.00445 | 0.48 | 0.50 | 0.47 | SNP |
| chr6_29910189_T_A | 29910189 | 1.31 | 1.09 | 1.57 | 0.00447 | 0.37 | 0.39 | 0.36 | SNP |
| chr6_32817774_T_G | 32817774 | 0.71 | 0.56 | 0.90 | 0.00450 | 0.17 | 0.14 | 0.18 | SNP |
| chr6_31324064_T_A | 31324064 | 0.60 | 0.41 | 0.84 | 0.00450 | 0.09 | 0.07 | 0.09 | SNP |
| chr6_32490732_G_A | 32490732 | 2.06 | 1.24 | 3.38 | 0.00450 | 0.02 | 0.04 | 0.02 | SNP |
| chr6_29892815_A_G | 29892815 | 1.46 | 1.12 | 1.90 | 0.00451 | 0.15 | 0.17 | 0.14 | SNP |
| chr6_31629995_G_A | 31629995 | 0.57 | 0.38 | 0.83 | 0.00451 | 0.06 | 0.04 | 0.07 | SNP |
| chr6_32782149_G_A | 32782149 | 0.61 | 0.42 | 0.85 | 0.00451 | 0.07 | 0.05 | 0.08 | SNP |
| chr6_31322690_A_G | 31322690 | 1.90 | 1.21 | 2.93 | 0.00456 | 0.03 | 0.04 | 0.02 | SNP |
| chr6_32608844_A_G | 32608844 | 1.38 | 1.10 | 1.72 | 0.00458 | 0.28 | 0.35 | 0.26 | SNP |
| chr6_32976185_G_A | 32976185 | 0.41 | 0.21 | 0.73 | 0.00458 | 0.03 | 0.01 | 0.03 | SNP |
| chr6_32498208_G_T | 32498208 | 1.38 | 1.10 | 1.71 | 0.00459 | 0.30 | 0.38 | 0.28 | SNP |
| chr6_32441008_C_T | 32441008 | 1.37 | 1.10 | 1.71 | 0.00465 | 0.31 | 0.38 | 0.29 | SNP |
| chr6_30857894_G_A | 30857894 | 1.76 | 1.19 | 2.59 | 0.00467 | 0.12 | 0.13 | 0.11 | SNP |
| chr6_30867527_G_A | 30867527 | 1.76 | 1.19 | 2.59 | 0.00467 | 0.12 | 0.13 | 0.11 | SNP |

| | | | | | | | | | |
|---|---|---|---|---|---|---|---|---|---|
| chr6_31473957_C_G | 31473957 | 1.96 | 1.23 | 3.13 | 0.00467 | 0.12 | 0.13 | 0.12 | SNP |
| chr6_31860337_C_T | 31860337 | 1.42 | 1.11 | 1.81 | 0.00469 | 0.37 | 0.39 | 0.37 | SNP |
| chr6_31322790_T_A | 31322790 | 1.90 | 1.22 | 2.97 | 0.00469 | 0.12 | 0.13 | 0.12 | SNP |
| chr6_32491581_A_C | 32491581 | 1.41 | 1.11 | 1.80 | 0.00472 | 0.26 | 0.33 | 0.25 | SNP |
| chr6_32609358_C_T | 32609358 | 1.38 | 1.10 | 1.73 | 0.00491 | 0.29 | 0.35 | 0.27 | SNP |
| chr6_31244230_C_A | 31244230 | 1.62 | 1.16 | 2.27 | 0.00492 | 0.18 | 0.20 | 0.17 | SNP |
| chr6_33052981_C_A | 33052981 | 1.42 | 1.11 | 1.81 | 0.00492 | 0.11 | 0.13 | 0.11 | SNP |
| chr6_33590009_A_T | 33590009 | 0.56 | 0.37 | 0.83 | 0.00494 | 0.06 | 0.04 | 0.06 | SNP |
| chr6_31507160_A_G | 31507160 | 0.42 | 0.22 | 0.74 | 0.00494 | 0.03 | 0.02 | 0.04 | SNP |
| chr6_31558702_C_T | 31558702 | 2.02 | 1.23 | 3.29 | 0.00496 | 0.12 | 0.13 | 0.12 | SNP |
| chr6_32812528_C_T | 32812528 | 1.33 | 1.09 | 1.63 | 0.00504 | 0.16 | 0.20 | 0.16 | SNP |
| chr6_32489960_A_G | 32489960 | 1.58 | 1.14 | 2.16 | 0.00505 | 0.06 | 0.09 | 0.06 | SNP |
| chr6_30879987_C_T | 30879987 | 1.76 | 1.18 | 2.60 | 0.00508 | 0.12 | 0.13 | 0.11 | SNP |
| chr6_31324531_T_C | 31324531 | 1.43 | 1.11 | 1.83 | 0.00517 | 0.31 | 0.33 | 0.31 | SNP |
| chr6_32151420_G_C | 32151420 | 0.60 | 0.41 | 0.85 | 0.00523 | 0.06 | 0.04 | 0.07 | SNP |
| chr6_32412580_C_T | 32412580 | 1.40 | 1.10 | 1.78 | 0.00524 | 0.23 | 0.25 | 0.22 | SNP |
| chr6_32163274_G_A | 32163274 | 1.75 | 1.17 | 2.57 | 0.00528 | 0.03 | 0.05 | 0.03 | SNP |
| chr6_32166380_C_G | 32166380 | 1.75 | 1.17 | 2.57 | 0.00528 | 0.03 | 0.05 | 0.03 | SNP |
| chr6_31837491_G_A | 31837491 | 1.40 | 1.11 | 1.77 | 0.00529 | 0.49 | 0.50 | 0.49 | SNP |
| chr6_31430060_G_T | 31430060 | 1.89 | 1.20 | 2.94 | 0.00529 | 0.03 | 0.04 | 0.02 | SNP |
| chr6_32340593_G_C | 32340593 | 0.51 | 0.31 | 0.80 | 0.00529 | 0.04 | 0.02 | 0.05 | SNP |
| chr6_33625696_T_C | 33625696 | 1.35 | 1.09 | 1.67 | 0.00531 | 0.26 | 0.31 | 0.25 | SNP |
| chr6_32500925_A_C | 32500925 | 0.74 | 0.60 | 0.91 | 0.00531 | 0.19 | 0.15 | 0.20 | SNP |
| chr6_31248256_C_T | 31248256 | 0.51 | 0.31 | 0.80 | 0.00534 | 0.04 | 0.03 | 0.05 | SNP |
| chr6_32785142_G_A | 32785142 | 1.58 | 1.14 | 2.19 | 0.00538 | 0.06 | 0.10 | 0.05 | SNP |
| chr6_29892196_T_A | 29892196 | 1.28 | 1.08 | 1.53 | 0.00539 | 0.47 | 0.48 | 0.47 | SNP |
| chr6_32826985_C_T | 32826985 | 0.78 | 0.66 | 0.93 | 0.00545 | 0.43 | 0.39 | 0.44 | SNP |
| chr6_32627480_G_A | 32627480 | 1.38 | 1.10 | 1.72 | 0.00548 | 0.38 | 0.44 | 0.37 | SNP |
| chr6_32490746_C_A | 32490746 | 2.00 | 1.21 | 3.23 | 0.00549 | 0.02 | 0.04 | 0.02 | SNP |
| chr6_33542523_T_C | 33542523 | 1.29 | 1.08 | 1.55 | 0.00550 | 0.22 | 0.25 | 0.21 | SNP |
| chr6_31084288_T_C | 31084288 | 0.76 | 0.62 | 0.92 | 0.00560 | 0.48 | 0.45 | 0.49 | SNP |
| chr6_31243895_T_C | 31243895 | 1.35 | 1.09 | 1.67 | 0.00560 | 0.39 | 0.41 | 0.38 | SNP |
| chr6_32134828_C_T | 32134828 | 0.46 | 0.26 | 0.78 | 0.00564 | 0.04 | 0.02 | 0.05 | SNP |
| chr6_32188713_C_G | 32188713 | 0.48 | 0.28 | 0.79 | 0.00571 | 0.04 | 0.02 | 0.04 | SNP |
| chr6_32080383_C_T | 32080383 | 0.62 | 0.44 | 0.86 | 0.00574 | 0.08 | 0.05 | 0.09 | SNP |
| chr6_32627446_C_T | 32627446 | 1.70 | 1.16 | 2.47 | 0.00576 | 0.04 | 0.06 | 0.04 | SNP |
| chr6_32611467_A_C | 32611467 | 1.44 | 1.11 | 1.86 | 0.00576 | 0.19 | 0.26 | 0.17 | SNP |
| chr6_32306922_T_C | 32306922 | 1.77 | 1.17 | 2.64 | 0.00580 | 0.03 | 0.05 | 0.03 | SNP |
| chr6_32012755_G_A | 32012755 | 0.60 | 0.41 | 0.86 | 0.00587 | 0.08 | 0.05 | 0.09 | SNP |
| chr6_32490747_T_A | 32490747 | 1.98 | 1.21 | 3.20 | 0.00592 | 0.02 | 0.04 | 0.02 | SNP |
| chr6_31243971_G_A | 31243971 | 1.46 | 1.12 | 1.92 | 0.00592 | 0.32 | 0.33 | 0.32 | SNP |
| chr6_30855211_T_G | 30855211 | 1.73 | 1.17 | 2.56 | 0.00601 | 0.12 | 0.13 | 0.11 | SNP |
| chr6_31243830_A_G | 31243830 | 1.35 | 1.09 | 1.67 | 0.00603 | 0.39 | 0.41 | 0.38 | SNP |
| xHLA_2field_DQB1_06_03 | 32627244 | 0.60 | 0.41 | 0.85 | 0.00617 | 0.08 | 0.05 | 0.09 | HLA (xHLA) |
| chr6_32976147_T_A | 32976147 | 0.41 | 0.20 | 0.74 | 0.00620 | 0.03 | 0.01 | 0.03 | SNP |
| chr6_31730568_A_G | 31730568 | 0.61 | 0.42 | 0.86 | 0.00623 | 0.07 | 0.05 | 0.08 | SNP |
| chr6_32975578_T_C | 32975578 | 1.32 | 1.08 | 1.62 | 0.00624 | 0.16 | 0.20 | 0.15 | SNP |
| chr6_31116142_G_A | 31116142 | 1.40 | 1.10 | 1.79 | 0.00625 | 0.26 | 0.27 | 0.26 | SNP |
| chr6_32036357_G_A | 32036357 | 0.65 | 0.47 | 0.88 | 0.00625 | 0.09 | 0.06 | 0.10 | SNP |
| chr6_32409530_A_G | 32409530 | 1.29 | 1.07 | 1.54 | 0.00629 | 0.40 | 0.42 | 0.39 | SNP |
| chr6_33546498_C_T | 33546498 | 1.29 | 1.07 | 1.55 | 0.00636 | 0.22 | 0.25 | 0.21 | SNP |
| chr6_32375347_T_G | 32375347 | 0.51 | 0.30 | 0.81 | 0.00638 | 0.04 | 0.02 | 0.04 | SNP |
| chr6_32186050_G_A | 32186050 | 1.34 | 1.09 | 1.66 | 0.00643 | 0.41 | 0.46 | 0.40 | SNP |
| chr6_31894355_C_T | 31894355 | 0.67 | 0.49 | 0.89 | 0.00659 | 0.10 | 0.08 | 0.10 | SNP |
| chr6_32405671_C_A | 32405671 | 1.31 | 1.08 | 1.59 | 0.00659 | 0.32 | 0.34 | 0.31 | SNP |
| chr6_32825379_A_G | 32825379 | 0.79 | 0.66 | 0.94 | 0.00663 | 0.45 | 0.42 | 0.46 | SNP |
| chr6_32077380_C_T | 32077380 | 0.63 | 0.45 | 0.87 | 0.00666 | 0.08 | 0.05 | 0.09 | SNP |
| chr6_31324051_C_A | 31324051 | 0.61 | 0.43 | 0.87 | 0.00667 | 0.09 | 0.07 | 0.09 | SNP |
| chr6_32785841_C_T | 32785841 | 1.56 | 1.13 | 2.14 | 0.00682 | 0.06 | 0.10 | 0.05 | SNP |
| chr6_32709204_G_C | 32709204 | 0.70 | 0.53 | 0.90 | 0.00683 | 0.21 | 0.17 | 0.22 | SNP |
| chr6_31118942_T_C | 31118942 | 1.39 | 1.09 | 1.77 | 0.00687 | 0.26 | 0.26 | 0.26 | SNP |
| chr6_32369601_T_C | 32369601 | 0.51 | 0.30 | 0.81 | 0.00689 | 0.04 | 0.02 | 0.04 | SNP |
| chr6_32154285_A_G | 32154285 | 1.50 | 1.11 | 2.00 | 0.00694 | 0.19 | 0.20 | 0.18 | SNP |
| chr6_32489964_G_A | 32489964 | 1.54 | 1.12 | 2.10 | 0.00695 | 0.06 | 0.09 | 0.06 | SNP |
| chr6_29892615_C_T | 29892615 | 0.80 | 0.68 | 0.94 | 0.00697 | 0.50 | 0.48 | 0.50 | SNP |
| chr6_31112602_G_C | 31112602 | 1.38 | 1.09 | 1.75 | 0.00699 | 0.26 | 0.27 | 0.26 | SNP |
| chr6_32783405_C_T | 32783405 | 1.55 | 1.13 | 2.14 | 0.00704 | 0.06 | 0.10 | 0.05 | SNP |
| chr6_32304466_T_A | 32304466 | 0.51 | 0.31 | 0.81 | 0.00707 | 0.04 | 0.02 | 0.05 | SNP |
| chr6_29909988_T_C | 29909988 | 1.28 | 1.07 | 1.54 | 0.00712 | 0.36 | 0.37 | 0.36 | SNP |
| chr6_33596664_A_C | 33596664 | 0.57 | 0.38 | 0.85 | 0.00717 | 0.05 | 0.04 | 0.06 | SNP |
| chr6_30878857_G_A | 30878857 | 1.54 | 1.12 | 2.10 | 0.00718 | 0.07 | 0.10 | 0.06 | SNP |
| chr6_32804381_A_T | 32804381 | 0.58 | 0.38 | 0.85 | 0.00719 | 0.06 | 0.04 | 0.06 | SNP |
| chr6_33545340_G_A | 33545340 | 0.75 | 0.60 | 0.92 | 0.00720 | 0.26 | 0.21 | 0.27 | SNP |
| chr6_31324953_T_C | 31324953 | 1.41 | 1.10 | 1.81 | 0.00736 | 0.37 | 0.40 | 0.37 | SNP |
| chr6_31243979_A_T | 31243979 | 1.45 | 1.10 | 1.90 | 0.00739 | 0.32 | 0.33 | 0.32 | SNP |
| chr6_31237918_G_A | 31237918 | 0.61 | 0.42 | 0.87 | 0.00741 | 0.08 | 0.05 | 0.09 | SNP |
| chr6_31930351_A_G | 31930351 | 0.55 | 0.35 | 0.84 | 0.00744 | 0.05 | 0.03 | 0.05 | SNP |
| chr6_32052983_C_T | 32052983 | 0.65 | 0.47 | 0.89 | 0.00745 | 0.09 | 0.06 | 0.10 | SNP |
| chr6_32633811_C_T | 32633811 | 1.63 | 1.13 | 2.32 | 0.00750 | 0.04 | 0.06 | 0.04 | SNP |
| chr6_31110391_G_C | 31110391 | 1.38 | 1.09 | 1.74 | 0.00751 | 0.26 | 0.26 | 0.26 | SNP |
| chr6_29912368_T_C | 29912368 | 1.28 | 1.07 | 1.54 | 0.00758 | 0.37 | 0.39 | 0.37 | SNP |
| chr6_32520337_A_G | 32520337 | 1.38 | 1.09 | 1.75 | 0.00759 | 0.32 | 0.42 | 0.30 | SNP |
| chr6_33136076_G_A | 33136076 | 1.43 | 1.10 | 1.85 | 0.00769 | 0.11 | 0.12 | 0.10 | SNP |
| xHLA_2field_C_03_04 | 31236526 | 0.71 | 0.55 | 0.91 | 0.00769 | 0.15 | 0.14 | 0.15 | HLA (xHLA) |
| chr6_31085356_C_T | 31085356 | 1.79 | 1.16 | 2.74 | 0.00772 | 0.12 | 0.13 | 0.12 | SNP |
| chr6_32611034_T_A | 32611034 | 1.35 | 1.08 | 1.68 | 0.00775 | 0.27 | 0.33 | 0.26 | SNP |
| chr6_32007625_C_T | 32007625 | 0.66 | 0.48 | 0.89 | 0.00791 | 0.10 | 0.07 | 0.10 | SNP |
| chr6_33381459_A_G | 33381459 | 0.50 | 0.28 | 0.81 | 0.00792 | 0.04 | 0.02 | 0.05 | SNP |
| chr6_32323905_C_A | 32323905 | 0.52 | 0.31 | 0.82 | 0.00795 | 0.04 | 0.02 | 0.05 | SNP |
| chr6_32707585_G_A | 32707585 | 0.70 | 0.54 | 0.91 | 0.00805 | 0.21 | 0.17 | 0.22 | SNP |
| chr6_32553662_A_G | 32553662 | 0.76 | 0.62 | 0.93 | 0.00817 | 0.38 | 0.34 | 0.40 | SNP |
| chr6_32558284_T_C | 32558284 | 0.64 | 0.45 | 0.88 | 0.00831 | 0.08 | 0.06 | 0.08 | SNP |
| chr6_31248324_C_T | 31248324 | 0.52 | 0.31 | 0.83 | 0.00833 | 0.04 | 0.03 | 0.05 | SNP |
| chr6_31429927_T_C | 31429927 | 1.51 | 1.11 | 2.05 | 0.00837 | 0.17 | 0.18 | 0.16 | SNP |
| chr6_32604644_G_A | 32604644 | 1.36 | 1.08 | 1.72 | 0.00840 | 0.28 | 0.35 | 0.27 | SNP |
| chr6_31707526_G_A | 31707526 | 1.80 | 1.15 | 2.78 | 0.00850 | 0.03 | 0.04 | 0.02 | SNP |
| chr6_30796545_T_C | 30796545 | 1.62 | 1.13 | 2.32 | 0.00862 | 0.12 | 0.13 | 0.12 | SNP |
| chr6_29913099_G_A | 29913099 | 0.48 | 0.27 | 0.81 | 0.00862 | 0.03 | 0.02 | 0.04 | SNP |
| chr6_31236853_A_G | 31236853 | 1.55 | 1.12 | 2.15 | 0.00878 | 0.18 | 0.19 | 0.17 | SNP |
| chr6_32822975_T_A | 32822975 | 0.80 | 0.67 | 0.94 | 0.00885 | 0.43 | 0.40 | 0.44 | SNP |
| chr6_32974843_C_T | 32974843 | 1.30 | 1.07 | 1.59 | 0.00893 | 0.16 | 0.20 | 0.15 | SNP |
| chr6_32522698_G_T | 32522698 | 0.76 | 0.61 | 0.93 | 0.00901 | 0.41 | 0.37 | 0.42 | SNP |
| chr6_31367882_G_A | 31367882 | 1.67 | 1.14 | 2.46 | 0.00904 | 0.17 | 0.19 | 0.17 | SNP |
| chr6_32973599_C_T | 32973599 | 1.30 | 1.07 | 1.59 | 0.00908 | 0.16 | 0.20 | 0.15 | SNP |
| chr6_32823257_T_C | 32823257 | 0.80 | 0.68 | 0.95 | 0.00908 | 0.45 | 0.42 | 0.46 | SNP |
| chr6_29908838_C_G | 29908838 | 1.27 | 1.06 | 1.52 | 0.00909 | 0.37 | 0.38 | 0.37 | SNP |
| chr6_32085278_C_T | 32085278 | 1.82 | 1.15 | 2.84 | 0.00916 | 0.03 | 0.04 | 0.03 | SNP |
| chr6_32975257_C_T | 32975257 | 1.25 | 1.06 | 1.48 | 0.00917 | 0.48 | 0.53 | 0.46 | SNP |
| chr6_29895973_G_C | 29895973 | 1.35 | 1.07 | 1.68 | 0.00918 | 0.18 | 0.19 | 0.17 | SNP |
| chr6_32826450_T_C | 32826450 | 0.79 | 0.66 | 0.94 | 0.00920 | 0.45 | 0.42 | 0.46 | SNP |
| chr6_29896268_C_T | 29896268 | 1.26 | 1.06 | 1.50 | 0.00921 | 0.44 | 0.46 | 0.43 | SNP |
| xHLA_2field_DRB1_13_01 | 32546552 | 0.64 | 0.45 | 0.89 | 0.00921 | 0.08 | 0.06 | 0.09 | HLA (xHLA) |
| chr6_33290872_G_A | 33290872 | 1.26 | 1.06 | 1.50 | 0.00924 | 0.35 | 0.41 | 0.34 | SNP |
| chr6_31084684_G_A | 31084684 | 0.78 | 0.64 | 0.94 | 0.00928 | 0.49 | 0.46 | 0.50 | SNP |
| chr6_32782387_G_A | 32782387 | 1.32 | 1.07 | 1.62 | 0.00929 | 0.21 | 0.22 | 0.21 | SNP |
| chr6_32076499_G_A | 32076499 | 1.30 | 1.07 | 1.59 | 0.00932 | 0.37 | 0.43 | 0.36 | SNP |
| chr6_31473247_C_T | 31473247 | 0.50 | 0.29 | 0.82 | 0.00935 | 0.03 | 0.02 | 0.04 | SNP |
| chr6_32973794_C_T | 32973794 | 1.30 | 1.07 | 1.58 | 0.00937 | 0.16 | 0.20 | 0.15 | SNP |
| chr6_31085340_G_A | 31085340 | 0.76 | 0.61 | 0.93 | 0.00937 | 0.23 | 0.22 | 0.24 | SNP |
| chr6_31844684_A_G | 31844684 | 0.61 | 0.41 | 0.87 | 0.00939 | 0.07 | 0.04 | 0.07 | SNP |
| chr6_32303474_G_T | 32303474 | 1.75 | 1.14 | 2.65 | 0.00943 | 0.03 | 0.04 | 0.03 | SNP |
| chr6_29908954_A_G | 29908954 | 1.27 | 1.06 | 1.52 | 0.00946 | 0.37 | 0.38 | 0.37 | SNP |
| chr6_31727897_C_T | 31727897 | 1.94 | 1.17 | 3.20 | 0.00957 | 0.12 | 0.12 | 0.12 | SNP |
| chr6_32821447_C_T | 32821447 | 1.42 | 1.09 | 1.84 | 0.00962 | 0.13 | 0.18 | 0.12 | SNP |
| chr6_32077690_G_A | 32077690 | 0.51 | 0.30 | 0.83 | 0.00966 | 0.04 | 0.02 | 0.04 | SNP |
| chr6_31540957_T_C | 31540957 | 0.50 | 0.29 | 0.83 | 0.00980 | 0.03 | 0.02 | 0.04 | SNP |
| chr6_32822907_C_T | 32822907 | 0.80 | 0.68 | 0.95 | 0.00980 | 0.45 | 0.42 | 0.46 | SNP |
| chr6_32822858_G_T | 32822858 | 0.80 | 0.67 | 0.95 | 0.00983 | 0.43 | 0.40 | 0.44 | SNP |
| chr6_29909745_G_A | 29909745 | 1.27 | 1.06 | 1.52 | 0.00985 | 0.37 | 0.38 | 0.37 | SNP |
| chr6_31538277_G_A | 31538277 | 0.50 | 0.29 | 0.83 | 0.00986 | 0.03 | 0.02 | 0.04 | SNP |
| chr6_31239896_T_C | 31239896 | 0.74 | 0.59 | 0.93 | 0.00995 | 0.21 | 0.21 | 0.21 | SNP |
| chr6_29912280_C_T | 29912280 | 1.27 | 1.06 | 1.53 | 0.00997 | 0.37 | 0.39 | 0.37 | SNP |
| chr6_33382288_A_G | 33382288 | 1.41 | 1.08 | 1.82 | 0.00998 | 0.10 | 0.12 | 0.09 | SNP |
| chr6_32975699_G_C | 32975699 | 1.31 | 1.06 | 1.60 | 0.00999 | 0.16 | 0.20 | 0.15 | SNP |
| chr6_32605825_G_A | 32605825 | 1.44 | 1.09 | 1.89 | 0.01005 | 0.08 | 0.10 | 0.07 | SNP |
| chr6_31509980_G_T | 31509980 | 1.82 | 1.14 | 2.84 | 0.01008 | 0.03 | 0.04 | 0.03 | SNP |
| chr6_32604124_A_G | 32604124 | 0.76 | 0.62 | 0.94 | 0.01010 | 0.50 | 0.56 | 0.48 | SNP |
| chr6_31371071_C_A | 31371071 | 1.86 | 1.15 | 2.96 | 0.01011 | 0.03 | 0.04 | 0.02 | SNP |
| chr6_32610934_A_G | 32610934 | 1.35 | 1.07 | 1.70 | 0.01024 | 0.31 | 0.37 | 0.29 | SNP |
| chr6_32017524_A_G | 32017524 | 0.49 | 0.27 | 0.82 | 0.01028 | 0.04 | 0.02 | 0.04 | SNP |
| chr6_32975014_T_C | 32975014 | 1.25 | 1.05 | 1.48 | 0.01030 | 0.48 | 0.54 | 0.47 | SNP |
| chr6_29909033_G_A | 29909033 | 1.27 | 1.06 | 1.52 | 0.01035 | 0.37 | 0.38 | 0.36 | SNP |
| chr6_32818430_C_G | 32818430 | 0.55 | 0.35 | 0.85 | 0.01035 | 0.04 | 0.03 | 0.05 | SNP |
| xHLA_2field_DQB1_03_03 | 32627244 | 1.66 | 1.12 | 2.44 | 0.01039 | 0.04 | 0.06 | 0.04 | HLA (xHLA) |
| chr6_32551700_T_G | 32551700 | 1.42 | 1.08 | 1.85 | 0.01041 | 0.16 | 0.22 | 0.14 | SNP |
| chr6_31244332_C_T | 31244332 | 0.56 | 0.35 | 0.85 | 0.01043 | 0.05 | 0.03 | 0.05 | SNP |
| chr6_32605132_T_C | 32605132 | 1.36 | 1.07 | 1.71 | 0.01044 | 0.27 | 0.34 | 0.25 | SNP |
| chr6_32171279_C_T | 32171279 | 1.66 | 1.12 | 2.44 | 0.01045 | 0.03 | 0.05 | 0.03 | SNP |
| chr6_32171288_C_T | 32171288 | 1.66 | 1.12 | 2.44 | 0.01045 | 0.03 | 0.05 | 0.03 | SNP |
| chr6_31566507_G_A | 31566507 | 0.58 | 0.37 | 0.87 | 0.01047 | 0.05 | 0.03 | 0.05 | SNP |
| chr6_29892709_A_G | 29892709 | 0.80 | 0.68 | 0.95 | 0.01054 | 0.50 | 0.51 | 0.50 | SNP |
| chr6_29909616_C_T | 29909616 | 1.27 | 1.06 | 1.52 | 0.01056 | 0.37 | 0.38 | 0.37 | SNP |
| chr6_31248091_G_A | 31248091 | 0.52 | 0.31 | 0.84 | 0.01062 | 0.04 | 0.03 | 0.05 | SNP |
| chr6_32605067_C_G | 32605067 | 1.43 | 1.08 | 1.88 | 0.01070 | 0.08 | 0.10 | 0.08 | SNP |
| chr6_31245620_T_C | 31245620 | 1.53 | 1.10 | 2.13 | 0.01071 | 0.18 | 0.19 | 0.17 | SNP |
| chr6_32604395_A_G | 32604395 | 1.43 | 1.08 | 1.89 | 0.01073 | 0.08 | 0.10 | 0.07 | SNP |
| chr6_31238029_C_T | 31238029 | 1.38 | 1.08 | 1.77 | 0.01077 | 0.36 | 0.38 | 0.35 | SNP |
| chr6_31244300_A_G | 31244300 | 1.55 | 1.11 | 2.18 | 0.01082 | 0.18 | 0.19 | 0.17 | SNP |
| chr6_31247067_G_T | 31247067 | 1.58 | 1.11 | 2.25 | 0.01084 | 0.17 | 0.18 | 0.17 | SNP |
| chr6_32604431_A_T | 32604431 | 1.43 | 1.08 | 1.89 | 0.01089 | 0.08 | 0.10 | 0.07 | SNP |
| chr6_29902373_G_A | 29902373 | 1.27 | 1.06 | 1.53 | 0.01091 | 0.40 | 0.41 | 0.39 | SNP |
| chr6_31705864_C_T | 31705864 | 1.91 | 1.16 | 3.14 | 0.01093 | 0.12 | 0.12 | 0.12 | SNP |

| | | | | | | | | | |
|---|---|---|---|---|---|---|---|---|---|
| chr6_31712196_G_A | 31712196 | 1.91 | 1.16 | 3.14 | 0.01093 | 0.12 | 0.12 | 0.12 | SNP |
| chr6_31725230_G_A | 31725230 | 1.91 | 1.16 | 3.14 | 0.01093 | 0.12 | 0.12 | 0.12 | SNP |
| chr6_31726794_T_C | 31726794 | 1.91 | 1.16 | 3.14 | 0.01093 | 0.12 | 0.12 | 0.12 | SNP |
| chr6_32017522_G_A | 32017522 | 0.49 | 0.27 | 0.83 | 0.01105 | 0.04 | 0.02 | 0.04 | SNP |
| chr6_32605326_A_G | 32605326 | 1.43 | 1.08 | 1.87 | 0.01114 | 0.08 | 0.10 | 0.08 | SNP |
| chr6_31795345_G_A | 31795345 | 0.52 | 0.31 | 0.84 | 0.01115 | 0.04 | 0.02 | 0.04 | SNP |
| chr6_31546495_G_C | 31546495 | 1.76 | 1.13 | 2.70 | 0.01118 | 0.03 | 0.04 | 0.03 | SNP |
| chr6_31726253_T_C | 31726253 | 1.90 | 1.16 | 3.13 | 0.01119 | 0.12 | 0.12 | 0.12 | SNP |
| chr6_32822296_C_G | 32822296 | 0.80 | 0.67 | 0.95 | 0.01122 | 0.43 | 0.39 | 0.44 | SNP |
| chr6_32609476_T_C | 32609476 | 1.43 | 1.08 | 1.88 | 0.01124 | 0.08 | 0.10 | 0.07 | SNP |
| chr6_33542661_G_T | 33542661 | 0.78 | 0.65 | 0.94 | 0.01126 | 0.26 | 0.22 | 0.27 | SNP |
| chr6_29909472_C_G | 29909472 | 1.26 | 1.05 | 1.51 | 0.01127 | 0.37 | 0.38 | 0.37 | SNP |
| chr6_31113214_C_A | 31113214 | 0.77 | 0.63 | 0.94 | 0.01128 | 0.25 | 0.24 | 0.26 | SNP |
| chr6_31236998_T_C | 31236998 | 1.53 | 1.10 | 2.13 | 0.01129 | 0.18 | 0.19 | 0.17 | SNP |
| chr6_29910358_G_C | 29910358 | 1.28 | 1.06 | 1.55 | 0.01138 | 0.46 | 0.48 | 0.45 | SNP |
| chr6_32604974_C_T | 32604974 | 1.43 | 1.08 | 1.88 | 0.01140 | 0.08 | 0.10 | 0.07 | SNP |
| chr6_31721033_G_A | 31721033 | 1.93 | 1.16 | 3.21 | 0.01156 | 0.12 | 0.12 | 0.12 | SNP |
| chr6_33039535_A_T | 33039535 | 1.37 | 1.07 | 1.74 | 0.01158 | 0.11 | 0.12 | 0.11 | SNP |
| chr6_33046662_T_C | 33046662 | 0.77 | 0.63 | 0.94 | 0.01159 | 0.23 | 0.18 | 0.25 | SNP |
| chr6_31745464_G_A | 31745464 | 1.94 | 1.16 | 3.26 | 0.01168 | 0.12 | 0.12 | 0.12 | SNP |
| chr6_31248114_C_T | 31248114 | 0.54 | 0.33 | 0.86 | 0.01170 | 0.04 | 0.03 | 0.05 | SNP |
| chr6_31725285_A_G | 31725285 | 1.90 | 1.15 | 3.12 | 0.01170 | 0.12 | 0.12 | 0.12 | SNP |
| chr6_31085382_C_A | 31085382 | 0.79 | 0.66 | 0.95 | 0.01175 | 0.48 | 0.46 | 0.49 | SNP |
| chr6_32817008_C_T | 32817008 | 0.53 | 0.31 | 0.85 | 0.01177 | 0.04 | 0.02 | 0.04 | SNP |
| chr6_31593265_G_T | 31593265 | 0.62 | 0.42 | 0.89 | 0.01181 | 0.06 | 0.04 | 0.06 | SNP |
| chr6_31275560_T_C | 31275560 | 0.62 | 0.42 | 0.89 | 0.01193 | 0.07 | 0.05 | 0.07 | SNP |
| chr6_31601735_G_A | 31601735 | 0.60 | 0.40 | 0.88 | 0.01200 | 0.06 | 0.04 | 0.06 | SNP |
| chr6_31275691_A_C | 31275691 | 0.62 | 0.42 | 0.89 | 0.01203 | 0.07 | 0.05 | 0.07 | SNP |
| chr6_32974081_C_T | 32974081 | 1.23 | 1.05 | 1.45 | 0.01211 | 0.48 | 0.54 | 0.47 | SNP |
| chr6_31325056_C_T | 31325056 | 1.52 | 1.09 | 2.10 | 0.01211 | 0.15 | 0.16 | 0.15 | SNP |
| chr6_32606695_T_C | 32606695 | 0.70 | 0.52 | 0.92 | 0.01211 | 0.11 | 0.08 | 0.12 | SNP |
| chr6_33280505_C_A | 33280505 | 1.23 | 1.05 | 1.45 | 0.01217 | 0.36 | 0.40 | 0.34 | SNP |
| chr6_31110671_G_C | 31110671 | 0.77 | 0.63 | 0.94 | 0.01220 | 0.25 | 0.24 | 0.26 | SNP |
| chr6_31560565_C_T | 31560565 | 0.51 | 0.29 | 0.84 | 0.01227 | 0.03 | 0.02 | 0.04 | SNP |
| chr6_32633287_C_T | 32633287 | 1.33 | 1.06 | 1.66 | 0.01232 | 0.31 | 0.38 | 0.30 | SNP |
| chr6_31837277_A_C | 31837277 | 1.30 | 1.06 | 1.60 | 0.01232 | 0.49 | 0.49 | 0.49 | SNP |
| chr6_32707023_T_C | 32707023 | 0.70 | 0.52 | 0.92 | 0.01235 | 0.21 | 0.17 | 0.22 | SNP |
| chr6_29910083_G_A | 29910083 | 1.33 | 1.06 | 1.66 | 0.01238 | 0.18 | 0.19 | 0.17 | SNP |
| chr6_33548493_C_T | 33548493 | 0.78 | 0.64 | 0.95 | 0.01240 | 0.33 | 0.28 | 0.35 | SNP |
| chr6_32605689_C_A | 32605689 | 1.42 | 1.07 | 1.87 | 0.01250 | 0.08 | 0.10 | 0.07 | SNP |
| chr6_32707040_G_A | 32707040 | 0.70 | 0.53 | 0.92 | 0.01256 | 0.21 | 0.17 | 0.22 | SNP |
| chr6_32029183_C_T | 32029183 | 0.67 | 0.49 | 0.91 | 0.01261 | 0.09 | 0.07 | 0.10 | SNP |
| chr6_29913171_A_G | 29913171 | 1.26 | 1.05 | 1.51 | 0.01261 | 0.37 | 0.39 | 0.37 | SNP |
| chr6_32711050_C_T | 32711050 | 0.71 | 0.54 | 0.93 | 0.01267 | 0.21 | 0.17 | 0.21 | SNP |
| chr6_33543409_A_C | 33543409 | 1.24 | 1.05 | 1.47 | 0.01267 | 0.40 | 0.46 | 0.39 | SNP |
| chr6_29897759_A_G | 29897759 | 1.32 | 1.06 | 1.64 | 0.01274 | 0.20 | 0.21 | 0.19 | SNP |
| chr6_29858253_G_A | 29858253 | 1.40 | 1.07 | 1.82 | 0.01275 | 0.15 | 0.17 | 0.14 | SNP |
| chr6_32155581_A_G | 32155581 | 1.35 | 1.07 | 1.71 | 0.01279 | 0.23 | 0.26 | 0.22 | SNP |
| chr6_31236201_G_A | 31236201 | 1.38 | 1.07 | 1.78 | 0.01285 | 0.10 | 0.11 | 0.09 | SNP |
| chr6_32977044_G_A | 32977044 | 1.40 | 1.07 | 1.82 | 0.01287 | 0.09 | 0.10 | 0.09 | SNP |
| chr6_32610105_A_G | 32610105 | 1.43 | 1.07 | 1.89 | 0.01295 | 0.08 | 0.10 | 0.07 | SNP |
| chr6_32712666_T_C | 32712666 | 1.46 | 1.08 | 1.96 | 0.01299 | 0.14 | 0.15 | 0.14 | SNP |
| chr6_30229267_G_C | 30229267 | 1.58 | 1.10 | 2.27 | 0.01300 | 0.10 | 0.12 | 0.10 | SNP |
| chr6_29909153_T_G | 29909153 | 1.26 | 1.05 | 1.51 | 0.01306 | 0.37 | 0.38 | 0.37 | SNP |
| chr6_29909840_A_G | 29909840 | 1.25 | 1.05 | 1.48 | 0.01313 | 0.46 | 0.47 | 0.45 | SNP |
| chr6_29944706_G_A | 29944706 | 1.39 | 1.07 | 1.81 | 0.01329 | 0.14 | 0.16 | 0.14 | SNP |
| chr6_32785714_T_C | 32785714 | 1.30 | 1.06 | 1.61 | 0.01339 | 0.21 | 0.22 | 0.21 | SNP |
| chr6_29893403_C_G | 29893403 | 1.39 | 1.07 | 1.80 | 0.01340 | 0.15 | 0.17 | 0.15 | SNP |
| chr6_29758117_G_A | 29758117 | 1.32 | 1.06 | 1.64 | 0.01345 | 0.19 | 0.20 | 0.19 | SNP |
| chr6_32376882_T_G | 32376882 | 1.31 | 1.06 | 1.61 | 0.01347 | 0.48 | 0.53 | 0.47 | SNP |
| chr6_31751071_C_T | 31751071 | 1.75 | 1.11 | 2.71 | 0.01347 | 0.03 | 0.04 | 0.03 | SNP |
| chr6_31237950_C_T | 31237950 | 1.37 | 1.07 | 1.75 | 0.01354 | 0.36 | 0.38 | 0.36 | SNP |
| chr6_32627456_A_G | 32627456 | 0.75 | 0.60 | 0.94 | 0.01362 | 0.35 | 0.31 | 0.37 | SNP |
| chr6_31765984_G_A | 31765984 | 1.94 | 1.15 | 3.28 | 0.01362 | 0.12 | 0.12 | 0.12 | SNP |
| chr6_29896112_C_T | 29896112 | 1.40 | 1.07 | 1.83 | 0.01383 | 0.16 | 0.18 | 0.15 | SNP |
| chr6_32817130_A_G | 32817130 | 0.76 | 0.61 | 0.94 | 0.01386 | 0.18 | 0.16 | 0.19 | SNP |
| chr6_32819865_T_C | 32819865 | 0.73 | 0.57 | 0.93 | 0.01391 | 0.13 | 0.11 | 0.13 | SNP |
| chr6_33256471_C_T | 33256471 | 1.34 | 1.06 | 1.69 | 0.01392 | 0.13 | 0.15 | 0.12 | SNP |
| chr6_32190484_G_A | 32190484 | 0.67 | 0.48 | 0.91 | 0.01394 | 0.09 | 0.06 | 0.09 | SNP |
| chr6_31611777_C_T | 31611777 | 1.54 | 1.09 | 2.16 | 0.01403 | 0.19 | 0.20 | 0.19 | SNP |
| chr6_32375745_G_A | 32375745 | 1.30 | 1.06 | 1.61 | 0.01404 | 0.49 | 0.53 | 0.47 | SNP |
| chr6_32376618_C_T | 32376618 | 1.30 | 1.06 | 1.61 | 0.01404 | 0.49 | 0.53 | 0.47 | SNP |
| chr6_32627700_A_G | 32627700 | 0.75 | 0.59 | 0.94 | 0.01407 | 0.19 | 0.14 | 0.20 | SNP |
| chr6_33268737_C_A | 33268737 | 1.22 | 1.04 | 1.44 | 0.01419 | 0.36 | 0.41 | 0.34 | SNP |
| chr6_31607050_T_C | 31607050 | 1.52 | 1.09 | 2.13 | 0.01425 | 0.19 | 0.20 | 0.19 | SNP |
| chr6_32610330_A_T | 32610330 | 1.41 | 1.07 | 1.86 | 0.01425 | 0.08 | 0.10 | 0.07 | SNP |
| chr6_29768705_C_A | 29768705 | 1.30 | 1.05 | 1.60 | 0.01429 | 0.25 | 0.26 | 0.25 | SNP |
| chr6_29893040_T_C | 29893040 | 1.31 | 1.05 | 1.62 | 0.01430 | 0.19 | 0.21 | 0.19 | SNP |
| chr6_29852855_T_C | 29852855 | 1.33 | 1.06 | 1.66 | 0.01430 | 0.18 | 0.19 | 0.17 | SNP |
| chr6_32085317_C_G | 32085317 | 0.53 | 0.31 | 0.86 | 0.01438 | 0.04 | 0.02 | 0.04 | SNP |
| chr6_32559674_T_A | 32559674 | 1.43 | 1.07 | 1.91 | 0.01440 | 0.08 | 0.10 | 0.07 | SNP |
| chr6_32376471_A_C | 32376471 | 1.33 | 1.06 | 1.67 | 0.01444 | 0.19 | 0.20 | 0.19 | SNP |
| chr6_31636742_A_G | 31636742 | 1.87 | 1.13 | 3.10 | 0.01444 | 0.12 | 0.12 | 0.12 | SNP |
| chr6_32410987_G_A | 32410987 | 1.49 | 1.08 | 2.05 | 0.01445 | 0.17 | 0.18 | 0.17 | SNP |
| chr6_33365640_C_T | 33365640 | 1.34 | 1.06 | 1.69 | 0.01448 | 0.13 | 0.15 | 0.12 | SNP |
| chr6_32376932_C_T | 32376932 | 1.30 | 1.05 | 1.61 | 0.01469 | 0.49 | 0.53 | 0.47 | SNP |
| chr6_32976654_G_A | 32976654 | 1.39 | 1.06 | 1.81 | 0.01473 | 0.09 | 0.10 | 0.09 | SNP |
| chr6_29798610_T_C | 29798610 | 1.32 | 1.05 | 1.64 | 0.01487 | 0.18 | 0.19 | 0.17 | SNP |
| chr6_32636521_G_A | 32636521 | 1.34 | 1.06 | 1.69 | 0.01489 | 0.17 | 0.22 | 0.16 | SNP |
| chr6_30851909_C_A | 30851909 | 1.72 | 1.11 | 2.65 | 0.01493 | 0.13 | 0.15 | 0.12 | SNP |
| chr6_29853750_G_T | 29853750 | 1.39 | 1.06 | 1.81 | 0.01494 | 0.14 | 0.16 | 0.14 | SNP |
| chr6_31240353_C_A | 31240353 | 1.50 | 1.08 | 2.08 | 0.01494 | 0.18 | 0.19 | 0.17 | SNP |
| chr6_31240663_A_T | 31240663 | 1.50 | 1.08 | 2.08 | 0.01494 | 0.18 | 0.19 | 0.17 | SNP |
| chr6_32362408_G_A | 32362408 | 0.72 | 0.54 | 0.93 | 0.01502 | 0.15 | 0.11 | 0.16 | SNP |
| chr6_29712866_C_T | 29712866 | 1.37 | 1.06 | 1.75 | 0.01526 | 0.19 | 0.20 | 0.18 | SNP |
| chr6_31727474_A_G | 31727474 | 1.86 | 1.12 | 3.08 | 0.01547 | 0.12 | 0.12 | 0.12 | SNP |
| chr6_29909751_C_T | 29909751 | 1.24 | 1.04 | 1.47 | 0.01552 | 0.46 | 0.47 | 0.45 | SNP |
| chr6_32559216_G_A | 32559216 | 1.29 | 1.05 | 1.58 | 0.01555 | 0.38 | 0.38 | 0.38 | SNP |
| chr6_29720604_G_A | 29720604 | 1.36 | 1.06 | 1.74 | 0.01556 | 0.20 | 0.21 | 0.20 | SNP |
| chr6_31702710_C_T | 31702710 | 0.63 | 0.43 | 0.91 | 0.01574 | 0.06 | 0.04 | 0.06 | SNP |
| chr6_31830593_A_C | 31830593 | 0.52 | 0.29 | 0.86 | 0.01576 | 0.04 | 0.03 | 0.05 | SNP |
| chr6_29945949_G_A | 29945949 | 1.47 | 1.07 | 2.00 | 0.01586 | 0.11 | 0.12 | 0.11 | SNP |
| chr6_32823186_T_G | 32823186 | 0.54 | 0.32 | 0.87 | 0.01586 | 0.04 | 0.02 | 0.04 | SNP |
| chr6_33382241_T_C | 33382241 | 1.21 | 1.04 | 1.42 | 0.01590 | 0.41 | 0.46 | 0.40 | SNP |
| chr6_32974401_G_T | 32974401 | 1.22 | 1.04 | 1.44 | 0.01594 | 0.48 | 0.54 | 0.47 | SNP |
| chr6_29972984_G_A | 29972984 | 1.27 | 1.05 | 1.55 | 0.01596 | 0.28 | 0.29 | 0.28 | SNP |
| chr6_32603729_A_G | 32603729 | 1.41 | 1.06 | 1.85 | 0.01602 | 0.08 | 0.10 | 0.08 | SNP |
| chr6_32501211_G_A | 32501211 | 1.41 | 1.07 | 1.87 | 0.01606 | 0.19 | 0.21 | 0.19 | SNP |
| chr6_29896680_G_A | 29896680 | 1.23 | 1.04 | 1.47 | 0.01612 | 0.49 | 0.50 | 0.49 | SNP |
| chr6_31462135_G_A | 31462135 | 1.93 | 1.13 | 3.29 | 0.01616 | 0.13 | 0.14 | 0.12 | SNP |
| chr6_31851354_G_A | 31851354 | 0.54 | 0.32 | 0.87 | 0.01617 | 0.04 | 0.03 | 0.05 | SNP |
| chr6_32521596_T_G | 32521596 | 1.32 | 1.05 | 1.67 | 0.01623 | 0.36 | 0.43 | 0.34 | SNP |
| chr6_31624864_A_G | 31624864 | 1.91 | 1.13 | 3.23 | 0.01626 | 0.12 | 0.12 | 0.12 | SNP |
| chr6_31929808_C_T | 31929808 | 1.72 | 1.09 | 2.66 | 0.01628 | 0.03 | 0.04 | 0.03 | SNP |
| chr6_29895469_G_A | 29895469 | 1.40 | 1.06 | 1.83 | 0.01634 | 0.14 | 0.16 | 0.14 | SNP |
| chr6_32556677_C_A | 32556677 | 1.42 | 1.06 | 1.88 | 0.01639 | 0.11 | 0.13 | 0.10 | SNP |
| chr6_32189192_G_A | 32189192 | 0.55 | 0.33 | 0.88 | 0.01642 | 0.04 | 0.02 | 0.04 | SNP |
| chr6_32825348_T_C | 32825348 | 0.81 | 0.68 | 0.96 | 0.01645 | 0.43 | 0.40 | 0.44 | SNP |
| chr6_31543758_G_A | 31543758 | 1.72 | 1.09 | 2.65 | 0.01646 | 0.03 | 0.04 | 0.03 | SNP |
| chr6_32709965_G_T | 32709965 | 0.72 | 0.55 | 0.94 | 0.01647 | 0.20 | 0.17 | 0.21 | SNP |
| chr6_31925921_G_A | 31925921 | 0.55 | 0.33 | 0.88 | 0.01666 | 0.04 | 0.03 | 0.04 | SNP |
| chr6_31241916_T_C | 31241916 | 0.73 | 0.57 | 0.94 | 0.01667 | 0.17 | 0.16 | 0.17 | SNP |
| chr6_32362416_G_T | 32362416 | 1.36 | 1.06 | 1.75 | 0.01670 | 0.24 | 0.25 | 0.23 | SNP |
| chr6_32713018_C_T | 32713018 | 0.71 | 0.54 | 0.94 | 0.01673 | 0.11 | 0.08 | 0.12 | SNP |
| chr6_32713867_C_T | 32713867 | 0.72 | 0.54 | 0.94 | 0.01674 | 0.20 | 0.16 | 0.20 | SNP |
| chr6_32710307_G_A | 32710307 | 0.72 | 0.54 | 0.94 | 0.01677 | 0.11 | 0.08 | 0.12 | SNP |
| chr6_32803999_A_T | 32803999 | 0.47 | 0.24 | 0.84 | 0.01687 | 0.03 | 0.01 | 0.03 | SNP |
| chr6_32500941_C_A | 32500941 | 1.43 | 1.07 | 1.93 | 0.01705 | 0.18 | 0.20 | 0.18 | SNP |
| chr6_32603321_C_T | 32603321 | 0.76 | 0.60 | 0.95 | 0.01706 | 0.22 | 0.16 | 0.23 | SNP |
| chr6_32187605_G_A | 32187605 | 0.78 | 0.64 | 0.96 | 0.01713 | 0.48 | 0.42 | 0.49 | SNP |
| chr6_33548394_G_T | 33548394 | 1.27 | 1.04 | 1.55 | 0.01715 | 0.22 | 0.25 | 0.21 | SNP |
| chr6_29909807_G_T | 29909807 | 1.31 | 1.05 | 1.63 | 0.01718 | 0.18 | 0.19 | 0.17 | SNP |
| chr6_32557962_A_G | 32557962 | 0.75 | 0.59 | 0.95 | 0.01727 | 0.27 | 0.24 | 0.28 | SNP |
| xHLA_2field_B_07_02 | 31321652 | 1.51 | 1.07 | 2.12 | 0.01732 | 0.17 | 0.19 | 0.17 | HLA (xHLA) |
| chr6_29893461_A_G | 29893461 | 1.30 | 1.04 | 1.60 | 0.01750 | 0.19 | 0.20 | 0.18 | SNP |
| chr6_32334258_G_T | 32334258 | 0.77 | 0.61 | 0.95 | 0.01752 | 0.40 | 0.40 | 0.40 | SNP |
| chr6_29913542_G_T | 29913542 | 1.31 | 1.05 | 1.63 | 0.01757 | 0.18 | 0.19 | 0.18 | SNP |
| chr6_29942983_A_G | 29942983 | 1.24 | 1.04 | 1.48 | 0.01763 | 0.46 | 0.47 | 0.46 | SNP |
| chr6_32183175_G_A | 32183175 | 1.24 | 1.04 | 1.49 | 0.01764 | 0.39 | 0.43 | 0.38 | SNP |
| chr6_29909559_A_G | 29909559 | 1.23 | 1.04 | 1.47 | 0.01766 | 0.46 | 0.47 | 0.45 | SNP |
| chr6_33288075_C_A | 33288075 | 1.21 | 1.03 | 1.43 | 0.01773 | 0.35 | 0.40 | 0.34 | SNP |
| chr6_29758726_G_C | 29758726 | 1.30 | 1.05 | 1.61 | 0.01785 | 0.19 | 0.20 | 0.18 | SNP |
| chr6_29892543_C_A | 29892543 | 1.23 | 1.04 | 1.45 | 0.01787 | 0.46 | 0.47 | 0.46 | SNP |
| chr6_29799103_G_A | 29799103 | 1.31 | 1.05 | 1.63 | 0.01791 | 0.18 | 0.19 | 0.17 | SNP |
| chr6_32628965_A_G | 32628965 | 1.35 | 1.05 | 1.72 | 0.01794 | 0.21 | 0.23 | 0.21 | SNP |
| chr6_31239999_A_T | 31239999 | 0.65 | 0.45 | 0.92 | 0.01799 | 0.09 | 0.06 | 0.09 | SNP |
| chr6_32628189_A_G | 32628189 | 1.32 | 1.05 | 1.67 | 0.01812 | 0.20 | 0.26 | 0.19 | SNP |
| chr6_32731313_T_A | 32731313 | 0.73 | 0.56 | 0.94 | 0.01812 | 0.20 | 0.17 | 0.21 | SNP |
| chr6_29911538_A_G | 29911538 | 1.25 | 1.04 | 1.50 | 0.01818 | 0.36 | 0.37 | 0.36 | SNP |
| chr6_32131667_G_A | 32131667 | 0.55 | 0.32 | 0.88 | 0.01818 | 0.04 | 0.02 | 0.04 | SNP |
| chr6_33288271_A_G | 33288271 | 1.21 | 1.03 | 1.43 | 0.01821 | 0.35 | 0.40 | 0.34 | SNP |
| chr6_29909447_C_G | 29909447 | 1.23 | 1.04 | 1.47 | 0.01844 | 0.46 | 0.47 | 0.45 | SNP |
| chr6_32409058_C_T | 32409058 | 1.46 | 1.07 | 2.01 | 0.01846 | 0.18 | 0.19 | 0.17 | SNP |
| chr6_31321919_G_T | 31321919 | 1.57 | 1.07 | 2.27 | 0.01848 | 0.04 | 0.05 | 0.04 | SNP |
| chr6_32606779_G_A | 32606779 | 1.40 | 1.05 | 1.84 | 0.01858 | 0.08 | 0.10 | 0.08 | SNP |
| chr6_31245534_A_G | 31245534 | 1.36 | 1.05 | 1.75 | 0.01860 | 0.10 | 0.11 | 0.09 | SNP |
| chr6_32190217_T_C | 32190217 | 1.30 | 1.04 | 1.61 | 0.01881 | 0.27 | 0.30 | 0.26 | SNP |
| chr6_32089142_G_T | 32089142 | 0.66 | 0.46 | 0.92 | 0.01889 | 0.09 | 0.06 | 0.09 | SNP |
| chr6_32406579_C_T | 32406579 | 1.46 | 1.06 | 2.01 | 0.01893 | 0.17 | 0.19 | 0.17 | SNP |
| chr6_29912935_T_C | 29912935 | 1.31 | 1.04 | 1.63 | 0.01898 | 0.17 | 0.18 | 0.17 | SNP |
| chr6_31113083_G_A | 31113083 | 1.32 | 1.05 | 1.67 | 0.01916 | 0.26 | 0.26 | 0.26 | SNP |
| chr6_33280309_C_T | 33280309 | 1.21 | 1.03 | 1.42 | 0.01930 | 0.35 | 0.40 | 0.34 | SNP |

| | | | | | | | | | |
|---|---|---|---|---|---|---|---|---|---|
| chr6_32825507_C_T | 32825507 | 0.80 | 0.66 | 0.96 | 0.01931 | 0.36 | 0.32 | 0.37 | SNP |
| chr6_31733650_G_A | 31733650 | 0.63 | 0.42 | 0.92 | 0.01935 | 0.06 | 0.04 | 0.06 | SNP |
| chr6_32606266_T_C | 32606266 | 1.39 | 1.05 | 1.83 | 0.01937 | 0.08 | 0.10 | 0.08 | SNP |
| chr6_32522703_T_G | 32522703 | 0.78 | 0.63 | 0.96 | 0.01965 | 0.40 | 0.37 | 0.41 | SNP |
| chr6_29912830_C_T | 29912830 | 1.29 | 1.04 | 1.60 | 0.01972 | 0.19 | 0.20 | 0.18 | SNP |
| chr6_32973281_C_T | 32973281 | 1.21 | 1.03 | 1.42 | 0.01990 | 0.48 | 0.50 | 0.47 | SNP |
| chr6_31918860_A_G | 31918860 | 1.80 | 1.10 | 2.94 | 0.01992 | 0.12 | 0.12 | 0.12 | SNP |
| chr6_29857730_C_G | 29857730 | 1.29 | 1.04 | 1.59 | 0.01992 | 0.18 | 0.19 | 0.18 | SNP |
| chr6_32052444_C_T | 32052444 | 0.57 | 0.34 | 0.90 | 0.01993 | 0.04 | 0.03 | 0.04 | SNP |
| chr6_33359142_A_G | 33359142 | 1.24 | 1.03 | 1.50 | 0.02005 | 0.40 | 0.45 | 0.39 | SNP |
| chr6_32725832_A_G | 32725832 | 0.73 | 0.56 | 0.95 | 0.02006 | 0.20 | 0.17 | 0.21 | SNP |
| chr6_32710466_G_T | 32710466 | 1.24 | 1.03 | 1.50 | 0.02009 | 0.37 | 0.39 | 0.36 | SNP |
| chr6_31607952_G_A | 31607952 | 0.64 | 0.44 | 0.92 | 0.02017 | 0.06 | 0.04 | 0.06 | SNP |
| chr6_33546825_T_C | 33546825 | 1.22 | 1.03 | 1.45 | 0.02033 | 0.40 | 0.45 | 0.39 | SNP |
| chr6_31248995_C_T | 31248995 | 0.57 | 0.35 | 0.90 | 0.02034 | 0.05 | 0.03 | 0.05 | SNP |
| chr6_32035427_T_C | 32035427 | 0.69 | 0.50 | 0.94 | 0.02042 | 0.09 | 0.07 | 0.10 | SNP |
| chr6_32005048_C_G | 32005048 | 0.57 | 0.34 | 0.90 | 0.02050 | 0.04 | 0.03 | 0.04 | SNP |
| chr6_32633415_C_T | 32633415 | 1.38 | 1.05 | 1.80 | 0.02061 | 0.16 | 0.17 | 0.16 | SNP |
| chr6_32725271_G_A | 32725271 | 0.73 | 0.56 | 0.95 | 0.02062 | 0.21 | 0.18 | 0.22 | SNP |
| chr6_32782605_C_T | 32782605 | 1.25 | 1.04 | 1.52 | 0.02062 | 0.37 | 0.40 | 0.36 | SNP |
| chr6_32032421_C_T | 32032421 | 0.69 | 0.50 | 0.94 | 0.02066 | 0.11 | 0.08 | 0.12 | SNP |
| chr6_31241933_A_C | 31241933 | 1.42 | 1.05 | 1.89 | 0.02068 | 0.07 | 0.10 | 0.07 | SNP |
| chr6_31465917_G_T | 31465917 | 1.68 | 1.07 | 2.58 | 0.02087 | 0.03 | 0.04 | 0.03 | SNP |
| chr6_32362741_C_T | 32362741 | 0.72 | 0.55 | 0.95 | 0.02094 | 0.15 | 0.11 | 0.16 | SNP |
| chr6_32362745_G_A | 32362745 | 0.72 | 0.55 | 0.95 | 0.02094 | 0.15 | 0.11 | 0.16 | SNP |
| chr6_31118511_T_G | 31118511 | 0.79 | 0.64 | 0.96 | 0.02097 | 0.25 | 0.24 | 0.25 | SNP |
| chr6_32803408_T_A | 32803408 | 1.70 | 1.07 | 2.65 | 0.02117 | 0.03 | 0.04 | 0.02 | SNP |
| chr6_31238632_C_T | 31238632 | 0.77 | 0.61 | 0.96 | 0.02119 | 0.21 | 0.21 | 0.21 | SNP |
| chr6_29895626_T_A | 29895626 | 1.36 | 1.04 | 1.77 | 0.02138 | 0.15 | 0.17 | 0.15 | SNP |
| chr6_29943039_G_T | 29943039 | 0.57 | 0.35 | 0.90 | 0.02155 | 0.04 | 0.03 | 0.04 | SNP |
| chr6_33382943_T_G | 33382943 | 1.38 | 1.05 | 1.82 | 0.02155 | 0.10 | 0.11 | 0.09 | SNP |
| chr6_32006791_G_A | 32006791 | 0.57 | 0.34 | 0.90 | 0.02176 | 0.04 | 0.03 | 0.04 | SNP |
| chr6_32806673_T_C | 32806673 | 0.57 | 0.35 | 0.90 | 0.02185 | 0.04 | 0.02 | 0.04 | SNP |
| xHLA_2field_DPB1_04_02 | 33043767 | 1.33 | 1.04 | 1.70 | 0.02199 | 0.11 | 0.12 | 0.11 | HLA (xHLA) |
| chr6_33169839_C_A | 33169839 | 1.35 | 1.04 | 1.73 | 0.02203 | 0.10 | 0.11 | 0.10 | SNP |
| chr6_29909440_A_G | 29909440 | 0.71 | 0.52 | 0.94 | 0.02209 | 0.09 | 0.07 | 0.10 | SNP |
| chr6_32412395_A_C | 32412395 | 1.29 | 1.04 | 1.61 | 0.02218 | 0.25 | 0.26 | 0.24 | SNP |
| chr6_32611860_G_A | 32611860 | 1.43 | 1.05 | 1.94 | 0.02233 | 0.18 | 0.19 | 0.18 | SNP |
| chr6_33260353_A_G | 33260353 | 1.65 | 1.06 | 2.53 | 0.02246 | 0.03 | 0.04 | 0.03 | SNP |
| chr6_29909916_T_A | 29909916 | 1.24 | 1.03 | 1.49 | 0.02256 | 0.39 | 0.40 | 0.39 | SNP |
| chr6_31919578_G_A | 31919578 | 0.77 | 0.62 | 0.96 | 0.02258 | 0.48 | 0.44 | 0.49 | SNP |
| chr6_32797466_G_A | 32797466 | 1.44 | 1.05 | 1.95 | 0.02261 | 0.08 | 0.12 | 0.07 | SNP |
| chr6_29909454_T_C | 29909454 | 0.71 | 0.52 | 0.95 | 0.02261 | 0.09 | 0.07 | 0.10 | SNP |
| chr6_32063896_G_A | 32063896 | 0.52 | 0.28 | 0.89 | 0.02279 | 0.04 | 0.02 | 0.04 | SNP |
| chr6_31559353_G_A | 31559353 | 1.52 | 1.06 | 2.17 | 0.02280 | 0.18 | 0.19 | 0.17 | SNP |
| chr6_31601344_C_T | 31601344 | 0.73 | 0.55 | 0.95 | 0.02281 | 0.29 | 0.25 | 0.30 | SNP |
| chr6_32029849_G_A | 32029849 | 0.69 | 0.49 | 0.94 | 0.02282 | 0.09 | 0.07 | 0.10 | SNP |
| chr6_32029205_G_A | 32029205 | 1.81 | 1.08 | 3.01 | 0.02293 | 0.12 | 0.13 | 0.12 | SNP |
| chr6_32362702_T_C | 32362702 | 0.73 | 0.55 | 0.95 | 0.02314 | 0.15 | 0.11 | 0.16 | SNP |
| chr6_32362703_G_T | 32362703 | 0.73 | 0.55 | 0.95 | 0.02314 | 0.15 | 0.11 | 0.16 | SNP |
| chr6_29909197_C_G | 29909197 | 1.29 | 1.03 | 1.61 | 0.02316 | 0.18 | 0.19 | 0.18 | SNP |
| chr6_32974934_G_A | 32974934 | 1.23 | 1.03 | 1.47 | 0.02317 | 0.28 | 0.32 | 0.27 | SNP |
| chr6_31244068_A_G | 31244068 | 1.47 | 1.05 | 2.05 | 0.02332 | 0.18 | 0.19 | 0.18 | SNP |
| chr6_32608217_T_C | 32608217 | 1.53 | 1.05 | 2.20 | 0.02338 | 0.04 | 0.06 | 0.04 | SNP |
| chr6_32604924_C_T | 32604924 | 1.37 | 1.04 | 1.80 | 0.02349 | 0.08 | 0.10 | 0.07 | SNP |
| chr6_32490708_A_C | 32490708 | 2.01 | 1.08 | 3.63 | 0.02350 | 0.02 | 0.03 | 0.01 | SNP |
| chr6_32603897_A_G | 32603897 | 1.38 | 1.04 | 1.82 | 0.02365 | 0.08 | 0.10 | 0.07 | SNP |
| chr6_29908667_G_A | 29908667 | 1.28 | 1.03 | 1.59 | 0.02370 | 0.19 | 0.20 | 0.18 | SNP |
| chr6_29892104_A_G | 29892104 | 1.21 | 1.03 | 1.44 | 0.02378 | 0.48 | 0.49 | 0.48 | SNP |
| chr6_29913042_T_C | 29913042 | 1.29 | 1.03 | 1.61 | 0.02383 | 0.18 | 0.19 | 0.18 | SNP |
| chr6_31321238_G_C | 31321238 | 1.54 | 1.05 | 2.23 | 0.02396 | 0.04 | 0.05 | 0.04 | SNP |
| chr6_32802807_C_T | 32802807 | 0.74 | 0.56 | 0.96 | 0.02404 | 0.15 | 0.11 | 0.16 | SNP |
| chr6_31935567_G_A | 31935567 | 0.60 | 0.38 | 0.92 | 0.02407 | 0.04 | 0.03 | 0.05 | SNP |
| chr6_29913672_A_G | 29913672 | 1.28 | 1.03 | 1.59 | 0.02407 | 0.19 | 0.20 | 0.19 | SNP |
| xHLA_2field_C_02_02 | 31236526 | 0.62 | 0.40 | 0.92 | 0.02411 | 0.06 | 0.04 | 0.06 | HLA (xHLA) |
| chr6_29856178_T_C | 29856178 | 1.33 | 1.04 | 1.71 | 0.02418 | 0.18 | 0.19 | 0.18 | SNP |
| chr6_31837250_A_G | 31837250 | 1.27 | 1.03 | 1.56 | 0.02426 | 0.49 | 0.49 | 0.49 | SNP |
| chr6_31244181_T_C | 31244181 | 1.47 | 1.05 | 2.07 | 0.02429 | 0.18 | 0.19 | 0.17 | SNP |
| chr6_31326131_C_T | 31326131 | 0.80 | 0.66 | 0.97 | 0.02432 | 0.27 | 0.23 | 0.28 | SNP |
| chr6_31600106_C_A | 31600106 | 0.52 | 0.28 | 0.89 | 0.02434 | 0.03 | 0.02 | 0.03 | SNP |
| chr6_29909181_C_A | 29909181 | 1.28 | 1.03 | 1.59 | 0.02439 | 0.19 | 0.20 | 0.18 | SNP |
| chr6_29897379_C_G | 29897379 | 1.21 | 1.02 | 1.43 | 0.02460 | 0.47 | 0.48 | 0.46 | SNP |
| chr6_32975511_G_A | 32975511 | 0.81 | 0.67 | 0.97 | 0.02464 | 0.32 | 0.29 | 0.33 | SNP |
| chr6_31112108_G_T | 31112108 | 1.31 | 1.03 | 1.67 | 0.02469 | 0.27 | 0.27 | 0.27 | SNP |
| chr6_32609520_C_A | 32609520 | 1.38 | 1.04 | 1.82 | 0.02475 | 0.08 | 0.10 | 0.08 | SNP |
| chr6_32362669_T_G | 32362669 | 0.74 | 0.56 | 0.96 | 0.02479 | 0.15 | 0.11 | 0.16 | SNP |
| chr6_32710695_T_G | 32710695 | 0.72 | 0.54 | 0.95 | 0.02485 | 0.11 | 0.08 | 0.11 | SNP |
| chr6_32944094_G_C | 32944094 | 1.35 | 1.04 | 1.74 | 0.02498 | 0.09 | 0.12 | 0.08 | SNP |
| chr6_29856194_G_C | 29856194 | 1.42 | 1.04 | 1.92 | 0.02500 | 0.15 | 0.19 | 0.14 | SNP |
| chr6_32056580_A_C | 32056580 | 0.70 | 0.50 | 0.95 | 0.02509 | 0.09 | 0.07 | 0.10 | SNP |
| chr6_33630846_G_A | 33630846 | 0.73 | 0.55 | 0.96 | 0.02515 | 0.14 | 0.12 | 0.15 | SNP |
| chr6_32627795_A_G | 32627795 | 1.24 | 1.03 | 1.50 | 0.02528 | 0.47 | 0.52 | 0.46 | SNP |
| chr6_32814244_A_T | 32814244 | 0.74 | 0.56 | 0.96 | 0.02538 | 0.10 | 0.09 | 0.11 | SNP |
| chr6_32707508_T_C | 32707508 | 0.73 | 0.55 | 0.96 | 0.02548 | 0.10 | 0.08 | 0.11 | SNP |
| chr6_32707569_G_C | 32707569 | 0.73 | 0.55 | 0.96 | 0.02548 | 0.10 | 0.08 | 0.11 | SNP |
| chr6_32708890_A_G | 32708890 | 0.73 | 0.55 | 0.96 | 0.02550 | 0.10 | 0.08 | 0.11 | SNP |
| chr6_32708413_G_A | 32708413 | 0.73 | 0.55 | 0.96 | 0.02552 | 0.10 | 0.08 | 0.11 | SNP |
| chr6_31325430_A_G | 31325430 | 1.24 | 1.03 | 1.50 | 0.02559 | 0.48 | 0.51 | 0.47 | SNP |
| chr6_31237917_C_T | 31237917 | 0.77 | 0.61 | 0.97 | 0.02562 | 0.21 | 0.21 | 0.21 | SNP |
| chr6_29910721_G_C | 29910721 | 0.69 | 0.49 | 0.95 | 0.02571 | 0.11 | 0.08 | 0.11 | SNP |
| chr6_31464091_G_A | 31464091 | 1.65 | 1.05 | 2.54 | 0.02572 | 0.03 | 0.04 | 0.03 | SNP |
| chr6_31474820_C_T | 31474820 | 1.65 | 1.05 | 2.54 | 0.02572 | 0.03 | 0.04 | 0.03 | SNP |
| chr6_31244082_C_T | 31244082 | 1.35 | 1.03 | 1.74 | 0.02572 | 0.10 | 0.11 | 0.09 | SNP |
| chr6_32707614_A_G | 32707614 | 0.73 | 0.55 | 0.96 | 0.02575 | 0.10 | 0.08 | 0.11 | SNP |
| chr6_32707638_C_T | 32707638 | 0.73 | 0.55 | 0.96 | 0.02575 | 0.10 | 0.08 | 0.11 | SNP |
| chr6_32707641_C_A | 32707641 | 0.73 | 0.55 | 0.96 | 0.02575 | 0.10 | 0.08 | 0.11 | SNP |
| chr6_32707740_T_C | 32707740 | 0.73 | 0.55 | 0.96 | 0.02575 | 0.10 | 0.08 | 0.11 | SNP |
| chr6_32707783_A_G | 32707783 | 0.73 | 0.55 | 0.96 | 0.02575 | 0.10 | 0.08 | 0.11 | SNP |
| chr6_32708798_T_C | 32708798 | 0.73 | 0.55 | 0.96 | 0.02575 | 0.10 | 0.08 | 0.11 | SNP |
| chr6_32709179_A_G | 32709179 | 0.73 | 0.55 | 0.96 | 0.02575 | 0.10 | 0.08 | 0.11 | SNP |
| chr6_32709586_G_A | 32709586 | 0.73 | 0.55 | 0.96 | 0.02575 | 0.10 | 0.08 | 0.11 | SNP |
| chr6_32709738_A_G | 32709738 | 0.73 | 0.55 | 0.96 | 0.02575 | 0.10 | 0.08 | 0.11 | SNP |
| chr6_29913727_C_T | 29913727 | 1.28 | 1.03 | 1.58 | 0.02575 | 0.19 | 0.20 | 0.19 | SNP |
| chr6_32604544_C_T | 32604544 | 1.29 | 1.03 | 1.60 | 0.02580 | 0.32 | 0.33 | 0.31 | SNP |
| chr6_31239681_T_A | 31239681 | 1.33 | 1.03 | 1.71 | 0.02585 | 0.41 | 0.43 | 0.40 | SNP |
| chr6_32377061_C_T | 32377061 | 1.28 | 1.03 | 1.59 | 0.02593 | 0.48 | 0.52 | 0.47 | SNP |
| chr6_31948400_T_G | 31948400 | 0.58 | 0.35 | 0.92 | 0.02595 | 0.04 | 0.03 | 0.04 | SNP |
| chr6_32811629_G_T | 32811629 | 1.32 | 1.03 | 1.69 | 0.02595 | 0.14 | 0.19 | 0.13 | SNP |
| xHLA_2field_C_07_04 | 31236526 | 0.44 | 0.20 | 0.86 | 0.02595 | 0.02 | 0.01 | 0.02 | HLA (xHLA) |
| chr6_29913948_T_G | 29913948 | 1.23 | 1.03 | 1.48 | 0.02601 | 0.38 | 0.39 | 0.38 | SNP |
| chr6_29913944_C_T | 29913944 | 1.23 | 1.02 | 1.48 | 0.02624 | 0.38 | 0.39 | 0.38 | SNP |
| chr6_29692305_G_A | 29692305 | 1.27 | 1.03 | 1.57 | 0.02628 | 0.20 | 0.21 | 0.20 | SNP |
| chr6_31325030_C_T | 31325030 | 1.45 | 1.04 | 2.02 | 0.02630 | 0.19 | 0.19 | 0.18 | SNP |
| chr6_31236231_G_T | 31236231 | 0.78 | 0.62 | 0.97 | 0.02643 | 0.21 | 0.21 | 0.21 | SNP |
| chr6_32707360_G_A | 32707360 | 0.73 | 0.55 | 0.96 | 0.02650 | 0.10 | 0.08 | 0.11 | SNP |
| chr6_32362389_G_A | 32362389 | 0.74 | 0.56 | 0.96 | 0.02662 | 0.15 | 0.11 | 0.16 | SNP |
| chr6_32191457_G_A | 32191457 | 0.78 | 0.63 | 0.97 | 0.02663 | 0.18 | 0.15 | 0.19 | SNP |
| chr6_32411726_A_G | 32411726 | 1.29 | 1.03 | 1.61 | 0.02681 | 0.25 | 0.26 | 0.24 | SNP |
| chr6_32707551_A_G | 32707551 | 1.22 | 1.02 | 1.46 | 0.02688 | 0.38 | 0.41 | 0.38 | SNP |
| chr6_31927342_G_A | 31927342 | 1.34 | 1.03 | 1.73 | 0.02704 | 0.34 | 0.35 | 0.34 | SNP |
| chr6_32707893_A_C | 32707893 | 0.73 | 0.55 | 0.96 | 0.02708 | 0.10 | 0.08 | 0.11 | SNP |
| chr6_32707558_G_A | 32707558 | 1.22 | 1.02 | 1.46 | 0.02710 | 0.38 | 0.41 | 0.38 | SNP |
| chr6_32707427_G_C | 32707427 | 0.73 | 0.55 | 0.96 | 0.02717 | 0.10 | 0.08 | 0.11 | SNP |
| chr6_30229694_A_C | 30229694 | 0.76 | 0.60 | 0.97 | 0.02721 | 0.19 | 0.15 | 0.20 | SNP |
| chr6_32707071_A_G | 32707071 | 0.72 | 0.54 | 0.96 | 0.02733 | 0.11 | 0.08 | 0.11 | SNP |
| chr6_32603939_G_T | 32603939 | 1.27 | 1.03 | 1.56 | 0.02739 | 0.33 | 0.34 | 0.32 | SNP |
| chr6_32707565_C_T | 32707565 | 1.22 | 1.02 | 1.46 | 0.02751 | 0.38 | 0.41 | 0.38 | SNP |
| chr6_32707566_A_G | 32707566 | 1.22 | 1.02 | 1.46 | 0.02751 | 0.38 | 0.41 | 0.38 | SNP |
| chr6_29692562_G_A | 29692562 | 1.27 | 1.02 | 1.56 | 0.02781 | 0.20 | 0.21 | 0.20 | SNP |
| chr6_29856774_A_G | 29856774 | 1.34 | 1.03 | 1.74 | 0.02786 | 0.15 | 0.16 | 0.14 | SNP |
| chr6_31248693_C_G | 31248693 | 0.56 | 0.32 | 0.92 | 0.02794 | 0.04 | 0.03 | 0.04 | SNP |
| chr6_31239306_G_T | 31239306 | 1.48 | 1.04 | 2.09 | 0.02796 | 0.19 | 0.20 | 0.19 | SNP |
| chr6_31922254_C_A | 31922254 | 1.34 | 1.03 | 1.74 | 0.02814 | 0.34 | 0.35 | 0.34 | SNP |
| chr6_29855120_T_G | 29855120 | 1.23 | 1.02 | 1.48 | 0.02819 | 0.31 | 0.32 | 0.31 | SNP |
| chr6_33272626_C_T | 33272626 | 0.79 | 0.63 | 0.97 | 0.02822 | 0.18 | 0.15 | 0.18 | SNP |
| chr6_29909021_C_G | 29909021 | 1.38 | 1.03 | 1.84 | 0.02843 | 0.14 | 0.15 | 0.13 | SNP |
| xHLA_2field_B_57_01 | 31321652 | 1.68 | 1.04 | 2.66 | 0.02852 | 0.03 | 0.04 | 0.02 | HLA (xHLA) |
| chr6_32813279_C_A | 32813279 | 0.74 | 0.56 | 0.96 | 0.02871 | 0.10 | 0.09 | 0.11 | SNP |
| chr6_32362832_G_A | 32362832 | 0.74 | 0.56 | 0.97 | 0.02890 | 0.15 | 0.11 | 0.16 | SNP |
| chr6_32150296_C_A | 32150296 | 0.65 | 0.44 | 0.95 | 0.02894 | 0.06 | 0.04 | 0.07 | SNP |
| chr6_31723389_T_C | 31723389 | 1.50 | 1.04 | 2.17 | 0.02896 | 0.18 | 0.19 | 0.18 | SNP |
| chr6_32707124_T_A | 32707124 | 0.73 | 0.55 | 0.96 | 0.02909 | 0.10 | 0.08 | 0.11 | SNP |
| chr6_31084792_C_T | 31084792 | 0.81 | 0.67 | 0.98 | 0.02942 | 0.48 | 0.46 | 0.48 | SNP |
| chr6_31275485_C_T | 31275485 | 0.65 | 0.43 | 0.95 | 0.02954 | 0.06 | 0.05 | 0.07 | SNP |
| chr6_29908789_T_C | 29908789 | 0.62 | 0.39 | 0.94 | 0.02966 | 0.05 | 0.03 | 0.05 | SNP |
| chr6_32710692_G_T | 32710692 | 1.22 | 1.02 | 1.46 | 0.02976 | 0.38 | 0.40 | 0.37 | SNP |
| chr6_29910176_T_C | 29910176 | 1.23 | 1.02 | 1.48 | 0.02980 | 0.39 | 0.40 | 0.38 | SNP |
| chr6_29913638_C_G | 29913638 | 1.27 | 1.02 | 1.58 | 0.02985 | 0.19 | 0.20 | 0.18 | SNP |
| chr6_29909970_T_A | 29909970 | 0.62 | 0.39 | 0.94 | 0.02998 | 0.05 | 0.03 | 0.05 | SNP |
| chr6_29896108_A_T | 29896108 | 1.34 | 1.03 | 1.74 | 0.03024 | 0.16 | 0.17 | 0.15 | SNP |
| chr6_32172993_C_T | 32172993 | 1.56 | 1.04 | 2.33 | 0.03033 | 0.13 | 0.14 | 0.13 | SNP |
| chr6_32335657_A_G | 32335657 | 1.50 | 1.03 | 2.15 | 0.03050 | 0.04 | 0.05 | 0.03 | SNP |
| chr6_29913137_A_G | 29913137 | 1.37 | 1.03 | 1.83 | 0.03062 | 0.14 | 0.15 | 0.13 | SNP |
| chr6_31237563_T_C | 31237563 | 1.31 | 1.03 | 1.68 | 0.03067 | 0.34 | 0.35 | 0.34 | SNP |
| chr6_31127037_C_T | 31127037 | 0.64 | 0.41 | 0.95 | 0.03082 | 0.05 | 0.03 | 0.06 | SNP |
| chr6_33627367_A_G | 33627367 | 0.61 | 0.38 | 0.94 | 0.03082 | 0.05 | 0.03 | 0.05 | SNP |
| chr6_29913374_C_G | 29913374 | 1.37 | 1.03 | 1.83 | 0.03125 | 0.14 | 0.15 | 0.13 | SNP |
| chr6_32134510_G_A | 32134510 | 1.43 | 1.03 | 1.99 | 0.03126 | 0.19 | 0.21 | 0.18 | SNP |
| chr6_32707884_G_A | 32707884 | 0.73 | 0.55 | 0.97 | 0.03127 | 0.10 | 0.08 | 0.11 | SNP |
| chr6_31248819_C_T | 31248819 | 1.36 | 1.02 | 1.78 | 0.03131 | 0.10 | 0.11 | 0.10 | SNP |
| chr6_30226123_C_T | 30226123 | 1.38 | 1.03 | 1.85 | 0.03140 | 0.13 | 0.14 | 0.12 | SNP |
| chr6_31238377_C_G | 31238377 | 1.47 | 1.03 | 2.08 | 0.03150 | 0.17 | 0.18 | 0.16 | SNP |

| | | | | | | | | | |
|---|---|---|---|---|---|---|---|---|---|
| chr6_32408044_C_A | 32408044 | 0.79 | 0.64 | 0.98 | 0.03163 | 0.50 | 0.44 | 0.51 | SNP |
| chr6_31675501_G_A | 31675501 | 0.69 | 0.48 | 0.96 | 0.03182 | 0.07 | 0.05 | 0.07 | SNP |
| chr6_32707173_T_G | 32707173 | 0.74 | 0.55 | 0.97 | 0.03221 | 0.10 | 0.08 | 0.11 | SNP |
| chr6_31906828_G_A | 31906828 | 0.60 | 0.37 | 0.94 | 0.03223 | 0.04 | 0.03 | 0.04 | SNP |
| chr6_32707806_T_C | 32707806 | 1.21 | 1.02 | 1.45 | 0.03238 | 0.38 | 0.41 | 0.38 | SNP |
| chr6_32501245_A_G | 32501245 | 0.80 | 0.65 | 0.98 | 0.03243 | 0.19 | 0.16 | 0.20 | SNP |
| chr6_29855062_A_G | 29855062 | 1.36 | 1.02 | 1.81 | 0.03261 | 0.15 | 0.16 | 0.14 | SNP |
| chr6_29924889_C_A | 29924889 | 0.81 | 0.67 | 0.98 | 0.03265 | 0.40 | 0.40 | 0.40 | SNP |
| chr6_32945843_T_A | 32945843 | 1.85 | 1.03 | 3.22 | 0.03268 | 0.02 | 0.02 | 0.01 | SNP |
| chr6_29895913_G_A | 29895913 | 0.83 | 0.70 | 0.98 | 0.03273 | 0.45 | 0.44 | 0.45 | SNP |
| chr6_29910286_T_C | 29910286 | 1.27 | 1.02 | 1.59 | 0.03278 | 0.19 | 0.20 | 0.19 | SNP |
| chr6_32797309_T_G | 32797309 | 1.38 | 1.02 | 1.85 | 0.03280 | 0.08 | 0.12 | 0.07 | SNP |
| chr6_32300470_T_G | 32300470 | 0.79 | 0.63 | 0.98 | 0.03280 | 0.40 | 0.40 | 0.40 | SNP |
| chr6_29857852_G_A | 29857852 | 1.25 | 1.02 | 1.54 | 0.03288 | 0.19 | 0.20 | 0.18 | SNP |
| chr6_33272677_C_T | 33272677 | 1.35 | 1.02 | 1.78 | 0.03299 | 0.08 | 0.09 | 0.07 | SNP |
| chr6_32549259_A_G | 32549259 | 1.39 | 1.03 | 1.89 | 0.03327 | 0.18 | 0.19 | 0.18 | SNP |
| chr6_31613991_G_A | 31613991 | 1.45 | 1.03 | 2.03 | 0.03328 | 0.20 | 0.20 | 0.19 | SNP |
| chr6_32707282_A_G | 32707282 | 1.21 | 1.02 | 1.45 | 0.03342 | 0.38 | 0.41 | 0.38 | SNP |
| chr6_32556030_T_C | 32556030 | 1.41 | 1.03 | 1.94 | 0.03346 | 0.17 | 0.18 | 0.17 | SNP |
| chr6_32707168_A_T | 32707168 | 0.74 | 0.55 | 0.97 | 0.03395 | 0.10 | 0.08 | 0.11 | SNP |
| chr6_31594628_A_T | 31594628 | 1.49 | 1.03 | 2.15 | 0.03404 | 0.18 | 0.19 | 0.18 | SNP |
| chr6_29796768_C_T | 29796768 | 1.25 | 1.02 | 1.52 | 0.03412 | 0.22 | 0.23 | 0.21 | SNP |
| chr6_29944314_C_T | 29944314 | 1.21 | 1.01 | 1.44 | 0.03413 | 0.46 | 0.47 | 0.46 | SNP |
| chr6_31240041_T_C | 31240041 | 1.26 | 1.02 | 1.57 | 0.03415 | 0.41 | 0.41 | 0.41 | SNP |
| chr6_29891825_T_C | 29891825 | 1.20 | 1.01 | 1.42 | 0.03417 | 0.48 | 0.48 | 0.48 | SNP |
| chr6_32611414_G_C | 32611414 | 0.55 | 0.30 | 0.92 | 0.03425 | 0.03 | 0.02 | 0.03 | SNP |
| chr6_32362433_C_A | 32362433 | 0.75 | 0.57 | 0.98 | 0.03435 | 0.15 | 0.11 | 0.16 | SNP |
| chr6_29914034_A_G | 29914034 | 1.23 | 1.02 | 1.49 | 0.03437 | 0.39 | 0.40 | 0.39 | SNP |
| chr6_31709778_A_G | 31709778 | 1.71 | 1.04 | 2.80 | 0.03438 | 0.12 | 0.12 | 0.12 | SNP |
| chr6_31947460_T_G | 31947460 | 1.35 | 1.02 | 1.79 | 0.03456 | 0.34 | 0.34 | 0.34 | SNP |
| chr6_31320562_C_T | 31320562 | 1.36 | 1.02 | 1.82 | 0.03461 | 0.19 | 0.21 | 0.19 | SNP |
| chr6_32362453_A_G | 32362453 | 0.75 | 0.57 | 0.98 | 0.03468 | 0.15 | 0.11 | 0.16 | SNP |
| chr6_32362639_G_A | 32362639 | 0.75 | 0.57 | 0.98 | 0.03471 | 0.15 | 0.11 | 0.16 | SNP |
| chr6_32709346_T_C | 32709346 | 1.21 | 1.01 | 1.44 | 0.03493 | 0.38 | 0.41 | 0.38 | SNP |
| chr6_32559076_A_G | 32559076 | 0.80 | 0.65 | 0.98 | 0.03519 | 0.34 | 0.31 | 0.35 | SNP |
| chr6_31241002_A_G | 31241002 | 1.32 | 1.02 | 1.69 | 0.03535 | 0.10 | 0.11 | 0.10 | SNP |
| chr6_32134656_T_C | 32134656 | 1.43 | 1.02 | 2.00 | 0.03548 | 0.19 | 0.21 | 0.18 | SNP |
| chr6_29908676_T_A | 29908676 | 1.36 | 1.02 | 1.82 | 0.03554 | 0.14 | 0.15 | 0.14 | SNP |
| chr6_31240871_G_C | 31240871 | 0.81 | 0.66 | 0.99 | 0.03555 | 0.35 | 0.36 | 0.35 | SNP |
| chr6_29854703_G_A | 29854703 | 1.25 | 1.01 | 1.53 | 0.03565 | 0.19 | 0.20 | 0.18 | SNP |
| chr6_29689164_T_A | 29689164 | 1.25 | 1.01 | 1.55 | 0.03568 | 0.20 | 0.21 | 0.20 | SNP |
| chr6_31929014_C_A | 31929014 | 1.33 | 1.02 | 1.74 | 0.03579 | 0.34 | 0.35 | 0.34 | SNP |
| chr6_31604894_C_T | 31604894 | 1.70 | 1.02 | 2.76 | 0.03587 | 0.02 | 0.03 | 0.02 | SNP |
| chr6_31803130_C_T | 31803130 | 0.60 | 0.37 | 0.95 | 0.03588 | 0.04 | 0.03 | 0.04 | SNP |
| chr6_32520338_G_A | 32520338 | 1.44 | 1.02 | 2.03 | 0.03602 | 0.17 | 0.18 | 0.16 | SNP |
| chr6_31366472_C_T | 31366472 | 1.47 | 1.02 | 2.10 | 0.03615 | 0.18 | 0.18 | 0.18 | SNP |
| chr6_31237087_C_A | 31237087 | 1.31 | 1.01 | 1.69 | 0.03626 | 0.10 | 0.11 | 0.10 | SNP |
| chr6_31850813_T_C | 31850813 | 0.63 | 0.40 | 0.96 | 0.03674 | 0.05 | 0.03 | 0.05 | SNP |
| chr6_33545125_A_G | 33545125 | 1.23 | 1.01 | 1.50 | 0.03679 | 0.21 | 0.24 | 0.20 | SNP |
| xHLA_2field_C_06_02 | 31236526 | 1.39 | 1.01 | 1.90 | 0.03694 | 0.06 | 0.08 | 0.06 | HLA (xHLA) |
| chr6_30515043_T_C | 30515043 | 1.46 | 1.02 | 2.09 | 0.03697 | 0.11 | 0.12 | 0.11 | SNP |
| chr6_31478960_G_A | 31478960 | 1.61 | 1.02 | 2.51 | 0.03705 | 0.03 | 0.04 | 0.03 | SNP |
| chr6_29692634_G_T | 29692634 | 1.25 | 1.01 | 1.54 | 0.03713 | 0.20 | 0.21 | 0.20 | SNP |
| chr6_32062687_G_A | 32062687 | 1.25 | 1.01 | 1.55 | 0.03738 | 0.34 | 0.39 | 0.32 | SNP |
| chr6_31240771_T_C | 31240771 | 1.31 | 1.01 | 1.69 | 0.03741 | 0.10 | 0.11 | 0.10 | SNP |
| chr6_31240932_C_A | 31240932 | 1.31 | 1.01 | 1.69 | 0.03741 | 0.10 | 0.11 | 0.10 | SNP |
| chr6_32942277_G_A | 32942277 | 1.82 | 1.02 | 3.15 | 0.03752 | 0.02 | 0.02 | 0.01 | SNP |
| chr6_32558183_G_T | 32558183 | 1.23 | 1.01 | 1.49 | 0.03754 | 0.49 | 0.53 | 0.48 | SNP |
| xHLA_2field_A_03_01 | 29910309 | 1.28 | 1.01 | 1.62 | 0.03766 | 0.17 | 0.18 | 0.17 | HLA (xHLA) |
| chr6_32321554_G_T | 32321554 | 1.48 | 1.01 | 2.14 | 0.03772 | 0.04 | 0.05 | 0.03 | SNP |
| chr6_32168771_G_T | 32168771 | 1.54 | 1.02 | 2.31 | 0.03775 | 0.13 | 0.14 | 0.13 | SNP |
| chr6_32080191_G_C | 32080191 | 1.74 | 1.03 | 2.95 | 0.03776 | 0.12 | 0.12 | 0.12 | SNP |
| chr6_31380341_G_A | 31380341 | 1.44 | 1.02 | 2.03 | 0.03794 | 0.18 | 0.19 | 0.18 | SNP |
| chr6_31236679_T_C | 31236679 | 0.79 | 0.63 | 0.99 | 0.03814 | 0.41 | 0.39 | 0.41 | SNP |
| chr6_33633527_A_G | 33633527 | 1.21 | 1.01 | 1.44 | 0.03821 | 0.30 | 0.34 | 0.29 | SNP |
| chr6_32020238_A_G | 32020238 | 1.44 | 1.01 | 2.03 | 0.03829 | 0.17 | 0.20 | 0.16 | SNP |
| chr6_29977145_G_C | 29977145 | 1.24 | 1.01 | 1.52 | 0.03837 | 0.25 | 0.26 | 0.25 | SNP |
| chr6_30029651_C_G | 30029651 | 1.24 | 1.01 | 1.53 | 0.03862 | 0.25 | 0.26 | 0.25 | SNP |
| chr6_31237605_G_A | 31237605 | 1.23 | 1.01 | 1.49 | 0.03871 | 0.21 | 0.24 | 0.20 | SNP |
| chr6_29635928_C_T | 29635928 | 1.25 | 1.01 | 1.54 | 0.03927 | 0.22 | 0.22 | 0.21 | SNP |
| chr6_31323020_T_C | 31323020 | 0.75 | 0.56 | 0.98 | 0.03951 | 0.14 | 0.10 | 0.14 | SNP |
| chr6_32377116_C_G | 32377116 | 1.27 | 1.01 | 1.59 | 0.03955 | 0.48 | 0.52 | 0.47 | SNP |
| chr6_31237592_C_A | 31237592 | 1.30 | 1.01 | 1.67 | 0.03976 | 0.34 | 0.35 | 0.34 | SNP |
| chr6_32605578_G_A | 32605578 | 1.25 | 1.01 | 1.53 | 0.03979 | 0.33 | 0.35 | 0.33 | SNP |
| chr6_33641194_C_T | 33641194 | 1.22 | 1.01 | 1.48 | 0.03984 | 0.29 | 0.32 | 0.28 | SNP |
| chr6_30025503_G_A | 30025503 | 1.22 | 1.01 | 1.47 | 0.04002 | 0.30 | 0.31 | 0.29 | SNP |
| xHLA_2field_DQB1_06_02 | 32627244 | 1.43 | 1.01 | 2.02 | 0.04035 | 0.17 | 0.17 | 0.17 | HLA (xHLA) |
| chr6_29942324_T_C | 29942324 | 0.83 | 0.69 | 0.99 | 0.04037 | 0.50 | 0.49 | 0.50 | SNP |
| chr6_32559516_T_C | 32559516 | 0.81 | 0.66 | 0.99 | 0.04049 | 0.44 | 0.40 | 0.44 | SNP |
| chr6_32555965_G_T | 32555965 | 1.39 | 1.01 | 1.91 | 0.04051 | 0.17 | 0.18 | 0.17 | SNP |
| xHLA_2field_DRB1_11_01 | 32546552 | 0.60 | 0.36 | 0.96 | 0.04052 | 0.04 | 0.02 | 0.04 | HLA (xHLA) |
| chr6_29913074_G_A | 29913074 | 1.25 | 1.01 | 1.56 | 0.04075 | 0.19 | 0.20 | 0.19 | SNP |
| chr6_31325270_C_G | 31325270 | 1.22 | 1.01 | 1.48 | 0.04087 | 0.48 | 0.51 | 0.48 | SNP |
| chr6_32193945_G_T | 32193945 | 0.80 | 0.64 | 0.99 | 0.04116 | 0.16 | 0.14 | 0.17 | SNP |
| chr6_29690026_T_C | 29690026 | 0.74 | 0.55 | 0.98 | 0.04118 | 0.10 | 0.08 | 0.10 | SNP |
| chr6_32629403_C_T | 32629403 | 1.30 | 1.01 | 1.67 | 0.04194 | 0.15 | 0.20 | 0.14 | SNP |
| chr6_32184024_C_T | 32184024 | 0.78 | 0.61 | 0.99 | 0.04205 | 0.14 | 0.13 | 0.14 | SNP |
| chr6_32605078_A_G | 32605078 | 1.30 | 1.01 | 1.66 | 0.04236 | 0.21 | 0.27 | 0.19 | SNP |
| chr6_29858840_A_C | 29858840 | 1.32 | 1.01 | 1.71 | 0.04243 | 0.15 | 0.16 | 0.14 | SNP |
| chr6_31383848_G_A | 31383848 | 0.73 | 0.53 | 0.98 | 0.04245 | 0.09 | 0.07 | 0.09 | SNP |
| chr6_31237048_G_A | 31237048 | 1.29 | 1.01 | 1.65 | 0.04261 | 0.34 | 0.35 | 0.34 | SNP |
| chr6_31239937_C_T | 31239937 | 0.79 | 0.63 | 0.99 | 0.04262 | 0.21 | 0.21 | 0.21 | SNP |
| chr6_31237463_G_C | 31237463 | 1.29 | 1.01 | 1.65 | 0.04268 | 0.34 | 0.36 | 0.34 | SNP |
| chr6_32796793_G_A | 32796793 | 0.73 | 0.53 | 0.98 | 0.04278 | 0.08 | 0.07 | 0.09 | SNP |
| chr6_29913078_G_A | 29913078 | 1.25 | 1.01 | 1.55 | 0.04281 | 0.19 | 0.20 | 0.19 | SNP |
| chr6_32014828_G_A | 32014828 | 1.72 | 1.02 | 2.92 | 0.04302 | 0.12 | 0.13 | 0.12 | SNP |
| chr6_33232055_G_A | 33232055 | 0.83 | 0.69 | 0.99 | 0.04306 | 0.33 | 0.29 | 0.34 | SNP |
| chr6_29691097_A_T | 29691097 | 0.74 | 0.55 | 0.98 | 0.04308 | 0.10 | 0.08 | 0.10 | SNP |
| chr6_31240614_C_T | 31240614 | 0.53 | 0.27 | 0.94 | 0.04309 | 0.02 | 0.01 | 0.02 | SNP |
| chr6_29973910_G_A | 29973910 | 1.24 | 1.01 | 1.51 | 0.04313 | 0.25 | 0.26 | 0.25 | SNP |
| chr6_29855063_C_G | 29855063 | 1.22 | 1.01 | 1.47 | 0.04317 | 0.31 | 0.32 | 0.31 | SNP |
| chr6_31236341_C_T | 31236341 | 0.80 | 0.64 | 0.99 | 0.04325 | 0.21 | 0.21 | 0.21 | SNP |
| chr6_32797876_A_G | 32797876 | 0.83 | 0.68 | 0.99 | 0.04339 | 0.41 | 0.35 | 0.42 | SNP |
| chr6_31237353_C_T | 31237353 | 1.29 | 1.01 | 1.65 | 0.04342 | 0.34 | 0.36 | 0.34 | SNP |
| chr6_32193564_C_T | 32193564 | 0.81 | 0.65 | 0.99 | 0.04370 | 0.21 | 0.18 | 0.22 | SNP |
| chr6_32612301_C_T | 32612301 | 1.38 | 1.01 | 1.90 | 0.04432 | 0.18 | 0.18 | 0.17 | SNP |
| chr6_29857890_C_T | 29857890 | 1.21 | 1.00 | 1.46 | 0.04442 | 0.23 | 0.24 | 0.23 | SNP |
| chr6_32798647_A_T | 32798647 | 0.62 | 0.38 | 0.97 | 0.04458 | 0.04 | 0.03 | 0.04 | SNP |
| chr6_29897018_G_A | 29897018 | 1.26 | 1.00 | 1.58 | 0.04480 | 0.18 | 0.19 | 0.18 | SNP |
| chr6_32021355_G_A | 32021355 | 1.89 | 0.99 | 3.47 | 0.04491 | 0.01 | 0.02 | 0.01 | SNP |
| chr6_31528130_C_T | 31528130 | 1.37 | 1.00 | 1.85 | 0.04512 | 0.08 | 0.09 | 0.08 | SNP |
| chr6_31244262_A_G | 31244262 | 0.53 | 0.27 | 0.95 | 0.04545 | 0.02 | 0.01 | 0.03 | SNP |
| chr6_32806576_C_T | 32806576 | 1.22 | 1.00 | 1.49 | 0.04553 | 0.42 | 0.47 | 0.41 | SNP |
| chr6_33547287_C_G | 33547287 | 0.58 | 0.33 | 0.96 | 0.04561 | 0.03 | 0.02 | 0.03 | SNP |
| chr6_29692334_A_G | 29692334 | 0.75 | 0.56 | 0.99 | 0.04563 | 0.10 | 0.08 | 0.11 | SNP |
| chr6_29895944_G_A | 29895944 | 0.84 | 0.71 | 1.00 | 0.04603 | 0.45 | 0.45 | 0.45 | SNP |
| chr6_29911207_G_A | 29911207 | 0.81 | 0.67 | 1.00 | 0.04618 | 0.41 | 0.41 | 0.41 | SNP |
| chr6_29693011_T_C | 29693011 | 1.24 | 1.00 | 1.53 | 0.04628 | 0.20 | 0.21 | 0.20 | SNP |
| chr6_29911154_C_A | 29911154 | 0.82 | 0.67 | 1.00 | 0.04660 | 0.49 | 0.49 | 0.50 | SNP |
| chr6_32193449_T_C | 32193449 | 0.80 | 0.64 | 0.99 | 0.04660 | 0.16 | 0.14 | 0.17 | SNP |
| chr6_31238613_G_A | 31238613 | 1.28 | 1.00 | 1.64 | 0.04681 | 0.35 | 0.35 | 0.34 | SNP |
| chr6_32818678_G_A | 32818678 | 1.21 | 1.00 | 1.46 | 0.04694 | 0.24 | 0.25 | 0.24 | SNP |
| chr6_31544189_A_G | 31544189 | 1.46 | 1.00 | 2.11 | 0.04694 | 0.04 | 0.05 | 0.04 | SNP |
| chr6_31974328_G_A | 31974328 | 1.43 | 1.00 | 2.03 | 0.04701 | 0.05 | 0.07 | 0.05 | SNP |
| chr6_31543101_G_A | 31543101 | 1.51 | 1.00 | 2.25 | 0.04713 | 0.04 | 0.04 | 0.03 | SNP |
| chr6_32363774_G_A | 32363774 | 0.76 | 0.58 | 0.99 | 0.04786 | 0.15 | 0.11 | 0.16 | SNP |
| chr6_29854539_G_A | 29854539 | 1.23 | 1.00 | 1.51 | 0.04805 | 0.19 | 0.20 | 0.19 | SNP |
| chr6_31240776_A_G | 31240776 | 0.50 | 0.24 | 0.95 | 0.04813 | 0.02 | 0.01 | 0.02 | SNP |
| chr6_32635629_G_A | 32635629 | 1.30 | 1.00 | 1.69 | 0.04824 | 0.23 | 0.26 | 0.22 | SNP |
| chr6_32549236_G_T | 32549236 | 1.36 | 1.00 | 1.85 | 0.04850 | 0.18 | 0.19 | 0.18 | SNP |
| chr6_32324055_C_A | 32324055 | 1.40 | 1.00 | 1.95 | 0.04852 | 0.18 | 0.19 | 0.18 | SNP |
| chr6_32338283_G_T | 32338283 | 1.25 | 1.00 | 1.55 | 0.04853 | 0.26 | 0.28 | 0.26 | SNP |
| chr6_32559673_T_G | 32559673 | 1.24 | 1.00 | 1.53 | 0.04871 | 0.31 | 0.36 | 0.30 | SNP |
| chr6_31931137_T_C | 31931137 | 0.77 | 0.58 | 0.99 | 0.04876 | 0.12 | 0.10 | 0.13 | SNP |
| chr6_32551665_A_G | 32551665 | 1.29 | 1.00 | 1.66 | 0.04888 | 0.20 | 0.25 | 0.18 | SNP |
| chr6_31275511_T_A | 31275511 | 0.52 | 0.25 | 0.96 | 0.04903 | 0.02 | 0.01 | 0.03 | SNP |
| chr6_31237405_T_C | 31237405 | 1.28 | 1.00 | 1.64 | 0.04906 | 0.34 | 0.35 | 0.34 | SNP |
| chr6_31238522_C_T | 31238522 | 1.22 | 1.00 | 1.49 | 0.04927 | 0.21 | 0.23 | 0.20 | SNP |
| chr6_31237162_G_C | 31237162 | 1.28 | 1.00 | 1.64 | 0.04946 | 0.34 | 0.35 | 0.34 | SNP |
| chr6_32411646_G_T | 32411646 | 0.81 | 0.66 | 1.00 | 0.04957 | 0.47 | 0.43 | 0.48 | SNP |
| chr6_32608821_G_A | 32608821 | 1.36 | 1.00 | 1.84 | 0.04967 | 0.19 | 0.20 | 0.18 | SNP |
| chr6_32317215_T_C | 32317215 | 0.79 | 0.63 | 1.00 | 0.04967 | 0.42 | 0.44 | 0.42 | SNP |
| chr6_29893195_G_A | 29893195 | 1.18 | 1.00 | 1.40 | 0.04992 | 0.47 | 0.48 | 0.47 | SNP |
| chr6_32096001_C_A | 32096001 | 1.69 | 1.00 | 2.85 | 0.04997 | 0.12 | 0.13 | 0.12 | SNP |

ANCA = anitneutrophil cytoplasmic antibody; AAV = ANCA-associated vasculitis; GPA = granulomatosis with polyangiitis; SNP = single nucleotide polymorphism; OR = Odds ratio; CI = confidence interval; MAF = minor allele frequency. SNPs and HLA alleles with P < 0.05 included in the table.

**Supplemtary Table 4.** Association analysis of MPO-ANCA+ AAV patients versus controls, adjusting for sex and genetic structure (principal components 1-4).

| Variant | position | OR | CI_lower | CI_upper | p | MAF | MAF_cases | MAF_controls | Type (SNP, HLA (xHLA)) |
|---|---|---|---|---|---|---|---|---|---|
| xHLA_2field_DRB1_04_04 | 32546552 | 4.5 | 3.1 | 6.4 | 4.51E-16 | 0.07 | 0.22 | 0.06 | HLA (xHLA) |
| chr6_32609479_C_A | 32609479 | 3.8 | 2.7 | 5.5 | 4.17E-13 | 0.22 | 0.44 | 0.20 | SNP |
| chr6_32635954_G_A | 32635954 | 3.6 | 2.5 | 5.1 | 1.89E-12 | 0.16 | 0.35 | 0.14 | SNP |
| chr6_32559648_G_T | 32559648 | 3.9 | 2.7 | 5.7 | 2.12E-12 | 0.20 | 0.41 | 0.18 | SNP |
| chr6_32606987_T_G | 32606987 | 3.5 | 2.5 | 5.0 | 2.87E-12 | 0.23 | 0.44 | 0.21 | SNP |
| chr6_32604585_A_T | 32604585 | 3.8 | 2.6 | 5.5 | 4.75E-12 | 0.23 | 0.44 | 0.21 | SNP |
| chr6_32603798_A_T | 32603798 | 3.5 | 2.4 | 5.0 | 6.46E-12 | 0.22 | 0.43 | 0.20 | SNP |
| chr6_32605696_G_T | 32605696 | 3.5 | 2.5 | 5.1 | 6.80E-12 | 0.23 | 0.43 | 0.21 | SNP |
| chr6_32605555_T_A | 32605555 | 3.5 | 2.4 | 5.0 | 7.00E-12 | 0.23 | 0.43 | 0.21 | SNP |
| chr6_32605609_G_A | 32605609 | 3.5 | 2.4 | 4.9 | 8.77E-12 | 0.23 | 0.43 | 0.21 | SNP |
| chr6_32629403_C_T | 32629403 | 3.4 | 2.4 | 4.8 | 1.00E-11 | 0.16 | 0.35 | 0.14 | SNP |
| chr6_32559191_A_G | 32559191 | 3.5 | 2.4 | 5.0 | 1.22E-11 | 0.21 | 0.42 | 0.19 | SNP |
| chr6_32629259_T_C | 32629259 | 3.4 | 2.4 | 4.9 | 1.35E-11 | 0.16 | 0.34 | 0.14 | SNP |
| chr6_32608478_T_C | 32608478 | 3.4 | 2.4 | 4.9 | 1.71E-11 | 0.22 | 0.43 | 0.20 | SNP |
| chr6_32607126_A_G | 32607126 | 3.3 | 2.3 | 4.7 | 3.07E-11 | 0.23 | 0.43 | 0.21 | SNP |
| chr6_32605078_A_G | 32605078 | 3.4 | 2.4 | 4.9 | 3.64E-11 | 0.21 | 0.42 | 0.19 | SNP |
| chr6_32605178_T_C | 32605178 | 3.3 | 2.3 | 4.7 | 4.21E-11 | 0.23 | 0.43 | 0.21 | SNP |
| xHLA_2field_DQB1_03_02 | 32627244 | 3.4 | 2.3 | 4.8 | 4.82E-11 | 0.16 | 0.36 | 0.14 | HLA (xHLA) |
| chr6_32559213_A_T | 32559213 | 3.5 | 2.4 | 5.0 | 4.85E-11 | 0.21 | 0.40 | 0.19 | SNP |
| chr6_32628571_C_T | 32628571 | 2.9 | 2.1 | 4.0 | 5.78E-11 | 0.23 | 0.42 | 0.21 | SNP |
| chr6_32628913_G_T | 32628913 | 3.5 | 2.4 | 5.1 | 5.93E-11 | 0.18 | 0.37 | 0.16 | SNP |
| chr6_32605132_T_C | 32605132 | 3.1 | 2.2 | 4.5 | 6.47E-11 | 0.27 | 0.48 | 0.25 | SNP |
| chr6_32559702_A_G | 32559702 | 3.6 | 2.5 | 5.4 | 8.45E-11 | 0.21 | 0.42 | 0.19 | SNP |
| chr6_32610401_G_A | 32610401 | 3.4 | 2.3 | 4.9 | 8.76E-11 | 0.22 | 0.41 | 0.20 | SNP |
| chr6_32608286_T_C | 32608286 | 3.6 | 2.4 | 5.3 | 1.07E-10 | 0.22 | 0.43 | 0.19 | SNP |
| chr6_32628148_C_T | 32628148 | 3.4 | 2.3 | 5.0 | 1.23E-10 | 0.16 | 0.34 | 0.14 | SNP |
| chr6_32611467_A_C | 32611467 | 3.4 | 2.3 | 4.9 | 1.60E-10 | 0.19 | 0.39 | 0.17 | SNP |
| chr6_32628605_T_C | 32628605 | 2.8 | 2.0 | 3.8 | 2.44E-10 | 0.23 | 0.42 | 0.22 | SNP |
| chr6_32559710_C_T | 32559710 | 3.4 | 2.4 | 5.1 | 2.54E-10 | 0.21 | 0.41 | 0.19 | SNP |
| chr6_32627480_G_A | 32627480 | 2.9 | 2.1 | 4.1 | 5.58E-10 | 0.39 | 0.57 | 0.37 | SNP |
| chr6_32629500_C_A | 32629500 | 2.7 | 2.0 | 3.7 | 6.17E-10 | 0.20 | 0.38 | 0.18 | SNP |
| chr6_32628617_A_C | 32628617 | 2.6 | 1.9 | 3.6 | 6.96E-10 | 0.24 | 0.42 | 0.22 | SNP |
| chr6_32634556_G_A | 32634556 | 2.7 | 2.0 | 3.7 | 7.04E-10 | 0.25 | 0.44 | 0.23 | SNP |
| chr6_32628506_C_A | 32628506 | 2.7 | 2.0 | 3.7 | 7.90E-10 | 0.23 | 0.41 | 0.21 | SNP |
| chr6_32551665_A_G | 32551665 | 3.1 | 2.1 | 4.4 | 9.66E-10 | 0.20 | 0.39 | 0.18 | SNP |
| chr6_32633971_A_G | 32633971 | 2.6 | 1.9 | 3.6 | 1.17E-09 | 0.20 | 0.38 | 0.18 | SNP |
| chr6_32607031_G_T | 32607031 | 2.8 | 2.0 | 3.9 | 1.47E-09 | 0.32 | 0.52 | 0.30 | SNP |
| chr6_32627818_C_A | 32627818 | 2.7 | 1.9 | 3.7 | 1.87E-09 | 0.21 | 0.39 | 0.19 | SNP |
| chr6_32628283_G_A | 32628283 | 2.8 | 2.0 | 3.9 | 1.90E-09 | 0.23 | 0.42 | 0.21 | SNP |
| chr6_32604931_C_A | 32604931 | 2.6 | 1.9 | 3.6 | 2.36E-09 | 0.30 | 0.51 | 0.28 | SNP |
| chr6_32629328_G_A | 32629328 | 2.6 | 1.9 | 3.6 | 2.39E-09 | 0.23 | 0.41 | 0.22 | SNP |
| chr6_32551700_T_G | 32551700 | 3.3 | 2.2 | 4.9 | 2.59E-09 | 0.16 | 0.36 | 0.14 | SNP |
| chr6_32635974_A_G | 32635974 | 2.5 | 1.9 | 3.4 | 2.61E-09 | 0.24 | 0.43 | 0.22 | SNP |
| chr6_32628633_T_C | 32628633 | 2.5 | 1.9 | 3.4 | 2.91E-09 | 0.24 | 0.42 | 0.22 | SNP |
| chr6_32629639_T_C | 32629639 | 2.5 | 1.8 | 3.4 | 2.96E-09 | 0.24 | 0.43 | 0.22 | SNP |
| chr6_32635293_C_G | 32635293 | 2.5 | 1.9 | 3.5 | 3.25E-09 | 0.24 | 0.42 | 0.22 | SNP |
| chr6_32629269_C_T | 32629269 | 2.6 | 1.9 | 3.5 | 3.33E-09 | 0.23 | 0.41 | 0.22 | SNP |
| chr6_32634007_A_G | 32634007 | 2.5 | 1.8 | 3.4 | 3.33E-09 | 0.24 | 0.43 | 0.22 | SNP |
| chr6_32629330_C_T | 32629330 | 2.6 | 1.9 | 3.5 | 3.72E-09 | 0.23 | 0.41 | 0.22 | SNP |
| chr6_32634545_C_A | 32634545 | 2.5 | 1.8 | 3.4 | 3.92E-09 | 0.24 | 0.43 | 0.22 | SNP |
| chr6_32634425_C_T | 32634425 | 2.5 | 1.8 | 3.4 | 3.98E-09 | 0.24 | 0.43 | 0.22 | SNP |
| chr6_32629066_T_C | 32629066 | 2.6 | 1.9 | 3.5 | 4.02E-09 | 0.23 | 0.41 | 0.22 | SNP |
| chr6_32627777_G_C | 32627777 | 2.5 | 1.8 | 3.4 | 4.26E-09 | 0.24 | 0.43 | 0.22 | SNP |
| chr6_32635230_G_A | 32635230 | 2.6 | 1.9 | 3.6 | 4.92E-09 | 0.23 | 0.41 | 0.22 | SNP |
| chr6_32634646_T_C | 32634646 | 2.5 | 1.8 | 3.3 | 5.04E-09 | 0.24 | 0.43 | 0.22 | SNP |
| chr6_32628760_C_A | 32628760 | 2.5 | 1.9 | 3.4 | 5.20E-09 | 0.24 | 0.42 | 0.22 | SNP |
| chr6_32628916_G_A | 32628916 | 2.5 | 1.8 | 3.4 | 5.39E-09 | 0.24 | 0.42 | 0.22 | SNP |
| chr6_32606616_A_T | 32606616 | 2.9 | 2.0 | 4.2 | 5.83E-09 | 0.28 | 0.45 | 0.26 | SNP |
| chr6_32635172_C_T | 32635172 | 2.4 | 1.8 | 3.3 | 5.95E-09 | 0.24 | 0.43 | 0.23 | SNP |
| chr6_32633636_G_A | 32633636 | 2.4 | 1.8 | 3.3 | 6.36E-09 | 0.24 | 0.43 | 0.23 | SNP |
| chr6_32634967_T_A | 32634967 | 2.4 | 1.8 | 3.3 | 7.12E-09 | 0.24 | 0.43 | 0.23 | SNP |
| chr6_32627494_T_C | 32627494 | 2.5 | 1.8 | 3.4 | 7.35E-09 | 0.25 | 0.44 | 0.23 | SNP |
| chr6_32629398_G_C | 32629398 | 2.5 | 1.8 | 3.3 | 7.39E-09 | 0.24 | 0.43 | 0.23 | SNP |
| chr6_32628765_C_T | 32628765 | 2.4 | 1.8 | 3.2 | 7.43E-09 | 0.40 | 0.57 | 0.38 | SNP |
| chr6_32628030_G_A | 32628030 | 2.4 | 1.8 | 3.2 | 7.90E-09 | 0.43 | 0.60 | 0.41 | SNP |
| chr6_32629549_G_A | 32629549 | 2.4 | 1.8 | 3.3 | 8.24E-09 | 0.24 | 0.43 | 0.23 | SNP |
| chr6_32610008_C_T | 32610008 | 2.5 | 1.9 | 3.5 | 8.56E-09 | 0.30 | 0.49 | 0.28 | SNP |
| chr6_32636443_A_C | 32636443 | 2.4 | 1.8 | 3.3 | 8.90E-09 | 0.24 | 0.42 | 0.22 | SNP |
| chr6_32633899_T_C | 32633899 | 2.4 | 1.8 | 3.3 | 9.13E-09 | 0.25 | 0.43 | 0.23 | SNP |
| chr6_32636521_G_A | 32636521 | 2.6 | 1.9 | 3.7 | 9.48E-09 | 0.18 | 0.35 | 0.16 | SNP |
| chr6_32633781_C_T | 32633781 | 2.4 | 1.8 | 3.3 | 9.54E-09 | 0.24 | 0.43 | 0.23 | SNP |
| chr6_32629531_A_G | 32629531 | 2.4 | 1.8 | 3.3 | 9.67E-09 | 0.24 | 0.43 | 0.23 | SNP |
| chr6_32634057_C_T | 32634057 | 2.4 | 1.8 | 3.3 | 9.77E-09 | 0.24 | 0.42 | 0.22 | SNP |
| chr6_32636351_T_C | 32636351 | 2.4 | 1.8 | 3.3 | 1.04E-08 | 0.24 | 0.42 | 0.22 | SNP |
| chr6_32521591_C_T | 32521591 | 2.6 | 1.9 | 3.5 | 1.08E-08 | 0.32 | 0.52 | 0.30 | SNP |
| chr6_32635248_A_T | 32635248 | 2.5 | 1.8 | 3.5 | 1.10E-08 | 0.23 | 0.40 | 0.21 | SNP |
| chr6_32559708_G_A | 32559708 | 2.7 | 1.9 | 3.8 | 1.12E-08 | 0.27 | 0.47 | 0.25 | SNP |
| chr6_32628787_C_T | 32628787 | 2.5 | 1.8 | 3.4 | 1.15E-08 | 0.24 | 0.42 | 0.22 | SNP |
| chr6_32629755_G_A | 32629755 | 2.4 | 1.8 | 3.3 | 1.18E-08 | 0.24 | 0.42 | 0.22 | SNP |
| chr6_32605724_A_G | 32605724 | 2.5 | 1.8 | 3.5 | 1.18E-08 | 0.29 | 0.48 | 0.27 | SNP |
| chr6_32628439_G_A | 32628439 | 2.4 | 1.8 | 3.2 | 1.18E-08 | 0.40 | 0.57 | 0.39 | SNP |
| chr6_32634355_G_A | 32634355 | 2.4 | 1.8 | 3.3 | 1.19E-08 | 0.24 | 0.42 | 0.22 | SNP |
| chr6_32629543_T_C | 32629543 | 2.4 | 1.8 | 3.2 | 1.20E-08 | 0.24 | 0.43 | 0.23 | SNP |
| chr6_32634506_C_T | 32634506 | 2.4 | 1.8 | 3.2 | 1.22E-08 | 0.25 | 0.43 | 0.23 | SNP |
| chr6_32634755_C_T | 32634755 | 2.4 | 1.8 | 3.3 | 1.29E-08 | 0.24 | 0.43 | 0.23 | SNP |
| chr6_32604394_T_C | 32604394 | 2.5 | 1.8 | 3.5 | 1.38E-08 | 0.30 | 0.49 | 0.28 | SNP |
| chr6_32607047_T_G | 32607047 | 2.6 | 1.9 | 3.6 | 1.38E-08 | 0.32 | 0.51 | 0.30 | SNP |
| chr6_32629530_C_T | 32629530 | 2.4 | 1.8 | 3.2 | 1.40E-08 | 0.25 | 0.43 | 0.23 | SNP |
| chr6_32629422_T_C | 32629422 | 2.4 | 1.8 | 3.3 | 1.43E-08 | 0.24 | 0.42 | 0.22 | SNP |
| chr6_32635266_T_G | 32635266 | 2.5 | 1.8 | 3.4 | 1.57E-08 | 0.23 | 0.40 | 0.21 | SNP |
| chr6_32609456_C_T | 32609456 | 2.5 | 1.8 | 3.5 | 1.60E-08 | 0.30 | 0.49 | 0.28 | SNP |
| chr6_32612326_C_T | 32612326 | 2.5 | 1.8 | 3.5 | 1.63E-08 | 0.29 | 0.48 | 0.27 | SNP |
| chr6_32604108_T_C | 32604108 | 2.5 | 1.8 | 3.4 | 1.71E-08 | 0.30 | 0.49 | 0.28 | SNP |
| chr6_32608537_A_C | 32608537 | 2.5 | 1.8 | 3.4 | 1.72E-08 | 0.29 | 0.49 | 0.27 | SNP |
| chr6_32627833_A_G | 32627833 | 2.3 | 1.7 | 3.1 | 1.73E-08 | 0.42 | 0.58 | 0.40 | SNP |
| chr6_32635033_T_G | 32635033 | 2.4 | 1.8 | 3.2 | 2.01E-08 | 0.24 | 0.42 | 0.22 | SNP |
| chr6_32634094_T_C | 32634094 | 2.4 | 1.8 | 3.3 | 2.13E-08 | 0.24 | 0.42 | 0.22 | SNP |
| chr6_32636138_A_G | 32636138 | 2.4 | 1.8 | 3.2 | 2.28E-08 | 0.24 | 0.41 | 0.22 | SNP |
| chr6_32633767_G_T | 32633767 | 2.4 | 1.8 | 3.2 | 2.36E-08 | 0.24 | 0.42 | 0.22 | SNP |
| chr6_32634593_G_C | 32634593 | 2.4 | 1.8 | 3.2 | 2.39E-08 | 0.24 | 0.42 | 0.22 | SNP |
| chr6_32634098_C_G | 32634098 | 2.4 | 1.8 | 3.2 | 2.61E-08 | 0.24 | 0.42 | 0.23 | SNP |
| chr6_32606732_G_A | 32606732 | 2.4 | 1.8 | 3.4 | 2.77E-08 | 0.30 | 0.49 | 0.28 | SNP |
| chr6_32559124_T_C | 32559124 | 2.5 | 1.8 | 3.5 | 2.87E-08 | 0.28 | 0.47 | 0.26 | SNP |
| chr6_32634137_A_C | 32634137 | 2.4 | 1.7 | 3.2 | 2.92E-08 | 0.24 | 0.42 | 0.22 | SNP |
| chr6_32634091_G_C | 32634091 | 2.4 | 1.7 | 3.2 | 2.95E-08 | 0.24 | 0.42 | 0.22 | SNP |
| chr6_32634090_T_C | 32634090 | 2.4 | 1.7 | 3.2 | 3.00E-08 | 0.24 | 0.42 | 0.22 | SNP |
| chr6_32628189_A_G | 32628189 | 2.6 | 1.9 | 3.6 | 3.07E-08 | 0.21 | 0.38 | 0.19 | SNP |
| chr6_32636217_G_A | 32636217 | 2.4 | 1.8 | 3.2 | 3.12E-08 | 0.24 | 0.41 | 0.22 | SNP |
| chr6_32636154_C_G | 32636154 | 2.4 | 1.7 | 3.2 | 3.14E-08 | 0.24 | 0.41 | 0.22 | SNP |
| chr6_32633959_T_C | 32633959 | 2.3 | 1.7 | 3.2 | 3.24E-08 | 0.24 | 0.42 | 0.23 | SNP |
| chr6_32606942_G_A | 32606942 | 2.4 | 1.8 | 3.3 | 3.86E-08 | 0.30 | 0.49 | 0.28 | SNP |
| chr6_32604151_C_T | 32604151 | 2.5 | 1.8 | 3.4 | 4.01E-08 | 0.30 | 0.49 | 0.28 | SNP |
| chr6_32636063_G_A | 32636063 | 2.3 | 1.7 | 3.1 | 4.26E-08 | 0.24 | 0.42 | 0.22 | SNP |
| chr6_32634261_C_T | 32634261 | 2.3 | 1.7 | 3.2 | 4.79E-08 | 0.24 | 0.42 | 0.23 | SNP |
| chr6_32634262_T_G | 32634262 | 2.3 | 1.7 | 3.2 | 4.90E-08 | 0.24 | 0.42 | 0.23 | SNP |
| chr6_32605101_A_G | 32605101 | 2.4 | 1.8 | 3.3 | 4.93E-08 | 0.30 | 0.49 | 0.28 | SNP |
| chr6_32608483_T_C | 32608483 | 2.4 | 1.8 | 3.4 | 5.17E-08 | 0.29 | 0.48 | 0.27 | SNP |
| chr6_32609216_T_G | 32609216 | 0.4 | 0.3 | 0.5 | 5.37E-08 | 0.50 | 0.34 | 0.52 | SNP |
| chr6_32604259_C_T | 32604259 | 2.4 | 1.8 | 3.4 | 5.51E-08 | 0.30 | 0.48 | 0.28 | SNP |
| chr6_32520664_G_A | 32520664 | 2.4 | 1.8 | 3.4 | 5.65E-08 | 0.31 | 0.51 | 0.29 | SNP |
| chr6_32559673_T_G | 32559673 | 2.4 | 1.8 | 3.3 | 6.23E-08 | 0.32 | 0.49 | 0.30 | SNP |
| chr6_32487677_T_C | 32487677 | 3.1 | 2.0 | 4.6 | 6.28E-08 | 0.15 | 0.33 | 0.14 | SNP |
| chr6_32612279_T_C | 32612279 | 2.4 | 1.8 | 3.4 | 6.29E-08 | 0.29 | 0.48 | 0.27 | SNP |
| chr6_32559212_A_G | 32559212 | 2.5 | 1.8 | 3.4 | 6.38E-08 | 0.29 | 0.47 | 0.27 | SNP |
| chr6_32605418_G_A | 32605418 | 2.4 | 1.7 | 3.3 | 6.46E-08 | 0.30 | 0.49 | 0.28 | SNP |
| chr6_32636172_C_T | 32636172 | 2.3 | 1.7 | 3.1 | 6.69E-08 | 0.24 | 0.41 | 0.22 | SNP |
| chr6_32608473_G_A | 32608473 | 2.4 | 1.8 | 3.4 | 6.96E-08 | 0.29 | 0.48 | 0.28 | SNP |
| chr6_32605336_G_A | 32605336 | 2.4 | 1.7 | 3.3 | 7.06E-08 | 0.30 | 0.49 | 0.28 | SNP |
| chr6_32634139_C_T | 32634139 | 2.3 | 1.7 | 3.1 | 7.13E-08 | 0.24 | 0.42 | 0.23 | SNP |
| chr6_32608467_G_A | 32608467 | 2.4 | 1.8 | 3.4 | 7.28E-08 | 0.29 | 0.48 | 0.27 | SNP |
| chr6_32612251_T_G | 32612251 | 2.4 | 1.8 | 3.4 | 7.88E-08 | 0.29 | 0.48 | 0.27 | SNP |
| chr6_32628941_G_C | 32628941 | 2.3 | 1.7 | 3.2 | 7.96E-08 | 0.23 | 0.41 | 0.22 | SNP |
| chr6_32608844_A_G | 32608844 | 2.4 | 1.8 | 3.4 | 8.13E-08 | 0.28 | 0.47 | 0.26 | SNP |
| chr6_32610403_C_A | 32610403 | 2.5 | 1.8 | 3.4 | 8.79E-08 | 0.29 | 0.47 | 0.28 | SNP |
| chr6_32605646_T_G | 32605646 | 2.4 | 1.7 | 3.3 | 8.95E-08 | 0.31 | 0.49 | 0.29 | SNP |
| chr6_32634150_A_C | 32634150 | 2.3 | 1.7 | 3.1 | 9.08E-08 | 0.24 | 0.42 | 0.23 | SNP |
| chr6_32604059_A_G | 32604059 | 2.4 | 1.7 | 3.3 | 9.22E-08 | 0.30 | 0.48 | 0.28 | SNP |
| chr6_32606052_T_G | 32606052 | 2.4 | 1.7 | 3.3 | 9.66E-08 | 0.30 | 0.49 | 0.29 | SNP |
| chr6_32369488_G_A | 32369488 | 2.6 | 1.8 | 3.7 | 1.01E-07 | 0.09 | 0.18 | 0.08 | SNP |
| chr6_32605419_G_C | 32605419 | 2.4 | 1.7 | 3.3 | 1.06E-07 | 0.30 | 0.49 | 0.28 | SNP |
| chr6_32520621_G_A | 32520621 | 2.4 | 1.7 | 3.3 | 1.07E-07 | 0.31 | 0.51 | 0.29 | SNP |
| chr6_32611971_G_C | 32611971 | 2.5 | 1.8 | 3.5 | 1.10E-07 | 0.28 | 0.45 | 0.26 | SNP |
| chr6_32441150_C_T | 32441150 | 2.4 | 1.7 | 3.3 | 1.14E-07 | 0.30 | 0.48 | 0.28 | SNP |

| | | | | | | | | | |
|---|---|---|---|---|---|---|---|---|---|
| chr6_32520460_C_T | 32520460 | 2.4 | 1.7 | 3.3 | 1.17E-07 | 0.32 | 0.51 | 0.30 | SNP |
| chr6_32628953_G_C | 32628953 | 0.4 | 0.3 | 0.6 | 1.18E-07 | 0.49 | 0.32 | 0.50 | SNP |
| chr6_32520639_G_A | 32520639 | 2.4 | 1.7 | 3.3 | 1.21E-07 | 0.31 | 0.51 | 0.29 | SNP |
| chr6_32611650_G_A | 32611650 | 2.4 | 1.8 | 3.4 | 1.23E-07 | 0.28 | 0.46 | 0.27 | SNP |
| chr6_32607193_T_C | 32607193 | 2.5 | 1.8 | 3.4 | 1.25E-07 | 0.34 | 0.53 | 0.32 | SNP |
| chr6_32559575_A_G | 32559575 | 2.7 | 1.9 | 3.9 | 1.27E-07 | 0.20 | 0.36 | 0.19 | SNP |
| chr6_32612397_A_G | 32612397 | 2.5 | 1.8 | 3.5 | 1.29E-07 | 0.47 | 0.66 | 0.45 | SNP |
| chr6_32611243_C_T | 32611243 | 2.4 | 1.7 | 3.3 | 1.46E-07 | 0.28 | 0.46 | 0.27 | SNP |
| chr6_32609207_T_C | 32609207 | 0.4 | 0.3 | 0.6 | 1.47E-07 | 0.50 | 0.66 | 0.48 | SNP |
| chr6_32611723_T_G | 32611723 | 0.4 | 0.3 | 0.6 | 1.48E-07 | 0.47 | 0.31 | 0.49 | SNP |
| chr6_32608880_G_A | 32608880 | 2.4 | 1.7 | 3.4 | 1.65E-07 | 0.33 | 0.51 | 0.31 | SNP |
| chr6_32628195_A_G | 32628195 | 3.5 | 2.2 | 5.6 | 1.66E-07 | 0.04 | 0.11 | 0.03 | SNP |
| chr6_32612241_A_T | 32612241 | 2.4 | 1.7 | 3.3 | 1.67E-07 | 0.29 | 0.47 | 0.27 | SNP |
| chr6_32636221_G_T | 32636221 | 0.4 | 0.3 | 0.6 | 1.86E-07 | 0.45 | 0.30 | 0.47 | SNP |
| chr6_32376517_C_T | 32376517 | 2.6 | 1.8 | 3.7 | 1.89E-07 | 0.09 | 0.18 | 0.08 | SNP |
| chr6_32609453_C_T | 32609453 | 2.4 | 1.7 | 3.3 | 1.92E-07 | 0.33 | 0.52 | 0.32 | SNP |
| chr6_32628967_T_C | 32628967 | 0.4 | 0.3 | 0.6 | 1.92E-07 | 0.49 | 0.33 | 0.51 | SNP |
| chr6_32604538_G_A | 32604538 | 2.5 | 1.8 | 3.5 | 2.04E-07 | 0.33 | 0.50 | 0.31 | SNP |
| chr6_32608856_T_C | 32608856 | 2.4 | 1.7 | 3.3 | 2.22E-07 | 0.28 | 0.46 | 0.27 | SNP |
| chr6_32605274_C_T | 32605274 | 0.4 | 0.3 | 0.6 | 2.55E-07 | 0.45 | 0.29 | 0.47 | SNP |
| chr6_32611725_G_A | 32611725 | 0.4 | 0.3 | 0.6 | 2.80E-07 | 0.47 | 0.31 | 0.49 | SNP |
| chr6_32611234_C_T | 32611234 | 2.3 | 1.7 | 3.2 | 2.87E-07 | 0.29 | 0.46 | 0.27 | SNP |
| chr6_32605759_A_T | 32605759 | 0.4 | 0.3 | 0.6 | 3.10E-07 | 0.46 | 0.30 | 0.47 | SNP |
| chr6_32610968_A_G | 32610968 | 2.4 | 1.7 | 3.3 | 3.12E-07 | 0.27 | 0.45 | 0.25 | SNP |
| chr6_32604000_T_G | 32604000 | 0.4 | 0.3 | 0.6 | 3.20E-07 | 0.46 | 0.30 | 0.47 | SNP |
| chr6_32635949_G_A | 32635949 | 2.1 | 1.6 | 2.9 | 3.23E-07 | 0.29 | 0.46 | 0.27 | SNP |
| chr6_32521596_T_G | 32521596 | 2.4 | 1.7 | 3.4 | 3.91E-07 | 0.36 | 0.54 | 0.34 | SNP |
| chr6_32603968_G_A | 32603968 | 0.4 | 0.3 | 0.6 | 3.95E-07 | 0.45 | 0.30 | 0.47 | SNP |
| chr6_32604564_C_T | 32604564 | 2.4 | 1.7 | 3.3 | 4.02E-07 | 0.33 | 0.51 | 0.31 | SNP |
| chr6_32611760_G_T | 32611760 | 2.3 | 1.7 | 3.3 | 4.09E-07 | 0.29 | 0.47 | 0.27 | SNP |
| chr6_32609126_C_T | 32609126 | 0.4 | 0.3 | 0.6 | 4.28E-07 | 0.46 | 0.31 | 0.48 | SNP |
| chr6_32611260_A_G | 32611260 | 2.3 | 1.7 | 3.2 | 4.38E-07 | 0.29 | 0.46 | 0.27 | SNP |
| chr6_32606217_G_A | 32606217 | 2.3 | 1.7 | 3.1 | 4.43E-07 | 0.31 | 0.48 | 0.29 | SNP |
| chr6_32634838_C_A | 32634838 | 0.4 | 0.3 | 0.6 | 4.56E-07 | 0.45 | 0.30 | 0.47 | SNP |
| chr6_32612041_A_G | 32612041 | 2.3 | 1.7 | 3.2 | 4.70E-07 | 0.29 | 0.47 | 0.27 | SNP |
| chr6_32629802_G_A | 32629802 | 0.4 | 0.3 | 0.6 | 4.78E-07 | 0.45 | 0.30 | 0.47 | SNP |
| chr6_32636289_T_C | 32636289 | 2.3 | 1.7 | 3.1 | 4.88E-07 | 0.23 | 0.39 | 0.22 | SNP |
| chr6_32607174_C_T | 32607174 | 2.3 | 1.7 | 3.2 | 4.96E-07 | 0.35 | 0.52 | 0.33 | SNP |
| chr6_32607008_T_G | 32607008 | 0.4 | 0.3 | 0.6 | 5.06E-07 | 0.45 | 0.30 | 0.47 | SNP |
| chr6_32501301_G_C | 32501301 | 0.4 | 0.3 | 0.6 | 5.07E-07 | 0.39 | 0.23 | 0.40 | SNP |
| chr6_32520774_C_T | 32520774 | 2.3 | 1.7 | 3.2 | 5.17E-07 | 0.30 | 0.49 | 0.29 | SNP |
| chr6_32605754_A_T | 32605754 | 0.4 | 0.3 | 0.6 | 5.24E-07 | 0.46 | 0.30 | 0.47 | SNP |
| chr6_32612050_T_A | 32612050 | 0.4 | 0.3 | 0.6 | 5.31E-07 | 0.47 | 0.32 | 0.49 | SNP |
| chr6_32501296_G_A | 32501296 | 0.4 | 0.3 | 0.6 | 5.36E-07 | 0.39 | 0.23 | 0.40 | SNP |
| chr6_32604124_A_G | 32604124 | 0.4 | 0.3 | 0.6 | 5.37E-07 | 0.50 | 0.66 | 0.48 | SNP |
| chr6_32608417_G_A | 32608417 | 2.3 | 1.7 | 3.2 | 5.46E-07 | 0.29 | 0.47 | 0.27 | SNP |
| chr6_32610994_C_A | 32610994 | 2.3 | 1.7 | 3.2 | 5.54E-07 | 0.27 | 0.44 | 0.25 | SNP |
| chr6_32634834_T_C | 32634834 | 0.4 | 0.3 | 0.6 | 5.57E-07 | 0.45 | 0.30 | 0.47 | SNP |
| chr6_32608809_T_C | 32608809 | 2.3 | 1.7 | 3.2 | 5.66E-07 | 0.27 | 0.44 | 0.25 | SNP |
| chr6_32610059_G_T | 32610059 | 0.4 | 0.3 | 0.6 | 5.92E-07 | 0.45 | 0.30 | 0.47 | SNP |
| chr6_32501459_T_C | 32501459 | 0.4 | 0.3 | 0.6 | 5.99E-07 | 0.39 | 0.24 | 0.41 | SNP |
| chr6_32610997_C_G | 32610997 | 2.3 | 1.7 | 3.2 | 6.00E-07 | 0.27 | 0.44 | 0.25 | SNP |
| chr6_32559709_C_T | 32559709 | 0.4 | 0.3 | 0.6 | 6.04E-07 | 0.44 | 0.28 | 0.46 | SNP |
| chr6_32612166_C_T | 32612166 | 2.3 | 1.7 | 3.2 | 6.08E-07 | 0.29 | 0.47 | 0.27 | SNP |
| chr6_32604372_A_G | 32604372 | 0.4 | 0.3 | 0.6 | 6.17E-07 | 0.46 | 0.31 | 0.48 | SNP |
| chr6_32604152_A_G | 32604152 | 2.2 | 1.6 | 3.1 | 6.67E-07 | 0.46 | 0.62 | 0.44 | SNP |
| chr6_32612008_T_C | 32612008 | 2.3 | 1.7 | 3.2 | 6.74E-07 | 0.29 | 0.46 | 0.27 | SNP |
| chr6_32609105_A_G | 32609105 | 0.4 | 0.3 | 0.6 | 6.78E-07 | 0.46 | 0.31 | 0.48 | SNP |
| chr6_32612161_C_A | 32612161 | 2.3 | 1.7 | 3.2 | 6.89E-07 | 0.29 | 0.47 | 0.27 | SNP |
| chr6_32605920_A_G | 32605920 | 0.4 | 0.3 | 0.6 | 6.99E-07 | 0.45 | 0.30 | 0.47 | SNP |
| chr6_32612111_G_A | 32612111 | 2.3 | 1.7 | 3.2 | 7.02E-07 | 0.29 | 0.46 | 0.27 | SNP |
| chr6_32559714_G_A | 32559714 | 0.4 | 0.3 | 0.6 | 7.19E-07 | 0.45 | 0.29 | 0.47 | SNP |
| chr6_32603412_T_C | 32603412 | 2.1 | 1.6 | 2.9 | 7.21E-07 | 0.48 | 0.66 | 0.46 | SNP |
| chr6_32605943_A_G | 32605943 | 0.4 | 0.3 | 0.6 | 7.39E-07 | 0.45 | 0.30 | 0.47 | SNP |
| chr6_32612104_C_T | 32612104 | 2.3 | 1.6 | 3.2 | 7.54E-07 | 0.29 | 0.46 | 0.27 | SNP |
| chr6_32636434_G_A | 32636434 | 0.4 | 0.3 | 0.6 | 7.71E-07 | 0.46 | 0.30 | 0.47 | SNP |
| chr6_32627714_G_T | 32627714 | 0.4 | 0.3 | 0.6 | 7.87E-07 | 0.45 | 0.30 | 0.47 | SNP |
| chr6_32606176_T_A | 32606176 | 0.4 | 0.3 | 0.6 | 8.14E-07 | 0.45 | 0.30 | 0.47 | SNP |
| chr6_32611964_C_T | 32611964 | 2.3 | 1.7 | 3.3 | 8.56E-07 | 0.28 | 0.45 | 0.27 | SNP |
| chr6_32605705_T_G | 32605705 | 0.4 | 0.3 | 0.6 | 8.94E-07 | 0.46 | 0.31 | 0.47 | SNP |
| chr6_32605708_A_G | 32605708 | 0.4 | 0.3 | 0.6 | 9.08E-07 | 0.46 | 0.31 | 0.47 | SNP |
| chr6_32609286_T_C | 32609286 | 0.4 | 0.3 | 0.6 | 9.88E-07 | 0.49 | 0.65 | 0.47 | SNP |
| chr6_32603487_C_T | 32603487 | 0.4 | 0.3 | 0.6 | 1.02E-06 | 0.45 | 0.30 | 0.47 | SNP |
| chr6_32636280_G_T | 32636280 | 2.2 | 1.6 | 3.1 | 1.04E-06 | 0.23 | 0.39 | 0.22 | SNP |
| chr6_32441008_C_T | 32441008 | 2.2 | 1.6 | 3.1 | 1.09E-06 | 0.31 | 0.48 | 0.29 | SNP |
| chr6_32629314_A_G | 32629314 | 0.4 | 0.3 | 0.6 | 1.13E-06 | 0.47 | 0.32 | 0.49 | SNP |
| chr6_32603098_T_C | 32603098 | 2.1 | 1.6 | 2.8 | 1.13E-06 | 0.48 | 0.66 | 0.46 | SNP |
| chr6_32501311_C_T | 32501311 | 0.4 | 0.3 | 0.6 | 1.14E-06 | 0.38 | 0.23 | 0.40 | SNP |
| chr6_32627600_T_G | 32627600 | 0.4 | 0.3 | 0.6 | 1.18E-06 | 0.45 | 0.30 | 0.47 | SNP |
| chr6_32606085_T_A | 32606085 | 0.4 | 0.3 | 0.6 | 1.19E-06 | 0.45 | 0.30 | 0.47 | SNP |
| chr6_32633617_A_G | 32633617 | 0.4 | 0.3 | 0.6 | 1.19E-06 | 0.44 | 0.30 | 0.46 | SNP |
| chr6_32550819_C_A | 32550819 | 2.2 | 1.6 | 3.1 | 1.20E-06 | 0.29 | 0.47 | 0.27 | SNP |
| chr6_32610860_G_T | 32610860 | 2.2 | 1.6 | 3.1 | 1.21E-06 | 0.29 | 0.46 | 0.27 | SNP |
| chr6_32627635_G_T | 32627635 | 0.4 | 0.3 | 0.6 | 1.28E-06 | 0.45 | 0.30 | 0.46 | SNP |
| chr6_32627489_G_A | 32627489 | 0.4 | 0.3 | 0.6 | 1.32E-06 | 0.43 | 0.28 | 0.45 | SNP |
| chr6_32627535_T_G | 32627535 | 0.4 | 0.3 | 0.6 | 1.37E-06 | 0.46 | 0.30 | 0.47 | SNP |
| chr6_32609514_T_C | 32609514 | 0.4 | 0.3 | 0.6 | 1.46E-06 | 0.46 | 0.31 | 0.47 | SNP |
| chr6_32627646_G_A | 32627646 | 0.4 | 0.3 | 0.6 | 1.51E-06 | 0.45 | 0.30 | 0.47 | SNP |
| chr6_32520665_A_C | 32520665 | 0.4 | 0.3 | 0.6 | 1.52E-06 | 0.37 | 0.23 | 0.39 | SNP |
| chr6_32520595_G_A | 32520595 | 0.4 | 0.3 | 0.6 | 1.56E-06 | 0.38 | 0.23 | 0.39 | SNP |
| chr6_32609312_C_A | 32609312 | 0.4 | 0.3 | 0.6 | 1.74E-06 | 0.46 | 0.30 | 0.48 | SNP |
| chr6_32611229_T_G | 32611229 | 0.4 | 0.3 | 0.6 | 1.80E-06 | 0.46 | 0.31 | 0.48 | SNP |
| chr6_32611034_T_A | 32611034 | 2.2 | 1.6 | 3.0 | 1.86E-06 | 0.27 | 0.44 | 0.26 | SNP |
| chr6_32635226_G_T | 32635226 | 0.4 | 0.3 | 0.6 | 1.88E-06 | 0.46 | 0.32 | 0.48 | SNP |
| chr6_32629347_T_C | 32629347 | 2.2 | 1.6 | 3.1 | 2.09E-06 | 0.37 | 0.54 | 0.35 | SNP |
| chr6_32604642_A_G | 32604642 | 2.3 | 1.6 | 3.2 | 2.09E-06 | 0.28 | 0.45 | 0.26 | SNP |
| chr6_32611769_G_C | 32611769 | 2.3 | 1.6 | 3.2 | 2.16E-06 | 0.31 | 0.47 | 0.29 | SNP |
| chr6_32627485_C_T | 32627485 | 0.4 | 0.3 | 0.6 | 2.16E-06 | 0.43 | 0.28 | 0.45 | SNP |
| chr6_32520337_A_G | 32520337 | 2.4 | 1.7 | 3.4 | 2.17E-06 | 0.32 | 0.51 | 0.30 | SNP |
| chr6_32628784_A_C | 32628784 | 0.4 | 0.3 | 0.6 | 2.26E-06 | 0.48 | 0.32 | 0.49 | SNP |
| chr6_32612003_T_C | 32612003 | 0.4 | 0.3 | 0.6 | 2.27E-06 | 0.47 | 0.33 | 0.49 | SNP |
| chr6_32611265_C_T | 32611265 | 2.2 | 1.6 | 3.0 | 2.44E-06 | 0.29 | 0.45 | 0.27 | SNP |
| chr6_32550947_A_G | 32550947 | 2.2 | 1.6 | 3.1 | 2.54E-06 | 0.22 | 0.42 | 0.20 | SNP |
| chr6_32611958_C_G | 32611958 | 2.3 | 1.6 | 3.2 | 2.70E-06 | 0.28 | 0.44 | 0.27 | SNP |
| chr6_32634103_C_T | 32634103 | 0.4 | 0.3 | 0.6 | 2.71E-06 | 0.45 | 0.30 | 0.47 | SNP |
| chr6_32629467_G_A | 32629467 | 2.2 | 1.6 | 3.0 | 2.87E-06 | 0.37 | 0.54 | 0.35 | SNP |
| chr6_32636385_A_T | 32636385 | 2.0 | 1.5 | 2.6 | 3.02E-06 | 0.38 | 0.51 | 0.36 | SNP |
| chr6_32551568_C_G | 32551568 | 0.4 | 0.3 | 0.6 | 3.05E-06 | 0.38 | 0.24 | 0.40 | SNP |
| chr6_32610934_A_G | 32610934 | 2.3 | 1.6 | 3.2 | 3.15E-06 | 0.31 | 0.47 | 0.29 | SNP |
| chr6_32606701_A_G | 32606701 | 0.3 | 0.2 | 0.5 | 3.24E-06 | 0.17 | 0.07 | 0.18 | SNP |
| chr6_32627517_G_T | 32627517 | 0.4 | 0.3 | 0.6 | 3.33E-06 | 0.45 | 0.31 | 0.47 | SNP |
| chr6_32628776_A_C | 32628776 | 0.4 | 0.3 | 0.6 | 3.40E-06 | 0.48 | 0.33 | 0.49 | SNP |
| chr6_32611931_T_A | 32611931 | 0.4 | 0.3 | 0.6 | 3.44E-06 | 0.47 | 0.32 | 0.48 | SNP |
| chr6_32611873_C_T | 32611873 | 0.4 | 0.3 | 0.6 | 3.48E-06 | 0.47 | 0.32 | 0.48 | SNP |
| chr6_32611874_T_G | 32611874 | 0.4 | 0.3 | 0.6 | 3.48E-06 | 0.47 | 0.32 | 0.48 | SNP |
| chr6_32629331_A_G | 32629331 | 2.2 | 1.6 | 3.1 | 3.66E-06 | 0.36 | 0.53 | 0.34 | SNP |
| chr6_32559045_T_C | 32559045 | 0.5 | 0.3 | 0.6 | 3.85E-06 | 0.43 | 0.29 | 0.44 | SNP |
| chr6_32521117_T_C | 32521117 | 0.5 | 0.3 | 0.6 | 3.93E-06 | 0.49 | 0.32 | 0.51 | SNP |
| chr6_32609427_C_T | 32609427 | 0.4 | 0.3 | 0.6 | 4.04E-06 | 0.46 | 0.31 | 0.47 | SNP |
| chr6_32609380_C_T | 32609380 | 2.3 | 1.6 | 3.3 | 4.23E-06 | 0.35 | 0.50 | 0.34 | SNP |
| chr6_32605295_C_T | 32605295 | 2.1 | 1.5 | 2.8 | 4.24E-06 | 0.32 | 0.50 | 0.30 | SNP |
| chr6_32526540_A_G | 32526540 | 0.5 | 0.3 | 0.6 | 4.34E-06 | 0.41 | 0.27 | 0.42 | SNP |
| chr6_32603029_T_C | 32603029 | 0.4 | 0.3 | 0.6 | 4.38E-06 | 0.27 | 0.13 | 0.29 | SNP |
| chr6_32611450_T_G | 32611450 | 0.4 | 0.3 | 0.6 | 4.40E-06 | 0.48 | 0.33 | 0.49 | SNP |
| chr6_32636364_G_A | 32636364 | 1.9 | 1.5 | 2.5 | 4.45E-06 | 0.38 | 0.51 | 0.37 | SNP |
| chr6_32609358_C_T | 32609358 | 2.2 | 1.6 | 3.0 | 4.63E-06 | 0.29 | 0.45 | 0.27 | SNP |
| chr6_32634387_T_C | 32634387 | 0.4 | 0.3 | 0.6 | 4.86E-06 | 0.31 | 0.20 | 0.32 | SNP |
| chr6_32520465_A_G | 32520465 | 0.5 | 0.3 | 0.6 | 5.71E-06 | 0.38 | 0.24 | 0.39 | SNP |
| chr6_32612410_T_C | 32612410 | 0.4 | 0.3 | 0.6 | 5.85E-06 | 0.45 | 0.29 | 0.46 | SNP |
| chr6_32611474_G_A | 32611474 | 2.1 | 1.5 | 3.0 | 5.93E-06 | 0.27 | 0.44 | 0.25 | SNP |
| chr6_32604644_G_A | 32604644 | 2.2 | 1.6 | 3.1 | 6.05E-06 | 0.28 | 0.45 | 0.27 | SNP |
| chr6_32603488_A_G | 32603488 | 0.4 | 0.3 | 0.6 | 6.13E-06 | 0.28 | 0.13 | 0.29 | SNP |
| chr6_32603355_T_C | 32603355 | 0.4 | 0.3 | 0.6 | 6.17E-06 | 0.28 | 0.13 | 0.29 | SNP |
| chr6_32500925_A_C | 32500925 | 0.4 | 0.2 | 0.6 | 6.31E-06 | 0.19 | 0.08 | 0.20 | SNP |
| chr6_32611466_A_G | 32611466 | 2.2 | 1.5 | 3.0 | 6.32E-06 | 0.27 | 0.44 | 0.25 | SNP |
| chr6_32627795_A_G | 32627795 | 1.9 | 1.5 | 2.6 | 6.33E-06 | 0.47 | 0.61 | 0.46 | SNP |
| chr6_32611968_T_G | 32611968 | 0.5 | 0.3 | 0.6 | 6.57E-06 | 0.48 | 0.34 | 0.49 | SNP |
| chr6_32501245_A_G | 32501245 | 0.4 | 0.2 | 0.6 | 7.05E-06 | 0.19 | 0.08 | 0.20 | SNP |
| chr6_32075563_T_C | 32075563 | 0.5 | 0.4 | 0.7 | 7.12E-06 | 0.48 | 0.42 | 0.49 | SNP |
| chr6_32628737_G_A | 32628737 | 0.5 | 0.3 | 0.6 | 7.52E-06 | 0.47 | 0.33 | 0.49 | SNP |
| chr6_31321267_A_C | 31321267 | 4.6 | 2.3 | 8.9 | 7.57E-06 | 0.12 | 0.13 | 0.12 | SNP |
| chr6_32610769_C_A | 32610769 | 0.4 | 0.3 | 0.6 | 7.77E-06 | 0.33 | 0.23 | 0.34 | SNP |
| chr6_32629509_A_C | 32629509 | 2.1 | 1.5 | 3.0 | 8.07E-06 | 0.37 | 0.54 | 0.36 | SNP |
| chr6_32558183_G_T | 32558183 | 2.0 | 1.5 | 2.8 | 8.57E-06 | 0.49 | 0.63 | 0.48 | SNP |
| chr6_32627747_G_A | 32627747 | 0.4 | 0.3 | 0.6 | 8.79E-06 | 0.30 | 0.20 | 0.32 | SNP |
| chr6_32608820_T_C | 32608820 | 0.5 | 0.3 | 0.6 | 9.00E-06 | 0.46 | 0.32 | 0.48 | SNP |
| chr6_32636040_C_T | 32636040 | 0.4 | 0.3 | 0.6 | 9.19E-06 | 0.31 | 0.21 | 0.32 | SNP |
| chr6_32628736_T_C | 32628736 | 0.5 | 0.3 | 0.6 | 9.54E-06 | 0.47 | 0.33 | 0.49 | SNP |

| | | | | | | | | | |
|---|---|---|---|---|---|---|---|---|---|
| chr6_31473746_G_A | 31473746 | 4.6 | 2.3 | 9.0 | 9.76E-06 | 0.12 | 0.14 | 0.12 | SNP |
| chr6_32629582_T_C | 32629582 | 0.4 | 0.3 | 0.6 | 1.02E-05 | 0.31 | 0.21 | 0.32 | SNP |
| chr6_32550931_C_A | 32550931 | 2.1 | 1.5 | 3.0 | 1.09E-05 | 0.23 | 0.42 | 0.21 | SNP |
| chr6_32521951_A_C | 32521951 | 0.5 | 0.3 | 0.6 | 1.10E-05 | 0.37 | 0.23 | 0.39 | SNP |
| chr6_32633816_C_G | 32633816 | 1.9 | 1.4 | 2.5 | 1.13E-05 | 0.38 | 0.50 | 0.36 | SNP |
| chr6_31323416_G_C | 31323416 | 4.1 | 2.2 | 7.6 | 1.14E-05 | 0.12 | 0.14 | 0.12 | SNP |
| xHLA_2field_C_07_01 | 31236526 | 2.7 | 1.7 | 4.2 | 1.20E-05 | 0.14 | 0.18 | 0.14 | HLA (xHLA) |
| chr6_32629535_C_A | 32629535 | 2.1 | 1.5 | 2.9 | 1.23E-05 | 0.37 | 0.53 | 0.35 | SNP |
| chr6_32633665_T_C | 32633665 | 0.4 | 0.3 | 0.6 | 1.24E-05 | 0.31 | 0.21 | 0.32 | SNP |
| chr6_32628751_T_C | 32628751 | 0.5 | 0.3 | 0.7 | 1.26E-05 | 0.47 | 0.33 | 0.49 | SNP |
| chr6_31326703_T_C | 31326703 | 4.4 | 2.2 | 8.5 | 1.37E-05 | 0.12 | 0.13 | 0.12 | SNP |
| chr6_32629297_C_T | 32629297 | 0.5 | 0.3 | 0.7 | 1.42E-05 | 0.45 | 0.30 | 0.46 | SNP |
| chr6_32610704_C_T | 32610704 | 0.4 | 0.3 | 0.6 | 1.49E-05 | 0.33 | 0.22 | 0.34 | SNP |
| chr6_32796310_A_G | 32796310 | 2.9 | 1.8 | 4.8 | 1.58E-05 | 0.03 | 0.09 | 0.03 | SNP |
| chr6_32627561_C_T | 32627561 | 0.4 | 0.3 | 0.6 | 1.59E-05 | 0.32 | 0.22 | 0.33 | SNP |
| chr6_32629764_C_T | 32629764 | 2.0 | 1.5 | 2.8 | 1.90E-05 | 0.37 | 0.54 | 0.36 | SNP |
| chr6_32611427_T_A | 32611427 | 2.1 | 1.5 | 3.0 | 1.93E-05 | 0.27 | 0.43 | 0.26 | SNP |
| chr6_32628420_C_T | 32628420 | 2.1 | 1.5 | 2.9 | 1.94E-05 | 0.36 | 0.52 | 0.34 | SNP |
| chr6_32634010_A_G | 32634010 | 0.4 | 0.3 | 0.6 | 1.94E-05 | 0.31 | 0.21 | 0.32 | SNP |
| chr6_32629320_G_A | 32629320 | 0.4 | 0.3 | 0.6 | 1.95E-05 | 0.33 | 0.22 | 0.34 | SNP |
| chr6_31326074_A_T | 31326074 | 4.3 | 2.2 | 8.4 | 2.03E-05 | 0.12 | 0.13 | 0.11 | SNP |
| chr6_32629409_G_A | 32629409 | 0.4 | 0.3 | 0.6 | 2.08E-05 | 0.32 | 0.21 | 0.33 | SNP |
| chr6_32553418_T_G | 32553418 | 0.5 | 0.3 | 0.7 | 2.22E-05 | 0.41 | 0.27 | 0.42 | SNP |
| chr6_32629400_A_G | 32629400 | 2.0 | 1.5 | 2.9 | 2.26E-05 | 0.37 | 0.53 | 0.35 | SNP |
| chr6_32633909_T_A | 32633909 | 0.5 | 0.3 | 0.6 | 2.35E-05 | 0.33 | 0.23 | 0.34 | SNP |
| chr6_31323506_C_T | 31323506 | 3.9 | 2.1 | 7.4 | 2.38E-05 | 0.12 | 0.13 | 0.12 | SNP |
| chr6_32633906_A_C | 32633906 | 0.5 | 0.3 | 0.6 | 2.46E-05 | 0.33 | 0.23 | 0.34 | SNP |
| xHLA_2field_B_08_01 | 31321652 | 4.0 | 2.1 | 7.7 | 2.49E-05 | 0.12 | 0.14 | 0.11 | HLA (xHLA) |
| chr6_32632777_C_T | 32632777 | 0.2 | 0.1 | 0.4 | 2.53E-05 | 0.14 | 0.04 | 0.15 | SNP |
| chr6_32611403_G_A | 32611403 | 2.0 | 1.5 | 2.8 | 2.71E-05 | 0.27 | 0.44 | 0.25 | SNP |
| chr6_32628845_G_A | 32628845 | 2.0 | 1.5 | 2.8 | 2.72E-05 | 0.37 | 0.53 | 0.35 | SNP |
| chr6_32611418_A_C | 32611418 | 2.1 | 1.5 | 2.9 | 3.29E-05 | 0.27 | 0.43 | 0.26 | SNP |
| chr6_31129310_C_T | 31129310 | 3.9 | 2.0 | 7.3 | 3.62E-05 | 0.12 | 0.14 | 0.12 | SNP |
| chr6_32636494_C_T | 32636494 | 0.5 | 0.3 | 0.7 | 3.77E-05 | 0.43 | 0.29 | 0.44 | SNP |
| chr6_31322790_T_A | 31322790 | 3.8 | 2.0 | 7.0 | 3.87E-05 | 0.12 | 0.14 | 0.12 | SNP |
| chr6_31558702_C_T | 31558702 | 4.3 | 2.1 | 8.5 | 4.00E-05 | 0.12 | 0.13 | 0.12 | SNP |
| chr6_32611510_C_G | 32611510 | 0.5 | 0.3 | 0.7 | 4.05E-05 | 0.47 | 0.34 | 0.48 | SNP |
| chr6_32634485_C_A | 32634485 | 0.5 | 0.3 | 0.7 | 4.41E-05 | 0.32 | 0.22 | 0.33 | SNP |
| chr6_32797488_T_C | 32797488 | 1.8 | 1.4 | 2.5 | 4.51E-05 | 0.31 | 0.41 | 0.30 | SNP |
| chr6_32605583_A_G | 32605583 | 1.8 | 1.4 | 2.4 | 4.61E-05 | 0.39 | 0.53 | 0.37 | SNP |
| chr6_31465047_G_T | 31465047 | 4.1 | 2.1 | 8.1 | 4.69E-05 | 0.12 | 0.13 | 0.12 | SNP |
| chr6_32627652_C_G | 32627652 | 2.0 | 1.4 | 2.7 | 4.90E-05 | 0.37 | 0.53 | 0.36 | SNP |
| chr6_32611504_C_T | 32611504 | 0.5 | 0.4 | 0.7 | 5.40E-05 | 0.47 | 0.34 | 0.49 | SNP |
| chr6_32636591_G_A | 32636591 | 0.4 | 0.3 | 0.7 | 5.56E-05 | 0.46 | 0.30 | 0.47 | SNP |
| chr6_31243895_T_C | 31243895 | 1.9 | 1.4 | 2.5 | 5.82E-05 | 0.39 | 0.43 | 0.38 | SNP |
| chr6_32628944_C_G | 32628944 | 2.0 | 1.4 | 2.8 | 6.52E-05 | 0.35 | 0.51 | 0.34 | SNP |
| chr6_31243830_A_G | 31243830 | 1.9 | 1.4 | 2.5 | 6.81E-05 | 0.39 | 0.44 | 0.38 | SNP |
| chr6_32603660_T_C | 32603660 | 1.8 | 1.3 | 2.4 | 7.23E-05 | 0.39 | 0.53 | 0.38 | SNP |
| chr6_32340070_C_T | 32340070 | 1.8 | 1.4 | 2.5 | 7.68E-05 | 0.39 | 0.53 | 0.38 | SNP |
| chr6_31708463_C_A | 31708463 | 4.6 | 2.2 | 9.9 | 7.90E-05 | 0.12 | 0.13 | 0.12 | SNP |
| chr6_32611472_G_A | 32611472 | 0.5 | 0.4 | 0.7 | 8.27E-05 | 0.47 | 0.33 | 0.48 | SNP |
| chr6_31514247_A_G | 31514247 | 4.0 | 2.0 | 8.1 | 8.82E-05 | 0.12 | 0.13 | 0.12 | SNP |
| chr6_31244021_A_G | 31244021 | 2.2 | 1.5 | 3.3 | 9.23E-05 | 0.32 | 0.35 | 0.31 | SNP |
| chr6_31240060_G_A | 31240060 | 2.2 | 1.5 | 3.2 | 0.000104 | 0.33 | 0.36 | 0.32 | SNP |
| chr6_31721033_G_A | 31721033 | 4.5 | 2.1 | 9.7 | 0.000107 | 0.12 | 0.13 | 0.12 | SNP |
| chr6_32627456_A_G | 32627456 | 0.5 | 0.3 | 0.7 | 0.000108 | 0.36 | 0.25 | 0.37 | SNP |
| chr6_31085356_C_T | 31085356 | 3.3 | 1.8 | 6.1 | 0.000109 | 0.12 | 0.13 | 0.12 | SNP |
| chr6_32629859_G_A | 32629859 | 0.5 | 0.4 | 0.7 | 0.000109 | 0.48 | 0.36 | 0.50 | SNP |
| chr6_31921486_A_G | 31921486 | 3.8 | 1.9 | 7.3 | 0.000111 | 0.01 | 0.04 | 0.01 | SNP |
| chr6_32609094_C_T | 32609094 | 1.9 | 1.4 | 2.6 | 0.000114 | 0.35 | 0.50 | 0.34 | SNP |
| chr6_31243884_G_A | 31243884 | 1.9 | 1.4 | 2.6 | 0.000119 | 0.21 | 0.25 | 0.21 | SNP |
| chr6_31430065_C_T | 31430065 | 3.9 | 1.9 | 7.8 | 0.000142 | 0.12 | 0.13 | 0.11 | SNP |
| chr6_31237048_G_A | 31237048 | 2.0 | 1.4 | 2.8 | 0.000157 | 0.34 | 0.38 | 0.34 | SNP |
| chr6_31473957_C_G | 31473957 | 3.6 | 1.8 | 6.9 | 0.000160 | 0.12 | 0.13 | 0.12 | SNP |
| chr6_32407068_G_A | 32407068 | 3.0 | 1.7 | 5.1 | 0.000161 | 0.02 | 0.06 | 0.02 | SNP |
| chr6_32405821_T_C | 32405821 | 1.9 | 1.4 | 2.7 | 0.000187 | 0.24 | 0.39 | 0.23 | SNP |
| chr6_31636742_A_G | 31636742 | 4.1 | 1.9 | 8.5 | 0.000187 | 0.12 | 0.13 | 0.12 | SNP |
| chr6_32184705_A_G | 32184705 | 1.7 | 1.3 | 2.3 | 0.000188 | 0.33 | 0.39 | 0.33 | SNP |
| chr6_32635713_C_T | 32635713 | 0.5 | 0.4 | 0.7 | 0.000194 | 0.48 | 0.34 | 0.49 | SNP |
| chr6_31238029_C_T | 31238029 | 2.0 | 1.4 | 2.8 | 0.000194 | 0.36 | 0.39 | 0.35 | SNP |
| chr6_31430010_G_A | 31430010 | 3.7 | 1.8 | 7.3 | 0.000206 | 0.12 | 0.13 | 0.12 | SNP |
| chr6_31237323_G_A | 31237323 | 2.0 | 1.4 | 2.8 | 0.000211 | 0.35 | 0.38 | 0.34 | SNP |
| chr6_31243979_A_T | 31243979 | 2.1 | 1.4 | 3.2 | 0.000218 | 0.32 | 0.35 | 0.32 | SNP |
| chr6_31705864_C_T | 31705864 | 4.0 | 1.9 | 8.4 | 0.000227 | 0.12 | 0.13 | 0.12 | SNP |
| chr6_31712196_G_A | 31712196 | 4.0 | 1.9 | 8.4 | 0.000227 | 0.12 | 0.13 | 0.12 | SNP |
| chr6_31725230_G_A | 31725230 | 4.0 | 1.9 | 8.4 | 0.000227 | 0.12 | 0.13 | 0.12 | SNP |
| chr6_31726794_T_C | 31726794 | 4.0 | 1.9 | 8.4 | 0.000227 | 0.12 | 0.13 | 0.12 | SNP |
| chr6_31727474_A_G | 31727474 | 4.0 | 1.9 | 8.4 | 0.000227 | 0.12 | 0.13 | 0.12 | SNP |
| chr6_31725285_A_G | 31725285 | 4.0 | 1.9 | 8.3 | 0.000236 | 0.12 | 0.13 | 0.12 | SNP |
| chr6_31765984_G_A | 31765984 | 4.5 | 2.0 | 9.9 | 0.000243 | 0.12 | 0.12 | 0.12 | SNP |
| chr6_31237233_G_A | 31237233 | 2.0 | 1.4 | 2.8 | 0.000264 | 0.35 | 0.39 | 0.35 | SNP |
| chr6_32606695_T_C | 32606695 | 0.3 | 0.2 | 0.6 | 0.000270 | 0.11 | 0.04 | 0.12 | SNP |
| chr6_31237333_A_T | 31237333 | 2.0 | 1.4 | 2.8 | 0.000282 | 0.35 | 0.38 | 0.34 | SNP |
| chr6_32190969_C_T | 32190969 | 3.5 | 1.7 | 6.7 | 0.000308 | 0.01 | 0.04 | 0.01 | SNP |
| chr6_31237449_C_T | 31237449 | 2.0 | 1.4 | 3.0 | 0.000312 | 0.32 | 0.35 | 0.31 | SNP |
| chr6_32376019_G_T | 32376019 | 0.3 | 0.2 | 0.6 | 0.000312 | 0.11 | 0.04 | 0.11 | SNP |
| chr6_32609341_T_C | 32609341 | 0.5 | 0.4 | 0.8 | 0.000314 | 0.48 | 0.35 | 0.49 | SNP |
| chr6_31237353_C_T | 31237353 | 1.9 | 1.3 | 2.8 | 0.000317 | 0.34 | 0.37 | 0.34 | SNP |
| chr6_32370684_A_G | 32370684 | 1.8 | 1.3 | 2.5 | 0.000320 | 0.27 | 0.42 | 0.26 | SNP |
| chr6_31241664_G_A | 31241664 | 2.3 | 1.5 | 3.7 | 0.000321 | 0.14 | 0.16 | 0.14 | SNP |
| chr6_31237061_T_C | 31237061 | 1.9 | 1.3 | 2.7 | 0.000327 | 0.36 | 0.39 | 0.35 | SNP |
| chr6_31243971_G_A | 31243971 | 2.1 | 1.4 | 3.1 | 0.000327 | 0.32 | 0.35 | 0.32 | SNP |
| chr6_32553662_A_G | 32553662 | 0.6 | 0.4 | 0.8 | 0.000338 | 0.38 | 0.28 | 0.40 | SNP |
| chr6_31237405_T_C | 31237405 | 1.9 | 1.3 | 2.8 | 0.000348 | 0.34 | 0.37 | 0.34 | SNP |
| chr6_31237230_G_A | 31237230 | 1.9 | 1.3 | 2.8 | 0.000356 | 0.35 | 0.38 | 0.35 | SNP |
| chr6_31239227_T_C | 31239227 | 2.1 | 1.4 | 3.2 | 0.000356 | 0.37 | 0.42 | 0.37 | SNP |
| chr6_32555317_C_T | 32555317 | 0.3 | 0.2 | 0.6 | 0.000363 | 0.10 | 0.04 | 0.10 | SNP |
| chr6_31237592_C_A | 31237592 | 1.9 | 1.3 | 2.8 | 0.000363 | 0.34 | 0.37 | 0.34 | SNP |
| chr6_31462135_G_A | 31462135 | 4.1 | 1.9 | 9.0 | 0.000366 | 0.13 | 0.14 | 0.12 | SNP |
| chr6_31237950_C_T | 31237950 | 1.9 | 1.3 | 2.8 | 0.000368 | 0.36 | 0.40 | 0.36 | SNP |
| chr6_32631039_G_A | 32631039 | 0.5 | 0.3 | 0.7 | 0.000370 | 0.37 | 0.27 | 0.38 | SNP |
| chr6_31709778_A_G | 31709778 | 3.9 | 1.8 | 8.4 | 0.000374 | 0.12 | 0.12 | 0.12 | SNP |
| chr6_31241092_G_C | 31241092 | 1.7 | 1.3 | 2.4 | 0.000379 | 0.38 | 0.41 | 0.38 | SNP |
| chr6_31239868_C_G | 31239868 | 1.9 | 1.3 | 2.8 | 0.000422 | 0.35 | 0.39 | 0.35 | SNP |
| chr6_31241311_A_G | 31241311 | 2.3 | 1.4 | 3.6 | 0.000433 | 0.14 | 0.16 | 0.14 | SNP |
| chr6_32611373_A_G | 32611373 | 0.5 | 0.4 | 0.7 | 0.000442 | 0.49 | 0.36 | 0.51 | SNP |
| chr6_32605508_G_T | 32605508 | 1.8 | 1.3 | 2.4 | 0.000445 | 0.38 | 0.53 | 0.36 | SNP |
| chr6_31238613_G_A | 31238613 | 1.9 | 1.3 | 2.7 | 0.000476 | 0.35 | 0.37 | 0.34 | SNP |
| chr6_31237162_G_C | 31237162 | 1.9 | 1.3 | 2.7 | 0.000479 | 0.34 | 0.37 | 0.34 | SNP |
| chr6_31237124_C_T | 31237124 | 1.9 | 1.3 | 2.7 | 0.000487 | 0.34 | 0.37 | 0.34 | SNP |
| chr6_31240041_T_C | 31240041 | 1.8 | 1.3 | 2.5 | 0.000489 | 0.41 | 0.43 | 0.41 | SNP |
| chr6_31246324_A_C | 31246324 | 2.3 | 1.4 | 3.7 | 0.000490 | 0.14 | 0.16 | 0.14 | SNP |
| chr6_32364356_A_G | 32364356 | 1.8 | 1.3 | 2.5 | 0.000495 | 0.28 | 0.42 | 0.26 | SNP |
| chr6_32376746_C_T | 32376746 | 1.8 | 1.3 | 2.4 | 0.000500 | 0.28 | 0.42 | 0.26 | SNP |
| chr6_32375352_A_C | 32375352 | 1.8 | 1.3 | 2.4 | 0.000510 | 0.29 | 0.43 | 0.27 | SNP |
| chr6_32605501_C_G | 32605501 | 1.7 | 1.3 | 2.3 | 0.000513 | 0.36 | 0.52 | 0.35 | SNP |
| chr6_32375371_A_T | 32375371 | 1.8 | 1.3 | 2.4 | 0.000524 | 0.29 | 0.43 | 0.27 | SNP |
| chr6_31238318_A_G | 31238318 | 1.9 | 1.3 | 2.7 | 0.000535 | 0.35 | 0.38 | 0.35 | SNP |
| chr6_32375374_G_A | 32375374 | 1.8 | 1.3 | 2.4 | 0.000572 | 0.29 | 0.43 | 0.28 | SNP |
| chr6_31237563_T_C | 31237563 | 1.9 | 1.3 | 2.7 | 0.000592 | 0.34 | 0.37 | 0.34 | SNP |
| chr6_32606406_A_G | 32606406 | 0.5 | 0.4 | 0.8 | 0.000607 | 0.38 | 0.27 | 0.39 | SNP |
| xHLA_2field_B_15_01 | 31321652 | 0.4 | 0.3 | 0.7 | 0.000608 | 0.10 | 0.08 | 0.10 | HLA (xHLA) |
| chr6_31238230_C_G | 31238230 | 1.9 | 1.3 | 2.7 | 0.000608 | 0.34 | 0.37 | 0.34 | SNP |
| chr6_32557962_A_G | 32557962 | 0.5 | 0.3 | 0.7 | 0.000616 | 0.27 | 0.18 | 0.28 | SNP |
| chr6_32376348_G_A | 32376348 | 1.8 | 1.3 | 2.5 | 0.000640 | 0.24 | 0.38 | 0.23 | SNP |
| chr6_32633819_A_G | 32633819 | 0.6 | 0.4 | 0.8 | 0.000664 | 0.50 | 0.61 | 0.49 | SNP |
| chr6_32375973_C_A | 32375973 | 1.8 | 1.3 | 2.5 | 0.000665 | 0.24 | 0.38 | 0.23 | SNP |
| chr6_32374622_A_T | 32374622 | 1.7 | 1.3 | 2.4 | 0.000665 | 0.28 | 0.42 | 0.26 | SNP |
| chr6_31238372_G_C | 31238372 | 1.9 | 1.3 | 2.7 | 0.000665 | 0.35 | 0.37 | 0.34 | SNP |
| chr6_31236854_T_C | 31236854 | 2.3 | 1.4 | 3.6 | 0.000691 | 0.14 | 0.16 | 0.14 | SNP |
| chr6_32376176_C_T | 32376176 | 1.7 | 1.3 | 2.4 | 0.000707 | 0.28 | 0.42 | 0.27 | SNP |
| chr6_32340654_G_A | 32340654 | 1.7 | 1.3 | 2.4 | 0.000730 | 0.28 | 0.42 | 0.26 | SNP |
| chr6_32370616_T_C | 32370616 | 1.8 | 1.3 | 2.4 | 0.000737 | 0.28 | 0.41 | 0.26 | SNP |
| chr6_31240293_T_C | 31240293 | 1.8 | 1.3 | 2.6 | 0.000743 | 0.35 | 0.37 | 0.34 | SNP |
| chr6_32375330_G_A | 32375330 | 1.7 | 1.3 | 2.4 | 0.000753 | 0.29 | 0.43 | 0.27 | SNP |
| chr6_32501522_A_G | 32501522 | 1.8 | 1.3 | 2.6 | 0.000773 | 0.23 | 0.30 | 0.23 | SNP |
| chr6_31727897_C_T | 31727897 | 3.6 | 1.7 | 7.6 | 0.000781 | 0.12 | 0.12 | 0.12 | SNP |
| chr6_31238297_T_C | 31238297 | 1.8 | 1.3 | 2.6 | 0.000789 | 0.35 | 0.37 | 0.34 | SNP |
| chr6_31243846_G_T | 31243846 | 2.3 | 1.4 | 3.6 | 0.000812 | 0.14 | 0.16 | 0.14 | SNP |
| chr6_32341719_C_T | 32341719 | 1.7 | 1.3 | 2.4 | 0.000819 | 0.28 | 0.41 | 0.26 | SNP |
| chr6_31238234_A_G | 31238234 | 1.9 | 1.3 | 2.7 | 0.000824 | 0.34 | 0.37 | 0.34 | SNP |
| chr6_31236524_G_A | 31236524 | 1.8 | 1.3 | 2.6 | 0.000831 | 0.35 | 0.37 | 0.35 | SNP |
| chr6_32786882_T_C | 32786882 | 1.9 | 1.3 | 2.8 | 0.000840 | 0.07 | 0.13 | 0.06 | SNP |
| chr6_31237437_C_T | 31237437 | 1.8 | 1.3 | 2.6 | 0.000865 | 0.35 | 0.37 | 0.34 | SNP |
| chr6_32339548_G_A | 32339548 | 1.7 | 1.2 | 2.2 | 0.000873 | 0.38 | 0.50 | 0.37 | SNP |
| chr6_31245796_C_T | 31245796 | 2.2 | 1.4 | 3.5 | 0.000881 | 0.14 | 0.16 | 0.14 | SNP |
| chr6_31378335_G_C | 31378335 | 0.5 | 0.3 | 0.7 | 0.000892 | 0.10 | 0.08 | 0.11 | SNP |
| chr6_31238524_A_G | 31238524 | 1.8 | 1.3 | 2.6 | 0.000904 | 0.36 | 0.38 | 0.36 | SNP |

| | | | | | | | | | |
|---|---|---|---|---|---|---|---|---|---|
| chr6_31325341_C_G | 31325341 | 1.9 | 1.3 | 2.7 | 0.000915 | 0.32 | 0.35 | 0.32 | SNP |
| chr6_32375424_G_A | 32375424 | 1.7 | 1.2 | 2.3 | 0.000917 | 0.29 | 0.43 | 0.28 | SNP |
| chr6_31243859_C_T | 31243859 | 1.8 | 1.3 | 2.4 | 0.000932 | 0.21 | 0.25 | 0.21 | SNP |
| chr6_32096001_C_A | 32096001 | 3.7 | 1.7 | 7.8 | 0.000942 | 0.12 | 0.13 | 0.12 | SNP |
| chr6_32089142_G_T | 32089142 | 0.2 | 0.1 | 0.5 | 0.000946 | 0.09 | 0.02 | 0.09 | SNP |
| chr6_31236523_G_A | 31236523 | 1.8 | 1.3 | 2.6 | 0.000955 | 0.35 | 0.37 | 0.35 | SNP |
| chr6_31324599_T_G | 31324599 | 0.5 | 0.3 | 0.7 | 0.000959 | 0.14 | 0.11 | 0.14 | SNP |
| chr6_32633467_A_G | 32633467 | 0.3 | 0.1 | 0.6 | 0.000967 | 0.09 | 0.03 | 0.10 | SNP |
| chr6_31726253_T_C | 31726253 | 3.5 | 1.7 | 7.5 | 0.000981 | 0.12 | 0.12 | 0.12 | SNP |
| chr6_32409656_G_A | 32409656 | 1.7 | 1.2 | 2.3 | 0.000984 | 0.35 | 0.50 | 0.34 | SNP |
| chr6_31325494_C_T | 31325494 | 1.9 | 1.3 | 2.7 | 0.001013 | 0.32 | 0.35 | 0.32 | SNP |
| chr6_32080383_C_T | 32080383 | 0.3 | 0.1 | 0.6 | 0.001035 | 0.08 | 0.03 | 0.09 | SNP |
| chr6_32374131_C_G | 32374131 | 1.7 | 1.2 | 2.4 | 0.001047 | 0.28 | 0.42 | 0.26 | SNP |
| chr6_29695180_G_A | 29695180 | 1.6 | 1.2 | 2.2 | 0.001058 | 0.23 | 0.28 | 0.22 | SNP |
| chr6_31325513_G_C | 31325513 | 1.9 | 1.3 | 2.7 | 0.001066 | 0.32 | 0.35 | 0.32 | SNP |
| chr6_31918860_A_G | 31918860 | 3.4 | 1.6 | 6.9 | 0.001072 | 0.12 | 0.12 | 0.12 | SNP |
| chr6_31237463_G_C | 31237463 | 1.8 | 1.3 | 2.6 | 0.001089 | 0.34 | 0.37 | 0.34 | SNP |
| chr6_32611152_G_A | 32611152 | 1.7 | 1.2 | 2.3 | 0.001097 | 0.21 | 0.35 | 0.20 | SNP |
| chr6_31237664_T_C | 31237664 | 1.8 | 1.3 | 2.6 | 0.001104 | 0.35 | 0.37 | 0.35 | SNP |
| chr6_31506854_T_C | 31506854 | 1.9 | 1.3 | 2.7 | 0.001107 | 0.23 | 0.29 | 0.23 | SNP |
| chr6_31378117_C_T | 31378117 | 0.4 | 0.3 | 0.7 | 0.001111 | 0.09 | 0.07 | 0.09 | SNP |
| chr6_32363844_C_T | 32363844 | 1.7 | 1.2 | 2.4 | 0.001114 | 0.28 | 0.41 | 0.26 | SNP |
| chr6_31325504_C_T | 31325504 | 1.9 | 1.3 | 2.7 | 0.001118 | 0.32 | 0.35 | 0.32 | SNP |
| chr6_31325380_T_C | 31325380 | 1.9 | 1.3 | 2.7 | 0.001125 | 0.32 | 0.35 | 0.32 | SNP |
| chr6_32558284_T_C | 32558284 | 0.3 | 0.1 | 0.6 | 0.001126 | 0.08 | 0.03 | 0.08 | SNP |
| chr6_32604213_A_C | 32604213 | 0.6 | 0.4 | 0.8 | 0.001135 | 0.40 | 0.28 | 0.41 | SNP |
| chr6_31236175_C_T | 31236175 | 0.6 | 0.5 | 0.8 | 0.001174 | 0.43 | 0.41 | 0.44 | SNP |
| chr6_32341473_T_C | 32341473 | 1.7 | 1.2 | 2.3 | 0.001176 | 0.28 | 0.42 | 0.26 | SNP |
| chr6_31924880_G_A | 31924880 | 0.3 | 0.1 | 0.6 | 0.001212 | 0.09 | 0.03 | 0.09 | SNP |
| chr6_32603825_C_T | 32603825 | 1.7 | 1.2 | 2.3 | 0.001213 | 0.40 | 0.55 | 0.39 | SNP |
| chr6_32375695_G_A | 32375695 | 1.7 | 1.2 | 2.3 | 0.001225 | 0.28 | 0.42 | 0.27 | SNP |
| chr6_32375280_C_G | 32375280 | 1.7 | 1.2 | 2.3 | 0.001227 | 0.29 | 0.43 | 0.27 | SNP |
| chr6_32607224_C_T | 32607224 | 0.3 | 0.1 | 0.6 | 0.001252 | 0.10 | 0.03 | 0.11 | SNP |
| chr6_31245821_A_G | 31245821 | 2.2 | 1.3 | 3.4 | 0.001275 | 0.14 | 0.16 | 0.14 | SNP |
| chr6_31239449_C_G | 31239449 | 2.0 | 1.3 | 3.1 | 0.001307 | 0.17 | 0.20 | 0.16 | SNP |
| chr6_31919956_T_C | 31919956 | 0.3 | 0.2 | 0.6 | 0.001307 | 0.08 | 0.03 | 0.09 | SNP |
| chr6_31916951_T_C | 31916951 | 0.3 | 0.2 | 0.6 | 0.001321 | 0.08 | 0.03 | 0.09 | SNP |
| chr6_31919917_T_C | 31919917 | 0.3 | 0.2 | 0.6 | 0.001321 | 0.08 | 0.03 | 0.09 | SNP |
| chr6_31910938_G_T | 31910938 | 0.3 | 0.2 | 0.6 | 0.001322 | 0.08 | 0.03 | 0.09 | SNP |
| chr6_31910929_G_A | 31910929 | 0.3 | 0.2 | 0.6 | 0.001331 | 0.08 | 0.03 | 0.09 | SNP |
| chr6_31237802_G_A | 31237802 | 1.8 | 1.3 | 2.6 | 0.001343 | 0.35 | 0.37 | 0.35 | SNP |
| chr6_31928306_A_G | 31928306 | 0.3 | 0.2 | 0.6 | 0.001349 | 0.08 | 0.03 | 0.09 | SNP |
| chr6_32634990_G_T | 32634990 | 0.3 | 0.2 | 0.6 | 0.001364 | 0.09 | 0.03 | 0.09 | SNP |
| chr6_32603321_C_T | 32603321 | 0.5 | 0.4 | 0.8 | 0.001376 | 0.22 | 0.11 | 0.23 | SNP |
| chr6_31429927_T_C | 31429927 | 2.0 | 1.3 | 3.2 | 0.001383 | 0.16 | 0.18 | 0.16 | SNP |
| chr6_31325532_A_G | 31325532 | 1.8 | 1.3 | 2.7 | 0.001395 | 0.32 | 0.35 | 0.32 | SNP |
| chr6_31325526_A_G | 31325526 | 1.8 | 1.3 | 2.7 | 0.001414 | 0.33 | 0.35 | 0.32 | SNP |
| chr6_31325529_C_T | 31325529 | 1.8 | 1.3 | 2.7 | 0.001414 | 0.33 | 0.35 | 0.32 | SNP |
| chr6_32607221_C_G | 32607221 | 0.3 | 0.1 | 0.6 | 0.001418 | 0.09 | 0.03 | 0.10 | SNP |
| chr6_31325521_C_G | 31325521 | 1.8 | 1.3 | 2.7 | 0.001420 | 0.33 | 0.35 | 0.32 | SNP |
| chr6_32020238_A_G | 32020238 | 2.2 | 1.3 | 3.5 | 0.001451 | 0.17 | 0.23 | 0.16 | SNP |
| chr6_32782030_A_C | 32782030 | 1.7 | 1.2 | 2.3 | 0.001485 | 0.18 | 0.22 | 0.18 | SNP |
| chr6_32491581_A_C | 32491581 | 1.7 | 1.2 | 2.5 | 0.001488 | 0.26 | 0.41 | 0.25 | SNP |
| chr6_31906865_C_T | 31906865 | 0.3 | 0.2 | 0.6 | 0.001493 | 0.08 | 0.03 | 0.09 | SNP |
| chr6_31905130_G_A | 31905130 | 0.3 | 0.2 | 0.6 | 0.001502 | 0.08 | 0.03 | 0.09 | SNP |
| chr6_31905328_G_T | 31905328 | 0.3 | 0.2 | 0.6 | 0.001502 | 0.08 | 0.03 | 0.09 | SNP |
| chr6_31907147_T_C | 31907147 | 0.3 | 0.2 | 0.6 | 0.001502 | 0.08 | 0.03 | 0.09 | SNP |
| chr6_31907168_C_T | 31907168 | 0.3 | 0.2 | 0.6 | 0.001502 | 0.08 | 0.03 | 0.09 | SNP |
| chr6_31322694_T_C | 31322694 | 0.5 | 0.3 | 0.8 | 0.001578 | 0.12 | 0.10 | 0.12 | SNP |
| chr6_31237660_G_A | 31237660 | 1.8 | 1.2 | 2.6 | 0.001585 | 0.35 | 0.37 | 0.35 | SNP |
| chr6_31502861_G_A | 31502861 | 1.9 | 1.3 | 2.7 | 0.001591 | 0.23 | 0.29 | 0.22 | SNP |
| chr6_32406530_G_A | 32406530 | 1.6 | 1.2 | 2.2 | 0.001618 | 0.35 | 0.50 | 0.34 | SNP |
| chr6_32609874_T_G | 32609874 | 0.3 | 0.2 | 0.6 | 0.001640 | 0.08 | 0.03 | 0.09 | SNP |
| chr6_32610075_A_G | 32610075 | 0.3 | 0.2 | 0.6 | 0.001640 | 0.08 | 0.03 | 0.09 | SNP |
| chr6_32610313_C_G | 32610313 | 0.3 | 0.2 | 0.6 | 0.001640 | 0.08 | 0.03 | 0.09 | SNP |
| chr6_31238535_C_T | 31238535 | 1.8 | 1.2 | 2.5 | 0.001686 | 0.36 | 0.38 | 0.36 | SNP |
| chr6_31325056_C_T | 31325056 | 2.1 | 1.3 | 3.3 | 0.001696 | 0.15 | 0.17 | 0.15 | SNP |
| chr6_32611759_T_G | 32611759 | 0.3 | 0.2 | 0.6 | 0.001698 | 0.09 | 0.03 | 0.09 | SNP |
| chr6_29693969_C_T | 29693969 | 1.6 | 1.2 | 2.2 | 0.001754 | 0.23 | 0.28 | 0.23 | SNP |
| chr6_32408527_C_G | 32408527 | 1.6 | 1.2 | 2.2 | 0.001754 | 0.35 | 0.50 | 0.34 | SNP |
| xHLA_2field_DQB1_06_03 | 32627244 | 0.3 | 0.1 | 0.6 | 0.001756 | 0.08 | 0.02 | 0.09 | HLA (xHLA) |
| chr6_29892815_A_G | 29892815 | 1.8 | 1.2 | 2.7 | 0.001762 | 0.15 | 0.18 | 0.14 | SNP |
| chr6_32078373_A_C | 32078373 | 1.8 | 1.2 | 2.5 | 0.001787 | 0.11 | 0.16 | 0.11 | SNP |
| chr6_32608934_A_T | 32608934 | 0.3 | 0.2 | 0.6 | 0.001788 | 0.08 | 0.03 | 0.09 | SNP |
| chr6_32077380_C_T | 32077380 | 0.3 | 0.2 | 0.6 | 0.001791 | 0.08 | 0.03 | 0.09 | SNP |
| chr6_31931137_T_C | 31931137 | 0.5 | 0.3 | 0.7 | 0.001821 | 0.12 | 0.06 | 0.13 | SNP |
| chr6_32608946_G_A | 32608946 | 0.3 | 0.2 | 0.6 | 0.001831 | 0.09 | 0.03 | 0.09 | SNP |
| chr6_32411833_C_T | 32411833 | 1.6 | 1.2 | 2.2 | 0.001834 | 0.40 | 0.54 | 0.38 | SNP |
| chr6_31238814_C_T | 31238814 | 1.8 | 1.2 | 2.5 | 0.001837 | 0.38 | 0.41 | 0.38 | SNP |
| chr6_32526702_C_T | 32526702 | 0.3 | 0.2 | 0.6 | 0.001840 | 0.08 | 0.03 | 0.09 | SNP |
| chr6_32610300_C_T | 32610300 | 0.3 | 0.2 | 0.6 | 0.001851 | 0.08 | 0.03 | 0.09 | SNP |
| chr6_32610688_G_C | 32610688 | 0.3 | 0.2 | 0.6 | 0.001858 | 0.08 | 0.03 | 0.09 | SNP |
| chr6_32612096_A_G | 32612096 | 0.3 | 0.2 | 0.6 | 0.001860 | 0.09 | 0.03 | 0.09 | SNP |
| chr6_29893960_T_C | 29893960 | 1.7 | 1.2 | 2.4 | 0.001871 | 0.20 | 0.25 | 0.19 | SNP |
| chr6_32610976_A_C | 32610976 | 0.3 | 0.2 | 0.6 | 0.001876 | 0.09 | 0.03 | 0.09 | SNP |
| chr6_32610696_G_A | 32610696 | 0.3 | 0.2 | 0.6 | 0.001889 | 0.08 | 0.03 | 0.09 | SNP |
| chr6_32607251_T_C | 32607251 | 0.6 | 0.4 | 0.8 | 0.001902 | 0.37 | 0.27 | 0.38 | SNP |
| chr6_32410941_C_T | 32410941 | 0.6 | 0.4 | 0.8 | 0.001922 | 0.50 | 0.39 | 0.51 | SNP |
| chr6_31914180_G_A | 31914180 | 0.3 | 0.2 | 0.6 | 0.001927 | 0.08 | 0.03 | 0.09 | SNP |
| chr6_32032421_C_T | 32032421 | 0.4 | 0.2 | 0.7 | 0.001939 | 0.11 | 0.04 | 0.12 | SNP |
| chr6_32410716_G_A | 32410716 | 1.6 | 1.2 | 2.2 | 0.001956 | 0.39 | 0.53 | 0.38 | SNP |
| chr6_32606912_A_G | 32606912 | 0.5 | 0.3 | 0.8 | 0.001971 | 0.16 | 0.08 | 0.16 | SNP |
| chr6_31506624_C_T | 31506624 | 1.8 | 1.2 | 2.6 | 0.002000 | 0.23 | 0.29 | 0.23 | SNP |
| chr6_32603405_C_T | 32603405 | 0.3 | 0.2 | 0.6 | 0.002021 | 0.08 | 0.03 | 0.09 | SNP |
| chr6_32606960_A_T | 32606960 | 0.3 | 0.2 | 0.6 | 0.002021 | 0.08 | 0.03 | 0.09 | SNP |
| chr6_32608967_A_G | 32608967 | 0.3 | 0.2 | 0.6 | 0.002025 | 0.09 | 0.03 | 0.10 | SNP |
| chr6_32611709_T_C | 32611709 | 0.3 | 0.2 | 0.6 | 0.002044 | 0.08 | 0.03 | 0.09 | SNP |
| chr6_31922254_C_A | 31922254 | 1.8 | 1.2 | 2.6 | 0.002059 | 0.35 | 0.36 | 0.34 | SNP |
| chr6_32606742_A_G | 32606742 | 0.3 | 0.2 | 0.6 | 0.002081 | 0.08 | 0.03 | 0.09 | SNP |
| chr6_32607270_A_G | 32607270 | 0.3 | 0.1 | 0.6 | 0.002111 | 0.08 | 0.03 | 0.09 | SNP |
| chr6_32341318_A_G | 32341318 | 1.7 | 1.2 | 2.3 | 0.002113 | 0.28 | 0.41 | 0.26 | SNP |
| chr6_32605861_A_G | 32605861 | 0.3 | 0.2 | 0.6 | 0.002151 | 0.08 | 0.03 | 0.09 | SNP |
| chr6_32609195_G_A | 32609195 | 0.3 | 0.2 | 0.6 | 0.002158 | 0.09 | 0.03 | 0.09 | SNP |
| chr6_32611215_C_T | 32611215 | 0.3 | 0.2 | 0.6 | 0.002158 | 0.09 | 0.03 | 0.09 | SNP |
| chr6_32606676_T_C | 32606676 | 0.3 | 0.2 | 0.6 | 0.002180 | 0.08 | 0.03 | 0.09 | SNP |
| chr6_32605950_C_T | 32605950 | 0.3 | 0.2 | 0.6 | 0.002190 | 0.08 | 0.03 | 0.09 | SNP |
| chr6_32604384_G_A | 32604384 | 0.3 | 0.2 | 0.6 | 0.002200 | 0.08 | 0.03 | 0.09 | SNP |
| chr6_32411035_C_A | 32411035 | 0.6 | 0.4 | 0.8 | 0.002222 | 0.50 | 0.39 | 0.51 | SNP |
| chr6_32441696_C_T | 32441696 | 0.3 | 0.1 | 0.6 | 0.002324 | 0.07 | 0.03 | 0.07 | SNP |
| chr6_32029205_G_A | 32029205 | 3.3 | 1.5 | 7.0 | 0.002380 | 0.12 | 0.12 | 0.12 | SNP |
| chr6_31248204_G_A | 31248204 | 0.6 | 0.4 | 0.8 | 0.002402 | 0.32 | 0.33 | 0.32 | SNP |
| chr6_30879636_C_T | 30879636 | 2.5 | 1.4 | 4.5 | 0.002448 | 0.11 | 0.13 | 0.11 | SNP |
| chr6_32607017_T_A | 32607017 | 0.3 | 0.2 | 0.7 | 0.002464 | 0.08 | 0.03 | 0.09 | SNP |
| chr6_31322487_G_A | 31322487 | 0.5 | 0.3 | 0.8 | 0.002505 | 0.10 | 0.09 | 0.11 | SNP |
| chr6_31745464_G_A | 31745464 | 3.2 | 1.5 | 6.8 | 0.002541 | 0.12 | 0.12 | 0.12 | SNP |
| chr6_32608425_C_A | 32608425 | 0.4 | 0.2 | 0.7 | 0.002646 | 0.08 | 0.03 | 0.09 | SNP |
| chr6_29853750_G_T | 29853750 | 1.8 | 1.2 | 2.6 | 0.002703 | 0.14 | 0.17 | 0.14 | SNP |
| chr6_29896112_C_T | 29896112 | 1.8 | 1.2 | 2.7 | 0.002738 | 0.16 | 0.19 | 0.15 | SNP |
| chr6_30855211_T_G | 30855211 | 2.4 | 1.3 | 4.1 | 0.002740 | 0.11 | 0.13 | 0.11 | SNP |
| chr6_31239855_G_C | 31239855 | 1.7 | 1.2 | 2.5 | 0.002810 | 0.36 | 0.38 | 0.35 | SNP |
| chr6_32609147_A_T | 32609147 | 0.5 | 0.4 | 0.8 | 0.002831 | 0.16 | 0.09 | 0.17 | SNP |
| chr6_31379115_G_A | 31379115 | 0.6 | 0.4 | 0.8 | 0.002927 | 0.21 | 0.19 | 0.22 | SNP |
| chr6_30857894_G_A | 30857894 | 2.3 | 1.3 | 4.1 | 0.002930 | 0.11 | 0.13 | 0.11 | SNP |
| chr6_30867527_G_A | 30867527 | 2.3 | 1.3 | 4.1 | 0.002930 | 0.11 | 0.13 | 0.11 | SNP |
| chr6_30879987_C_T | 30879987 | 2.4 | 1.3 | 4.2 | 0.002930 | 0.11 | 0.12 | 0.11 | SNP |
| chr6_31240871_G_C | 31240871 | 0.6 | 0.5 | 0.9 | 0.002956 | 0.35 | 0.35 | 0.35 | SNP |
| chr6_31864304_A_G | 31864304 | 1.7 | 1.2 | 2.4 | 0.002975 | 0.37 | 0.39 | 0.37 | SNP |
| chr6_32407709_C_A | 32407709 | 1.6 | 1.2 | 2.1 | 0.002981 | 0.36 | 0.50 | 0.34 | SNP |
| chr6_32611145_T_A | 32611145 | 1.6 | 1.2 | 2.2 | 0.003034 | 0.21 | 0.35 | 0.20 | SNP |
| chr6_32143991_G_C | 32143991 | 1.7 | 1.2 | 2.4 | 0.003072 | 0.16 | 0.20 | 0.15 | SNP |
| chr6_32943151_C_T | 32943151 | 0.7 | 0.5 | 0.9 | 0.003072 | 0.40 | 0.33 | 0.41 | SNP |
| chr6_32014828_G_A | 32014828 | 3.2 | 1.5 | 6.8 | 0.003076 | 0.12 | 0.13 | 0.12 | SNP |
| chr6_32635629_G_A | 32635629 | 1.8 | 1.2 | 2.6 | 0.003130 | 0.23 | 0.31 | 0.22 | SNP |
| xHLA_2field_DRB1_13_01 | 32546552 | 0.4 | 0.2 | 0.7 | 0.003155 | 0.08 | 0.03 | 0.09 | HLA (xHLA) |
| chr6_32052983_C_T | 32052983 | 0.4 | 0.2 | 0.7 | 0.003216 | 0.09 | 0.04 | 0.10 | SNP |
| chr6_31325684_C_A | 31325684 | 0.5 | 0.3 | 0.8 | 0.003222 | 0.10 | 0.09 | 0.11 | SNP |
| chr6_31325795_G_T | 31325795 | 0.5 | 0.3 | 0.8 | 0.003222 | 0.10 | 0.09 | 0.11 | SNP |
| chr6_32325562_G_A | 32325562 | 3.0 | 1.4 | 6.1 | 0.003228 | 0.12 | 0.12 | 0.12 | SNP |
| chr6_31325116_T_G | 31325116 | 1.8 | 1.2 | 2.6 | 0.003245 | 0.34 | 0.35 | 0.33 | SNP |
| chr6_31932177_C_T | 31932177 | 0.4 | 0.2 | 0.7 | 0.003246 | 0.12 | 0.05 | 0.12 | SNP |
| chr6_32411523_T_C | 32411523 | 1.6 | 1.2 | 2.1 | 0.003309 | 0.39 | 0.53 | 0.38 | SNP |
| chr6_32628556_C_T | 32628556 | 1.6 | 1.2 | 2.2 | 0.003336 | 0.20 | 0.31 | 0.19 | SNP |
| chr6_31323725_T_C | 31323725 | 0.5 | 0.3 | 0.8 | 0.003368 | 0.10 | 0.09 | 0.11 | SNP |
| chr6_32559444_C_T | 32559444 | 0.4 | 0.2 | 0.7 | 0.003381 | 0.09 | 0.03 | 0.10 | SNP |
| chr6_32036357_G_A | 32036357 | 0.4 | 0.2 | 0.7 | 0.003417 | 0.09 | 0.04 | 0.10 | SNP |
| chr6_31506744_C_A | 31506744 | 1.8 | 1.2 | 2.5 | 0.003420 | 0.23 | 0.28 | 0.23 | SNP |
| chr6_31323721_G_C | 31323721 | 0.5 | 0.3 | 0.8 | 0.003422 | 0.10 | 0.09 | 0.11 | SNP |
| chr6_32029183_C_T | 32029183 | 0.4 | 0.2 | 0.7 | 0.003431 | 0.09 | 0.04 | 0.10 | SNP |

| | | | | | | | | | |
|---|---|---|---|---|---|---|---|---|---|
| chr6_32005649_A_G | 32005649 | 0.5 | 0.3 | 0.8 | 0.003499 | 0.11 | 0.05 | 0.12 | SNP |
| chr6_31326292_A_G | 31326292 | 0.5 | 0.3 | 0.8 | 0.003511 | 0.10 | 0.09 | 0.11 | SNP |
| chr6_31370367_G_A | 31370367 | 0.5 | 0.3 | 0.8 | 0.003515 | 0.10 | 0.09 | 0.11 | SNP |
| chr6_29638434_A_G | 29638434 | 0.6 | 0.4 | 0.8 | 0.003620 | 0.15 | 0.09 | 0.15 | SNP |
| chr6_32610349_C_A | 32610349 | 1.6 | 1.2 | 2.1 | 0.003664 | 0.45 | 0.55 | 0.43 | SNP |
| chr6_32522698_G_T | 32522698 | 0.6 | 0.4 | 0.8 | 0.003693 | 0.41 | 0.33 | 0.42 | SNP |
| chr6_31508761_T_C | 31508761 | 1.7 | 1.2 | 2.5 | 0.003696 | 0.24 | 0.29 | 0.23 | SNP |
| chr6_32604660_C_T | 32604660 | 0.4 | 0.2 | 0.7 | 0.003711 | 0.09 | 0.03 | 0.10 | SNP |
| chr6_31370329_C_A | 31370329 | 0.5 | 0.3 | 0.8 | 0.003722 | 0.10 | 0.09 | 0.10 | SNP |
| chr6_32409305_A_G | 32409305 | 1.6 | 1.2 | 2.1 | 0.003822 | 0.39 | 0.53 | 0.38 | SNP |
| chr6_32559520_C_T | 32559520 | 1.6 | 1.2 | 2.1 | 0.003909 | 0.33 | 0.48 | 0.31 | SNP |
| chr6_31321268_T_G | 31321268 | 0.6 | 0.4 | 0.8 | 0.003922 | 0.20 | 0.15 | 0.20 | SNP |
| chr6_31540429_G_C | 31540429 | 1.5 | 1.1 | 2.0 | 0.003923 | 0.38 | 0.44 | 0.37 | SNP |
| chr6_29907740_A_G | 29907740 | 0.6 | 0.5 | 0.9 | 0.003949 | 0.47 | 0.45 | 0.47 | SNP |
| chr6_32441100_C_A | 32441100 | 0.6 | 0.4 | 0.8 | 0.003958 | 0.28 | 0.21 | 0.28 | SNP |
| chr6_31464992_G_A | 31464992 | 1.6 | 1.2 | 2.3 | 0.003982 | 0.29 | 0.32 | 0.28 | SNP |
| chr6_32603277_G_T | 32603277 | 0.3 | 0.1 | 0.6 | 0.004029 | 0.05 | 0.02 | 0.06 | SNP |
| chr6_31561211_C_T | 31561211 | 1.7 | 1.2 | 2.3 | 0.004047 | 0.26 | 0.31 | 0.26 | SNP |
| chr6_32610947_A_C | 32610947 | 0.3 | 0.1 | 0.6 | 0.004064 | 0.06 | 0.02 | 0.06 | SNP |
| chr6_31734345_G_T | 31734345 | 3.6 | 1.5 | 8.4 | 0.004080 | 0.12 | 0.13 | 0.12 | SNP |
| chr6_31320849_T_C | 31320849 | 0.5 | 0.3 | 0.8 | 0.004091 | 0.10 | 0.09 | 0.10 | SNP |
| chr6_32606682_C_G | 32606682 | 0.3 | 0.1 | 0.6 | 0.004123 | 0.05 | 0.02 | 0.06 | SNP |
| chr6_31601735_G_A | 31601735 | 0.3 | 0.1 | 0.6 | 0.004128 | 0.06 | 0.02 | 0.06 | SNP |
| chr6_32406807_G_A | 32406807 | 1.7 | 1.2 | 2.4 | 0.004157 | 0.37 | 0.55 | 0.36 | SNP |
| chr6_32606691_T_G | 32606691 | 0.3 | 0.1 | 0.6 | 0.004167 | 0.05 | 0.02 | 0.06 | SNP |
| chr6_30796545_T_C | 30796545 | 2.1 | 1.3 | 3.5 | 0.004215 | 0.12 | 0.14 | 0.12 | SNP |
| chr6_32609047_C_T | 32609047 | 0.3 | 0.1 | 0.6 | 0.004245 | 0.05 | 0.02 | 0.06 | SNP |
| chr6_31324955_C_T | 31324955 | 0.5 | 0.3 | 0.8 | 0.004281 | 0.10 | 0.09 | 0.10 | SNP |
| chr6_32187722_T_C | 32187722 | 1.5 | 1.1 | 2.0 | 0.004303 | 0.47 | 0.51 | 0.46 | SNP |
| chr6_31947086_G_A | 31947086 | 0.5 | 0.3 | 0.8 | 0.004307 | 0.11 | 0.05 | 0.12 | SNP |
| chr6_32709990_C_T | 32709990 | 0.6 | 0.4 | 0.9 | 0.004351 | 0.32 | 0.22 | 0.33 | SNP |
| chr6_32604166_A_G | 32604166 | 0.3 | 0.1 | 0.6 | 0.004369 | 0.06 | 0.02 | 0.06 | SNP |
| chr6_32441129_G_A | 32441129 | 0.3 | 0.1 | 0.6 | 0.004455 | 0.06 | 0.02 | 0.06 | SNP |
| chr6_32609009_T_G | 32609009 | 0.3 | 0.1 | 0.6 | 0.004475 | 0.06 | 0.02 | 0.06 | SNP |
| chr6_32710010_G_T | 32710010 | 0.6 | 0.4 | 0.9 | 0.004497 | 0.32 | 0.22 | 0.33 | SNP |
| chr6_32709964_T_C | 32709964 | 0.6 | 0.4 | 0.9 | 0.004555 | 0.32 | 0.22 | 0.33 | SNP |
| chr6_32559076_A_G | 32559076 | 0.6 | 0.4 | 0.9 | 0.004558 | 0.34 | 0.25 | 0.35 | SNP |
| chr6_32609425_C_G | 32609425 | 0.3 | 0.1 | 0.6 | 0.004607 | 0.06 | 0.02 | 0.06 | SNP |
| chr6_32603896_T_C | 32603896 | 0.3 | 0.1 | 0.6 | 0.004610 | 0.06 | 0.02 | 0.06 | SNP |
| chr6_32608350_G_A | 32608350 | 0.4 | 0.2 | 0.7 | 0.004621 | 0.10 | 0.03 | 0.10 | SNP |
| chr6_29856774_A_G | 29856774 | 1.7 | 1.2 | 2.5 | 0.004622 | 0.15 | 0.17 | 0.14 | SNP |
| chr6_31366295_C_A | 31366295 | 0.5 | 0.3 | 0.8 | 0.004639 | 0.11 | 0.10 | 0.11 | SNP |
| chr6_32013500_T_C | 32013500 | 0.4 | 0.2 | 0.7 | 0.004664 | 0.10 | 0.04 | 0.10 | SNP |
| chr6_32336299_A_G | 32336299 | 0.2 | 0.1 | 0.6 | 0.004676 | 0.05 | 0.01 | 0.05 | SNP |
| chr6_31464571_T_C | 31464571 | 1.6 | 1.2 | 2.2 | 0.004678 | 0.29 | 0.32 | 0.28 | SNP |
| chr6_32606376_T_C | 32606376 | 0.3 | 0.1 | 0.6 | 0.004706 | 0.06 | 0.02 | 0.06 | SNP |
| chr6_31325115_G_A | 31325115 | 1.7 | 1.2 | 2.5 | 0.004722 | 0.34 | 0.35 | 0.34 | SNP |
| chr6_31498497_A_C | 31498497 | 1.7 | 1.2 | 2.6 | 0.004755 | 0.23 | 0.28 | 0.22 | SNP |
| chr6_32441046_G_A | 32441046 | 0.3 | 0.1 | 0.6 | 0.004820 | 0.06 | 0.02 | 0.06 | SNP |
| chr6_32608435_T_C | 32608435 | 0.3 | 0.1 | 0.6 | 0.004870 | 0.06 | 0.02 | 0.06 | SNP |
| chr6_32797809_C_T | 32797809 | 1.6 | 1.1 | 2.2 | 0.004900 | 0.11 | 0.17 | 0.11 | SNP |
| chr6_32310863_T_C | 32310863 | 2.7 | 1.3 | 5.3 | 0.004931 | 0.12 | 0.12 | 0.12 | SNP |
| chr6_31624864_A_G | 31624864 | 3.1 | 1.4 | 6.9 | 0.004941 | 0.12 | 0.11 | 0.12 | SNP |
| chr6_32628606_C_A | 32628606 | 0.3 | 0.1 | 0.6 | 0.004965 | 0.05 | 0.02 | 0.06 | SNP |
| chr6_32439696_T_G | 32439696 | 0.3 | 0.1 | 0.6 | 0.004985 | 0.06 | 0.02 | 0.06 | SNP |
| chr6_31513864_T_A | 31513864 | 1.7 | 1.2 | 2.5 | 0.004991 | 0.23 | 0.28 | 0.23 | SNP |
| chr6_31326629_T_C | 31326629 | 1.7 | 1.2 | 2.4 | 0.005001 | 0.34 | 0.35 | 0.34 | SNP |
| chr6_31540556_T_C | 31540556 | 0.6 | 0.4 | 0.9 | 0.005017 | 0.23 | 0.19 | 0.24 | SNP |
| chr6_29693938_G_A | 29693938 | 0.6 | 0.4 | 0.8 | 0.005019 | 0.14 | 0.09 | 0.15 | SNP |
| chr6_31320164_G_A | 31320164 | 1.6 | 1.2 | 2.2 | 0.005049 | 0.23 | 0.28 | 0.22 | SNP |
| chr6_31324891_C_T | 31324891 | 0.5 | 0.3 | 0.8 | 0.005054 | 0.11 | 0.09 | 0.11 | SNP |
| chr6_31325792_G_A | 31325792 | 1.6 | 1.2 | 2.3 | 0.005141 | 0.35 | 0.37 | 0.35 | SNP |
| chr6_32080191_G_C | 32080191 | 3.1 | 1.4 | 6.8 | 0.005149 | 0.12 | 0.11 | 0.12 | SNP |
| chr6_32942302_A_G | 32942302 | 0.7 | 0.5 | 0.9 | 0.005158 | 0.41 | 0.33 | 0.41 | SNP |
| chr6_31634193_A_G | 31634193 | 1.5 | 1.1 | 2.0 | 0.005161 | 0.38 | 0.43 | 0.38 | SNP |
| chr6_32187605_G_A | 32187605 | 1.5 | 1.1 | 2.0 | 0.005167 | 0.50 | 0.54 | 0.49 | SNP |
| chr6_29691019_G_A | 29691019 | 0.7 | 0.5 | 0.9 | 0.005195 | 0.44 | 0.41 | 0.44 | SNP |
| chr6_31248169_C_T | 31248169 | 2.1 | 1.2 | 3.5 | 0.005243 | 0.14 | 0.15 | 0.14 | SNP |
| chr6_31463680_A_G | 31463680 | 1.6 | 1.1 | 2.2 | 0.005272 | 0.29 | 0.32 | 0.29 | SNP |
| chr6_32442703_G_A | 32442703 | 0.3 | 0.1 | 0.6 | 0.005280 | 0.05 | 0.02 | 0.06 | SNP |
| chr6_31927342_G_A | 31927342 | 1.7 | 1.2 | 2.4 | 0.005291 | 0.34 | 0.35 | 0.34 | SNP |
| chr6_31430143_G_C | 31430143 | 0.6 | 0.4 | 0.8 | 0.005346 | 0.18 | 0.14 | 0.19 | SNP |
| chr6_32164869_G_C | 32164869 | 0.7 | 0.5 | 0.9 | 0.005578 | 0.29 | 0.26 | 0.29 | SNP |
| chr6_30864279_C_T | 30864279 | 2.2 | 1.3 | 3.9 | 0.005638 | 0.11 | 0.12 | 0.11 | SNP |
| xHLA_2field_DRB1_13_02 | 32546552 | 0.3 | 0.1 | 0.7 | 0.005640 | 0.06 | 0.02 | 0.06 | HLA (xHLA) |
| chr6_31322703_C_T | 31322703 | 0.5 | 0.3 | 0.8 | 0.005680 | 0.11 | 0.10 | 0.11 | SNP |
| chr6_31325092_G_A | 31325092 | 1.7 | 1.2 | 2.5 | 0.005695 | 0.33 | 0.35 | 0.33 | SNP |
| chr6_32782387_G_A | 32782387 | 1.5 | 1.1 | 2.0 | 0.005743 | 0.21 | 0.25 | 0.21 | SNP |
| chr6_29901806_C_T | 29901806 | 1.6 | 1.1 | 2.2 | 0.005749 | 0.22 | 0.26 | 0.21 | SNP |
| chr6_31528326_C_G | 31528326 | 1.5 | 1.1 | 2.0 | 0.005806 | 0.38 | 0.44 | 0.38 | SNP |
| chr6_32153409_A_C | 32153409 | 1.5 | 1.1 | 2.1 | 0.005849 | 0.39 | 0.50 | 0.38 | SNP |
| chr6_31324595_C_G | 31324595 | 0.6 | 0.4 | 0.9 | 0.005923 | 0.31 | 0.25 | 0.31 | SNP |
| chr6_32629545_T_C | 32629545 | 0.3 | 0.1 | 0.7 | 0.005927 | 0.05 | 0.02 | 0.06 | SNP |
| chr6_32340068_T_C | 32340068 | 0.7 | 0.5 | 0.9 | 0.005932 | 0.50 | 0.39 | 0.51 | SNP |
| chr6_32628843_C_T | 32628843 | 0.3 | 0.1 | 0.7 | 0.005988 | 0.05 | 0.02 | 0.06 | SNP |
| chr6_31324348_C_T | 31324348 | 0.5 | 0.3 | 0.8 | 0.006022 | 0.11 | 0.09 | 0.11 | SNP |
| chr6_29892137_A_G | 29892137 | 1.5 | 1.1 | 2.0 | 0.006023 | 0.23 | 0.28 | 0.23 | SNP |
| chr6_32056580_A_C | 32056580 | 0.4 | 0.2 | 0.8 | 0.006146 | 0.09 | 0.04 | 0.10 | SNP |
| chr6_32633273_T_G | 32633273 | 0.6 | 0.5 | 0.9 | 0.006197 | 0.36 | 0.27 | 0.37 | SNP |
| chr6_32551638_C_T | 32551638 | 0.4 | 0.2 | 0.7 | 0.006201 | 0.07 | 0.03 | 0.08 | SNP |
| chr6_32303692_T_A | 32303692 | 2.6 | 1.3 | 5.2 | 0.006329 | 0.12 | 0.12 | 0.12 | SNP |
| chr6_32724857_C_T | 32724857 | 1.6 | 1.1 | 2.3 | 0.006340 | 0.09 | 0.14 | 0.09 | SNP |
| chr6_32169574_T_C | 32169574 | 0.7 | 0.5 | 0.9 | 0.006368 | 0.30 | 0.26 | 0.30 | SNP |
| chr6_32270222_C_A | 32270222 | 0.3 | 0.1 | 0.6 | 0.006368 | 0.05 | 0.02 | 0.05 | SNP |
| chr6_29858253_G_A | 29858253 | 1.7 | 1.2 | 2.5 | 0.006458 | 0.14 | 0.17 | 0.14 | SNP |
| chr6_31629995_G_A | 31629995 | 0.3 | 0.1 | 0.7 | 0.006529 | 0.06 | 0.02 | 0.07 | SNP |
| chr6_29896108_A_T | 29896108 | 1.7 | 1.1 | 2.5 | 0.006630 | 0.16 | 0.18 | 0.15 | SNP |
| chr6_31473844_C_T | 31473844 | 1.6 | 1.1 | 2.2 | 0.006730 | 0.29 | 0.32 | 0.29 | SNP |
| chr6_32498208_G_T | 32498208 | 1.5 | 1.1 | 2.1 | 0.006844 | 0.30 | 0.43 | 0.28 | SNP |
| chr6_32409242_C_A | 32409242 | 0.7 | 0.5 | 0.9 | 0.006866 | 0.43 | 0.31 | 0.45 | SNP |
| chr6_29896713_G_A | 29896713 | 1.5 | 1.1 | 2.1 | 0.006922 | 0.23 | 0.27 | 0.23 | SNP |
| chr6_32035427_T_C | 32035427 | 0.4 | 0.2 | 0.8 | 0.007011 | 0.09 | 0.04 | 0.10 | SNP |
| chr6_31236115_T_C | 31236115 | 1.5 | 1.1 | 2.0 | 0.007055 | 0.40 | 0.43 | 0.40 | SNP |
| chr6_32556677_C_A | 32556677 | 1.7 | 1.2 | 2.6 | 0.007158 | 0.10 | 0.17 | 0.10 | SNP |
| chr6_32303160_G_T | 32303160 | 0.3 | 0.1 | 0.6 | 0.007170 | 0.05 | 0.02 | 0.05 | SNP |
| chr6_31463621_G_A | 31463621 | 1.6 | 1.1 | 2.2 | 0.007209 | 0.29 | 0.33 | 0.29 | SNP |
| chr6_31238851_C_T | 31238851 | 1.8 | 1.2 | 2.7 | 0.007239 | 0.31 | 0.32 | 0.31 | SNP |
| chr6_32261940_G_C | 32261940 | 0.3 | 0.1 | 0.6 | 0.007242 | 0.05 | 0.02 | 0.05 | SNP |
| chr6_29893922_A_T | 29893922 | 1.6 | 1.1 | 2.2 | 0.007249 | 0.20 | 0.24 | 0.20 | SNP |
| chr6_31379752_C_T | 31379752 | 1.8 | 1.2 | 2.8 | 0.007318 | 0.07 | 0.10 | 0.07 | SNP |
| chr6_32261252_T_G | 32261252 | 2.6 | 1.3 | 5.1 | 0.007322 | 0.12 | 0.12 | 0.12 | SNP |
| chr6_32290208_C_T | 32290208 | 2.6 | 1.3 | 5.1 | 0.007322 | 0.12 | 0.12 | 0.12 | SNP |
| chr6_32786833_C_G | 32786833 | 0.7 | 0.5 | 0.9 | 0.007547 | 0.38 | 0.31 | 0.39 | SNP |
| xHLA_2field_DQB1_02_01 | 32627244 | 2.2 | 1.2 | 4.0 | 0.007565 | 0.13 | 0.14 | 0.12 | HLA (xHLA) |
| chr6_31540141_C_A | 31540141 | 1.5 | 1.1 | 2.0 | 0.007619 | 0.38 | 0.44 | 0.38 | SNP |
| chr6_29893403_C_G | 29893403 | 1.7 | 1.1 | 2.4 | 0.007619 | 0.15 | 0.17 | 0.15 | SNP |
| chr6_32012755_G_A | 32012755 | 0.4 | 0.2 | 0.7 | 0.007703 | 0.08 | 0.03 | 0.09 | SNP |
| chr6_32732585_C_T | 32732585 | 1.6 | 1.1 | 2.3 | 0.007742 | 0.10 | 0.14 | 0.09 | SNP |
| chr6_31502767_T_C | 31502767 | 1.7 | 1.1 | 2.5 | 0.007775 | 0.23 | 0.28 | 0.22 | SNP |
| chr6_31730568_A_G | 31730568 | 0.4 | 0.2 | 0.8 | 0.007832 | 0.07 | 0.03 | 0.08 | SNP |
| chr6_31320176_C_T | 31320176 | 0.6 | 0.4 | 0.9 | 0.007840 | 0.18 | 0.15 | 0.19 | SNP |
| chr6_32785714_T_C | 32785714 | 1.5 | 1.1 | 2.0 | 0.007841 | 0.21 | 0.25 | 0.21 | SNP |
| chr6_32011316_G_T | 32011316 | 0.3 | 0.1 | 0.6 | 0.007877 | 0.07 | 0.02 | 0.07 | SNP |
| chr6_31324448_G_A | 31324448 | 0.5 | 0.3 | 0.8 | 0.007985 | 0.11 | 0.10 | 0.11 | SNP |
| chr6_32627700_A_G | 32627700 | 0.6 | 0.4 | 0.9 | 0.008007 | 0.19 | 0.11 | 0.20 | SNP |
| chr6_33036950_C_T | 33036950 | 0.3 | 0.1 | 0.7 | 0.008039 | 0.05 | 0.02 | 0.06 | SNP |
| chr6_32029849_G_A | 32029849 | 0.4 | 0.2 | 0.8 | 0.008052 | 0.10 | 0.04 | 0.10 | SNP |
| chr6_31947460_T_G | 31947460 | 1.7 | 1.2 | 2.6 | 0.008160 | 0.34 | 0.34 | 0.34 | SNP |
| chr6_32123639_G_A | 32123639 | 1.5 | 1.1 | 2.1 | 0.008191 | 0.16 | 0.20 | 0.16 | SNP |
| chr6_31237255_A_G | 31237255 | 1.5 | 1.1 | 2.1 | 0.008282 | 0.40 | 0.41 | 0.40 | SNP |
| chr6_32007625_C_T | 32007625 | 0.5 | 0.3 | 0.8 | 0.008282 | 0.10 | 0.05 | 0.10 | SNP |
| chr6_32011317_T_C | 32011317 | 0.3 | 0.1 | 0.6 | 0.008430 | 0.07 | 0.02 | 0.07 | SNP |
| chr6_31321327_A_G | 31321327 | 0.7 | 0.5 | 0.9 | 0.008491 | 0.24 | 0.19 | 0.24 | SNP |
| chr6_32631029_T_C | 32631029 | 0.3 | 0.1 | 0.7 | 0.008573 | 0.06 | 0.02 | 0.07 | SNP |
| chr6_31633165_C_A | 31633165 | 1.5 | 1.1 | 2.0 | 0.008622 | 0.18 | 0.23 | 0.18 | SNP |
| chr6_31474688_G_T | 31474688 | 1.6 | 1.1 | 2.2 | 0.008643 | 0.29 | 0.32 | 0.29 | SNP |
| chr6_33590061_C_T | 33590061 | 0.4 | 0.2 | 0.8 | 0.008853 | 0.06 | 0.03 | 0.06 | SNP |
| chr6_32373801_T_G | 32373801 | 1.5 | 1.1 | 2.0 | 0.008893 | 0.38 | 0.50 | 0.36 | SNP |
| chr6_31238762_C_T | 31238762 | 1.6 | 1.1 | 2.3 | 0.008951 | 0.36 | 0.37 | 0.36 | SNP |
| chr6_29896636_C_T | 29896636 | 1.5 | 1.1 | 2.0 | 0.009315 | 0.23 | 0.27 | 0.22 | SNP |
| chr6_31473546_G_A | 31473546 | 1.6 | 1.1 | 2.2 | 0.009424 | 0.28 | 0.32 | 0.28 | SNP |
| chr6_32628272_A_G | 32628272 | 0.6 | 0.4 | 0.9 | 0.009580 | 0.13 | 0.10 | 0.13 | SNP |
| chr6_31860337_C_T | 31860337 | 1.6 | 1.1 | 2.3 | 0.009585 | 0.37 | 0.38 | 0.37 | SNP |
| chr6_31935311_C_T | 31935311 | 0.5 | 0.3 | 0.8 | 0.009674 | 0.11 | 0.06 | 0.12 | SNP |
| chr6_32411646_G_T | 32411646 | 0.7 | 0.5 | 0.9 | 0.009818 | 0.47 | 0.37 | 0.48 | SNP |
| chr6_31602489_T_C | 31602489 | 1.5 | 1.1 | 2.1 | 0.009861 | 0.18 | 0.23 | 0.18 | SNP |
| chr6_31466547_C_T | 31466547 | 1.6 | 1.1 | 2.2 | 0.009911 | 0.28 | 0.32 | 0.28 | SNP |
| chr6_31593265_G_T | 31593265 | 0.4 | 0.2 | 0.7 | 0.009956 | 0.06 | 0.02 | 0.06 | SNP |

| | | | | | | | | | |
|---|---|---|---|---|---|---|---|---|---|
| chr6_29893982_C_T | 29893982 | 1.6 | 1.1 | 2.3 | 0.010033 | 0.19 | 0.22 | 0.19 | SNP |
| chr6_33036717_C_G | 33036717 | 0.6 | 0.4 | 0.9 | 0.010048 | 0.14 | 0.08 | 0.15 | SNP |
| chr6_32339498_C_G | 32339498 | 0.3 | 0.1 | 0.7 | 0.010052 | 0.05 | 0.02 | 0.05 | SNP |
| chr6_29858840_A_C | 29858840 | 1.7 | 1.1 | 2.4 | 0.010096 | 0.15 | 0.17 | 0.14 | SNP |
| chr6_32412539_G_A | 32412539 | 0.7 | 0.5 | 0.9 | 0.010172 | 0.47 | 0.37 | 0.48 | SNP |
| chr6_30851909_C_A | 30851909 | 2.3 | 1.2 | 4.3 | 0.010176 | 0.12 | 0.14 | 0.12 | SNP |
| chr6_33036708_C_T | 33036708 | 0.6 | 0.4 | 0.9 | 0.010255 | 0.14 | 0.08 | 0.15 | SNP |
| chr6_33036709_A_G | 33036709 | 0.6 | 0.4 | 0.9 | 0.010255 | 0.14 | 0.08 | 0.15 | SNP |
| chr6_32796793_G_A | 32796793 | 1.6 | 1.1 | 2.4 | 0.010277 | 0.09 | 0.15 | 0.09 | SNP |
| chr6_32409781_C_T | 32409781 | 1.5 | 1.1 | 2.0 | 0.010284 | 0.39 | 0.52 | 0.38 | SNP |
| chr6_32410215_T_C | 32410215 | 0.6 | 0.4 | 0.9 | 0.010357 | 0.22 | 0.15 | 0.23 | SNP |
| chr6_32178773_T_C | 32178773 | 0.7 | 0.5 | 0.9 | 0.010362 | 0.48 | 0.42 | 0.48 | SNP |
| chr6_31616366_G_A | 31616366 | 1.4 | 1.1 | 1.9 | 0.010400 | 0.38 | 0.41 | 0.38 | SNP |
| chr6_32339348_T_C | 32339348 | 0.6 | 0.5 | 0.9 | 0.010524 | 0.34 | 0.27 | 0.35 | SNP |
| chr6_32303848_G_A | 32303848 | 1.6 | 1.1 | 2.2 | 0.010601 | 0.24 | 0.35 | 0.23 | SNP |
| chr6_32410210_T_C | 32410210 | 0.6 | 0.4 | 0.9 | 0.010617 | 0.22 | 0.15 | 0.23 | SNP |
| chr6_32442296_C_T | 32442296 | 0.6 | 0.4 | 0.9 | 0.010619 | 0.29 | 0.21 | 0.30 | SNP |
| chr6_32725271_G_A | 32725271 | 0.6 | 0.4 | 0.9 | 0.010660 | 0.21 | 0.14 | 0.22 | SNP |
| chr6_32796751_T_C | 32796751 | 0.6 | 0.4 | 0.9 | 0.010697 | 0.23 | 0.14 | 0.24 | SNP |
| chr6_31607952_G_A | 31607952 | 0.4 | 0.2 | 0.8 | 0.010751 | 0.06 | 0.02 | 0.06 | SNP |
| chr6_32498205_G_T | 32498205 | 1.5 | 1.1 | 2.0 | 0.010891 | 0.30 | 0.43 | 0.28 | SNP |
| chr6_31934372_C_T | 31934372 | 0.5 | 0.3 | 0.8 | 0.010942 | 0.12 | 0.06 | 0.12 | SNP |
| chr6_32633519_A_G | 32633519 | 0.6 | 0.4 | 0.9 | 0.010943 | 0.15 | 0.12 | 0.15 | SNP |
| chr6_32014575_A_G | 32014575 | 0.7 | 0.5 | 0.9 | 0.010958 | 0.48 | 0.48 | 0.48 | SNP |
| chr6_31627449_C_G | 31627449 | 1.5 | 1.1 | 2.0 | 0.010979 | 0.18 | 0.23 | 0.18 | SNP |
| chr6_32035603_C_T | 32035603 | 0.4 | 0.2 | 0.8 | 0.011296 | 0.09 | 0.04 | 0.10 | SNP |
| chr6_31996524_C_A | 31996524 | 0.4 | 0.2 | 0.8 | 0.011320 | 0.07 | 0.03 | 0.08 | SNP |
| chr6_31473286_T_C | 31473286 | 1.6 | 1.1 | 2.2 | 0.011321 | 0.28 | 0.31 | 0.28 | SNP |
| chr6_32412480_A_G | 32412480 | 0.7 | 0.5 | 0.9 | 0.011461 | 0.47 | 0.37 | 0.48 | SNP |
| chr6_31542308_T_C | 31542308 | 0.6 | 0.5 | 0.9 | 0.011480 | 0.19 | 0.17 | 0.19 | SNP |
| chr6_32409060_G_A | 32409060 | 0.6 | 0.4 | 0.9 | 0.011493 | 0.22 | 0.16 | 0.23 | SNP |
| chr6_32522703_T_G | 32522703 | 0.6 | 0.4 | 0.9 | 0.011652 | 0.40 | 0.33 | 0.41 | SNP |
| chr6_32408497_A_G | 32408497 | 2.5 | 1.2 | 4.9 | 0.011767 | 0.12 | 0.12 | 0.12 | SNP |
| chr6_32375745_G_A | 32375745 | 1.5 | 1.1 | 2.1 | 0.011789 | 0.48 | 0.58 | 0.47 | SNP |
| chr6_32376618_C_T | 32376618 | 1.5 | 1.1 | 2.1 | 0.011789 | 0.48 | 0.58 | 0.47 | SNP |
| chr6_32498206_A_T | 32498206 | 1.5 | 1.1 | 2.0 | 0.011970 | 0.30 | 0.43 | 0.28 | SNP |
| chr6_30229267_G_C | 30229267 | 2.0 | 1.2 | 3.4 | 0.012015 | 0.10 | 0.11 | 0.10 | SNP |
| chr6_32025870_C_T | 32025870 | 0.4 | 0.2 | 0.8 | 0.012305 | 0.09 | 0.04 | 0.10 | SNP |
| chr6_29895626_T_A | 29895626 | 1.6 | 1.1 | 2.4 | 0.012496 | 0.15 | 0.17 | 0.15 | SNP |
| chr6_32604968_A_C | 32604968 | 0.6 | 0.4 | 0.9 | 0.012615 | 0.25 | 0.18 | 0.26 | SNP |
| chr6_32628098_G_C | 32628098 | 0.3 | 0.1 | 0.7 | 0.012715 | 0.05 | 0.02 | 0.05 | SNP |
| chr6_32340871_C_T | 32340871 | 2.5 | 1.2 | 4.9 | 0.012763 | 0.12 | 0.12 | 0.12 | SNP |
| chr6_32409787_T_C | 32409787 | 1.5 | 1.1 | 2.0 | 0.012816 | 0.39 | 0.52 | 0.38 | SNP |
| chr6_32948426_C_T | 32948426 | 0.5 | 0.3 | 0.9 | 0.012847 | 0.09 | 0.06 | 0.10 | SNP |
| chr6_29691857_C_G | 29691857 | 0.7 | 0.5 | 0.9 | 0.012888 | 0.46 | 0.43 | 0.46 | SNP |
| xHLA_2field_C_03_04 | 31236526 | 0.6 | 0.4 | 0.9 | 0.013371 | 0.15 | 0.15 | 0.15 | HLA (xHLA) |
| chr6_32011369_G_T | 32011369 | 0.4 | 0.2 | 0.8 | 0.013430 | 0.09 | 0.04 | 0.10 | SNP |
| chr6_31929014_C_A | 31929014 | 1.6 | 1.1 | 2.4 | 0.013596 | 0.34 | 0.34 | 0.34 | SNP |
| chr6_32410691_G_A | 32410691 | 0.6 | 0.4 | 0.9 | 0.013679 | 0.22 | 0.16 | 0.23 | SNP |
| chr6_32709204_G_C | 32709204 | 0.6 | 0.4 | 0.9 | 0.013727 | 0.21 | 0.14 | 0.22 | SNP |
| chr6_29924889_C_A | 29924889 | 0.7 | 0.5 | 0.9 | 0.014018 | 0.40 | 0.40 | 0.40 | SNP |
| chr6_31349922_C_T | 31349922 | 0.6 | 0.4 | 0.9 | 0.014098 | 0.12 | 0.11 | 0.12 | SNP |
| chr6_29901865_G_C | 29901865 | 1.4 | 1.1 | 1.9 | 0.014103 | 0.28 | 0.31 | 0.28 | SNP |
| chr6_32373698_G_T | 32373698 | 1.5 | 1.1 | 2.0 | 0.014302 | 0.38 | 0.50 | 0.36 | SNP |
| chr6_32412809_T_A | 32412809 | 0.6 | 0.4 | 0.9 | 0.014616 | 0.22 | 0.16 | 0.23 | SNP |
| chr6_32812528_C_T | 32812528 | 1.4 | 1.1 | 1.9 | 0.014622 | 0.16 | 0.20 | 0.16 | SNP |
| chr6_32164587_C_T | 32164587 | 0.4 | 0.1 | 0.7 | 0.014657 | 0.04 | 0.02 | 0.05 | SNP |
| chr6_32412592_G_A | 32412592 | 0.6 | 0.4 | 0.9 | 0.014718 | 0.22 | 0.16 | 0.23 | SNP |
| chr6_32412398_T_C | 32412398 | 0.7 | 0.5 | 0.9 | 0.014887 | 0.47 | 0.38 | 0.48 | SNP |
| chr6_32707585_G_A | 32707585 | 0.6 | 0.4 | 0.9 | 0.014911 | 0.21 | 0.14 | 0.22 | SNP |
| chr6_31607050_T_C | 31607050 | 1.8 | 1.1 | 2.9 | 0.015074 | 0.19 | 0.21 | 0.19 | SNP |
| chr6_31657701_C_A | 31657701 | 1.5 | 1.1 | 2.2 | 0.015108 | 0.15 | 0.21 | 0.14 | SNP |
| chr6_31844684_A_G | 31844684 | 0.4 | 0.2 | 0.8 | 0.015162 | 0.07 | 0.03 | 0.07 | SNP |
| chr6_32411307_C_T | 32411307 | 0.6 | 0.4 | 0.9 | 0.015170 | 0.22 | 0.16 | 0.23 | SNP |
| chr6_32412566_T_A | 32412566 | 0.6 | 0.4 | 0.9 | 0.015170 | 0.22 | 0.16 | 0.23 | SNP |
| chr6_32192436_A_G | 32192436 | 0.5 | 0.2 | 0.8 | 0.015187 | 0.07 | 0.03 | 0.07 | SNP |
| chr6_31498737_G_A | 31498737 | 0.6 | 0.5 | 0.9 | 0.015255 | 0.16 | 0.15 | 0.16 | SNP |
| chr6_29892732_G_A | 29892732 | 1.5 | 1.1 | 2.0 | 0.015280 | 0.20 | 0.24 | 0.20 | SNP |
| chr6_32550591_G_A | 32550591 | 0.4 | 0.2 | 0.8 | 0.015354 | 0.06 | 0.03 | 0.06 | SNP |
| chr6_32376882_T_G | 32376882 | 1.5 | 1.1 | 2.0 | 0.015539 | 0.48 | 0.57 | 0.47 | SNP |
| chr6_31539768_G_A | 31539768 | 1.4 | 1.1 | 1.9 | 0.015562 | 0.35 | 0.40 | 0.34 | SNP |
| chr6_32822186_T_G | 32822186 | 0.4 | 0.2 | 0.8 | 0.015701 | 0.05 | 0.03 | 0.06 | SNP |
| chr6_32184024_C_T | 32184024 | 0.6 | 0.4 | 0.9 | 0.015792 | 0.14 | 0.11 | 0.14 | SNP |
| chr6_32192617_T_C | 32192617 | 0.4 | 0.2 | 0.8 | 0.015815 | 0.05 | 0.02 | 0.06 | SNP |
| chr6_31601513_A_G | 31601513 | 1.4 | 1.1 | 1.9 | 0.015916 | 0.39 | 0.41 | 0.38 | SNP |
| chr6_31506801_G_A | 31506801 | 0.7 | 0.5 | 0.9 | 0.015920 | 0.16 | 0.15 | 0.16 | SNP |
| chr6_29912188_T_C | 29912188 | 0.7 | 0.5 | 0.9 | 0.015978 | 0.43 | 0.42 | 0.43 | SNP |
| chr6_32604551_C_T | 32604551 | 2.2 | 1.1 | 4.0 | 0.016171 | 0.13 | 0.13 | 0.13 | SNP |
| chr6_32943345_T_C | 32943345 | 0.7 | 0.6 | 0.9 | 0.016175 | 0.46 | 0.39 | 0.46 | SNP |
| chr6_32973281_C_T | 32973281 | 0.7 | 0.6 | 0.9 | 0.016295 | 0.46 | 0.39 | 0.47 | SNP |
| chr6_31324953_T_C | 31324953 | 1.6 | 1.1 | 2.3 | 0.016349 | 0.37 | 0.39 | 0.37 | SNP |
| chr6_31465915_C_G | 31465915 | 1.5 | 1.1 | 2.1 | 0.016358 | 0.29 | 0.31 | 0.28 | SNP |
| chr6_29910378_C_T | 29910378 | 0.7 | 0.5 | 0.9 | 0.016482 | 0.35 | 0.34 | 0.35 | SNP |
| chr6_32189921_A_G | 32189921 | 2.1 | 1.1 | 3.6 | 0.016550 | 0.13 | 0.13 | 0.13 | SNP |
| chr6_32634893_G_A | 32634893 | 0.6 | 0.4 | 0.9 | 0.016586 | 0.14 | 0.11 | 0.14 | SNP |
| chr6_32559516_T_C | 32559516 | 0.7 | 0.5 | 0.9 | 0.016659 | 0.44 | 0.35 | 0.44 | SNP |
| chr6_31321883_G_A | 31321883 | 0.6 | 0.4 | 0.9 | 0.016660 | 0.14 | 0.13 | 0.14 | SNP |
| chr6_31477461_T_C | 31477461 | 1.5 | 1.1 | 2.1 | 0.016904 | 0.28 | 0.31 | 0.28 | SNP |
| chr6_30878857_G_A | 30878857 | 0.5 | 0.3 | 0.9 | 0.016906 | 0.06 | 0.06 | 0.06 | SNP |
| chr6_33037560_G_A | 33037560 | 0.6 | 0.3 | 0.9 | 0.017036 | 0.12 | 0.06 | 0.12 | SNP |
| chr6_32376932_C_T | 32376932 | 1.5 | 1.1 | 2.0 | 0.017080 | 0.48 | 0.57 | 0.47 | SNP |
| chr6_33036401_C_T | 33036401 | 0.6 | 0.4 | 0.9 | 0.017225 | 0.15 | 0.09 | 0.15 | SNP |
| chr6_31930462_G_A | 31930462 | 0.6 | 0.4 | 0.9 | 0.017454 | 0.14 | 0.09 | 0.14 | SNP |
| chr6_31837277_A_C | 31837277 | 1.5 | 1.1 | 2.0 | 0.017540 | 0.49 | 0.49 | 0.49 | SNP |
| chr6_29892107_C_T | 29892107 | 1.5 | 1.1 | 2.0 | 0.017712 | 0.21 | 0.24 | 0.20 | SNP |
| chr6_31675765_G_A | 31675765 | 1.5 | 1.1 | 2.1 | 0.018018 | 0.19 | 0.24 | 0.19 | SNP |
| chr6_31321425_G_A | 31321425 | 0.7 | 0.5 | 0.9 | 0.018040 | 0.21 | 0.18 | 0.21 | SNP |
| chr6_31320241_G_A | 31320241 | 1.5 | 1.1 | 2.1 | 0.018045 | 0.21 | 0.25 | 0.21 | SNP |
| chr6_31239827_T_C | 31239827 | 1.6 | 1.1 | 2.3 | 0.018078 | 0.37 | 0.38 | 0.37 | SNP |
| chr6_29912595_G_A | 29912595 | 0.7 | 0.6 | 0.9 | 0.018124 | 0.41 | 0.40 | 0.41 | SNP |
| chr6_31245080_G_A | 31245080 | 1.9 | 1.1 | 3.2 | 0.018316 | 0.04 | 0.06 | 0.04 | SNP |
| chr6_32370835_T_C | 32370835 | 1.4 | 1.1 | 2.0 | 0.018546 | 0.37 | 0.49 | 0.36 | SNP |
| chr6_31239869_T_C | 31239869 | 0.7 | 0.5 | 0.9 | 0.018724 | 0.30 | 0.24 | 0.30 | SNP |
| chr6_32559400_A_T | 32559400 | 1.5 | 1.1 | 2.0 | 0.018801 | 0.50 | 0.57 | 0.49 | SNP |
| chr6_32814244_A_T | 32814244 | 0.6 | 0.4 | 0.9 | 0.018846 | 0.11 | 0.06 | 0.11 | SNP |
| chr6_33045698_G_A | 33045698 | 0.3 | 0.1 | 0.8 | 0.018906 | 0.04 | 0.02 | 0.05 | SNP |
| chr6_32306480_A_T | 32306480 | 0.4 | 0.2 | 0.8 | 0.018981 | 0.07 | 0.03 | 0.07 | SNP |
| chr6_32813279_C_A | 32813279 | 0.6 | 0.4 | 0.9 | 0.019003 | 0.11 | 0.06 | 0.11 | SNP |
| chr6_31633689_C_T | 31633689 | 1.5 | 1.1 | 2.1 | 0.019196 | 0.18 | 0.22 | 0.18 | SNP |
| chr6_33382288_A_G | 33382288 | 0.5 | 0.3 | 0.9 | 0.019294 | 0.09 | 0.06 | 0.09 | SNP |
| chr6_31509432_A_G | 31509432 | 1.5 | 1.1 | 2.0 | 0.019303 | 0.31 | 0.35 | 0.31 | SNP |
| chr6_32501211_G_A | 32501211 | 1.6 | 1.1 | 2.5 | 0.019326 | 0.19 | 0.23 | 0.19 | SNP |
| chr6_32797876_A_G | 32797876 | 0.7 | 0.5 | 0.9 | 0.019356 | 0.41 | 0.31 | 0.42 | SNP |
| chr6_32408842_A_G | 32408842 | 0.6 | 0.4 | 0.9 | 0.019368 | 0.22 | 0.16 | 0.23 | SNP |
| chr6_29908491_G_A | 29908491 | 1.4 | 1.1 | 1.9 | 0.019498 | 0.18 | 0.23 | 0.18 | SNP |
| chr6_29909797_C_T | 29909797 | 1.5 | 1.1 | 2.1 | 0.019562 | 0.18 | 0.20 | 0.18 | SNP |
| chr6_33033313_G_T | 33033313 | 0.6 | 0.3 | 0.9 | 0.019718 | 0.12 | 0.07 | 0.12 | SNP |
| chr6_32411376_T_G | 32411376 | 0.6 | 0.4 | 0.9 | 0.019743 | 0.22 | 0.16 | 0.23 | SNP |
| chr6_32297209_G_A | 32297209 | 0.5 | 0.2 | 0.8 | 0.019851 | 0.07 | 0.03 | 0.07 | SNP |
| chr6_29896773_C_T | 29896773 | 1.4 | 1.1 | 2.0 | 0.019882 | 0.23 | 0.25 | 0.23 | SNP |
| chr6_32339925_T_A | 32339925 | 1.4 | 1.0 | 1.8 | 0.019949 | 0.33 | 0.36 | 0.33 | SNP |
| chr6_31464325_T_C | 31464325 | 0.7 | 0.5 | 0.9 | 0.020045 | 0.30 | 0.24 | 0.30 | SNP |
| chr6_32441396_A_G | 32441396 | 0.7 | 0.5 | 0.9 | 0.020129 | 0.37 | 0.29 | 0.38 | SNP |
| chr6_29895469_G_A | 29895469 | 1.6 | 1.1 | 2.4 | 0.020169 | 0.14 | 0.16 | 0.14 | SNP |
| chr6_32406887_C_T | 32406887 | 0.7 | 0.5 | 0.9 | 0.020249 | 0.32 | 0.21 | 0.33 | SNP |
| chr6_32406611_C_A | 32406611 | 0.6 | 0.4 | 0.9 | 0.020443 | 0.22 | 0.16 | 0.23 | SNP |
| chr6_29892229_T_G | 29892229 | 1.4 | 1.1 | 1.9 | 0.020525 | 0.25 | 0.28 | 0.25 | SNP |
| chr6_32192107_C_G | 32192107 | 0.6 | 0.4 | 0.9 | 0.020775 | 0.14 | 0.10 | 0.14 | SNP |
| chr6_32145205_C_T | 32145205 | 1.7 | 1.1 | 2.7 | 0.020792 | 0.18 | 0.20 | 0.17 | SNP |
| chr6_32376788_C_T | 32376788 | 1.4 | 1.1 | 2.0 | 0.020806 | 0.45 | 0.54 | 0.44 | SNP |
| chr6_29913374_C_G | 29913374 | 1.6 | 1.1 | 2.5 | 0.020912 | 0.14 | 0.15 | 0.13 | SNP |
| chr6_32439753_T_A | 32439753 | 0.6 | 0.4 | 0.9 | 0.021152 | 0.31 | 0.23 | 0.32 | SNP |
| chr6_31380449_C_T | 31380449 | 1.5 | 1.1 | 2.1 | 0.021161 | 0.34 | 0.35 | 0.34 | SNP |
| chr6_32725832_A_G | 32725832 | 0.6 | 0.4 | 0.9 | 0.021341 | 0.20 | 0.14 | 0.21 | SNP |
| chr6_31611840_C_A | 31611840 | 1.5 | 1.1 | 2.0 | 0.021384 | 0.18 | 0.22 | 0.17 | SNP |
| chr6_33037878_G_A | 33037878 | 0.6 | 0.4 | 0.9 | 0.021398 | 0.14 | 0.08 | 0.14 | SNP |
| chr6_31325090_T_C | 31325090 | 1.5 | 1.1 | 2.1 | 0.021437 | 0.36 | 0.37 | 0.36 | SNP |
| chr6_31237605_G_A | 31237605 | 0.7 | 0.5 | 0.9 | 0.021508 | 0.20 | 0.16 | 0.20 | SNP |
| chr6_33036394_G_A | 33036394 | 0.6 | 0.4 | 0.9 | 0.021518 | 0.14 | 0.09 | 0.15 | SNP |
| chr6_29911207_G_A | 29911207 | 0.7 | 0.5 | 0.9 | 0.021581 | 0.41 | 0.41 | 0.41 | SNP |
| chr6_29908676_T_A | 29908676 | 1.6 | 1.1 | 2.5 | 0.021673 | 0.14 | 0.16 | 0.14 | SNP |
| chr6_32193945_G_T | 32193945 | 0.7 | 0.5 | 0.9 | 0.021729 | 0.17 | 0.13 | 0.17 | SNP |
| chr6_29896105_G_A | 29896105 | 1.4 | 1.0 | 2.0 | 0.021736 | 0.22 | 0.25 | 0.22 | SNP |
| chr6_32628428_C_A | 32628428 | 2.1 | 1.1 | 4.0 | 0.021869 | 0.12 | 0.12 | 0.12 | SNP |
| chr6_33036617_C_T | 33036617 | 0.6 | 0.4 | 0.9 | 0.021932 | 0.15 | 0.09 | 0.15 | SNP |
| chr6_29913137_A_G | 29913137 | 1.6 | 1.1 | 2.5 | 0.021955 | 0.13 | 0.15 | 0.13 | SNP |
| chr6_32170433_C_T | 32170433 | 0.7 | 0.5 | 1.0 | 0.022160 | 0.47 | 0.43 | 0.48 | SNP |
| chr6_32172993_C_T | 32172993 | 2.0 | 1.1 | 3.6 | 0.022181 | 0.13 | 0.13 | 0.13 | SNP |

| | | | | | | | | | |
|---|---|---|---|---|---|---|---|---|---|
| chr6_31382994_C_A | 31382994 | 1.5 | 1.1 | 2.0 | 0.022233 | 0.18 | 0.19 | 0.18 | SNP |
| chr6_31592820_C_T | 31592820 | 0.7 | 0.5 | 0.9 | 0.022304 | 0.34 | 0.33 | 0.34 | SNP |
| chr6_30515043_T_C | 30515043 | 1.8 | 1.1 | 3.1 | 0.022309 | 0.11 | 0.12 | 0.11 | SNP |
| chr6_29893139_G_A | 29893139 | 1.4 | 1.0 | 2.0 | 0.022318 | 0.21 | 0.24 | 0.21 | SNP |
| chr6_29911019_T_C | 29911019 | 0.7 | 0.5 | 1.0 | 0.022587 | 0.45 | 0.43 | 0.45 | SNP |
| chr6_32971708_G_A | 32971708 | 0.7 | 0.5 | 0.9 | 0.022589 | 0.20 | 0.14 | 0.20 | SNP |
| chr6_32333827_A_C | 32333827 | 2.2 | 1.1 | 4.5 | 0.022953 | 0.13 | 0.12 | 0.13 | SNP |
| chr6_33036630_G_T | 33036630 | 0.6 | 0.4 | 0.9 | 0.022981 | 0.15 | 0.09 | 0.15 | SNP |
| chr6_33036631_A_C | 33036631 | 0.6 | 0.4 | 0.9 | 0.022981 | 0.15 | 0.09 | 0.15 | SNP |
| chr6_29911684_C_T | 29911684 | 1.5 | 1.1 | 2.1 | 0.023070 | 0.19 | 0.21 | 0.18 | SNP |
| chr6_33036388_C_T | 33036388 | 0.6 | 0.4 | 0.9 | 0.023232 | 0.14 | 0.09 | 0.15 | SNP |
| chr6_32731313_T_A | 32731313 | 0.6 | 0.4 | 0.9 | 0.023241 | 0.21 | 0.14 | 0.21 | SNP |
| chr6_29911037_G_C | 29911037 | 0.7 | 0.5 | 1.0 | 0.023243 | 0.45 | 0.44 | 0.46 | SNP |
| chr6_29913881_T_C | 29913881 | 0.7 | 0.6 | 1.0 | 0.023308 | 0.40 | 0.40 | 0.40 | SNP |
| chr6_29896852_C_T | 29896852 | 1.4 | 1.0 | 2.0 | 0.023432 | 0.25 | 0.28 | 0.24 | SNP |
| chr6_31556581_T_A | 31556581 | 0.7 | 0.5 | 0.9 | 0.023492 | 0.24 | 0.22 | 0.24 | SNP |
| chr6_29712878_G_A | 29712878 | 2.2 | 1.1 | 4.2 | 0.023511 | 0.02 | 0.05 | 0.02 | SNP |
| chr6_31542476_C_A | 31542476 | 0.7 | 0.5 | 0.9 | 0.023565 | 0.16 | 0.15 | 0.16 | SNP |
| chr6_32797537_T_G | 32797537 | 0.7 | 0.5 | 0.9 | 0.023717 | 0.35 | 0.24 | 0.36 | SNP |
| chr6_33033266_T_A | 33033266 | 0.6 | 0.4 | 0.9 | 0.023751 | 0.14 | 0.09 | 0.15 | SNP |
| chr6_31795345_G_A | 31795345 | 0.3 | 0.1 | 0.8 | 0.023919 | 0.04 | 0.02 | 0.04 | SNP |
| chr6_31475127_G_A | 31475127 | 1.5 | 1.0 | 2.1 | 0.023949 | 0.28 | 0.31 | 0.28 | SNP |
| chr6_33037579_A_T | 33037579 | 0.6 | 0.4 | 0.9 | 0.024077 | 0.14 | 0.09 | 0.15 | SNP |
| chr6_33037580_T_G | 33037580 | 0.6 | 0.4 | 0.9 | 0.024077 | 0.14 | 0.09 | 0.15 | SNP |
| chr6_29892233_T_C | 29892233 | 1.4 | 1.0 | 1.9 | 0.024125 | 0.25 | 0.28 | 0.25 | SNP |
| chr6_32193449_T_C | 32193449 | 0.7 | 0.5 | 0.9 | 0.024156 | 0.16 | 0.13 | 0.17 | SNP |
| chr6_33386728_G_C | 33386728 | 1.9 | 1.1 | 3.4 | 0.024162 | 0.03 | 0.05 | 0.03 | SNP |
| chr6_32633285_T_C | 32633285 | 0.7 | 0.5 | 0.9 | 0.024186 | 0.36 | 0.28 | 0.37 | SNP |
| chr6_29907861_T_G | 29907861 | 1.4 | 1.0 | 2.0 | 0.024521 | 0.19 | 0.23 | 0.18 | SNP |
| chr6_32723715_C_A | 32723715 | 1.5 | 1.0 | 2.1 | 0.024547 | 0.09 | 0.13 | 0.09 | SNP |
| chr6_31506648_T_G | 31506648 | 1.4 | 1.0 | 2.0 | 0.024714 | 0.31 | 0.34 | 0.31 | SNP |
| chr6_31239681_T_A | 31239681 | 1.5 | 1.1 | 2.2 | 0.024761 | 0.40 | 0.43 | 0.40 | SNP |
| chr6_32363816_T_C | 32363816 | 1.4 | 1.0 | 1.9 | 0.024913 | 0.43 | 0.52 | 0.42 | SNP |
| chr6_33033267_G_A | 33033267 | 0.6 | 0.4 | 0.9 | 0.025147 | 0.14 | 0.09 | 0.15 | SNP |
| chr6_33035986_T_C | 33035986 | 0.6 | 0.4 | 0.9 | 0.025288 | 0.13 | 0.08 | 0.13 | SNP |
| chr6_33477013_T_G | 33477013 | 0.6 | 0.4 | 0.9 | 0.025418 | 0.11 | 0.08 | 0.11 | SNP |
| chr6_29913430_C_T | 29913430 | 1.5 | 1.0 | 2.1 | 0.025583 | 0.18 | 0.20 | 0.18 | SNP |
| chr6_31605199_G_C | 31605199 | 0.7 | 0.5 | 1.0 | 0.025653 | 0.44 | 0.43 | 0.44 | SNP |
| chr6_31930351_A_G | 31930351 | 0.4 | 0.2 | 0.8 | 0.025690 | 0.05 | 0.02 | 0.05 | SNP |
| chr6_32169621_T_A | 32169621 | 0.7 | 0.6 | 1.0 | 0.025742 | 0.48 | 0.43 | 0.48 | SNP |
| chr6_31836827_G_T | 31836827 | 1.6 | 1.1 | 2.3 | 0.025760 | 0.33 | 0.32 | 0.33 | SNP |
| chr6_32633502_T_C | 32633502 | 0.6 | 0.4 | 0.9 | 0.025791 | 0.13 | 0.11 | 0.13 | SNP |
| chr6_31464321_T_A | 31464321 | 0.7 | 0.5 | 1.0 | 0.025824 | 0.30 | 0.24 | 0.30 | SNP |
| chr6_29909071_G_A | 29909071 | 1.5 | 1.0 | 2.1 | 0.025951 | 0.19 | 0.20 | 0.18 | SNP |
| chr6_32184345_T_C | 32184345 | 0.7 | 0.4 | 0.9 | 0.026014 | 0.13 | 0.11 | 0.14 | SNP |
| chr6_32184574_G_A | 32184574 | 0.7 | 0.4 | 0.9 | 0.026014 | 0.13 | 0.11 | 0.14 | SNP |
| chr6_33553239_C_T | 33553239 | 2.6 | 1.0 | 5.6 | 0.026085 | 0.01 | 0.02 | 0.01 | SNP |
| chr6_33045659_T_C | 33045659 | 0.4 | 0.1 | 0.8 | 0.026092 | 0.04 | 0.02 | 0.04 | SNP |
| chr6_32299592_G_A | 32299592 | 0.7 | 0.5 | 0.9 | 0.026119 | 0.31 | 0.24 | 0.32 | SNP |
| chr6_32796521_C_T | 32796521 | 0.7 | 0.5 | 0.9 | 0.026134 | 0.22 | 0.15 | 0.23 | SNP |
| chr6_29892203_C_A | 29892203 | 1.4 | 1.0 | 1.9 | 0.026138 | 0.25 | 0.28 | 0.24 | SNP |
| chr6_33036505_G_C | 33036505 | 0.6 | 0.4 | 0.9 | 0.026201 | 0.15 | 0.09 | 0.15 | SNP |
| chr6_32439964_T_G | 32439964 | 0.7 | 0.5 | 1.0 | 0.026262 | 0.38 | 0.29 | 0.38 | SNP |
| chr6_32559380_G_A | 32559380 | 0.7 | 0.5 | 1.0 | 0.026314 | 0.49 | 0.43 | 0.50 | SNP |
| chr6_29895701_T_C | 29895701 | 1.4 | 1.0 | 1.9 | 0.026624 | 0.21 | 0.24 | 0.21 | SNP |
| chr6_31323353_G_A | 31323353 | 0.7 | 0.6 | 1.0 | 0.026659 | 0.36 | 0.35 | 0.36 | SNP |
| chr6_29909070_T_C | 29909070 | 0.8 | 0.6 | 1.0 | 0.026761 | 0.45 | 0.45 | 0.45 | SNP |
| chr6_29795657_G_A | 29795657 | 1.4 | 1.0 | 1.9 | 0.026771 | 0.39 | 0.42 | 0.39 | SNP |
| chr6_29945949_G_A | 29945949 | 1.7 | 1.1 | 2.7 | 0.026920 | 0.11 | 0.12 | 0.11 | SNP |
| xHLA_2field_DQB1_06_04 | 32627244 | 0.4 | 0.2 | 0.8 | 0.026952 | 0.05 | 0.02 | 0.05 | HLA (xHLA) |
| chr6_31322996_C_T | 31322996 | 0.7 | 0.6 | 1.0 | 0.027066 | 0.38 | 0.33 | 0.38 | SNP |
| chr6_33260353_A_G | 33260353 | 2.0 | 1.0 | 3.5 | 0.027068 | 0.03 | 0.05 | 0.03 | SNP |
| xHLA_2field_A_02_01 | 29910309 | 0.7 | 0.6 | 1.0 | 0.027390 | 0.35 | 0.36 | 0.35 | HLA (xHLA) |
| chr6_31837250_A_G | 31837250 | 1.4 | 1.0 | 1.9 | 0.027391 | 0.49 | 0.49 | 0.49 | SNP |
| chr6_31238708_G_A | 31238708 | 0.6 | 0.4 | 0.9 | 0.027398 | 0.09 | 0.07 | 0.10 | SNP |
| chr6_32083300_A_G | 32083300 | 0.6 | 0.4 | 0.9 | 0.027522 | 0.15 | 0.10 | 0.16 | SNP |
| chr6_33033390_A_T | 33033390 | 0.6 | 0.4 | 0.9 | 0.027570 | 0.15 | 0.09 | 0.15 | SNP |
| chr6_33033235_G_C | 33033235 | 0.6 | 0.4 | 0.9 | 0.027744 | 0.15 | 0.09 | 0.15 | SNP |
| chr6_32377116_C_G | 32377116 | 1.5 | 1.0 | 2.1 | 0.027922 | 0.48 | 0.56 | 0.47 | SNP |
| chr6_29911198_T_C | 29911198 | 0.7 | 0.5 | 1.0 | 0.027999 | 0.41 | 0.41 | 0.41 | SNP |
| chr6_29912796_G_A | 29912796 | 0.8 | 0.6 | 1.0 | 0.028011 | 0.41 | 0.41 | 0.41 | SNP |
| chr6_31322303_C_G | 31322303 | 0.7 | 0.6 | 1.0 | 0.028046 | 0.34 | 0.30 | 0.35 | SNP |
| chr6_32797684_A_G | 32797684 | 0.7 | 0.5 | 1.0 | 0.028094 | 0.36 | 0.26 | 0.37 | SNP |
| chr6_33382943_T_G | 33382943 | 0.6 | 0.3 | 0.9 | 0.028218 | 0.09 | 0.06 | 0.09 | SNP |
| chr6_32711050_C_T | 32711050 | 0.6 | 0.4 | 0.9 | 0.028583 | 0.21 | 0.14 | 0.21 | SNP |
| chr6_31839309_T_C | 31839309 | 1.4 | 1.0 | 1.9 | 0.028690 | 0.50 | 0.50 | 0.50 | SNP |
| chr6_31320263_C_T | 31320263 | 0.6 | 0.4 | 0.9 | 0.028724 | 0.10 | 0.08 | 0.11 | SNP |
| chr6_32189192_G_A | 32189192 | 0.3 | 0.1 | 0.8 | 0.029107 | 0.04 | 0.01 | 0.04 | SNP |
| chr6_32131667_G_A | 32131667 | 0.3 | 0.1 | 0.8 | 0.029245 | 0.04 | 0.01 | 0.04 | SNP |
| chr6_29913135_G_C | 29913135 | 0.8 | 0.6 | 1.0 | 0.029249 | 0.41 | 0.41 | 0.41 | SNP |
| chr6_29913639_A_G | 29913639 | 1.5 | 1.0 | 2.1 | 0.029285 | 0.19 | 0.20 | 0.18 | SNP |
| chr6_29909021_C_G | 29909021 | 1.6 | 1.0 | 2.4 | 0.029286 | 0.13 | 0.15 | 0.13 | SNP |
| chr6_32636514_A_G | 32636514 | 0.7 | 0.5 | 1.0 | 0.029509 | 0.18 | 0.15 | 0.18 | SNP |
| chr6_32557301_T_C | 32557301 | 0.4 | 0.1 | 0.8 | 0.029577 | 0.04 | 0.02 | 0.04 | SNP |
| chr6_32521796_A_C | 32521796 | 0.7 | 0.5 | 1.0 | 0.029583 | 0.29 | 0.21 | 0.30 | SNP |
| chr6_31238522_C_T | 31238522 | 0.7 | 0.5 | 1.0 | 0.029739 | 0.20 | 0.16 | 0.20 | SNP |
| chr6_29914011_G_T | 29914011 | 0.7 | 0.6 | 1.0 | 0.029744 | 0.40 | 0.40 | 0.40 | SNP |
| chr6_33036177_A_G | 33036177 | 0.6 | 0.4 | 0.9 | 0.029772 | 0.15 | 0.09 | 0.15 | SNP |
| chr6_32171683_T_C | 32171683 | 0.7 | 0.6 | 1.0 | 0.029793 | 0.47 | 0.43 | 0.47 | SNP |
| chr6_29913734_C_T | 29913734 | 0.8 | 0.6 | 1.0 | 0.029918 | 0.40 | 0.41 | 0.40 | SNP |
| chr6_33037866_A_G | 33037866 | 0.6 | 0.4 | 0.9 | 0.029922 | 0.14 | 0.09 | 0.14 | SNP |
| chr6_32713867_C_T | 32713867 | 0.6 | 0.4 | 0.9 | 0.030208 | 0.20 | 0.14 | 0.20 | SNP |
| chr6_33039484_A_G | 33039484 | 0.6 | 0.4 | 0.9 | 0.030651 | 0.14 | 0.09 | 0.15 | SNP |
| chr6_29796115_A_C | 29796115 | 1.4 | 1.0 | 1.9 | 0.030748 | 0.43 | 0.46 | 0.43 | SNP |
| chr6_31635710_T_C | 31635710 | 1.4 | 1.0 | 1.8 | 0.030803 | 0.20 | 0.24 | 0.20 | SNP |
| chr6_29909924_G_A | 29909924 | 0.8 | 0.6 | 1.0 | 0.031011 | 0.45 | 0.45 | 0.45 | SNP |
| chr6_32634992_T_C | 32634992 | 0.7 | 0.4 | 1.0 | 0.031246 | 0.13 | 0.11 | 0.13 | SNP |
| chr6_32377061_C_T | 32377061 | 1.4 | 1.0 | 2.0 | 0.031295 | 0.48 | 0.57 | 0.47 | SNP |
| chr6_29910725_C_G | 29910725 | 1.6 | 1.0 | 2.5 | 0.031300 | 0.10 | 0.13 | 0.09 | SNP |
| chr6_31602967_G_A | 31602967 | 1.4 | 1.0 | 1.8 | 0.031551 | 0.37 | 0.39 | 0.37 | SNP |
| chr6_32629497_A_G | 32629497 | 0.7 | 0.4 | 1.0 | 0.031898 | 0.13 | 0.11 | 0.13 | SNP |
| chr6_32780914_G_A | 32780914 | 0.6 | 0.3 | 0.9 | 0.032012 | 0.10 | 0.06 | 0.10 | SNP |
| chr6_32559371_T_C | 32559371 | 0.7 | 0.5 | 1.0 | 0.032012 | 0.49 | 0.43 | 0.50 | SNP |
| chr6_32634074_C_T | 32634074 | 0.7 | 0.4 | 1.0 | 0.032106 | 0.13 | 0.11 | 0.13 | SNP |
| chr6_32633640_T_A | 32633640 | 0.7 | 0.4 | 1.0 | 0.032235 | 0.13 | 0.11 | 0.13 | SNP |
| chr6_32633646_C_T | 32633646 | 0.7 | 0.4 | 1.0 | 0.032235 | 0.13 | 0.11 | 0.13 | SNP |
| chr6_31084435_G_A | 31084435 | 0.7 | 0.5 | 1.0 | 0.032356 | 0.32 | 0.31 | 0.32 | SNP |
| chr6_32633281_C_T | 32633281 | 0.7 | 0.4 | 1.0 | 0.032374 | 0.13 | 0.11 | 0.13 | SNP |
| chr6_31239430_C_T | 31239430 | 0.7 | 0.5 | 1.0 | 0.032479 | 0.48 | 0.47 | 0.48 | SNP |
| chr6_32150296_C_A | 32150296 | 0.5 | 0.2 | 0.9 | 0.032559 | 0.06 | 0.03 | 0.07 | SNP |
| chr6_32609594_G_A | 32609594 | 2.1 | 1.0 | 4.0 | 0.032611 | 0.13 | 0.15 | 0.13 | SNP |
| chr6_32550587_T_G | 32550587 | 0.4 | 0.2 | 0.9 | 0.032690 | 0.05 | 0.02 | 0.05 | SNP |
| chr6_29913817_C_T | 29913817 | 0.8 | 0.6 | 1.0 | 0.032691 | 0.40 | 0.41 | 0.40 | SNP |
| chr6_32052444_C_T | 32052444 | 0.4 | 0.2 | 0.9 | 0.032848 | 0.04 | 0.02 | 0.04 | SNP |
| chr6_32634899_A_G | 32634899 | 0.7 | 0.4 | 1.0 | 0.032920 | 0.13 | 0.11 | 0.13 | SNP |
| chr6_31537606_C_T | 31537606 | 0.7 | 0.5 | 1.0 | 0.032985 | 0.16 | 0.15 | 0.16 | SNP |
| chr6_32633836_A_C | 32633836 | 0.7 | 0.4 | 1.0 | 0.033065 | 0.13 | 0.11 | 0.13 | SNP |
| chr6_33035982_G_A | 33035982 | 0.6 | 0.4 | 0.9 | 0.033087 | 0.13 | 0.08 | 0.13 | SNP |
| chr6_32408735_A_C | 32408735 | 0.7 | 0.5 | 1.0 | 0.033154 | 0.34 | 0.24 | 0.35 | SNP |
| chr6_32636495_G_A | 32636495 | 0.7 | 0.5 | 1.0 | 0.033230 | 0.18 | 0.15 | 0.18 | SNP |
| chr6_32945530_C_G | 32945530 | 0.6 | 0.4 | 0.9 | 0.033230 | 0.11 | 0.07 | 0.11 | SNP |
| chr6_32628084_C_T | 32628084 | 2.0 | 1.0 | 3.8 | 0.033273 | 0.13 | 0.13 | 0.13 | SNP |
| chr6_31382932_G_A | 31382932 | 1.4 | 1.0 | 1.9 | 0.033318 | 0.23 | 0.23 | 0.23 | SNP |
| chr6_32707040_G_A | 32707040 | 0.6 | 0.4 | 1.0 | 0.033390 | 0.21 | 0.15 | 0.22 | SNP |
| chr6_33036951_A_G | 33036951 | 0.6 | 0.4 | 1.0 | 0.033444 | 0.14 | 0.09 | 0.14 | SNP |
| chr6_31369151_G_A | 31369151 | 0.7 | 0.5 | 1.0 | 0.033489 | 0.34 | 0.32 | 0.34 | SNP |
| chr6_31125777_C_A | 31125777 | 1.4 | 1.0 | 1.9 | 0.033512 | 0.28 | 0.29 | 0.28 | SNP |
| chr6_32634229_C_T | 32634229 | 0.7 | 0.4 | 1.0 | 0.033610 | 0.13 | 0.11 | 0.13 | SNP |
| chr6_32636518_A_C | 32636518 | 0.7 | 0.5 | 1.0 | 0.033672 | 0.18 | 0.15 | 0.18 | SNP |
| xHLA_2field_DPB1_02_01 | 33043767 | 1.4 | 1.0 | 2.0 | 0.033715 | 0.13 | 0.16 | 0.12 | HLA (xHLA) |
| chr6_31606756_A_T | 31606756 | 0.6 | 0.4 | 0.9 | 0.033771 | 0.11 | 0.07 | 0.11 | SNP |
| chr6_32790167_C_G | 32790167 | 0.7 | 0.5 | 1.0 | 0.033885 | 0.23 | 0.16 | 0.24 | SNP |
| chr6_32168771_G_T | 32168771 | 1.9 | 1.0 | 3.4 | 0.033907 | 0.13 | 0.13 | 0.13 | SNP |
| chr6_31675501_G_A | 31675501 | 0.5 | 0.3 | 0.9 | 0.034019 | 0.07 | 0.04 | 0.07 | SNP |
| chr6_29910761_G_A | 29910761 | 1.4 | 1.0 | 2.0 | 0.034128 | 0.32 | 0.35 | 0.32 | SNP |
| chr6_33641897_G_A | 33641897 | 0.6 | 0.3 | 0.9 | 0.034220 | 0.11 | 0.07 | 0.11 | SNP |
| xHLA_2field_DQB1_03_01 | 32627244 | 0.6 | 0.4 | 1.0 | 0.034228 | 0.13 | 0.11 | 0.13 | HLA (xHLA) |
| chr6_31239577_C_A | 31239577 | 1.4 | 1.0 | 2.0 | 0.034274 | 0.47 | 0.50 | 0.47 | SNP |
| chr6_32284108_T_G | 32284108 | 2.1 | 1.0 | 4.2 | 0.034362 | 0.11 | 0.11 | 0.11 | SNP |
| chr6_31538244_C_G | 31538244 | 0.7 | 0.5 | 1.0 | 0.034366 | 0.16 | 0.15 | 0.16 | SNP |
| chr6_29913912_C_T | 29913912 | 0.8 | 0.6 | 1.0 | 0.034564 | 0.40 | 0.41 | 0.40 | SNP |
| chr6_33037304_G_T | 33037304 | 0.6 | 0.4 | 1.0 | 0.034653 | 0.14 | 0.09 | 0.15 | SNP |
| chr6_32634614_A_C | 32634614 | 0.7 | 0.6 | 1.0 | 0.034663 | 0.45 | 0.36 | 0.46 | SNP |
| chr6_29908804_G_A | 29908804 | 0.8 | 0.6 | 1.0 | 0.034799 | 0.41 | 0.41 | 0.41 | SNP |
| chr6_29911063_T_G | 29911063 | 0.7 | 0.5 | 1.0 | 0.034808 | 0.40 | 0.39 | 0.40 | SNP |
| chr6_32781076_C_A | 32781076 | 0.6 | 0.3 | 0.9 | 0.034819 | 0.10 | 0.06 | 0.10 | SNP |
| chr6_32796653_C_A | 32796653 | 0.7 | 0.5 | 1.0 | 0.034822 | 0.23 | 0.15 | 0.24 | SNP |
| chr6_32628771_T_C | 32628771 | 0.7 | 0.4 | 1.0 | 0.034914 | 0.13 | 0.11 | 0.13 | SNP |

| | | | | | | | | | |
|---|---|---|---|---|---|---|---|---|---|
| chr6_29908469_T_C | 29908469 | 0.8 | 0.6 | 1.0 | 0.035012 | 0.41 | 0.41 | 0.41 | SNP |
| chr6_29691090_T_A | 29691090 | 0.8 | 0.6 | 1.0 | 0.035149 | 0.47 | 0.46 | 0.47 | SNP |
| chr6_30524614_A_G | 30524614 | 2.1 | 1.0 | 4.2 | 0.035178 | 0.02 | 0.04 | 0.02 | SNP |
| chr6_32797115_T_G | 32797115 | 0.7 | 0.5 | 1.0 | 0.035412 | 0.30 | 0.25 | 0.30 | SNP |
| chr6_33037104_C_T | 33037104 | 0.6 | 0.4 | 1.0 | 0.035486 | 0.14 | 0.09 | 0.15 | SNP |
| chr6_32826450_T_C | 32826450 | 0.8 | 0.6 | 1.0 | 0.035507 | 0.45 | 0.39 | 0.46 | SNP |
| chr6_29891850_A_G | 29891850 | 1.3 | 1.0 | 1.7 | 0.035518 | 0.42 | 0.43 | 0.42 | SNP |
| chr6_33036287_G_A | 33036287 | 0.7 | 0.4 | 1.0 | 0.035520 | 0.15 | 0.10 | 0.15 | SNP |
| chr6_32944746_G_A | 32944746 | 1.3 | 1.0 | 1.7 | 0.035590 | 0.42 | 0.50 | 0.42 | SNP |
| chr6_33036312_A_G | 33036312 | 0.7 | 0.4 | 1.0 | 0.035596 | 0.15 | 0.10 | 0.15 | SNP |
| chr6_31620020_A_G | 31620020 | 1.4 | 1.0 | 1.9 | 0.035905 | 0.38 | 0.41 | 0.38 | SNP |
| chr6_32633462_T_A | 32633462 | 0.7 | 0.4 | 1.0 | 0.035997 | 0.13 | 0.11 | 0.13 | SNP |
| chr6_33036288_G_A | 33036288 | 0.7 | 0.4 | 1.0 | 0.036112 | 0.15 | 0.10 | 0.15 | SNP |
| chr6_31325947_A_C | 31325947 | 1.4 | 1.0 | 2.0 | 0.036300 | 0.14 | 0.16 | 0.14 | SNP |
| chr6_29796257_C_T | 29796257 | 1.4 | 1.0 | 1.9 | 0.036345 | 0.44 | 0.48 | 0.44 | SNP |
| chr6_29940202_G_A | 29940202 | 1.4 | 1.0 | 1.9 | 0.036580 | 0.27 | 0.28 | 0.27 | SNP |
| chr6_31902645_A_G | 31902645 | 0.5 | 0.2 | 0.9 | 0.036738 | 0.06 | 0.02 | 0.06 | SNP |
| chr6_33036233_A_G | 33036233 | 0.7 | 0.4 | 1.0 | 0.036787 | 0.15 | 0.10 | 0.15 | SNP |
| chr6_29895913_G_A | 29895913 | 0.8 | 0.6 | 1.0 | 0.037030 | 0.45 | 0.45 | 0.45 | SNP |
| chr6_31322133_A_G | 31322133 | 0.7 | 0.6 | 1.0 | 0.037335 | 0.35 | 0.31 | 0.35 | SNP |
| chr6_33033202_C_T | 33033202 | 0.7 | 0.4 | 1.0 | 0.037340 | 0.15 | 0.10 | 0.15 | SNP |
| chr6_31322402_C_T | 31322402 | 1.3 | 1.0 | 1.8 | 0.037583 | 0.34 | 0.37 | 0.34 | SNP |
| chr6_32797109_G_A | 32797109 | 0.7 | 0.5 | 1.0 | 0.037602 | 0.22 | 0.15 | 0.23 | SNP |
| chr6_33033388_A_C | 33033388 | 0.6 | 0.4 | 1.0 | 0.037932 | 0.15 | 0.09 | 0.15 | SNP |
| chr6_33037294_T_C | 33037294 | 0.6 | 0.4 | 1.0 | 0.038058 | 0.14 | 0.09 | 0.15 | SNP |
| chr6_29908967_G_A | 29908967 | 0.8 | 0.6 | 1.0 | 0.038148 | 0.41 | 0.41 | 0.41 | SNP |
| chr6_31902289_C_G | 31902289 | 0.5 | 0.2 | 0.9 | 0.038176 | 0.06 | 0.02 | 0.06 | SNP |
| chr6_31510105_G_A | 31510105 | 1.4 | 1.0 | 2.0 | 0.038253 | 0.31 | 0.34 | 0.31 | SNP |
| chr6_33036242_T_C | 33036242 | 0.7 | 0.4 | 1.0 | 0.038260 | 0.15 | 0.10 | 0.15 | SNP |
| chr6_32628939_C_G | 32628939 | 0.7 | 0.4 | 1.0 | 0.038489 | 0.13 | 0.11 | 0.13 | SNP |
| chr6_33037522_T_C | 33037522 | 0.7 | 0.4 | 1.0 | 0.038561 | 0.15 | 0.10 | 0.15 | SNP |
| chr6_31509372_A_G | 31509372 | 1.4 | 1.0 | 2.0 | 0.038648 | 0.33 | 0.36 | 0.32 | SNP |
| chr6_32605935_G_T | 32605935 | 1.9 | 1.0 | 3.6 | 0.038699 | 0.12 | 0.12 | 0.12 | SNP |
| chr6_31601520_T_C | 31601520 | 1.4 | 1.0 | 1.9 | 0.038729 | 0.19 | 0.22 | 0.18 | SNP |
| chr6_33037865_C_T | 33037865 | 0.6 | 0.4 | 1.0 | 0.038771 | 0.14 | 0.09 | 0.14 | SNP |
| chr6_33033146_T_C | 33033146 | 0.7 | 0.4 | 1.0 | 0.038780 | 0.15 | 0.10 | 0.15 | SNP |
| chr6_31903121_G_A | 31903121 | 0.5 | 0.2 | 0.9 | 0.038995 | 0.06 | 0.03 | 0.06 | SNP |
| chr6_29912463_G_A | 29912463 | 1.3 | 1.0 | 1.7 | 0.039163 | 0.49 | 0.49 | 0.49 | SNP |
| chr6_31240959_C_G | 31240959 | 0.7 | 0.5 | 1.0 | 0.039205 | 0.23 | 0.19 | 0.23 | SNP |
| chr6_32375347_T_G | 32375347 | 0.4 | 0.1 | 0.9 | 0.039208 | 0.04 | 0.02 | 0.04 | SNP |
| chr6_32304466_T_A | 32304466 | 0.4 | 0.1 | 0.9 | 0.039299 | 0.04 | 0.02 | 0.05 | SNP |
| chr6_29910402_G_A | 29910402 | 1.5 | 1.0 | 2.3 | 0.039410 | 0.09 | 0.13 | 0.09 | SNP |
| chr6_32363955_T_C | 32363955 | 1.4 | 1.0 | 1.8 | 0.039442 | 0.43 | 0.52 | 0.43 | SNP |
| chr6_32904981_A_G | 32904981 | 0.7 | 0.5 | 1.0 | 0.039492 | 0.21 | 0.17 | 0.21 | SNP |
| chr6_33037557_C_T | 33037557 | 0.6 | 0.4 | 1.0 | 0.039935 | 0.11 | 0.06 | 0.11 | SNP |
| chr6_32635164_T_C | 32635164 | 0.7 | 0.6 | 1.0 | 0.040040 | 0.45 | 0.37 | 0.46 | SNP |
| chr6_32604221_A_G | 32604221 | 1.9 | 1.0 | 3.6 | 0.040108 | 0.13 | 0.12 | 0.13 | SNP |
| chr6_32707023_T_C | 32707023 | 0.6 | 0.4 | 1.0 | 0.040243 | 0.21 | 0.14 | 0.22 | SNP |
| chr6_32785275_G_A | 32785275 | 0.6 | 0.4 | 1.0 | 0.040464 | 0.10 | 0.06 | 0.10 | SNP |
| chr6_32605884_C_T | 32605884 | 1.9 | 1.0 | 3.6 | 0.040690 | 0.12 | 0.12 | 0.12 | SNP |
| chr6_32007063_C_T | 32007063 | 0.7 | 0.4 | 1.0 | 0.040792 | 0.27 | 0.20 | 0.27 | SNP |
| chr6_32628922_G_C | 32628922 | 0.7 | 0.5 | 1.0 | 0.040929 | 0.13 | 0.11 | 0.13 | SNP |
| chr6_29909044_G_C | 29909044 | 0.8 | 0.6 | 1.0 | 0.040959 | 0.41 | 0.41 | 0.40 | SNP |
| chr6_32628722_A_T | 32628722 | 0.7 | 0.5 | 1.0 | 0.041073 | 0.13 | 0.11 | 0.13 | SNP |
| chr6_32635001_T_C | 32635001 | 0.7 | 0.5 | 1.0 | 0.041377 | 0.13 | 0.11 | 0.13 | SNP |
| chr6_31248432_A_G | 31248432 | 0.7 | 0.5 | 1.0 | 0.041402 | 0.47 | 0.48 | 0.47 | SNP |
| chr6_33033170_T_C | 33033170 | 0.7 | 0.4 | 1.0 | 0.041599 | 0.14 | 0.10 | 0.15 | SNP |
| chr6_33033058_T_C | 33033058 | 0.7 | 0.4 | 1.0 | 0.041987 | 0.14 | 0.10 | 0.15 | SNP |
| chr6_33036210_G_T | 33036210 | 0.7 | 0.4 | 1.0 | 0.042050 | 0.14 | 0.10 | 0.15 | SNP |
| chr6_29713155_G_C | 29713155 | 1.5 | 1.0 | 2.2 | 0.042062 | 0.09 | 0.12 | 0.09 | SNP |
| chr6_31925921_G_A | 31925921 | 0.4 | 0.2 | 0.9 | 0.042240 | 0.04 | 0.02 | 0.04 | SNP |
| chr6_33037080_G_A | 33037080 | 0.7 | 0.4 | 1.0 | 0.042260 | 0.14 | 0.09 | 0.15 | SNP |
| chr6_33037082_A_G | 33037082 | 0.7 | 0.4 | 1.0 | 0.042260 | 0.14 | 0.09 | 0.15 | SNP |
| chr6_32946133_T_C | 32946133 | 1.3 | 1.0 | 1.7 | 0.042266 | 0.42 | 0.50 | 0.41 | SNP |
| chr6_33033188_C_T | 33033188 | 0.7 | 0.4 | 1.0 | 0.042487 | 0.14 | 0.10 | 0.15 | SNP |
| chr6_32323905_C_A | 32323905 | 0.4 | 0.1 | 0.9 | 0.042621 | 0.04 | 0.02 | 0.05 | SNP |
| chr6_33547430_G_A | 33547430 | 0.7 | 0.5 | 1.0 | 0.042621 | 0.18 | 0.15 | 0.18 | SNP |
| chr6_31373174_C_T | 31373174 | 0.7 | 0.6 | 1.0 | 0.042708 | 0.36 | 0.34 | 0.36 | SNP |
| chr6_31930063_G_A | 31930063 | 1.7 | 1.0 | 2.8 | 0.042733 | 0.04 | 0.06 | 0.04 | SNP |
| chr6_32006337_A_C | 32006337 | 0.7 | 0.5 | 1.0 | 0.042800 | 0.34 | 0.28 | 0.34 | SNP |
| chr6_32633948_C_A | 32633948 | 0.7 | 0.5 | 1.0 | 0.042840 | 0.13 | 0.11 | 0.13 | SNP |
| chr6_32605266_G_A | 32605266 | 0.6 | 0.3 | 0.9 | 0.042845 | 0.07 | 0.04 | 0.08 | SNP |
| chr6_33476718_T_C | 33476718 | 0.8 | 0.6 | 1.0 | 0.042871 | 0.35 | 0.30 | 0.36 | SNP |
| chr6_32628597_C_T | 32628597 | 0.7 | 0.5 | 1.0 | 0.043119 | 0.13 | 0.12 | 0.14 | SNP |
| chr6_31378640_A_G | 31378640 | 1.6 | 1.0 | 2.5 | 0.043139 | 0.08 | 0.09 | 0.07 | SNP |
| chr6_29908659_T_C | 29908659 | 0.8 | 0.6 | 1.0 | 0.043145 | 0.43 | 0.43 | 0.43 | SNP |
| chr6_32633854_A_G | 32633854 | 0.7 | 0.5 | 1.0 | 0.043163 | 0.13 | 0.11 | 0.13 | SNP |
| chr6_33033178_G_A | 33033178 | 0.7 | 0.4 | 1.0 | 0.043203 | 0.14 | 0.10 | 0.15 | SNP |
| chr6_33045896_A_G | 33045896 | 0.4 | 0.1 | 0.9 | 0.043259 | 0.04 | 0.02 | 0.04 | SNP |
| chr6_33037419_G_A | 33037419 | 0.6 | 0.4 | 1.0 | 0.043653 | 0.14 | 0.09 | 0.14 | SNP |
| chr6_32408012_G_A | 32408012 | 0.7 | 0.5 | 1.0 | 0.043681 | 0.33 | 0.24 | 0.34 | SNP |
| chr6_33033177_G_A | 33033177 | 0.7 | 0.4 | 1.0 | 0.043708 | 0.14 | 0.10 | 0.15 | SNP |
| chr6_31509355_A_G | 31509355 | 1.4 | 1.0 | 1.9 | 0.043763 | 0.33 | 0.36 | 0.33 | SNP |
| chr6_31932209_G_A | 31932209 | 0.3 | 0.1 | 0.8 | 0.043778 | 0.04 | 0.01 | 0.04 | SNP |
| xHLA_2field_DRB1_11_01 | 32546552 | 0.4 | 0.1 | 0.9 | 0.043804 | 0.04 | 0.02 | 0.04 | HLA (xHLA) |
| chr6_33045891_C_T | 33045891 | 0.4 | 0.1 | 0.9 | 0.043864 | 0.04 | 0.02 | 0.04 | SNP |
| chr6_32340596_T_A | 32340596 | 0.4 | 0.2 | 0.9 | 0.044196 | 0.05 | 0.02 | 0.05 | SNP |
| chr6_32781121_G_C | 32781121 | 0.6 | 0.4 | 1.0 | 0.044252 | 0.10 | 0.06 | 0.10 | SNP |
| chr6_32796967_G_A | 32796967 | 0.7 | 0.5 | 1.0 | 0.044365 | 0.23 | 0.15 | 0.24 | SNP |
| chr6_31709814_G_C | 31709814 | 0.6 | 0.3 | 1.0 | 0.044375 | 0.06 | 0.07 | 0.06 | SNP |
| chr6_32076499_G_A | 32076499 | 0.7 | 0.5 | 1.0 | 0.044480 | 0.36 | 0.36 | 0.36 | SNP |
| chr6_30229306_A_G | 30229306 | 1.4 | 1.0 | 1.8 | 0.044585 | 0.32 | 0.33 | 0.32 | SNP |
| chr6_32629425_G_A | 32629425 | 0.7 | 0.5 | 1.0 | 0.044606 | 0.13 | 0.11 | 0.13 | SNP |
| chr6_33032822_G_A | 33032822 | 0.7 | 0.4 | 1.0 | 0.044631 | 0.14 | 0.09 | 0.15 | SNP |
| chr6_29908426_C_T | 29908426 | 0.8 | 0.6 | 1.0 | 0.044782 | 0.41 | 0.41 | 0.41 | SNP |
| chr6_33037424_T_C | 33037424 | 0.7 | 0.4 | 1.0 | 0.044813 | 0.14 | 0.09 | 0.14 | SNP |
| chr6_31322459_T_C | 31322459 | 1.3 | 1.0 | 1.7 | 0.044834 | 0.35 | 0.37 | 0.34 | SNP |
| chr6_32192331_G_A | 32192331 | 1.3 | 1.0 | 1.8 | 0.044957 | 0.47 | 0.56 | 0.46 | SNP |
| chr6_30880156_G_A | 30880156 | 1.8 | 1.0 | 3.1 | 0.044963 | 0.03 | 0.06 | 0.03 | SNP |
| chr6_32063896_G_A | 32063896 | 0.3 | 0.1 | 0.8 | 0.045295 | 0.04 | 0.01 | 0.04 | SNP |
| chr6_31322047_A_G | 31322047 | 0.7 | 0.5 | 1.0 | 0.045382 | 0.15 | 0.15 | 0.16 | SNP |
| chr6_31558978_G_C | 31558978 | 0.7 | 0.5 | 1.0 | 0.045739 | 0.14 | 0.14 | 0.14 | SNP |
| chr6_32634765_T_C | 32634765 | 0.7 | 0.5 | 1.0 | 0.045788 | 0.13 | 0.12 | 0.13 | SNP |
| chr6_33037187_C_G | 33037187 | 0.7 | 0.4 | 1.0 | 0.045790 | 0.15 | 0.10 | 0.15 | SNP |
| chr6_31539767_C_T | 31539767 | 0.7 | 0.5 | 1.0 | 0.046499 | 0.15 | 0.15 | 0.15 | SNP |
| chr6_31321681_C_T | 31321681 | 0.7 | 0.5 | 1.0 | 0.046654 | 0.20 | 0.22 | 0.20 | SNP |
| chr6_31509967_G_T | 31509967 | 0.7 | 0.5 | 1.0 | 0.046817 | 0.16 | 0.16 | 0.16 | SNP |
| chr6_32785284_T_C | 32785284 | 0.6 | 0.4 | 1.0 | 0.047211 | 0.10 | 0.06 | 0.10 | SNP |
| chr6_29944670_G_C | 29944670 | 1.3 | 1.0 | 1.8 | 0.047420 | 0.46 | 0.46 | 0.46 | SNP |
| chr6_32709965_G_T | 32709965 | 0.6 | 0.4 | 1.0 | 0.047542 | 0.20 | 0.14 | 0.21 | SNP |
| chr6_31326027_A_C | 31326027 | 1.4 | 1.0 | 1.9 | 0.047592 | 0.14 | 0.16 | 0.14 | SNP |
| chr6_32629613_C_G | 32629613 | 0.7 | 0.5 | 1.0 | 0.047821 | 0.13 | 0.11 | 0.13 | SNP |
| chr6_32629618_G_A | 32629618 | 0.7 | 0.5 | 1.0 | 0.047821 | 0.13 | 0.11 | 0.13 | SNP |
| chr6_31322688_T_C | 31322688 | 1.3 | 1.0 | 1.7 | 0.047874 | 0.35 | 0.37 | 0.34 | SNP |
| chr6_31634570_A_C | 31634570 | 0.5 | 0.3 | 1.0 | 0.048148 | 0.06 | 0.04 | 0.07 | SNP |
| chr6_31655438_G_A | 31655438 | 0.5 | 0.3 | 1.0 | 0.048243 | 0.06 | 0.04 | 0.07 | SNP |
| chr6_31320347_C_T | 31320347 | 0.7 | 0.5 | 1.0 | 0.048293 | 0.18 | 0.16 | 0.18 | SNP |
| chr6_29943715_C_T | 29943715 | 1.3 | 1.0 | 1.7 | 0.048364 | 0.40 | 0.41 | 0.40 | SNP |
| chr6_29910986_G_A | 29910986 | 0.7 | 0.5 | 1.0 | 0.048747 | 0.35 | 0.35 | 0.35 | SNP |
| chr6_32017524_A_G | 32017524 | 0.4 | 0.1 | 0.9 | 0.049014 | 0.04 | 0.01 | 0.04 | SNP |
| chr6_32627938_C_T | 32627938 | 0.7 | 0.5 | 1.0 | 0.049171 | 0.13 | 0.12 | 0.13 | SNP |
| chr6_31731014_C_G | 31731014 | 1.4 | 1.0 | 1.9 | 0.049225 | 0.17 | 0.21 | 0.17 | SNP |
| chr6_32796346_T_C | 32796346 | 0.7 | 0.5 | 1.0 | 0.049253 | 0.23 | 0.16 | 0.24 | SNP |
| chr6_31839494_C_T | 31839494 | 0.7 | 0.5 | 1.0 | 0.049397 | 0.48 | 0.48 | 0.48 | SNP |

ANCA = anitneutrophil cytoplasmic antibody; MPO-ANCA+ = myeloperoxidase ANCA positive; AAV = ANCA-associated vasculitis; SNP = single nucleotide polymorphism; OR = Odds ratio; CI = confidence interval; MAF = minor allele frequency. SNPs and HLA alleles with P < 0.05 included in the table.

**Supplemtary Table 5.** Association analysis of patients with MPA versus controls, adjusting for sex and genetic structure (principal components 1-4).

| Variant | position | OR | CI_lower | CI_upper | p | MAF | MAF_cases | MAF_controls | Type (SNP, HLA (xHLA)) |
|---|---|---|---|---|---|---|---|---|---|
| xHLA_2field_DRB1_04_04 | 32546552 | 3.9 | 2.6 | 5.8 | 8.64E-12 | 0.07 | 0.20 | 0.06 | HLA (xHLA) |
| chr6_32559648_G_T | 32559648 | 3.4 | 2.3 | 5.1 | 3.09E-09 | 0.20 | 0.38 | 0.18 | SNP |
| chr6_32609479_C_A | 32609479 | 3.1 | 2.1 | 4.6 | 1.11E-08 | 0.22 | 0.40 | 0.20 | SNP |
| chr6_32604585_A_T | 32604585 | 3.2 | 2.1 | 4.8 | 1.72E-08 | 0.22 | 0.41 | 0.21 | SNP |
| chr6_32559191_A_G | 32559191 | 3.0 | 2.1 | 4.5 | 2.18E-08 | 0.21 | 0.39 | 0.19 | SNP |
| chr6_32605555_T_A | 32605555 | 2.9 | 2.0 | 4.3 | 3.21E-08 | 0.22 | 0.40 | 0.21 | SNP |
| chr6_32635954_G_A | 32635954 | 2.9 | 2.0 | 4.3 | 3.35E-08 | 0.15 | 0.31 | 0.14 | SNP |
| chr6_32606987_T_G | 32606987 | 2.9 | 2.0 | 4.2 | 3.88E-08 | 0.22 | 0.40 | 0.21 | SNP |
| chr6_32605609_G_A | 32605609 | 2.9 | 2.0 | 4.3 | 3.95E-08 | 0.22 | 0.40 | 0.21 | SNP |
| chr6_32605078_A_G | 32605078 | 3.0 | 2.0 | 4.4 | 4.99E-08 | 0.21 | 0.39 | 0.19 | SNP |
| chr6_32605696_G_T | 32605696 | 2.9 | 2.0 | 4.3 | 5.56E-08 | 0.23 | 0.40 | 0.21 | SNP |
| chr6_32559213_A_T | 32559213 | 3.0 | 2.0 | 4.5 | 5.59E-08 | 0.20 | 0.38 | 0.19 | SNP |
| chr6_32608478_T_C | 32608478 | 2.9 | 2.0 | 4.2 | 6.40E-08 | 0.22 | 0.39 | 0.20 | SNP |
| chr6_32605178_T_C | 32605178 | 2.8 | 1.9 | 4.2 | 8.70E-08 | 0.22 | 0.40 | 0.21 | SNP |
| chr6_32487677_T_C | 32487677 | 3.3 | 2.1 | 5.1 | 9.24E-08 | 0.15 | 0.32 | 0.14 | SNP |
| chr6_32628148_C_T | 32628148 | 3.0 | 2.0 | 4.4 | 9.29E-08 | 0.15 | 0.31 | 0.14 | SNP |
| chr6_32603798_A_T | 32603798 | 2.8 | 1.9 | 4.1 | 1.00E-07 | 0.22 | 0.40 | 0.20 | SNP |
| chr6_32629259_T_C | 32629259 | 2.9 | 1.9 | 4.2 | 1.14E-07 | 0.16 | 0.31 | 0.14 | SNP |
| chr6_32629403_C_T | 32629403 | 2.8 | 1.9 | 4.1 | 1.18E-07 | 0.15 | 0.31 | 0.14 | SNP |
| chr6_32610401_G_A | 32610401 | 2.9 | 2.0 | 4.3 | 1.18E-07 | 0.22 | 0.39 | 0.20 | SNP |
| chr6_32607126_A_G | 32607126 | 2.8 | 1.9 | 4.0 | 1.34E-07 | 0.22 | 0.40 | 0.21 | SNP |
| chr6_32559702_A_G | 32559702 | 3.0 | 2.0 | 4.6 | 1.42E-07 | 0.21 | 0.38 | 0.19 | SNP |
| chr6_32551700_T_G | 32551700 | 3.0 | 2.0 | 4.6 | 2.43E-07 | 0.16 | 0.33 | 0.14 | SNP |
| chr6_32608286_T_C | 32608286 | 2.9 | 1.9 | 4.4 | 2.69E-07 | 0.21 | 0.40 | 0.19 | SNP |
| chr6_32559710_C_T | 32559710 | 2.9 | 1.9 | 4.3 | 4.18E-07 | 0.20 | 0.37 | 0.19 | SNP |
| chr6_32551665_A_G | 32551665 | 2.7 | 1.8 | 4.0 | 5.56E-07 | 0.19 | 0.36 | 0.18 | SNP |
| chr6_32605132_T_C | 32605132 | 2.6 | 1.8 | 3.7 | 6.50E-07 | 0.27 | 0.44 | 0.25 | SNP |
| chr6_32611467_A_C | 32611467 | 2.8 | 1.9 | 4.1 | 6.74E-07 | 0.19 | 0.35 | 0.17 | SNP |
| chr6_32628913_G_T | 32628913 | 2.7 | 1.8 | 4.0 | 8.79E-07 | 0.18 | 0.33 | 0.16 | SNP |
| chr6_32628571_C_T | 32628571 | 2.3 | 1.6 | 3.2 | 1.79E-06 | 0.23 | 0.37 | 0.21 | SNP |
| xHLA_2field_DQB1_03_02 | 32627244 | 2.6 | 1.8 | 3.9 | 1.90E-06 | 0.15 | 0.31 | 0.14 | HLA (xHLA) |
| chr6_32607031_G_T | 32607031 | 2.4 | 1.7 | 3.4 | 2.28E-06 | 0.31 | 0.49 | 0.30 | SNP |
| chr6_32559708_G_A | 32559708 | 2.4 | 1.7 | 3.5 | 2.44E-06 | 0.27 | 0.44 | 0.25 | SNP |
| chr6_32369488_G_A | 32369488 | 2.5 | 1.7 | 3.6 | 3.79E-06 | 0.09 | 0.17 | 0.08 | SNP |
| chr6_32627480_G_A | 32627480 | 2.4 | 1.6 | 3.4 | 3.82E-06 | 0.38 | 0.53 | 0.37 | SNP |
| chr6_32636521_G_A | 32636521 | 2.3 | 1.6 | 3.3 | 5.17E-06 | 0.17 | 0.31 | 0.16 | SNP |
| chr6_32604931_C_A | 32604931 | 2.2 | 1.6 | 3.1 | 5.25E-06 | 0.30 | 0.47 | 0.28 | SNP |
| chr6_32559124_T_C | 32559124 | 2.3 | 1.6 | 3.3 | 5.35E-06 | 0.28 | 0.44 | 0.26 | SNP |
| chr6_32608467_G_A | 32608467 | 2.2 | 1.6 | 3.1 | 5.72E-06 | 0.29 | 0.45 | 0.27 | SNP |
| chr6_32612326_C_T | 32612326 | 2.2 | 1.6 | 3.1 | 5.74E-06 | 0.29 | 0.45 | 0.27 | SNP |
| chr6_32559673_T_G | 32559673 | 2.2 | 1.6 | 3.1 | 5.85E-06 | 0.31 | 0.46 | 0.30 | SNP |
| chr6_32612279_T_C | 32612279 | 2.2 | 1.6 | 3.2 | 6.31E-06 | 0.29 | 0.45 | 0.27 | SNP |
| chr6_32612241_A_T | 32612241 | 2.2 | 1.6 | 3.1 | 6.61E-06 | 0.29 | 0.45 | 0.27 | SNP |
| chr6_32633971_A_G | 32633971 | 2.2 | 1.5 | 3.0 | 6.85E-06 | 0.19 | 0.34 | 0.18 | SNP |
| chr6_32376517_C_T | 32376517 | 2.4 | 1.6 | 3.6 | 6.89E-06 | 0.08 | 0.17 | 0.08 | SNP |
| chr6_32612251_T_G | 32612251 | 2.2 | 1.6 | 3.2 | 7.05E-06 | 0.29 | 0.45 | 0.27 | SNP |
| chr6_32629500_C_A | 32629500 | 2.2 | 1.6 | 3.1 | 7.28E-06 | 0.19 | 0.34 | 0.18 | SNP |
| chr6_32604394_T_C | 32604394 | 2.2 | 1.6 | 3.1 | 7.65E-06 | 0.29 | 0.46 | 0.28 | SNP |
| chr6_32604108_T_C | 32604108 | 2.2 | 1.6 | 3.1 | 7.68E-06 | 0.29 | 0.46 | 0.28 | SNP |
| chr6_32608537_A_C | 32608537 | 2.2 | 1.6 | 3.1 | 8.33E-06 | 0.29 | 0.46 | 0.27 | SNP |
| chr6_32609456_C_T | 32609456 | 2.2 | 1.6 | 3.1 | 8.48E-06 | 0.29 | 0.46 | 0.28 | SNP |
| chr6_32605724_A_G | 32605724 | 2.2 | 1.5 | 3.1 | 8.49E-06 | 0.29 | 0.45 | 0.27 | SNP |
| chr6_32604259_C_T | 32604259 | 2.2 | 1.6 | 3.1 | 8.75E-06 | 0.29 | 0.45 | 0.28 | SNP |
| chr6_32610008_C_T | 32610008 | 2.2 | 1.5 | 3.1 | 8.97E-06 | 0.29 | 0.45 | 0.28 | SNP |
| chr6_32628283_G_A | 32628283 | 2.2 | 1.6 | 3.2 | 9.19E-06 | 0.22 | 0.38 | 0.21 | SNP |
| chr6_32075563_T_C | 32075563 | 0.5 | 0.4 | 0.7 | 9.47E-06 | 0.48 | 0.40 | 0.49 | SNP |
| chr6_32608483_T_C | 32608483 | 2.2 | 1.5 | 3.1 | 9.52E-06 | 0.29 | 0.45 | 0.27 | SNP |
| chr6_32628195_A_G | 32628195 | 3.2 | 1.9 | 5.4 | 9.77E-06 | 0.03 | 0.10 | 0.03 | SNP |
| chr6_32628605_T_C | 32628605 | 2.1 | 1.5 | 3.0 | 1.02E-05 | 0.23 | 0.37 | 0.22 | SNP |
| xHLA_2field_C_07_01 | 31236526 | 2.9 | 1.8 | 4.5 | 1.09E-05 | 0.14 | 0.19 | 0.14 | HLA (xHLA) |
| chr6_32559212_A_G | 32559212 | 2.2 | 1.5 | 3.1 | 1.18E-05 | 0.28 | 0.44 | 0.27 | SNP |
| chr6_32559714_G_A | 32559714 | 0.5 | 0.3 | 0.6 | 1.19E-05 | 0.45 | 0.30 | 0.47 | SNP |
| chr6_32611971_G_C | 32611971 | 2.2 | 1.6 | 3.2 | 1.24E-05 | 0.28 | 0.42 | 0.26 | SNP |
| chr6_32609216_T_G | 32609216 | 0.4 | 0.3 | 0.6 | 1.27E-05 | 0.50 | 0.63 | 0.48 | SNP |
| chr6_32607047_T_G | 32607047 | 2.2 | 1.5 | 3.1 | 1.28E-05 | 0.31 | 0.48 | 0.30 | SNP |
| chr6_32608473_G_A | 32608473 | 2.2 | 1.5 | 3.1 | 1.31E-05 | 0.29 | 0.45 | 0.28 | SNP |
| chr6_32628506_C_A | 32628506 | 2.1 | 1.5 | 3.0 | 1.32E-05 | 0.23 | 0.37 | 0.21 | SNP |
| chr6_32608844_A_G | 32608844 | 2.2 | 1.5 | 3.1 | 1.32E-05 | 0.28 | 0.44 | 0.26 | SNP |
| chr6_32627818_C_A | 32627818 | 2.2 | 1.5 | 3.0 | 1.42E-05 | 0.20 | 0.34 | 0.19 | SNP |
| chr6_32606616_A_T | 32606616 | 2.4 | 1.6 | 3.5 | 1.43E-05 | 0.28 | 0.42 | 0.26 | SNP |
| chr6_32606942_G_A | 32606942 | 2.1 | 1.5 | 3.0 | 1.43E-05 | 0.30 | 0.46 | 0.28 | SNP |
| chr6_32604151_C_T | 32604151 | 2.1 | 1.5 | 3.0 | 1.52E-05 | 0.29 | 0.45 | 0.28 | SNP |
| chr6_32611723_T_G | 32611723 | 0.4 | 0.3 | 0.6 | 1.53E-05 | 0.48 | 0.34 | 0.49 | SNP |
| chr6_32628765_C_T | 32628765 | 2.0 | 1.5 | 2.7 | 1.56E-05 | 0.39 | 0.53 | 0.38 | SNP |
| chr6_32604124_A_G | 32604124 | 0.5 | 0.3 | 0.7 | 1.59E-05 | 0.49 | 0.63 | 0.48 | SNP |
| chr6_32550947_A_G | 32550947 | 2.2 | 1.5 | 3.2 | 1.62E-05 | 0.22 | 0.39 | 0.20 | SNP |
| chr6_32559709_C_T | 32559709 | 0.5 | 0.3 | 0.7 | 1.66E-05 | 0.45 | 0.30 | 0.46 | SNP |
| chr6_32610403_C_A | 32610403 | 2.2 | 1.5 | 3.1 | 1.68E-05 | 0.29 | 0.44 | 0.28 | SNP |
| chr6_32636221_G_T | 32636221 | 0.4 | 0.3 | 0.6 | 1.71E-05 | 0.46 | 0.32 | 0.47 | SNP |
| chr6_32604152_A_G | 32604152 | 2.1 | 1.5 | 3.0 | 1.76E-05 | 0.45 | 0.60 | 0.44 | SNP |
| chr6_32635293_C_G | 32635293 | 2.1 | 1.5 | 2.9 | 1.84E-05 | 0.23 | 0.38 | 0.22 | SNP |
| chr6_32605101_A_G | 32605101 | 2.1 | 1.5 | 3.0 | 1.89E-05 | 0.30 | 0.46 | 0.28 | SNP |
| chr6_32604059_A_G | 32604059 | 2.1 | 1.5 | 3.0 | 1.98E-05 | 0.29 | 0.45 | 0.28 | SNP |
| chr6_32559575_A_G | 32559575 | 2.4 | 1.6 | 3.5 | 2.06E-05 | 0.20 | 0.34 | 0.19 | SNP |
| chr6_32609286_T_C | 32609286 | 0.5 | 0.3 | 0.7 | 2.06E-05 | 0.48 | 0.63 | 0.47 | SNP |
| chr6_32629066_T_C | 32629066 | 2.1 | 1.5 | 2.9 | 2.08E-05 | 0.23 | 0.37 | 0.22 | SNP |
| chr6_32606732_G_A | 32606732 | 2.1 | 1.5 | 2.9 | 2.09E-05 | 0.30 | 0.46 | 0.28 | SNP |
| chr6_32441150_C_T | 32441150 | 2.1 | 1.5 | 3.0 | 2.12E-05 | 0.29 | 0.45 | 0.28 | SNP |
| chr6_32520664_G_A | 32520664 | 2.1 | 1.5 | 3.0 | 2.12E-05 | 0.31 | 0.47 | 0.29 | SNP |
| chr6_32628953_G_C | 32628953 | 0.4 | 0.3 | 0.6 | 2.15E-05 | 0.49 | 0.36 | 0.50 | SNP |
| chr6_32635248_A_T | 32635248 | 2.1 | 1.5 | 2.9 | 2.25E-05 | 0.23 | 0.36 | 0.21 | SNP |
| chr6_32611243_C_T | 32611243 | 2.1 | 1.5 | 3.0 | 2.27E-05 | 0.28 | 0.43 | 0.27 | SNP |
| chr6_32628617_A_C | 32628617 | 2.0 | 1.5 | 2.9 | 2.31E-05 | 0.23 | 0.37 | 0.22 | SNP |
| chr6_32520621_G_A | 32520621 | 2.1 | 1.5 | 3.0 | 2.37E-05 | 0.31 | 0.47 | 0.29 | SNP |
| chr6_32612050_T_A | 32612050 | 0.5 | 0.3 | 0.7 | 2.39E-05 | 0.47 | 0.35 | 0.49 | SNP |
| chr6_32605336_G_A | 32605336 | 2.1 | 1.5 | 2.9 | 2.41E-05 | 0.30 | 0.46 | 0.28 | SNP |
| chr6_32635230_G_A | 32635230 | 2.1 | 1.5 | 2.9 | 2.45E-05 | 0.23 | 0.36 | 0.22 | SNP |
| chr6_32605418_G_A | 32605418 | 2.1 | 1.5 | 2.9 | 2.51E-05 | 0.29 | 0.45 | 0.28 | SNP |
| chr6_32605274_C_T | 32605274 | 0.5 | 0.3 | 0.7 | 2.61E-05 | 0.46 | 0.32 | 0.47 | SNP |
| chr6_32605419_G_C | 32605419 | 2.1 | 1.5 | 3.0 | 2.63E-05 | 0.30 | 0.46 | 0.28 | SNP |
| chr6_32634834_T_C | 32634834 | 0.5 | 0.3 | 0.7 | 2.72E-05 | 0.46 | 0.32 | 0.47 | SNP |
| chr6_32521591_C_T | 32521591 | 2.1 | 1.5 | 3.0 | 2.75E-05 | 0.32 | 0.47 | 0.30 | SNP |
| chr6_32628967_T_C | 32628967 | 0.5 | 0.3 | 0.7 | 2.81E-05 | 0.49 | 0.36 | 0.51 | SNP |
| chr6_32612166_C_T | 32612166 | 2.1 | 1.5 | 3.0 | 2.86E-05 | 0.29 | 0.44 | 0.27 | SNP |
| chr6_32629297_C_T | 32629297 | 0.5 | 0.3 | 0.7 | 2.95E-05 | 0.45 | 0.31 | 0.46 | SNP |
| chr6_32520639_G_A | 32520639 | 2.1 | 1.5 | 3.0 | 2.96E-05 | 0.31 | 0.47 | 0.29 | SNP |
| chr6_32605646_T_G | 32605646 | 2.1 | 1.5 | 2.9 | 3.05E-05 | 0.30 | 0.46 | 0.29 | SNP |
| chr6_32609207_T_C | 32609207 | 0.5 | 0.3 | 0.7 | 3.09E-05 | 0.49 | 0.63 | 0.48 | SNP |
| chr6_32606052_T_G | 32606052 | 2.1 | 1.5 | 2.9 | 3.13E-05 | 0.30 | 0.46 | 0.29 | SNP |
| chr6_32612161_C_A | 32612161 | 2.1 | 1.5 | 3.0 | 3.13E-05 | 0.29 | 0.44 | 0.27 | SNP |
| chr6_32635266_T_G | 32635266 | 2.0 | 1.5 | 2.9 | 3.15E-05 | 0.23 | 0.36 | 0.21 | SNP |
| chr6_32611725_G_A | 32611725 | 0.5 | 0.3 | 0.7 | 3.19E-05 | 0.47 | 0.34 | 0.49 | SNP |
| chr6_32611260_A_G | 32611260 | 2.1 | 1.5 | 3.0 | 3.28E-05 | 0.28 | 0.44 | 0.27 | SNP |
| chr6_32608856_T_C | 32608856 | 2.1 | 1.5 | 3.0 | 3.36E-05 | 0.28 | 0.43 | 0.27 | SNP |
| chr6_32605759_A_T | 32605759 | 0.5 | 0.3 | 0.7 | 3.44E-05 | 0.46 | 0.33 | 0.47 | SNP |
| chr6_32634556_G_A | 32634556 | 2.0 | 1.5 | 2.8 | 3.49E-05 | 0.24 | 0.38 | 0.23 | SNP |
| chr6_32612397_A_G | 32612397 | 2.1 | 1.5 | 3.1 | 3.53E-05 | 0.46 | 0.63 | 0.45 | SNP |
| chr6_32604000_T_G | 32604000 | 0.5 | 0.3 | 0.7 | 3.54E-05 | 0.46 | 0.33 | 0.47 | SNP |
| chr6_32629269_C_T | 32629269 | 2.0 | 1.5 | 2.8 | 3.79E-05 | 0.23 | 0.36 | 0.22 | SNP |
| chr6_32628760_C_A | 32628760 | 2.0 | 1.4 | 2.8 | 3.83E-05 | 0.23 | 0.37 | 0.22 | SNP |
| chr6_32629802_G_A | 32629802 | 0.5 | 0.3 | 0.7 | 3.85E-05 | 0.46 | 0.32 | 0.47 | SNP |
| chr6_32634103_C_T | 32634103 | 0.5 | 0.3 | 0.7 | 3.92E-05 | 0.46 | 0.32 | 0.47 | SNP |
| chr6_32635974_A_G | 32635974 | 2.0 | 1.4 | 2.7 | 4.06E-05 | 0.24 | 0.38 | 0.22 | SNP |
| chr6_32603968_G_A | 32603968 | 0.5 | 0.3 | 0.7 | 4.17E-05 | 0.46 | 0.33 | 0.47 | SNP |
| chr6_32636434_G_A | 32636434 | 0.5 | 0.3 | 0.7 | 4.24E-05 | 0.46 | 0.33 | 0.47 | SNP |
| chr6_32628439_G_A | 32628439 | 1.9 | 1.4 | 2.6 | 4.24E-05 | 0.40 | 0.53 | 0.39 | SNP |
| chr6_32634007_A_G | 32634007 | 2.0 | 1.4 | 2.7 | 4.38E-05 | 0.24 | 0.38 | 0.22 | SNP |
| chr6_32634838_C_A | 32634838 | 0.5 | 0.3 | 0.7 | 4.38E-05 | 0.46 | 0.33 | 0.47 | SNP |
| chr6_32629639_T_C | 32629639 | 2.0 | 1.4 | 2.7 | 4.38E-05 | 0.24 | 0.38 | 0.22 | SNP |
| chr6_32629314_A_G | 32629314 | 0.5 | 0.3 | 0.7 | 4.41E-05 | 0.48 | 0.34 | 0.49 | SNP |
| chr6_32634057_C_T | 32634057 | 2.0 | 1.4 | 2.7 | 4.43E-05 | 0.24 | 0.38 | 0.22 | SNP |
| chr6_32636351_T_C | 32636351 | 2.0 | 1.4 | 2.8 | 4.48E-05 | 0.23 | 0.37 | 0.22 | SNP |
| chr6_32627535_T_G | 32627535 | 0.5 | 0.3 | 0.7 | 4.49E-05 | 0.46 | 0.32 | 0.47 | SNP |
| chr6_32636154_C_G | 32636154 | 2.0 | 1.4 | 2.7 | 4.62E-05 | 0.23 | 0.37 | 0.22 | SNP |
| chr6_32604372_A_G | 32604372 | 0.5 | 0.3 | 0.7 | 4.66E-05 | 0.47 | 0.33 | 0.48 | SNP |
| chr6_32634094_T_C | 32634094 | 2.0 | 1.4 | 2.7 | 4.72E-05 | 0.24 | 0.38 | 0.22 | SNP |
| chr6_32611760_G_T | 32611760 | 2.1 | 1.5 | 3.0 | 4.83E-05 | 0.29 | 0.44 | 0.27 | SNP |
| chr6_32634010_A_G | 32634010 | 0.4 | 0.3 | 0.6 | 4.91E-05 | 0.31 | 0.22 | 0.32 | SNP |
| chr6_32633899_T_C | 32633899 | 2.0 | 1.4 | 2.7 | 4.96E-05 | 0.24 | 0.38 | 0.23 | SNP |
| chr6_32627600_T_G | 32627600 | 0.5 | 0.3 | 0.7 | 5.15E-05 | 0.45 | 0.32 | 0.47 | SNP |
| chr6_32634425_C_T | 32634425 | 2.0 | 1.4 | 2.7 | 5.16E-05 | 0.24 | 0.38 | 0.22 | SNP |
| chr6_32611234_C_T | 32611234 | 2.1 | 1.4 | 2.9 | 5.22E-05 | 0.28 | 0.43 | 0.27 | SNP |
| chr6_32628189_A_G | 32628189 | 2.1 | 1.5 | 3.0 | 5.24E-05 | 0.20 | 0.34 | 0.19 | SNP |
| chr6_32634545_C_A | 32634545 | 2.0 | 1.4 | 2.7 | 5.28E-05 | 0.24 | 0.38 | 0.22 | SNP |
| chr6_32634755_C_T | 32634755 | 2.0 | 1.4 | 2.7 | 5.29E-05 | 0.24 | 0.38 | 0.23 | SNP |
| chr6_32550931_C_A | 32550931 | 2.1 | 1.5 | 3.0 | 5.33E-05 | 0.23 | 0.40 | 0.21 | SNP |
| chr6_32634098_C_G | 32634098 | 2.0 | 1.4 | 2.7 | 5.36E-05 | 0.24 | 0.38 | 0.23 | SNP |
| chr6_32520460_C_T | 32520460 | 2.0 | 1.4 | 2.9 | 5.50E-05 | 0.31 | 0.47 | 0.30 | SNP |
| chr6_32605754_A_T | 32605754 | 0.5 | 0.3 | 0.7 | 5.69E-05 | 0.46 | 0.33 | 0.47 | SNP |
| chr6_32629320_G_A | 32629320 | 0.4 | 0.3 | 0.6 | 5.73E-05 | 0.33 | 0.24 | 0.34 | SNP |

| | | | | | | | | | |
|---|---|---|---|---|---|---|---|---|---|
| chr6_32559045_T_C | 32559045 | 0.5 | 0.4 | 0.7 | 5.83E-05 | 0.43 | 0.30 | 0.44 | SNP |
| chr6_32550819_C_A | 32550819 | 2.0 | 1.4 | 2.9 | 5.87E-05 | 0.28 | 0.44 | 0.27 | SNP |
| chr6_32634506_C_T | 32634506 | 1.9 | 1.4 | 2.7 | 5.91E-05 | 0.24 | 0.38 | 0.23 | SNP |
| chr6_32634646_T_C | 32634646 | 1.9 | 1.4 | 2.7 | 6.01E-05 | 0.24 | 0.38 | 0.22 | SNP |
| chr6_32604538_G_A | 32604538 | 2.1 | 1.5 | 3.1 | 6.03E-05 | 0.32 | 0.47 | 0.31 | SNP |
| chr6_32634137_A_C | 32634137 | 1.9 | 1.4 | 2.7 | 6.13E-05 | 0.24 | 0.38 | 0.22 | SNP |
| chr6_32633636_G_A | 32633636 | 1.9 | 1.4 | 2.7 | 6.19E-05 | 0.24 | 0.38 | 0.23 | SNP |
| chr6_32441008_C_T | 32441008 | 2.0 | 1.4 | 2.9 | 6.21E-05 | 0.31 | 0.46 | 0.29 | SNP |
| chr6_32611229_T_G | 32611229 | 0.5 | 0.3 | 0.7 | 6.22E-05 | 0.46 | 0.34 | 0.48 | SNP |
| chr6_32634091_G_C | 32634091 | 1.9 | 1.4 | 2.7 | 6.23E-05 | 0.24 | 0.37 | 0.22 | SNP |
| chr6_32634387_T_C | 32634387 | 0.4 | 0.3 | 0.7 | 6.23E-05 | 0.31 | 0.22 | 0.32 | SNP |
| chr6_32628784_A_C | 32628784 | 0.5 | 0.3 | 0.7 | 6.25E-05 | 0.48 | 0.35 | 0.49 | SNP |
| chr6_32612111_G_A | 32612111 | 2.0 | 1.4 | 2.9 | 6.26E-05 | 0.29 | 0.43 | 0.27 | SNP |
| chr6_32634090_T_C | 32634090 | 1.9 | 1.4 | 2.7 | 6.33E-05 | 0.24 | 0.37 | 0.22 | SNP |
| chr6_32628633_T_C | 32628633 | 2.0 | 1.4 | 2.7 | 6.35E-05 | 0.23 | 0.37 | 0.22 | SNP |
| chr6_32610704_C_T | 32610704 | 0.4 | 0.3 | 0.7 | 6.36E-05 | 0.33 | 0.24 | 0.34 | SNP |
| chr6_32635226_G_T | 32635226 | 0.5 | 0.3 | 0.7 | 6.37E-05 | 0.47 | 0.34 | 0.48 | SNP |
| chr6_32627494_T_C | 32627494 | 2.0 | 1.4 | 2.7 | 6.45E-05 | 0.24 | 0.39 | 0.23 | SNP |
| chr6_32635172_C_T | 32635172 | 1.9 | 1.4 | 2.7 | 6.61E-05 | 0.24 | 0.38 | 0.23 | SNP |
| chr6_32609126_C_T | 32609126 | 0.5 | 0.3 | 0.7 | 6.61E-05 | 0.47 | 0.34 | 0.48 | SNP |
| chr6_32612104_C_T | 32612104 | 2.0 | 1.4 | 2.9 | 6.68E-05 | 0.29 | 0.43 | 0.27 | SNP |
| chr6_32627777_G_C | 32627777 | 1.9 | 1.4 | 2.7 | 6.83E-05 | 0.24 | 0.38 | 0.22 | SNP |
| chr6_32603487_C_T | 32603487 | 0.5 | 0.3 | 0.7 | 6.90E-05 | 0.46 | 0.33 | 0.47 | SNP |
| chr6_32604564_C_T | 32604564 | 2.1 | 1.4 | 2.9 | 6.90E-05 | 0.32 | 0.47 | 0.31 | SNP |
| chr6_32605920_A_G | 32605920 | 0.5 | 0.3 | 0.7 | 6.94E-05 | 0.46 | 0.33 | 0.47 | SNP |
| chr6_32636217_G_A | 32636217 | 2.0 | 1.4 | 2.7 | 6.99E-05 | 0.23 | 0.36 | 0.22 | SNP |
| chr6_32605943_A_G | 32605943 | 0.5 | 0.3 | 0.7 | 6.99E-05 | 0.46 | 0.33 | 0.47 | SNP |
| chr6_32629755_G_A | 32629755 | 1.9 | 1.4 | 2.7 | 7.01E-05 | 0.24 | 0.37 | 0.22 | SNP |
| chr6_32610968_A_G | 32610968 | 2.1 | 1.5 | 3.0 | 7.13E-05 | 0.26 | 0.40 | 0.25 | SNP |
| chr6_32607008_T_G | 32607008 | 0.5 | 0.3 | 0.7 | 7.20E-05 | 0.46 | 0.33 | 0.47 | SNP |
| chr6_32610769_C_A | 32610769 | 0.5 | 0.3 | 0.7 | 7.29E-05 | 0.34 | 0.25 | 0.34 | SNP |
| chr6_32636443_A_C | 32636443 | 1.9 | 1.4 | 2.7 | 7.35E-05 | 0.23 | 0.37 | 0.22 | SNP |
| chr6_32608880_G_A | 32608880 | 2.0 | 1.4 | 2.9 | 7.35E-05 | 0.33 | 0.47 | 0.31 | SNP |
| chr6_32634485_C_A | 32634485 | 0.4 | 0.3 | 0.7 | 7.42E-05 | 0.32 | 0.23 | 0.33 | SNP |
| chr6_32612008_T_C | 32612008 | 2.0 | 1.4 | 2.9 | 7.43E-05 | 0.28 | 0.43 | 0.27 | SNP |
| chr6_32634967_T_A | 32634967 | 1.9 | 1.4 | 2.7 | 7.57E-05 | 0.24 | 0.38 | 0.23 | SNP |
| chr6_32629543_T_C | 32629543 | 1.9 | 1.4 | 2.7 | 7.63E-05 | 0.24 | 0.38 | 0.23 | SNP |
| chr6_32606176_T_A | 32606176 | 0.5 | 0.3 | 0.7 | 7.69E-05 | 0.46 | 0.33 | 0.47 | SNP |
| chr6_32629530_C_T | 32629530 | 1.9 | 1.4 | 2.7 | 7.77E-05 | 0.24 | 0.38 | 0.23 | SNP |
| chr6_32628787_C_T | 32628787 | 2.0 | 1.4 | 2.7 | 8.10E-05 | 0.23 | 0.37 | 0.22 | SNP |
| chr6_32610059_G_T | 32610059 | 0.5 | 0.3 | 0.7 | 8.15E-05 | 0.46 | 0.33 | 0.47 | SNP |
| chr6_32628030_G_A | 32628030 | 1.9 | 1.4 | 2.6 | 8.27E-05 | 0.42 | 0.55 | 0.41 | SNP |
| chr6_32608417_G_A | 32608417 | 2.0 | 1.4 | 2.8 | 8.39E-05 | 0.29 | 0.44 | 0.27 | SNP |
| chr6_32636138_A_G | 32636138 | 1.9 | 1.4 | 2.7 | 8.57E-05 | 0.23 | 0.36 | 0.22 | SNP |
| chr6_32611964_C_T | 32611964 | 2.1 | 1.4 | 3.0 | 8.62E-05 | 0.28 | 0.42 | 0.27 | SNP |
| chr6_32628916_G_A | 32628916 | 1.9 | 1.4 | 2.7 | 8.62E-05 | 0.23 | 0.37 | 0.22 | SNP |
| chr6_32606217_G_A | 32606217 | 2.0 | 1.4 | 2.8 | 8.89E-05 | 0.30 | 0.45 | 0.29 | SNP |
| chr6_32628776_A_C | 32628776 | 0.5 | 0.3 | 0.7 | 9.05E-05 | 0.48 | 0.35 | 0.49 | SNP |
| chr6_32612041_A_G | 32612041 | 2.0 | 1.4 | 2.9 | 9.20E-05 | 0.29 | 0.43 | 0.27 | SNP |
| chr6_32612410_T_C | 32612410 | 0.4 | 0.3 | 0.7 | 9.23E-05 | 0.45 | 0.31 | 0.46 | SNP |
| chr6_32633767_G_T | 32633767 | 1.9 | 1.4 | 2.6 | 9.25E-05 | 0.24 | 0.37 | 0.22 | SNP |
| chr6_32634593_G_C | 32634593 | 1.9 | 1.4 | 2.7 | 9.38E-05 | 0.23 | 0.37 | 0.22 | SNP |
| chr6_32627489_G_A | 32627489 | 0.5 | 0.3 | 0.7 | 9.45E-05 | 0.44 | 0.31 | 0.45 | SNP |
| chr6_32612003_T_C | 32612003 | 0.5 | 0.3 | 0.7 | 9.47E-05 | 0.48 | 0.35 | 0.49 | SNP |
| chr6_32633781_C_T | 32633781 | 1.9 | 1.4 | 2.6 | 9.54E-05 | 0.24 | 0.37 | 0.23 | SNP |
| chr6_32609105_A_G | 32609105 | 0.5 | 0.3 | 0.7 | 9.55E-05 | 0.47 | 0.34 | 0.48 | SNP |
| chr6_32629328_G_A | 32629328 | 2.0 | 1.4 | 2.8 | 9.82E-05 | 0.23 | 0.35 | 0.22 | SNP |
| chr6_32627833_A_G | 32627833 | 1.8 | 1.4 | 2.5 | 9.97E-05 | 0.41 | 0.53 | 0.40 | SNP |
| chr6_32605705_T_G | 32605705 | 0.5 | 0.3 | 0.7 | 0.00010 | 0.46 | 0.34 | 0.47 | SNP |
| chr6_32609514_T_C | 32609514 | 0.5 | 0.3 | 0.7 | 0.00010 | 0.46 | 0.34 | 0.47 | SNP |
| chr6_32636063_G_A | 32636063 | 1.9 | 1.4 | 2.6 | 0.00010 | 0.24 | 0.37 | 0.22 | SNP |
| chr6_32605708_A_G | 32605708 | 0.5 | 0.3 | 0.7 | 0.00010 | 0.46 | 0.34 | 0.47 | SNP |
| chr6_32627714_G_T | 32627714 | 0.5 | 0.3 | 0.7 | 0.00010 | 0.45 | 0.33 | 0.47 | SNP |
| chr6_32627485_C_T | 32627485 | 0.5 | 0.3 | 0.7 | 0.00010 | 0.44 | 0.31 | 0.45 | SNP |
| chr6_32611650_G_A | 32611650 | 2.0 | 1.4 | 2.9 | 0.00010 | 0.28 | 0.42 | 0.27 | SNP |
| chr6_32629549_G_A | 32629549 | 1.9 | 1.4 | 2.6 | 0.00011 | 0.24 | 0.37 | 0.23 | SNP |
| chr6_32606085_T_A | 32606085 | 0.5 | 0.3 | 0.7 | 0.00011 | 0.46 | 0.33 | 0.47 | SNP |
| chr6_32611958_C_G | 32611958 | 2.1 | 1.4 | 3.0 | 0.00011 | 0.28 | 0.41 | 0.27 | SNP |
| chr6_32629531_A_G | 32629531 | 1.9 | 1.4 | 2.6 | 0.00011 | 0.24 | 0.37 | 0.23 | SNP |
| chr6_32520774_C_T | 32520774 | 2.0 | 1.4 | 2.9 | 0.00011 | 0.30 | 0.45 | 0.29 | SNP |
| chr6_32610994_C_A | 32610994 | 2.0 | 1.4 | 2.9 | 0.00011 | 0.26 | 0.40 | 0.25 | SNP |
| chr6_32627635_G_T | 32627635 | 0.5 | 0.3 | 0.7 | 0.00011 | 0.45 | 0.33 | 0.46 | SNP |
| xHLA_2field_B_08_01 | 31321652 | 3.8 | 1.9 | 7.6 | 0.00012 | 0.12 | 0.14 | 0.11 | HLA (xHLA) |
| chr6_32634355_G_A | 32634355 | 1.9 | 1.4 | 2.6 | 0.00012 | 0.24 | 0.37 | 0.22 | SNP |
| chr6_32501301_G_C | 32501301 | 0.5 | 0.3 | 0.7 | 0.00012 | 0.39 | 0.26 | 0.40 | SNP |
| chr6_32610997_C_G | 32610997 | 2.0 | 1.4 | 2.9 | 0.00012 | 0.26 | 0.40 | 0.25 | SNP |
| chr6_32627747_G_A | 32627747 | 0.5 | 0.3 | 0.7 | 0.00013 | 0.31 | 0.22 | 0.32 | SNP |
| chr6_32501296_G_A | 32501296 | 0.5 | 0.3 | 0.7 | 0.00013 | 0.39 | 0.26 | 0.40 | SNP |
| chr6_32608809_T_C | 32608809 | 2.0 | 1.4 | 2.9 | 0.00013 | 0.26 | 0.41 | 0.25 | SNP |
| chr6_32501459_T_C | 32501459 | 0.5 | 0.3 | 0.7 | 0.00013 | 0.39 | 0.26 | 0.41 | SNP |
| chr6_32636172_C_T | 32636172 | 1.9 | 1.4 | 2.6 | 0.00013 | 0.23 | 0.37 | 0.22 | SNP |
| chr6_32634139_C_T | 32634139 | 1.9 | 1.4 | 2.6 | 0.00013 | 0.24 | 0.37 | 0.23 | SNP |
| chr6_32633617_A_G | 32633617 | 0.5 | 0.3 | 0.7 | 0.00014 | 0.45 | 0.33 | 0.46 | SNP |
| chr6_32627517_G_T | 32627517 | 0.5 | 0.3 | 0.7 | 0.00014 | 0.46 | 0.33 | 0.47 | SNP |
| chr6_32627646_G_A | 32627646 | 0.5 | 0.3 | 0.7 | 0.00014 | 0.45 | 0.33 | 0.47 | SNP |
| chr6_32629330_C_T | 32629330 | 1.9 | 1.4 | 2.7 | 0.00014 | 0.23 | 0.35 | 0.22 | SNP |
| chr6_32605295_C_T | 32605295 | 1.9 | 1.4 | 2.7 | 0.00014 | 0.32 | 0.47 | 0.30 | SNP |
| xHLA_2field_B_15_01 | 31321652 | 0.3 | 0.2 | 0.6 | 0.00014 | 0.10 | 0.06 | 0.10 | HLA (xHLA) |
| chr6_31323416_G_C | 31323416 | 3.7 | 1.9 | 7.1 | 0.00015 | 0.12 | 0.14 | 0.12 | SNP |
| chr6_32607193_T_C | 32607193 | 2.0 | 1.4 | 2.9 | 0.00015 | 0.33 | 0.48 | 0.32 | SNP |
| chr6_32627561_C_T | 32627561 | 0.5 | 0.3 | 0.7 | 0.00015 | 0.32 | 0.24 | 0.33 | SNP |
| chr6_32611769_G_C | 32611769 | 2.0 | 1.4 | 2.9 | 0.00015 | 0.30 | 0.44 | 0.29 | SNP |
| chr6_32633959_T_C | 32633959 | 1.9 | 1.4 | 2.6 | 0.00016 | 0.24 | 0.37 | 0.23 | SNP |
| chr6_32609312_C_A | 32609312 | 0.5 | 0.3 | 0.7 | 0.00016 | 0.47 | 0.34 | 0.48 | SNP |
| chr6_32633909_T_A | 32633909 | 0.5 | 0.3 | 0.7 | 0.00016 | 0.33 | 0.25 | 0.34 | SNP |
| chr6_32611968_T_G | 32611968 | 0.5 | 0.3 | 0.7 | 0.00016 | 0.48 | 0.37 | 0.49 | SNP |
| chr6_32634150_A_C | 32634150 | 1.9 | 1.4 | 2.6 | 0.00016 | 0.24 | 0.37 | 0.23 | SNP |
| chr6_32628751_T_C | 32628751 | 0.5 | 0.3 | 0.7 | 0.00016 | 0.48 | 0.35 | 0.49 | SNP |
| chr6_32611931_T_A | 32611931 | 0.5 | 0.3 | 0.7 | 0.00016 | 0.47 | 0.35 | 0.48 | SNP |
| chr6_32633906_A_C | 32633906 | 0.5 | 0.3 | 0.7 | 0.00016 | 0.33 | 0.25 | 0.34 | SNP |
| chr6_32611265_C_T | 32611265 | 2.0 | 1.4 | 2.8 | 0.00016 | 0.28 | 0.42 | 0.27 | SNP |
| chr6_32629422_T_C | 32629422 | 1.9 | 1.4 | 2.6 | 0.00016 | 0.23 | 0.37 | 0.22 | SNP |
| chr6_31321267_A_C | 31321267 | 3.9 | 1.9 | 8.0 | 0.00016 | 0.12 | 0.13 | 0.12 | SNP |
| chr6_32629582_T_C | 32629582 | 0.5 | 0.3 | 0.7 | 0.00017 | 0.31 | 0.23 | 0.32 | SNP |
| chr6_32636040_C_T | 32636040 | 0.5 | 0.3 | 0.7 | 0.00017 | 0.32 | 0.23 | 0.32 | SNP |
| chr6_32629398_G_C | 32629398 | 1.9 | 1.4 | 2.6 | 0.00018 | 0.24 | 0.37 | 0.23 | SNP |
| chr6_32609453_C_T | 32609453 | 2.0 | 1.4 | 2.8 | 0.00018 | 0.33 | 0.48 | 0.32 | SNP |
| chr6_32629347_T_C | 32629347 | 2.0 | 1.4 | 2.8 | 0.00018 | 0.36 | 0.51 | 0.35 | SNP |
| chr6_32635033_T_G | 32635033 | 1.9 | 1.3 | 2.6 | 0.00018 | 0.24 | 0.37 | 0.22 | SNP |
| chr6_32610860_G_T | 32610860 | 2.0 | 1.4 | 2.8 | 0.00018 | 0.28 | 0.43 | 0.27 | SNP |
| chr6_32604642_A_G | 32604642 | 2.0 | 1.4 | 2.9 | 0.00021 | 0.28 | 0.42 | 0.26 | SNP |
| chr6_32796310_A_G | 32796310 | 2.8 | 1.6 | 4.7 | 0.00022 | 0.03 | 0.08 | 0.03 | SNP |
| chr6_32501311_C_T | 32501311 | 0.5 | 0.4 | 0.7 | 0.00023 | 0.39 | 0.26 | 0.40 | SNP |
| chr6_32628737_G_A | 32628737 | 0.5 | 0.4 | 0.7 | 0.00023 | 0.48 | 0.35 | 0.49 | SNP |
| chr6_32606701_A_G | 32606701 | 0.4 | 0.3 | 0.7 | 0.00024 | 0.17 | 0.08 | 0.18 | SNP |
| chr6_32611450_T_G | 32611450 | 0.5 | 0.3 | 0.7 | 0.00024 | 0.48 | 0.36 | 0.49 | SNP |
| chr6_31326703_T_C | 31326703 | 3.8 | 1.8 | 7.6 | 0.00026 | 0.12 | 0.13 | 0.12 | SNP |
| chr6_31322487_G_A | 31322487 | 0.4 | 0.2 | 0.6 | 0.00026 | 0.10 | 0.07 | 0.11 | SNP |
| chr6_32633665_T_C | 32633665 | 0.5 | 0.3 | 0.7 | 0.00027 | 0.32 | 0.23 | 0.32 | SNP |
| chr6_32553418_T_G | 32553418 | 0.5 | 0.4 | 0.7 | 0.00028 | 0.41 | 0.28 | 0.42 | SNP |
| chr6_32611034_T_A | 32611034 | 1.9 | 1.3 | 2.7 | 0.00028 | 0.27 | 0.40 | 0.26 | SNP |
| chr6_32611873_C_T | 32611873 | 0.5 | 0.4 | 0.7 | 0.00028 | 0.47 | 0.35 | 0.48 | SNP |
| chr6_32611874_T_G | 32611874 | 0.5 | 0.4 | 0.7 | 0.00028 | 0.47 | 0.35 | 0.48 | SNP |
| chr6_32520595_G_A | 32520595 | 0.5 | 0.4 | 0.7 | 0.00029 | 0.38 | 0.26 | 0.39 | SNP |
| chr6_32629467_G_A | 32629467 | 1.9 | 1.3 | 2.7 | 0.00029 | 0.36 | 0.51 | 0.35 | SNP |
| chr6_32628736_T_C | 32628736 | 0.5 | 0.4 | 0.7 | 0.00029 | 0.48 | 0.35 | 0.49 | SNP |
| chr6_32604644_G_A | 32604644 | 2.0 | 1.4 | 2.8 | 0.00030 | 0.28 | 0.42 | 0.27 | SNP |
| chr6_32520337_A_G | 32520337 | 2.0 | 1.4 | 2.9 | 0.00031 | 0.31 | 0.47 | 0.30 | SNP |
| chr6_32629409_G_A | 32629409 | 0.5 | 0.3 | 0.7 | 0.00031 | 0.32 | 0.23 | 0.33 | SNP |
| chr6_31378335_G_C | 31378335 | 0.4 | 0.2 | 0.6 | 0.00031 | 0.10 | 0.07 | 0.11 | SNP |
| chr6_31323506_C_T | 31323506 | 3.5 | 1.7 | 6.8 | 0.00031 | 0.12 | 0.13 | 0.12 | SNP |
| chr6_32608820_T_C | 32608820 | 0.5 | 0.4 | 0.7 | 0.00032 | 0.47 | 0.35 | 0.48 | SNP |
| chr6_32629331_A_G | 32629331 | 1.9 | 1.3 | 2.8 | 0.00033 | 0.36 | 0.50 | 0.34 | SNP |
| chr6_32558183_G_T | 32558183 | 1.8 | 1.3 | 2.6 | 0.00034 | 0.49 | 0.62 | 0.48 | SNP |
| chr6_32184705_A_G | 32184705 | 1.8 | 1.3 | 2.4 | 0.00034 | 0.33 | 0.40 | 0.33 | SNP |
| chr6_32521596_T_G | 32521596 | 2.0 | 1.4 | 2.8 | 0.00035 | 0.35 | 0.49 | 0.34 | SNP |
| chr6_32636494_C_T | 32636494 | 0.5 | 0.4 | 0.7 | 0.00035 | 0.43 | 0.31 | 0.44 | SNP |
| chr6_32610934_A_G | 32610934 | 2.0 | 1.4 | 2.9 | 0.00036 | 0.30 | 0.43 | 0.29 | SNP |
| chr6_32526540_A_G | 32526540 | 0.5 | 0.4 | 0.8 | 0.00036 | 0.41 | 0.29 | 0.42 | SNP |
| chr6_32634261_C_T | 32634261 | 1.8 | 1.3 | 2.5 | 0.00036 | 0.24 | 0.36 | 0.23 | SNP |
| chr6_32634262_T_G | 32634262 | 1.8 | 1.3 | 2.5 | 0.00037 | 0.24 | 0.36 | 0.23 | SNP |
| chr6_31326074_A_T | 31326074 | 3.7 | 1.8 | 7.5 | 0.00038 | 0.12 | 0.13 | 0.11 | SNP |
| chr6_32609427_C_T | 32609427 | 0.5 | 0.4 | 0.7 | 0.00039 | 0.46 | 0.34 | 0.47 | SNP |
| chr6_32607174_C_T | 32607174 | 1.9 | 1.3 | 2.7 | 0.00039 | 0.34 | 0.48 | 0.33 | SNP |
| chr6_32628941_G_C | 32628941 | 1.8 | 1.3 | 2.6 | 0.00040 | 0.23 | 0.35 | 0.22 | SNP |
| chr6_32340070_C_T | 32340070 | 1.8 | 1.3 | 2.5 | 0.00040 | 0.39 | 0.52 | 0.38 | SNP |
| chr6_32635949_G_A | 32635949 | 1.8 | 1.3 | 2.4 | 0.00041 | 0.28 | 0.41 | 0.27 | SNP |
| chr6_32143991_G_C | 32143991 | 1.9 | 1.3 | 2.8 | 0.00042 | 0.16 | 0.21 | 0.15 | SNP |
| chr6_32551568_C_G | 32551568 | 0.5 | 0.4 | 0.7 | 0.00043 | 0.39 | 0.26 | 0.40 | SNP |
| chr6_32627456_A_G | 32627456 | 0.5 | 0.3 | 0.7 | 0.00045 | 0.36 | 0.27 | 0.37 | SNP |
| chr6_31322790_T_A | 31322790 | 3.3 | 1.7 | 6.5 | 0.00046 | 0.12 | 0.14 | 0.12 | SNP |
| chr6_31322694_T_C | 31322694 | 0.4 | 0.3 | 0.7 | 0.00047 | 0.12 | 0.09 | 0.12 | SNP |
| chr6_32520665_A_C | 32520665 | 0.5 | 0.4 | 0.8 | 0.00047 | 0.37 | 0.26 | 0.39 | SNP |
| chr6_32632777_C_T | 32632777 | 0.3 | 0.2 | 0.6 | 0.00053 | 0.15 | 0.06 | 0.15 | SNP |
| chr6_32636591_G_A | 32636591 | 0.5 | 0.3 | 0.7 | 0.00053 | 0.46 | 0.32 | 0.47 | SNP |

| | | | | | | | | | |
|---|---|---|---|---|---|---|---|---|---|
| chr6_31473746_G_A | 31473746 | 3.6 | 1.7 | 7.4 | 0.00054 | 0.12 | 0.14 | 0.12 | SNP |
| chr6_32603098_T_C | 32603098 | 1.7 | 1.3 | 2.4 | 0.00058 | 0.48 | 0.63 | 0.46 | SNP |
| chr6_32609358_C_T | 32609358 | 1.9 | 1.3 | 2.7 | 0.00059 | 0.29 | 0.42 | 0.27 | SNP |
| chr6_31558702_C_T | 31558702 | 3.6 | 1.7 | 7.6 | 0.00061 | 0.12 | 0.13 | 0.12 | SNP |
| chr6_31322703_C_T | 31322703 | 0.4 | 0.2 | 0.7 | 0.00066 | 0.10 | 0.08 | 0.11 | SNP |
| chr6_32629509_A_C | 32629509 | 1.9 | 1.3 | 2.6 | 0.00067 | 0.37 | 0.50 | 0.36 | SNP |
| chr6_32636385_A_T | 32636385 | 1.7 | 1.2 | 2.3 | 0.00068 | 0.37 | 0.47 | 0.36 | SNP |
| chr6_32603412_T_C | 32603412 | 1.7 | 1.3 | 2.4 | 0.00069 | 0.48 | 0.63 | 0.46 | SNP |
| chr6_31324955_C_T | 31324955 | 0.4 | 0.2 | 0.7 | 0.00069 | 0.10 | 0.07 | 0.10 | SNP |
| chr6_32782030_A_C | 32782030 | 1.8 | 1.3 | 2.5 | 0.00071 | 0.18 | 0.22 | 0.18 | SNP |
| chr6_32603029_T_C | 32603029 | 0.5 | 0.4 | 0.8 | 0.00073 | 0.28 | 0.15 | 0.29 | SNP |
| chr6_32609380_C_T | 32609380 | 1.9 | 1.3 | 2.8 | 0.00073 | 0.35 | 0.47 | 0.34 | SNP |
| chr6_32636364_G_A | 32636364 | 1.7 | 1.2 | 2.3 | 0.00074 | 0.37 | 0.47 | 0.37 | SNP |
| chr6_32631039_G_A | 32631039 | 0.5 | 0.3 | 0.7 | 0.00075 | 0.37 | 0.29 | 0.38 | SNP |
| chr6_31325684_C_A | 31325684 | 0.4 | 0.2 | 0.7 | 0.00078 | 0.10 | 0.08 | 0.11 | SNP |
| chr6_31325795_G_T | 31325795 | 0.4 | 0.2 | 0.7 | 0.00078 | 0.10 | 0.08 | 0.11 | SNP |
| chr6_32370684_A_G | 32370684 | 1.8 | 1.3 | 2.6 | 0.00079 | 0.27 | 0.40 | 0.26 | SNP |
| chr6_31323725_T_C | 31323725 | 0.4 | 0.2 | 0.7 | 0.00081 | 0.10 | 0.08 | 0.11 | SNP |
| chr6_32078373_A_C | 32078373 | 1.9 | 1.3 | 2.8 | 0.00082 | 0.11 | 0.16 | 0.11 | SNP |
| chr6_31323721_G_C | 31323721 | 0.4 | 0.2 | 0.7 | 0.00082 | 0.10 | 0.08 | 0.11 | SNP |
| chr6_29907740_A_G | 29907740 | 0.6 | 0.4 | 0.8 | 0.00083 | 0.47 | 0.42 | 0.47 | SNP |
| chr6_31326292_A_G | 31326292 | 0.4 | 0.3 | 0.7 | 0.00084 | 0.10 | 0.08 | 0.11 | SNP |
| chr6_31370367_G_A | 31370367 | 0.4 | 0.2 | 0.7 | 0.00085 | 0.10 | 0.08 | 0.11 | SNP |
| chr6_32501245_A_G | 32501245 | 0.5 | 0.3 | 0.7 | 0.00085 | 0.19 | 0.10 | 0.20 | SNP |
| chr6_31370329_C_A | 31370329 | 0.4 | 0.3 | 0.7 | 0.00088 | 0.10 | 0.08 | 0.10 | SNP |
| chr6_32629535_C_A | 32629535 | 1.8 | 1.3 | 2.6 | 0.00089 | 0.37 | 0.50 | 0.35 | SNP |
| chr6_32521951_A_C | 32521951 | 0.5 | 0.4 | 0.8 | 0.00090 | 0.38 | 0.26 | 0.39 | SNP |
| chr6_32500925_A_C | 32500925 | 0.5 | 0.3 | 0.7 | 0.00090 | 0.19 | 0.10 | 0.20 | SNP |
| chr6_32340654_G_A | 32340654 | 1.8 | 1.3 | 2.6 | 0.00092 | 0.27 | 0.41 | 0.26 | SNP |
| chr6_32370616_T_C | 32370616 | 1.8 | 1.3 | 2.6 | 0.00093 | 0.27 | 0.40 | 0.26 | SNP |
| chr6_32520465_A_G | 32520465 | 0.6 | 0.4 | 0.8 | 0.00093 | 0.38 | 0.26 | 0.39 | SNP |
| chr6_31324599_T_G | 31324599 | 0.4 | 0.3 | 0.7 | 0.00094 | 0.14 | 0.10 | 0.14 | SNP |
| chr6_32603488_A_G | 32603488 | 0.5 | 0.4 | 0.8 | 0.00094 | 0.28 | 0.15 | 0.29 | SNP |
| chr6_31239227_T_C | 31239227 | 2.1 | 1.4 | 3.3 | 0.00095 | 0.38 | 0.45 | 0.37 | SNP |
| chr6_32123639_G_A | 32123639 | 1.7 | 1.2 | 2.4 | 0.00095 | 0.16 | 0.21 | 0.16 | SNP |
| chr6_32603355_T_C | 32603355 | 0.5 | 0.4 | 0.8 | 0.00096 | 0.28 | 0.15 | 0.29 | SNP |
| chr6_32611504_C_T | 32611504 | 0.5 | 0.4 | 0.8 | 0.00101 | 0.48 | 0.37 | 0.49 | SNP |
| chr6_32364356_A_G | 32364356 | 1.8 | 1.3 | 2.6 | 0.00102 | 0.27 | 0.40 | 0.26 | SNP |
| chr6_32636289_T_C | 32636289 | 1.8 | 1.3 | 2.5 | 0.00109 | 0.23 | 0.34 | 0.22 | SNP |
| chr6_32376746_C_T | 32376746 | 1.8 | 1.3 | 2.5 | 0.00110 | 0.27 | 0.41 | 0.26 | SNP |
| chr6_32628845_G_A | 32628845 | 1.8 | 1.3 | 2.6 | 0.00111 | 0.36 | 0.50 | 0.35 | SNP |
| chr6_32341719_C_T | 32341719 | 1.8 | 1.3 | 2.6 | 0.00111 | 0.27 | 0.40 | 0.26 | SNP |
| chr6_31239868_C_G | 31239868 | 1.9 | 1.3 | 2.8 | 0.00113 | 0.36 | 0.41 | 0.35 | SNP |
| chr6_32405821_T_C | 32405821 | 1.8 | 1.3 | 2.7 | 0.00115 | 0.24 | 0.37 | 0.23 | SNP |
| chr6_29893960_T_C | 29893960 | 1.8 | 1.3 | 2.6 | 0.00115 | 0.20 | 0.25 | 0.19 | SNP |
| chr6_32629764_C_T | 32629764 | 1.8 | 1.3 | 2.5 | 0.00119 | 0.37 | 0.50 | 0.36 | SNP |
| chr6_32629859_G_A | 32629859 | 0.6 | 0.4 | 0.8 | 0.00123 | 0.49 | 0.39 | 0.50 | SNP |
| chr6_31514247_A_G | 31514247 | 3.4 | 1.6 | 7.2 | 0.00124 | 0.12 | 0.13 | 0.12 | SNP |
| chr6_31378117_C_T | 31378117 | 0.4 | 0.2 | 0.7 | 0.00133 | 0.09 | 0.06 | 0.09 | SNP |
| chr6_32611510_C_G | 32611510 | 0.6 | 0.4 | 0.8 | 0.00133 | 0.47 | 0.36 | 0.48 | SNP |
| chr6_31366295_C_A | 31366295 | 0.4 | 0.3 | 0.7 | 0.00137 | 0.11 | 0.08 | 0.11 | SNP |
| chr6_32521117_T_C | 32521117 | 0.6 | 0.4 | 0.8 | 0.00142 | 0.49 | 0.35 | 0.51 | SNP |
| chr6_32374622_A_T | 32374622 | 1.8 | 1.2 | 2.5 | 0.00143 | 0.28 | 0.41 | 0.26 | SNP |
| chr6_32611466_A_G | 32611466 | 1.8 | 1.3 | 2.6 | 0.00145 | 0.26 | 0.40 | 0.25 | SNP |
| chr6_32341473_T_C | 32341473 | 1.8 | 1.2 | 2.5 | 0.00147 | 0.28 | 0.41 | 0.26 | SNP |
| chr6_31320849_T_C | 31320849 | 0.4 | 0.3 | 0.7 | 0.00149 | 0.10 | 0.07 | 0.10 | SNP |
| chr6_31324348_C_T | 31324348 | 0.4 | 0.2 | 0.7 | 0.00150 | 0.11 | 0.07 | 0.11 | SNP |
| chr6_31324891_C_T | 31324891 | 0.4 | 0.2 | 0.7 | 0.00150 | 0.10 | 0.07 | 0.11 | SNP |
| chr6_31240060_G_A | 31240060 | 2.0 | 1.3 | 3.0 | 0.00150 | 0.33 | 0.37 | 0.32 | SNP |
| chr6_32611474_G_A | 32611474 | 1.8 | 1.2 | 2.6 | 0.00151 | 0.26 | 0.40 | 0.25 | SNP |
| chr6_32410941_C_T | 32410941 | 0.6 | 0.4 | 0.8 | 0.00152 | 0.50 | 0.60 | 0.49 | SNP |
| chr6_32341318_A_G | 32341318 | 1.7 | 1.2 | 2.5 | 0.00152 | 0.28 | 0.41 | 0.26 | SNP |
| chr6_32375371_A_T | 32375371 | 1.7 | 1.2 | 2.5 | 0.00156 | 0.29 | 0.41 | 0.27 | SNP |
| chr6_32363844_C_T | 32363844 | 1.8 | 1.2 | 2.5 | 0.00156 | 0.27 | 0.40 | 0.26 | SNP |
| chr6_32375352_A_C | 32375352 | 1.7 | 1.2 | 2.5 | 0.00158 | 0.29 | 0.41 | 0.27 | SNP |
| chr6_32629400_A_G | 32629400 | 1.8 | 1.2 | 2.5 | 0.00159 | 0.37 | 0.50 | 0.35 | SNP |
| chr6_32636280_G_T | 32636280 | 1.7 | 1.2 | 2.5 | 0.00161 | 0.23 | 0.34 | 0.22 | SNP |
| chr6_32635713_C_T | 32635713 | 0.6 | 0.4 | 0.8 | 0.00166 | 0.48 | 0.37 | 0.49 | SNP |
| chr6_31241664_G_A | 31241664 | 2.2 | 1.3 | 3.6 | 0.00166 | 0.14 | 0.17 | 0.14 | SNP |
| chr6_32611472_G_A | 32611472 | 0.6 | 0.4 | 0.8 | 0.00167 | 0.47 | 0.36 | 0.48 | SNP |
| chr6_32375374_G_A | 32375374 | 1.7 | 1.2 | 2.5 | 0.00169 | 0.29 | 0.41 | 0.28 | SNP |
| chr6_32605583_A_G | 32605583 | 1.6 | 1.2 | 2.2 | 0.00182 | 0.38 | 0.50 | 0.37 | SNP |
| chr6_31324448_G_A | 31324448 | 0.4 | 0.2 | 0.7 | 0.00185 | 0.11 | 0.08 | 0.11 | SNP |
| chr6_31429927_T_C | 31429927 | 2.1 | 1.3 | 3.3 | 0.00187 | 0.17 | 0.19 | 0.16 | SNP |
| chr6_31129310_C_T | 31129310 | 3.0 | 1.5 | 5.8 | 0.00193 | 0.12 | 0.13 | 0.12 | SNP |
| chr6_31721033_G_A | 31721033 | 3.6 | 1.6 | 8.1 | 0.00198 | 0.12 | 0.13 | 0.12 | SNP |
| chr6_32609094_C_T | 32609094 | 1.7 | 1.2 | 2.4 | 0.00206 | 0.35 | 0.47 | 0.34 | SNP |
| chr6_31241311_A_G | 31241311 | 2.2 | 1.3 | 3.5 | 0.00207 | 0.14 | 0.17 | 0.14 | SNP |
| chr6_29892137_A_G | 29892137 | 1.6 | 1.2 | 2.2 | 0.00208 | 0.23 | 0.29 | 0.23 | SNP |
| chr6_31243895_T_C | 31243895 | 1.7 | 1.2 | 2.4 | 0.00213 | 0.39 | 0.44 | 0.38 | SNP |
| chr6_32611152_G_A | 32611152 | 1.7 | 1.2 | 2.4 | 0.00214 | 0.21 | 0.34 | 0.20 | SNP |
| chr6_32797488_T_C | 32797488 | 1.6 | 1.2 | 2.2 | 0.00216 | 0.30 | 0.40 | 0.30 | SNP |
| chr6_31243830_A_G | 31243830 | 1.7 | 1.2 | 2.3 | 0.00217 | 0.39 | 0.44 | 0.38 | SNP |
| chr6_31465047_G_T | 31465047 | 3.2 | 1.5 | 6.5 | 0.00218 | 0.12 | 0.13 | 0.12 | SNP |
| chr6_31243979_A_T | 31243979 | 1.9 | 1.3 | 2.9 | 0.00223 | 0.32 | 0.37 | 0.32 | SNP |
| chr6_32376348_G_A | 32376348 | 1.8 | 1.2 | 2.6 | 0.00223 | 0.24 | 0.37 | 0.23 | SNP |
| chr6_29910358_G_C | 29910358 | 1.6 | 1.2 | 2.2 | 0.00232 | 0.46 | 0.51 | 0.45 | SNP |
| chr6_32375973_C_A | 32375973 | 1.8 | 1.2 | 2.5 | 0.00232 | 0.24 | 0.37 | 0.23 | SNP |
| chr6_32374131_C_G | 32374131 | 1.7 | 1.2 | 2.5 | 0.00232 | 0.27 | 0.40 | 0.26 | SNP |
| chr6_31708463_C_A | 31708463 | 3.5 | 1.6 | 7.9 | 0.00235 | 0.12 | 0.13 | 0.12 | SNP |
| chr6_31321883_G_A | 31321883 | 0.5 | 0.3 | 0.8 | 0.00236 | 0.14 | 0.10 | 0.14 | SNP |
| chr6_32375330_G_A | 32375330 | 1.7 | 1.2 | 2.4 | 0.00236 | 0.28 | 0.41 | 0.27 | SNP |
| chr6_31244021_A_G | 31244021 | 1.9 | 1.3 | 2.9 | 0.00239 | 0.32 | 0.36 | 0.31 | SNP |
| chr6_32376176_C_T | 32376176 | 1.7 | 1.2 | 2.4 | 0.00239 | 0.28 | 0.41 | 0.27 | SNP |
| chr6_32375695_G_A | 32375695 | 1.7 | 1.2 | 2.4 | 0.00239 | 0.28 | 0.41 | 0.27 | SNP |
| chr6_32411035_C_A | 32411035 | 0.6 | 0.4 | 0.8 | 0.00241 | 0.50 | 0.40 | 0.51 | SNP |
| chr6_32522698_G_T | 32522698 | 0.5 | 0.4 | 0.8 | 0.00245 | 0.41 | 0.32 | 0.42 | SNP |
| chr6_32611403_G_A | 32611403 | 1.7 | 1.2 | 2.5 | 0.00256 | 0.26 | 0.40 | 0.25 | SNP |
| chr6_32375424_G_A | 32375424 | 1.7 | 1.2 | 2.4 | 0.00256 | 0.29 | 0.41 | 0.28 | SNP |
| chr6_31379115_G_A | 31379115 | 0.6 | 0.4 | 0.8 | 0.00256 | 0.21 | 0.17 | 0.22 | SNP |
| chr6_32633816_C_G | 32633816 | 1.6 | 1.2 | 2.2 | 0.00263 | 0.37 | 0.46 | 0.36 | SNP |
| chr6_32555317_C_T | 32555317 | 0.4 | 0.2 | 0.7 | 0.00267 | 0.10 | 0.04 | 0.10 | SNP |
| chr6_32190969_C_T | 32190969 | 3.1 | 1.4 | 6.4 | 0.00269 | 0.01 | 0.04 | 0.01 | SNP |
| chr6_31675765_G_A | 31675765 | 1.7 | 1.2 | 2.4 | 0.00275 | 0.19 | 0.24 | 0.19 | SNP |
| chr6_31243846_G_T | 31243846 | 2.2 | 1.3 | 3.6 | 0.00276 | 0.14 | 0.16 | 0.14 | SNP |
| chr6_31430065_C_T | 31430065 | 3.1 | 1.5 | 6.6 | 0.00280 | 0.11 | 0.13 | 0.11 | SNP |
| chr6_31237048_G_A | 31237048 | 1.8 | 1.2 | 2.6 | 0.00280 | 0.34 | 0.39 | 0.34 | SNP |
| chr6_31246324_A_C | 31246324 | 2.2 | 1.3 | 3.5 | 0.00282 | 0.14 | 0.16 | 0.14 | SNP |
| chr6_32169574_T_C | 32169574 | 0.6 | 0.5 | 0.8 | 0.00289 | 0.29 | 0.24 | 0.30 | SNP |
| chr6_32627652_C_G | 32627652 | 1.7 | 1.2 | 2.4 | 0.00291 | 0.37 | 0.50 | 0.36 | SNP |
| chr6_32610349_C_A | 32610349 | 1.6 | 1.2 | 2.2 | 0.00294 | 0.44 | 0.54 | 0.43 | SNP |
| chr6_31636742_A_G | 31636742 | 3.3 | 1.5 | 7.2 | 0.00299 | 0.12 | 0.13 | 0.12 | SNP |
| chr6_32627795_A_G | 32627795 | 1.6 | 1.2 | 2.2 | 0.00301 | 0.47 | 0.57 | 0.46 | SNP |
| chr6_32628420_C_T | 32628420 | 1.7 | 1.2 | 2.5 | 0.00302 | 0.35 | 0.48 | 0.34 | SNP |
| chr6_29901806_C_T | 29901806 | 1.7 | 1.2 | 2.4 | 0.00303 | 0.22 | 0.26 | 0.21 | SNP |
| chr6_31243884_G_A | 31243884 | 1.7 | 1.2 | 2.5 | 0.00307 | 0.21 | 0.24 | 0.21 | SNP |
| chr6_31238029_C_T | 31238029 | 1.8 | 1.2 | 2.6 | 0.00309 | 0.36 | 0.40 | 0.35 | SNP |
| chr6_32376019_G_T | 32376019 | 0.4 | 0.2 | 0.7 | 0.00310 | 0.11 | 0.05 | 0.11 | SNP |
| chr6_29912188_T_C | 29912188 | 0.6 | 0.5 | 0.9 | 0.00310 | 0.43 | 0.40 | 0.43 | SNP |
| chr6_32611427_T_A | 32611427 | 1.8 | 1.2 | 2.5 | 0.00312 | 0.27 | 0.39 | 0.26 | SNP |
| chr6_31237233_G_A | 31237233 | 1.8 | 1.2 | 2.6 | 0.00317 | 0.36 | 0.40 | 0.35 | SNP |
| chr6_29896713_G_A | 29896713 | 1.6 | 1.2 | 2.2 | 0.00317 | 0.23 | 0.28 | 0.23 | SNP |
| chr6_31243971_G_A | 31243971 | 1.9 | 1.2 | 2.9 | 0.00326 | 0.32 | 0.36 | 0.32 | SNP |
| chr6_31633165_C_A | 31633165 | 1.6 | 1.2 | 2.2 | 0.00329 | 0.18 | 0.23 | 0.18 | SNP |
| chr6_32363816_T_C | 32363816 | 1.6 | 1.2 | 2.2 | 0.00331 | 0.43 | 0.53 | 0.42 | SNP |
| chr6_31705864_C_T | 31705864 | 3.3 | 1.5 | 7.1 | 0.00337 | 0.12 | 0.13 | 0.12 | SNP |
| chr6_31712196_G_A | 31712196 | 3.3 | 1.5 | 7.1 | 0.00337 | 0.12 | 0.13 | 0.12 | SNP |
| chr6_31725230_G_A | 31725230 | 3.3 | 1.5 | 7.1 | 0.00337 | 0.12 | 0.13 | 0.12 | SNP |
| chr6_31726794_T_C | 31726794 | 3.3 | 1.5 | 7.1 | 0.00337 | 0.12 | 0.13 | 0.12 | SNP |
| chr6_31727474_A_G | 31727474 | 3.3 | 1.5 | 7.1 | 0.00337 | 0.12 | 0.13 | 0.12 | SNP |
| chr6_32611418_A_C | 32611418 | 1.7 | 1.2 | 2.5 | 0.00343 | 0.27 | 0.39 | 0.26 | SNP |
| chr6_29945949_G_A | 29945949 | 2.1 | 1.3 | 3.4 | 0.00343 | 0.11 | 0.14 | 0.11 | SNP |
| chr6_31634193_A_G | 31634193 | 1.6 | 1.2 | 2.1 | 0.00349 | 0.38 | 0.43 | 0.38 | SNP |
| chr6_31725285_A_G | 31725285 | 3.2 | 1.5 | 7.1 | 0.00352 | 0.12 | 0.13 | 0.12 | SNP |
| chr6_32943151_C_T | 32943151 | 0.7 | 0.5 | 0.9 | 0.00355 | 0.41 | 0.33 | 0.41 | SNP |
| chr6_32603660_T_C | 32603660 | 1.6 | 1.2 | 2.1 | 0.00357 | 0.39 | 0.50 | 0.38 | SNP |
| chr6_32375280_C_G | 32375280 | 1.7 | 1.2 | 2.4 | 0.00364 | 0.29 | 0.41 | 0.27 | SNP |
| chr6_32942302_A_G | 32942302 | 0.7 | 0.5 | 0.9 | 0.00374 | 0.41 | 0.33 | 0.41 | SNP |
| chr6_31349922_C_T | 31349922 | 0.5 | 0.3 | 0.8 | 0.00374 | 0.12 | 0.10 | 0.12 | SNP |
| chr6_32628944_C_G | 32628944 | 1.7 | 1.2 | 2.5 | 0.00385 | 0.35 | 0.47 | 0.34 | SNP |
| chr6_31240041_T_C | 31240041 | 1.7 | 1.2 | 2.3 | 0.00388 | 0.41 | 0.44 | 0.41 | SNP |
| chr6_29909751_C_T | 29909751 | 1.5 | 1.1 | 2.0 | 0.00392 | 0.46 | 0.49 | 0.45 | SNP |
| chr6_32363955_T_C | 32363955 | 1.6 | 1.2 | 2.2 | 0.00394 | 0.43 | 0.53 | 0.43 | SNP |
| chr6_32164869_G_C | 32164869 | 0.6 | 0.4 | 0.9 | 0.00406 | 0.29 | 0.24 | 0.29 | SNP |
| chr6_29892732_G_A | 29892732 | 1.6 | 1.2 | 2.2 | 0.00434 | 0.20 | 0.25 | 0.20 | SNP |
| chr6_29896773_C_T | 29896773 | 1.6 | 1.2 | 2.2 | 0.00437 | 0.23 | 0.27 | 0.23 | SNP |
| chr6_32407068_G_A | 32407068 | 2.5 | 1.3 | 4.6 | 0.00438 | 0.02 | 0.05 | 0.02 | SNP |
| chr6_31430010_G_A | 31430010 | 2.9 | 1.4 | 6.0 | 0.00441 | 0.12 | 0.13 | 0.12 | SNP |
| chr6_31237405_T_C | 31237405 | 1.7 | 1.2 | 2.6 | 0.00441 | 0.34 | 0.38 | 0.34 | SNP |
| chr6_31765984_G_A | 31765984 | 3.4 | 1.5 | 8.0 | 0.00442 | 0.12 | 0.12 | 0.12 | SNP |
| chr6_29891850_A_G | 29891850 | 1.5 | 1.1 | 1.9 | 0.00451 | 0.42 | 0.46 | 0.42 | SNP |
| chr6_31239449_C_G | 31239449 | 2.0 | 1.2 | 3.1 | 0.00455 | 0.17 | 0.20 | 0.16 | SNP |
| chr6_29911019_T_C | 29911019 | 0.6 | 0.4 | 0.9 | 0.00455 | 0.44 | 0.40 | 0.45 | SNP |
| chr6_31240293_T_C | 31240293 | 1.7 | 1.2 | 2.5 | 0.00455 | 0.35 | 0.39 | 0.34 | SNP |
| chr6_30879987_C_T | 30879987 | 2.4 | 1.3 | 4.5 | 0.00455 | 0.11 | 0.13 | 0.11 | SNP |

| | | | | | | | | | |
|---|---|---|---|---|---|---|---|---|---|
| chr6_31627449_C_G | 31627449 | 1.6 | 1.1 | 2.2 | 0.00460 | 0.18 | 0.23 | 0.18 | SNP |
| chr6_31245796_C_T | 31245796 | 2.1 | 1.2 | 3.4 | 0.00472 | 0.14 | 0.16 | 0.14 | SNP |
| xHLA_2field_A_02_01 | 29910309 | 0.6 | 0.5 | 0.9 | 0.00476 | 0.35 | 0.33 | 0.35 | HLA (xHLA) |
| chr6_32153409_A_C | 32153409 | 1.6 | 1.2 | 2.3 | 0.00479 | 0.39 | 0.49 | 0.38 | SNP |
| chr6_31237230_G_A | 31237230 | 1.7 | 1.2 | 2.5 | 0.00481 | 0.35 | 0.39 | 0.35 | SNP |
| chr6_31237449_C_T | 31237449 | 1.8 | 1.2 | 2.7 | 0.00482 | 0.32 | 0.36 | 0.31 | SNP |
| chr6_32814244_A_T | 32814244 | 0.5 | 0.3 | 0.8 | 0.00487 | 0.11 | 0.05 | 0.11 | SNP |
| chr6_32813279_C_A | 32813279 | 0.5 | 0.3 | 0.8 | 0.00490 | 0.11 | 0.05 | 0.11 | SNP |
| chr6_31601513_A_G | 31601513 | 1.6 | 1.1 | 2.1 | 0.00504 | 0.39 | 0.42 | 0.38 | SNP |
| chr6_32610947_A_C | 32610947 | 0.3 | 0.1 | 0.6 | 0.00512 | 0.06 | 0.02 | 0.06 | SNP |
| chr6_32411833_C_T | 32411833 | 1.6 | 1.2 | 2.2 | 0.00517 | 0.40 | 0.52 | 0.38 | SNP |
| chr6_29909840_A_G | 29909840 | 1.5 | 1.1 | 1.9 | 0.00529 | 0.46 | 0.49 | 0.45 | SNP |
| chr6_32080383_C_T | 32080383 | 0.4 | 0.2 | 0.7 | 0.00532 | 0.08 | 0.03 | 0.09 | SNP |
| chr6_31238318_A_G | 31238318 | 1.7 | 1.2 | 2.5 | 0.00537 | 0.35 | 0.39 | 0.35 | SNP |
| chr6_32786882_T_C | 32786882 | 1.8 | 1.2 | 2.7 | 0.00538 | 0.07 | 0.13 | 0.06 | SNP |
| chr6_32409656_G_A | 32409656 | 1.6 | 1.1 | 2.2 | 0.00539 | 0.35 | 0.48 | 0.34 | SNP |
| chr6_31239855_G_C | 31239855 | 1.7 | 1.2 | 2.6 | 0.00539 | 0.36 | 0.41 | 0.35 | SNP |
| chr6_29909070_T_C | 29909070 | 0.7 | 0.5 | 0.9 | 0.00542 | 0.45 | 0.42 | 0.45 | SNP |
| chr6_31709778_A_G | 31709778 | 3.2 | 1.4 | 7.1 | 0.00544 | 0.12 | 0.12 | 0.12 | SNP |
| chr6_31473957_C_G | 31473957 | 2.8 | 1.3 | 5.6 | 0.00547 | 0.12 | 0.13 | 0.12 | SNP |
| chr6_31238613_G_A | 31238613 | 1.7 | 1.2 | 2.5 | 0.00552 | 0.35 | 0.39 | 0.34 | SNP |
| chr6_31237323_G_A | 31237323 | 1.7 | 1.2 | 2.5 | 0.00555 | 0.35 | 0.39 | 0.34 | SNP |
| chr6_31635710_T_C | 31635710 | 1.5 | 1.1 | 2.1 | 0.00556 | 0.20 | 0.25 | 0.20 | SNP |
| chr6_31921486_A_G | 31921486 | 3.0 | 1.3 | 6.3 | 0.00560 | 0.01 | 0.03 | 0.01 | SNP |
| chr6_32032421_C_T | 32032421 | 0.4 | 0.2 | 0.7 | 0.00566 | 0.11 | 0.05 | 0.12 | SNP |
| chr6_31237162_G_C | 31237162 | 1.7 | 1.2 | 2.5 | 0.00571 | 0.34 | 0.38 | 0.34 | SNP |
| chr6_31243859_C_T | 31243859 | 1.7 | 1.2 | 2.4 | 0.00574 | 0.21 | 0.24 | 0.21 | SNP |
| chr6_31237061_T_C | 31237061 | 1.7 | 1.2 | 2.4 | 0.00574 | 0.36 | 0.40 | 0.35 | SNP |
| chr6_32633819_A_G | 32633819 | 0.6 | 0.4 | 0.9 | 0.00575 | 0.50 | 0.59 | 0.49 | SNP |
| chr6_31601520_T_C | 31601520 | 1.6 | 1.1 | 2.3 | 0.00578 | 0.19 | 0.24 | 0.18 | SNP |
| chr6_31321268_T_G | 31321268 | 0.6 | 0.4 | 0.8 | 0.00580 | 0.20 | 0.15 | 0.20 | SNP |
| chr6_31237592_C_A | 31237592 | 1.7 | 1.2 | 2.5 | 0.00585 | 0.34 | 0.38 | 0.34 | SNP |
| chr6_29691019_G_A | 29691019 | 0.7 | 0.5 | 0.9 | 0.00586 | 0.44 | 0.40 | 0.44 | SNP |
| chr6_29896852_C_T | 29896852 | 1.6 | 1.1 | 2.2 | 0.00588 | 0.25 | 0.29 | 0.24 | SNP |
| chr6_31237124_C_T | 31237124 | 1.7 | 1.2 | 2.5 | 0.00589 | 0.34 | 0.38 | 0.34 | SNP |
| chr6_29796265_G_T | 29796265 | 2.5 | 1.3 | 4.6 | 0.00590 | 0.03 | 0.06 | 0.03 | SNP |
| chr6_29909559_A_G | 29909559 | 1.5 | 1.1 | 1.9 | 0.00602 | 0.46 | 0.49 | 0.45 | SNP |
| chr6_29909447_C_G | 29909447 | 1.5 | 1.1 | 1.9 | 0.00604 | 0.46 | 0.49 | 0.45 | SNP |
| chr6_32178773_T_C | 32178773 | 0.6 | 0.5 | 0.9 | 0.00618 | 0.48 | 0.41 | 0.48 | SNP |
| chr6_32410716_G_A | 32410716 | 1.6 | 1.1 | 2.2 | 0.00624 | 0.39 | 0.51 | 0.38 | SNP |
| chr6_32336299_A_G | 32336299 | 0.2 | 0.0 | 0.5 | 0.00626 | 0.05 | 0.01 | 0.05 | SNP |
| chr6_29795913_A_T | 29795913 | 2.5 | 1.2 | 4.6 | 0.00627 | 0.03 | 0.06 | 0.03 | SNP |
| chr6_31602489_T_C | 31602489 | 1.6 | 1.1 | 2.2 | 0.00629 | 0.18 | 0.23 | 0.18 | SNP |
| chr6_32603277_G_T | 32603277 | 0.3 | 0.1 | 0.6 | 0.00640 | 0.06 | 0.02 | 0.06 | SNP |
| chr6_29913881_T_C | 29913881 | 0.7 | 0.5 | 0.9 | 0.00640 | 0.40 | 0.38 | 0.40 | SNP |
| chr6_32609047_C_T | 32609047 | 0.3 | 0.1 | 0.6 | 0.00642 | 0.06 | 0.02 | 0.06 | SNP |
| chr6_32606406_A_G | 32606406 | 0.6 | 0.4 | 0.9 | 0.00644 | 0.38 | 0.29 | 0.39 | SNP |
| chr6_31237563_T_C | 31237563 | 1.7 | 1.2 | 2.5 | 0.00646 | 0.34 | 0.38 | 0.34 | SNP |
| chr6_32501522_A_G | 32501522 | 1.7 | 1.2 | 2.5 | 0.00646 | 0.23 | 0.30 | 0.23 | SNP |
| chr6_29909924_G_A | 29909924 | 0.7 | 0.5 | 0.9 | 0.00649 | 0.45 | 0.42 | 0.45 | SNP |
| chr6_32782387_G_A | 32782387 | 1.6 | 1.1 | 2.2 | 0.00650 | 0.21 | 0.24 | 0.21 | SNP |
| chr6_31237950_C_T | 31237950 | 1.7 | 1.2 | 2.5 | 0.00650 | 0.36 | 0.41 | 0.36 | SNP |
| chr6_29911198_T_C | 29911198 | 0.6 | 0.5 | 0.9 | 0.00651 | 0.41 | 0.38 | 0.41 | SNP |
| chr6_30855211_T_G | 30855211 | 2.3 | 1.3 | 4.3 | 0.00652 | 0.11 | 0.13 | 0.11 | SNP |
| chr6_32606682_C_G | 32606682 | 0.3 | 0.1 | 0.6 | 0.00654 | 0.06 | 0.02 | 0.06 | SNP |
| chr6_32089142_G_T | 32089142 | 0.3 | 0.1 | 0.7 | 0.00656 | 0.09 | 0.03 | 0.09 | SNP |
| chr6_32606691_T_G | 32606691 | 0.3 | 0.1 | 0.6 | 0.00659 | 0.06 | 0.02 | 0.06 | SNP |
| chr6_31245821_A_G | 31245821 | 2.0 | 1.2 | 3.3 | 0.00659 | 0.14 | 0.16 | 0.14 | SNP |
| chr6_32604166_A_G | 32604166 | 0.3 | 0.1 | 0.6 | 0.00661 | 0.06 | 0.02 | 0.06 | SNP |
| chr6_32339548_G_A | 32339548 | 1.6 | 1.1 | 2.1 | 0.00668 | 0.38 | 0.48 | 0.37 | SNP |
| chr6_29910378_C_T | 29910378 | 0.6 | 0.4 | 0.9 | 0.00672 | 0.35 | 0.32 | 0.35 | SNP |
| chr6_31325056_C_T | 31325056 | 2.0 | 1.2 | 3.2 | 0.00673 | 0.15 | 0.17 | 0.15 | SNP |
| chr6_32441129_G_A | 32441129 | 0.3 | 0.1 | 0.6 | 0.00675 | 0.06 | 0.02 | 0.06 | SNP |
| chr6_32609009_T_G | 32609009 | 0.3 | 0.1 | 0.6 | 0.00677 | 0.06 | 0.02 | 0.06 | SNP |
| chr6_31237333_A_T | 31237333 | 1.7 | 1.2 | 2.5 | 0.00677 | 0.35 | 0.39 | 0.34 | SNP |
| chr6_30857894_G_A | 30857894 | 2.3 | 1.2 | 4.2 | 0.00684 | 0.11 | 0.13 | 0.11 | SNP |
| chr6_30867527_G_A | 30867527 | 2.3 | 1.2 | 4.2 | 0.00684 | 0.11 | 0.13 | 0.11 | SNP |
| chr6_31238524_A_G | 31238524 | 1.7 | 1.2 | 2.5 | 0.00684 | 0.36 | 0.40 | 0.36 | SNP |
| chr6_31237353_C_T | 31237353 | 1.7 | 1.2 | 2.5 | 0.00689 | 0.34 | 0.38 | 0.34 | SNP |
| chr6_32609425_C_G | 32609425 | 0.3 | 0.1 | 0.6 | 0.00692 | 0.06 | 0.02 | 0.06 | SNP |
| chr6_32603896_T_C | 32603896 | 0.3 | 0.1 | 0.6 | 0.00699 | 0.06 | 0.02 | 0.06 | SNP |
| chr6_32608435_T_C | 32608435 | 0.3 | 0.1 | 0.6 | 0.00708 | 0.06 | 0.02 | 0.06 | SNP |
| chr6_32439696_T_G | 32439696 | 0.3 | 0.1 | 0.6 | 0.00709 | 0.06 | 0.02 | 0.06 | SNP |
| chr6_32781076_C_A | 32781076 | 0.4 | 0.2 | 0.8 | 0.00711 | 0.10 | 0.05 | 0.10 | SNP |
| chr6_29910892_A_G | 29910892 | 0.6 | 0.5 | 0.9 | 0.00718 | 0.44 | 0.40 | 0.44 | SNP |
| chr6_30515043_T_C | 30515043 | 2.1 | 1.2 | 3.6 | 0.00720 | 0.11 | 0.13 | 0.11 | SNP |
| chr6_31324595_C_G | 31324595 | 0.6 | 0.4 | 0.9 | 0.00727 | 0.31 | 0.23 | 0.31 | SNP |
| chr6_31320164_G_A | 31320164 | 1.6 | 1.1 | 2.3 | 0.00728 | 0.23 | 0.28 | 0.22 | SNP |
| chr6_31464992_G_A | 31464992 | 1.6 | 1.1 | 2.3 | 0.00738 | 0.29 | 0.34 | 0.28 | SNP |
| chr6_32441046_G_A | 32441046 | 0.3 | 0.1 | 0.6 | 0.00747 | 0.06 | 0.02 | 0.06 | SNP |
| chr6_31238372_G_C | 31238372 | 1.7 | 1.1 | 2.5 | 0.00749 | 0.35 | 0.38 | 0.34 | SNP |
| chr6_32006337_A_C | 32006337 | 0.6 | 0.4 | 0.9 | 0.00752 | 0.34 | 0.27 | 0.34 | SNP |
| chr6_32628606_C_A | 32628606 | 0.3 | 0.1 | 0.6 | 0.00759 | 0.05 | 0.02 | 0.06 | SNP |
| chr6_31616366_G_A | 31616366 | 1.5 | 1.1 | 2.0 | 0.00763 | 0.38 | 0.41 | 0.38 | SNP |
| chr6_32606376_T_C | 32606376 | 0.3 | 0.1 | 0.7 | 0.00765 | 0.06 | 0.02 | 0.06 | SNP |
| chr6_32187722_T_C | 32187722 | 1.5 | 1.1 | 2.1 | 0.00766 | 0.47 | 0.52 | 0.46 | SNP |
| chr6_31611840_C_A | 31611840 | 1.6 | 1.1 | 2.2 | 0.00767 | 0.18 | 0.22 | 0.17 | SNP |
| chr6_30229267_G_C | 30229267 | 2.2 | 1.2 | 3.8 | 0.00777 | 0.10 | 0.12 | 0.10 | SNP |
| chr6_29893982_C_T | 29893982 | 1.7 | 1.1 | 2.4 | 0.00781 | 0.19 | 0.22 | 0.19 | SNP |
| chr6_32611145_T_A | 32611145 | 1.6 | 1.1 | 2.2 | 0.00790 | 0.21 | 0.33 | 0.20 | SNP |
| chr6_32558284_T_C | 32558284 | 0.4 | 0.2 | 0.8 | 0.00793 | 0.08 | 0.04 | 0.08 | SNP |
| chr6_31236524_G_A | 31236524 | 1.7 | 1.1 | 2.5 | 0.00797 | 0.35 | 0.39 | 0.35 | SNP |
| chr6_32441696_C_T | 32441696 | 0.4 | 0.2 | 0.7 | 0.00801 | 0.07 | 0.03 | 0.07 | SNP |
| chr6_31085356_C_T | 31085356 | 2.5 | 1.2 | 4.8 | 0.00802 | 0.12 | 0.13 | 0.12 | SNP |
| chr6_32557962_A_G | 32557962 | 0.6 | 0.4 | 0.9 | 0.00805 | 0.27 | 0.20 | 0.28 | SNP |
| chr6_32442703_G_A | 32442703 | 0.3 | 0.1 | 0.7 | 0.00808 | 0.05 | 0.02 | 0.06 | SNP |
| chr6_32522703_T_G | 32522703 | 0.6 | 0.4 | 0.9 | 0.00814 | 0.40 | 0.32 | 0.41 | SNP |
| chr6_29911063_T_G | 29911063 | 0.6 | 0.5 | 0.9 | 0.00815 | 0.40 | 0.37 | 0.40 | SNP |
| chr6_31238297_T_C | 31238297 | 1.7 | 1.1 | 2.4 | 0.00831 | 0.35 | 0.38 | 0.34 | SNP |
| xHLA_2field_DRB1_13_02 | 32546552 | 0.3 | 0.1 | 0.7 | 0.00837 | 0.06 | 0.02 | 0.06 | HLA (xHLA) |
| chr6_30879636_C_T | 30879636 | 2.4 | 1.2 | 4.5 | 0.00841 | 0.11 | 0.13 | 0.11 | SNP |
| chr6_29911037_G_C | 29911037 | 0.6 | 0.5 | 0.9 | 0.00843 | 0.45 | 0.42 | 0.46 | SNP |
| chr6_32169621_T_A | 32169621 | 0.7 | 0.5 | 0.9 | 0.00843 | 0.47 | 0.41 | 0.48 | SNP |
| chr6_32406530_G_A | 32406530 | 1.6 | 1.1 | 2.2 | 0.00845 | 0.35 | 0.47 | 0.34 | SNP |
| chr6_32553662_A_G | 32553662 | 0.6 | 0.4 | 0.9 | 0.00855 | 0.39 | 0.30 | 0.40 | SNP |
| chr6_32193945_G_T | 32193945 | 0.6 | 0.4 | 0.9 | 0.00857 | 0.17 | 0.11 | 0.17 | SNP |
| chr6_32628843_C_T | 32628843 | 0.3 | 0.1 | 0.7 | 0.00869 | 0.05 | 0.02 | 0.06 | SNP |
| chr6_29853750_G_T | 29853750 | 1.7 | 1.1 | 2.6 | 0.00869 | 0.14 | 0.17 | 0.14 | SNP |
| chr6_32611373_A_G | 32611373 | 0.6 | 0.4 | 0.9 | 0.00870 | 0.50 | 0.39 | 0.51 | SNP |
| chr6_31464571_T_C | 31464571 | 1.6 | 1.1 | 2.3 | 0.00874 | 0.29 | 0.34 | 0.28 | SNP |
| chr6_31236854_T_C | 31236854 | 2.0 | 1.2 | 3.3 | 0.00875 | 0.14 | 0.16 | 0.14 | SNP |
| chr6_32408527_C_G | 32408527 | 1.5 | 1.1 | 2.1 | 0.00878 | 0.35 | 0.47 | 0.34 | SNP |
| chr6_32270222_C_A | 32270222 | 0.3 | 0.1 | 0.6 | 0.00883 | 0.05 | 0.01 | 0.05 | SNP |
| chr6_31236523_G_A | 31236523 | 1.7 | 1.1 | 2.4 | 0.00883 | 0.35 | 0.39 | 0.35 | SNP |
| chr6_32607224_C_T | 32607224 | 0.4 | 0.2 | 0.7 | 0.00884 | 0.10 | 0.04 | 0.11 | SNP |
| chr6_32629545_T_C | 32629545 | 0.3 | 0.1 | 0.7 | 0.00889 | 0.05 | 0.02 | 0.06 | SNP |
| chr6_32077380_C_T | 32077380 | 0.4 | 0.2 | 0.8 | 0.00894 | 0.08 | 0.03 | 0.09 | SNP |
| chr6_31237437_C_T | 31237437 | 1.7 | 1.1 | 2.4 | 0.00925 | 0.35 | 0.38 | 0.34 | SNP |
| chr6_29910986_G_A | 29910986 | 0.6 | 0.4 | 0.9 | 0.00929 | 0.35 | 0.32 | 0.35 | SNP |
| chr6_29912796_G_A | 29912796 | 0.7 | 0.5 | 0.9 | 0.00933 | 0.40 | 0.39 | 0.41 | SNP |
| chr6_32193449_T_C | 32193449 | 0.6 | 0.4 | 0.9 | 0.00933 | 0.16 | 0.11 | 0.17 | SNP |
| chr6_32785275_G_A | 32785275 | 0.5 | 0.3 | 0.8 | 0.00936 | 0.10 | 0.05 | 0.10 | SNP |
| chr6_29943281_G_A | 29943281 | 0.7 | 0.5 | 0.9 | 0.00947 | 0.35 | 0.33 | 0.35 | SNP |
| chr6_29901865_G_C | 29901865 | 1.5 | 1.1 | 2.0 | 0.00948 | 0.28 | 0.32 | 0.28 | SNP |
| chr6_32406807_G_A | 32406807 | 1.7 | 1.1 | 2.5 | 0.00959 | 0.37 | 0.53 | 0.36 | SNP |
| chr6_32607221_C_G | 32607221 | 0.4 | 0.2 | 0.8 | 0.00964 | 0.10 | 0.04 | 0.10 | SNP |
| chr6_29796106_G_T | 29796106 | 2.3 | 1.2 | 4.3 | 0.00966 | 0.03 | 0.06 | 0.03 | SNP |
| chr6_32192436_A_G | 32192436 | 0.4 | 0.2 | 0.7 | 0.00967 | 0.07 | 0.03 | 0.07 | SNP |
| chr6_29913135_G_C | 29913135 | 0.7 | 0.5 | 0.9 | 0.00972 | 0.40 | 0.39 | 0.41 | SNP |
| chr6_31463680_A_G | 31463680 | 1.6 | 1.1 | 2.3 | 0.00985 | 0.29 | 0.34 | 0.29 | SNP |
| chr6_29913817_C_T | 29913817 | 0.7 | 0.5 | 0.9 | 0.00985 | 0.40 | 0.38 | 0.40 | SNP |
| chr6_32303160_G_T | 32303160 | 0.3 | 0.1 | 0.6 | 0.00987 | 0.05 | 0.01 | 0.05 | SNP |
| chr6_32631029_T_C | 32631029 | 0.3 | 0.1 | 0.7 | 0.00990 | 0.06 | 0.02 | 0.07 | SNP |
| chr6_31237664_T_C | 31237664 | 1.7 | 1.1 | 2.4 | 0.00992 | 0.35 | 0.38 | 0.35 | SNP |
| chr6_32170433_C_T | 32170433 | 0.7 | 0.5 | 0.9 | 0.00994 | 0.47 | 0.41 | 0.48 | SNP |
| chr6_31238814_C_T | 31238814 | 1.6 | 1.1 | 2.4 | 0.00998 | 0.39 | 0.42 | 0.38 | SNP |
| chr6_32187605_G_A | 32187605 | 1.5 | 1.1 | 2.1 | 0.01002 | 0.50 | 0.54 | 0.49 | SNP |
| chr6_31238234_A_G | 31238234 | 1.7 | 1.1 | 2.4 | 0.01007 | 0.35 | 0.38 | 0.34 | SNP |
| chr6_32411523_T_C | 32411523 | 1.5 | 1.1 | 2.1 | 0.01007 | 0.39 | 0.51 | 0.38 | SNP |
| chr6_31320241_G_A | 31320241 | 1.6 | 1.1 | 2.3 | 0.01007 | 0.21 | 0.26 | 0.21 | SNP |
| chr6_29924889_C_A | 29924889 | 0.7 | 0.5 | 0.9 | 0.01009 | 0.40 | 0.39 | 0.40 | SNP |
| chr6_32781121_G_C | 32781121 | 0.5 | 0.3 | 0.8 | 0.01013 | 0.10 | 0.05 | 0.10 | SNP |
| chr6_31727897_C_T | 31727897 | 2.9 | 1.3 | 6.3 | 0.01025 | 0.12 | 0.12 | 0.12 | SNP |
| chr6_32373801_T_G | 32373801 | 1.5 | 1.1 | 2.1 | 0.01038 | 0.37 | 0.49 | 0.36 | SNP |
| chr6_32024395_C_T | 32024395 | 2.1 | 1.2 | 3.6 | 0.01039 | 0.06 | 0.07 | 0.06 | SNP |
| chr6_31561211_C_T | 31561211 | 1.6 | 1.1 | 2.3 | 0.01040 | 0.26 | 0.33 | 0.26 | SNP |
| chr6_29912595_G_A | 29912595 | 0.7 | 0.5 | 0.9 | 0.01046 | 0.40 | 0.39 | 0.41 | SNP |
| chr6_29896636_C_T | 29896636 | 1.5 | 1.1 | 2.1 | 0.01047 | 0.23 | 0.27 | 0.22 | SNP |
| chr6_29896105_G_A | 29896105 | 1.5 | 1.1 | 2.1 | 0.01051 | 0.22 | 0.26 | 0.22 | SNP |
| chr6_31241092_G_C | 31241092 | 1.5 | 1.1 | 2.2 | 0.01057 | 0.38 | 0.42 | 0.38 | SNP |
| chr6_32633273_T_G | 32633273 | 0.6 | 0.5 | 0.9 | 0.01072 | 0.36 | 0.29 | 0.37 | SNP |
| chr6_29895913_G_A | 29895913 | 0.7 | 0.5 | 0.9 | 0.01074 | 0.45 | 0.43 | 0.45 | SNP |
| chr6_32780914_G_A | 32780914 | 0.5 | 0.3 | 0.8 | 0.01080 | 0.10 | 0.05 | 0.10 | SNP |
| chr6_32052983_C_T | 32052983 | 0.5 | 0.2 | 0.8 | 0.01082 | 0.10 | 0.04 | 0.10 | SNP |
| chr6_32164587_C_T | 32164587 | 0.3 | 0.1 | 0.7 | 0.01084 | 0.04 | 0.01 | 0.05 | SNP |

| | | | | | | | | | |
|---|---|---|---|---|---|---|---|---|---|
| chr6_32785284_T_C | 32785284 | 0.5 | 0.3 | 0.8 | 0.01090 | 0.10 | 0.05 | 0.10 | SNP |
| chr6_31325341_C_G | 31325341 | 1.7 | 1.1 | 2.5 | 0.01091 | 0.32 | 0.36 | 0.32 | SNP |
| chr6_32261940_G_C | 32261940 | 0.3 | 0.1 | 0.7 | 0.01099 | 0.05 | 0.02 | 0.05 | SNP |
| chr6_32943345_T_C | 32943345 | 0.7 | 0.5 | 0.9 | 0.01101 | 0.46 | 0.38 | 0.46 | SNP |
| chr6_32609341_T_C | 32609341 | 0.6 | 0.4 | 0.9 | 0.01103 | 0.48 | 0.39 | 0.49 | SNP |
| chr6_29695180_G_A | 29695180 | 1.5 | 1.1 | 2.1 | 0.01112 | 0.23 | 0.27 | 0.22 | SNP |
| chr6_31633689_C_T | 31633689 | 1.6 | 1.1 | 2.3 | 0.01118 | 0.18 | 0.22 | 0.18 | SNP |
| chr6_31237802_G_A | 31237802 | 1.6 | 1.1 | 2.4 | 0.01118 | 0.35 | 0.38 | 0.35 | SNP |
| chr6_32559076_A_G | 32559076 | 0.6 | 0.4 | 0.9 | 0.01119 | 0.34 | 0.25 | 0.35 | SNP |
| chr6_31238230_C_G | 31238230 | 1.6 | 1.1 | 2.4 | 0.01124 | 0.35 | 0.38 | 0.34 | SNP |
| chr6_29911207_G_A | 29911207 | 0.7 | 0.5 | 0.9 | 0.01125 | 0.41 | 0.39 | 0.41 | SNP |
| chr6_31382932_G_A | 31382932 | 1.5 | 1.1 | 2.1 | 0.01126 | 0.23 | 0.24 | 0.23 | SNP |
| chr6_32373698_G_T | 32373698 | 1.5 | 1.1 | 2.1 | 0.01130 | 0.37 | 0.49 | 0.36 | SNP |
| chr6_31237463_G_C | 31237463 | 1.6 | 1.1 | 2.4 | 0.01130 | 0.34 | 0.38 | 0.34 | SNP |
| chr6_32020238_A_G | 32020238 | 2.0 | 1.1 | 3.3 | 0.01132 | 0.17 | 0.22 | 0.16 | SNP |
| chr6_29895944_G_A | 29895944 | 0.7 | 0.5 | 0.9 | 0.01137 | 0.45 | 0.43 | 0.45 | SNP |
| chr6_31325494_C_T | 31325494 | 1.7 | 1.1 | 2.5 | 0.01138 | 0.33 | 0.36 | 0.32 | SNP |
| chr6_32409305_A_G | 32409305 | 1.5 | 1.1 | 2.1 | 0.01154 | 0.39 | 0.51 | 0.38 | SNP |
| chr6_32607251_T_C | 32607251 | 0.6 | 0.4 | 0.9 | 0.01155 | 0.38 | 0.29 | 0.38 | SNP |
| xHLA_2field_DRB1_13_01 | 32546552 | 0.4 | 0.2 | 0.8 | 0.01160 | 0.09 | 0.03 | 0.09 | HLA (xHLA) |
| chr6_29893922_A_T | 29893922 | 1.6 | 1.1 | 2.2 | 0.01165 | 0.20 | 0.23 | 0.20 | SNP |
| chr6_31466547_C_T | 31466547 | 1.6 | 1.1 | 2.4 | 0.01174 | 0.29 | 0.34 | 0.28 | SNP |
| chr6_32036357_G_A | 32036357 | 0.5 | 0.2 | 0.8 | 0.01176 | 0.10 | 0.04 | 0.10 | SNP |
| chr6_32011316_G_T | 32011316 | 0.2 | 0.1 | 0.6 | 0.01181 | 0.07 | 0.01 | 0.07 | SNP |
| chr6_29716169_G_T | 29716169 | 2.4 | 1.2 | 4.5 | 0.01187 | 0.02 | 0.05 | 0.02 | SNP |
| chr6_33036950_C_T | 33036950 | 0.3 | 0.1 | 0.7 | 0.01192 | 0.05 | 0.02 | 0.06 | SNP |
| chr6_31325513_G_C | 31325513 | 1.7 | 1.1 | 2.5 | 0.01203 | 0.33 | 0.36 | 0.32 | SNP |
| chr6_29908804_G_A | 29908804 | 0.7 | 0.5 | 0.9 | 0.01214 | 0.41 | 0.39 | 0.41 | SNP |
| chr6_29913734_C_T | 29913734 | 0.7 | 0.5 | 0.9 | 0.01218 | 0.40 | 0.39 | 0.40 | SNP |
| chr6_31473844_C_T | 31473844 | 1.6 | 1.1 | 2.2 | 0.01219 | 0.29 | 0.34 | 0.29 | SNP |
| chr6_31238535_C_T | 31238535 | 1.6 | 1.1 | 2.4 | 0.01223 | 0.36 | 0.39 | 0.36 | SNP |
| chr6_32021130_C_T | 32021130 | 2.0 | 1.1 | 3.3 | 0.01225 | 0.06 | 0.07 | 0.06 | SNP |
| chr6_32096001_C_A | 32096001 | 2.8 | 1.2 | 6.3 | 0.01225 | 0.12 | 0.13 | 0.12 | SNP |
| chr6_29892107_C_T | 29892107 | 1.5 | 1.1 | 2.1 | 0.01230 | 0.21 | 0.24 | 0.20 | SNP |
| chr6_31918860_A_G | 31918860 | 2.7 | 1.2 | 5.8 | 0.01231 | 0.12 | 0.12 | 0.12 | SNP |
| chr6_29893139_G_A | 29893139 | 1.5 | 1.1 | 2.1 | 0.01235 | 0.21 | 0.24 | 0.21 | SNP |
| chr6_31325504_C_T | 31325504 | 1.7 | 1.1 | 2.5 | 0.01241 | 0.33 | 0.36 | 0.32 | SNP |
| chr6_31325380_T_C | 31325380 | 1.7 | 1.1 | 2.5 | 0.01248 | 0.33 | 0.36 | 0.32 | SNP |
| chr6_32785714_T_C | 32785714 | 1.5 | 1.1 | 2.1 | 0.01253 | 0.21 | 0.24 | 0.21 | SNP |
| chr6_32011317_T_C | 32011317 | 0.2 | 0.1 | 0.6 | 0.01257 | 0.07 | 0.01 | 0.07 | SNP |
| chr6_32029183_C_T | 32029183 | 0.5 | 0.2 | 0.8 | 0.01258 | 0.09 | 0.04 | 0.10 | SNP |
| chr6_29908469_T_C | 29908469 | 0.7 | 0.5 | 0.9 | 0.01259 | 0.41 | 0.39 | 0.41 | SNP |
| chr6_32605508_G_T | 32605508 | 1.5 | 1.1 | 2.2 | 0.01269 | 0.37 | 0.50 | 0.36 | SNP |
| chr6_29859054_C_G | 29859054 | 0.7 | 0.5 | 0.9 | 0.01271 | 0.47 | 0.45 | 0.47 | SNP |
| chr6_31726253_T_C | 31726253 | 2.8 | 1.2 | 6.2 | 0.01273 | 0.12 | 0.11 | 0.12 | SNP |
| chr6_29910371_C_T | 29910371 | 1.8 | 1.1 | 2.9 | 0.01274 | 0.08 | 0.12 | 0.08 | SNP |
| chr6_32605501_C_G | 32605501 | 1.5 | 1.1 | 2.1 | 0.01286 | 0.36 | 0.49 | 0.35 | SNP |
| chr6_32526702_C_T | 32526702 | 0.4 | 0.2 | 0.8 | 0.01292 | 0.08 | 0.04 | 0.09 | SNP |
| chr6_32407709_C_A | 32407709 | 1.5 | 1.1 | 2.1 | 0.01309 | 0.35 | 0.47 | 0.34 | SNP |
| chr6_30864279_C_T | 30864279 | 2.2 | 1.2 | 4.0 | 0.01315 | 0.11 | 0.13 | 0.11 | SNP |
| chr6_31322522_C_A | 31322522 | 0.6 | 0.4 | 0.9 | 0.01318 | 0.17 | 0.14 | 0.17 | SNP |
| chr6_32171683_T_C | 32171683 | 0.7 | 0.5 | 0.9 | 0.01319 | 0.47 | 0.41 | 0.47 | SNP |
| chr6_29908967_G_A | 29908967 | 0.7 | 0.5 | 0.9 | 0.01330 | 0.40 | 0.39 | 0.41 | SNP |
| chr6_32783365_T_C | 32783365 | 0.5 | 0.3 | 0.8 | 0.01339 | 0.10 | 0.05 | 0.10 | SNP |
| chr6_31325116_T_G | 31325116 | 1.7 | 1.1 | 2.5 | 0.01351 | 0.34 | 0.37 | 0.33 | SNP |
| chr6_29914011_G_T | 29914011 | 0.7 | 0.5 | 0.9 | 0.01366 | 0.40 | 0.38 | 0.40 | SNP |
| chr6_31657701_C_A | 31657701 | 1.6 | 1.1 | 2.3 | 0.01369 | 0.14 | 0.21 | 0.14 | SNP |
| chr6_29892229_T_G | 29892229 | 1.5 | 1.1 | 2.1 | 0.01390 | 0.25 | 0.28 | 0.25 | SNP |
| chr6_32411646_G_T | 32411646 | 0.7 | 0.5 | 0.9 | 0.01394 | 0.47 | 0.39 | 0.48 | SNP |
| chr6_32192617_T_C | 32192617 | 0.4 | 0.1 | 0.7 | 0.01399 | 0.05 | 0.02 | 0.06 | SNP |
| chr6_31237660_G_A | 31237660 | 1.6 | 1.1 | 2.4 | 0.01401 | 0.35 | 0.38 | 0.35 | SNP |
| chr6_31248169_C_T | 31248169 | 2.0 | 1.1 | 3.4 | 0.01408 | 0.14 | 0.15 | 0.14 | SNP |
| chr6_32412539_G_A | 32412539 | 0.7 | 0.5 | 0.9 | 0.01408 | 0.47 | 0.39 | 0.48 | SNP |
| chr6_33036717_C_G | 33036717 | 0.6 | 0.4 | 0.9 | 0.01413 | 0.14 | 0.08 | 0.15 | SNP |
| chr6_32412398_T_C | 32412398 | 0.7 | 0.5 | 0.9 | 0.01422 | 0.47 | 0.39 | 0.48 | SNP |
| chr6_29909044_G_C | 29909044 | 0.7 | 0.5 | 0.9 | 0.01427 | 0.40 | 0.39 | 0.40 | SNP |
| chr6_32633467_A_G | 32633467 | 0.5 | 0.2 | 0.8 | 0.01437 | 0.09 | 0.04 | 0.10 | SNP |
| chr6_31321327_A_G | 31321327 | 0.6 | 0.5 | 0.9 | 0.01446 | 0.24 | 0.18 | 0.24 | SNP |
| chr6_31474688_G_T | 31474688 | 1.6 | 1.1 | 2.2 | 0.01447 | 0.29 | 0.34 | 0.29 | SNP |
| chr6_29638434_A_G | 29638434 | 0.6 | 0.4 | 0.9 | 0.01449 | 0.15 | 0.10 | 0.15 | SNP |
| xHLA_2field_DQB1_02_01 | 32627244 | 2.2 | 1.1 | 4.0 | 0.01452 | 0.13 | 0.15 | 0.12 | HLA (xHLA) |
| chr6_32785029_T_C | 32785029 | 0.5 | 0.3 | 0.8 | 0.01456 | 0.10 | 0.05 | 0.10 | SNP |
| chr6_33053352_C_T | 33053352 | 0.6 | 0.4 | 0.9 | 0.01464 | 0.21 | 0.16 | 0.22 | SNP |
| chr6_33036708_C_T | 33036708 | 0.6 | 0.4 | 0.9 | 0.01465 | 0.14 | 0.08 | 0.15 | SNP |
| chr6_33036709_A_G | 33036709 | 0.6 | 0.4 | 0.9 | 0.01465 | 0.14 | 0.08 | 0.15 | SNP |
| chr6_32339498_C_G | 32339498 | 0.3 | 0.1 | 0.7 | 0.01482 | 0.05 | 0.02 | 0.05 | SNP |
| chr6_31325532_A_G | 31325532 | 1.6 | 1.1 | 2.4 | 0.01489 | 0.33 | 0.36 | 0.32 | SNP |
| chr6_31463621_G_A | 31463621 | 1.6 | 1.1 | 2.2 | 0.01494 | 0.29 | 0.34 | 0.29 | SNP |
| chr6_32785035_A_G | 32785035 | 0.5 | 0.3 | 0.8 | 0.01494 | 0.10 | 0.05 | 0.10 | SNP |
| chr6_32628098_G_C | 32628098 | 0.3 | 0.1 | 0.7 | 0.01502 | 0.05 | 0.02 | 0.05 | SNP |
| chr6_32013500_T_C | 32013500 | 0.5 | 0.2 | 0.8 | 0.01511 | 0.10 | 0.05 | 0.10 | SNP |
| chr6_29913912_C_T | 29913912 | 0.7 | 0.5 | 0.9 | 0.01516 | 0.40 | 0.39 | 0.40 | SNP |
| chr6_32946133_T_C | 32946133 | 1.4 | 1.1 | 1.9 | 0.01517 | 0.42 | 0.51 | 0.41 | SNP |
| chr6_31325526_A_G | 31325526 | 1.6 | 1.1 | 2.4 | 0.01518 | 0.33 | 0.36 | 0.32 | SNP |
| chr6_31325529_C_T | 31325529 | 1.6 | 1.1 | 2.4 | 0.01518 | 0.33 | 0.36 | 0.32 | SNP |
| chr6_31325521_C_G | 31325521 | 1.6 | 1.1 | 2.4 | 0.01521 | 0.33 | 0.36 | 0.32 | SNP |
| chr6_29712878_G_A | 29712878 | 2.4 | 1.1 | 4.7 | 0.01543 | 0.02 | 0.05 | 0.02 | SNP |
| chr6_29893235_C_T | 29893235 | 0.7 | 0.5 | 0.9 | 0.01551 | 0.45 | 0.43 | 0.45 | SNP |
| chr6_31592820_C_T | 31592820 | 0.7 | 0.5 | 0.9 | 0.01552 | 0.34 | 0.31 | 0.34 | SNP |
| chr6_29892233_T_C | 29892233 | 1.5 | 1.1 | 2.0 | 0.01559 | 0.25 | 0.28 | 0.25 | SNP |
| chr6_32412480_A_G | 32412480 | 0.7 | 0.5 | 0.9 | 0.01578 | 0.47 | 0.39 | 0.48 | SNP |
| chr6_29901898_C_G | 29901898 | 0.7 | 0.5 | 0.9 | 0.01580 | 0.44 | 0.43 | 0.44 | SNP |
| chr6_31321425_G_A | 31321425 | 0.6 | 0.4 | 0.9 | 0.01585 | 0.21 | 0.17 | 0.21 | SNP |
| chr6_29893403_C_G | 29893403 | 1.6 | 1.1 | 2.5 | 0.01604 | 0.15 | 0.17 | 0.15 | SNP |
| chr6_32812528_C_T | 32812528 | 1.5 | 1.1 | 2.0 | 0.01621 | 0.16 | 0.21 | 0.16 | SNP |
| chr6_31369151_G_A | 31369151 | 0.7 | 0.5 | 0.9 | 0.01623 | 0.34 | 0.30 | 0.34 | SNP |
| chr6_29896659_C_T | 29896659 | 0.7 | 0.5 | 0.9 | 0.01636 | 0.45 | 0.43 | 0.45 | SNP |
| chr6_32948426_C_T | 32948426 | 0.5 | 0.3 | 0.9 | 0.01638 | 0.09 | 0.06 | 0.10 | SNP |
| chr6_30524614_A_G | 30524614 | 2.5 | 1.1 | 5.0 | 0.01652 | 0.02 | 0.04 | 0.02 | SNP |
| chr6_32606695_T_C | 32606695 | 0.5 | 0.3 | 0.9 | 0.01665 | 0.11 | 0.06 | 0.12 | SNP |
| chr6_31731014_C_G | 31731014 | 1.5 | 1.1 | 2.1 | 0.01669 | 0.17 | 0.21 | 0.17 | SNP |
| chr6_29908426_C_T | 29908426 | 0.7 | 0.5 | 0.9 | 0.01700 | 0.40 | 0.39 | 0.41 | SNP |
| chr6_29713155_G_C | 29713155 | 1.7 | 1.1 | 2.5 | 0.01712 | 0.09 | 0.13 | 0.09 | SNP |
| chr6_32634990_G_T | 32634990 | 0.5 | 0.2 | 0.8 | 0.01714 | 0.09 | 0.04 | 0.09 | SNP |
| chr6_32029431_G_A | 32029431 | 2.0 | 1.1 | 3.4 | 0.01725 | 0.06 | 0.07 | 0.06 | SNP |
| chr6_29896120_G_T | 29896120 | 0.7 | 0.5 | 0.9 | 0.01727 | 0.48 | 0.47 | 0.48 | SNP |
| chr6_31326629_T_C | 31326629 | 1.6 | 1.1 | 2.3 | 0.01728 | 0.34 | 0.37 | 0.34 | SNP |
| chr6_29693969_C_T | 29693969 | 1.5 | 1.1 | 2.0 | 0.01729 | 0.23 | 0.27 | 0.23 | SNP |
| chr6_29693938_G_A | 29693938 | 0.6 | 0.4 | 0.9 | 0.01739 | 0.15 | 0.10 | 0.15 | SNP |
| chr6_31542308_T_C | 31542308 | 0.6 | 0.4 | 0.9 | 0.01742 | 0.19 | 0.16 | 0.19 | SNP |
| chr6_29691857_C_G | 29691857 | 0.7 | 0.5 | 0.9 | 0.01777 | 0.46 | 0.42 | 0.46 | SNP |
| chr6_31473546_G_A | 31473546 | 1.6 | 1.1 | 2.2 | 0.01790 | 0.29 | 0.33 | 0.28 | SNP |
| chr6_32303848_G_A | 32303848 | 1.6 | 1.1 | 2.3 | 0.01792 | 0.24 | 0.34 | 0.23 | SNP |
| chr6_32604213_A_C | 32604213 | 0.6 | 0.4 | 0.9 | 0.01807 | 0.40 | 0.31 | 0.41 | SNP |
| chr6_32606912_A_G | 32606912 | 0.6 | 0.4 | 0.9 | 0.01807 | 0.16 | 0.09 | 0.16 | SNP |
| chr6_29893154_A_T | 29893154 | 0.7 | 0.5 | 0.9 | 0.01813 | 0.45 | 0.44 | 0.45 | SNP |
| chr6_32056580_A_C | 32056580 | 0.5 | 0.2 | 0.8 | 0.01821 | 0.10 | 0.04 | 0.10 | SNP |
| chr6_32491581_A_C | 32491581 | 1.6 | 1.1 | 2.3 | 0.01821 | 0.26 | 0.38 | 0.25 | SNP |
| chr6_32551638_C_T | 32551638 | 0.4 | 0.2 | 0.8 | 0.01830 | 0.07 | 0.04 | 0.08 | SNP |
| chr6_32014575_A_G | 32014575 | 0.7 | 0.5 | 0.9 | 0.01844 | 0.48 | 0.47 | 0.48 | SNP |
| chr6_29943178_A_G | 29943178 | 0.7 | 0.5 | 0.9 | 0.01867 | 0.35 | 0.33 | 0.35 | SNP |
| chr6_29944670_G_C | 29944670 | 1.5 | 1.1 | 2.0 | 0.01886 | 0.46 | 0.49 | 0.46 | SNP |
| chr6_32786833_C_G | 32786833 | 0.7 | 0.5 | 0.9 | 0.01914 | 0.38 | 0.31 | 0.39 | SNP |
| chr6_31465915_C_G | 31465915 | 1.6 | 1.1 | 2.2 | 0.01918 | 0.29 | 0.33 | 0.28 | SNP |
| chr6_32375745_G_A | 32375745 | 1.5 | 1.1 | 2.1 | 0.01925 | 0.48 | 0.56 | 0.47 | SNP |
| chr6_32376618_C_T | 32376618 | 1.5 | 1.1 | 2.1 | 0.01925 | 0.48 | 0.56 | 0.47 | SNP |
| chr6_32007625_C_T | 32007625 | 0.5 | 0.3 | 0.9 | 0.01927 | 0.10 | 0.05 | 0.10 | SNP |
| chr6_29892203_C_A | 29892203 | 1.5 | 1.1 | 2.0 | 0.01931 | 0.25 | 0.28 | 0.24 | SNP |
| chr6_31502861_G_A | 31502861 | 1.6 | 1.1 | 2.5 | 0.01943 | 0.23 | 0.29 | 0.22 | SNP |
| chr6_31325115_G_A | 31325115 | 1.6 | 1.1 | 2.4 | 0.01952 | 0.34 | 0.37 | 0.34 | SNP |
| chr6_33386728_G_C | 33386728 | 2.1 | 1.1 | 3.7 | 0.01963 | 0.03 | 0.05 | 0.03 | SNP |
| chr6_29914034_A_G | 29914034 | 1.4 | 1.1 | 1.9 | 0.01969 | 0.39 | 0.41 | 0.39 | SNP |
| chr6_31602967_G_A | 31602967 | 1.4 | 1.1 | 2.0 | 0.01980 | 0.37 | 0.40 | 0.37 | SNP |
| chr6_29859039_C_G | 29859039 | 0.7 | 0.5 | 0.9 | 0.02004 | 0.46 | 0.44 | 0.46 | SNP |
| chr6_29896114_G_A | 29896114 | 0.7 | 0.6 | 1.0 | 0.02026 | 0.48 | 0.47 | 0.48 | SNP |
| chr6_29759511_C_T | 29759511 | 2.1 | 1.1 | 3.8 | 0.02028 | 0.03 | 0.05 | 0.02 | SNP |
| chr6_29892815_A_G | 29892815 | 1.6 | 1.1 | 2.5 | 0.02034 | 0.15 | 0.17 | 0.14 | SNP |
| chr6_32410215_T_C | 32410215 | 0.6 | 0.4 | 0.9 | 0.02051 | 0.22 | 0.16 | 0.23 | SNP |
| chr6_31322133_A_G | 31322133 | 0.7 | 0.5 | 0.9 | 0.02092 | 0.35 | 0.29 | 0.35 | SNP |
| chr6_32709990_C_T | 32709990 | 0.7 | 0.5 | 0.9 | 0.02093 | 0.32 | 0.23 | 0.33 | SNP |
| chr6_32410210_T_C | 32410210 | 0.6 | 0.4 | 0.9 | 0.02093 | 0.22 | 0.16 | 0.23 | SNP |
| chr6_33257767_T_G | 33257767 | 2.6 | 1.1 | 5.7 | 0.02144 | 0.01 | 0.03 | 0.01 | SNP |
| chr6_30796545_T_C | 30796545 | 1.9 | 1.1 | 3.4 | 0.02150 | 0.12 | 0.13 | 0.12 | SNP |
| chr6_31473286_T_C | 31473286 | 1.5 | 1.1 | 2.2 | 0.02155 | 0.29 | 0.33 | 0.28 | SNP |
| chr6_32710010_G_T | 32710010 | 0.7 | 0.5 | 0.9 | 0.02159 | 0.32 | 0.23 | 0.33 | SNP |
| chr6_32052444_C_T | 32052444 | 0.3 | 0.1 | 0.7 | 0.02160 | 0.04 | 0.01 | 0.04 | SNP |
| chr6_32029849_G_A | 32029849 | 0.5 | 0.2 | 0.9 | 0.02161 | 0.10 | 0.04 | 0.10 | SNP |
| chr6_29909797_C_T | 29909797 | 1.5 | 1.1 | 2.2 | 0.02169 | 0.18 | 0.21 | 0.18 | SNP |
| chr6_32971708_G_A | 32971708 | 0.6 | 0.4 | 0.9 | 0.02170 | 0.20 | 0.14 | 0.20 | SNP |
| chr6_32709964_T_C | 32709964 | 0.7 | 0.5 | 0.9 | 0.02181 | 0.32 | 0.23 | 0.33 | SNP |
| chr6_32559444_C_T | 32559444 | 0.5 | 0.3 | 0.9 | 0.02188 | 0.10 | 0.04 | 0.10 | SNP |
| chr6_32607270_A_G | 32607270 | 0.5 | 0.2 | 0.9 | 0.02196 | 0.08 | 0.04 | 0.09 | SNP |
| chr6_31382882_C_A | 31382882 | 1.5 | 1.1 | 2.0 | 0.02200 | 0.23 | 0.24 | 0.23 | SNP |
| chr6_32377061_C_T | 32377061 | 1.5 | 1.1 | 2.2 | 0.02206 | 0.48 | 0.55 | 0.47 | SNP |
| chr6_31323353_G_A | 31323353 | 0.7 | 0.5 | 0.9 | 0.02215 | 0.36 | 0.34 | 0.36 | SNP |

| | | | | | | | | | |
|---|---|---|---|---|---|---|---|---|---|
| chr6_31506854_T_C | 31506854 | 1.6 | 1.1 | 2.4 | 0.02226 | 0.23 | 0.29 | 0.23 | SNP |
| chr6_32409060_G_A | 32409060 | 0.6 | 0.4 | 0.9 | 0.02236 | 0.22 | 0.16 | 0.23 | SNP |
| chr6_31924880_G_A | 31924880 | 0.5 | 0.2 | 0.9 | 0.02245 | 0.09 | 0.04 | 0.09 | SNP |
| chr6_29855401_C_G | 29855401 | 0.7 | 0.5 | 1.0 | 0.02247 | 0.43 | 0.41 | 0.43 | SNP |
| chr6_31620020_A_G | 31620020 | 1.5 | 1.1 | 2.0 | 0.02278 | 0.38 | 0.41 | 0.38 | SNP |
| chr6_32550591_G_A | 32550591 | 0.4 | 0.1 | 0.8 | 0.02313 | 0.06 | 0.03 | 0.06 | SNP |
| chr6_33045698_G_A | 33045698 | 0.3 | 0.1 | 0.7 | 0.02318 | 0.04 | 0.01 | 0.05 | SNP |
| chr6_31930351_A_G | 31930351 | 0.4 | 0.1 | 0.8 | 0.02333 | 0.05 | 0.02 | 0.05 | SNP |
| chr6_31932177_C_T | 31932177 | 0.5 | 0.3 | 0.9 | 0.02363 | 0.12 | 0.06 | 0.12 | SNP |
| chr6_29856774_A_G | 29856774 | 1.6 | 1.1 | 2.4 | 0.02364 | 0.14 | 0.17 | 0.14 | SNP |
| chr6_29908659_T_C | 29908659 | 0.7 | 0.5 | 1.0 | 0.02380 | 0.43 | 0.42 | 0.43 | SNP |
| chr6_31462135_G_A | 31462135 | 2.7 | 1.1 | 6.2 | 0.02385 | 0.12 | 0.13 | 0.12 | SNP |
| chr6_31556581_T_A | 31556581 | 0.7 | 0.5 | 0.9 | 0.02414 | 0.24 | 0.21 | 0.24 | SNP |
| chr6_32818432_C_A | 32818432 | 0.3 | 0.1 | 0.7 | 0.02430 | 0.04 | 0.01 | 0.04 | SNP |
| chr6_31925921_G_A | 31925921 | 0.3 | 0.1 | 0.8 | 0.02434 | 0.04 | 0.01 | 0.04 | SNP |
| chr6_31919956_T_C | 31919956 | 0.5 | 0.2 | 0.9 | 0.02442 | 0.09 | 0.04 | 0.09 | SNP |
| chr6_32725271_G_A | 32725271 | 0.6 | 0.4 | 0.9 | 0.02444 | 0.21 | 0.15 | 0.22 | SNP |
| chr6_31709814_G_C | 31709814 | 0.5 | 0.3 | 0.9 | 0.02454 | 0.06 | 0.06 | 0.06 | SNP |
| chr6_31910938_G_T | 31910938 | 0.5 | 0.2 | 0.9 | 0.02461 | 0.09 | 0.04 | 0.09 | SNP |
| chr6_31916951_T_C | 31916951 | 0.5 | 0.2 | 0.9 | 0.02461 | 0.09 | 0.04 | 0.09 | SNP |
| chr6_31919917_T_C | 31919917 | 0.5 | 0.2 | 0.9 | 0.02461 | 0.09 | 0.04 | 0.09 | SNP |
| chr6_32441100_C_A | 32441100 | 0.6 | 0.4 | 0.9 | 0.02462 | 0.28 | 0.23 | 0.28 | SNP |
| chr6_31910929_G_A | 31910929 | 0.5 | 0.2 | 0.9 | 0.02474 | 0.09 | 0.04 | 0.09 | SNP |
| chr6_31464325_T_C | 31464325 | 0.7 | 0.5 | 0.9 | 0.02481 | 0.30 | 0.22 | 0.30 | SNP |
| chr6_31322303_C_G | 31322303 | 0.7 | 0.5 | 1.0 | 0.02485 | 0.34 | 0.29 | 0.35 | SNP |
| chr6_31912009_G_C | 31912009 | 2.0 | 1.1 | 3.5 | 0.02496 | 0.05 | 0.05 | 0.05 | SNP |
| chr6_29796103_T_C | 29796103 | 1.5 | 1.0 | 2.0 | 0.02501 | 0.44 | 0.48 | 0.43 | SNP |
| chr6_31928306_A_G | 31928306 | 0.5 | 0.2 | 0.9 | 0.02504 | 0.09 | 0.04 | 0.09 | SNP |
| chr6_31325092_G_A | 31325092 | 1.6 | 1.1 | 2.4 | 0.02506 | 0.34 | 0.37 | 0.33 | SNP |
| chr6_32605861_A_G | 32605861 | 0.5 | 0.2 | 0.9 | 0.02507 | 0.08 | 0.04 | 0.09 | SNP |
| chr6_32035427_T_C | 32035427 | 0.5 | 0.2 | 0.9 | 0.02511 | 0.10 | 0.05 | 0.10 | SNP |
| chr6_32341353_G_A | 32341353 | 1.4 | 1.0 | 2.0 | 0.02526 | 0.47 | 0.55 | 0.46 | SNP |
| chr6_32782018_C_T | 32782018 | 0.5 | 0.3 | 0.9 | 0.02539 | 0.10 | 0.06 | 0.10 | SNP |
| chr6_32603405_C_T | 32603405 | 0.5 | 0.2 | 0.9 | 0.02546 | 0.08 | 0.04 | 0.09 | SNP |
| chr6_32605950_C_T | 32605950 | 0.5 | 0.2 | 0.9 | 0.02546 | 0.08 | 0.04 | 0.09 | SNP |
| chr6_32606960_A_T | 32606960 | 0.5 | 0.2 | 0.9 | 0.02546 | 0.08 | 0.04 | 0.09 | SNP |
| chr6_31380449_C_T | 31380449 | 1.5 | 1.1 | 2.2 | 0.02554 | 0.34 | 0.35 | 0.34 | SNP |
| chr6_32024234_G_A | 32024234 | 3.2 | 1.0 | 8.2 | 0.02556 | 0.01 | 0.02 | 0.01 | SNP |
| chr6_32370835_T_C | 32370835 | 1.5 | 1.0 | 2.0 | 0.02559 | 0.37 | 0.48 | 0.36 | SNP |
| chr6_29895604_C_G | 29895604 | 0.7 | 0.6 | 1.0 | 0.02564 | 0.45 | 0.45 | 0.45 | SNP |
| chr6_32409781_C_T | 32409781 | 1.5 | 1.0 | 2.0 | 0.02586 | 0.39 | 0.50 | 0.38 | SNP |
| chr6_31795345_G_A | 31795345 | 0.3 | 0.1 | 0.8 | 0.02601 | 0.04 | 0.01 | 0.04 | SNP |
| chr6_32606742_A_G | 32606742 | 0.5 | 0.2 | 0.9 | 0.02612 | 0.08 | 0.04 | 0.09 | SNP |
| chr6_32604384_G_A | 32604384 | 0.5 | 0.2 | 0.9 | 0.02619 | 0.08 | 0.04 | 0.09 | SNP |
| chr6_32376882_T_G | 32376882 | 1.5 | 1.0 | 2.1 | 0.02625 | 0.48 | 0.55 | 0.47 | SNP |
| chr6_31239430_C_T | 31239430 | 0.7 | 0.5 | 1.0 | 0.02630 | 0.47 | 0.44 | 0.48 | SNP |
| chr6_33036401_C_T | 33036401 | 0.6 | 0.4 | 0.9 | 0.02632 | 0.15 | 0.09 | 0.15 | SNP |
| chr6_29858840_A_C | 29858840 | 1.6 | 1.0 | 2.4 | 0.02670 | 0.15 | 0.17 | 0.14 | SNP |
| chr6_29893293_A_G | 29893293 | 1.4 | 1.0 | 1.8 | 0.02691 | 0.47 | 0.48 | 0.47 | SNP |
| chr6_32606676_T_C | 32606676 | 0.5 | 0.2 | 0.9 | 0.02708 | 0.08 | 0.04 | 0.09 | SNP |
| chr6_31382938_A_G | 31382938 | 1.4 | 1.0 | 2.0 | 0.02727 | 0.23 | 0.23 | 0.23 | SNP |
| chr6_31382942_G_C | 31382942 | 1.4 | 1.0 | 2.0 | 0.02727 | 0.23 | 0.23 | 0.23 | SNP |
| chr6_31593476_A_G | 31593476 | 0.7 | 0.5 | 1.0 | 0.02741 | 0.33 | 0.31 | 0.33 | SNP |
| chr6_31378387_T_C | 31378387 | 1.5 | 1.0 | 2.0 | 0.02741 | 0.24 | 0.24 | 0.24 | SNP |
| chr6_31382994_C_A | 31382994 | 1.5 | 1.0 | 2.1 | 0.02767 | 0.18 | 0.18 | 0.18 | SNP |
| chr6_29942527_T_C | 29942527 | 0.5 | 0.3 | 0.9 | 0.02773 | 0.07 | 0.05 | 0.07 | SNP |
| chr6_32029205_G_A | 32029205 | 2.5 | 1.1 | 5.6 | 0.02779 | 0.12 | 0.12 | 0.12 | SNP |
| chr6_32608425_C_A | 32608425 | 0.5 | 0.2 | 0.9 | 0.02780 | 0.08 | 0.04 | 0.09 | SNP |
| chr6_31320176_C_T | 31320176 | 0.6 | 0.4 | 0.9 | 0.02804 | 0.18 | 0.15 | 0.19 | SNP |
| chr6_29796115_A_C | 29796115 | 1.4 | 1.0 | 2.0 | 0.02807 | 0.43 | 0.46 | 0.43 | SNP |
| chr6_31323233_A_G | 31323233 | 0.7 | 0.5 | 1.0 | 0.02811 | 0.36 | 0.34 | 0.37 | SNP |
| chr6_31239827_T_C | 31239827 | 1.6 | 1.0 | 2.4 | 0.02817 | 0.37 | 0.40 | 0.37 | SNP |
| chr6_31506624_C_T | 31506624 | 1.6 | 1.0 | 2.3 | 0.02820 | 0.23 | 0.29 | 0.23 | SNP |
| chr6_32376932_C_T | 32376932 | 1.5 | 1.0 | 2.1 | 0.02827 | 0.48 | 0.55 | 0.47 | SNP |
| chr6_32627700_A_G | 32627700 | 0.6 | 0.4 | 0.9 | 0.02830 | 0.20 | 0.12 | 0.20 | SNP |
| chr6_32607017_T_A | 32607017 | 0.5 | 0.3 | 0.9 | 0.02835 | 0.08 | 0.04 | 0.09 | SNP |
| chr6_31367865_G_A | 31367865 | 0.7 | 0.5 | 1.0 | 0.02876 | 0.33 | 0.31 | 0.34 | SNP |
| chr6_31590898_C_T | 31590898 | 0.7 | 0.5 | 1.0 | 0.02913 | 0.33 | 0.31 | 0.33 | SNP |
| chr6_29909071_G_A | 29909071 | 1.5 | 1.0 | 2.2 | 0.02914 | 0.19 | 0.21 | 0.18 | SNP |
| chr6_29908491_G_A | 29908491 | 1.4 | 1.0 | 1.9 | 0.02916 | 0.18 | 0.23 | 0.18 | SNP |
| chr6_32340176_C_T | 32340176 | 0.6 | 0.4 | 0.9 | 0.02928 | 0.24 | 0.17 | 0.24 | SNP |
| chr6_32412809_T_A | 32412809 | 0.6 | 0.4 | 0.9 | 0.02935 | 0.23 | 0.17 | 0.23 | SNP |
| chr6_29911684_C_T | 29911684 | 1.5 | 1.0 | 2.2 | 0.02938 | 0.19 | 0.21 | 0.18 | SNP |
| chr6_29913430_C_T | 29913430 | 1.5 | 1.0 | 2.2 | 0.02942 | 0.18 | 0.20 | 0.18 | SNP |
| chr6_32412592_G_A | 32412592 | 0.6 | 0.4 | 0.9 | 0.02949 | 0.23 | 0.17 | 0.23 | SNP |
| chr6_33054861_A_G | 33054861 | 0.7 | 0.5 | 1.0 | 0.02950 | 0.22 | 0.17 | 0.22 | SNP |
| chr6_32012755_G_A | 32012755 | 0.5 | 0.2 | 0.9 | 0.02958 | 0.08 | 0.04 | 0.09 | SNP |
| chr6_32556677_C_A | 32556677 | 1.6 | 1.0 | 2.5 | 0.02962 | 0.10 | 0.16 | 0.10 | SNP |
| chr6_32796793_G_A | 32796793 | 1.6 | 1.0 | 2.3 | 0.02962 | 0.09 | 0.14 | 0.09 | SNP |
| chr6_32340068_T_C | 32340068 | 0.7 | 0.5 | 1.0 | 0.02963 | 0.50 | 0.59 | 0.49 | SNP |
| chr6_32005649_A_G | 32005649 | 0.6 | 0.3 | 0.9 | 0.02970 | 0.11 | 0.06 | 0.12 | SNP |
| chr6_31931137_T_C | 31931137 | 0.6 | 0.3 | 0.9 | 0.02978 | 0.12 | 0.07 | 0.13 | SNP |
| chr6_32340872_C_G | 32340872 | 0.6 | 0.4 | 0.9 | 0.02995 | 0.24 | 0.18 | 0.24 | SNP |
| chr6_32410691_G_A | 32410691 | 0.6 | 0.4 | 0.9 | 0.02995 | 0.23 | 0.17 | 0.23 | SNP |
| chr6_33045659_T_C | 33045659 | 0.3 | 0.1 | 0.8 | 0.03017 | 0.04 | 0.01 | 0.04 | SNP |
| chr6_29892709_A_G | 29892709 | 0.7 | 0.6 | 1.0 | 0.03038 | 0.50 | 0.51 | 0.50 | SNP |
| chr6_31528326_C_G | 31528326 | 1.4 | 1.0 | 1.9 | 0.03044 | 0.38 | 0.44 | 0.38 | SNP |
| chr6_32819865_T_C | 32819865 | 0.6 | 0.4 | 0.9 | 0.03047 | 0.13 | 0.09 | 0.13 | SNP |
| chr6_33053942_T_C | 33053942 | 0.7 | 0.5 | 1.0 | 0.03051 | 0.22 | 0.17 | 0.22 | SNP |
| chr6_32411307_C_T | 32411307 | 0.6 | 0.4 | 0.9 | 0.03052 | 0.23 | 0.17 | 0.23 | SNP |
| chr6_32412566_T_A | 32412566 | 0.6 | 0.4 | 0.9 | 0.03052 | 0.23 | 0.17 | 0.23 | SNP |
| chr6_32709204_G_C | 32709204 | 0.6 | 0.4 | 0.9 | 0.03060 | 0.21 | 0.15 | 0.22 | SNP |
| chr6_29896112_C_T | 29896112 | 1.6 | 1.0 | 2.4 | 0.03066 | 0.16 | 0.18 | 0.15 | SNP |
| chr6_32944746_G_A | 32944746 | 1.3 | 1.0 | 1.8 | 0.03092 | 0.42 | 0.50 | 0.42 | SNP |
| chr6_33053812_A_G | 33053812 | 0.7 | 0.5 | 1.0 | 0.03131 | 0.22 | 0.17 | 0.22 | SNP |
| chr6_33036394_G_A | 33036394 | 0.6 | 0.4 | 0.9 | 0.03136 | 0.14 | 0.09 | 0.15 | SNP |
| chr6_33053396_A_G | 33053396 | 0.7 | 0.5 | 1.0 | 0.03137 | 0.21 | 0.17 | 0.22 | SNP |
| chr6_31465637_C_T | 31465637 | 0.3 | 0.1 | 0.8 | 0.03148 | 0.05 | 0.02 | 0.05 | SNP |
| chr6_32559520_C_T | 32559520 | 1.4 | 1.0 | 2.0 | 0.03148 | 0.32 | 0.45 | 0.31 | SNP |
| chr6_31464321_T_A | 31464321 | 0.7 | 0.5 | 1.0 | 0.03157 | 0.30 | 0.23 | 0.30 | SNP |
| chr6_30878857_G_A | 30878857 | 0.5 | 0.3 | 0.9 | 0.03158 | 0.06 | 0.06 | 0.06 | SNP |
| chr6_31325792_G_A | 31325792 | 1.5 | 1.0 | 2.2 | 0.03176 | 0.36 | 0.39 | 0.35 | SNP |
| chr6_32409787_T_C | 32409787 | 1.4 | 1.0 | 2.0 | 0.03204 | 0.39 | 0.50 | 0.38 | SNP |
| chr6_32007063_C_T | 32007063 | 0.6 | 0.4 | 0.9 | 0.03211 | 0.27 | 0.20 | 0.27 | SNP |
| chr6_32611759_T_G | 32611759 | 0.5 | 0.3 | 0.9 | 0.03221 | 0.09 | 0.04 | 0.09 | SNP |
| chr6_33036617_C_T | 33036617 | 0.6 | 0.4 | 0.9 | 0.03225 | 0.15 | 0.09 | 0.15 | SNP |
| chr6_29913639_A_G | 29913639 | 1.5 | 1.0 | 2.1 | 0.03227 | 0.19 | 0.21 | 0.18 | SNP |
| chr6_32192107_C_G | 32192107 | 0.6 | 0.4 | 0.9 | 0.03235 | 0.14 | 0.10 | 0.14 | SNP |
| chr6_29895929_C_G | 29895929 | 0.7 | 0.6 | 1.0 | 0.03237 | 0.48 | 0.46 | 0.48 | SNP |
| chr6_31466057_G_A | 31466057 | 0.3 | 0.1 | 0.8 | 0.03245 | 0.05 | 0.01 | 0.05 | SNP |
| chr6_31373174_C_T | 31373174 | 0.7 | 0.5 | 1.0 | 0.03275 | 0.36 | 0.33 | 0.36 | SNP |
| chr6_32557301_T_C | 32557301 | 0.3 | 0.1 | 0.8 | 0.03295 | 0.04 | 0.01 | 0.04 | SNP |
| chr6_32707585_G_A | 32707585 | 0.6 | 0.4 | 0.9 | 0.03306 | 0.21 | 0.15 | 0.22 | SNP |
| chr6_33033266_T_A | 33033266 | 0.6 | 0.4 | 0.9 | 0.03323 | 0.15 | 0.09 | 0.15 | SNP |
| chr6_31540429_G_C | 31540429 | 1.4 | 1.0 | 1.9 | 0.03337 | 0.38 | 0.44 | 0.37 | SNP |
| chr6_33036630_G_T | 33036630 | 0.6 | 0.4 | 0.9 | 0.03340 | 0.15 | 0.09 | 0.15 | SNP |
| chr6_33036631_A_C | 33036631 | 0.6 | 0.4 | 0.9 | 0.03340 | 0.15 | 0.09 | 0.15 | SNP |
| chr6_31605199_G_C | 31605199 | 0.7 | 0.5 | 1.0 | 0.03350 | 0.44 | 0.41 | 0.44 | SNP |
| chr6_32609147_A_T | 32609147 | 0.6 | 0.4 | 0.9 | 0.03362 | 0.16 | 0.10 | 0.17 | SNP |
| chr6_33037579_A_T | 33037579 | 0.6 | 0.4 | 0.9 | 0.03362 | 0.15 | 0.09 | 0.15 | SNP |
| chr6_33037580_T_G | 33037580 | 0.6 | 0.4 | 0.9 | 0.03362 | 0.15 | 0.09 | 0.15 | SNP |
| chr6_29768748_C_T | 29768748 | 2.1 | 1.0 | 3.9 | 0.03372 | 0.03 | 0.05 | 0.02 | SNP |
| chr6_33036388_C_T | 33036388 | 0.6 | 0.4 | 0.9 | 0.03375 | 0.14 | 0.09 | 0.15 | SNP |
| chr6_32025870_C_T | 32025870 | 0.5 | 0.2 | 0.9 | 0.03377 | 0.10 | 0.04 | 0.10 | SNP |
| chr6_29895701_T_C | 29895701 | 1.4 | 1.0 | 2.0 | 0.03381 | 0.21 | 0.24 | 0.21 | SNP |
| chr6_29911225_A_G | 29911225 | 1.6 | 1.0 | 2.3 | 0.03400 | 0.10 | 0.14 | 0.10 | SNP |
| chr6_32604660_C_T | 32604660 | 0.5 | 0.3 | 0.9 | 0.03400 | 0.09 | 0.04 | 0.10 | SNP |
| chr6_31914180_G_A | 31914180 | 0.5 | 0.3 | 0.9 | 0.03414 | 0.08 | 0.04 | 0.09 | SNP |
| chr6_29974179_T_C | 29974179 | 2.4 | 1.0 | 5.0 | 0.03419 | 0.02 | 0.03 | 0.02 | SNP |
| chr6_32612096_A_G | 32612096 | 0.5 | 0.3 | 0.9 | 0.03424 | 0.09 | 0.04 | 0.09 | SNP |
| chr6_32610976_A_C | 32610976 | 0.5 | 0.3 | 0.9 | 0.03426 | 0.09 | 0.04 | 0.09 | SNP |
| chr6_31372569_C_T | 31372569 | 0.7 | 0.5 | 1.0 | 0.03427 | 0.35 | 0.32 | 0.35 | SNP |
| xHLA_2field_DQB1_06_04 | 32627244 | 0.4 | 0.1 | 0.8 | 0.03438 | 0.05 | 0.02 | 0.05 | HLA (xHLA) |
| chr6_32014828_G_A | 32014828 | 2.4 | 1.1 | 5.5 | 0.03462 | 0.12 | 0.13 | 0.12 | SNP |
| chr6_29759222_G_A | 29759222 | 2.0 | 1.0 | 3.8 | 0.03470 | 0.02 | 0.04 | 0.02 | SNP |
| chr6_29910419_G_T | 29910419 | 1.7 | 1.0 | 2.7 | 0.03470 | 0.08 | 0.11 | 0.08 | SNP |
| chr6_29943916_A_T | 29943916 | 0.7 | 0.6 | 1.0 | 0.03487 | 0.35 | 0.35 | 0.35 | SNP |
| chr6_29912754_A_G | 29912754 | 1.4 | 1.0 | 1.8 | 0.03493 | 0.48 | 0.50 | 0.48 | SNP |
| chr6_29942639_A_G | 29942639 | 0.7 | 0.6 | 1.0 | 0.03498 | 0.35 | 0.35 | 0.35 | SNP |
| chr6_29758222_T_G | 29758222 | 2.0 | 1.0 | 3.6 | 0.03514 | 0.03 | 0.05 | 0.02 | SNP |
| chr6_31248204_G_A | 31248204 | 0.7 | 0.5 | 1.0 | 0.03527 | 0.32 | 0.33 | 0.32 | SNP |
| chr6_32608946_G_A | 32608946 | 0.5 | 0.3 | 0.9 | 0.03532 | 0.09 | 0.04 | 0.09 | SNP |
| chr6_33033267_G_A | 33033267 | 0.6 | 0.4 | 0.9 | 0.03533 | 0.15 | 0.09 | 0.15 | SNP |
| chr6_29895626_T_A | 29895626 | 1.6 | 1.0 | 2.3 | 0.03536 | 0.15 | 0.17 | 0.15 | SNP |
| chr6_32612110_C_T | 32612110 | 0.7 | 0.5 | 1.0 | 0.03544 | 0.49 | 0.43 | 0.49 | SNP |
| chr6_31321681_C_T | 31321681 | 0.7 | 0.5 | 1.0 | 0.03548 | 0.20 | 0.21 | 0.20 | SNP |
| chr6_32035603_C_T | 32035603 | 0.5 | 0.2 | 0.9 | 0.03552 | 0.09 | 0.04 | 0.10 | SNP |
| chr6_29912766_T_A | 29912766 | 1.4 | 1.0 | 1.8 | 0.03560 | 0.48 | 0.50 | 0.48 | SNP |
| xHLA_2field_DQB1_06_03 | 32627244 | 0.5 | 0.2 | 0.9 | 0.03574 | 0.09 | 0.04 | 0.09 | HLA (xHLA) |
| chr6_31695368_G_A | 31695368 | 0.5 | 0.3 | 0.9 | 0.03577 | 0.07 | 0.07 | 0.08 | SNP |
| chr6_31466769_G_C | 31466769 | 0.7 | 0.5 | 1.0 | 0.03585 | 0.30 | 0.23 | 0.30 | SNP |
| chr6_32406611_C_A | 32406611 | 0.6 | 0.4 | 1.0 | 0.03587 | 0.22 | 0.17 | 0.23 | SNP |
| chr6_32609874_T_G | 32609874 | 0.5 | 0.3 | 0.9 | 0.03587 | 0.09 | 0.04 | 0.09 | SNP |
| chr6_32610075_A_G | 32610075 | 0.5 | 0.3 | 0.9 | 0.03587 | 0.09 | 0.04 | 0.09 | SNP |
| chr6_32610313_C_G | 32610313 | 0.5 | 0.3 | 0.9 | 0.03587 | 0.09 | 0.04 | 0.09 | SNP |

| | | | | | | | | | |
|---|---|---|---|---|---|---|---|---|---|
| chr6_29892196_T_A | 29892196 | 1.4 | 1.0 | 1.8 | 0.03590 | 0.47 | 0.47 | 0.47 | SNP |
| chr6_32608934_A_T | 32608934 | 0.5 | 0.3 | 0.9 | 0.03593 | 0.09 | 0.04 | 0.09 | SNP |
| chr6_32306480_A_T | 32306480 | 0.5 | 0.2 | 0.9 | 0.03620 | 0.07 | 0.03 | 0.07 | SNP |
| chr6_31239681_T_A | 31239681 | 1.5 | 1.0 | 2.3 | 0.03622 | 0.41 | 0.46 | 0.40 | SNP |
| chr6_31239378_G_T | 31239378 | 1.5 | 1.0 | 2.2 | 0.03626 | 0.50 | 0.52 | 0.50 | SNP |
| chr6_32635605_G_A | 32635605 | 1.4 | 1.0 | 2.0 | 0.03640 | 0.31 | 0.38 | 0.30 | SNP |
| chr6_31379109_G_A | 31379109 | 1.4 | 1.0 | 2.0 | 0.03641 | 0.24 | 0.24 | 0.24 | SNP |
| chr6_32076499_G_A | 32076499 | 0.7 | 0.5 | 1.0 | 0.03647 | 0.36 | 0.35 | 0.36 | SNP |
| chr6_31506744_C_A | 31506744 | 1.5 | 1.0 | 2.3 | 0.03649 | 0.23 | 0.29 | 0.23 | SNP |
| chr6_29856693_C_T | 29856693 | 0.8 | 0.6 | 1.0 | 0.03652 | 0.45 | 0.44 | 0.45 | SNP |
| chr6_29912463_G_A | 29912463 | 1.4 | 1.0 | 1.8 | 0.03666 | 0.49 | 0.50 | 0.49 | SNP |
| chr6_29895469_G_A | 29895469 | 1.6 | 1.0 | 2.4 | 0.03671 | 0.14 | 0.16 | 0.14 | SNP |
| chr6_32609195_G_A | 32609195 | 0.5 | 0.3 | 0.9 | 0.03672 | 0.09 | 0.04 | 0.09 | SNP |
| chr6_31378425_A_G | 31378425 | 1.4 | 1.0 | 2.0 | 0.03688 | 0.24 | 0.24 | 0.24 | SNP |
| chr6_29897259_T_A | 29897259 | 0.8 | 0.6 | 1.0 | 0.03691 | 0.44 | 0.44 | 0.44 | SNP |
| chr6_33045839_C_T | 33045839 | 0.5 | 0.2 | 0.9 | 0.03692 | 0.06 | 0.03 | 0.07 | SNP |
| chr6_29896084_A_G | 29896084 | 0.8 | 0.6 | 1.0 | 0.03701 | 0.46 | 0.46 | 0.46 | SNP |
| chr6_31745464_G_A | 31745464 | 2.3 | 1.0 | 5.2 | 0.03715 | 0.12 | 0.12 | 0.12 | SNP |
| chr6_32191457_G_A | 32191457 | 0.7 | 0.5 | 1.0 | 0.03721 | 0.19 | 0.13 | 0.19 | SNP |
| chr6_31383187_A_G | 31383187 | 1.4 | 1.0 | 2.0 | 0.03726 | 0.23 | 0.23 | 0.23 | SNP |
| chr6_32611215_C_T | 32611215 | 0.5 | 0.3 | 0.9 | 0.03741 | 0.09 | 0.04 | 0.09 | SNP |
| chr6_32611709_T_C | 32611709 | 0.5 | 0.3 | 0.9 | 0.03741 | 0.09 | 0.04 | 0.09 | SNP |
| chr6_29759729_C_T | 29759729 | 2.0 | 1.0 | 3.6 | 0.03744 | 0.03 | 0.05 | 0.02 | SNP |
| chr6_29795113_C_G | 29795113 | 2.0 | 1.0 | 3.6 | 0.03746 | 0.03 | 0.05 | 0.02 | SNP |
| chr6_33033390_A_T | 33033390 | 0.6 | 0.4 | 0.9 | 0.03758 | 0.15 | 0.09 | 0.15 | SNP |
| chr6_29940943_T_C | 29940943 | 0.7 | 0.6 | 1.0 | 0.03761 | 0.36 | 0.36 | 0.36 | SNP |
| chr6_32339925_T_A | 32339925 | 1.3 | 1.0 | 1.8 | 0.03768 | 0.33 | 0.35 | 0.33 | SNP |
| chr6_32610688_G_C | 32610688 | 0.5 | 0.3 | 0.9 | 0.03768 | 0.09 | 0.04 | 0.09 | SNP |
| chr6_32408842_A_G | 32408842 | 0.6 | 0.4 | 1.0 | 0.03779 | 0.22 | 0.17 | 0.23 | SNP |
| chr6_33037560_G_A | 33037560 | 0.6 | 0.3 | 0.9 | 0.03794 | 0.12 | 0.07 | 0.12 | SNP |
| chr6_32610696_G_A | 32610696 | 0.5 | 0.3 | 0.9 | 0.03814 | 0.09 | 0.04 | 0.09 | SNP |
| chr6_33053468_G_T | 33053468 | 0.7 | 0.5 | 1.0 | 0.03820 | 0.27 | 0.22 | 0.28 | SNP |
| chr6_32310863_T_C | 32310863 | 2.2 | 1.0 | 4.6 | 0.03832 | 0.12 | 0.12 | 0.12 | SNP |
| chr6_31383108_T_C | 31383108 | 1.4 | 1.0 | 2.0 | 0.03846 | 0.23 | 0.23 | 0.23 | SNP |
| chr6_32411376_T_G | 32411376 | 0.6 | 0.4 | 1.0 | 0.03848 | 0.22 | 0.17 | 0.23 | SNP |
| chr6_33036505_G_C | 33036505 | 0.6 | 0.4 | 1.0 | 0.03855 | 0.15 | 0.09 | 0.15 | SNP |
| chr6_32193220_A_G | 32193220 | 0.7 | 0.5 | 1.0 | 0.03857 | 0.33 | 0.26 | 0.34 | SNP |
| chr6_32193698_A_T | 32193698 | 0.7 | 0.5 | 1.0 | 0.03857 | 0.33 | 0.26 | 0.34 | SNP |
| chr6_32192978_T_A | 32192978 | 0.7 | 0.5 | 1.0 | 0.03863 | 0.30 | 0.24 | 0.30 | SNP |
| chr6_29896108_A_T | 29896108 | 1.6 | 1.0 | 2.4 | 0.03871 | 0.15 | 0.17 | 0.15 | SNP |
| chr6_32610300_C_T | 32610300 | 0.5 | 0.3 | 0.9 | 0.03875 | 0.09 | 0.04 | 0.09 | SNP |
| chr6_31850813_T_C | 31850813 | 0.4 | 0.1 | 0.9 | 0.03888 | 0.05 | 0.02 | 0.05 | SNP |
| chr6_33045838_G_A | 33045838 | 0.5 | 0.2 | 0.9 | 0.03889 | 0.06 | 0.03 | 0.07 | SNP |
| chr6_33158020_C_T | 33158020 | 1.6 | 1.0 | 2.4 | 0.03921 | 0.12 | 0.14 | 0.12 | SNP |
| chr6_29896275_G_A | 29896275 | 0.7 | 0.6 | 1.0 | 0.03922 | 0.46 | 0.45 | 0.46 | SNP |
| chr6_32377116_C_G | 32377116 | 1.5 | 1.0 | 2.2 | 0.03926 | 0.48 | 0.54 | 0.47 | SNP |
| chr6_32005048_C_G | 32005048 | 0.4 | 0.1 | 0.9 | 0.03974 | 0.04 | 0.02 | 0.04 | SNP |
| chr6_32786516_C_T | 32786516 | 0.5 | 0.3 | 0.9 | 0.03984 | 0.08 | 0.04 | 0.08 | SNP |
| chr6_29909391_C_A | 29909391 | 1.6 | 1.0 | 2.4 | 0.03986 | 0.08 | 0.11 | 0.08 | SNP |
| chr6_29909409_C_T | 29909409 | 1.6 | 1.0 | 2.4 | 0.03986 | 0.08 | 0.11 | 0.08 | SNP |
| chr6_31508761_T_C | 31508761 | 1.5 | 1.0 | 2.3 | 0.03989 | 0.24 | 0.29 | 0.23 | SNP |
| chr6_32490708_A_C | 32490708 | 2.4 | 1.0 | 5.2 | 0.03993 | 0.02 | 0.03 | 0.01 | SNP |
| chr6_31657754_C_G | 31657754 | 1.4 | 1.0 | 1.9 | 0.04001 | 0.27 | 0.31 | 0.27 | SNP |
| chr6_31601735_G_A | 31601735 | 0.5 | 0.2 | 0.9 | 0.04006 | 0.06 | 0.03 | 0.06 | SNP |
| chr6_32608967_A_G | 32608967 | 0.5 | 0.3 | 0.9 | 0.04010 | 0.09 | 0.05 | 0.10 | SNP |
| chr6_29909426_T_C | 29909426 | 1.6 | 1.0 | 2.4 | 0.04012 | 0.08 | 0.11 | 0.08 | SNP |
| chr6_31383083_G_A | 31383083 | 1.4 | 1.0 | 2.0 | 0.04023 | 0.23 | 0.23 | 0.23 | SNP |
| chr6_32783406_G_A | 32783406 | 0.5 | 0.3 | 0.9 | 0.04026 | 0.08 | 0.04 | 0.08 | SNP |
| chr6_32783450_C_G | 32783450 | 0.5 | 0.3 | 0.9 | 0.04026 | 0.08 | 0.04 | 0.08 | SNP |
| chr6_31383071_A_G | 31383071 | 1.4 | 1.0 | 2.0 | 0.04026 | 0.23 | 0.23 | 0.23 | SNP |
| chr6_32409242_C_A | 32409242 | 0.7 | 0.5 | 1.0 | 0.04038 | 0.44 | 0.32 | 0.45 | SNP |
| chr6_33033235_G_C | 33033235 | 0.6 | 0.4 | 1.0 | 0.04043 | 0.15 | 0.09 | 0.15 | SNP |
| chr6_33077588_A_G | 33077588 | 1.9 | 1.0 | 3.5 | 0.04052 | 0.06 | 0.07 | 0.06 | SNP |
| chr6_32006791_G_A | 32006791 | 0.4 | 0.1 | 0.9 | 0.04053 | 0.04 | 0.02 | 0.04 | SNP |
| chr6_33037878_G_A | 33037878 | 0.6 | 0.4 | 1.0 | 0.04071 | 0.14 | 0.08 | 0.14 | SNP |
| chr6_31723088_C_T | 31723088 | 2.4 | 1.0 | 5.5 | 0.04105 | 0.01 | 0.03 | 0.01 | SNP |
| chr6_31322047_A_G | 31322047 | 0.7 | 0.4 | 1.0 | 0.04108 | 0.15 | 0.14 | 0.16 | SNP |
| chr6_29895945_A_T | 29895945 | 2.6 | 0.9 | 6.2 | 0.04135 | 0.01 | 0.02 | 0.01 | SNP |
| chr6_33045835_A_G | 33045835 | 0.5 | 0.2 | 0.9 | 0.04145 | 0.06 | 0.03 | 0.07 | SNP |
| chr6_31935567_G_A | 31935567 | 0.4 | 0.2 | 0.9 | 0.04172 | 0.05 | 0.02 | 0.05 | SNP |
| chr6_31708910_T_C | 31708910 | 0.6 | 0.3 | 1.0 | 0.04181 | 0.07 | 0.07 | 0.07 | SNP |
| chr6_32725832_A_G | 32725832 | 0.6 | 0.4 | 1.0 | 0.04187 | 0.21 | 0.14 | 0.21 | SNP |
| chr6_32005664_C_T | 32005664 | 1.8 | 1.0 | 3.1 | 0.04196 | 0.06 | 0.06 | 0.06 | SNP |
| chr6_32635629_G_A | 32635629 | 1.6 | 1.0 | 2.3 | 0.04213 | 0.22 | 0.29 | 0.22 | SNP |
| chr6_32192331_G_A | 32192331 | 1.4 | 1.0 | 1.9 | 0.04215 | 0.47 | 0.57 | 0.46 | SNP |
| chr6_31542476_C_A | 31542476 | 0.7 | 0.5 | 1.0 | 0.04226 | 0.16 | 0.14 | 0.16 | SNP |
| chr6_29858253_G_A | 29858253 | 1.6 | 1.0 | 2.4 | 0.04237 | 0.14 | 0.16 | 0.14 | SNP |
| chr6_32376788_C_T | 32376788 | 1.4 | 1.0 | 2.0 | 0.04246 | 0.45 | 0.52 | 0.44 | SNP |
| chr6_32782149_G_A | 32782149 | 0.5 | 0.3 | 0.9 | 0.04258 | 0.08 | 0.04 | 0.08 | SNP |
| chr6_33036177_A_G | 33036177 | 0.6 | 0.4 | 1.0 | 0.04287 | 0.15 | 0.09 | 0.15 | SNP |
| chr6_32297209_G_A | 32297209 | 0.5 | 0.2 | 0.9 | 0.04311 | 0.07 | 0.04 | 0.07 | SNP |
| chr6_32816958_A_G | 32816958 | 1.3 | 1.0 | 1.8 | 0.04355 | 0.38 | 0.40 | 0.37 | SNP |
| chr6_29896999_C_T | 29896999 | 1.6 | 1.0 | 2.5 | 0.04386 | 0.15 | 0.17 | 0.15 | SNP |
| chr6_29942045_G_T | 29942045 | 0.7 | 0.5 | 1.0 | 0.04411 | 0.34 | 0.34 | 0.34 | SNP |
| chr6_31464739_G_A | 31464739 | 0.7 | 0.5 | 1.0 | 0.04413 | 0.30 | 0.24 | 0.30 | SNP |
| chr6_29942673_T_G | 29942673 | 0.7 | 0.6 | 1.0 | 0.04414 | 0.35 | 0.35 | 0.35 | SNP |
| chr6_29973526_T_C | 29973526 | 0.7 | 0.6 | 1.0 | 0.04415 | 0.35 | 0.35 | 0.35 | SNP |
| chr6_32731313_T_A | 32731313 | 0.6 | 0.4 | 1.0 | 0.04430 | 0.21 | 0.14 | 0.21 | SNP |
| chr6_33039484_A_G | 33039484 | 0.6 | 0.4 | 1.0 | 0.04443 | 0.14 | 0.09 | 0.15 | SNP |
| chr6_31948400_T_G | 31948400 | 0.4 | 0.1 | 0.9 | 0.04460 | 0.04 | 0.02 | 0.04 | SNP |
| chr6_33053477_G_C | 33053477 | 0.7 | 0.5 | 1.0 | 0.04469 | 0.27 | 0.22 | 0.28 | SNP |
| chr6_33476718_T_C | 33476718 | 0.8 | 0.6 | 1.0 | 0.04469 | 0.35 | 0.30 | 0.36 | SNP |
| chr6_29942083_C_T | 29942083 | 0.7 | 0.6 | 1.0 | 0.04484 | 0.35 | 0.35 | 0.35 | SNP |
| chr6_32797809_C_T | 32797809 | 1.5 | 1.0 | 2.1 | 0.04490 | 0.11 | 0.16 | 0.11 | SNP |
| chr6_31785228_C_G | 31785228 | 0.6 | 0.4 | 1.0 | 0.04507 | 0.28 | 0.23 | 0.28 | SNP |
| chr6_33045896_A_G | 33045896 | 0.4 | 0.1 | 0.9 | 0.04519 | 0.04 | 0.01 | 0.04 | SNP |
| chr6_32439753_T_A | 32439753 | 0.7 | 0.5 | 1.0 | 0.04520 | 0.31 | 0.24 | 0.32 | SNP |
| chr6_33033313_G_T | 33033313 | 0.6 | 0.4 | 1.0 | 0.04526 | 0.12 | 0.07 | 0.12 | SNP |
| chr6_32608350_G_A | 32608350 | 0.5 | 0.3 | 0.9 | 0.04535 | 0.10 | 0.05 | 0.10 | SNP |
| chr6_29941431_G_A | 29941431 | 0.7 | 0.6 | 1.0 | 0.04536 | 0.35 | 0.35 | 0.35 | SNP |
| chr6_29891825_T_C | 29891825 | 1.3 | 1.0 | 1.7 | 0.04552 | 0.48 | 0.48 | 0.48 | SNP |
| chr6_29892217_T_C | 29892217 | 1.4 | 1.0 | 1.9 | 0.04555 | 0.25 | 0.28 | 0.25 | SNP |
| chr6_31540141_C_A | 31540141 | 1.4 | 1.0 | 1.9 | 0.04560 | 0.38 | 0.44 | 0.38 | SNP |
| chr6_32151420_G_C | 32151420 | 0.5 | 0.3 | 0.9 | 0.04586 | 0.07 | 0.04 | 0.07 | SNP |
| chr6_33045891_C_T | 33045891 | 0.4 | 0.1 | 0.9 | 0.04606 | 0.04 | 0.01 | 0.04 | SNP |
| chr6_32826450_T_C | 32826450 | 0.7 | 0.6 | 1.0 | 0.04624 | 0.45 | 0.39 | 0.46 | SNP |
| chr6_29896967_C_T | 29896967 | 1.4 | 1.0 | 2.1 | 0.04642 | 0.19 | 0.22 | 0.19 | SNP |
| chr6_31906865_C_T | 31906865 | 0.5 | 0.3 | 1.0 | 0.04646 | 0.09 | 0.04 | 0.09 | SNP |
| chr6_29942842_C_A | 29942842 | 0.7 | 0.5 | 1.0 | 0.04661 | 0.34 | 0.34 | 0.34 | SNP |
| chr6_31905130_G_A | 31905130 | 0.5 | 0.3 | 1.0 | 0.04667 | 0.09 | 0.04 | 0.09 | SNP |
| chr6_31905328_G_T | 31905328 | 0.5 | 0.3 | 1.0 | 0.04667 | 0.09 | 0.04 | 0.09 | SNP |
| chr6_31907147_T_C | 31907147 | 0.5 | 0.3 | 1.0 | 0.04667 | 0.09 | 0.04 | 0.09 | SNP |
| chr6_31907168_C_T | 31907168 | 0.5 | 0.3 | 1.0 | 0.04667 | 0.09 | 0.04 | 0.09 | SNP |
| chr6_31365707_G_A | 31365707 | 0.7 | 0.5 | 1.0 | 0.04671 | 0.34 | 0.32 | 0.34 | SNP |
| chr6_32303692_T_A | 32303692 | 2.1 | 1.0 | 4.4 | 0.04676 | 0.12 | 0.12 | 0.12 | SNP |
| chr6_31475127_G_A | 31475127 | 1.4 | 1.0 | 2.1 | 0.04703 | 0.28 | 0.32 | 0.28 | SNP |
| chr6_32188713_C_G | 32188713 | 0.4 | 0.1 | 0.9 | 0.04716 | 0.04 | 0.01 | 0.04 | SNP |
| chr6_33036951_A_G | 33036951 | 0.6 | 0.4 | 1.0 | 0.04719 | 0.14 | 0.09 | 0.14 | SNP |
| chr6_29913374_C_G | 29913374 | 1.6 | 1.0 | 2.5 | 0.04721 | 0.13 | 0.15 | 0.13 | SNP |
| chr6_31239869_T_C | 31239869 | 0.7 | 0.5 | 1.0 | 0.04725 | 0.30 | 0.24 | 0.30 | SNP |
| chr6_33053270_T_A | 33053270 | 0.7 | 0.5 | 1.0 | 0.04737 | 0.27 | 0.22 | 0.28 | SNP |
| chr6_31379620_G_A | 31379620 | 1.4 | 1.0 | 1.9 | 0.04760 | 0.24 | 0.24 | 0.24 | SNP |
| chr6_32083175_C_T | 32083175 | 0.7 | 0.5 | 1.0 | 0.04771 | 0.29 | 0.29 | 0.29 | SNP |
| chr6_33282338_G_A | 33282338 | 0.7 | 0.5 | 1.0 | 0.04773 | 0.17 | 0.13 | 0.18 | SNP |
| chr6_32822186_T_G | 32822186 | 0.5 | 0.2 | 0.9 | 0.04778 | 0.05 | 0.04 | 0.06 | SNP |
| chr6_32145707_C_A | 32145707 | 1.4 | 1.0 | 1.9 | 0.04782 | 0.43 | 0.46 | 0.43 | SNP |
| chr6_32635687_T_C | 32635687 | 1.4 | 1.0 | 1.9 | 0.04789 | 0.37 | 0.44 | 0.37 | SNP |
| chr6_32812858_A_G | 32812858 | 0.7 | 0.4 | 1.0 | 0.04797 | 0.15 | 0.11 | 0.16 | SNP |
| chr6_31722262_C_T | 31722262 | 2.4 | 0.9 | 5.2 | 0.04829 | 0.01 | 0.03 | 0.01 | SNP |
| chr6_31732207_C_T | 31732207 | 2.4 | 0.9 | 5.2 | 0.04829 | 0.01 | 0.03 | 0.01 | SNP |
| chr6_29941829_A_T | 29941829 | 0.8 | 0.6 | 1.0 | 0.04876 | 0.35 | 0.35 | 0.35 | SNP |
| chr6_32732677_A_T | 32732677 | 1.3 | 1.0 | 1.8 | 0.04920 | 0.31 | 0.33 | 0.31 | SNP |
| chr6_31539768_G_A | 31539768 | 1.4 | 1.0 | 1.8 | 0.04938 | 0.35 | 0.40 | 0.34 | SNP |
| chr6_31382621_C_T | 31382621 | 1.4 | 1.0 | 2.0 | 0.04939 | 0.23 | 0.23 | 0.23 | SNP |
| chr6_29942051_T_A | 29942051 | 0.7 | 0.5 | 1.0 | 0.04959 | 0.34 | 0.34 | 0.34 | SNP |
| chr6_32134828_C_T | 32134828 | 0.4 | 0.1 | 0.9 | 0.04966 | 0.04 | 0.02 | 0.05 | SNP |
| chr6_29769032_C_A | 29769032 | 1.9 | 1.0 | 3.6 | 0.04973 | 0.03 | 0.05 | 0.02 | SNP |
| chr6_31848440_A_T | 31848440 | 0.6 | 0.3 | 1.0 | 0.04977 | 0.10 | 0.09 | 0.10 | SNP |
| chr6_33037304_G_T | 33037304 | 0.6 | 0.4 | 1.0 | 0.04978 | 0.14 | 0.09 | 0.15 | SNP |
| chr6_29896925_C_G | 29896925 | 1.3 | 1.0 | 1.8 | 0.04987 | 0.35 | 0.37 | 0.35 | SNP |

ANCA = anitneutrophil cytoplasmic antibody; AAV = ANCA-associated vasculitis; MPA = microscopic polyangiitis; SNP = single nucleotide polymorphism; OR = Odds ratio; CI = confidence interval; MAF = minor allele frequency. SNPs and HLA alleles with P < 0.05 included in the table.

**Supplemtary Table 6**. Association analysis of pulmonary involvement in all AAV patients versus controls, adjusting for sex and genetic structure (principal components 1-4).

| Variant | position | OR | CI_lower | CI_upper | p | MAF | MAF_cases | MAF_controls | Type (SNP, HLA (xHLA)) |
|---|---|---|---|---|---|---|---|---|---|
| chr6_32943151_C_T | 32943151 | 1.70 | 1.32 | 2.20 | 5.35E-05 | 0.39 | 0.44 | 0.34 | SNP |
| chr6_32943345_T_C | 32943345 | 1.61 | 1.26 | 2.08 | 0.00018 | 0.44 | 0.48 | 0.39 | SNP |
| chr6_31323416_G_C | 31323416 | 0.27 | 0.12 | 0.56 | 0.00059 | 0.13 | 0.13 | 0.15 | SNP |
| chr6_32942302_A_G | 32942302 | 1.58 | 1.22 | 2.05 | 0.00066 | 0.39 | 0.43 | 0.35 | SNP |
| chr6_31323506_C_T | 31323506 | 0.28 | 0.13 | 0.58 | 0.00088 | 0.13 | 0.13 | 0.14 | SNP |
| chr6_32012817_C_T | 32012817 | 2.48 | 1.47 | 4.31 | 0.00091 | 0.08 | 0.11 | 0.06 | SNP |
| chr6_31322790_T_A | 31322790 | 0.29 | 0.14 | 0.61 | 0.00115 | 0.13 | 0.12 | 0.14 | SNP |
| chr6_31129310_C_T | 31129310 | 0.31 | 0.15 | 0.65 | 0.00234 | 0.13 | 0.12 | 0.14 | SNP |
| chr6_31430010_G_A | 31430010 | 0.30 | 0.13 | 0.66 | 0.00322 | 0.13 | 0.12 | 0.14 | SNP |
| chr6_33048640_A_G | 33048640 | 0.51 | 0.32 | 0.80 | 0.00372 | 0.10 | 0.07 | 0.13 | SNP |
| chr6_31239227_T_C | 31239227 | 0.50 | 0.31 | 0.80 | 0.00383 | 0.39 | 0.37 | 0.42 | SNP |
| chr6_32943407_A_T | 32943407 | 0.39 | 0.20 | 0.73 | 0.00395 | 0.08 | 0.07 | 0.10 | SNP |
| chr6_31430065_C_T | 31430065 | 0.32 | 0.14 | 0.69 | 0.00417 | 0.13 | 0.12 | 0.14 | SNP |
| chr6_33048663_G_A | 33048663 | 0.49 | 0.30 | 0.80 | 0.00486 | 0.08 | 0.06 | 0.11 | SNP |
| chr6_31326703_T_C | 31326703 | 0.33 | 0.15 | 0.71 | 0.00499 | 0.13 | 0.13 | 0.14 | SNP |
| chr6_33050078_A_G | 33050078 | 0.57 | 0.38 | 0.84 | 0.00559 | 0.10 | 0.07 | 0.13 | SNP |
| chr6_33050079_C_G | 33050079 | 0.57 | 0.38 | 0.84 | 0.00559 | 0.10 | 0.07 | 0.13 | SNP |
| chr6_31326074_A_T | 31326074 | 0.34 | 0.15 | 0.72 | 0.00561 | 0.13 | 0.12 | 0.13 | SNP |
| chr6_33048628_A_C | 33048628 | 0.47 | 0.27 | 0.80 | 0.00618 | 0.07 | 0.05 | 0.09 | SNP |
| chr6_33048661_A_G | 33048661 | 0.50 | 0.30 | 0.81 | 0.00620 | 0.08 | 0.06 | 0.11 | SNP |
| chr6_33545340_G_A | 33545340 | 0.64 | 0.46 | 0.88 | 0.00690 | 0.23 | 0.19 | 0.27 | SNP |
| chr6_33071777_A_G | 33071777 | 0.55 | 0.35 | 0.84 | 0.00699 | 0.09 | 0.06 | 0.12 | SNP |
| chr6_32944746_G_A | 32944746 | 0.71 | 0.55 | 0.91 | 0.00711 | 0.46 | 0.43 | 0.50 | SNP |
| chr6_32946133_T_C | 32946133 | 0.71 | 0.55 | 0.91 | 0.00755 | 0.46 | 0.43 | 0.50 | SNP |
| chr6_33050089_G_A | 33050089 | 0.58 | 0.39 | 0.86 | 0.00798 | 0.10 | 0.07 | 0.12 | SNP |
| chr6_33055079_A_G | 33055079 | 0.59 | 0.39 | 0.87 | 0.00819 | 0.10 | 0.07 | 0.13 | SNP |
| chr6_32939894_T_G | 32939894 | 1.90 | 1.19 | 3.08 | 0.00819 | 0.12 | 0.14 | 0.10 | SNP |
| chr6_33050107_G_A | 33050107 | 0.58 | 0.39 | 0.87 | 0.00843 | 0.10 | 0.07 | 0.12 | SNP |
| chr6_33047432_A_C | 33047432 | 0.60 | 0.40 | 0.87 | 0.00846 | 0.10 | 0.08 | 0.13 | SNP |
| chr6_33053455_C_T | 33053455 | 0.58 | 0.38 | 0.87 | 0.00885 | 0.10 | 0.07 | 0.12 | SNP |
| xHLA_2field_DQB1_03_03 | 32627244 | 2.46 | 1.29 | 5.04 | 0.00893 | 0.05 | 0.07 | 0.03 | HLA (xHLA) |
| chr6_31465661_T_G | 31465661 | 1.46 | 1.10 | 1.95 | 0.00951 | 0.38 | 0.41 | 0.34 | SNP |
| chr6_33071754_C_A | 33071754 | 0.57 | 0.37 | 0.87 | 0.00966 | 0.09 | 0.07 | 0.12 | SNP |
| chr6_32917544_C_T | 32917544 | 1.78 | 1.16 | 2.80 | 0.01008 | 0.09 | 0.11 | 0.07 | SNP |
| chr6_33050279_A_G | 33050279 | 0.61 | 0.41 | 0.89 | 0.01125 | 0.10 | 0.08 | 0.13 | SNP |
| chr6_33049211_C_T | 33049211 | 0.61 | 0.41 | 0.89 | 0.01133 | 0.10 | 0.08 | 0.13 | SNP |
| chr6_33050232_T_G | 33050232 | 0.60 | 0.40 | 0.89 | 0.01140 | 0.10 | 0.07 | 0.12 | SNP |
| chr6_33050148_T_C | 33050148 | 0.59 | 0.38 | 0.88 | 0.01173 | 0.09 | 0.07 | 0.12 | SNP |
| chr6_33048599_C_A | 33048599 | 0.70 | 0.53 | 0.92 | 0.01224 | 0.29 | 0.25 | 0.33 | SNP |
| chr6_31669496_C_T | 31669496 | 1.76 | 1.14 | 2.77 | 0.01269 | 0.09 | 0.12 | 0.07 | SNP |
| chr6_33166034_A_G | 33166034 | 0.59 | 0.38 | 0.89 | 0.01274 | 0.10 | 0.08 | 0.12 | SNP |
| chr6_33046752_G_C | 33046752 | 0.51 | 0.30 | 0.86 | 0.01274 | 0.06 | 0.04 | 0.08 | SNP |
| chr6_33055123_A_G | 33055123 | 0.61 | 0.40 | 0.90 | 0.01283 | 0.10 | 0.08 | 0.13 | SNP |
| xHLA_2field_DPB1_04_01 | 33043767 | 1.33 | 1.06 | 1.68 | 0.01283 | 0.34 | 0.30 | 0.38 | HLA (xHLA) |
| chr6_33071708_G_A | 33071708 | 0.58 | 0.37 | 0.88 | 0.01286 | 0.09 | 0.07 | 0.11 | SNP |
| chr6_31473746_G_A | 31473746 | 0.38 | 0.18 | 0.81 | 0.01286 | 0.13 | 0.13 | 0.14 | SNP |
| chr6_32627818_C_A | 32627818 | 0.66 | 0.47 | 0.91 | 0.01290 | 0.28 | 0.26 | 0.31 | SNP |
| chr6_31636742_A_G | 31636742 | 0.37 | 0.17 | 0.81 | 0.01306 | 0.12 | 0.12 | 0.13 | SNP |
| chr6_33048380_G_A | 33048380 | 0.51 | 0.30 | 0.86 | 0.01308 | 0.06 | 0.04 | 0.08 | SNP |
| chr6_33050223_G_A | 33050223 | 0.61 | 0.41 | 0.90 | 0.01309 | 0.10 | 0.08 | 0.12 | SNP |
| chr6_33050024_C_A | 33050024 | 0.61 | 0.41 | 0.90 | 0.01314 | 0.10 | 0.08 | 0.12 | SNP |
| chr6_33050045_T_C | 33050045 | 0.61 | 0.41 | 0.90 | 0.01314 | 0.10 | 0.08 | 0.12 | SNP |
| chr6_33050179_A_G | 33050179 | 0.61 | 0.40 | 0.90 | 0.01332 | 0.10 | 0.08 | 0.12 | SNP |
| chr6_33054659_A_G | 33054659 | 0.61 | 0.41 | 0.90 | 0.01339 | 0.10 | 0.08 | 0.12 | SNP |
| chr6_33050168_C_A | 33050168 | 0.61 | 0.40 | 0.90 | 0.01348 | 0.10 | 0.08 | 0.12 | SNP |
| chr6_33047031_T_C | 33047031 | 0.61 | 0.41 | 0.90 | 0.01354 | 0.10 | 0.08 | 0.12 | SNP |
| chr6_33053477_G_C | 33053477 | 0.60 | 0.39 | 0.90 | 0.01384 | 0.09 | 0.07 | 0.12 | SNP |
| chr6_33053307_C_T | 33053307 | 0.60 | 0.40 | 0.90 | 0.01399 | 0.10 | 0.07 | 0.12 | SNP |
| chr6_33050185_C_A | 33050185 | 0.61 | 0.41 | 0.90 | 0.01402 | 0.10 | 0.08 | 0.12 | SNP |
| chr6_33050125_G_A | 33050125 | 0.60 | 0.39 | 0.90 | 0.01433 | 0.09 | 0.07 | 0.12 | SNP |
| chr6_33071600_C_A | 33071600 | 0.59 | 0.38 | 0.89 | 0.01437 | 0.09 | 0.07 | 0.11 | SNP |
| chr6_33160425_G_T | 33160425 | 0.60 | 0.40 | 0.90 | 0.01441 | 0.09 | 0.07 | 0.12 | SNP |
| chr6_31085356_C_T | 31085356 | 0.41 | 0.19 | 0.83 | 0.01467 | 0.13 | 0.13 | 0.13 | SNP |
| chr6_33053682_G_A | 33053682 | 0.62 | 0.42 | 0.91 | 0.01479 | 0.10 | 0.08 | 0.12 | SNP |
| chr6_33053772_G_A | 33053772 | 0.62 | 0.42 | 0.91 | 0.01479 | 0.10 | 0.08 | 0.12 | SNP |
| chr6_33053780_T_A | 33053780 | 0.62 | 0.42 | 0.91 | 0.01479 | 0.10 | 0.08 | 0.12 | SNP |
| chr6_33053789_A_G | 33053789 | 0.62 | 0.42 | 0.91 | 0.01479 | 0.10 | 0.08 | 0.12 | SNP |
| chr6_33053868_A_G | 33053868 | 0.62 | 0.42 | 0.91 | 0.01479 | 0.10 | 0.08 | 0.12 | SNP |
| chr6_33053871_A_G | 33053871 | 0.62 | 0.42 | 0.91 | 0.01479 | 0.10 | 0.08 | 0.12 | SNP |
| chr6_33053877_G_A | 33053877 | 0.62 | 0.42 | 0.91 | 0.01479 | 0.10 | 0.08 | 0.12 | SNP |
| chr6_33053887_A_G | 33053887 | 0.62 | 0.42 | 0.91 | 0.01479 | 0.10 | 0.08 | 0.12 | SNP |
| chr6_33053890_G_A | 33053890 | 0.62 | 0.42 | 0.91 | 0.01479 | 0.10 | 0.08 | 0.12 | SNP |
| chr6_33053982_G_C | 33053982 | 0.62 | 0.42 | 0.91 | 0.01479 | 0.10 | 0.08 | 0.12 | SNP |
| chr6_33054091_C_T | 33054091 | 0.62 | 0.42 | 0.91 | 0.01479 | 0.10 | 0.08 | 0.12 | SNP |
| chr6_33054141_T_C | 33054141 | 0.62 | 0.42 | 0.91 | 0.01479 | 0.10 | 0.08 | 0.12 | SNP |
| chr6_33054142_G_C | 33054142 | 0.62 | 0.42 | 0.91 | 0.01479 | 0.10 | 0.08 | 0.12 | SNP |
| chr6_33054148_C_T | 33054148 | 0.62 | 0.42 | 0.91 | 0.01479 | 0.10 | 0.08 | 0.12 | SNP |
| chr6_33054149_T_C | 33054149 | 0.62 | 0.42 | 0.91 | 0.01479 | 0.10 | 0.08 | 0.12 | SNP |
| chr6_33054152_A_G | 33054152 | 0.62 | 0.42 | 0.91 | 0.01479 | 0.10 | 0.08 | 0.12 | SNP |
| chr6_33054155_C_T | 33054155 | 0.62 | 0.42 | 0.91 | 0.01479 | 0.10 | 0.08 | 0.12 | SNP |
| chr6_33054156_A_G | 33054156 | 0.62 | 0.42 | 0.91 | 0.01479 | 0.10 | 0.08 | 0.12 | SNP |
| chr6_33054231_C_T | 33054231 | 0.62 | 0.42 | 0.91 | 0.01479 | 0.10 | 0.08 | 0.12 | SNP |
| chr6_33054235_T_G | 33054235 | 0.62 | 0.42 | 0.91 | 0.01479 | 0.10 | 0.08 | 0.12 | SNP |
| chr6_33054268_C_A | 33054268 | 0.62 | 0.42 | 0.91 | 0.01479 | 0.10 | 0.08 | 0.12 | SNP |
| chr6_33054280_T_C | 33054280 | 0.62 | 0.42 | 0.91 | 0.01479 | 0.10 | 0.08 | 0.12 | SNP |
| chr6_33054281_G_A | 33054281 | 0.62 | 0.42 | 0.91 | 0.01479 | 0.10 | 0.08 | 0.12 | SNP |
| chr6_33054302_T_C | 33054302 | 0.62 | 0.42 | 0.91 | 0.01479 | 0.10 | 0.08 | 0.12 | SNP |
| chr6_33054325_G_C | 33054325 | 0.62 | 0.42 | 0.91 | 0.01479 | 0.10 | 0.08 | 0.12 | SNP |
| chr6_33054331_T_C | 33054331 | 0.62 | 0.42 | 0.91 | 0.01479 | 0.10 | 0.08 | 0.12 | SNP |
| chr6_33054457_A_G | 33054457 | 0.62 | 0.42 | 0.91 | 0.01479 | 0.10 | 0.08 | 0.12 | SNP |
| chr6_33054550_G_A | 33054550 | 0.62 | 0.42 | 0.91 | 0.01479 | 0.10 | 0.08 | 0.12 | SNP |
| chr6_33054579_C_T | 33054579 | 0.62 | 0.42 | 0.91 | 0.01479 | 0.10 | 0.08 | 0.12 | SNP |
| chr6_33054586_A_G | 33054586 | 0.62 | 0.42 | 0.91 | 0.01479 | 0.10 | 0.08 | 0.12 | SNP |
| chr6_33054595_C_T | 33054595 | 0.62 | 0.42 | 0.91 | 0.01479 | 0.10 | 0.08 | 0.12 | SNP |
| chr6_33054619_G_A | 33054619 | 0.62 | 0.42 | 0.91 | 0.01479 | 0.10 | 0.08 | 0.12 | SNP |
| chr6_33054656_A_C | 33054656 | 0.62 | 0.42 | 0.91 | 0.01479 | 0.10 | 0.08 | 0.12 | SNP |
| chr6_33054675_C_G | 33054675 | 0.62 | 0.42 | 0.91 | 0.01479 | 0.10 | 0.08 | 0.12 | SNP |
| chr6_33054683_A_G | 33054683 | 0.62 | 0.42 | 0.91 | 0.01479 | 0.10 | 0.08 | 0.12 | SNP |
| chr6_33054687_G_A | 33054687 | 0.62 | 0.42 | 0.91 | 0.01479 | 0.10 | 0.08 | 0.12 | SNP |
| chr6_33054711_G_T | 33054711 | 0.62 | 0.42 | 0.91 | 0.01479 | 0.10 | 0.08 | 0.12 | SNP |
| chr6_33054721_C_T | 33054721 | 0.62 | 0.42 | 0.91 | 0.01479 | 0.10 | 0.08 | 0.12 | SNP |
| chr6_33054807_A_G | 33054807 | 0.62 | 0.42 | 0.91 | 0.01479 | 0.10 | 0.08 | 0.12 | SNP |
| chr6_33054890_C_T | 33054890 | 0.62 | 0.42 | 0.91 | 0.01479 | 0.10 | 0.08 | 0.12 | SNP |
| chr6_31237605_G_A | 31237605 | 1.45 | 1.08 | 1.96 | 0.01496 | 0.22 | 0.25 | 0.19 | SNP |
| chr6_31240959_C_G | 31240959 | 1.43 | 1.07 | 1.91 | 0.01498 | 0.24 | 0.27 | 0.21 | SNP |
| chr6_33143756_A_G | 33143756 | 0.61 | 0.41 | 0.91 | 0.01498 | 0.10 | 0.08 | 0.12 | SNP |
| chr6_33055009_C_A | 33055009 | 0.62 | 0.42 | 0.91 | 0.01501 | 0.10 | 0.08 | 0.13 | SNP |
| chr6_33047466_T_C | 33047466 | 0.57 | 0.36 | 0.89 | 0.01513 | 0.07 | 0.06 | 0.10 | SNP |
| chr6_33046726_T_C | 33046726 | 0.54 | 0.33 | 0.88 | 0.01544 | 0.06 | 0.05 | 0.08 | SNP |
| chr6_33047612_G_C | 33047612 | 0.54 | 0.33 | 0.88 | 0.01544 | 0.06 | 0.05 | 0.08 | SNP |
| chr6_33050118_C_T | 33050118 | 0.60 | 0.40 | 0.90 | 0.01544 | 0.09 | 0.07 | 0.12 | SNP |
| chr6_33178010_G_T | 33178010 | 0.61 | 0.41 | 0.91 | 0.01563 | 0.10 | 0.08 | 0.13 | SNP |
| chr6_33055047_A_G | 33055047 | 0.62 | 0.42 | 0.91 | 0.01565 | 0.10 | 0.08 | 0.12 | SNP |
| chr6_33046915_T_A | 33046915 | 0.54 | 0.33 | 0.89 | 0.01609 | 0.06 | 0.05 | 0.08 | SNP |
| chr6_32440879_T_C | 32440879 | 1.54 | 1.08 | 2.21 | 0.01669 | 0.34 | 0.35 | 0.34 | SNP |
| chr6_31275583_C_T | 31275583 | 1.69 | 1.11 | 2.62 | 0.01689 | 0.11 | 0.13 | 0.08 | SNP |
| xHLA_2field_DPB1_02_01 | 33043767 | 0.59 | 0.37 | 0.90 | 0.01689 | 0.08 | 0.06 | 0.11 | HLA (xHLA) |
| chr6_33050186_T_C | 33050186 | 0.62 | 0.41 | 0.91 | 0.01696 | 0.10 | 0.08 | 0.12 | SNP |
| chr6_32945466_A_G | 32945466 | 0.47 | 0.25 | 0.87 | 0.01753 | 0.08 | 0.07 | 0.08 | SNP |
| chr6_33054074_C_T | 33054074 | 0.57 | 0.36 | 0.90 | 0.01773 | 0.07 | 0.06 | 0.10 | SNP |
| chr6_33046742_T_C | 33046742 | 0.56 | 0.34 | 0.90 | 0.01789 | 0.07 | 0.05 | 0.09 | SNP |
| chr6_33053468_G_T | 33053468 | 0.61 | 0.40 | 0.91 | 0.01792 | 0.09 | 0.07 | 0.12 | SNP |
| chr6_33054046_T_G | 33054046 | 0.63 | 0.42 | 0.92 | 0.01801 | 0.10 | 0.08 | 0.12 | SNP |
| chr6_33054057_G_A | 33054057 | 0.63 | 0.42 | 0.92 | 0.01801 | 0.10 | 0.08 | 0.12 | SNP |
| chr6_33054071_G_C | 33054071 | 0.63 | 0.42 | 0.92 | 0.01801 | 0.10 | 0.08 | 0.12 | SNP |
| chr6_31239827_T_C | 31239827 | 0.61 | 0.40 | 0.92 | 0.01812 | 0.38 | 0.35 | 0.41 | SNP |
| chr6_33143136_G_A | 33143136 | 0.53 | 0.31 | 0.89 | 0.01815 | 0.08 | 0.06 | 0.10 | SNP |
| chr6_33048602_C_A | 33048602 | 0.71 | 0.54 | 0.94 | 0.01843 | 0.28 | 0.24 | 0.32 | SNP |
| chr6_33052950_T_C | 33052950 | 0.61 | 0.41 | 0.92 | 0.01850 | 0.09 | 0.07 | 0.12 | SNP |
| chr6_33053968_T_A | 33053968 | 0.63 | 0.42 | 0.92 | 0.01857 | 0.10 | 0.08 | 0.12 | SNP |
| chr6_33053971_T_C | 33053971 | 0.63 | 0.42 | 0.92 | 0.01857 | 0.10 | 0.08 | 0.12 | SNP |
| chr6_33048606_G_C | 33048606 | 0.53 | 0.30 | 0.89 | 0.01877 | 0.07 | 0.05 | 0.09 | SNP |
| chr6_33054353_G_A | 33054353 | 0.63 | 0.42 | 0.92 | 0.01883 | 0.10 | 0.08 | 0.12 | SNP |
| chr6_33054355_A_C | 33054355 | 0.63 | 0.42 | 0.92 | 0.01883 | 0.10 | 0.08 | 0.12 | SNP |
| chr6_31462245_C_T | 31462245 | 0.66 | 0.47 | 0.93 | 0.01908 | 0.34 | 0.31 | 0.37 | SNP |
| chr6_31238522_C_T | 31238522 | 1.44 | 1.06 | 1.97 | 0.01949 | 0.22 | 0.25 | 0.19 | SNP |
| xHLA_2field_DQB1_03_02 | 32627244 | 0.63 | 0.42 | 0.93 | 0.01952 | 0.22 | 0.19 | 0.25 | HLA (xHLA) |
| chr6_33048102_C_T | 33048102 | 0.53 | 0.31 | 0.90 | 0.01987 | 0.06 | 0.04 | 0.08 | SNP |
| chr6_33048466_G_T | 33048466 | 0.56 | 0.34 | 0.91 | 0.01999 | 0.07 | 0.05 | 0.09 | SNP |
| chr6_33048467_G_T | 33048467 | 0.56 | 0.34 | 0.91 | 0.01999 | 0.07 | 0.05 | 0.09 | SNP |
| chr6_30521137_A_G | 30521137 | 0.73 | 0.56 | 0.95 | 0.02018 | 0.40 | 0.37 | 0.44 | SNP |
| chr6_31239766_C_T | 31239766 | 1.69 | 1.09 | 2.65 | 0.02025 | 0.12 | 0.14 | 0.09 | SNP |
| chr6_33053812_A_G | 33053812 | 0.59 | 0.37 | 0.92 | 0.02034 | 0.08 | 0.06 | 0.10 | SNP |
| chr6_33053942_T_C | 33053942 | 0.59 | 0.37 | 0.92 | 0.02034 | 0.08 | 0.06 | 0.10 | SNP |
| chr6_33054861_A_G | 33054861 | 0.59 | 0.37 | 0.92 | 0.02034 | 0.08 | 0.06 | 0.10 | SNP |
| chr6_33053399_T_C | 33053399 | 0.62 | 0.41 | 0.93 | 0.02041 | 0.10 | 0.08 | 0.12 | SNP |
| chr6_32135137_G_A | 32135137 | 2.15 | 1.14 | 4.21 | 0.02065 | 0.06 | 0.08 | 0.04 | SNP |
| chr6_33174783_A_G | 33174783 | 0.67 | 0.47 | 0.94 | 0.02071 | 0.14 | 0.12 | 0.17 | SNP |
| chr6_32973743_T_C | 32973743 | 1.48 | 1.07 | 2.08 | 0.02072 | 0.18 | 0.20 | 0.16 | SNP |
| chr6_32973878_T_C | 32973878 | 1.48 | 1.07 | 2.08 | 0.02072 | 0.18 | 0.20 | 0.16 | SNP |
| chr6_33052986_T_C | 33052986 | 0.63 | 0.43 | 0.93 | 0.02120 | 0.10 | 0.08 | 0.12 | SNP |
| chr6_33053723_T_C | 33053723 | 0.63 | 0.43 | 0.93 | 0.02120 | 0.10 | 0.08 | 0.12 | SNP |
| chr6_33053728_C_T | 33053728 | 0.63 | 0.43 | 0.93 | 0.02120 | 0.10 | 0.08 | 0.12 | SNP |
| chr6_32629259_T_C | 32629259 | 0.65 | 0.45 | 0.94 | 0.02127 | 0.23 | 0.21 | 0.25 | SNP |
| chr6_32185605_G_A | 32185605 | 1.87 | 1.11 | 3.23 | 0.02136 | 0.07 | 0.09 | 0.05 | SNP |
| chr6_33050084_C_T | 33050084 | 0.56 | 0.34 | 0.91 | 0.02141 | 0.06 | 0.05 | 0.08 | SNP |
| chr6_33050325_A_C | 33050325 | 0.56 | 0.34 | 0.91 | 0.02141 | 0.06 | 0.05 | 0.08 | SNP |
| chr6_33054145_A_G | 33054145 | 0.56 | 0.34 | 0.91 | 0.02141 | 0.06 | 0.05 | 0.08 | SNP |

| | | | | | | | | | |
|---|---|---|---|---|---|---|---|---|---|
| chr6_33050196_G_C | 33050196 | 0.63 | 0.42 | 0.93 | 0.02220 | 0.10 | 0.08 | 0.12 | SNP |
| chr6_33053396_A_G | 33053396 | 0.58 | 0.37 | 0.92 | 0.02227 | 0.07 | 0.06 | 0.09 | SNP |
| chr6_30796545_T_C | 30796545 | 0.51 | 0.28 | 0.90 | 0.02247 | 0.13 | 0.13 | 0.14 | SNP |
| chr6_30225908_C_T | 30225908 | 1.99 | 1.12 | 3.70 | 0.02257 | 0.05 | 0.07 | 0.03 | SNP |
| chr6_31765984_G_A | 31765984 | 0.39 | 0.17 | 0.87 | 0.02279 | 0.12 | 0.11 | 0.13 | SNP |
| xHLA_2field_B_08_01 | 31321652 | 0.44 | 0.21 | 0.88 | 0.02289 | 0.13 | 0.13 | 0.14 | HLA (xHLA) |
| chr6_33050499_A_G | 33050499 | 0.63 | 0.42 | 0.94 | 0.02327 | 0.10 | 0.08 | 0.12 | SNP |
| chr6_31326082_T_C | 31326082 | 1.41 | 1.05 | 1.91 | 0.02352 | 0.25 | 0.28 | 0.22 | SNP |
| chr6_32185796_C_T | 32185796 | 2.24 | 1.14 | 4.69 | 0.02356 | 0.04 | 0.05 | 0.02 | SNP |
| chr6_33054413_T_G | 33054413 | 0.64 | 0.43 | 0.94 | 0.02361 | 0.10 | 0.08 | 0.12 | SNP |
| chr6_33054414_T_A | 33054414 | 0.64 | 0.43 | 0.94 | 0.02361 | 0.10 | 0.08 | 0.12 | SNP |
| chr6_33041064_C_A | 33041064 | 0.56 | 0.33 | 0.92 | 0.02376 | 0.06 | 0.04 | 0.08 | SNP |
| chr6_33054342_T_A | 33054342 | 0.64 | 0.43 | 0.94 | 0.02378 | 0.10 | 0.08 | 0.12 | SNP |
| chr6_33054373_C_T | 33054373 | 0.64 | 0.43 | 0.94 | 0.02378 | 0.10 | 0.08 | 0.12 | SNP |
| chr6_33054381_T_C | 33054381 | 0.64 | 0.43 | 0.94 | 0.02378 | 0.10 | 0.08 | 0.12 | SNP |
| chr6_33054386_C_T | 33054386 | 0.64 | 0.43 | 0.94 | 0.02378 | 0.10 | 0.08 | 0.12 | SNP |
| chr6_31238708_G_A | 31238708 | 1.64 | 1.07 | 2.54 | 0.02394 | 0.10 | 0.13 | 0.08 | SNP |
| chr6_31323353_G_A | 31323353 | 1.38 | 1.05 | 1.84 | 0.02400 | 0.40 | 0.43 | 0.36 | SNP |
| chr6_33053902_A_G | 33053902 | 0.64 | 0.43 | 0.94 | 0.02502 | 0.10 | 0.08 | 0.12 | SNP |
| chr6_33053906_G_T | 33053906 | 0.64 | 0.43 | 0.94 | 0.02502 | 0.10 | 0.08 | 0.12 | SNP |
| chr6_31465047_G_T | 31465047 | 0.42 | 0.20 | 0.89 | 0.02520 | 0.13 | 0.13 | 0.13 | SNP |
| chr6_31323233_A_G | 31323233 | 1.39 | 1.04 | 1.86 | 0.02543 | 0.40 | 0.43 | 0.36 | SNP |
| chr6_32628189_A_G | 32628189 | 0.68 | 0.48 | 0.95 | 0.02593 | 0.27 | 0.25 | 0.30 | SNP |
| chr6_33048457_C_G | 33048457 | 0.62 | 0.41 | 0.94 | 0.02615 | 0.09 | 0.07 | 0.11 | SNP |
| chr6_33048461_T_A | 33048461 | 0.62 | 0.41 | 0.94 | 0.02637 | 0.09 | 0.07 | 0.11 | SNP |
| chr6_33050473_A_G | 33050473 | 0.64 | 0.43 | 0.95 | 0.02661 | 0.10 | 0.08 | 0.12 | SNP |
| chr6_31239449_C_G | 31239449 | 0.53 | 0.30 | 0.92 | 0.02679 | 0.17 | 0.16 | 0.19 | SNP |
| chr6_32166384_C_G | 32166384 | 1.78 | 1.08 | 3.02 | 0.02706 | 0.07 | 0.09 | 0.05 | SNP |
| chr6_33048459_T_G | 33048459 | 0.63 | 0.41 | 0.95 | 0.02727 | 0.09 | 0.07 | 0.11 | SNP |
| chr6_29971636_G_C | 29971636 | 1.96 | 1.10 | 3.66 | 0.02751 | 0.05 | 0.07 | 0.03 | SNP |
| chr6_33050203_C_G | 33050203 | 0.64 | 0.43 | 0.95 | 0.02758 | 0.10 | 0.08 | 0.12 | SNP |
| chr6_32628272_A_G | 32628272 | 1.55 | 1.06 | 2.30 | 0.02764 | 0.13 | 0.16 | 0.10 | SNP |
| chr6_33049979_A_G | 33049979 | 0.65 | 0.44 | 0.95 | 0.02765 | 0.10 | 0.08 | 0.12 | SNP |
| chr6_33049983_C_T | 33049983 | 0.65 | 0.44 | 0.95 | 0.02765 | 0.10 | 0.08 | 0.12 | SNP |
| chr6_31604355_C_A | 31604355 | 1.80 | 1.08 | 3.10 | 0.02766 | 0.07 | 0.08 | 0.05 | SNP |
| chr6_31325745_C_T | 31325745 | 0.73 | 0.55 | 0.97 | 0.02816 | 0.50 | 0.52 | 0.47 | SNP |
| chr6_32078373_A_C | 32078373 | 0.64 | 0.43 | 0.95 | 0.02866 | 0.11 | 0.09 | 0.14 | SNP |
| chr6_33050036_T_C | 33050036 | 0.58 | 0.35 | 0.94 | 0.02892 | 0.06 | 0.05 | 0.08 | SNP |
| chr6_32169145_G_A | 32169145 | 1.79 | 1.07 | 3.07 | 0.02906 | 0.07 | 0.08 | 0.05 | SNP |
| chr6_31325116_T_G | 31325116 | 0.63 | 0.41 | 0.95 | 0.02932 | 0.36 | 0.34 | 0.39 | SNP |
| chr6_31321267_A_C | 31321267 | 0.44 | 0.21 | 0.92 | 0.02945 | 0.13 | 0.13 | 0.14 | SNP |
| chr6_31473957_C_G | 31473957 | 0.43 | 0.20 | 0.91 | 0.02954 | 0.13 | 0.13 | 0.13 | SNP |
| chr6_33052958_C_T | 33052958 | 0.64 | 0.43 | 0.95 | 0.02959 | 0.10 | 0.08 | 0.12 | SNP |
| chr6_31592064_C_T | 31592064 | 2.24 | 1.11 | 4.80 | 0.02977 | 0.04 | 0.05 | 0.03 | SNP |
| chr6_33054400_T_C | 33054400 | 0.65 | 0.43 | 0.95 | 0.02988 | 0.10 | 0.08 | 0.12 | SNP |
| chr6_33054404_A_G | 33054404 | 0.65 | 0.43 | 0.95 | 0.02988 | 0.10 | 0.08 | 0.12 | SNP |
| chr6_33054410_A_C | 33054410 | 0.65 | 0.43 | 0.95 | 0.02988 | 0.10 | 0.08 | 0.12 | SNP |
| chr6_32611467_A_C | 32611467 | 0.65 | 0.44 | 0.96 | 0.03015 | 0.28 | 0.26 | 0.30 | SNP |
| xHLA_2field_DRB1_04_04 | 32546552 | 0.62 | 0.40 | 0.95 | 0.03029 | 0.09 | 0.07 | 0.12 | HLA (xHLA) |
| chr6_31474000_G_A | 31474000 | 0.72 | 0.53 | 0.97 | 0.03070 | 0.34 | 0.33 | 0.36 | SNP |
| chr6_31239543_C_T | 31239543 | 1.66 | 1.05 | 2.67 | 0.03132 | 0.11 | 0.13 | 0.09 | SNP |
| chr6_33053167_A_C | 33053167 | 0.65 | 0.44 | 0.96 | 0.03133 | 0.10 | 0.08 | 0.13 | SNP |
| chr6_32439964_T_G | 32439964 | 1.48 | 1.04 | 2.14 | 0.03155 | 0.34 | 0.35 | 0.33 | SNP |
| chr6_33053352_C_T | 33053352 | 0.60 | 0.38 | 0.95 | 0.03214 | 0.07 | 0.06 | 0.09 | SNP |
| chr6_32180431_A_T | 32180431 | 1.76 | 1.06 | 3.00 | 0.03219 | 0.07 | 0.09 | 0.05 | SNP |
| chr6_32628195_A_G | 32628195 | 0.54 | 0.30 | 0.94 | 0.03227 | 0.05 | 0.03 | 0.07 | SNP |
| chr6_31236854_T_C | 31236854 | 0.51 | 0.27 | 0.94 | 0.03231 | 0.15 | 0.14 | 0.15 | SNP |
| chr6_31478986_C_T | 31478986 | 0.72 | 0.54 | 0.97 | 0.03233 | 0.34 | 0.33 | 0.35 | SNP |
| chr6_33050144_C_T | 33050144 | 0.58 | 0.35 | 0.95 | 0.03255 | 0.06 | 0.05 | 0.08 | SNP |
| chr6_33039625_T_C | 33039625 | 0.68 | 0.48 | 0.97 | 0.03274 | 0.12 | 0.10 | 0.14 | SNP |
| chr6_33050463_C_G | 33050463 | 0.58 | 0.35 | 0.95 | 0.03280 | 0.06 | 0.05 | 0.08 | SNP |
| chr6_32190217_T_C | 32190217 | 1.43 | 1.03 | 1.99 | 0.03317 | 0.29 | 0.32 | 0.25 | SNP |
| chr6_31467170_G_A | 31467170 | 0.73 | 0.54 | 0.97 | 0.03369 | 0.34 | 0.33 | 0.35 | SNP |
| chr6_33050394_G_A | 33050394 | 0.58 | 0.35 | 0.95 | 0.03393 | 0.06 | 0.05 | 0.08 | SNP |
| chr6_31591928_A_G | 31591928 | 1.43 | 1.03 | 1.99 | 0.03434 | 0.37 | 0.40 | 0.34 | SNP |
| chr6_31325115_G_A | 31325115 | 0.64 | 0.42 | 0.96 | 0.03437 | 0.37 | 0.35 | 0.39 | SNP |
| chr6_32904981_A_G | 32904981 | 1.40 | 1.03 | 1.91 | 0.03441 | 0.21 | 0.23 | 0.19 | SNP |
| chr6_32629403_C_T | 32629403 | 0.68 | 0.47 | 0.97 | 0.03442 | 0.23 | 0.21 | 0.25 | SNP |
| chr6_32154711_A_G | 32154711 | 1.76 | 1.06 | 3.04 | 0.03447 | 0.07 | 0.08 | 0.05 | SNP |
| chr6_31325494_C_T | 31325494 | 0.65 | 0.43 | 0.97 | 0.03462 | 0.36 | 0.34 | 0.38 | SNP |
| chr6_31325504_C_T | 31325504 | 0.65 | 0.43 | 0.97 | 0.03462 | 0.36 | 0.34 | 0.38 | SNP |
| chr6_31325513_G_C | 31325513 | 0.65 | 0.43 | 0.97 | 0.03462 | 0.36 | 0.34 | 0.38 | SNP |
| chr6_31325521_C_G | 31325521 | 0.65 | 0.43 | 0.97 | 0.03462 | 0.36 | 0.34 | 0.38 | SNP |
| chr6_31325526_A_G | 31325526 | 0.65 | 0.43 | 0.97 | 0.03462 | 0.36 | 0.34 | 0.38 | SNP |
| chr6_31325529_C_T | 31325529 | 0.65 | 0.43 | 0.97 | 0.03462 | 0.36 | 0.34 | 0.38 | SNP |
| chr6_31325532_A_G | 31325532 | 0.65 | 0.43 | 0.97 | 0.03462 | 0.36 | 0.34 | 0.38 | SNP |
| chr6_32489964_G_A | 32489964 | 1.77 | 1.06 | 3.07 | 0.03473 | 0.08 | 0.10 | 0.05 | SNP |
| chr6_32151443_C_T | 32151443 | 1.69 | 1.05 | 2.78 | 0.03475 | 0.08 | 0.09 | 0.06 | SNP |
| chr6_29895945_A_T | 29895945 | 0.35 | 0.12 | 0.89 | 0.03529 | 0.02 | 0.01 | 0.03 | SNP |
| chr6_29913698_G_C | 29913698 | 0.64 | 0.41 | 0.97 | 0.03617 | 0.08 | 0.06 | 0.10 | SNP |
| chr6_32627450_G_A | 32627450 | 1.42 | 1.02 | 1.98 | 0.03634 | 0.36 | 0.40 | 0.32 | SNP |
| chr6_33382943_T_G | 33382943 | 1.58 | 1.03 | 2.44 | 0.03647 | 0.10 | 0.12 | 0.09 | SNP |
| chr6_32489960_A_G | 32489960 | 1.76 | 1.06 | 3.06 | 0.03649 | 0.08 | 0.10 | 0.05 | SNP |
| chr6_32604585_A_T | 32604585 | 0.66 | 0.44 | 0.97 | 0.03726 | 0.31 | 0.29 | 0.32 | SNP |
| chr6_33050521_G_T | 33050521 | 0.65 | 0.43 | 0.97 | 0.03751 | 0.10 | 0.08 | 0.12 | SNP |
| chr6_33596641_A_G | 33596641 | 1.36 | 1.02 | 1.83 | 0.03759 | 0.24 | 0.27 | 0.21 | SNP |
| chr6_31326072_G_A | 31326072 | 1.45 | 1.02 | 2.07 | 0.03773 | 0.15 | 0.17 | 0.13 | SNP |
| chr6_32190028_G_A | 32190028 | 0.57 | 0.33 | 0.97 | 0.03831 | 0.18 | 0.15 | 0.20 | SNP |
| chr6_33050525_T_C | 33050525 | 0.65 | 0.44 | 0.97 | 0.03844 | 0.10 | 0.08 | 0.12 | SNP |
| chr6_32096001_C_A | 32096001 | 0.41 | 0.17 | 0.95 | 0.03888 | 0.13 | 0.13 | 0.14 | SNP |
| chr6_32633811_C_T | 32633811 | 1.82 | 1.05 | 3.29 | 0.03910 | 0.05 | 0.06 | 0.03 | SNP |
| chr6_32802820_C_T | 32802820 | 1.31 | 1.01 | 1.71 | 0.03931 | 0.37 | 0.39 | 0.35 | SNP |
| chr6_31463790_T_C | 31463790 | 0.73 | 0.54 | 0.98 | 0.03939 | 0.34 | 0.33 | 0.35 | SNP |
| chr6_31237776_C_T | 31237776 | 1.36 | 1.02 | 1.83 | 0.03940 | 0.24 | 0.26 | 0.21 | SNP |
| chr6_33050526_C_T | 33050526 | 0.66 | 0.44 | 0.98 | 0.03985 | 0.10 | 0.08 | 0.12 | SNP |
| chr6_32080191_G_C | 32080191 | 0.41 | 0.17 | 0.95 | 0.03996 | 0.12 | 0.12 | 0.12 | SNP |
| chr6_32803999_A_T | 32803999 | 0.43 | 0.18 | 0.93 | 0.04046 | 0.02 | 0.01 | 0.03 | SNP |
| chr6_32627480_G_A | 32627480 | 0.71 | 0.51 | 0.98 | 0.04073 | 0.46 | 0.45 | 0.49 | SNP |
| chr6_32500957_A_T | 32500957 | 0.57 | 0.33 | 0.97 | 0.04076 | 0.15 | 0.15 | 0.16 | SNP |
| chr6_32945530_C_G | 32945530 | 1.62 | 1.03 | 2.60 | 0.04077 | 0.09 | 0.10 | 0.08 | SNP |
| chr6_31464365_G_C | 31464365 | 0.73 | 0.54 | 0.99 | 0.04077 | 0.34 | 0.33 | 0.36 | SNP |
| chr6_32551638_C_T | 32551638 | 1.80 | 1.04 | 3.20 | 0.04078 | 0.06 | 0.07 | 0.04 | SNP |
| chr6_33053252_G_A | 33053252 | 0.65 | 0.42 | 0.98 | 0.04085 | 0.10 | 0.08 | 0.12 | SNP |
| chr6_31467365_C_T | 31467365 | 0.73 | 0.54 | 0.99 | 0.04102 | 0.34 | 0.33 | 0.35 | SNP |
| chr6_33239869_C_T | 33239869 | 0.67 | 0.46 | 0.98 | 0.04107 | 0.11 | 0.09 | 0.13 | SNP |
| chr6_31326055_G_A | 31326055 | 1.44 | 1.02 | 2.05 | 0.04182 | 0.15 | 0.17 | 0.13 | SNP |
| chr6_30552937_C_A | 30552937 | 0.76 | 0.58 | 0.99 | 0.04195 | 0.40 | 0.37 | 0.43 | SNP |
| chr6_32325562_G_A | 32325562 | 0.43 | 0.19 | 0.96 | 0.04202 | 0.12 | 0.12 | 0.12 | SNP |
| chr6_32180146_G_A | 32180146 | 0.57 | 0.33 | 0.98 | 0.04213 | 0.17 | 0.15 | 0.20 | SNP |
| chr6_32374595_A_G | 32374595 | 0.55 | 0.31 | 0.97 | 0.04251 | 0.14 | 0.14 | 0.15 | SNP |
| chr6_32375796_A_T | 32375796 | 0.55 | 0.31 | 0.97 | 0.04251 | 0.14 | 0.14 | 0.15 | SNP |
| chr6_30851909_C_A | 30851909 | 0.50 | 0.25 | 0.97 | 0.04273 | 0.15 | 0.14 | 0.16 | SNP |
| chr6_33136638_A_G | 33136638 | 1.28 | 1.01 | 1.63 | 0.04276 | 0.43 | 0.46 | 0.40 | SNP |
| chr6_31325944_A_G | 31325944 | 1.43 | 1.01 | 2.04 | 0.04303 | 0.15 | 0.17 | 0.13 | SNP |
| chr6_31325950_T_G | 31325950 | 1.43 | 1.01 | 2.04 | 0.04303 | 0.15 | 0.17 | 0.13 | SNP |
| chr6_31321413_T_C | 31321413 | 1.54 | 1.02 | 2.35 | 0.04318 | 0.10 | 0.12 | 0.09 | SNP |
| chr6_31473561_A_G | 31473561 | 0.74 | 0.54 | 0.99 | 0.04342 | 0.34 | 0.33 | 0.35 | SNP |
| chr6_31325792_G_A | 31325792 | 0.68 | 0.47 | 0.99 | 0.04359 | 0.38 | 0.36 | 0.41 | SNP |
| chr6_31466885_G_A | 31466885 | 0.74 | 0.54 | 0.99 | 0.04363 | 0.33 | 0.32 | 0.35 | SNP |
| chr6_29909999_C_T | 29909999 | 0.65 | 0.42 | 0.98 | 0.04392 | 0.08 | 0.07 | 0.10 | SNP |
| chr6_33142253_G_C | 33142253 | 1.27 | 1.01 | 1.61 | 0.04394 | 0.45 | 0.48 | 0.42 | SNP |
| chr6_32945097_C_T | 32945097 | 0.43 | 0.18 | 0.95 | 0.04400 | 0.03 | 0.01 | 0.03 | SNP |
| chr6_31465490_G_C | 31465490 | 0.74 | 0.55 | 0.99 | 0.04464 | 0.34 | 0.33 | 0.36 | SNP |
| chr6_29912297_A_G | 29912297 | 0.65 | 0.42 | 0.99 | 0.04465 | 0.08 | 0.07 | 0.10 | SNP |
| chr6_31465558_G_C | 31465558 | 0.73 | 0.54 | 0.99 | 0.04470 | 0.34 | 0.33 | 0.36 | SNP |
| chr6_31322855_C_T | 31322855 | 0.62 | 0.38 | 0.99 | 0.04548 | 0.07 | 0.06 | 0.09 | SNP |
| chr6_29913824_T_C | 29913824 | 0.65 | 0.42 | 0.99 | 0.04609 | 0.08 | 0.06 | 0.10 | SNP |
| chr6_29913830_C_T | 29913830 | 0.65 | 0.42 | 0.99 | 0.04609 | 0.08 | 0.06 | 0.10 | SNP |
| chr6_29912281_G_A | 29912281 | 0.65 | 0.42 | 0.99 | 0.04621 | 0.08 | 0.07 | 0.10 | SNP |
| chr6_32635954_G_A | 32635954 | 0.69 | 0.48 | 0.99 | 0.04626 | 0.22 | 0.21 | 0.24 | SNP |
| chr6_32551700_T_G | 32551700 | 0.67 | 0.45 | 0.99 | 0.04636 | 0.25 | 0.22 | 0.27 | SNP |
| chr6_33632618_C_T | 33632618 | 2.28 | 1.04 | 5.39 | 0.04651 | 0.03 | 0.04 | 0.02 | SNP |
| chr6_29912562_T_G | 29912562 | 0.65 | 0.42 | 0.99 | 0.04652 | 0.08 | 0.07 | 0.10 | SNP |
| chr6_29912564_A_C | 29912564 | 0.65 | 0.42 | 0.99 | 0.04652 | 0.08 | 0.07 | 0.10 | SNP |
| chr6_29913201_T_G | 29913201 | 0.65 | 0.42 | 0.99 | 0.04652 | 0.08 | 0.07 | 0.10 | SNP |
| chr6_29913449_C_G | 29913449 | 0.65 | 0.42 | 0.99 | 0.04652 | 0.08 | 0.07 | 0.10 | SNP |
| chr6_32821447_C_T | 32821447 | 1.51 | 1.01 | 2.29 | 0.04660 | 0.17 | 0.19 | 0.16 | SNP |
| chr6_32180241_C_T | 32180241 | 1.57 | 1.01 | 2.48 | 0.04665 | 0.09 | 0.10 | 0.07 | SNP |
| chr6_33053248_A_G | 33053248 | 0.65 | 0.43 | 0.99 | 0.04708 | 0.10 | 0.08 | 0.12 | SNP |
| chr6_32948426_C_T | 32948426 | 1.54 | 1.01 | 2.37 | 0.04710 | 0.10 | 0.12 | 0.08 | SNP |
| chr6_31464798_C_T | 31464798 | 0.74 | 0.55 | 1.00 | 0.04722 | 0.34 | 0.33 | 0.35 | SNP |
| chr6_29913886_T_G | 29913886 | 0.65 | 0.42 | 0.99 | 0.04727 | 0.08 | 0.07 | 0.10 | SNP |
| chr6_32021355_G_A | 32021355 | 0.35 | 0.12 | 0.95 | 0.04766 | 0.02 | 0.01 | 0.03 | SNP |
| xHLA_2field_B_44_02 | 31321652 | 1.48 | 1.01 | 2.21 | 0.04802 | 0.11 | 0.13 | 0.09 | HLA (xHLA) |
| chr6_31462135_G_A | 31462135 | 0.42 | 0.18 | 0.98 | 0.04830 | 0.14 | 0.13 | 0.15 | SNP |
| chr6_31721033_G_A | 31721033 | 0.46 | 0.21 | 0.99 | 0.04840 | 0.12 | 0.12 | 0.13 | SNP |
| chr6_30530245_C_T | 30530245 | 0.77 | 0.59 | 1.00 | 0.04863 | 0.38 | 0.35 | 0.42 | SNP |
| chr6_32172065_G_T | 32172065 | 0.57 | 0.33 | 0.99 | 0.04866 | 0.17 | 0.15 | 0.20 | SNP |
| chr6_30879987_C_T | 30879987 | 0.53 | 0.28 | 0.99 | 0.04878 | 0.13 | 0.13 | 0.13 | SNP |
| chr6_31915614_G_A | 31915614 | 2.00 | 1.02 | 4.08 | 0.04879 | 0.04 | 0.05 | 0.03 | SNP |
| chr6_31240721_C_T | 31240721 | 1.32 | 1.00 | 1.76 | 0.04882 | 0.38 | 0.41 | 0.35 | SNP |
| chr6_31467111_G_T | 31467111 | 0.74 | 0.55 | 1.00 | 0.04899 | 0.34 | 0.33 | 0.35 | SNP |
| chr6_31479126_C_T | 31479126 | 1.33 | 1.00 | 1.77 | 0.04947 | 0.35 | 0.38 | 0.32 | SNP |
| chr6_33636232_C_T | 33636232 | 1.46 | 1.00 | 2.14 | 0.04958 | 0.14 | 0.16 | 0.12 | SNP |
| chr6_32029205_G_A | 32029205 | 0.44 | 0.19 | 0.99 | 0.04983 | 0.12 | 0.12 | 0.13 | SNP |
| xHLA_2field_DPB1_03_01 | 33043767 | 0.54 | 0.29 | 0.99 | 0.04991 | 0.04 | 0.03 | 0.06 | HLA (xHLA) |

ANCA = anitneutrophil cytoplasmic antibody; AAV = ANCA-associated vasculitis; SNP = single nucleotide polymorphism;
OR = Odds ratio; CI = confidence interval; MAF = minor allele frequency. SNPs and HLA alleles with P < 0.05 included in the table.

**Supplemtary Table 7**. Association analysis of ear-nose- and throat involvement in all AAV patients versus controls, adjusting for sex and genetic structure (principal components 1-4).

| Variant | position | OR | CI_lower | CI_upper | p | MAF | MAF_cases | MAF_controls | Type (SNP, HLA (xHLA)) |
|---|---|---|---|---|---|---|---|---|---|
| chr6_33053455_C_T | 33053455 | 0.33 | 0.22 | 0.50 | 2.45E-07 | 0.10 | 0.06 | 0.16 | SNP |
| chr6_33053399_T_C | 33053399 | 0.34 | 0.22 | 0.51 | 3.83E-07 | 0.10 | 0.06 | 0.17 | SNP |
| chr6_33053477_G_C | 33053477 | 0.34 | 0.22 | 0.52 | 5.77E-07 | 0.09 | 0.06 | 0.16 | SNP |
| chr6_33052986_T_C | 33052986 | 0.36 | 0.24 | 0.53 | 5.91E-07 | 0.10 | 0.06 | 0.17 | SNP |
| chr6_33053723_T_C | 33053723 | 0.36 | 0.24 | 0.53 | 5.91E-07 | 0.10 | 0.06 | 0.17 | SNP |
| chr6_33053728_C_T | 33053728 | 0.36 | 0.24 | 0.53 | 5.91E-07 | 0.10 | 0.06 | 0.17 | SNP |
| chr6_33053468_G_T | 33053468 | 0.35 | 0.22 | 0.53 | 9.98E-07 | 0.09 | 0.06 | 0.16 | SNP |
| chr6_33050499_A_G | 33050499 | 0.36 | 0.24 | 0.54 | 1.01E-06 | 0.10 | 0.06 | 0.17 | SNP |
| xHLA_2field_DRB1_04_04 | 32546552 | 0.33 | 0.21 | 0.51 | 1.04E-06 | 0.09 | 0.06 | 0.16 | HLA (xHLA) |
| chr6_33050148_T_C | 33050148 | 0.35 | 0.23 | 0.53 | 1.24E-06 | 0.09 | 0.06 | 0.16 | SNP |
| chr6_33071754_C_A | 33071754 | 0.34 | 0.21 | 0.52 | 1.36E-06 | 0.09 | 0.06 | 0.15 | SNP |
| chr6_33050118_C_T | 33050118 | 0.35 | 0.23 | 0.54 | 1.41E-06 | 0.09 | 0.06 | 0.16 | SNP |
| chr6_33055009_C_A | 33055009 | 0.38 | 0.25 | 0.56 | 1.50E-06 | 0.10 | 0.07 | 0.17 | SNP |
| chr6_33053682_G_A | 33053682 | 0.38 | 0.25 | 0.56 | 1.63E-06 | 0.10 | 0.07 | 0.17 | SNP |
| chr6_33053772_G_A | 33053772 | 0.38 | 0.25 | 0.56 | 1.63E-06 | 0.10 | 0.07 | 0.17 | SNP |
| chr6_33053780_T_A | 33053780 | 0.38 | 0.25 | 0.56 | 1.63E-06 | 0.10 | 0.07 | 0.17 | SNP |
| chr6_33053789_A_G | 33053789 | 0.38 | 0.25 | 0.56 | 1.63E-06 | 0.10 | 0.07 | 0.17 | SNP |
| chr6_33053868_A_G | 33053868 | 0.38 | 0.25 | 0.56 | 1.63E-06 | 0.10 | 0.07 | 0.17 | SNP |
| chr6_33053871_A_G | 33053871 | 0.38 | 0.25 | 0.56 | 1.63E-06 | 0.10 | 0.07 | 0.17 | SNP |
| chr6_33053877_G_A | 33053877 | 0.38 | 0.25 | 0.56 | 1.63E-06 | 0.10 | 0.07 | 0.17 | SNP |
| chr6_33053887_A_G | 33053887 | 0.38 | 0.25 | 0.56 | 1.63E-06 | 0.10 | 0.07 | 0.17 | SNP |
| chr6_33053890_G_A | 33053890 | 0.38 | 0.25 | 0.56 | 1.63E-06 | 0.10 | 0.07 | 0.17 | SNP |
| chr6_33053982_G_C | 33053982 | 0.38 | 0.25 | 0.56 | 1.63E-06 | 0.10 | 0.07 | 0.17 | SNP |
| chr6_33054091_C_T | 33054091 | 0.38 | 0.25 | 0.56 | 1.63E-06 | 0.10 | 0.07 | 0.17 | SNP |
| chr6_33054141_T_C | 33054141 | 0.38 | 0.25 | 0.56 | 1.63E-06 | 0.10 | 0.07 | 0.17 | SNP |
| chr6_33054142_G_C | 33054142 | 0.38 | 0.25 | 0.56 | 1.63E-06 | 0.10 | 0.07 | 0.17 | SNP |
| chr6_33054148_C_T | 33054148 | 0.38 | 0.25 | 0.56 | 1.63E-06 | 0.10 | 0.07 | 0.17 | SNP |
| chr6_33054149_T_C | 33054149 | 0.38 | 0.25 | 0.56 | 1.63E-06 | 0.10 | 0.07 | 0.17 | SNP |
| chr6_33054152_A_G | 33054152 | 0.38 | 0.25 | 0.56 | 1.63E-06 | 0.10 | 0.07 | 0.17 | SNP |
| chr6_33054155_C_T | 33054155 | 0.38 | 0.25 | 0.56 | 1.63E-06 | 0.10 | 0.07 | 0.17 | SNP |
| chr6_33054156_A_G | 33054156 | 0.38 | 0.25 | 0.56 | 1.63E-06 | 0.10 | 0.07 | 0.17 | SNP |
| chr6_33054231_C_T | 33054231 | 0.38 | 0.25 | 0.56 | 1.63E-06 | 0.10 | 0.07 | 0.17 | SNP |
| chr6_33054235_T_G | 33054235 | 0.38 | 0.25 | 0.56 | 1.63E-06 | 0.10 | 0.07 | 0.17 | SNP |
| chr6_33054268_C_A | 33054268 | 0.38 | 0.25 | 0.56 | 1.63E-06 | 0.10 | 0.07 | 0.17 | SNP |
| chr6_33054280_T_C | 33054280 | 0.38 | 0.25 | 0.56 | 1.63E-06 | 0.10 | 0.07 | 0.17 | SNP |
| chr6_33054281_G_A | 33054281 | 0.38 | 0.25 | 0.56 | 1.63E-06 | 0.10 | 0.07 | 0.17 | SNP |
| chr6_33054302_T_C | 33054302 | 0.38 | 0.25 | 0.56 | 1.63E-06 | 0.10 | 0.07 | 0.17 | SNP |
| chr6_33054325_G_C | 33054325 | 0.38 | 0.25 | 0.56 | 1.63E-06 | 0.10 | 0.07 | 0.17 | SNP |
| chr6_33054331_T_C | 33054331 | 0.38 | 0.25 | 0.56 | 1.63E-06 | 0.10 | 0.07 | 0.17 | SNP |
| chr6_33054457_A_G | 33054457 | 0.38 | 0.25 | 0.56 | 1.63E-06 | 0.10 | 0.07 | 0.17 | SNP |
| chr6_33054550_G_A | 33054550 | 0.38 | 0.25 | 0.56 | 1.63E-06 | 0.10 | 0.07 | 0.17 | SNP |
| chr6_33054579_C_T | 33054579 | 0.38 | 0.25 | 0.56 | 1.63E-06 | 0.10 | 0.07 | 0.17 | SNP |
| chr6_33054586_A_G | 33054586 | 0.38 | 0.25 | 0.56 | 1.63E-06 | 0.10 | 0.07 | 0.17 | SNP |
| chr6_33054595_C_T | 33054595 | 0.38 | 0.25 | 0.56 | 1.63E-06 | 0.10 | 0.07 | 0.17 | SNP |
| chr6_33054619_G_A | 33054619 | 0.38 | 0.25 | 0.56 | 1.63E-06 | 0.10 | 0.07 | 0.17 | SNP |
| chr6_33054656_A_C | 33054656 | 0.38 | 0.25 | 0.56 | 1.63E-06 | 0.10 | 0.07 | 0.17 | SNP |
| chr6_33054675_C_G | 33054675 | 0.38 | 0.25 | 0.56 | 1.63E-06 | 0.10 | 0.07 | 0.17 | SNP |
| chr6_33054683_A_G | 33054683 | 0.38 | 0.25 | 0.56 | 1.63E-06 | 0.10 | 0.07 | 0.17 | SNP |
| chr6_33054687_G_A | 33054687 | 0.38 | 0.25 | 0.56 | 1.63E-06 | 0.10 | 0.07 | 0.17 | SNP |
| chr6_33054711_G_T | 33054711 | 0.38 | 0.25 | 0.56 | 1.63E-06 | 0.10 | 0.07 | 0.17 | SNP |
| chr6_33054721_C_T | 33054721 | 0.38 | 0.25 | 0.56 | 1.63E-06 | 0.10 | 0.07 | 0.17 | SNP |
| chr6_33054807_A_G | 33054807 | 0.38 | 0.25 | 0.56 | 1.63E-06 | 0.10 | 0.07 | 0.17 | SNP |
| chr6_33054890_C_T | 33054890 | 0.38 | 0.25 | 0.56 | 1.63E-06 | 0.10 | 0.07 | 0.17 | SNP |
| chr6_33055047_A_G | 33055047 | 0.38 | 0.25 | 0.56 | 1.76E-06 | 0.10 | 0.07 | 0.17 | SNP |
| chr6_33050473_A_G | 33050473 | 0.37 | 0.25 | 0.55 | 1.80E-06 | 0.10 | 0.06 | 0.16 | SNP |
| chr6_33052958_C_T | 33052958 | 0.37 | 0.24 | 0.55 | 1.81E-06 | 0.10 | 0.06 | 0.16 | SNP |
| chr6_33050279_A_G | 33050279 | 0.38 | 0.26 | 0.56 | 1.87E-06 | 0.10 | 0.07 | 0.17 | SNP |
| chr6_33050078_A_G | 33050078 | 0.37 | 0.25 | 0.56 | 1.96E-06 | 0.10 | 0.07 | 0.16 | SNP |
| chr6_33050079_C_G | 33050079 | 0.37 | 0.25 | 0.56 | 1.96E-06 | 0.10 | 0.07 | 0.16 | SNP |
| chr6_33050125_G_A | 33050125 | 0.36 | 0.23 | 0.55 | 2.09E-06 | 0.09 | 0.06 | 0.15 | SNP |
| chr6_33055123_A_G | 33055123 | 0.37 | 0.25 | 0.56 | 2.21E-06 | 0.10 | 0.07 | 0.17 | SNP |
| chr6_33049211_C_T | 33049211 | 0.38 | 0.25 | 0.57 | 2.22E-06 | 0.10 | 0.07 | 0.17 | SNP |
| chr6_33054046_T_G | 33054046 | 0.38 | 0.25 | 0.57 | 2.38E-06 | 0.10 | 0.07 | 0.16 | SNP |
| chr6_33054057_G_A | 33054057 | 0.38 | 0.25 | 0.57 | 2.38E-06 | 0.10 | 0.07 | 0.16 | SNP |
| chr6_33054071_G_C | 33054071 | 0.38 | 0.25 | 0.57 | 2.38E-06 | 0.10 | 0.07 | 0.16 | SNP |
| chr6_33053968_T_A | 33053968 | 0.38 | 0.25 | 0.57 | 2.45E-06 | 0.10 | 0.07 | 0.16 | SNP |
| chr6_33053971_T_C | 33053971 | 0.38 | 0.25 | 0.57 | 2.45E-06 | 0.10 | 0.07 | 0.16 | SNP |
| chr6_33047432_A_C | 33047432 | 0.39 | 0.26 | 0.57 | 2.49E-06 | 0.10 | 0.07 | 0.17 | SNP |
| chr6_32604585_A_T | 32604585 | 0.35 | 0.23 | 0.54 | 2.51E-06 | 0.31 | 0.27 | 0.38 | SNP |
| chr6_33054353_G_A | 33054353 | 0.38 | 0.25 | 0.57 | 2.60E-06 | 0.10 | 0.07 | 0.16 | SNP |
| chr6_33054355_A_C | 33054355 | 0.38 | 0.25 | 0.57 | 2.60E-06 | 0.10 | 0.07 | 0.16 | SNP |
| chr6_33053252_G_A | 33053252 | 0.36 | 0.23 | 0.55 | 2.79E-06 | 0.10 | 0.06 | 0.16 | SNP |
| chr6_33050179_A_G | 33050179 | 0.38 | 0.25 | 0.57 | 3.22E-06 | 0.10 | 0.07 | 0.16 | SNP |
| chr6_33050168_C_A | 33050168 | 0.38 | 0.25 | 0.57 | 3.35E-06 | 0.10 | 0.07 | 0.16 | SNP |
| chr6_33050185_C_A | 33050185 | 0.38 | 0.25 | 0.57 | 3.44E-06 | 0.10 | 0.07 | 0.16 | SNP |
| chr6_33050089_G_A | 33050089 | 0.38 | 0.25 | 0.57 | 3.49E-06 | 0.10 | 0.07 | 0.16 | SNP |
| chr6_33054659_A_G | 33054659 | 0.39 | 0.26 | 0.58 | 3.61E-06 | 0.10 | 0.07 | 0.16 | SNP |
| chr6_33048599_C_A | 33048599 | 0.49 | 0.36 | 0.66 | 3.82E-06 | 0.29 | 0.24 | 0.38 | SNP |
| chr6_32607126_A_G | 32607126 | 0.38 | 0.25 | 0.57 | 3.85E-06 | 0.31 | 0.27 | 0.38 | SNP |
| chr6_33039625_T_C | 33039625 | 0.43 | 0.29 | 0.61 | 3.90E-06 | 0.12 | 0.08 | 0.19 | SNP |
| chr6_33054413_T_G | 33054413 | 0.38 | 0.25 | 0.57 | 3.90E-06 | 0.10 | 0.07 | 0.16 | SNP |
| chr6_33054414_T_A | 33054414 | 0.38 | 0.25 | 0.57 | 3.90E-06 | 0.10 | 0.07 | 0.16 | SNP |
| chr6_33050223_G_A | 33050223 | 0.39 | 0.26 | 0.58 | 3.91E-06 | 0.10 | 0.07 | 0.16 | SNP |
| chr6_33053248_A_G | 33053248 | 0.36 | 0.23 | 0.55 | 4.00E-06 | 0.10 | 0.06 | 0.16 | SNP |
| chr6_33053902_A_G | 33053902 | 0.39 | 0.26 | 0.58 | 4.15E-06 | 0.10 | 0.07 | 0.16 | SNP |
| chr6_33053906_G_T | 33053906 | 0.39 | 0.26 | 0.58 | 4.15E-06 | 0.10 | 0.07 | 0.16 | SNP |
| chr6_33054342_T_A | 33054342 | 0.39 | 0.26 | 0.58 | 4.21E-06 | 0.10 | 0.07 | 0.16 | SNP |
| chr6_33054373_C_T | 33054373 | 0.39 | 0.26 | 0.58 | 4.21E-06 | 0.10 | 0.07 | 0.16 | SNP |
| chr6_33054381_T_C | 33054381 | 0.39 | 0.26 | 0.58 | 4.21E-06 | 0.10 | 0.07 | 0.16 | SNP |
| chr6_33054386_C_T | 33054386 | 0.39 | 0.26 | 0.58 | 4.21E-06 | 0.10 | 0.07 | 0.16 | SNP |
| chr6_33050232_T_G | 33050232 | 0.38 | 0.25 | 0.57 | 4.22E-06 | 0.10 | 0.07 | 0.16 | SNP |
| chr6_33055079_A_G | 33055079 | 0.39 | 0.26 | 0.58 | 4.28E-06 | 0.10 | 0.07 | 0.16 | SNP |
| chr6_33050107_G_A | 33050107 | 0.38 | 0.25 | 0.57 | 4.28E-06 | 0.10 | 0.07 | 0.16 | SNP |
| chr6_33050525_T_C | 33050525 | 0.38 | 0.25 | 0.57 | 4.35E-06 | 0.10 | 0.06 | 0.16 | SNP |
| chr6_33071708_G_A | 33071708 | 0.35 | 0.22 | 0.55 | 4.56E-06 | 0.09 | 0.06 | 0.15 | SNP |
| chr6_33050526_C_T | 33050526 | 0.38 | 0.25 | 0.57 | 4.59E-06 | 0.10 | 0.06 | 0.16 | SNP |
| chr6_33052950_T_C | 33052950 | 0.38 | 0.25 | 0.57 | 4.65E-06 | 0.09 | 0.06 | 0.15 | SNP |
| chr6_33050521_G_T | 33050521 | 0.38 | 0.25 | 0.57 | 4.89E-06 | 0.10 | 0.07 | 0.16 | SNP |
| chr6_32605178_T_C | 32605178 | 0.39 | 0.26 | 0.58 | 4.92E-06 | 0.30 | 0.27 | 0.37 | SNP |
| chr6_33050186_T_C | 33050186 | 0.39 | 0.25 | 0.58 | 5.11E-06 | 0.10 | 0.07 | 0.16 | SNP |
| xHLA_2field_DPB1_04_01 | 33043767 | 1.76 | 1.38 | 2.24 | 5.45E-06 | 0.34 | 0.29 | 0.44 | HLA (xHLA) |
| chr6_32605555_T_A | 32605555 | 0.39 | 0.26 | 0.58 | 5.51E-06 | 0.31 | 0.27 | 0.38 | SNP |
| chr6_33071777_A_G | 33071777 | 0.36 | 0.23 | 0.55 | 5.67E-06 | 0.09 | 0.06 | 0.15 | SNP |
| chr6_33053270_T_A | 33053270 | 0.36 | 0.23 | 0.56 | 6.02E-06 | 0.09 | 0.06 | 0.15 | SNP |
| chr6_32609479_C_A | 32609479 | 0.39 | 0.26 | 0.59 | 6.09E-06 | 0.30 | 0.27 | 0.37 | SNP |
| chr6_33053271_T_C | 33053271 | 0.36 | 0.23 | 0.56 | 6.15E-06 | 0.09 | 0.06 | 0.16 | SNP |
| chr6_32608478_T_C | 32608478 | 0.39 | 0.26 | 0.58 | 6.30E-06 | 0.30 | 0.27 | 0.37 | SNP |
| chr6_33048602_C_A | 33048602 | 0.50 | 0.37 | 0.67 | 6.45E-06 | 0.28 | 0.23 | 0.37 | SNP |
| chr6_33054400_T_C | 33054400 | 0.39 | 0.26 | 0.59 | 6.45E-06 | 0.10 | 0.07 | 0.16 | SNP |
| chr6_33054404_A_G | 33054404 | 0.39 | 0.26 | 0.59 | 6.45E-06 | 0.10 | 0.07 | 0.16 | SNP |
| chr6_33054410_A_C | 33054410 | 0.39 | 0.26 | 0.59 | 6.45E-06 | 0.10 | 0.07 | 0.16 | SNP |
| chr6_33050196_G_C | 33050196 | 0.39 | 0.26 | 0.59 | 6.61E-06 | 0.10 | 0.07 | 0.16 | SNP |
| chr6_32605609_G_A | 32605609 | 0.39 | 0.26 | 0.59 | 6.64E-06 | 0.31 | 0.27 | 0.38 | SNP |
| chr6_32559191_A_G | 32559191 | 0.39 | 0.26 | 0.58 | 6.67E-06 | 0.29 | 0.25 | 0.36 | SNP |
| chr6_32606987_T_G | 32606987 | 0.40 | 0.26 | 0.59 | 6.69E-06 | 0.31 | 0.27 | 0.38 | SNP |
| chr6_33053167_A_C | 33053167 | 0.40 | 0.27 | 0.59 | 6.96E-06 | 0.10 | 0.07 | 0.17 | SNP |
| chr6_33050024_C_A | 33050024 | 0.40 | 0.26 | 0.59 | 7.00E-06 | 0.10 | 0.07 | 0.16 | SNP |
| chr6_33050045_T_C | 33050045 | 0.40 | 0.26 | 0.59 | 7.00E-06 | 0.10 | 0.07 | 0.16 | SNP |
| chr6_33053307_C_T | 33053307 | 0.38 | 0.25 | 0.58 | 7.38E-06 | 0.10 | 0.06 | 0.16 | SNP |
| chr6_33045208_T_C | 33045208 | 0.51 | 0.38 | 0.69 | 8.23E-06 | 0.24 | 0.20 | 0.31 | SNP |
| chr6_33050203_C_G | 33050203 | 0.40 | 0.26 | 0.60 | 8.45E-06 | 0.10 | 0.07 | 0.16 | SNP |
| chr6_32605696_G_T | 32605696 | 0.40 | 0.26 | 0.59 | 8.65E-06 | 0.31 | 0.27 | 0.37 | SNP |
| chr6_33047031_T_C | 33047031 | 0.40 | 0.27 | 0.60 | 9.15E-06 | 0.10 | 0.07 | 0.16 | SNP |

| | | | | | | | | | |
|---|---|---|---|---|---|---|---|---|---|
| chr6_33045255_T_C | 33045255 | 0.52 | 0.39 | 0.69 | 9.97E-06 | 0.24 | 0.20 | 0.31 | SNP |
| chr6_33071600_C_A | 33071600 | 0.37 | 0.24 | 0.57 | 1.02E-05 | 0.09 | 0.06 | 0.15 | SNP |
| chr6_32610401_G_A | 32610401 | 0.39 | 0.26 | 0.59 | 1.04E-05 | 0.30 | 0.27 | 0.36 | SNP |
| chr6_32603798_A_T | 32603798 | 0.40 | 0.27 | 0.60 | 1.10E-05 | 0.30 | 0.26 | 0.37 | SNP |
| chr6_33049979_A_G | 33049979 | 0.41 | 0.28 | 0.61 | 1.12E-05 | 0.10 | 0.07 | 0.16 | SNP |
| chr6_33049983_C_T | 33049983 | 0.41 | 0.28 | 0.61 | 1.12E-05 | 0.10 | 0.07 | 0.16 | SNP |
| chr6_33053396_A_G | 33053396 | 0.36 | 0.22 | 0.57 | 1.60E-05 | 0.07 | 0.05 | 0.13 | SNP |
| chr6_32559213_A_T | 32559213 | 0.40 | 0.26 | 0.60 | 1.61E-05 | 0.29 | 0.25 | 0.35 | SNP |
| chr6_33048542_C_T | 33048542 | 0.55 | 0.42 | 0.72 | 1.71E-05 | 0.29 | 0.24 | 0.37 | SNP |
| chr6_33048459_T_G | 33048459 | 0.39 | 0.25 | 0.60 | 1.77E-05 | 0.09 | 0.06 | 0.14 | SNP |
| chr6_33048461_T_A | 33048461 | 0.39 | 0.26 | 0.60 | 2.01E-05 | 0.09 | 0.06 | 0.14 | SNP |
| chr6_32606616_A_T | 32606616 | 0.42 | 0.28 | 0.62 | 2.06E-05 | 0.33 | 0.30 | 0.39 | SNP |
| chr6_33048457_C_G | 33048457 | 0.40 | 0.26 | 0.61 | 2.19E-05 | 0.09 | 0.06 | 0.14 | SNP |
| chr6_33050144_C_T | 33050144 | 0.34 | 0.20 | 0.56 | 2.70E-05 | 0.06 | 0.04 | 0.11 | SNP |
| chr6_33050394_G_A | 33050394 | 0.34 | 0.20 | 0.56 | 2.86E-05 | 0.06 | 0.04 | 0.11 | SNP |
| chr6_33050463_C_G | 33050463 | 0.34 | 0.20 | 0.56 | 2.88E-05 | 0.06 | 0.04 | 0.11 | SNP |
| chr6_32608286_T_C | 32608286 | 0.40 | 0.26 | 0.61 | 3.11E-05 | 0.31 | 0.27 | 0.38 | SNP |
| chr6_33053812_A_G | 33053812 | 0.38 | 0.24 | 0.60 | 3.25E-05 | 0.08 | 0.05 | 0.13 | SNP |
| chr6_33053942_T_C | 33053942 | 0.38 | 0.24 | 0.60 | 3.25E-05 | 0.08 | 0.05 | 0.13 | SNP |
| chr6_33054861_A_G | 33054861 | 0.38 | 0.24 | 0.60 | 3.25E-05 | 0.08 | 0.05 | 0.13 | SNP |
| chr6_33047466_T_C | 33047466 | 0.38 | 0.24 | 0.61 | 4.42E-05 | 0.07 | 0.05 | 0.13 | SNP |
| chr6_33095615_T_C | 33095615 | 0.53 | 0.39 | 0.72 | 4.55E-05 | 0.23 | 0.19 | 0.31 | SNP |
| chr6_32605078_A_G | 32605078 | 0.43 | 0.29 | 0.65 | 4.79E-05 | 0.30 | 0.27 | 0.36 | SNP |
| chr6_33048640_A_G | 33048640 | 0.39 | 0.25 | 0.62 | 6.00E-05 | 0.10 | 0.07 | 0.16 | SNP |
| chr6_33046742_T_C | 33046742 | 0.37 | 0.23 | 0.60 | 6.02E-05 | 0.07 | 0.04 | 0.11 | SNP |
| chr6_33048466_G_T | 33048466 | 0.37 | 0.22 | 0.60 | 6.32E-05 | 0.07 | 0.04 | 0.11 | SNP |
| chr6_33048467_G_T | 33048467 | 0.37 | 0.22 | 0.60 | 6.32E-05 | 0.07 | 0.04 | 0.11 | SNP |
| chr6_33590009_A_T | 33590009 | 0.29 | 0.16 | 0.53 | 6.86E-05 | 0.05 | 0.03 | 0.08 | SNP |
| chr6_33054074_C_T | 33054074 | 0.39 | 0.24 | 0.62 | 7.17E-05 | 0.07 | 0.05 | 0.12 | SNP |
| chr6_33095801_C_T | 33095801 | 0.54 | 0.40 | 0.73 | 8.58E-05 | 0.23 | 0.19 | 0.30 | SNP |
| chr6_33050084_C_T | 33050084 | 0.37 | 0.22 | 0.61 | 9.01E-05 | 0.06 | 0.04 | 0.11 | SNP |
| chr6_33050325_A_C | 33050325 | 0.37 | 0.22 | 0.61 | 9.01E-05 | 0.06 | 0.04 | 0.11 | SNP |
| chr6_33054145_A_G | 33054145 | 0.37 | 0.22 | 0.61 | 9.01E-05 | 0.06 | 0.04 | 0.11 | SNP |
| chr6_33045147_A_T | 33045147 | 0.53 | 0.38 | 0.73 | 9.27E-05 | 0.22 | 0.18 | 0.28 | SNP |
| chr6_32559575_A_G | 32559575 | 0.43 | 0.27 | 0.65 | 0.00011 | 0.27 | 0.24 | 0.32 | SNP |
| chr6_33050036_T_C | 33050036 | 0.38 | 0.23 | 0.62 | 0.00013 | 0.06 | 0.04 | 0.11 | SNP |
| chr6_32551665_A_G | 32551665 | 0.45 | 0.30 | 0.68 | 0.00014 | 0.28 | 0.24 | 0.34 | SNP |
| chr6_33046915_T_A | 33046915 | 0.38 | 0.23 | 0.62 | 0.00014 | 0.06 | 0.04 | 0.11 | SNP |
| chr6_32635954_G_A | 32635954 | 0.47 | 0.32 | 0.69 | 0.00015 | 0.22 | 0.19 | 0.28 | SNP |
| chr6_33046726_T_C | 33046726 | 0.38 | 0.23 | 0.63 | 0.00015 | 0.06 | 0.04 | 0.11 | SNP |
| chr6_33047612_G_C | 33047612 | 0.38 | 0.23 | 0.63 | 0.00015 | 0.06 | 0.04 | 0.11 | SNP |
| chr6_33596664_A_C | 33596664 | 0.31 | 0.16 | 0.56 | 0.00016 | 0.05 | 0.03 | 0.08 | SNP |
| chr6_32603660_T_C | 32603660 | 0.53 | 0.38 | 0.74 | 0.00017 | 0.41 | 0.38 | 0.49 | SNP |
| chr6_33053352_C_T | 33053352 | 0.41 | 0.25 | 0.65 | 0.00017 | 0.07 | 0.05 | 0.12 | SNP |
| chr6_32559648_G_T | 32559648 | 0.46 | 0.30 | 0.69 | 0.00018 | 0.30 | 0.27 | 0.36 | SNP |
| chr6_33095636_A_G | 33095636 | 0.56 | 0.41 | 0.76 | 0.00018 | 0.23 | 0.19 | 0.30 | SNP |
| chr6_32559710_C_T | 32559710 | 0.43 | 0.28 | 0.67 | 0.00019 | 0.30 | 0.27 | 0.36 | SNP |
| chr6_32605583_A_G | 32605583 | 0.53 | 0.38 | 0.74 | 0.00019 | 0.41 | 0.37 | 0.48 | SNP |
| chr6_32605132_T_C | 32605132 | 0.48 | 0.33 | 0.71 | 0.00020 | 0.36 | 0.33 | 0.42 | SNP |
| chr6_32487677_T_C | 32487677 | 0.42 | 0.26 | 0.66 | 0.00020 | 0.25 | 0.22 | 0.31 | SNP |
| chr6_32611467_A_C | 32611467 | 0.45 | 0.30 | 0.68 | 0.00020 | 0.28 | 0.25 | 0.34 | SNP |
| chr6_33048102_C_T | 33048102 | 0.36 | 0.21 | 0.62 | 0.00021 | 0.06 | 0.04 | 0.10 | SNP |
| chr6_33095836_G_A | 33095836 | 0.56 | 0.41 | 0.76 | 0.00024 | 0.23 | 0.19 | 0.30 | SNP |
| chr6_33048606_G_C | 33048606 | 0.36 | 0.21 | 0.62 | 0.00025 | 0.07 | 0.05 | 0.11 | SNP |
| chr6_33178010_G_T | 33178010 | 0.48 | 0.32 | 0.71 | 0.00030 | 0.10 | 0.08 | 0.16 | SNP |
| chr6_33041734_T_C | 33041734 | 0.42 | 0.26 | 0.67 | 0.00030 | 0.08 | 0.05 | 0.11 | SNP |
| chr6_33166034_A_G | 33166034 | 0.46 | 0.30 | 0.70 | 0.00034 | 0.10 | 0.07 | 0.15 | SNP |
| chr6_33095684_A_G | 33095684 | 0.58 | 0.43 | 0.78 | 0.00036 | 0.24 | 0.20 | 0.30 | SNP |
| chr6_33045558_G_A | 33045558 | 0.57 | 0.41 | 0.77 | 0.00037 | 0.20 | 0.17 | 0.25 | SNP |
| chr6_33048628_A_C | 33048628 | 0.37 | 0.22 | 0.64 | 0.00037 | 0.07 | 0.05 | 0.11 | SNP |
| chr6_32629259_T_C | 32629259 | 0.49 | 0.33 | 0.72 | 0.00038 | 0.23 | 0.20 | 0.28 | SNP |
| chr6_32628195_A_G | 32628195 | 0.36 | 0.20 | 0.62 | 0.00040 | 0.05 | 0.03 | 0.09 | SNP |
| chr6_33095555_T_A | 33095555 | 0.37 | 0.21 | 0.64 | 0.00041 | 0.05 | 0.04 | 0.09 | SNP |
| chr6_33032851_T_C | 33032851 | 0.43 | 0.27 | 0.69 | 0.00041 | 0.08 | 0.06 | 0.12 | SNP |
| chr6_33037199_G_C | 33037199 | 0.43 | 0.27 | 0.69 | 0.00041 | 0.08 | 0.06 | 0.12 | SNP |
| chr6_32629403_C_T | 32629403 | 0.50 | 0.34 | 0.73 | 0.00045 | 0.23 | 0.20 | 0.28 | SNP |
| chr6_33033274_T_C | 33033274 | 0.44 | 0.27 | 0.69 | 0.00048 | 0.08 | 0.06 | 0.12 | SNP |
| chr6_31237605_G_A | 31237605 | 1.83 | 1.31 | 2.58 | 0.00052 | 0.22 | 0.25 | 0.16 | SNP |
| chr6_32609094_C_T | 32609094 | 0.54 | 0.38 | 0.76 | 0.00054 | 0.41 | 0.37 | 0.46 | SNP |
| chr6_33095748_C_G | 33095748 | 0.59 | 0.43 | 0.79 | 0.00054 | 0.23 | 0.20 | 0.30 | SNP |
| chr6_33547440_G_C | 33547440 | 1.72 | 1.27 | 2.36 | 0.00055 | 0.43 | 0.47 | 0.35 | SNP |
| chr6_33095762_G_A | 33095762 | 0.38 | 0.22 | 0.66 | 0.00056 | 0.05 | 0.04 | 0.09 | SNP |
| chr6_33160425_G_T | 33160425 | 0.48 | 0.32 | 0.73 | 0.00056 | 0.09 | 0.07 | 0.14 | SNP |
| chr6_33095737_G_A | 33095737 | 0.59 | 0.43 | 0.79 | 0.00057 | 0.23 | 0.20 | 0.30 | SNP |
| chr6_33043520_C_G | 33043520 | 0.44 | 0.28 | 0.70 | 0.00060 | 0.08 | 0.06 | 0.11 | SNP |
| chr6_33048380_G_A | 33048380 | 0.40 | 0.24 | 0.67 | 0.00061 | 0.06 | 0.04 | 0.10 | SNP |
| chr6_33143756_A_G | 33143756 | 0.50 | 0.33 | 0.74 | 0.00066 | 0.10 | 0.08 | 0.14 | SNP |
| chr6_33046752_G_C | 33046752 | 0.40 | 0.24 | 0.68 | 0.00066 | 0.06 | 0.04 | 0.10 | SNP |
| chr6_33037557_C_T | 33037557 | 0.26 | 0.12 | 0.56 | 0.00068 | 0.03 | 0.02 | 0.05 | SNP |
| chr6_33037560_G_A | 33037560 | 0.26 | 0.12 | 0.56 | 0.00068 | 0.03 | 0.02 | 0.05 | SNP |
| xHLA_2field_DQB1_03_02 | 32627244 | 0.48 | 0.31 | 0.73 | 0.00070 | 0.22 | 0.19 | 0.29 | HLA (xHLA) |
| chr6_33037522_T_C | 33037522 | 0.35 | 0.19 | 0.64 | 0.00072 | 0.04 | 0.03 | 0.07 | SNP |
| chr6_33033313_G_T | 33033313 | 0.28 | 0.13 | 0.57 | 0.00074 | 0.03 | 0.02 | 0.05 | SNP |
| chr6_33039681_A_G | 33039681 | 0.29 | 0.14 | 0.59 | 0.00076 | 0.03 | 0.02 | 0.05 | SNP |
| chr6_33039694_G_T | 33039694 | 0.29 | 0.14 | 0.59 | 0.00076 | 0.03 | 0.02 | 0.05 | SNP |
| chr6_33039697_T_G | 33039697 | 0.29 | 0.14 | 0.59 | 0.00076 | 0.03 | 0.02 | 0.05 | SNP |
| chr6_33040044_A_G | 33040044 | 0.29 | 0.14 | 0.59 | 0.00076 | 0.03 | 0.02 | 0.05 | SNP |
| chr6_33040654_A_G | 33040654 | 0.29 | 0.14 | 0.59 | 0.00076 | 0.03 | 0.02 | 0.05 | SNP |
| chr6_33042368_A_G | 33042368 | 0.29 | 0.14 | 0.59 | 0.00076 | 0.03 | 0.02 | 0.05 | SNP |
| chr6_33042406_C_G | 33042406 | 0.29 | 0.14 | 0.59 | 0.00076 | 0.03 | 0.02 | 0.05 | SNP |
| chr6_33042492_T_C | 33042492 | 0.29 | 0.14 | 0.59 | 0.00076 | 0.03 | 0.02 | 0.05 | SNP |
| chr6_33042880_A_G | 33042880 | 0.29 | 0.14 | 0.59 | 0.00076 | 0.03 | 0.02 | 0.05 | SNP |
| chr6_33044188_T_A | 33044188 | 0.29 | 0.14 | 0.59 | 0.00076 | 0.03 | 0.02 | 0.05 | SNP |
| chr6_33044258_G_A | 33044258 | 0.29 | 0.14 | 0.59 | 0.00076 | 0.03 | 0.02 | 0.05 | SNP |
| chr6_33044388_T_C | 33044388 | 0.29 | 0.14 | 0.59 | 0.00076 | 0.03 | 0.02 | 0.05 | SNP |
| chr6_32559702_A_G | 32559702 | 0.47 | 0.30 | 0.73 | 0.00077 | 0.31 | 0.28 | 0.36 | SNP |
| chr6_32369488_G_A | 32369488 | 0.47 | 0.30 | 0.73 | 0.00082 | 0.10 | 0.08 | 0.14 | SNP |
| chr6_33036959_G_A | 33036959 | 0.35 | 0.18 | 0.64 | 0.00082 | 0.04 | 0.03 | 0.07 | SNP |
| chr6_32782030_A_C | 32782030 | 0.56 | 0.39 | 0.78 | 0.00083 | 0.19 | 0.16 | 0.24 | SNP |
| chr6_33041226_C_T | 33041226 | 0.37 | 0.20 | 0.66 | 0.00084 | 0.04 | 0.03 | 0.07 | SNP |
| chr6_33039606_C_T | 33039606 | 0.37 | 0.20 | 0.66 | 0.00087 | 0.04 | 0.03 | 0.07 | SNP |
| chr6_33039686_T_C | 33039686 | 0.37 | 0.20 | 0.66 | 0.00087 | 0.04 | 0.03 | 0.07 | SNP |
| chr6_33039719_A_G | 33039719 | 0.37 | 0.20 | 0.66 | 0.00087 | 0.04 | 0.03 | 0.07 | SNP |
| chr6_33039767_A_G | 33039767 | 0.37 | 0.20 | 0.66 | 0.00087 | 0.04 | 0.03 | 0.07 | SNP |
| chr6_33039837_G_A | 33039837 | 0.37 | 0.20 | 0.66 | 0.00087 | 0.04 | 0.03 | 0.07 | SNP |
| chr6_33039980_A_G | 33039980 | 0.37 | 0.20 | 0.66 | 0.00087 | 0.04 | 0.03 | 0.07 | SNP |
| chr6_33040596_A_G | 33040596 | 0.37 | 0.20 | 0.66 | 0.00087 | 0.04 | 0.03 | 0.07 | SNP |
| chr6_33040715_G_A | 33040715 | 0.37 | 0.20 | 0.66 | 0.00087 | 0.04 | 0.03 | 0.07 | SNP |
| chr6_33040736_T_C | 33040736 | 0.37 | 0.20 | 0.66 | 0.00087 | 0.04 | 0.03 | 0.07 | SNP |
| chr6_33040744_G_C | 33040744 | 0.37 | 0.20 | 0.66 | 0.00087 | 0.04 | 0.03 | 0.07 | SNP |
| chr6_33040781_A_G | 33040781 | 0.37 | 0.20 | 0.66 | 0.00087 | 0.04 | 0.03 | 0.07 | SNP |
| chr6_33040798_T_C | 33040798 | 0.37 | 0.20 | 0.66 | 0.00087 | 0.04 | 0.03 | 0.07 | SNP |
| chr6_33040835_A_G | 33040835 | 0.37 | 0.20 | 0.66 | 0.00087 | 0.04 | 0.03 | 0.07 | SNP |
| chr6_33040877_T_C | 33040877 | 0.37 | 0.20 | 0.66 | 0.00087 | 0.04 | 0.03 | 0.07 | SNP |
| chr6_33041034_A_G | 33041034 | 0.37 | 0.20 | 0.66 | 0.00087 | 0.04 | 0.03 | 0.07 | SNP |
| chr6_33041176_C_T | 33041176 | 0.37 | 0.20 | 0.66 | 0.00087 | 0.04 | 0.03 | 0.07 | SNP |
| chr6_33043930_A_G | 33043930 | 0.37 | 0.20 | 0.66 | 0.00087 | 0.04 | 0.03 | 0.07 | SNP |
| chr6_33044257_T_C | 33044257 | 0.37 | 0.20 | 0.66 | 0.00087 | 0.04 | 0.03 | 0.07 | SNP |
| chr6_33138839_A_T | 33138839 | 0.42 | 0.25 | 0.70 | 0.00087 | 0.07 | 0.05 | 0.11 | SNP |
| chr6_33032822_G_A | 33032822 | 0.36 | 0.19 | 0.65 | 0.00088 | 0.04 | 0.03 | 0.07 | SNP |
| chr6_33037080_G_A | 33037080 | 0.36 | 0.19 | 0.65 | 0.00088 | 0.04 | 0.03 | 0.07 | SNP |
| chr6_33037082_A_G | 33037082 | 0.36 | 0.19 | 0.65 | 0.00088 | 0.04 | 0.03 | 0.07 | SNP |
| chr6_33037104_C_T | 33037104 | 0.36 | 0.19 | 0.65 | 0.00092 | 0.04 | 0.03 | 0.07 | SNP |
| chr6_33033235_G_C | 33033235 | 0.36 | 0.19 | 0.65 | 0.00093 | 0.04 | 0.03 | 0.07 | SNP |
| chr6_33033287_G_C | 33033287 | 0.36 | 0.20 | 0.66 | 0.00097 | 0.04 | 0.03 | 0.07 | SNP |
| chr6_33046662_T_C | 33046662 | 0.59 | 0.43 | 0.81 | 0.00098 | 0.20 | 0.17 | 0.24 | SNP |
| chr6_32376517_C_T | 32376517 | 0.48 | 0.31 | 0.74 | 0.00104 | 0.09 | 0.07 | 0.14 | SNP |
| chr6_31275663_C_A | 31275663 | 0.48 | 0.31 | 0.75 | 0.00106 | 0.16 | 0.14 | 0.20 | SNP |

| | | | | | | | | | |
|---|---|---|---|---|---|---|---|---|---|
| chr6_33041186_G_A | 33041186 | 0.38 | 0.20 | 0.67 | 0.00113 | 0.04 | 0.03 | 0.07 | SNP |
| chr6_33095516_G_A | 33095516 | 0.57 | 0.41 | 0.80 | 0.00116 | 0.23 | 0.19 | 0.29 | SNP |
| chr6_31237087_C_A | 31237087 | 2.26 | 1.41 | 3.79 | 0.00118 | 0.10 | 0.13 | 0.06 | SNP |
| chr6_33032865_A_C | 33032865 | 0.37 | 0.20 | 0.67 | 0.00120 | 0.04 | 0.03 | 0.07 | SNP |
| chr6_33033058_T_C | 33033058 | 0.37 | 0.20 | 0.67 | 0.00120 | 0.04 | 0.03 | 0.07 | SNP |
| chr6_33033146_T_C | 33033146 | 0.37 | 0.20 | 0.67 | 0.00120 | 0.04 | 0.03 | 0.07 | SNP |
| chr6_33033170_T_C | 33033170 | 0.37 | 0.20 | 0.67 | 0.00120 | 0.04 | 0.03 | 0.07 | SNP |
| chr6_33033177_G_A | 33033177 | 0.37 | 0.20 | 0.67 | 0.00120 | 0.04 | 0.03 | 0.07 | SNP |
| chr6_33033178_G_A | 33033178 | 0.37 | 0.20 | 0.67 | 0.00120 | 0.04 | 0.03 | 0.07 | SNP |
| chr6_33036003_G_C | 33036003 | 0.35 | 0.18 | 0.65 | 0.00121 | 0.04 | 0.03 | 0.06 | SNP |
| chr6_33033202_C_T | 33033202 | 0.37 | 0.20 | 0.67 | 0.00124 | 0.04 | 0.03 | 0.07 | SNP |
| chr6_33033188_C_T | 33033188 | 0.37 | 0.20 | 0.67 | 0.00124 | 0.04 | 0.03 | 0.07 | SNP |
| chr6_32610349_C_A | 32610349 | 0.59 | 0.42 | 0.81 | 0.00128 | 0.47 | 0.44 | 0.53 | SNP |
| chr6_33036951_A_G | 33036951 | 0.36 | 0.19 | 0.67 | 0.00132 | 0.04 | 0.03 | 0.07 | SNP |
| chr6_32798283_T_C | 32798283 | 0.49 | 0.31 | 0.75 | 0.00135 | 0.09 | 0.08 | 0.13 | SNP |
| chr6_33037675_C_A | 33037675 | 0.38 | 0.21 | 0.68 | 0.00137 | 0.04 | 0.03 | 0.07 | SNP |
| chr6_33037690_C_T | 33037690 | 0.38 | 0.21 | 0.68 | 0.00137 | 0.04 | 0.03 | 0.07 | SNP |
| chr6_32407068_G_A | 32407068 | 0.28 | 0.12 | 0.60 | 0.00138 | 0.03 | 0.01 | 0.05 | SNP |
| chr6_33033357_C_G | 33033357 | 0.36 | 0.19 | 0.67 | 0.00139 | 0.04 | 0.03 | 0.07 | SNP |
| chr6_33037768_G_A | 33037768 | 0.38 | 0.21 | 0.68 | 0.00142 | 0.04 | 0.03 | 0.07 | SNP |
| chr6_33143136_G_A | 33143136 | 0.43 | 0.25 | 0.72 | 0.00143 | 0.08 | 0.06 | 0.12 | SNP |
| chr6_32551700_T_G | 32551700 | 0.50 | 0.33 | 0.76 | 0.00145 | 0.25 | 0.22 | 0.30 | SNP |
| chr6_33041064_C_A | 33041064 | 0.44 | 0.27 | 0.73 | 0.00160 | 0.06 | 0.04 | 0.10 | SNP |
| chr6_33546825_T_C | 33546825 | 1.58 | 1.19 | 2.12 | 0.00164 | 0.43 | 0.46 | 0.36 | SNP |
| chr6_32798350_A_C | 32798350 | 0.49 | 0.31 | 0.76 | 0.00166 | 0.09 | 0.08 | 0.13 | SNP |
| chr6_32603939_G_T | 32603939 | 1.82 | 1.26 | 2.65 | 0.00168 | 0.33 | 0.36 | 0.26 | SNP |
| chr6_33037579_A_T | 33037579 | 0.36 | 0.19 | 0.68 | 0.00170 | 0.04 | 0.03 | 0.06 | SNP |
| chr6_33037580_T_G | 33037580 | 0.36 | 0.19 | 0.68 | 0.00170 | 0.04 | 0.03 | 0.06 | SNP |
| chr6_33037294_T_C | 33037294 | 0.37 | 0.20 | 0.69 | 0.00171 | 0.04 | 0.03 | 0.07 | SNP |
| chr6_33037304_G_T | 33037304 | 0.37 | 0.20 | 0.69 | 0.00171 | 0.04 | 0.03 | 0.07 | SNP |
| chr6_33037419_G_A | 33037419 | 0.37 | 0.20 | 0.69 | 0.00171 | 0.04 | 0.03 | 0.07 | SNP |
| chr6_33037424_T_C | 33037424 | 0.37 | 0.20 | 0.69 | 0.00171 | 0.04 | 0.03 | 0.07 | SNP |
| chr6_31245534_A_G | 31245534 | 2.20 | 1.36 | 3.68 | 0.00181 | 0.10 | 0.12 | 0.06 | SNP |
| chr6_33039295_T_A | 33039295 | 0.38 | 0.21 | 0.70 | 0.00185 | 0.04 | 0.03 | 0.07 | SNP |
| chr6_33033266_T_A | 33033266 | 0.37 | 0.20 | 0.69 | 0.00193 | 0.04 | 0.03 | 0.06 | SNP |
| chr6_33095584_G_C | 33095584 | 1.95 | 1.29 | 3.02 | 0.00196 | 0.14 | 0.16 | 0.09 | SNP |
| chr6_31238522_C_T | 31238522 | 1.72 | 1.23 | 2.44 | 0.00199 | 0.22 | 0.25 | 0.16 | SNP |
| chr6_33033267_G_A | 33033267 | 0.37 | 0.20 | 0.69 | 0.00199 | 0.04 | 0.03 | 0.06 | SNP |
| chr6_33039729_T_G | 33039729 | 0.38 | 0.21 | 0.70 | 0.00202 | 0.04 | 0.03 | 0.07 | SNP |
| chr6_31236201_G_A | 31236201 | 2.15 | 1.34 | 3.57 | 0.00206 | 0.11 | 0.13 | 0.07 | SNP |
| chr6_32189032_T_C | 32189032 | 1.63 | 1.20 | 2.24 | 0.00206 | 0.32 | 0.35 | 0.27 | SNP |
| chr6_31240771_T_C | 31240771 | 2.14 | 1.34 | 3.56 | 0.00212 | 0.11 | 0.13 | 0.07 | SNP |
| chr6_31240932_C_A | 31240932 | 2.14 | 1.34 | 3.56 | 0.00212 | 0.11 | 0.13 | 0.07 | SNP |
| chr6_31241002_A_G | 31241002 | 2.14 | 1.34 | 3.56 | 0.00212 | 0.11 | 0.13 | 0.07 | SNP |
| chr6_33036085_C_G | 33036085 | 0.40 | 0.22 | 0.72 | 0.00218 | 0.04 | 0.03 | 0.07 | SNP |
| chr6_33036116_T_G | 33036116 | 0.40 | 0.22 | 0.72 | 0.00218 | 0.04 | 0.03 | 0.07 | SNP |
| chr6_33036132_A_G | 33036132 | 0.40 | 0.22 | 0.72 | 0.00218 | 0.04 | 0.03 | 0.07 | SNP |
| chr6_33036133_G_A | 33036133 | 0.40 | 0.22 | 0.72 | 0.00218 | 0.04 | 0.03 | 0.07 | SNP |
| chr6_33036853_A_C | 33036853 | 0.40 | 0.22 | 0.72 | 0.00218 | 0.04 | 0.03 | 0.07 | SNP |
| chr6_33036971_C_T | 33036971 | 0.40 | 0.22 | 0.72 | 0.00218 | 0.04 | 0.03 | 0.07 | SNP |
| chr6_33036999_T_C | 33036999 | 0.40 | 0.22 | 0.72 | 0.00218 | 0.04 | 0.03 | 0.07 | SNP |
| chr6_33037187_C_G | 33037187 | 0.40 | 0.22 | 0.72 | 0.00218 | 0.04 | 0.03 | 0.07 | SNP |
| chr6_33037279_A_G | 33037279 | 0.40 | 0.22 | 0.72 | 0.00218 | 0.04 | 0.03 | 0.07 | SNP |
| chr6_32605578_G_A | 32605578 | 1.79 | 1.24 | 2.61 | 0.00222 | 0.33 | 0.36 | 0.26 | SNP |
| chr6_33037142_A_G | 33037142 | 0.40 | 0.22 | 0.72 | 0.00222 | 0.04 | 0.03 | 0.07 | SNP |
| chr6_32406122_G_A | 32406122 | 4.41 | 1.87 | 12.99 | 0.00223 | 0.04 | 0.05 | 0.01 | SNP |
| chr6_31244082_C_T | 31244082 | 2.16 | 1.34 | 3.63 | 0.00227 | 0.10 | 0.12 | 0.06 | SNP |
| chr6_33040478_C_A | 33040478 | 0.33 | 0.16 | 0.66 | 0.00233 | 0.03 | 0.02 | 0.05 | SNP |
| chr6_32408932_G_A | 32408932 | 4.37 | 1.85 | 12.89 | 0.00235 | 0.04 | 0.05 | 0.01 | SNP |
| chr6_32411573_C_T | 32411573 | 4.37 | 1.85 | 12.89 | 0.00235 | 0.04 | 0.05 | 0.01 | SNP |
| chr6_33039503_C_T | 33039503 | 0.39 | 0.21 | 0.71 | 0.00245 | 0.04 | 0.03 | 0.07 | SNP |
| chr6_32441100_C_A | 32441100 | 1.86 | 1.26 | 2.82 | 0.00248 | 0.27 | 0.28 | 0.24 | SNP |
| chr6_33033351_G_T | 33033351 | 0.38 | 0.20 | 0.71 | 0.00257 | 0.04 | 0.03 | 0.07 | SNP |
| chr6_33095865_C_T | 33095865 | 1.91 | 1.27 | 2.95 | 0.00271 | 0.14 | 0.16 | 0.09 | SNP |
| chr6_33041073_A_C | 33041073 | 0.38 | 0.19 | 0.71 | 0.00272 | 0.04 | 0.03 | 0.06 | SNP |
| chr6_33132365_T_C | 33132365 | 0.22 | 0.07 | 0.57 | 0.00281 | 0.02 | 0.01 | 0.04 | SNP |
| chr6_33556605_G_C | 33556605 | 0.60 | 0.43 | 0.84 | 0.00306 | 0.17 | 0.15 | 0.22 | SNP |
| chr6_31248819_C_T | 31248819 | 2.22 | 1.33 | 3.86 | 0.00309 | 0.10 | 0.12 | 0.06 | SNP |
| chr6_31549556_T_G | 31549556 | 3.49 | 1.62 | 8.73 | 0.00319 | 0.04 | 0.06 | 0.02 | SNP |
| chr6_33036210_G_T | 33036210 | 0.41 | 0.22 | 0.74 | 0.00335 | 0.04 | 0.03 | 0.07 | SNP |
| chr6_33036233_A_G | 33036233 | 0.41 | 0.22 | 0.74 | 0.00335 | 0.04 | 0.03 | 0.07 | SNP |
| chr6_33036242_T_C | 33036242 | 0.41 | 0.22 | 0.74 | 0.00335 | 0.04 | 0.03 | 0.07 | SNP |
| chr6_33036287_G_A | 33036287 | 0.41 | 0.22 | 0.74 | 0.00335 | 0.04 | 0.03 | 0.07 | SNP |
| chr6_33036288_G_A | 33036288 | 0.41 | 0.22 | 0.74 | 0.00335 | 0.04 | 0.03 | 0.07 | SNP |
| chr6_33036312_A_G | 33036312 | 0.41 | 0.22 | 0.74 | 0.00335 | 0.04 | 0.03 | 0.07 | SNP |
| chr6_33035980_T_A | 33035980 | 0.38 | 0.20 | 0.72 | 0.00340 | 0.04 | 0.03 | 0.06 | SNP |
| chr6_33174783_A_G | 33174783 | 0.59 | 0.42 | 0.84 | 0.00346 | 0.14 | 0.12 | 0.19 | SNP |
| chr6_33035974_A_G | 33035974 | 0.38 | 0.20 | 0.73 | 0.00354 | 0.04 | 0.03 | 0.06 | SNP |
| chr6_33037865_C_T | 33037865 | 0.39 | 0.20 | 0.73 | 0.00370 | 0.04 | 0.03 | 0.06 | SNP |
| chr6_33039405_C_G | 33039405 | 0.41 | 0.22 | 0.74 | 0.00374 | 0.04 | 0.03 | 0.06 | SNP |
| chr6_33050083_C_A | 33050083 | 0.26 | 0.10 | 0.63 | 0.00379 | 0.02 | 0.01 | 0.04 | SNP |
| chr6_31240959_C_G | 31240959 | 1.60 | 1.17 | 2.22 | 0.00381 | 0.24 | 0.27 | 0.19 | SNP |
| chr6_32628913_G_T | 32628913 | 0.54 | 0.35 | 0.82 | 0.00384 | 0.27 | 0.24 | 0.31 | SNP |
| chr6_32184705_A_G | 32184705 | 0.61 | 0.44 | 0.85 | 0.00392 | 0.32 | 0.29 | 0.39 | SNP |
| chr6_33048348_G_A | 33048348 | 0.35 | 0.17 | 0.71 | 0.00402 | 0.03 | 0.02 | 0.05 | SNP |
| chr6_32362447_C_T | 32362447 | 4.06 | 1.71 | 12.02 | 0.00408 | 0.04 | 0.05 | 0.01 | SNP |
| chr6_32373796_A_G | 32373796 | 4.06 | 1.71 | 12.02 | 0.00408 | 0.04 | 0.05 | 0.01 | SNP |
| chr6_33037891_C_T | 33037891 | 0.36 | 0.18 | 0.72 | 0.00419 | 0.04 | 0.02 | 0.06 | SNP |
| chr6_32376471_A_C | 32376471 | 1.77 | 1.21 | 2.65 | 0.00424 | 0.18 | 0.20 | 0.14 | SNP |
| chr6_33036290_G_A | 33036290 | 1.58 | 1.16 | 2.17 | 0.00429 | 0.24 | 0.27 | 0.19 | SNP |
| chr6_33641379_C_T | 33641379 | 1.45 | 1.13 | 1.88 | 0.00430 | 0.47 | 0.50 | 0.40 | SNP |
| chr6_32408917_C_T | 32408917 | 1.70 | 1.19 | 2.47 | 0.00432 | 0.28 | 0.30 | 0.25 | SNP |
| chr6_33543409_A_C | 33543409 | 1.52 | 1.14 | 2.03 | 0.00443 | 0.44 | 0.47 | 0.37 | SNP |
| chr6_31669295_A_G | 31669295 | 3.37 | 1.55 | 8.48 | 0.00447 | 0.04 | 0.05 | 0.02 | SNP |
| chr6_33158189_C_A | 33158189 | 1.45 | 1.12 | 1.87 | 0.00448 | 0.45 | 0.48 | 0.39 | SNP |
| chr6_33039804_C_T | 33039804 | 1.55 | 1.15 | 2.11 | 0.00448 | 0.25 | 0.28 | 0.20 | SNP |
| chr6_32299592_G_A | 32299592 | 1.70 | 1.19 | 2.49 | 0.00460 | 0.29 | 0.33 | 0.23 | SNP |
| chr6_33033390_A_T | 33033390 | 0.35 | 0.17 | 0.72 | 0.00473 | 0.04 | 0.02 | 0.06 | SNP |
| chr6_32559673_T_G | 32559673 | 0.61 | 0.43 | 0.86 | 0.00477 | 0.38 | 0.35 | 0.44 | SNP |
| chr6_33045823_A_G | 33045823 | 0.40 | 0.21 | 0.75 | 0.00484 | 0.04 | 0.03 | 0.06 | SNP |
| chr6_33036401_C_T | 33036401 | 0.41 | 0.22 | 0.76 | 0.00485 | 0.04 | 0.03 | 0.06 | SNP |
| chr6_32610314_T_C | 32610314 | 1.70 | 1.18 | 2.47 | 0.00486 | 0.32 | 0.36 | 0.26 | SNP |
| chr6_33036617_C_T | 33036617 | 0.41 | 0.22 | 0.76 | 0.00490 | 0.04 | 0.03 | 0.06 | SNP |
| chr6_33036630_G_T | 33036630 | 0.41 | 0.22 | 0.76 | 0.00490 | 0.04 | 0.03 | 0.06 | SNP |
| chr6_33036631_A_C | 33036631 | 0.41 | 0.22 | 0.76 | 0.00490 | 0.04 | 0.03 | 0.06 | SNP |
| chr6_32628148_C_T | 32628148 | 0.56 | 0.37 | 0.84 | 0.00499 | 0.23 | 0.21 | 0.27 | SNP |
| chr6_33136638_A_G | 33136638 | 1.45 | 1.12 | 1.88 | 0.00500 | 0.43 | 0.46 | 0.37 | SNP |
| chr6_33077588_A_G | 33077588 | 0.29 | 0.11 | 0.67 | 0.00507 | 0.02 | 0.02 | 0.05 | SNP |
| chr6_32409056_G_A | 32409056 | 1.64 | 1.17 | 2.35 | 0.00521 | 0.30 | 0.31 | 0.27 | SNP |
| chr6_33079626_T_C | 33079626 | 0.24 | 0.09 | 0.65 | 0.00523 | 0.02 | 0.01 | 0.03 | SNP |
| chr6_32185796_C_T | 32185796 | 3.91 | 1.64 | 11.61 | 0.00530 | 0.04 | 0.05 | 0.01 | SNP |
| chr6_31237776_C_T | 31237776 | 1.59 | 1.15 | 2.21 | 0.00537 | 0.24 | 0.26 | 0.19 | SNP |
| chr6_31707526_G_A | 31707526 | 4.55 | 1.74 | 15.63 | 0.00547 | 0.03 | 0.04 | 0.01 | SNP |
| chr6_33039484_A_G | 33039484 | 0.41 | 0.22 | 0.77 | 0.00559 | 0.04 | 0.03 | 0.06 | SNP |
| chr6_32410137_C_T | 32410137 | 1.64 | 1.16 | 2.33 | 0.00561 | 0.30 | 0.31 | 0.27 | SNP |
| chr6_32340070_C_T | 32340070 | 0.63 | 0.45 | 0.87 | 0.00561 | 0.45 | 0.43 | 0.48 | SNP |
| chr6_33048663_G_A | 33048663 | 0.50 | 0.31 | 0.82 | 0.00564 | 0.08 | 0.07 | 0.12 | SNP |
| chr6_32612397_A_G | 32612397 | 0.60 | 0.41 | 0.86 | 0.00571 | 0.43 | 0.45 | 0.39 | SNP |
| chr6_33036177_A_G | 33036177 | 0.43 | 0.23 | 0.78 | 0.00575 | 0.04 | 0.03 | 0.06 | SNP |
| chr6_33036505_G_C | 33036505 | 0.43 | 0.23 | 0.78 | 0.00575 | 0.04 | 0.03 | 0.06 | SNP |
| chr6_33049726_G_T | 33049726 | 0.34 | 0.15 | 0.72 | 0.00582 | 0.03 | 0.02 | 0.04 | SNP |
| chr6_33049727_A_T | 33049727 | 0.34 | 0.15 | 0.72 | 0.00582 | 0.03 | 0.02 | 0.04 | SNP |
| chr6_33146053_C_G | 33146053 | 0.51 | 0.32 | 0.82 | 0.00582 | 0.07 | 0.05 | 0.10 | SNP |
| chr6_33148878_C_T | 33148878 | 0.51 | 0.32 | 0.82 | 0.00582 | 0.07 | 0.05 | 0.10 | SNP |
| chr6_33154656_G_A | 33154656 | 0.51 | 0.32 | 0.82 | 0.00582 | 0.07 | 0.05 | 0.10 | SNP |
| chr6_33045272_C_A | 33045272 | 0.62 | 0.45 | 0.87 | 0.00600 | 0.16 | 0.15 | 0.20 | SNP |
| chr6_33035969_A_G | 33035969 | 0.40 | 0.21 | 0.77 | 0.00614 | 0.04 | 0.03 | 0.06 | SNP |
| chr6_33545340_G_A | 33545340 | 0.63 | 0.45 | 0.88 | 0.00616 | 0.23 | 0.20 | 0.28 | SNP |

| | | | | | | | | | |
|---|---|---|---|---|---|---|---|---|---|
| chr6_31751071_C_T | 31751071 | 4.44 | 1.69 | 15.28 | 0.00642 | 0.03 | 0.04 | 0.01 | SNP |
| xHLA_2field_DPB1_02_01 | 33043767 | 0.54 | 0.35 | 0.84 | 0.00648 | 0.08 | 0.06 | 0.12 | HLA (xHLA) |
| chr6_33037866_A_G | 33037866 | 0.41 | 0.21 | 0.78 | 0.00663 | 0.04 | 0.03 | 0.06 | SNP |
| chr6_31592064_C_T | 31592064 | 3.85 | 1.57 | 11.60 | 0.00694 | 0.04 | 0.05 | 0.02 | SNP |
| chr6_32782112_C_T | 32782112 | 4.35 | 1.67 | 14.94 | 0.00698 | 0.03 | 0.04 | 0.01 | SNP |
| chr6_32306922_T_C | 32306922 | 3.38 | 1.50 | 9.11 | 0.00706 | 0.04 | 0.05 | 0.02 | SNP |
| chr6_31241127_T_C | 31241127 | 1.95 | 1.22 | 3.24 | 0.00715 | 0.10 | 0.12 | 0.07 | SNP |
| chr6_33048661_A_G | 33048661 | 0.51 | 0.31 | 0.83 | 0.00717 | 0.08 | 0.07 | 0.12 | SNP |
| chr6_33033388_A_C | 33033388 | 0.38 | 0.19 | 0.77 | 0.00718 | 0.04 | 0.03 | 0.06 | SNP |
| chr6_32439964_T_G | 32439964 | 1.72 | 1.16 | 2.57 | 0.00719 | 0.34 | 0.35 | 0.31 | SNP |
| chr6_33048921_A_G | 33048921 | 0.41 | 0.21 | 0.78 | 0.00720 | 0.04 | 0.03 | 0.06 | SNP |
| chr6_33141280_G_A | 33141280 | 0.53 | 0.33 | 0.84 | 0.00727 | 0.08 | 0.06 | 0.11 | SNP |
| chr6_32410576_C_T | 32410576 | 1.61 | 1.14 | 2.30 | 0.00737 | 0.29 | 0.31 | 0.27 | SNP |
| chr6_31465917_G_T | 31465917 | 4.31 | 1.65 | 14.81 | 0.00743 | 0.03 | 0.04 | 0.01 | SNP |
| chr6_31373445_T_C | 31373445 | 3.78 | 1.55 | 11.35 | 0.00750 | 0.04 | 0.05 | 0.01 | SNP |
| chr6_33036717_C_G | 33036717 | 0.41 | 0.22 | 0.79 | 0.00767 | 0.04 | 0.03 | 0.06 | SNP |
| chr6_33163451_A_G | 33163451 | 1.42 | 1.10 | 1.84 | 0.00770 | 0.45 | 0.48 | 0.40 | SNP |
| chr6_31241109_C_T | 31241109 | 1.95 | 1.21 | 3.24 | 0.00780 | 0.10 | 0.12 | 0.07 | SNP |
| chr6_33036388_C_T | 33036388 | 0.43 | 0.23 | 0.80 | 0.00783 | 0.04 | 0.03 | 0.06 | SNP |
| chr6_33036394_G_A | 33036394 | 0.43 | 0.23 | 0.80 | 0.00783 | 0.04 | 0.03 | 0.06 | SNP |
| chr6_32604152_A_G | 32604152 | 0.63 | 0.45 | 0.88 | 0.00786 | 0.47 | 0.48 | 0.44 | SNP |
| chr6_32559124_T_C | 32559124 | 0.61 | 0.42 | 0.88 | 0.00789 | 0.38 | 0.36 | 0.43 | SNP |
| chr6_33140983_G_A | 33140983 | 1.41 | 1.10 | 1.83 | 0.00793 | 0.45 | 0.48 | 0.40 | SNP |
| chr6_33137403_T_C | 33137403 | 1.40 | 1.09 | 1.80 | 0.00802 | 0.45 | 0.48 | 0.40 | SNP |
| chr6_33036708_C_T | 33036708 | 0.42 | 0.22 | 0.80 | 0.00807 | 0.04 | 0.03 | 0.06 | SNP |
| chr6_33036709_A_G | 33036709 | 0.42 | 0.22 | 0.80 | 0.00807 | 0.04 | 0.03 | 0.06 | SNP |
| chr6_33169895_C_G | 33169895 | 1.78 | 1.17 | 2.76 | 0.00816 | 0.13 | 0.15 | 0.09 | SNP |
| chr6_32370684_A_G | 32370684 | 0.61 | 0.42 | 0.88 | 0.00826 | 0.34 | 0.32 | 0.38 | SNP |
| chr6_32603729_A_G | 32603729 | 2.03 | 1.23 | 3.54 | 0.00841 | 0.09 | 0.10 | 0.05 | SNP |
| chr6_33139475_G_T | 33139475 | 1.42 | 1.10 | 1.85 | 0.00842 | 0.44 | 0.48 | 0.39 | SNP |
| chr6_33137689_C_T | 33137689 | 1.77 | 1.17 | 2.75 | 0.00855 | 0.13 | 0.14 | 0.09 | SNP |
| chr6_33144466_C_A | 33144466 | 0.32 | 0.13 | 0.73 | 0.00876 | 0.02 | 0.01 | 0.04 | SNP |
| chr6_33158912_G_A | 33158912 | 0.32 | 0.13 | 0.73 | 0.00876 | 0.02 | 0.01 | 0.04 | SNP |
| chr6_33149152_C_G | 33149152 | 0.53 | 0.32 | 0.85 | 0.00879 | 0.07 | 0.05 | 0.10 | SNP |
| chr6_33139662_G_A | 33139662 | 1.40 | 1.09 | 1.80 | 0.00885 | 0.45 | 0.48 | 0.40 | SNP |
| chr6_32370835_T_C | 32370835 | 0.64 | 0.45 | 0.89 | 0.00907 | 0.42 | 0.40 | 0.46 | SNP |
| chr6_31628216_C_T | 31628216 | 3.09 | 1.40 | 7.83 | 0.00907 | 0.04 | 0.05 | 0.02 | SNP |
| chr6_33138015_T_C | 33138015 | 0.32 | 0.13 | 0.74 | 0.00910 | 0.02 | 0.01 | 0.04 | SNP |
| chr6_33151880_C_A | 33151880 | 0.32 | 0.13 | 0.74 | 0.00910 | 0.02 | 0.01 | 0.04 | SNP |
| chr6_32978587_C_A | 32978587 | 0.37 | 0.17 | 0.77 | 0.00913 | 0.03 | 0.02 | 0.05 | SNP |
| chr6_33045699_T_C | 33045699 | 0.32 | 0.13 | 0.74 | 0.00918 | 0.02 | 0.01 | 0.04 | SNP |
| chr6_33048539_T_A | 33048539 | 0.37 | 0.17 | 0.77 | 0.00918 | 0.03 | 0.02 | 0.04 | SNP |
| chr6_31323353_G_A | 31323353 | 1.50 | 1.11 | 2.03 | 0.00932 | 0.40 | 0.42 | 0.36 | SNP |
| chr6_32339076_A_G | 32339076 | 1.64 | 1.14 | 2.40 | 0.00932 | 0.29 | 0.33 | 0.24 | SNP |
| chr6_31546495_G_C | 31546495 | 3.64 | 1.49 | 10.92 | 0.00935 | 0.03 | 0.04 | 0.01 | SNP |
| chr6_32797488_T_C | 32797488 | 0.65 | 0.47 | 0.90 | 0.00937 | 0.33 | 0.30 | 0.40 | SNP |
| chr6_32322071_G_A | 32322071 | 3.27 | 1.44 | 8.86 | 0.00948 | 0.04 | 0.05 | 0.02 | SNP |
| chr6_31323116_C_T | 31323116 | 2.88 | 1.36 | 6.86 | 0.00949 | 0.04 | 0.06 | 0.02 | SNP |
| chr6_31478960_G_A | 31478960 | 4.16 | 1.58 | 14.34 | 0.00950 | 0.03 | 0.04 | 0.01 | SNP |
| chr6_32441396_A_G | 32441396 | 1.63 | 1.13 | 2.37 | 0.00967 | 0.34 | 0.35 | 0.32 | SNP |
| chr6_33145710_G_A | 33145710 | 1.39 | 1.08 | 1.79 | 0.00973 | 0.45 | 0.48 | 0.40 | SNP |
| chr6_33035982_G_A | 33035982 | 0.41 | 0.21 | 0.81 | 0.00982 | 0.04 | 0.03 | 0.06 | SNP |
| chr6_33144373_A_G | 33144373 | 1.39 | 1.08 | 1.78 | 0.01007 | 0.45 | 0.48 | 0.40 | SNP |
| chr6_31785228_C_G | 31785228 | 1.80 | 1.16 | 2.85 | 0.01014 | 0.27 | 0.28 | 0.25 | SNP |
| chr6_33037878_G_A | 33037878 | 0.41 | 0.21 | 0.81 | 0.01017 | 0.04 | 0.03 | 0.06 | SNP |
| chr6_33135392_G_A | 33135392 | 1.48 | 1.10 | 2.01 | 0.01029 | 0.48 | 0.51 | 0.43 | SNP |
| chr6_33142253_G_C | 33142253 | 1.39 | 1.08 | 1.79 | 0.01043 | 0.45 | 0.48 | 0.40 | SNP |
| chr6_31110671_G_C | 31110671 | 0.66 | 0.48 | 0.91 | 0.01050 | 0.25 | 0.23 | 0.30 | SNP |
| chr6_33132314_G_A | 33132314 | 0.31 | 0.12 | 0.74 | 0.01051 | 0.02 | 0.01 | 0.04 | SNP |
| chr6_31543758_G_A | 31543758 | 3.58 | 1.46 | 10.75 | 0.01059 | 0.03 | 0.04 | 0.01 | SNP |
| chr6_33048937_C_G | 33048937 | 0.41 | 0.20 | 0.81 | 0.01066 | 0.03 | 0.02 | 0.05 | SNP |
| chr6_31118511_T_G | 31118511 | 0.66 | 0.48 | 0.91 | 0.01068 | 0.25 | 0.23 | 0.30 | SNP |
| chr6_32303474_G_T | 32303474 | 3.19 | 1.41 | 8.63 | 0.01079 | 0.04 | 0.05 | 0.02 | SNP |
| chr6_33035986_T_C | 33035986 | 0.42 | 0.21 | 0.82 | 0.01080 | 0.04 | 0.03 | 0.05 | SNP |
| chr6_32136029_A_G | 32136029 | 3.23 | 1.40 | 8.79 | 0.01105 | 0.04 | 0.05 | 0.02 | SNP |
| chr6_32627818_C_A | 32627818 | 0.64 | 0.45 | 0.90 | 0.01112 | 0.28 | 0.26 | 0.33 | SNP |
| chr6_31239869_T_C | 31239869 | 1.48 | 1.10 | 2.01 | 0.01115 | 0.29 | 0.31 | 0.24 | SNP |
| xHLA_2field_DRB1_07_01 | 32546552 | 2.08 | 1.21 | 3.80 | 0.01119 | 0.08 | 0.10 | 0.05 | HLA (xHLA) |
| chr6_33141920_C_G | 33141920 | 1.38 | 1.08 | 1.78 | 0.01122 | 0.45 | 0.48 | 0.40 | SNP |
| chr6_33130430_G_A | 33130430 | 0.64 | 0.45 | 0.90 | 0.01129 | 0.15 | 0.13 | 0.19 | SNP |
| chr6_31464091_G_A | 31464091 | 3.52 | 1.44 | 10.58 | 0.01146 | 0.03 | 0.04 | 0.01 | SNP |
| chr6_31474820_C_T | 31474820 | 3.52 | 1.44 | 10.58 | 0.01146 | 0.03 | 0.04 | 0.01 | SNP |
| chr6_31235505_G_A | 31235505 | 1.80 | 1.15 | 2.88 | 0.01162 | 0.23 | 0.26 | 0.18 | SNP |
| chr6_33161661_G_A | 33161661 | 1.38 | 1.08 | 1.77 | 0.01186 | 0.45 | 0.48 | 0.40 | SNP |
| chr6_32608844_A_G | 32608844 | 0.63 | 0.44 | 0.90 | 0.01188 | 0.37 | 0.34 | 0.42 | SNP |
| chr6_33138498_C_T | 33138498 | 1.39 | 1.08 | 1.80 | 0.01197 | 0.46 | 0.49 | 0.41 | SNP |
| chr6_33047898_C_A | 33047898 | 0.39 | 0.18 | 0.81 | 0.01200 | 0.03 | 0.02 | 0.04 | SNP |
| chr6_32605067_C_G | 32605067 | 1.94 | 1.18 | 3.34 | 0.01205 | 0.09 | 0.10 | 0.05 | SNP |
| chr6_32609476_T_C | 32609476 | 1.95 | 1.18 | 3.36 | 0.01229 | 0.09 | 0.10 | 0.05 | SNP |
| chr6_31322108_T_G | 31322108 | 3.17 | 1.37 | 8.64 | 0.01240 | 0.04 | 0.05 | 0.02 | SNP |
| chr6_32605326_A_G | 32605326 | 1.93 | 1.18 | 3.32 | 0.01252 | 0.09 | 0.10 | 0.05 | SNP |
| chr6_33054378_G_A | 33054378 | 0.36 | 0.16 | 0.79 | 0.01255 | 0.02 | 0.02 | 0.04 | SNP |
| chr6_33054559_T_C | 33054559 | 0.36 | 0.16 | 0.79 | 0.01255 | 0.02 | 0.02 | 0.04 | SNP |
| chr6_33162215_C_A | 33162215 | 1.37 | 1.07 | 1.77 | 0.01258 | 0.45 | 0.48 | 0.40 | SNP |
| chr6_33542661_G_T | 33542661 | 0.68 | 0.51 | 0.92 | 0.01261 | 0.23 | 0.20 | 0.27 | SNP |
| chr6_32188603_C_T | 32188603 | 1.47 | 1.09 | 2.01 | 0.01265 | 0.32 | 0.34 | 0.28 | SNP |
| chr6_32796793_G_A | 32796793 | 0.57 | 0.37 | 0.89 | 0.01271 | 0.09 | 0.07 | 0.12 | SNP |
| chr6_33138955_A_G | 33138955 | 1.37 | 1.07 | 1.76 | 0.01278 | 0.45 | 0.48 | 0.41 | SNP |
| chr6_31246969_A_G | 31246969 | 0.54 | 0.34 | 0.88 | 0.01286 | 0.09 | 0.08 | 0.12 | SNP |
| chr6_32603936_A_G | 32603936 | 1.68 | 1.12 | 2.55 | 0.01288 | 0.27 | 0.30 | 0.21 | SNP |
| chr6_32605825_G_A | 32605825 | 1.93 | 1.17 | 3.31 | 0.01295 | 0.09 | 0.10 | 0.05 | SNP |
| chr6_32606266_T_C | 32606266 | 1.93 | 1.17 | 3.32 | 0.01313 | 0.09 | 0.10 | 0.06 | SNP |
| chr6_32604395_A_G | 32604395 | 1.92 | 1.17 | 3.31 | 0.01316 | 0.09 | 0.10 | 0.05 | SNP |
| chr6_32604431_A_T | 32604431 | 1.92 | 1.17 | 3.31 | 0.01316 | 0.09 | 0.10 | 0.05 | SNP |
| chr6_32406473_G_A | 32406473 | 1.71 | 1.13 | 2.63 | 0.01316 | 0.23 | 0.23 | 0.21 | SNP |
| chr6_32604924_C_T | 32604924 | 1.94 | 1.17 | 3.36 | 0.01318 | 0.09 | 0.10 | 0.05 | SNP |
| chr6_31322611_C_T | 31322611 | 3.13 | 1.36 | 8.54 | 0.01332 | 0.04 | 0.05 | 0.02 | SNP |
| chr6_31322987_C_T | 31322987 | 3.13 | 1.36 | 8.54 | 0.01332 | 0.04 | 0.05 | 0.02 | SNP |
| chr6_31323455_A_G | 31323455 | 3.13 | 1.36 | 8.54 | 0.01332 | 0.04 | 0.05 | 0.02 | SNP |
| chr6_31378257_G_A | 31378257 | 3.13 | 1.36 | 8.54 | 0.01332 | 0.04 | 0.05 | 0.02 | SNP |
| chr6_32604974_C_T | 32604974 | 1.92 | 1.17 | 3.30 | 0.01342 | 0.09 | 0.10 | 0.05 | SNP |
| chr6_32605689_C_A | 32605689 | 1.92 | 1.17 | 3.30 | 0.01342 | 0.09 | 0.10 | 0.05 | SNP |
| chr6_32604544_C_T | 32604544 | 1.62 | 1.11 | 2.40 | 0.01342 | 0.32 | 0.35 | 0.26 | SNP |
| chr6_32373698_G_T | 32373698 | 0.66 | 0.47 | 0.92 | 0.01343 | 0.43 | 0.40 | 0.46 | SNP |
| chr6_32610330_A_T | 32610330 | 1.91 | 1.16 | 3.29 | 0.01391 | 0.09 | 0.10 | 0.05 | SNP |
| chr6_32636521_G_A | 32636521 | 0.63 | 0.44 | 0.91 | 0.01399 | 0.25 | 0.22 | 0.29 | SNP |
| chr6_33143948_C_G | 33143948 | 0.72 | 0.55 | 0.94 | 0.01431 | 0.26 | 0.24 | 0.31 | SNP |
| chr6_31322690_A_G | 31322690 | 3.10 | 1.34 | 8.47 | 0.01441 | 0.03 | 0.04 | 0.02 | SNP |
| chr6_29910725_C_G | 29910725 | 0.51 | 0.29 | 0.88 | 0.01446 | 0.08 | 0.07 | 0.11 | SNP |
| chr6_31113214_C_A | 31113214 | 0.67 | 0.48 | 0.92 | 0.01448 | 0.25 | 0.23 | 0.30 | SNP |
| chr6_32341398_A_G | 32341398 | 1.76 | 1.13 | 2.80 | 0.01448 | 0.13 | 0.14 | 0.10 | SNP |
| chr6_32612326_C_T | 32612326 | 0.64 | 0.45 | 0.92 | 0.01449 | 0.39 | 0.36 | 0.43 | SNP |
| chr6_32559520_C_T | 32559520 | 0.62 | 0.43 | 0.91 | 0.01460 | 0.39 | 0.37 | 0.44 | SNP |
| chr6_32604644_G_A | 32604644 | 0.62 | 0.42 | 0.91 | 0.01487 | 0.37 | 0.35 | 0.40 | SNP |
| chr6_32364356_A_G | 32364356 | 0.63 | 0.44 | 0.91 | 0.01489 | 0.34 | 0.32 | 0.38 | SNP |
| chr6_32603897_A_G | 32603897 | 1.93 | 1.16 | 3.35 | 0.01490 | 0.09 | 0.10 | 0.05 | SNP |
| chr6_32606779_G_A | 32606779 | 1.90 | 1.16 | 3.28 | 0.01497 | 0.09 | 0.10 | 0.06 | SNP |
| chr6_32612279_T_C | 32612279 | 0.65 | 0.46 | 0.92 | 0.01504 | 0.39 | 0.36 | 0.43 | SNP |
| chr6_32803408_T_A | 32803408 | 3.78 | 1.44 | 13.03 | 0.01522 | 0.03 | 0.04 | 0.01 | SNP |
| chr6_33045500_T_C | 33045500 | 0.43 | 0.22 | 0.85 | 0.01526 | 0.03 | 0.02 | 0.05 | SNP |
| chr6_32341318_A_G | 32341318 | 0.64 | 0.45 | 0.92 | 0.01527 | 0.34 | 0.31 | 0.37 | SNP |
| chr6_32340654_G_A | 32340654 | 0.64 | 0.45 | 0.92 | 0.01531 | 0.34 | 0.31 | 0.37 | SNP |
| chr6_32786882_T_C | 32786882 | 0.57 | 0.36 | 0.90 | 0.01546 | 0.08 | 0.06 | 0.11 | SNP |
| chr6_32373801_T_G | 32373801 | 0.66 | 0.48 | 0.92 | 0.01547 | 0.43 | 0.40 | 0.46 | SNP |
| chr6_33049902_T_A | 33049902 | 0.39 | 0.18 | 0.83 | 0.01565 | 0.03 | 0.02 | 0.04 | SNP |
| chr6_32627480_G_A | 32627480 | 0.65 | 0.45 | 0.92 | 0.01609 | 0.46 | 0.45 | 0.50 | SNP |
| chr6_32411362_A_C | 32411362 | 1.75 | 1.12 | 2.80 | 0.01635 | 0.12 | 0.14 | 0.10 | SNP |
| chr6_32412435_G_A | 32412435 | 1.75 | 1.12 | 2.80 | 0.01635 | 0.12 | 0.14 | 0.10 | SNP |

| | | | | | | | | | |
|---|---|---|---|---|---|---|---|---|---|
| chr6_32609520_C_A | 32609520 | 1.90 | 1.15 | 3.30 | 0.01643 | 0.09 | 0.10 | 0.05 | SNP |
| chr6_31321776_T_C | 31321776 | 3.03 | 1.31 | 8.26 | 0.01646 | 0.03 | 0.04 | 0.02 | SNP |
| chr6_33144243_T_A | 33144243 | 0.72 | 0.55 | 0.94 | 0.01707 | 0.26 | 0.24 | 0.31 | SNP |
| chr6_32188297_G_C | 32188297 | 1.45 | 1.07 | 1.98 | 0.01711 | 0.33 | 0.35 | 0.29 | SNP |
| chr6_31544189_A_G | 31544189 | 2.41 | 1.21 | 5.23 | 0.01722 | 0.05 | 0.06 | 0.03 | SNP |
| chr6_32942302_A_G | 32942302 | 1.40 | 1.06 | 1.85 | 0.01737 | 0.39 | 0.41 | 0.35 | SNP |
| chr6_32610105_A_G | 32610105 | 1.88 | 1.14 | 3.25 | 0.01738 | 0.09 | 0.10 | 0.05 | SNP |
| chr6_33047173_T_C | 33047173 | 0.39 | 0.17 | 0.84 | 0.01758 | 0.03 | 0.02 | 0.04 | SNP |
| chr6_33048354_A_T | 33048354 | 0.39 | 0.17 | 0.84 | 0.01758 | 0.03 | 0.02 | 0.04 | SNP |
| chr6_33049965_G_A | 33049965 | 0.39 | 0.17 | 0.84 | 0.01758 | 0.02 | 0.02 | 0.04 | SNP |
| chr6_32608790_C_T | 32608790 | 2.62 | 1.27 | 6.39 | 0.01770 | 0.04 | 0.05 | 0.02 | SNP |
| chr6_31378864_A_G | 31378864 | 3.00 | 1.29 | 8.21 | 0.01771 | 0.03 | 0.04 | 0.02 | SNP |
| chr6_32611260_A_G | 32611260 | 0.65 | 0.45 | 0.93 | 0.01785 | 0.38 | 0.36 | 0.42 | SNP |
| chr6_31323233_A_G | 31323233 | 1.45 | 1.07 | 1.99 | 0.01788 | 0.40 | 0.42 | 0.35 | SNP |
| chr6_32341473_T_C | 32341473 | 0.65 | 0.46 | 0.93 | 0.01824 | 0.34 | 0.32 | 0.37 | SNP |
| chr6_32363844_C_T | 32363844 | 0.65 | 0.46 | 0.93 | 0.01824 | 0.34 | 0.32 | 0.37 | SNP |
| chr6_32375352_A_C | 32375352 | 0.65 | 0.46 | 0.93 | 0.01880 | 0.35 | 0.33 | 0.38 | SNP |
| chr6_32375371_A_T | 32375371 | 0.65 | 0.46 | 0.93 | 0.01880 | 0.35 | 0.33 | 0.38 | SNP |
| chr6_32375374_G_A | 32375374 | 0.65 | 0.46 | 0.93 | 0.01880 | 0.35 | 0.33 | 0.38 | SNP |
| chr6_32375424_G_A | 32375424 | 0.65 | 0.46 | 0.93 | 0.01880 | 0.35 | 0.33 | 0.38 | SNP |
| chr6_32501245_A_G | 32501245 | 1.55 | 1.09 | 2.28 | 0.01894 | 0.14 | 0.16 | 0.10 | SNP |
| chr6_33095647_G_C | 33095647 | 0.64 | 0.44 | 0.93 | 0.01911 | 0.14 | 0.11 | 0.17 | SNP |
| chr6_29689254_G_C | 29689254 | 0.43 | 0.21 | 0.87 | 0.01940 | 0.03 | 0.02 | 0.05 | SNP |
| chr6_32500957_A_T | 32500957 | 0.52 | 0.30 | 0.90 | 0.01944 | 0.15 | 0.15 | 0.16 | SNP |
| chr6_32609456_C_T | 32609456 | 0.66 | 0.46 | 0.93 | 0.01949 | 0.39 | 0.36 | 0.43 | SNP |
| chr6_32604642_A_G | 32604642 | 0.63 | 0.42 | 0.93 | 0.01954 | 0.37 | 0.35 | 0.40 | SNP |
| chr6_32806584_G_A | 32806584 | 1.49 | 1.07 | 2.10 | 0.01962 | 0.50 | 0.47 | 0.55 | SNP |
| chr6_32376746_C_T | 32376746 | 0.66 | 0.46 | 0.93 | 0.01994 | 0.34 | 0.32 | 0.37 | SNP |
| chr6_30865204_A_C | 30865204 | 1.72 | 1.10 | 2.75 | 0.01999 | 0.12 | 0.14 | 0.08 | SNP |
| chr6_32341719_C_T | 32341719 | 0.66 | 0.46 | 0.93 | 0.02009 | 0.34 | 0.31 | 0.37 | SNP |
| chr6_33142162_C_A | 33142162 | 0.58 | 0.36 | 0.92 | 0.02016 | 0.07 | 0.06 | 0.10 | SNP |
| chr6_29945270_A_G | 29945270 | 0.58 | 0.37 | 0.92 | 0.02060 | 0.08 | 0.07 | 0.11 | SNP |
| chr6_32363816_T_C | 32363816 | 0.69 | 0.50 | 0.94 | 0.02089 | 0.45 | 0.43 | 0.49 | SNP |
| chr6_32375695_G_A | 32375695 | 0.65 | 0.45 | 0.94 | 0.02089 | 0.34 | 0.32 | 0.38 | SNP |
| chr6_32405671_C_A | 32405671 | 1.47 | 1.07 | 2.06 | 0.02093 | 0.32 | 0.34 | 0.30 | SNP |
| chr6_32610994_C_A | 32610994 | 0.65 | 0.45 | 0.94 | 0.02124 | 0.35 | 0.32 | 0.39 | SNP |
| chr6_32610997_C_G | 32610997 | 0.65 | 0.45 | 0.94 | 0.02124 | 0.35 | 0.32 | 0.39 | SNP |
| chr6_32374622_A_T | 32374622 | 0.66 | 0.46 | 0.94 | 0.02125 | 0.34 | 0.32 | 0.37 | SNP |
| chr6_33547430_G_A | 33547430 | 1.57 | 1.08 | 2.33 | 0.02127 | 0.19 | 0.21 | 0.15 | SNP |
| chr6_32409530_A_G | 32409530 | 1.42 | 1.06 | 1.91 | 0.02129 | 0.41 | 0.42 | 0.38 | SNP |
| chr6_32608537_A_C | 32608537 | 0.66 | 0.47 | 0.94 | 0.02147 | 0.39 | 0.37 | 0.43 | SNP |
| chr6_32375973_C_A | 32375973 | 0.64 | 0.43 | 0.93 | 0.02151 | 0.31 | 0.29 | 0.34 | SNP |
| chr6_32610008_C_T | 32610008 | 0.66 | 0.47 | 0.94 | 0.02172 | 0.39 | 0.37 | 0.43 | SNP |
| chr6_32178773_T_C | 32178773 | 1.44 | 1.06 | 1.96 | 0.02177 | 0.49 | 0.52 | 0.43 | SNP |
| chr6_33641897_G_A | 33641897 | 1.85 | 1.11 | 3.22 | 0.02177 | 0.10 | 0.12 | 0.07 | SNP |
| chr6_32943345_T_C | 32943345 | 1.36 | 1.05 | 1.77 | 0.02231 | 0.44 | 0.46 | 0.40 | SNP |
| chr6_32612410_T_C | 32612410 | 1.60 | 1.07 | 2.41 | 0.02262 | 0.36 | 0.37 | 0.33 | SNP |
| chr6_32169621_T_A | 32169621 | 1.40 | 1.05 | 1.88 | 0.02286 | 0.49 | 0.52 | 0.43 | SNP |
| chr6_33050178_C_T | 33050178 | 0.40 | 0.18 | 0.88 | 0.02294 | 0.03 | 0.02 | 0.04 | SNP |
| chr6_31367874_T_C | 31367874 | 2.88 | 1.24 | 7.91 | 0.02295 | 0.04 | 0.05 | 0.02 | SNP |
| xHLA_2field_B_57_01 | 31321652 | 3.14 | 1.27 | 9.50 | 0.02318 | 0.03 | 0.04 | 0.01 | HLA (xHLA) |
| chr6_32170433_C_T | 32170433 | 1.41 | 1.05 | 1.89 | 0.02320 | 0.49 | 0.52 | 0.43 | SNP |
| chr6_31243859_C_T | 31243859 | 0.62 | 0.42 | 0.94 | 0.02325 | 0.22 | 0.22 | 0.23 | SNP |
| chr6_32409781_C_T | 32409781 | 0.67 | 0.47 | 0.94 | 0.02329 | 0.45 | 0.42 | 0.49 | SNP |
| xHLA_2field_C_06_02 | 31236526 | 1.98 | 1.13 | 3.70 | 0.02332 | 0.07 | 0.08 | 0.04 | HLA (xHLA) |
| chr6_32612241_A_T | 32612241 | 0.66 | 0.47 | 0.95 | 0.02348 | 0.39 | 0.36 | 0.43 | SNP |
| chr6_32559216_G_A | 32559216 | 1.47 | 1.06 | 2.07 | 0.02351 | 0.37 | 0.38 | 0.34 | SNP |
| chr6_32608483_T_C | 32608483 | 0.67 | 0.47 | 0.95 | 0.02361 | 0.39 | 0.37 | 0.43 | SNP |
| chr6_32611971_G_C | 32611971 | 0.66 | 0.46 | 0.94 | 0.02361 | 0.38 | 0.35 | 0.41 | SNP |
| chr6_32406704_G_T | 32406704 | 1.64 | 1.08 | 2.55 | 0.02363 | 0.22 | 0.23 | 0.21 | SNP |
| chr6_32611958_C_G | 32611958 | 0.65 | 0.45 | 0.94 | 0.02367 | 0.37 | 0.34 | 0.41 | SNP |
| chr6_32440879_T_C | 32440879 | 1.55 | 1.06 | 2.27 | 0.02369 | 0.34 | 0.35 | 0.33 | SNP |
| chr6_32014456_C_T | 32014456 | 2.10 | 1.14 | 4.20 | 0.02375 | 0.06 | 0.07 | 0.03 | SNP |
| chr6_32085278_C_T | 32085278 | 3.11 | 1.26 | 9.40 | 0.02381 | 0.03 | 0.04 | 0.02 | SNP |
| chr6_32191339_C_T | 32191339 | 2.13 | 1.14 | 4.28 | 0.02382 | 0.05 | 0.07 | 0.03 | SNP |
| chr6_32610934_A_G | 32610934 | 0.65 | 0.44 | 0.94 | 0.02391 | 0.38 | 0.36 | 0.42 | SNP |
| chr6_32809223_C_A | 32809223 | 1.40 | 1.05 | 1.88 | 0.02396 | 0.45 | 0.48 | 0.40 | SNP |
| chr6_32363955_T_C | 32363955 | 0.70 | 0.51 | 0.95 | 0.02399 | 0.45 | 0.43 | 0.49 | SNP |
| chr6_32628189_A_G | 32628189 | 0.65 | 0.45 | 0.94 | 0.02402 | 0.27 | 0.26 | 0.31 | SNP |
| chr6_32171683_T_C | 32171683 | 1.40 | 1.05 | 1.89 | 0.02428 | 0.48 | 0.51 | 0.43 | SNP |
| chr6_32612251_T_G | 32612251 | 0.67 | 0.47 | 0.95 | 0.02432 | 0.39 | 0.36 | 0.42 | SNP |
| chr6_32611964_C_T | 32611964 | 0.66 | 0.46 | 0.95 | 0.02450 | 0.38 | 0.35 | 0.41 | SNP |
| chr6_33590061_C_T | 33590061 | 1.92 | 1.11 | 3.49 | 0.02453 | 0.07 | 0.08 | 0.05 | SNP |
| chr6_32411833_C_T | 32411833 | 0.68 | 0.48 | 0.95 | 0.02459 | 0.46 | 0.43 | 0.49 | SNP |
| chr6_31248995_C_T | 31248995 | 0.45 | 0.22 | 0.90 | 0.02461 | 0.04 | 0.03 | 0.06 | SNP |
| chr6_31236467_G_T | 31236467 | 1.39 | 1.04 | 1.85 | 0.02465 | 0.34 | 0.37 | 0.30 | SNP |
| chr6_32603177_A_G | 32603177 | 1.58 | 1.07 | 2.38 | 0.02471 | 0.17 | 0.19 | 0.14 | SNP |
| chr6_32781076_C_A | 32781076 | 1.99 | 1.13 | 3.79 | 0.02472 | 0.06 | 0.07 | 0.04 | SNP |
| chr6_32376176_C_T | 32376176 | 0.66 | 0.46 | 0.95 | 0.02475 | 0.35 | 0.32 | 0.38 | SNP |
| chr6_32376348_G_A | 32376348 | 0.65 | 0.44 | 0.94 | 0.02491 | 0.31 | 0.29 | 0.34 | SNP |
| chr6_33377506_G_C | 33377506 | 0.46 | 0.24 | 0.91 | 0.02493 | 0.04 | 0.03 | 0.05 | SNP |
| chr6_32409787_T_C | 32409787 | 0.67 | 0.47 | 0.95 | 0.02496 | 0.45 | 0.42 | 0.48 | SNP |
| chr6_32520460_C_T | 32520460 | 0.67 | 0.48 | 0.95 | 0.02496 | 0.41 | 0.39 | 0.45 | SNP |
| chr6_32606942_G_A | 32606942 | 0.67 | 0.47 | 0.95 | 0.02497 | 0.40 | 0.37 | 0.43 | SNP |
| chr6_32441150_C_T | 32441150 | 0.67 | 0.48 | 0.95 | 0.02512 | 0.40 | 0.37 | 0.43 | SNP |
| chr6_32605646_T_G | 32605646 | 0.67 | 0.47 | 0.95 | 0.02513 | 0.40 | 0.37 | 0.43 | SNP |
| chr6_31473730_G_A | 31473730 | 3.03 | 1.25 | 9.08 | 0.02517 | 0.03 | 0.04 | 0.01 | SNP |
| chr6_32188383_T_C | 32188383 | 1.42 | 1.05 | 1.95 | 0.02519 | 0.45 | 0.48 | 0.40 | SNP |
| chr6_32608856_T_C | 32608856 | 0.67 | 0.47 | 0.95 | 0.02570 | 0.37 | 0.35 | 0.41 | SNP |
| chr6_33048975_G_A | 33048975 | 0.40 | 0.17 | 0.89 | 0.02586 | 0.02 | 0.02 | 0.04 | SNP |
| chr6_29716169_G_T | 29716169 | 0.40 | 0.17 | 0.89 | 0.02592 | 0.03 | 0.02 | 0.05 | SNP |
| chr6_32611243_C_T | 32611243 | 0.66 | 0.46 | 0.95 | 0.02608 | 0.38 | 0.36 | 0.42 | SNP |
| chr6_32605501_C_G | 32605501 | 0.67 | 0.48 | 0.95 | 0.02615 | 0.43 | 0.41 | 0.47 | SNP |
| chr6_32375280_C_G | 32375280 | 0.67 | 0.47 | 0.95 | 0.02641 | 0.35 | 0.32 | 0.38 | SNP |
| chr6_31430951_C_T | 31430951 | 0.54 | 0.31 | 0.93 | 0.02645 | 0.07 | 0.06 | 0.09 | SNP |
| chr6_32550819_C_A | 32550819 | 0.67 | 0.47 | 0.95 | 0.02647 | 0.38 | 0.36 | 0.42 | SNP |
| chr6_32192331_G_A | 32192331 | 0.71 | 0.53 | 0.96 | 0.02753 | 0.48 | 0.51 | 0.42 | SNP |
| chr6_32610403_C_A | 32610403 | 0.67 | 0.47 | 0.96 | 0.02754 | 0.39 | 0.36 | 0.42 | SNP |
| chr6_32943151_C_T | 32943151 | 1.35 | 1.04 | 1.77 | 0.02758 | 0.39 | 0.41 | 0.36 | SNP |
| chr6_31324750_G_A | 31324750 | 0.56 | 0.33 | 0.94 | 0.02772 | 0.08 | 0.07 | 0.12 | SNP |
| chr6_32806576_C_T | 32806576 | 1.45 | 1.04 | 2.02 | 0.02798 | 0.47 | 0.49 | 0.42 | SNP |
| chr6_32604259_C_T | 32604259 | 0.67 | 0.47 | 0.96 | 0.02802 | 0.39 | 0.37 | 0.43 | SNP |
| chr6_31320687_C_A | 31320687 | 0.57 | 0.34 | 0.94 | 0.02826 | 0.07 | 0.06 | 0.10 | SNP |
| chr6_32375330_G_A | 32375330 | 0.67 | 0.47 | 0.96 | 0.02856 | 0.35 | 0.33 | 0.38 | SNP |
| chr6_32612008_T_C | 32612008 | 0.67 | 0.47 | 0.96 | 0.02856 | 0.37 | 0.35 | 0.41 | SNP |
| chr6_32605336_G_A | 32605336 | 0.68 | 0.48 | 0.96 | 0.02896 | 0.40 | 0.37 | 0.43 | SNP |
| chr6_30876996_G_C | 30876996 | 1.54 | 1.05 | 2.29 | 0.02933 | 0.15 | 0.16 | 0.11 | SNP |
| xHLA_2field_DPB1_01_01 | 33043767 | 0.41 | 0.18 | 0.91 | 0.02940 | 0.02 | 0.02 | 0.04 | HLA (xHLA) |
| chr6_32945097_C_T | 32945097 | 0.42 | 0.19 | 0.92 | 0.02969 | 0.03 | 0.02 | 0.04 | SNP |
| chr6_33384473_C_T | 33384473 | 1.73 | 1.07 | 2.89 | 0.02977 | 0.10 | 0.11 | 0.07 | SNP |
| chr6_30849822_G_A | 30849822 | 1.58 | 1.06 | 2.41 | 0.02985 | 0.12 | 0.14 | 0.09 | SNP |
| chr6_32821447_C_T | 32821447 | 1.65 | 1.06 | 2.64 | 0.03016 | 0.17 | 0.19 | 0.14 | SNP |
| chr6_30920957_G_A | 30920957 | 1.54 | 1.05 | 2.32 | 0.03018 | 0.14 | 0.16 | 0.11 | SNP |
| chr6_32629500_C_A | 32629500 | 0.68 | 0.48 | 0.96 | 0.03060 | 0.27 | 0.25 | 0.32 | SNP |
| chr6_33053577_G_A | 33053577 | 0.40 | 0.17 | 0.92 | 0.03089 | 0.02 | 0.02 | 0.03 | SNP |
| chr6_32608473_G_A | 32608473 | 0.68 | 0.48 | 0.96 | 0.03103 | 0.39 | 0.37 | 0.43 | SNP |
| chr6_30867958_T_C | 30867958 | 1.58 | 1.05 | 2.42 | 0.03103 | 0.12 | 0.14 | 0.09 | SNP |
| chr6_29691800_C_G | 29691800 | 0.42 | 0.19 | 0.92 | 0.03107 | 0.03 | 0.02 | 0.05 | SNP |
| chr6_30877760_A_C | 30877760 | 1.55 | 1.05 | 2.34 | 0.03144 | 0.14 | 0.16 | 0.11 | SNP |
| chr6_32441008_C_T | 32441008 | 0.69 | 0.48 | 0.97 | 0.03220 | 0.40 | 0.37 | 0.43 | SNP |
| chr6_32191607_C_T | 32191607 | 1.74 | 1.07 | 2.96 | 0.03229 | 0.09 | 0.11 | 0.07 | SNP |
| chr6_33141161_G_A | 33141161 | 1.32 | 1.03 | 1.71 | 0.03233 | 0.45 | 0.48 | 0.42 | SNP |
| chr6_31929808_C_T | 31929808 | 2.70 | 1.16 | 7.39 | 0.03246 | 0.03 | 0.04 | 0.02 | SNP |
| chr6_32604394_T_C | 32604394 | 0.68 | 0.48 | 0.97 | 0.03251 | 0.40 | 0.37 | 0.43 | SNP |
| chr6_32826233_A_G | 32826233 | 0.40 | 0.17 | 0.94 | 0.03304 | 0.02 | 0.02 | 0.03 | SNP |
| chr6_30865725_C_T | 30865725 | 1.59 | 1.05 | 2.46 | 0.03307 | 0.13 | 0.15 | 0.10 | SNP |
| chr6_31367865_G_A | 31367865 | 1.50 | 1.04 | 2.18 | 0.03308 | 0.38 | 0.40 | 0.33 | SNP |
| chr6_32410716_G_A | 32410716 | 0.69 | 0.49 | 0.97 | 0.03309 | 0.45 | 0.43 | 0.49 | SNP |

| | | | | | | | | | |
|---|---|---|---|---|---|---|---|---|---|
| chr6_31235869_T_C | 31235869 | 1.62 | 1.05 | 2.57 | 0.03325 | 0.23 | 0.26 | 0.19 | SNP |
| chr6_32551638_C_T | 32551638 | 2.01 | 1.09 | 3.98 | 0.03334 | 0.06 | 0.07 | 0.04 | SNP |
| chr6_31240041_T_C | 31240041 | 0.67 | 0.47 | 0.97 | 0.03344 | 0.42 | 0.41 | 0.45 | SNP |
| chr6_31977391_T_C | 31977391 | 1.70 | 1.05 | 2.82 | 0.03345 | 0.23 | 0.25 | 0.21 | SNP |
| chr6_30864927_C_T | 30864927 | 2.03 | 1.09 | 4.07 | 0.03358 | 0.05 | 0.06 | 0.03 | SNP |
| chr6_32606732_G_A | 32606732 | 0.69 | 0.49 | 0.97 | 0.03391 | 0.40 | 0.38 | 0.43 | SNP |
| chr6_32606052_T_G | 32606052 | 0.69 | 0.48 | 0.97 | 0.03418 | 0.40 | 0.38 | 0.44 | SNP |
| chr6_32605724_A_G | 32605724 | 0.69 | 0.49 | 0.97 | 0.03434 | 0.39 | 0.37 | 0.42 | SNP |
| chr6_31241370_T_C | 31241370 | 1.42 | 1.03 | 1.98 | 0.03440 | 0.36 | 0.37 | 0.34 | SNP |
| chr6_31543101_G_A | 31543101 | 2.38 | 1.12 | 5.71 | 0.03455 | 0.04 | 0.05 | 0.02 | SNP |
| chr6_32611234_C_T | 32611234 | 0.68 | 0.47 | 0.97 | 0.03484 | 0.38 | 0.36 | 0.41 | SNP |
| chr6_32184066_G_T | 32184066 | 1.38 | 1.02 | 1.86 | 0.03496 | 0.45 | 0.48 | 0.40 | SNP |
| chr6_33137570_C_T | 33137570 | 0.43 | 0.19 | 0.94 | 0.03502 | 0.02 | 0.02 | 0.04 | SNP |
| chr6_33158898_C_T | 33158898 | 0.43 | 0.19 | 0.94 | 0.03502 | 0.02 | 0.02 | 0.04 | SNP |
| chr6_32805049_T_C | 32805049 | 1.37 | 1.02 | 1.84 | 0.03547 | 0.47 | 0.49 | 0.42 | SNP |
| chr6_33138021_G_A | 33138021 | 0.43 | 0.19 | 0.95 | 0.03558 | 0.02 | 0.02 | 0.04 | SNP |
| chr6_32551541_C_T | 32551541 | 1.64 | 1.04 | 2.61 | 0.03563 | 0.21 | 0.22 | 0.20 | SNP |
| chr6_32166380_C_G | 32166380 | 2.24 | 1.10 | 5.05 | 0.03567 | 0.04 | 0.05 | 0.02 | SNP |
| chr6_32171279_C_T | 32171279 | 2.24 | 1.10 | 5.05 | 0.03567 | 0.04 | 0.05 | 0.02 | SNP |
| chr6_32171288_C_T | 32171288 | 2.24 | 1.10 | 5.05 | 0.03567 | 0.04 | 0.05 | 0.02 | SNP |
| chr6_32802807_C_T | 32802807 | 0.65 | 0.43 | 0.97 | 0.03581 | 0.11 | 0.10 | 0.14 | SNP |
| chr6_32633811_C_T | 32633811 | 2.05 | 1.09 | 4.21 | 0.03586 | 0.05 | 0.06 | 0.03 | SNP |
| chr6_32604108_T_C | 32604108 | 0.69 | 0.49 | 0.98 | 0.03604 | 0.40 | 0.37 | 0.43 | SNP |
| chr6_30866342_C_G | 30866342 | 1.60 | 1.04 | 2.52 | 0.03607 | 0.14 | 0.16 | 0.10 | SNP |
| chr6_31239449_C_G | 31239449 | 0.53 | 0.30 | 0.96 | 0.03611 | 0.17 | 0.17 | 0.19 | SNP |
| chr6_32558183_G_T | 32558183 | 0.71 | 0.51 | 0.98 | 0.03641 | 0.45 | 0.48 | 0.39 | SNP |
| chr6_29910947_T_A | 29910947 | 0.58 | 0.35 | 0.97 | 0.03643 | 0.08 | 0.07 | 0.10 | SNP |
| chr6_31243884_G_A | 31243884 | 0.65 | 0.44 | 0.98 | 0.03658 | 0.22 | 0.22 | 0.23 | SNP |
| chr6_32945466_A_G | 32945466 | 0.50 | 0.26 | 0.96 | 0.03690 | 0.08 | 0.07 | 0.08 | SNP |
| chr6_32610968_A_G | 32610968 | 0.68 | 0.47 | 0.98 | 0.03698 | 0.35 | 0.33 | 0.39 | SNP |
| chr6_32163274_G_A | 32163274 | 2.23 | 1.10 | 5.02 | 0.03713 | 0.04 | 0.05 | 0.02 | SNP |
| chr6_32559212_A_G | 32559212 | 0.69 | 0.48 | 0.98 | 0.03743 | 0.38 | 0.36 | 0.42 | SNP |
| chr6_31322996_C_T | 31322996 | 1.37 | 1.02 | 1.85 | 0.03751 | 0.38 | 0.40 | 0.34 | SNP |
| chr6_30889389_T_C | 30889389 | 1.56 | 1.04 | 2.39 | 0.03760 | 0.13 | 0.15 | 0.10 | SNP |
| chr6_32611216_A_G | 32611216 | 1.58 | 1.04 | 2.46 | 0.03761 | 0.11 | 0.13 | 0.08 | SNP |
| chr6_30859355_G_A | 30859355 | 1.55 | 1.04 | 2.37 | 0.03766 | 0.13 | 0.14 | 0.09 | SNP |
| chr6_30887972_C_T | 30887972 | 1.52 | 1.03 | 2.28 | 0.03773 | 0.14 | 0.15 | 0.11 | SNP |
| chr6_32635248_A_T | 32635248 | 0.69 | 0.49 | 0.98 | 0.03784 | 0.31 | 0.29 | 0.35 | SNP |
| chr6_31894626_A_G | 31894626 | 0.71 | 0.51 | 0.98 | 0.03798 | 0.42 | 0.41 | 0.46 | SNP |
| chr6_30877202_T_C | 30877202 | 1.51 | 1.03 | 2.26 | 0.03815 | 0.15 | 0.16 | 0.11 | SNP |
| chr6_31321919_G_T | 31321919 | 2.14 | 1.08 | 4.63 | 0.03832 | 0.05 | 0.06 | 0.03 | SNP |
| chr6_32604538_G_A | 32604538 | 0.68 | 0.47 | 0.98 | 0.03858 | 0.43 | 0.41 | 0.46 | SNP |
| chr6_32405821_T_C | 32405821 | 0.67 | 0.46 | 0.98 | 0.03874 | 0.31 | 0.29 | 0.34 | SNP |
| chr6_32191581_T_A | 32191581 | 1.49 | 1.03 | 2.19 | 0.03880 | 0.18 | 0.19 | 0.14 | SNP |
| chr6_31248114_C_T | 31248114 | 0.48 | 0.23 | 0.97 | 0.03914 | 0.03 | 0.02 | 0.05 | SNP |
| chr6_31127562_C_T | 31127562 | 1.67 | 1.04 | 2.75 | 0.03917 | 0.21 | 0.23 | 0.16 | SNP |
| chr6_32191730_G_A | 32191730 | 1.41 | 1.02 | 1.98 | 0.03927 | 0.25 | 0.27 | 0.22 | SNP |
| chr6_32804570_G_A | 32804570 | 1.39 | 1.02 | 1.91 | 0.03990 | 0.44 | 0.46 | 0.40 | SNP |
| chr6_32612041_A_G | 32612041 | 0.69 | 0.48 | 0.98 | 0.04007 | 0.38 | 0.36 | 0.42 | SNP |
| chr6_32611760_G_T | 32611760 | 0.69 | 0.48 | 0.98 | 0.04016 | 0.38 | 0.36 | 0.42 | SNP |
| chr6_32193220_A_G | 32193220 | 1.38 | 1.02 | 1.88 | 0.04022 | 0.28 | 0.30 | 0.25 | SNP |
| chr6_32193698_A_T | 32193698 | 1.38 | 1.02 | 1.88 | 0.04022 | 0.28 | 0.30 | 0.25 | SNP |
| chr6_32604059_A_G | 32604059 | 0.69 | 0.49 | 0.98 | 0.04033 | 0.39 | 0.37 | 0.43 | SNP |
| chr6_32781121_G_C | 32781121 | 1.86 | 1.06 | 3.47 | 0.04040 | 0.06 | 0.07 | 0.04 | SNP |
| chr6_31430060_G_T | 31430060 | 2.46 | 1.10 | 6.28 | 0.04047 | 0.03 | 0.04 | 0.02 | SNP |
| chr6_32605101_A_G | 32605101 | 0.69 | 0.49 | 0.98 | 0.04049 | 0.40 | 0.37 | 0.43 | SNP |
| chr6_32973281_C_T | 32973281 | 1.31 | 1.01 | 1.70 | 0.04050 | 0.49 | 0.51 | 0.45 | SNP |
| chr6_32608467_G_A | 32608467 | 0.69 | 0.49 | 0.98 | 0.04058 | 0.39 | 0.37 | 0.43 | SNP |
| chr6_31379752_C_T | 31379752 | 0.58 | 0.34 | 0.98 | 0.04076 | 0.06 | 0.06 | 0.08 | SNP |
| chr6_32609358_C_T | 32609358 | 0.69 | 0.48 | 0.98 | 0.04106 | 0.37 | 0.35 | 0.40 | SNP |
| chr6_32303672_T_A | 32303672 | 1.82 | 1.05 | 3.35 | 0.04119 | 0.06 | 0.07 | 0.04 | SNP |
| chr6_32633971_A_G | 32633971 | 0.70 | 0.49 | 0.99 | 0.04129 | 0.27 | 0.25 | 0.31 | SNP |
| chr6_32409305_A_G | 32409305 | 0.71 | 0.50 | 0.99 | 0.04148 | 0.45 | 0.43 | 0.49 | SNP |
| chr6_32411523_T_C | 32411523 | 0.71 | 0.50 | 0.99 | 0.04148 | 0.45 | 0.43 | 0.49 | SNP |
| chr6_29893139_G_A | 29893139 | 0.68 | 0.47 | 0.99 | 0.04151 | 0.23 | 0.22 | 0.24 | SNP |
| chr6_32338283_G_T | 32338283 | 1.50 | 1.02 | 2.24 | 0.04153 | 0.26 | 0.29 | 0.21 | SNP |
| chr6_31325090_T_C | 31325090 | 1.54 | 1.02 | 2.34 | 0.04155 | 0.40 | 0.41 | 0.37 | SNP |
| chr6_32612104_C_T | 32612104 | 0.69 | 0.48 | 0.99 | 0.04167 | 0.39 | 0.37 | 0.42 | SNP |
| chr6_32612111_G_A | 32612111 | 0.69 | 0.48 | 0.99 | 0.04167 | 0.39 | 0.37 | 0.42 | SNP |
| chr6_29893919_G_C | 29893919 | 0.56 | 0.32 | 0.98 | 0.04171 | 0.05 | 0.04 | 0.07 | SNP |
| chr6_32606217_G_A | 32606217 | 0.69 | 0.49 | 0.99 | 0.04188 | 0.40 | 0.37 | 0.43 | SNP |
| chr6_32608417_G_A | 32608417 | 0.69 | 0.49 | 0.99 | 0.04232 | 0.39 | 0.37 | 0.42 | SNP |
| chr6_32797466_G_A | 32797466 | 1.74 | 1.03 | 3.01 | 0.04234 | 0.11 | 0.12 | 0.08 | SNP |
| chr6_32610860_G_T | 32610860 | 0.69 | 0.48 | 0.99 | 0.04250 | 0.38 | 0.36 | 0.41 | SNP |
| chr6_32604151_C_T | 32604151 | 0.70 | 0.49 | 0.99 | 0.04292 | 0.39 | 0.37 | 0.43 | SNP |
| chr6_31321364_G_A | 31321364 | 0.60 | 0.36 | 0.99 | 0.04308 | 0.07 | 0.06 | 0.09 | SNP |
| chr6_31125978_A_T | 31125978 | 1.78 | 1.03 | 3.16 | 0.04319 | 0.22 | 0.24 | 0.17 | SNP |
| chr6_31240635_G_C | 31240635 | 0.65 | 0.42 | 0.99 | 0.04326 | 0.12 | 0.11 | 0.14 | SNP |
| chr6_33627367_A_G | 33627367 | 0.50 | 0.25 | 0.99 | 0.04329 | 0.04 | 0.03 | 0.06 | SNP |
| chr6_32407906_T_C | 32407906 | 2.06 | 1.07 | 4.40 | 0.04336 | 0.05 | 0.06 | 0.03 | SNP |
| chr6_29941851_G_T | 29941851 | 0.53 | 0.29 | 0.98 | 0.04342 | 0.04 | 0.03 | 0.06 | SNP |
| chr6_31322367_T_G | 31322367 | 0.61 | 0.38 | 0.99 | 0.04346 | 0.07 | 0.06 | 0.09 | SNP |
| chr6_32793981_C_A | 32793981 | 0.71 | 0.51 | 0.99 | 0.04398 | 0.25 | 0.23 | 0.30 | SNP |
| chr6_32780914_G_A | 32780914 | 1.83 | 1.05 | 3.40 | 0.04402 | 0.06 | 0.07 | 0.04 | SNP |
| chr6_32559708_G_A | 32559708 | 0.69 | 0.48 | 0.99 | 0.04407 | 0.38 | 0.36 | 0.42 | SNP |
| chr6_32635230_G_A | 32635230 | 0.70 | 0.50 | 0.99 | 0.04423 | 0.31 | 0.30 | 0.36 | SNP |
| chr6_31322402_C_T | 31322402 | 0.73 | 0.54 | 0.99 | 0.04437 | 0.34 | 0.33 | 0.35 | SNP |
| chr6_29910473_G_A | 29910473 | 0.60 | 0.36 | 0.99 | 0.04437 | 0.08 | 0.07 | 0.11 | SNP |
| chr6_30867666_T_C | 30867666 | 1.48 | 1.02 | 2.20 | 0.04446 | 0.15 | 0.16 | 0.11 | SNP |
| chr6_32611265_C_T | 32611265 | 0.69 | 0.48 | 0.99 | 0.04448 | 0.38 | 0.36 | 0.41 | SNP |
| chr6_29893960_T_C | 29893960 | 0.68 | 0.47 | 0.99 | 0.04453 | 0.22 | 0.21 | 0.24 | SNP |
| chr6_30857072_G_C | 30857072 | 1.52 | 1.02 | 2.32 | 0.04454 | 0.14 | 0.16 | 0.11 | SNP |
| chr6_30852747_C_T | 30852747 | 1.51 | 1.02 | 2.28 | 0.04457 | 0.14 | 0.16 | 0.11 | SNP |
| chr6_29759222_G_A | 29759222 | 0.46 | 0.21 | 0.98 | 0.04486 | 0.02 | 0.02 | 0.04 | SNP |
| chr6_32184094_G_A | 32184094 | 1.35 | 1.01 | 1.82 | 0.04514 | 0.46 | 0.49 | 0.41 | SNP |
| chr6_32605418_G_A | 32605418 | 0.70 | 0.49 | 0.99 | 0.04521 | 0.39 | 0.37 | 0.43 | SNP |
| chr6_32782387_G_A | 32782387 | 0.72 | 0.52 | 0.99 | 0.04523 | 0.23 | 0.22 | 0.26 | SNP |
| chr6_32803999_A_T | 32803999 | 0.46 | 0.21 | 0.98 | 0.04560 | 0.02 | 0.02 | 0.04 | SNP |
| chr6_33257162_C_T | 33257162 | 1.51 | 1.02 | 2.30 | 0.04570 | 0.14 | 0.16 | 0.12 | SNP |
| chr6_33382288_A_G | 33382288 | 1.58 | 1.02 | 2.50 | 0.04592 | 0.11 | 0.12 | 0.08 | SNP |
| chr6_32798647_A_T | 32798647 | 0.46 | 0.21 | 0.98 | 0.04642 | 0.02 | 0.02 | 0.04 | SNP |
| chr6_31322855_C_T | 31322855 | 0.61 | 0.38 | 1.00 | 0.04663 | 0.07 | 0.06 | 0.09 | SNP |
| chr6_29911069_A_T | 29911069 | 0.61 | 0.37 | 1.00 | 0.04670 | 0.08 | 0.07 | 0.10 | SNP |
| chr6_32370616_T_C | 32370616 | 0.69 | 0.48 | 0.99 | 0.04685 | 0.34 | 0.32 | 0.37 | SNP |
| chr6_30865115_G_A | 30865115 | 1.67 | 1.02 | 2.82 | 0.04706 | 0.14 | 0.16 | 0.10 | SNP |
| chr6_32604564_C_T | 32604564 | 0.69 | 0.48 | 0.99 | 0.04726 | 0.43 | 0.41 | 0.46 | SNP |
| chr6_32187605_G_A | 32187605 | 0.72 | 0.52 | 1.00 | 0.04736 | 0.45 | 0.43 | 0.51 | SNP |
| chr6_33160906_G_T | 33160906 | 0.39 | 0.15 | 0.99 | 0.04758 | 0.02 | 0.01 | 0.03 | SNP |
| chr6_32184221_G_A | 32184221 | 1.35 | 1.00 | 1.81 | 0.04782 | 0.46 | 0.49 | 0.41 | SNP |
| chr6_31930462_G_A | 31930462 | 1.60 | 1.02 | 2.59 | 0.04803 | 0.10 | 0.11 | 0.08 | SNP |
| chr6_33132931_T_C | 33132931 | 1.35 | 1.00 | 1.81 | 0.04804 | 0.48 | 0.50 | 0.43 | SNP |
| chr6_32297209_G_A | 32297209 | 1.83 | 1.03 | 3.46 | 0.04811 | 0.06 | 0.07 | 0.04 | SNP |
| chr6_31327034_C_A | 31327034 | 0.59 | 0.34 | 1.00 | 0.04828 | 0.06 | 0.05 | 0.08 | SNP |
| chr6_32629066_T_C | 32629066 | 0.71 | 0.51 | 1.00 | 0.04831 | 0.32 | 0.30 | 0.36 | SNP |
| chr6_29896967_C_T | 29896967 | 0.68 | 0.47 | 1.00 | 0.04832 | 0.20 | 0.19 | 0.22 | SNP |
| chr6_32192978_T_A | 32192978 | 1.36 | 1.01 | 1.85 | 0.04860 | 0.27 | 0.28 | 0.23 | SNP |
| chr6_33134102_C_T | 33134102 | 1.29 | 1.00 | 1.65 | 0.04870 | 0.47 | 0.50 | 0.43 | SNP |
| chr6_30878769_C_T | 30878769 | 1.48 | 1.01 | 2.20 | 0.04888 | 0.15 | 0.16 | 0.11 | SNP |
| chr6_29907740_A_G | 29907740 | 1.37 | 1.00 | 1.88 | 0.04901 | 0.47 | 0.48 | 0.44 | SNP |
| chr6_31322129_C_G | 31322129 | 0.62 | 0.38 | 1.00 | 0.04924 | 0.07 | 0.06 | 0.09 | SNP |
| chr6_32608809_T_C | 32608809 | 0.69 | 0.48 | 1.00 | 0.04945 | 0.36 | 0.34 | 0.39 | SNP |
| chr6_32604124_A_G | 32604124 | 1.41 | 1.00 | 2.00 | 0.04965 | 0.42 | 0.43 | 0.41 | SNP |
| chr6_32627446_C_T | 32627446 | 2.01 | 1.04 | 4.29 | 0.04978 | 0.05 | 0.06 | 0.03 | SNP |
| chr6_30877054_G_A | 30877054 | 1.49 | 1.01 | 2.26 | 0.04981 | 0.13 | 0.15 | 0.11 | SNP |

ANCA = anitneutrophil cytoplasmic antibody; AAV = ANCA-associated vasculitis; SNP = single nucleotide polymorphism;
OR = Odds ratio; CI = confidence interval; MAF = minor allele frequency. SNPs and HLA alleles with P < 0.05 included in the table.

**Supplemtary Table 8**. Association analysis of renal involvement in all AAV patients versus controls, adjusting for sex and genetic structure (principal components 1-4).

| Variant | position | OR | CI_lower | CI_upper | p | MAF | MAF_cases | MAF_controls | Type (SNP, HLA (xHLA)) |
|---|---|---|---|---|---|---|---|---|---|
| xHLA_2field_DRB1_04_04 | 32546552 | 4.31 | 2.37 | 8.56 | 6.96E-06 | 0.09 | 0.12 | 0.04 | HLA (xHLA) |
| chr6_31324531_T_C | 31324531 | 0.44 | 0.29 | 0.66 | 7.64E-05 | 0.33 | 0.30 | 0.38 | SNP |
| chr6_29892107_C_T | 29892107 | 2.08 | 1.41 | 3.13 | 0.00030 | 0.23 | 0.25 | 0.18 | SNP |
| chr6_31244961_G_A | 31244961 | 4.29 | 2.03 | 10.34 | 0.00040 | 0.06 | 0.08 | 0.03 | SNP |
| chr6_31235775_T_C | 31235775 | 3.00 | 1.64 | 5.88 | 0.00070 | 0.08 | 0.09 | 0.04 | SNP |
| chr6_32796310_A_G | 32796310 | 6.08 | 2.38 | 20.68 | 0.00080 | 0.04 | 0.05 | 0.01 | SNP |
| chr6_31113052_A_G | 31113052 | 0.54 | 0.38 | 0.78 | 0.00086 | 0.31 | 0.29 | 0.34 | SNP |
| chr6_32376517_C_T | 32376517 | 2.46 | 1.47 | 4.27 | 0.00088 | 0.09 | 0.11 | 0.06 | SNP |
| chr6_31320687_C_A | 31320687 | 2.90 | 1.59 | 5.68 | 0.00096 | 0.07 | 0.09 | 0.04 | SNP |
| chr6_32369488_G_A | 32369488 | 2.36 | 1.43 | 4.06 | 0.00122 | 0.10 | 0.11 | 0.06 | SNP |
| chr6_29893922_A_T | 29893922 | 2.00 | 1.33 | 3.10 | 0.00123 | 0.22 | 0.24 | 0.19 | SNP |
| chr6_31247108_G_A | 31247108 | 3.36 | 1.68 | 7.42 | 0.00127 | 0.07 | 0.08 | 0.03 | SNP |
| chr6_33032851_T_C | 33032851 | 2.68 | 1.51 | 5.09 | 0.00134 | 0.08 | 0.09 | 0.04 | SNP |
| chr6_33037199_G_C | 33037199 | 2.68 | 1.51 | 5.09 | 0.00134 | 0.08 | 0.09 | 0.04 | SNP |
| chr6_33033274_T_C | 33033274 | 2.66 | 1.50 | 5.05 | 0.00148 | 0.08 | 0.09 | 0.04 | SNP |
| chr6_29892137_A_G | 29892137 | 1.84 | 1.27 | 2.71 | 0.00151 | 0.26 | 0.28 | 0.22 | SNP |
| chr6_29896105_G_A | 29896105 | 1.87 | 1.28 | 2.78 | 0.00153 | 0.24 | 0.26 | 0.20 | SNP |
| chr6_31246015_C_T | 31246015 | 2.96 | 1.56 | 6.08 | 0.00164 | 0.07 | 0.08 | 0.03 | SNP |
| chr6_31246542_G_T | 31246542 | 2.96 | 1.56 | 6.09 | 0.00170 | 0.07 | 0.08 | 0.03 | SNP |
| chr6_29893960_T_C | 29893960 | 1.95 | 1.30 | 3.00 | 0.00181 | 0.22 | 0.24 | 0.19 | SNP |
| chr6_32628195_A_G | 32628195 | 3.61 | 1.73 | 8.91 | 0.00185 | 0.05 | 0.07 | 0.02 | SNP |
| chr6_31246668_A_G | 31246668 | 3.06 | 1.57 | 6.49 | 0.00188 | 0.06 | 0.08 | 0.03 | SNP |
| chr6_31238538_G_C | 31238538 | 2.82 | 1.51 | 5.66 | 0.00196 | 0.07 | 0.08 | 0.04 | SNP |
| chr6_31241032_A_G | 31241032 | 2.80 | 1.50 | 5.62 | 0.00210 | 0.07 | 0.08 | 0.04 | SNP |
| chr6_31244298_A_G | 31244298 | 2.80 | 1.50 | 5.63 | 0.00218 | 0.07 | 0.08 | 0.04 | SNP |
| chr6_29892203_C_A | 29892203 | 1.81 | 1.25 | 2.67 | 0.00221 | 0.26 | 0.28 | 0.23 | SNP |
| chr6_29895701_T_C | 29895701 | 1.81 | 1.25 | 2.67 | 0.00232 | 0.23 | 0.25 | 0.19 | SNP |
| chr6_33041734_T_C | 33041734 | 2.56 | 1.44 | 4.86 | 0.00232 | 0.08 | 0.09 | 0.04 | SNP |
| chr6_31245309_C_A | 31245309 | 2.77 | 1.48 | 5.56 | 0.00236 | 0.07 | 0.08 | 0.04 | SNP |
| chr6_31237779_C_T | 31237779 | 2.83 | 1.49 | 5.75 | 0.00238 | 0.07 | 0.08 | 0.04 | SNP |
| chr6_31324599_T_G | 31324599 | 0.51 | 0.33 | 0.79 | 0.00252 | 0.16 | 0.14 | 0.22 | SNP |
| chr6_31246100_A_G | 31246100 | 2.75 | 1.47 | 5.52 | 0.00253 | 0.07 | 0.08 | 0.04 | SNP |
| chr6_31235598_G_C | 31235598 | 0.61 | 0.44 | 0.84 | 0.00256 | 0.22 | 0.20 | 0.27 | SNP |
| chr6_31246253_T_C | 31246253 | 2.74 | 1.47 | 5.51 | 0.00256 | 0.07 | 0.08 | 0.04 | SNP |
| chr6_31241793_G_A | 31241793 | 2.76 | 1.47 | 5.57 | 0.00257 | 0.07 | 0.08 | 0.04 | SNP |
| chr6_31244151_T_C | 31244151 | 2.75 | 1.47 | 5.52 | 0.00257 | 0.07 | 0.08 | 0.04 | SNP |
| chr6_31245180_T_C | 31245180 | 2.76 | 1.47 | 5.57 | 0.00259 | 0.07 | 0.08 | 0.04 | SNP |
| chr6_31245182_A_G | 31245182 | 2.76 | 1.47 | 5.57 | 0.00259 | 0.07 | 0.08 | 0.04 | SNP |
| chr6_31245476_C_A | 31245476 | 2.73 | 1.46 | 5.49 | 0.00266 | 0.07 | 0.08 | 0.04 | SNP |
| chr6_31246095_T_C | 31246095 | 2.73 | 1.46 | 5.47 | 0.00273 | 0.07 | 0.08 | 0.04 | SNP |
| chr6_31246676_C_T | 31246676 | 2.83 | 1.48 | 5.85 | 0.00289 | 0.07 | 0.08 | 0.03 | SNP |
| chr6_31246119_T_C | 31246119 | 2.70 | 1.45 | 5.44 | 0.00303 | 0.07 | 0.08 | 0.04 | SNP |
| chr6_31320097_C_A | 31320097 | 3.22 | 1.56 | 7.44 | 0.00305 | 0.06 | 0.07 | 0.03 | SNP |
| chr6_31246177_G_A | 31246177 | 2.70 | 1.44 | 5.43 | 0.00306 | 0.07 | 0.08 | 0.04 | SNP |
| chr6_31246632_G_A | 31246632 | 2.78 | 1.46 | 5.74 | 0.00319 | 0.06 | 0.08 | 0.03 | SNP |
| chr6_31246410_G_A | 31246410 | 2.69 | 1.44 | 5.41 | 0.00319 | 0.07 | 0.08 | 0.04 | SNP |
| chr6_31245177_G_C | 31245177 | 2.71 | 1.44 | 5.47 | 0.00321 | 0.07 | 0.08 | 0.04 | SNP |
| chr6_29892217_T_C | 29892217 | 1.76 | 1.21 | 2.60 | 0.00368 | 0.26 | 0.28 | 0.23 | SNP |
| chr6_29897770_G_A | 29897770 | 1.71 | 1.20 | 2.49 | 0.00371 | 0.27 | 0.29 | 0.24 | SNP |
| chr6_32629859_G_A | 32629859 | 0.57 | 0.38 | 0.83 | 0.00372 | 0.42 | 0.40 | 0.46 | SNP |
| chr6_30229267_G_C | 30229267 | 2.61 | 1.38 | 5.10 | 0.00381 | 0.12 | 0.12 | 0.10 | SNP |
| chr6_30867958_T_C | 30867958 | 0.58 | 0.40 | 0.84 | 0.00421 | 0.12 | 0.10 | 0.17 | SNP |
| chr6_29892229_T_G | 29892229 | 1.76 | 1.20 | 2.61 | 0.00424 | 0.26 | 0.27 | 0.23 | SNP |
| chr6_31365547_G_A | 31365547 | 2.10 | 1.28 | 3.57 | 0.00429 | 0.10 | 0.12 | 0.07 | SNP |
| chr6_31113106_G_A | 31113106 | 0.58 | 0.39 | 0.84 | 0.00447 | 0.29 | 0.28 | 0.31 | SNP |
| chr6_32786882_T_C | 32786882 | 2.31 | 1.33 | 4.27 | 0.00455 | 0.08 | 0.09 | 0.04 | SNP |
| chr6_33039625_T_C | 33039625 | 1.85 | 1.23 | 2.89 | 0.00465 | 0.12 | 0.14 | 0.08 | SNP |
| chr6_29892732_G_A | 29892732 | 1.72 | 1.19 | 2.54 | 0.00468 | 0.23 | 0.24 | 0.19 | SNP |
| chr6_33048640_A_G | 33048640 | 2.17 | 1.30 | 3.83 | 0.00471 | 0.10 | 0.12 | 0.06 | SNP |
| chr6_29893139_G_A | 29893139 | 1.77 | 1.20 | 2.67 | 0.00474 | 0.23 | 0.24 | 0.20 | SNP |
| chr6_29892233_T_C | 29892233 | 1.74 | 1.19 | 2.59 | 0.00486 | 0.26 | 0.27 | 0.23 | SNP |
| chr6_29896925_C_G | 29896925 | 1.59 | 1.15 | 2.21 | 0.00517 | 0.34 | 0.36 | 0.30 | SNP |
| chr6_31777946_C_T | 31777946 | 1.63 | 1.16 | 2.30 | 0.00535 | 0.36 | 0.38 | 0.32 | SNP |
| chr6_31118565_G_C | 31118565 | 0.59 | 0.40 | 0.85 | 0.00540 | 0.29 | 0.28 | 0.31 | SNP |
| chr6_31246969_A_G | 31246969 | 2.24 | 1.29 | 4.05 | 0.00541 | 0.09 | 0.11 | 0.06 | SNP |
| chr6_31244436_C_G | 31244436 | 2.74 | 1.39 | 5.88 | 0.00562 | 0.06 | 0.08 | 0.03 | SNP |
| chr6_31371587_C_G | 31371587 | 2.66 | 1.38 | 5.63 | 0.00597 | 0.07 | 0.08 | 0.04 | SNP |
| chr6_29910761_G_A | 29910761 | 1.74 | 1.18 | 2.62 | 0.00628 | 0.33 | 0.35 | 0.29 | SNP |
| chr6_29856279_G_T | 29856279 | 0.39 | 0.20 | 0.76 | 0.00647 | 0.04 | 0.03 | 0.08 | SNP |
| chr6_31239742_G_C | 31239742 | 2.65 | 1.35 | 5.58 | 0.00673 | 0.07 | 0.08 | 0.04 | SNP |
| chr6_29858357_A_G | 29858357 | 0.45 | 0.25 | 0.79 | 0.00673 | 0.04 | 0.03 | 0.07 | SNP |
| chr6_33043520_C_G | 33043520 | 2.21 | 1.28 | 4.05 | 0.00685 | 0.08 | 0.09 | 0.04 | SNP |
| chr6_31475513_G_A | 31475513 | 0.31 | 0.13 | 0.71 | 0.00700 | 0.02 | 0.01 | 0.04 | SNP |
| chr6_31921475_G_A | 31921475 | 1.61 | 1.14 | 2.29 | 0.00721 | 0.40 | 0.42 | 0.36 | SNP |
| chr6_31117695_G_A | 31117695 | 2.65 | 1.35 | 5.68 | 0.00754 | 0.05 | 0.07 | 0.03 | SNP |
| xHLA_2field_B_15_01 | 31321652 | 0.54 | 0.35 | 0.85 | 0.00763 | 0.11 | 0.10 | 0.14 | HLA (xHLA) |
| chr6_29896636_C_T | 29896636 | 1.66 | 1.15 | 2.43 | 0.00765 | 0.25 | 0.26 | 0.22 | SNP |
| chr6_30877760_A_C | 30877760 | 0.61 | 0.42 | 0.88 | 0.00770 | 0.14 | 0.12 | 0.18 | SNP |
| chr6_31133870_G_A | 31133870 | 2.64 | 1.34 | 5.65 | 0.00784 | 0.05 | 0.07 | 0.03 | SNP |
| chr6_31349819_A_C | 31349819 | 0.66 | 0.49 | 0.90 | 0.00797 | 0.44 | 0.42 | 0.46 | SNP |
| chr6_31117673_C_T | 31117673 | 2.64 | 1.34 | 5.68 | 0.00800 | 0.05 | 0.07 | 0.03 | SNP |
| chr6_31112484_C_T | 31112484 | 2.62 | 1.33 | 5.62 | 0.00824 | 0.05 | 0.07 | 0.03 | SNP |
| chr6_31126697_C_A | 31126697 | 2.62 | 1.33 | 5.62 | 0.00824 | 0.05 | 0.07 | 0.03 | SNP |
| chr6_31127694_A_G | 31127694 | 2.62 | 1.33 | 5.62 | 0.00824 | 0.05 | 0.07 | 0.03 | SNP |
| chr6_31127800_G_T | 31127800 | 2.62 | 1.33 | 5.62 | 0.00824 | 0.05 | 0.07 | 0.03 | SNP |
| chr6_29896713_G_A | 29896713 | 1.63 | 1.14 | 2.37 | 0.00826 | 0.25 | 0.27 | 0.22 | SNP |
| chr6_31112899_T_C | 31112899 | 0.58 | 0.38 | 0.87 | 0.00839 | 0.28 | 0.27 | 0.30 | SNP |
| chr6_31129707_C_T | 31129707 | 2.61 | 1.33 | 5.60 | 0.00841 | 0.05 | 0.07 | 0.03 | SNP |
| chr6_31592064_C_T | 31592064 | 0.40 | 0.20 | 0.79 | 0.00852 | 0.04 | 0.03 | 0.07 | SNP |
| chr6_31133663_T_C | 31133663 | 2.60 | 1.32 | 5.58 | 0.00866 | 0.05 | 0.07 | 0.03 | SNP |
| chr6_30883920_C_T | 30883920 | 1.52 | 1.11 | 2.08 | 0.00880 | 0.41 | 0.44 | 0.36 | SNP |
| chr6_32551568_C_G | 32551568 | 0.64 | 0.46 | 0.89 | 0.00882 | 0.31 | 0.28 | 0.36 | SNP |
| chr6_29893982_C_T | 29893982 | 1.75 | 1.16 | 2.71 | 0.00886 | 0.21 | 0.23 | 0.19 | SNP |
| chr6_32797488_T_C | 32797488 | 1.57 | 1.12 | 2.21 | 0.00908 | 0.33 | 0.36 | 0.28 | SNP |
| chr6_32633819_A_G | 32633819 | 0.60 | 0.41 | 0.88 | 0.00912 | 0.44 | 0.41 | 0.47 | SNP |
| xHLA_2field_DQB1_03_02 | 32627244 | 1.76 | 1.16 | 2.72 | 0.00926 | 0.22 | 0.24 | 0.19 | HLA (xHLA) |
| chr6_30882856_A_G | 30882856 | 1.53 | 1.11 | 2.11 | 0.00945 | 0.41 | 0.44 | 0.36 | SNP |
| chr6_31118898_A_T | 31118898 | 2.68 | 1.32 | 5.95 | 0.00963 | 0.05 | 0.06 | 0.03 | SNP |
| chr6_31125257_C_A | 31125257 | 2.58 | 1.30 | 5.57 | 0.00972 | 0.05 | 0.06 | 0.03 | SNP |
| chr6_32610401_G_A | 32610401 | 1.73 | 1.15 | 2.64 | 0.00977 | 0.30 | 0.31 | 0.26 | SNP |
| chr6_30849822_G_A | 30849822 | 0.62 | 0.43 | 0.89 | 0.00978 | 0.12 | 0.10 | 0.17 | SNP |
| chr6_31132956_G_C | 31132956 | 2.59 | 1.30 | 5.58 | 0.00982 | 0.05 | 0.06 | 0.03 | SNP |
| chr6_32629514_T_A | 32629514 | 0.49 | 0.29 | 0.84 | 0.00992 | 0.17 | 0.15 | 0.21 | SNP |
| chr6_31125402_C_G | 31125402 | 2.58 | 1.30 | 5.57 | 0.00994 | 0.05 | 0.06 | 0.03 | SNP |
| chr6_32942354_G_T | 32942354 | 0.31 | 0.13 | 0.76 | 0.01003 | 0.02 | 0.01 | 0.03 | SNP |
| chr6_32942355_C_G | 32942355 | 0.31 | 0.13 | 0.76 | 0.01003 | 0.02 | 0.01 | 0.03 | SNP |
| chr6_31894626_A_G | 31894626 | 1.53 | 1.11 | 2.13 | 0.01018 | 0.42 | 0.45 | 0.38 | SNP |
| chr6_32943108_T_A | 32943108 | 0.32 | 0.13 | 0.76 | 0.01034 | 0.02 | 0.01 | 0.03 | SNP |
| chr6_31240041_T_C | 31240041 | 1.64 | 1.13 | 2.42 | 0.01049 | 0.42 | 0.44 | 0.38 | SNP |
| chr6_29896773_C_T | 29896773 | 1.62 | 1.13 | 2.37 | 0.01055 | 0.25 | 0.26 | 0.22 | SNP |
| chr6_31320654_T_C | 31320654 | 1.64 | 1.13 | 2.42 | 0.01058 | 0.18 | 0.20 | 0.15 | SNP |
| chr6_30893831_G_A | 30893831 | 1.50 | 1.10 | 2.06 | 0.01059 | 0.41 | 0.44 | 0.36 | SNP |
| chr6_29896852_C_T | 29896852 | 1.62 | 1.13 | 2.37 | 0.01065 | 0.25 | 0.27 | 0.23 | SNP |
| chr6_31325764_C_G | 31325764 | 1.87 | 1.17 | 3.10 | 0.01092 | 0.10 | 0.12 | 0.07 | SNP |
| chr6_31371342_C_G | 31371342 | 0.29 | 0.11 | 0.75 | 0.01102 | 0.02 | 0.01 | 0.03 | SNP |
| chr6_32628103_C_T | 32628103 | 0.50 | 0.29 | 0.85 | 0.01132 | 0.19 | 0.16 | 0.23 | SNP |
| chr6_31116133_A_C | 31116133 | 2.54 | 1.28 | 5.48 | 0.01135 | 0.05 | 0.07 | 0.03 | SNP |
| chr6_30866342_C_G | 30866342 | 0.60 | 0.41 | 0.89 | 0.01157 | 0.14 | 0.12 | 0.19 | SNP |
| chr6_31325745_C_T | 31325745 | 0.67 | 0.50 | 0.91 | 0.01158 | 0.50 | 0.51 | 0.46 | SNP |
| chr6_32299592_G_A | 32299592 | 0.64 | 0.45 | 0.91 | 0.01181 | 0.29 | 0.27 | 0.36 | SNP |
| chr6_31326929_A_C | 31326929 | 1.88 | 1.17 | 3.14 | 0.01193 | 0.10 | 0.12 | 0.07 | SNP |
| chr6_30859355_G_A | 30859355 | 0.62 | 0.43 | 0.90 | 0.01203 | 0.13 | 0.11 | 0.17 | SNP |
| chr6_33632618_C_T | 33632618 | 0.38 | 0.17 | 0.81 | 0.01207 | 0.03 | 0.02 | 0.06 | SNP |
| chr6_31325317_C_T | 31325317 | 1.88 | 1.17 | 3.14 | 0.01217 | 0.10 | 0.11 | 0.07 | SNP |
| chr6_31326683_G_A | 31326683 | 1.88 | 1.17 | 3.14 | 0.01217 | 0.10 | 0.11 | 0.07 | SNP |
| chr6_31326948_C_A | 31326948 | 1.88 | 1.17 | 3.14 | 0.01217 | 0.10 | 0.11 | 0.07 | SNP |
| chr6_31320277_T_A | 31320277 | 0.68 | 0.51 | 0.92 | 0.01222 | 0.47 | 0.46 | 0.51 | SNP |
| chr6_31241270_A_G | 31241270 | 0.57 | 0.36 | 0.88 | 0.01253 | 0.24 | 0.23 | 0.25 | SNP |
| chr6_32628256_G_T | 32628256 | 0.51 | 0.30 | 0.87 | 0.01262 | 0.18 | 0.16 | 0.23 | SNP |
| chr6_32629504_C_T | 32629504 | 0.51 | 0.30 | 0.87 | 0.01267 | 0.17 | 0.15 | 0.21 | SNP |
| chr6_32304085_A_G | 32304085 | 2.12 | 1.20 | 3.95 | 0.01297 | 0.08 | 0.10 | 0.05 | SNP |

| | | | | | | | | | |
|---|---|---|---|---|---|---|---|---|---|
| chr6_32373378_A_T | 32373378 | 0.52 | 0.31 | 0.87 | 0.01311 | 0.09 | 0.08 | 0.12 | SNP |
| chr6_31129616_G_A | 31129616 | 0.62 | 0.42 | 0.90 | 0.01342 | 0.29 | 0.28 | 0.31 | SNP |
| xHLA_2field_C_04_01 | 31236526 | 2.18 | 1.21 | 4.21 | 0.01395 | 0.07 | 0.08 | 0.04 | HLA (xHLA) |
| chr6_29901865_G_C | 29901865 | 1.54 | 1.10 | 2.18 | 0.01407 | 0.29 | 0.30 | 0.27 | SNP |
| chr6_30890055_T_C | 30890055 | 1.48 | 1.08 | 2.04 | 0.01444 | 0.41 | 0.43 | 0.36 | SNP |
| chr6_32629259_T_C | 32629259 | 1.66 | 1.11 | 2.50 | 0.01454 | 0.23 | 0.24 | 0.20 | SNP |
| chr6_32184705_A_G | 32184705 | 1.54 | 1.09 | 2.20 | 0.01474 | 0.32 | 0.34 | 0.27 | SNP |
| chr6_31125569_G_T | 31125569 | 0.68 | 0.50 | 0.93 | 0.01481 | 0.43 | 0.42 | 0.45 | SNP |
| chr6_30878579_A_T | 30878579 | 0.64 | 0.45 | 0.92 | 0.01494 | 0.15 | 0.13 | 0.19 | SNP |
| chr6_32153409_A_C | 32153409 | 1.54 | 1.09 | 2.19 | 0.01501 | 0.44 | 0.47 | 0.40 | SNP |
| chr6_32187722_T_C | 32187722 | 1.50 | 1.08 | 2.08 | 0.01504 | 0.43 | 0.46 | 0.37 | SNP |
| chr6_31239501_G_T | 31239501 | 2.43 | 1.23 | 5.19 | 0.01509 | 0.06 | 0.08 | 0.04 | SNP |
| chr6_31320241_G_A | 31320241 | 1.73 | 1.12 | 2.72 | 0.01518 | 0.22 | 0.22 | 0.21 | SNP |
| chr6_29856552_C_T | 29856552 | 0.45 | 0.23 | 0.85 | 0.01532 | 0.04 | 0.03 | 0.06 | SNP |
| chr6_30887972_C_T | 30887972 | 0.64 | 0.45 | 0.92 | 0.01543 | 0.14 | 0.12 | 0.18 | SNP |
| chr6_31324764_C_A | 31324764 | 0.37 | 0.16 | 0.82 | 0.01556 | 0.03 | 0.02 | 0.05 | SNP |
| chr6_31321329_G_A | 31321329 | 2.52 | 1.24 | 5.62 | 0.01585 | 0.05 | 0.06 | 0.03 | SNP |
| chr6_31321336_G_A | 31321336 | 2.52 | 1.24 | 5.62 | 0.01585 | 0.05 | 0.06 | 0.03 | SNP |
| chr6_31462002_G_C | 31462002 | 0.53 | 0.32 | 0.89 | 0.01600 | 0.09 | 0.07 | 0.13 | SNP |
| chr6_31112925_T_C | 31112925 | 0.61 | 0.40 | 0.91 | 0.01602 | 0.28 | 0.28 | 0.29 | SNP |
| chr6_31105633_T_C | 31105633 | 0.63 | 0.43 | 0.92 | 0.01619 | 0.22 | 0.21 | 0.25 | SNP |
| chr6_31320562_C_T | 31320562 | 0.57 | 0.36 | 0.90 | 0.01631 | 0.20 | 0.20 | 0.22 | SNP |
| chr6_31126232_C_T | 31126232 | 2.53 | 1.23 | 5.67 | 0.01647 | 0.05 | 0.07 | 0.03 | SNP |
| chr6_30515043_T_C | 30515043 | 2.26 | 1.18 | 4.47 | 0.01654 | 0.12 | 0.12 | 0.12 | SNP |
| chr6_30876996_G_C | 30876996 | 0.65 | 0.45 | 0.92 | 0.01655 | 0.15 | 0.13 | 0.19 | SNP |
| chr6_31321110_C_T | 31321110 | 1.80 | 1.13 | 2.97 | 0.01658 | 0.10 | 0.11 | 0.07 | SNP |
| chr6_31843924_C_T | 31843924 | 1.53 | 1.08 | 2.17 | 0.01694 | 0.38 | 0.40 | 0.34 | SNP |
| chr6_31326080_T_C | 31326080 | 1.91 | 1.15 | 3.35 | 0.01701 | 0.08 | 0.10 | 0.06 | SNP |
| chr6_31320164_G_A | 31320164 | 1.67 | 1.10 | 2.57 | 0.01743 | 0.23 | 0.24 | 0.22 | SNP |
| chr6_31239430_C_T | 31239430 | 0.61 | 0.41 | 0.91 | 0.01767 | 0.46 | 0.44 | 0.52 | SNP |
| chr6_30880757_G_A | 30880757 | 1.46 | 1.07 | 1.99 | 0.01771 | 0.41 | 0.43 | 0.35 | SNP |
| chr6_30867666_T_C | 30867666 | 0.65 | 0.46 | 0.93 | 0.01777 | 0.15 | 0.13 | 0.19 | SNP |
| chr6_31126409_G_T | 31126409 | 2.49 | 1.22 | 5.59 | 0.01794 | 0.06 | 0.07 | 0.03 | SNP |
| chr6_31383239_T_C | 31383239 | 1.91 | 1.14 | 3.34 | 0.01808 | 0.09 | 0.11 | 0.06 | SNP |
| chr6_30882634_C_T | 30882634 | 1.49 | 1.07 | 2.09 | 0.01848 | 0.41 | 0.43 | 0.37 | SNP |
| chr6_33071754_C_A | 33071754 | 1.83 | 1.13 | 3.10 | 0.01848 | 0.09 | 0.10 | 0.06 | SNP |
| chr6_31236467_G_T | 31236467 | 0.71 | 0.54 | 0.94 | 0.01849 | 0.34 | 0.32 | 0.39 | SNP |
| chr6_30889389_T_C | 30889389 | 0.64 | 0.44 | 0.93 | 0.01901 | 0.13 | 0.11 | 0.17 | SNP |
| chr6_31466051_T_A | 31466051 | 0.55 | 0.33 | 0.91 | 0.01905 | 0.09 | 0.07 | 0.13 | SNP |
| chr6_31125158_T_C | 31125158 | 0.69 | 0.51 | 0.94 | 0.01938 | 0.43 | 0.42 | 0.45 | SNP |
| chr6_30877202_T_C | 30877202 | 0.65 | 0.45 | 0.93 | 0.01944 | 0.15 | 0.13 | 0.18 | SNP |
| chr6_32557962_A_G | 32557962 | 0.63 | 0.43 | 0.93 | 0.01955 | 0.23 | 0.21 | 0.28 | SNP |
| chr6_32134656_T_C | 32134656 | 0.50 | 0.28 | 0.90 | 0.01957 | 0.20 | 0.18 | 0.24 | SNP |
| chr6_32627934_G_A | 32627934 | 0.55 | 0.33 | 0.91 | 0.01969 | 0.18 | 0.15 | 0.21 | SNP |
| chr6_29856774_A_G | 29856774 | 1.72 | 1.10 | 2.75 | 0.01985 | 0.16 | 0.17 | 0.15 | SNP |
| chr6_32187605_G_A | 32187605 | 1.47 | 1.07 | 2.05 | 0.01999 | 0.45 | 0.48 | 0.39 | SNP |
| chr6_32410691_G_A | 32410691 | 0.55 | 0.33 | 0.91 | 0.02024 | 0.17 | 0.15 | 0.21 | SNP |
| chr6_32628420_C_T | 32628420 | 1.57 | 1.08 | 2.30 | 0.02031 | 0.45 | 0.47 | 0.41 | SNP |
| chr6_30888169_G_A | 30888169 | 1.44 | 1.06 | 1.96 | 0.02032 | 0.41 | 0.43 | 0.36 | SNP |
| chr6_33050078_A_G | 33050078 | 1.73 | 1.11 | 2.80 | 0.02048 | 0.10 | 0.11 | 0.07 | SNP |
| chr6_33050079_C_G | 33050079 | 1.73 | 1.11 | 2.80 | 0.02048 | 0.10 | 0.11 | 0.07 | SNP |
| chr6_31117619_C_T | 31117619 | 0.62 | 0.41 | 0.93 | 0.02070 | 0.29 | 0.28 | 0.30 | SNP |
| chr6_32633941_A_G | 32633941 | 0.55 | 0.33 | 0.92 | 0.02133 | 0.17 | 0.15 | 0.21 | SNP |
| chr6_31320309_T_C | 31320309 | 0.71 | 0.53 | 0.95 | 0.02138 | 0.46 | 0.44 | 0.49 | SNP |
| chr6_30885639_A_G | 30885639 | 1.43 | 1.06 | 1.96 | 0.02148 | 0.41 | 0.44 | 0.37 | SNP |
| chr6_30878919_T_C | 30878919 | 0.65 | 0.46 | 0.94 | 0.02163 | 0.15 | 0.13 | 0.18 | SNP |
| chr6_33050499_A_G | 33050499 | 1.72 | 1.10 | 2.79 | 0.02197 | 0.10 | 0.11 | 0.07 | SNP |
| chr6_32636138_A_G | 32636138 | 1.50 | 1.06 | 2.13 | 0.02246 | 0.32 | 0.34 | 0.28 | SNP |
| chr6_31549556_T_G | 31549556 | 0.50 | 0.27 | 0.91 | 0.02266 | 0.04 | 0.03 | 0.07 | SNP |
| chr6_32634318_C_A | 32634318 | 0.55 | 0.33 | 0.92 | 0.02296 | 0.17 | 0.15 | 0.21 | SNP |
| chr6_33052950_T_C | 33052950 | 1.75 | 1.10 | 2.88 | 0.02307 | 0.09 | 0.11 | 0.07 | SNP |
| chr6_29910801_C_A | 29910801 | 1.63 | 1.08 | 2.51 | 0.02317 | 0.27 | 0.28 | 0.24 | SNP |
| chr6_32374595_A_G | 32374595 | 0.51 | 0.28 | 0.91 | 0.02317 | 0.14 | 0.13 | 0.18 | SNP |
| chr6_32375796_A_T | 32375796 | 0.51 | 0.28 | 0.91 | 0.02317 | 0.14 | 0.13 | 0.18 | SNP |
| chr6_31236035_G_A | 31236035 | 1.95 | 1.12 | 3.59 | 0.02331 | 0.07 | 0.08 | 0.05 | SNP |
| chr6_31917540_T_C | 31917540 | 1.47 | 1.06 | 2.06 | 0.02340 | 0.40 | 0.42 | 0.36 | SNP |
| chr6_30850191_T_C | 30850191 | 0.66 | 0.47 | 0.95 | 0.02344 | 0.15 | 0.13 | 0.18 | SNP |
| chr6_30851194_C_G | 30851194 | 0.66 | 0.47 | 0.95 | 0.02344 | 0.15 | 0.13 | 0.18 | SNP |
| chr6_30854090_G_T | 30854090 | 0.66 | 0.47 | 0.95 | 0.02344 | 0.15 | 0.13 | 0.18 | SNP |
| chr6_30855251_G_A | 30855251 | 0.66 | 0.47 | 0.95 | 0.02344 | 0.15 | 0.13 | 0.18 | SNP |
| chr6_30857542_G_A | 30857542 | 0.66 | 0.47 | 0.95 | 0.02344 | 0.15 | 0.13 | 0.18 | SNP |
| chr6_31326959_C_T | 31326959 | 0.35 | 0.14 | 0.87 | 0.02345 | 0.02 | 0.01 | 0.03 | SNP |
| chr6_32793981_C_A | 32793981 | 1.52 | 1.06 | 2.20 | 0.02347 | 0.25 | 0.28 | 0.21 | SNP |
| chr6_32710407_T_C | 32710407 | 0.56 | 0.33 | 0.93 | 0.02367 | 0.09 | 0.08 | 0.11 | SNP |
| chr6_30859520_C_T | 30859520 | 0.66 | 0.46 | 0.95 | 0.02372 | 0.13 | 0.12 | 0.17 | SNP |
| chr6_29853750_G_T | 29853750 | 1.68 | 1.08 | 2.67 | 0.02386 | 0.16 | 0.17 | 0.15 | SNP |
| chr6_31241664_G_A | 31241664 | 2.17 | 1.13 | 4.32 | 0.02400 | 0.15 | 0.15 | 0.15 | SNP |
| chr6_31320642_C_T | 31320642 | 1.54 | 1.06 | 2.25 | 0.02416 | 0.18 | 0.20 | 0.15 | SNP |
| chr6_30887988_C_T | 30887988 | 1.42 | 1.05 | 1.93 | 0.02420 | 0.41 | 0.43 | 0.36 | SNP |
| chr6_32629282_A_T | 32629282 | 0.55 | 0.33 | 0.93 | 0.02423 | 0.17 | 0.15 | 0.21 | SNP |
| chr6_32629289_C_A | 32629289 | 0.56 | 0.33 | 0.93 | 0.02431 | 0.17 | 0.15 | 0.21 | SNP |
| chr6_31349844_A_G | 31349844 | 2.60 | 1.19 | 6.39 | 0.02436 | 0.04 | 0.05 | 0.02 | SNP |
| chr6_31324051_C_A | 31324051 | 2.01 | 1.12 | 3.81 | 0.02451 | 0.08 | 0.10 | 0.06 | SNP |
| chr6_33054659_A_G | 33054659 | 1.69 | 1.09 | 2.73 | 0.02489 | 0.10 | 0.11 | 0.07 | SNP |
| chr6_32780724_G_A | 32780724 | 0.71 | 0.53 | 0.96 | 0.02519 | 0.31 | 0.29 | 0.36 | SNP |
| chr6_32629347_T_C | 32629347 | 1.54 | 1.06 | 2.25 | 0.02523 | 0.45 | 0.47 | 0.42 | SNP |
| chr6_30877054_G_A | 30877054 | 0.66 | 0.46 | 0.95 | 0.02525 | 0.13 | 0.12 | 0.17 | SNP |
| chr6_32944575_T_C | 32944575 | 0.38 | 0.16 | 0.88 | 0.02525 | 0.02 | 0.01 | 0.03 | SNP |
| chr6_31243785_G_T | 31243785 | 1.59 | 1.07 | 2.40 | 0.02528 | 0.17 | 0.18 | 0.14 | SNP |
| chr6_32633879_A_G | 32633879 | 0.56 | 0.34 | 0.93 | 0.02545 | 0.18 | 0.15 | 0.21 | SNP |
| chr6_33050089_G_A | 33050089 | 1.70 | 1.08 | 2.76 | 0.02557 | 0.10 | 0.11 | 0.07 | SNP |
| chr6_30861000_C_T | 30861000 | 1.49 | 1.05 | 2.12 | 0.02564 | 0.42 | 0.44 | 0.39 | SNP |
| chr6_29911086_T_C | 29911086 | 1.56 | 1.06 | 2.32 | 0.02569 | 0.26 | 0.28 | 0.23 | SNP |
| chr6_32803408_T_A | 32803408 | 0.44 | 0.21 | 0.91 | 0.02573 | 0.03 | 0.02 | 0.05 | SNP |
| chr6_30867106_C_T | 30867106 | 0.67 | 0.47 | 0.95 | 0.02575 | 0.15 | 0.13 | 0.18 | SNP |
| chr6_32629091_C_G | 32629091 | 0.56 | 0.33 | 0.93 | 0.02582 | 0.17 | 0.15 | 0.21 | SNP |
| chr6_32634126_G_A | 32634126 | 0.56 | 0.33 | 0.93 | 0.02583 | 0.17 | 0.15 | 0.21 | SNP |
| chr6_30878769_C_T | 30878769 | 0.67 | 0.47 | 0.95 | 0.02595 | 0.15 | 0.13 | 0.18 | SNP |
| chr6_33053399_T_C | 33053399 | 1.71 | 1.09 | 2.81 | 0.02597 | 0.10 | 0.11 | 0.07 | SNP |
| chr6_33647936_C_T | 33647936 | 4.21 | 1.35 | 18.55 | 0.02598 | 0.02 | 0.03 | 0.01 | SNP |
| chr6_33050473_A_G | 33050473 | 1.69 | 1.08 | 2.74 | 0.02609 | 0.10 | 0.11 | 0.07 | SNP |
| chr6_32558183_G_T | 32558183 | 1.44 | 1.04 | 1.98 | 0.02612 | 0.45 | 0.43 | 0.50 | SNP |
| chr6_32798795_A_C | 32798795 | 1.42 | 1.05 | 1.94 | 0.02620 | 0.39 | 0.42 | 0.34 | SNP |
| chr6_31246324_A_C | 31246324 | 2.16 | 1.11 | 4.34 | 0.02643 | 0.15 | 0.15 | 0.15 | SNP |
| chr6_33055079_A_G | 33055079 | 1.68 | 1.08 | 2.71 | 0.02648 | 0.10 | 0.11 | 0.07 | SNP |
| chr6_30865115_G_A | 30865115 | 0.60 | 0.39 | 0.94 | 0.02655 | 0.14 | 0.12 | 0.18 | SNP |
| chr6_32634952_G_T | 32634952 | 0.56 | 0.33 | 0.94 | 0.02684 | 0.17 | 0.15 | 0.21 | SNP |
| chr6_32782112_C_T | 32782112 | 0.45 | 0.22 | 0.92 | 0.02699 | 0.03 | 0.02 | 0.05 | SNP |
| chr6_32631109_A_T | 32631109 | 0.50 | 0.27 | 0.93 | 0.02718 | 0.16 | 0.14 | 0.21 | SNP |
| chr6_30864700_C_T | 30864700 | 1.41 | 1.04 | 1.92 | 0.02727 | 0.41 | 0.43 | 0.36 | SNP |
| chr6_30920957_G_A | 30920957 | 0.67 | 0.47 | 0.96 | 0.02730 | 0.14 | 0.12 | 0.18 | SNP |
| chr6_30885686_T_A | 30885686 | 1.42 | 1.04 | 1.94 | 0.02732 | 0.41 | 0.43 | 0.36 | SNP |
| chr6_32629462_C_A | 32629462 | 0.56 | 0.33 | 0.94 | 0.02736 | 0.17 | 0.15 | 0.21 | SNP |
| chr6_31782544_C_A | 31782544 | 0.37 | 0.15 | 0.90 | 0.02742 | 0.02 | 0.01 | 0.03 | SNP |
| chr6_33050107_G_A | 33050107 | 1.69 | 1.08 | 2.75 | 0.02755 | 0.10 | 0.11 | 0.07 | SNP |
| chr6_32605696_G_T | 32605696 | 1.58 | 1.06 | 2.39 | 0.02761 | 0.31 | 0.32 | 0.28 | SNP |
| chr6_32977315_A_G | 32977315 | 0.68 | 0.48 | 0.96 | 0.02762 | 0.15 | 0.13 | 0.19 | SNP |
| chr6_32633749_A_G | 32633749 | 0.56 | 0.34 | 0.94 | 0.02764 | 0.17 | 0.15 | 0.21 | SNP |
| chr6_32633753_C_T | 32633753 | 0.56 | 0.34 | 0.94 | 0.02764 | 0.17 | 0.15 | 0.21 | SNP |
| chr6_32633816_C_G | 32633816 | 1.42 | 1.04 | 1.96 | 0.02766 | 0.43 | 0.45 | 0.40 | SNP |
| chr6_32628084_C_T | 32628084 | 0.43 | 0.20 | 0.91 | 0.02781 | 0.13 | 0.11 | 0.17 | SNP |
| chr6_32629716_T_C | 32629716 | 0.56 | 0.34 | 0.94 | 0.02787 | 0.17 | 0.15 | 0.21 | SNP |
| chr6_32010272_T_A | 32010272 | 1.52 | 1.05 | 2.21 | 0.02787 | 0.38 | 0.40 | 0.35 | SNP |
| chr6_33045208_T_C | 33045208 | 1.41 | 1.04 | 1.92 | 0.02787 | 0.24 | 0.25 | 0.20 | SNP |
| xHLA_2field_DPB1_04_01 | 33043767 | 0.75 | 0.58 | 0.97 | 0.02796 | 0.34 | 0.36 | 0.29 | HLA (xHLA) |
| chr6_32633928_G_A | 32633928 | 0.56 | 0.34 | 0.94 | 0.02805 | 0.17 | 0.15 | 0.21 | SNP |
| chr6_31247121_G_A | 31247121 | 0.70 | 0.50 | 0.96 | 0.02829 | 0.41 | 0.40 | 0.43 | SNP |
| chr6_31324064_T_A | 31324064 | 1.98 | 1.10 | 3.76 | 0.02841 | 0.08 | 0.09 | 0.06 | SNP |
| chr6_31378211_C_T | 31378211 | 0.40 | 0.17 | 0.91 | 0.02859 | 0.02 | 0.02 | 0.04 | SNP |
| xHLA_2field_B_35_01 | 31321652 | 2.87 | 1.19 | 8.11 | 0.02861 | 0.03 | 0.04 | 0.02 | HLA (xHLA) |
| chr6_32633287_C_T | 32633287 | 1.53 | 1.05 | 2.26 | 0.02871 | 0.39 | 0.41 | 0.34 | SNP |
| chr6_31116245_C_T | 31116245 | 3.00 | 1.21 | 9.07 | 0.02909 | 0.03 | 0.04 | 0.02 | SNP |
| chr6_31245911_G_A | 31245911 | 0.50 | 0.26 | 0.93 | 0.02926 | 0.04 | 0.03 | 0.06 | SNP |
| chr6_33055047_A_G | 33055047 | 1.64 | 1.07 | 2.63 | 0.02973 | 0.10 | 0.11 | 0.07 | SNP |
| chr6_31127745_C_A | 31127745 | 0.48 | 0.24 | 0.93 | 0.02982 | 0.03 | 0.02 | 0.05 | SNP |
| chr6_32628845_G_A | 32628845 | 1.52 | 1.04 | 2.24 | 0.02987 | 0.45 | 0.47 | 0.42 | SNP |

| | | | | | | | | | |
|---|---|---|---|---|---|---|---|---|---|
| chr6_29908676_T_A | 29908676 | 1.72 | 1.06 | 2.85 | 0.02987 | 0.15 | 0.16 | 0.14 | SNP |
| chr6_31125993_C_G | 31125993 | 2.30 | 1.13 | 5.15 | 0.02995 | 0.05 | 0.06 | 0.03 | SNP |
| chr6_33055123_A_G | 33055123 | 1.66 | 1.07 | 2.69 | 0.03021 | 0.10 | 0.11 | 0.07 | SNP |
| chr6_29913430_C_T | 29913430 | 1.56 | 1.05 | 2.36 | 0.03034 | 0.19 | 0.20 | 0.18 | SNP |
| chr6_30858857_C_T | 30858857 | 0.68 | 0.47 | 0.96 | 0.03034 | 0.15 | 0.13 | 0.18 | SNP |
| chr6_30885821_G_A | 30885821 | 1.41 | 1.04 | 1.93 | 0.03050 | 0.41 | 0.43 | 0.37 | SNP |
| chr6_31726688_C_A | 31726688 | 1.45 | 1.04 | 2.04 | 0.03068 | 0.38 | 0.40 | 0.34 | SNP |
| chr6_31245796_C_T | 31245796 | 2.11 | 1.09 | 4.24 | 0.03072 | 0.15 | 0.15 | 0.15 | SNP |
| chr6_31245821_A_G | 31245821 | 2.12 | 1.09 | 4.27 | 0.03083 | 0.15 | 0.15 | 0.15 | SNP |
| chr6_33053682_G_A | 33053682 | 1.64 | 1.06 | 2.62 | 0.03092 | 0.10 | 0.11 | 0.07 | SNP |
| chr6_33053772_G_A | 33053772 | 1.64 | 1.06 | 2.62 | 0.03092 | 0.10 | 0.11 | 0.07 | SNP |
| chr6_33053780_T_A | 33053780 | 1.64 | 1.06 | 2.62 | 0.03092 | 0.10 | 0.11 | 0.07 | SNP |
| chr6_33053789_A_G | 33053789 | 1.64 | 1.06 | 2.62 | 0.03092 | 0.10 | 0.11 | 0.07 | SNP |
| chr6_33053868_A_G | 33053868 | 1.64 | 1.06 | 2.62 | 0.03092 | 0.10 | 0.11 | 0.07 | SNP |
| chr6_33053871_A_G | 33053871 | 1.64 | 1.06 | 2.62 | 0.03092 | 0.10 | 0.11 | 0.07 | SNP |
| chr6_33053877_G_A | 33053877 | 1.64 | 1.06 | 2.62 | 0.03092 | 0.10 | 0.11 | 0.07 | SNP |
| chr6_33053887_A_G | 33053887 | 1.64 | 1.06 | 2.62 | 0.03092 | 0.10 | 0.11 | 0.07 | SNP |
| chr6_33053890_G_A | 33053890 | 1.64 | 1.06 | 2.62 | 0.03092 | 0.10 | 0.11 | 0.07 | SNP |
| chr6_33053982_G_C | 33053982 | 1.64 | 1.06 | 2.62 | 0.03092 | 0.10 | 0.11 | 0.07 | SNP |
| chr6_33054091_C_T | 33054091 | 1.64 | 1.06 | 2.62 | 0.03092 | 0.10 | 0.11 | 0.07 | SNP |
| chr6_33054141_T_C | 33054141 | 1.64 | 1.06 | 2.62 | 0.03092 | 0.10 | 0.11 | 0.07 | SNP |
| chr6_33054142_G_C | 33054142 | 1.64 | 1.06 | 2.62 | 0.03092 | 0.10 | 0.11 | 0.07 | SNP |
| chr6_33054148_C_T | 33054148 | 1.64 | 1.06 | 2.62 | 0.03092 | 0.10 | 0.11 | 0.07 | SNP |
| chr6_33054149_T_C | 33054149 | 1.64 | 1.06 | 2.62 | 0.03092 | 0.10 | 0.11 | 0.07 | SNP |
| chr6_33054152_A_G | 33054152 | 1.64 | 1.06 | 2.62 | 0.03092 | 0.10 | 0.11 | 0.07 | SNP |
| chr6_33054155_C_T | 33054155 | 1.64 | 1.06 | 2.62 | 0.03092 | 0.10 | 0.11 | 0.07 | SNP |
| chr6_33054156_A_G | 33054156 | 1.64 | 1.06 | 2.62 | 0.03092 | 0.10 | 0.11 | 0.07 | SNP |
| chr6_33054231_C_T | 33054231 | 1.64 | 1.06 | 2.62 | 0.03092 | 0.10 | 0.11 | 0.07 | SNP |
| chr6_33054235_T_G | 33054235 | 1.64 | 1.06 | 2.62 | 0.03092 | 0.10 | 0.11 | 0.07 | SNP |
| chr6_33054268_C_A | 33054268 | 1.64 | 1.06 | 2.62 | 0.03092 | 0.10 | 0.11 | 0.07 | SNP |
| chr6_33054280_T_C | 33054280 | 1.64 | 1.06 | 2.62 | 0.03092 | 0.10 | 0.11 | 0.07 | SNP |
| chr6_33054281_G_A | 33054281 | 1.64 | 1.06 | 2.62 | 0.03092 | 0.10 | 0.11 | 0.07 | SNP |
| chr6_33054302_T_C | 33054302 | 1.64 | 1.06 | 2.62 | 0.03092 | 0.10 | 0.11 | 0.07 | SNP |
| chr6_33054325_G_C | 33054325 | 1.64 | 1.06 | 2.62 | 0.03092 | 0.10 | 0.11 | 0.07 | SNP |
| chr6_33054331_T_C | 33054331 | 1.64 | 1.06 | 2.62 | 0.03092 | 0.10 | 0.11 | 0.07 | SNP |
| chr6_33054457_A_G | 33054457 | 1.64 | 1.06 | 2.62 | 0.03092 | 0.10 | 0.11 | 0.07 | SNP |
| chr6_33054550_G_A | 33054550 | 1.64 | 1.06 | 2.62 | 0.03092 | 0.10 | 0.11 | 0.07 | SNP |
| chr6_33054579_C_T | 33054579 | 1.64 | 1.06 | 2.62 | 0.03092 | 0.10 | 0.11 | 0.07 | SNP |
| chr6_33054586_A_G | 33054586 | 1.64 | 1.06 | 2.62 | 0.03092 | 0.10 | 0.11 | 0.07 | SNP |
| chr6_33054595_C_T | 33054595 | 1.64 | 1.06 | 2.62 | 0.03092 | 0.10 | 0.11 | 0.07 | SNP |
| chr6_33054619_G_A | 33054619 | 1.64 | 1.06 | 2.62 | 0.03092 | 0.10 | 0.11 | 0.07 | SNP |
| chr6_33054656_A_C | 33054656 | 1.64 | 1.06 | 2.62 | 0.03092 | 0.10 | 0.11 | 0.07 | SNP |
| chr6_33054675_C_G | 33054675 | 1.64 | 1.06 | 2.62 | 0.03092 | 0.10 | 0.11 | 0.07 | SNP |
| chr6_33054683_A_G | 33054683 | 1.64 | 1.06 | 2.62 | 0.03092 | 0.10 | 0.11 | 0.07 | SNP |
| chr6_33054687_G_A | 33054687 | 1.64 | 1.06 | 2.62 | 0.03092 | 0.10 | 0.11 | 0.07 | SNP |
| chr6_33054711_G_T | 33054711 | 1.64 | 1.06 | 2.62 | 0.03092 | 0.10 | 0.11 | 0.07 | SNP |
| chr6_33054721_C_T | 33054721 | 1.64 | 1.06 | 2.62 | 0.03092 | 0.10 | 0.11 | 0.07 | SNP |
| chr6_33054807_A_G | 33054807 | 1.64 | 1.06 | 2.62 | 0.03092 | 0.10 | 0.11 | 0.07 | SNP |
| chr6_33054890_C_T | 33054890 | 1.64 | 1.06 | 2.62 | 0.03092 | 0.10 | 0.11 | 0.07 | SNP |
| chr6_31429913_G_C | 31429913 | 0.43 | 0.20 | 0.93 | 0.03099 | 0.03 | 0.02 | 0.04 | SNP |
| xHLA_2field_DRB1_03_01 | 32546552 | 0.43 | 0.20 | 0.92 | 0.03108 | 0.12 | 0.11 | 0.16 | HLA (xHLA) |
| chr6_33055009_C_A | 33055009 | 1.64 | 1.06 | 2.62 | 0.03136 | 0.10 | 0.11 | 0.07 | SNP |
| chr6_32628559_T_C | 32628559 | 0.56 | 0.33 | 0.95 | 0.03148 | 0.17 | 0.15 | 0.21 | SNP |
| chr6_32628560_G_T | 32628560 | 0.56 | 0.33 | 0.95 | 0.03164 | 0.17 | 0.15 | 0.21 | SNP |
| chr6_32629331_A_G | 32629331 | 1.51 | 1.04 | 2.20 | 0.03177 | 0.45 | 0.47 | 0.42 | SNP |
| chr6_31544189_A_G | 31544189 | 0.53 | 0.29 | 0.95 | 0.03183 | 0.05 | 0.04 | 0.07 | SNP |
| chr6_29897225_C_T | 29897225 | 0.53 | 0.29 | 0.94 | 0.03186 | 0.04 | 0.03 | 0.06 | SNP |
| chr6_29858253_G_A | 29858253 | 1.64 | 1.05 | 2.62 | 0.03188 | 0.16 | 0.17 | 0.15 | SNP |
| chr6_31324953_T_C | 31324953 | 0.63 | 0.41 | 0.96 | 0.03206 | 0.40 | 0.39 | 0.41 | SNP |
| chr6_30882781_G_T | 30882781 | 1.41 | 1.03 | 1.93 | 0.03211 | 0.41 | 0.43 | 0.36 | SNP |
| chr6_33048542_C_T | 33048542 | 1.36 | 1.03 | 1.81 | 0.03225 | 0.29 | 0.30 | 0.24 | SNP |
| chr6_31379059_A_T | 31379059 | 0.36 | 0.14 | 0.92 | 0.03249 | 0.02 | 0.01 | 0.03 | SNP |
| chr6_32629509_A_C | 32629509 | 1.50 | 1.04 | 2.19 | 0.03251 | 0.46 | 0.47 | 0.43 | SNP |
| chr6_33053455_C_T | 33053455 | 1.67 | 1.06 | 2.73 | 0.03255 | 0.10 | 0.11 | 0.07 | SNP |
| chr6_31322790_T_A | 31322790 | 2.35 | 1.09 | 5.24 | 0.03258 | 0.13 | 0.13 | 0.13 | SNP |
| chr6_32633897_T_A | 32633897 | 0.57 | 0.34 | 0.96 | 0.03269 | 0.17 | 0.15 | 0.21 | SNP |
| chr6_32633898_C_A | 32633898 | 0.57 | 0.34 | 0.96 | 0.03269 | 0.17 | 0.15 | 0.21 | SNP |
| chr6_32634613_C_T | 32634613 | 0.57 | 0.34 | 0.96 | 0.03279 | 0.17 | 0.15 | 0.21 | SNP |
| chr6_31324955_C_T | 31324955 | 0.62 | 0.40 | 0.96 | 0.03280 | 0.12 | 0.11 | 0.14 | SNP |
| chr6_32629417_G_C | 32629417 | 0.57 | 0.34 | 0.96 | 0.03292 | 0.17 | 0.15 | 0.21 | SNP |
| chr6_32629400_A_G | 32629400 | 1.50 | 1.04 | 2.19 | 0.03293 | 0.46 | 0.48 | 0.43 | SNP |
| chr6_31239733_C_T | 31239733 | 0.48 | 0.24 | 0.95 | 0.03302 | 0.05 | 0.03 | 0.07 | SNP |
| chr6_31239727_C_A | 31239727 | 0.48 | 0.24 | 0.95 | 0.03305 | 0.05 | 0.03 | 0.07 | SNP |
| chr6_31239601_C_T | 31239601 | 0.47 | 0.23 | 0.94 | 0.03319 | 0.04 | 0.03 | 0.06 | SNP |
| chr6_31236862_C_T | 31236862 | 0.51 | 0.27 | 0.95 | 0.03326 | 0.04 | 0.03 | 0.06 | SNP |
| chr6_31237040_A_T | 31237040 | 0.51 | 0.27 | 0.95 | 0.03326 | 0.04 | 0.03 | 0.06 | SNP |
| chr6_31237254_C_T | 31237254 | 0.51 | 0.27 | 0.95 | 0.03326 | 0.04 | 0.03 | 0.06 | SNP |
| chr6_31238179_C_T | 31238179 | 0.51 | 0.27 | 0.95 | 0.03326 | 0.04 | 0.03 | 0.06 | SNP |
| chr6_31238334_C_T | 31238334 | 0.51 | 0.27 | 0.95 | 0.03326 | 0.04 | 0.03 | 0.06 | SNP |
| chr6_32627851_G_A | 32627851 | 0.56 | 0.33 | 0.96 | 0.03354 | 0.20 | 0.18 | 0.23 | SNP |
| chr6_31122126_C_G | 31122126 | 0.69 | 0.49 | 0.97 | 0.03362 | 0.42 | 0.40 | 0.44 | SNP |
| chr6_33053968_T_A | 33053968 | 1.63 | 1.06 | 2.61 | 0.03365 | 0.10 | 0.11 | 0.07 | SNP |
| chr6_33053971_T_C | 33053971 | 1.63 | 1.06 | 2.61 | 0.03365 | 0.10 | 0.11 | 0.07 | SNP |
| chr6_33050125_G_A | 33050125 | 1.68 | 1.06 | 2.78 | 0.03369 | 0.09 | 0.10 | 0.07 | SNP |
| chr6_33054353_G_A | 33054353 | 1.63 | 1.05 | 2.61 | 0.03376 | 0.10 | 0.11 | 0.07 | SNP |
| chr6_33054355_A_C | 33054355 | 1.63 | 1.05 | 2.61 | 0.03376 | 0.10 | 0.11 | 0.07 | SNP |
| chr6_32627830_C_T | 32627830 | 0.57 | 0.34 | 0.96 | 0.03378 | 0.17 | 0.15 | 0.21 | SNP |
| chr6_32635197_T_C | 32635197 | 0.57 | 0.34 | 0.96 | 0.03378 | 0.17 | 0.15 | 0.21 | SNP |
| chr6_31510105_G_A | 31510105 | 1.60 | 1.04 | 2.47 | 0.03379 | 0.31 | 0.33 | 0.27 | SNP |
| chr6_31275663_C_A | 31275663 | 1.61 | 1.05 | 2.53 | 0.03380 | 0.16 | 0.17 | 0.14 | SNP |
| chr6_31350232_G_A | 31350232 | 0.62 | 0.40 | 0.96 | 0.03384 | 0.23 | 0.23 | 0.24 | SNP |
| chr6_32339925_T_A | 32339925 | 1.41 | 1.03 | 1.94 | 0.03418 | 0.32 | 0.34 | 0.29 | SNP |
| chr6_32634243_G_A | 32634243 | 0.58 | 0.34 | 0.96 | 0.03441 | 0.17 | 0.15 | 0.21 | SNP |
| chr6_32636364_G_A | 32636364 | 1.40 | 1.03 | 1.92 | 0.03455 | 0.43 | 0.45 | 0.40 | SNP |
| chr6_32627494_T_C | 32627494 | 1.46 | 1.03 | 2.08 | 0.03473 | 0.35 | 0.36 | 0.32 | SNP |
| chr6_31246323_A_G | 31246323 | 0.51 | 0.27 | 0.96 | 0.03479 | 0.04 | 0.03 | 0.06 | SNP |
| chr6_31243846_G_T | 31243846 | 2.08 | 1.07 | 4.21 | 0.03491 | 0.15 | 0.15 | 0.15 | SNP |
| chr6_32628558_G_A | 32628558 | 0.57 | 0.33 | 0.96 | 0.03492 | 0.17 | 0.15 | 0.20 | SNP |
| chr6_33050118_C_T | 33050118 | 1.67 | 1.05 | 2.73 | 0.03511 | 0.09 | 0.11 | 0.07 | SNP |
| chr6_31239999_A_T | 31239999 | 0.55 | 0.31 | 0.96 | 0.03525 | 0.06 | 0.05 | 0.08 | SNP |
| chr6_32786917_A_G | 32786917 | 1.48 | 1.03 | 2.15 | 0.03526 | 0.25 | 0.27 | 0.21 | SNP |
| chr6_31239100_A_G | 31239100 | 0.48 | 0.24 | 0.95 | 0.03554 | 0.04 | 0.03 | 0.06 | SNP |
| chr6_30850582_G_A | 30850582 | 1.40 | 1.03 | 1.92 | 0.03555 | 0.40 | 0.42 | 0.36 | SNP |
| chr6_31321407_C_T | 31321407 | 1.64 | 1.05 | 2.64 | 0.03558 | 0.10 | 0.11 | 0.08 | SNP |
| chr6_31239869_T_C | 31239869 | 0.73 | 0.55 | 0.98 | 0.03558 | 0.29 | 0.27 | 0.33 | SNP |
| chr6_33054046_T_G | 33054046 | 1.62 | 1.05 | 2.61 | 0.03560 | 0.10 | 0.11 | 0.07 | SNP |
| chr6_33054057_G_A | 33054057 | 1.62 | 1.05 | 2.61 | 0.03560 | 0.10 | 0.11 | 0.07 | SNP |
| chr6_33054071_G_C | 33054071 | 1.62 | 1.05 | 2.61 | 0.03560 | 0.10 | 0.11 | 0.07 | SNP |
| chr6_32604585_A_T | 32604585 | 1.57 | 1.03 | 2.41 | 0.03583 | 0.31 | 0.32 | 0.29 | SNP |
| chr6_30851305_G_C | 30851305 | 0.68 | 0.48 | 0.98 | 0.03589 | 0.14 | 0.13 | 0.18 | SNP |
| chr6_32633697_C_A | 32633697 | 0.58 | 0.35 | 0.97 | 0.03603 | 0.17 | 0.15 | 0.21 | SNP |
| chr6_33050526_C_T | 33050526 | 1.65 | 1.05 | 2.68 | 0.03605 | 0.10 | 0.11 | 0.07 | SNP |
| chr6_31239630_C_T | 31239630 | 0.47 | 0.23 | 0.96 | 0.03614 | 0.04 | 0.03 | 0.07 | SNP |
| chr6_33045255_T_C | 33045255 | 1.38 | 1.03 | 1.88 | 0.03615 | 0.24 | 0.25 | 0.20 | SNP |
| chr6_32408842_A_G | 32408842 | 0.59 | 0.35 | 0.97 | 0.03620 | 0.17 | 0.15 | 0.21 | SNP |
| chr6_32411376_T_G | 32411376 | 0.59 | 0.35 | 0.97 | 0.03620 | 0.17 | 0.15 | 0.21 | SNP |
| chr6_31237918_G_A | 31237918 | 0.54 | 0.30 | 0.96 | 0.03631 | 0.05 | 0.04 | 0.07 | SNP |
| chr6_32409060_G_A | 32409060 | 0.58 | 0.35 | 0.97 | 0.03634 | 0.16 | 0.15 | 0.20 | SNP |
| chr6_29895945_A_T | 29895945 | 0.38 | 0.15 | 0.95 | 0.03664 | 0.02 | 0.01 | 0.03 | SNP |
| chr6_29943355_C_T | 29943355 | 0.49 | 0.25 | 0.96 | 0.03665 | 0.04 | 0.03 | 0.06 | SNP |
| chr6_33071708_G_A | 33071708 | 1.72 | 1.05 | 2.92 | 0.03666 | 0.09 | 0.10 | 0.06 | SNP |
| chr6_31113149_T_A | 31113149 | 0.50 | 0.25 | 0.96 | 0.03672 | 0.03 | 0.02 | 0.05 | SNP |
| chr6_33054413_T_G | 33054413 | 1.62 | 1.05 | 2.61 | 0.03674 | 0.10 | 0.11 | 0.07 | SNP |
| chr6_33054414_T_A | 33054414 | 1.62 | 1.05 | 2.61 | 0.03674 | 0.10 | 0.11 | 0.07 | SNP |
| chr6_31323468_G_T | 31323468 | 1.76 | 1.05 | 3.05 | 0.03680 | 0.09 | 0.10 | 0.06 | SNP |
| chr6_32627784_C_T | 32627784 | 0.58 | 0.35 | 0.97 | 0.03682 | 0.17 | 0.15 | 0.21 | SNP |
| chr6_33050521_G_T | 33050521 | 1.65 | 1.05 | 2.68 | 0.03683 | 0.10 | 0.11 | 0.07 | SNP |
| chr6_33050525_T_C | 33050525 | 1.64 | 1.05 | 2.68 | 0.03684 | 0.10 | 0.11 | 0.07 | SNP |
| chr6_32298814_C_A | 32298814 | 0.64 | 0.42 | 0.98 | 0.03685 | 0.21 | 0.19 | 0.26 | SNP |
| chr6_32299822_G_A | 32299822 | 0.64 | 0.42 | 0.98 | 0.03685 | 0.21 | 0.19 | 0.26 | SNP |
| chr6_32559714_G_A | 32559714 | 0.70 | 0.50 | 0.98 | 0.03691 | 0.36 | 0.34 | 0.41 | SNP |
| chr6_32635998_G_T | 32635998 | 0.58 | 0.35 | 0.97 | 0.03698 | 0.17 | 0.15 | 0.21 | SNP |
| chr6_32605178_T_C | 32605178 | 1.53 | 1.03 | 2.31 | 0.03711 | 0.30 | 0.32 | 0.27 | SNP |
| chr6_32629535_C_A | 32629535 | 1.48 | 1.03 | 2.16 | 0.03719 | 0.46 | 0.47 | 0.43 | SNP |
| chr6_31324941_C_T | 31324941 | 0.37 | 0.14 | 0.95 | 0.03727 | 0.02 | 0.01 | 0.03 | SNP |
| chr6_30884806_A_C | 30884806 | 1.40 | 1.02 | 1.91 | 0.03729 | 0.42 | 0.44 | 0.37 | SNP |

| | | | | | | | | | |
|---|---|---|---|---|---|---|---|---|---|
| chr6_32406611_C_A | 32406611 | 0.59 | 0.36 | 0.97 | 0.03740 | 0.17 | 0.15 | 0.21 | SNP |
| chr6_29895626_T_A | 29895626 | 1.63 | 1.04 | 2.61 | 0.03745 | 0.17 | 0.17 | 0.15 | SNP |
| chr6_29892815_A_G | 29892815 | 1.61 | 1.04 | 2.55 | 0.03755 | 0.17 | 0.17 | 0.16 | SNP |
| chr6_33054342_T_A | 33054342 | 1.61 | 1.04 | 2.59 | 0.03770 | 0.10 | 0.11 | 0.07 | SNP |
| chr6_33054373_C_T | 33054373 | 1.61 | 1.04 | 2.59 | 0.03770 | 0.10 | 0.11 | 0.07 | SNP |
| chr6_33054381_T_C | 33054381 | 1.61 | 1.04 | 2.59 | 0.03770 | 0.10 | 0.11 | 0.07 | SNP |
| chr6_33054386_C_T | 33054386 | 1.61 | 1.04 | 2.59 | 0.03770 | 0.10 | 0.11 | 0.07 | SNP |
| chr6_32636172_C_T | 32636172 | 1.44 | 1.02 | 2.03 | 0.03777 | 0.32 | 0.34 | 0.29 | SNP |
| chr6_32636154_C_G | 32636154 | 1.44 | 1.02 | 2.04 | 0.03783 | 0.32 | 0.33 | 0.29 | SNP |
| chr6_32629269_C_T | 32629269 | 1.45 | 1.02 | 2.06 | 0.03790 | 0.31 | 0.33 | 0.28 | SNP |
| chr6_32606987_T_G | 32606987 | 1.52 | 1.03 | 2.28 | 0.03801 | 0.31 | 0.32 | 0.28 | SNP |
| chr6_29911203_A_C | 29911203 | 1.62 | 1.04 | 2.60 | 0.03801 | 0.14 | 0.16 | 0.11 | SNP |
| chr6_31778077_T_G | 31778077 | 0.40 | 0.17 | 0.96 | 0.03806 | 0.02 | 0.01 | 0.03 | SNP |
| chr6_29913374_C_G | 29913374 | 1.68 | 1.04 | 2.77 | 0.03815 | 0.15 | 0.15 | 0.14 | SNP |
| chr6_32797684_A_G | 32797684 | 0.71 | 0.51 | 0.98 | 0.03817 | 0.32 | 0.30 | 0.38 | SNP |
| chr6_32635954_G_A | 32635954 | 1.51 | 1.03 | 2.26 | 0.03820 | 0.22 | 0.24 | 0.20 | SNP |
| chr6_30920890_G_A | 30920890 | 1.39 | 1.02 | 1.90 | 0.03832 | 0.41 | 0.43 | 0.37 | SNP |
| chr6_31239297_G_C | 31239297 | 0.48 | 0.24 | 0.96 | 0.03836 | 0.04 | 0.03 | 0.06 | SNP |
| chr6_29913137_A_G | 29913137 | 1.68 | 1.04 | 2.77 | 0.03841 | 0.15 | 0.15 | 0.14 | SNP |
| chr6_31325990_C_A | 31325990 | 1.75 | 1.05 | 3.03 | 0.03849 | 0.09 | 0.10 | 0.06 | SNP |
| chr6_31326024_T_G | 31326024 | 1.75 | 1.05 | 3.03 | 0.03849 | 0.09 | 0.10 | 0.06 | SNP |
| chr6_33052986_T_C | 33052986 | 1.61 | 1.04 | 2.58 | 0.03853 | 0.10 | 0.11 | 0.07 | SNP |
| chr6_33053723_T_C | 33053723 | 1.61 | 1.04 | 2.58 | 0.03853 | 0.10 | 0.11 | 0.07 | SNP |
| chr6_33053728_C_T | 33053728 | 1.61 | 1.04 | 2.58 | 0.03853 | 0.10 | 0.11 | 0.07 | SNP |
| chr6_29911684_C_T | 29911684 | 1.52 | 1.03 | 2.29 | 0.03861 | 0.20 | 0.20 | 0.18 | SNP |
| chr6_31239776_A_C | 31239776 | 1.45 | 1.02 | 2.08 | 0.03871 | 0.29 | 0.30 | 0.25 | SNP |
| chr6_32339647_T_C | 32339647 | 0.69 | 0.49 | 0.98 | 0.03881 | 0.21 | 0.19 | 0.26 | SNP |
| chr6_32627652_C_G | 32627652 | 1.48 | 1.02 | 2.15 | 0.03894 | 0.46 | 0.48 | 0.43 | SNP |
| chr6_32627818_C_A | 32627818 | 1.45 | 1.02 | 2.07 | 0.03913 | 0.28 | 0.30 | 0.25 | SNP |
| chr6_32629650_T_C | 32629650 | 0.59 | 0.35 | 0.98 | 0.03922 | 0.17 | 0.15 | 0.21 | SNP |
| chr6_32632753_C_T | 32632753 | 0.55 | 0.31 | 0.97 | 0.03935 | 0.19 | 0.18 | 0.22 | SNP |
| chr6_31324750_G_A | 31324750 | 1.87 | 1.05 | 3.49 | 0.03977 | 0.08 | 0.09 | 0.06 | SNP |
| chr6_31127037_C_T | 31127037 | 0.50 | 0.26 | 0.97 | 0.03998 | 0.03 | 0.02 | 0.05 | SNP |
| xHLA_2field_C_01_02 | 31236526 | 0.52 | 0.27 | 0.97 | 0.04004 | 0.04 | 0.03 | 0.06 | HLA (xHLA) |
| chr6_31367989_C_A | 31367989 | 1.67 | 1.04 | 2.78 | 0.04006 | 0.11 | 0.12 | 0.08 | SNP |
| chr6_32190390_T_G | 32190390 | 0.69 | 0.48 | 0.98 | 0.04024 | 0.40 | 0.38 | 0.45 | SNP |
| chr6_33053902_A_G | 33053902 | 1.61 | 1.04 | 2.59 | 0.04050 | 0.10 | 0.11 | 0.07 | SNP |
| chr6_33053906_G_T | 33053906 | 1.61 | 1.04 | 2.59 | 0.04050 | 0.10 | 0.11 | 0.07 | SNP |
| chr6_31241311_A_G | 31241311 | 2.01 | 1.05 | 3.98 | 0.04058 | 0.15 | 0.15 | 0.15 | SNP |
| chr6_32917544_C_T | 32917544 | 0.64 | 0.42 | 0.98 | 0.04059 | 0.09 | 0.08 | 0.12 | SNP |
| chr6_32634584_C_G | 32634584 | 0.58 | 0.35 | 0.98 | 0.04069 | 0.17 | 0.15 | 0.21 | SNP |
| chr6_33045147_A_T | 33045147 | 1.41 | 1.02 | 1.98 | 0.04081 | 0.22 | 0.22 | 0.18 | SNP |
| chr6_31383824_G_A | 31383824 | 0.49 | 0.24 | 0.98 | 0.04098 | 0.03 | 0.03 | 0.04 | SNP |
| chr6_29909071_G_A | 29909071 | 1.52 | 1.02 | 2.28 | 0.04102 | 0.19 | 0.20 | 0.18 | SNP |
| chr6_29909797_C_T | 29909797 | 1.52 | 1.02 | 2.28 | 0.04102 | 0.19 | 0.20 | 0.18 | SNP |
| chr6_32629066_T_C | 32629066 | 1.44 | 1.02 | 2.05 | 0.04108 | 0.32 | 0.33 | 0.29 | SNP |
| chr6_33054400_T_C | 33054400 | 1.61 | 1.03 | 2.58 | 0.04115 | 0.10 | 0.11 | 0.07 | SNP |
| chr6_33054404_A_G | 33054404 | 1.61 | 1.03 | 2.58 | 0.04115 | 0.10 | 0.11 | 0.07 | SNP |
| chr6_33054410_A_C | 33054410 | 1.61 | 1.03 | 2.58 | 0.04115 | 0.10 | 0.11 | 0.07 | SNP |
| chr6_30887311_C_T | 30887311 | 1.42 | 1.02 | 2.00 | 0.04123 | 0.40 | 0.43 | 0.35 | SNP |
| chr6_29858840_A_C | 29858840 | 1.60 | 1.03 | 2.54 | 0.04138 | 0.16 | 0.17 | 0.15 | SNP |
| chr6_31380449_C_T | 31380449 | 1.46 | 1.02 | 2.10 | 0.04156 | 0.33 | 0.34 | 0.30 | SNP |
| chr6_31245061_G_T | 31245061 | 0.70 | 0.50 | 0.99 | 0.04160 | 0.16 | 0.15 | 0.20 | SNP |
| chr6_32608478_T_C | 32608478 | 1.52 | 1.02 | 2.30 | 0.04162 | 0.30 | 0.32 | 0.27 | SNP |
| chr6_32629398_G_C | 32629398 | 1.43 | 1.02 | 2.01 | 0.04171 | 0.33 | 0.35 | 0.30 | SNP |
| chr6_30857988_T_C | 30857988 | 0.69 | 0.48 | 0.99 | 0.04173 | 0.14 | 0.13 | 0.18 | SNP |
| chr6_32634467_C_T | 32634467 | 0.58 | 0.35 | 0.98 | 0.04184 | 0.17 | 0.15 | 0.21 | SNP |
| chr6_32605132_T_C | 32605132 | 1.49 | 1.02 | 2.20 | 0.04231 | 0.36 | 0.38 | 0.33 | SNP |
| chr6_32633959_T_C | 32633959 | 1.42 | 1.01 | 2.00 | 0.04266 | 0.33 | 0.34 | 0.30 | SNP |
| chr6_29768705_C_A | 29768705 | 0.71 | 0.51 | 0.99 | 0.04269 | 0.25 | 0.23 | 0.28 | SNP |
| chr6_31380387_G_A | 31380387 | 1.65 | 1.03 | 2.71 | 0.04279 | 0.10 | 0.11 | 0.08 | SNP |
| chr6_33053167_A_C | 33053167 | 1.59 | 1.03 | 2.54 | 0.04285 | 0.10 | 0.11 | 0.08 | SNP |
| chr6_31244154_A_C | 31244154 | 0.52 | 0.28 | 0.98 | 0.04298 | 0.04 | 0.03 | 0.06 | SNP |
| chr6_33053477_G_C | 33053477 | 1.63 | 1.03 | 2.68 | 0.04301 | 0.09 | 0.10 | 0.07 | SNP |
| chr6_32559673_T_G | 32559673 | 1.43 | 1.01 | 2.04 | 0.04313 | 0.38 | 0.40 | 0.35 | SNP |
| chr6_32636217_G_A | 32636217 | 1.42 | 1.01 | 2.01 | 0.04348 | 0.32 | 0.34 | 0.29 | SNP |
| chr6_30865725_C_T | 30865725 | 0.68 | 0.46 | 0.99 | 0.04380 | 0.13 | 0.11 | 0.17 | SNP |
| chr6_32411307_C_T | 32411307 | 0.60 | 0.36 | 0.99 | 0.04422 | 0.17 | 0.15 | 0.21 | SNP |
| chr6_32412566_T_A | 32412566 | 0.60 | 0.36 | 0.99 | 0.04422 | 0.17 | 0.15 | 0.21 | SNP |
| chr6_32412592_G_A | 32412592 | 0.60 | 0.36 | 0.99 | 0.04422 | 0.17 | 0.15 | 0.21 | SNP |
| chr6_32412809_T_A | 32412809 | 0.60 | 0.36 | 0.99 | 0.04422 | 0.17 | 0.15 | 0.21 | SNP |
| chr6_33050279_A_G | 33050279 | 1.57 | 1.03 | 2.50 | 0.04424 | 0.10 | 0.11 | 0.08 | SNP |
| chr6_32610947_A_C | 32610947 | 0.46 | 0.22 | 0.99 | 0.04427 | 0.03 | 0.02 | 0.04 | SNP |
| chr6_32629403_C_T | 32629403 | 1.50 | 1.01 | 2.23 | 0.04439 | 0.23 | 0.24 | 0.20 | SNP |
| chr6_32604221_A_G | 32604221 | 0.46 | 0.22 | 0.98 | 0.04445 | 0.12 | 0.11 | 0.16 | SNP |
| chr6_30887344_T_C | 30887344 | 1.40 | 1.01 | 1.94 | 0.04450 | 0.41 | 0.43 | 0.36 | SNP |
| chr6_29892207_G_T | 29892207 | 0.55 | 0.30 | 0.98 | 0.04493 | 0.04 | 0.03 | 0.06 | SNP |
| xHLA_2field_A_01_01 | 29910309 | 1.74 | 1.02 | 3.02 | 0.04499 | 0.15 | 0.15 | 0.14 | HLA (xHLA) |
| chr6_32442703_G_A | 32442703 | 0.43 | 0.19 | 0.99 | 0.04554 | 0.02 | 0.02 | 0.03 | SNP |
| chr6_29913639_A_G | 29913639 | 1.50 | 1.01 | 2.26 | 0.04593 | 0.19 | 0.20 | 0.18 | SNP |
| chr6_30858247_C_G | 30858247 | 0.68 | 0.47 | 1.00 | 0.04599 | 0.14 | 0.12 | 0.17 | SNP |
| chr6_30858254_T_C | 30858254 | 0.68 | 0.47 | 1.00 | 0.04599 | 0.14 | 0.12 | 0.17 | SNP |
| chr6_29944577_A_G | 29944577 | 0.47 | 0.22 | 0.99 | 0.04630 | 0.03 | 0.02 | 0.05 | SNP |
| chr6_32605609_G_A | 32605609 | 1.50 | 1.01 | 2.25 | 0.04652 | 0.31 | 0.32 | 0.28 | SNP |
| chr6_31378335_G_C | 31378335 | 0.65 | 0.43 | 1.00 | 0.04664 | 0.13 | 0.12 | 0.14 | SNP |
| chr6_32629330_C_T | 32629330 | 1.43 | 1.01 | 2.04 | 0.04665 | 0.31 | 0.33 | 0.29 | SNP |
| chr6_32634139_C_T | 32634139 | 1.42 | 1.01 | 2.01 | 0.04679 | 0.33 | 0.34 | 0.30 | SNP |
| chr6_30865204_A_C | 30865204 | 0.67 | 0.45 | 1.00 | 0.04680 | 0.12 | 0.10 | 0.16 | SNP |
| chr6_29896108_A_T | 29896108 | 1.58 | 1.02 | 2.51 | 0.04709 | 0.17 | 0.18 | 0.16 | SNP |
| chr6_31851469_C_A | 31851469 | 1.46 | 1.01 | 2.13 | 0.04711 | 0.38 | 0.40 | 0.34 | SNP |
| chr6_30852747_C_T | 30852747 | 0.69 | 0.48 | 1.00 | 0.04716 | 0.14 | 0.13 | 0.18 | SNP |
| chr6_32299908_G_C | 32299908 | 1.49 | 1.01 | 2.25 | 0.04723 | 0.14 | 0.16 | 0.11 | SNP |
| chr6_31538497_T_A | 31538497 | 0.72 | 0.52 | 0.99 | 0.04726 | 0.37 | 0.36 | 0.40 | SNP |
| chr6_31323416_G_C | 31323416 | 2.24 | 1.02 | 5.05 | 0.04737 | 0.13 | 0.13 | 0.14 | SNP |
| chr6_32627812_C_G | 32627812 | 0.60 | 0.36 | 1.00 | 0.04742 | 0.17 | 0.15 | 0.21 | SNP |
| chr6_32605555_T_A | 32605555 | 1.50 | 1.01 | 2.24 | 0.04745 | 0.31 | 0.32 | 0.28 | SNP |
| chr6_29910286_T_C | 29910286 | 0.69 | 0.48 | 1.00 | 0.04745 | 0.19 | 0.18 | 0.22 | SNP |
| chr6_31326157_C_G | 31326157 | 0.40 | 0.16 | 1.00 | 0.04750 | 0.02 | 0.01 | 0.03 | SNP |
| chr6_32170433_C_T | 32170433 | 0.74 | 0.55 | 1.00 | 0.04751 | 0.49 | 0.46 | 0.54 | SNP |
| chr6_33050148_T_C | 33050148 | 1.62 | 1.02 | 2.67 | 0.04753 | 0.09 | 0.10 | 0.07 | SNP |
| xHLA_2field_C_02_02 | 31236526 | 0.53 | 0.28 | 1.00 | 0.04770 | 0.04 | 0.03 | 0.05 | HLA (xHLA) |
| chr6_31349805_A_C | 31349805 | 0.71 | 0.50 | 1.00 | 0.04772 | 0.30 | 0.29 | 0.32 | SNP |
| chr6_32628960_T_C | 32628960 | 0.59 | 0.35 | 1.00 | 0.04775 | 0.18 | 0.16 | 0.21 | SNP |
| chr6_29896967_C_T | 29896967 | 1.51 | 1.01 | 2.29 | 0.04794 | 0.20 | 0.21 | 0.19 | SNP |
| chr6_33050024_C_A | 33050024 | 1.57 | 1.02 | 2.51 | 0.04797 | 0.10 | 0.11 | 0.08 | SNP |
| chr6_33050045_T_C | 33050045 | 1.57 | 1.02 | 2.51 | 0.04797 | 0.10 | 0.11 | 0.08 | SNP |
| chr6_32298484_G_T | 32298484 | 0.66 | 0.43 | 1.00 | 0.04812 | 0.22 | 0.20 | 0.27 | SNP |
| chr6_32501311_C_T | 32501311 | 0.72 | 0.52 | 1.00 | 0.04828 | 0.30 | 0.28 | 0.35 | SNP |
| chr6_29909021_C_G | 29909021 | 1.64 | 1.01 | 2.70 | 0.04833 | 0.15 | 0.15 | 0.14 | SNP |
| chr6_33053468_G_T | 33053468 | 1.62 | 1.02 | 2.65 | 0.04835 | 0.09 | 0.10 | 0.07 | SNP |
| chr6_32501459_T_C | 32501459 | 0.72 | 0.52 | 1.00 | 0.04876 | 0.30 | 0.28 | 0.35 | SNP |
| chr6_31466860_G_A | 31466860 | 0.63 | 0.40 | 1.00 | 0.04892 | 0.09 | 0.07 | 0.11 | SNP |
| chr6_32606616_A_T | 32606616 | 1.50 | 1.01 | 2.25 | 0.04900 | 0.33 | 0.34 | 0.31 | SNP |
| chr6_31429927_T_C | 31429927 | 1.73 | 1.02 | 3.03 | 0.04907 | 0.18 | 0.18 | 0.18 | SNP |
| chr6_32629467_G_A | 32629467 | 1.45 | 1.00 | 2.12 | 0.04913 | 0.46 | 0.48 | 0.43 | SNP |
| chr6_33071600_C_A | 33071600 | 1.64 | 1.02 | 2.74 | 0.04914 | 0.09 | 0.10 | 0.06 | SNP |
| chr6_31241537_T_C | 31241537 | 0.71 | 0.51 | 1.00 | 0.04920 | 0.32 | 0.31 | 0.33 | SNP |
| chr6_32605884_C_T | 32605884 | 0.47 | 0.22 | 1.00 | 0.04955 | 0.12 | 0.11 | 0.16 | SNP |
| chr6_32605935_G_T | 32605935 | 0.47 | 0.22 | 1.00 | 0.04955 | 0.12 | 0.11 | 0.16 | SNP |
| chr6_32297310_T_A | 32297310 | 1.70 | 1.02 | 2.95 | 0.04977 | 0.08 | 0.09 | 0.06 | SNP |
| chr6_32297818_T_C | 32297818 | 1.70 | 1.02 | 2.95 | 0.04977 | 0.08 | 0.09 | 0.06 | SNP |
| chr6_32298137_G_A | 32298137 | 1.70 | 1.02 | 2.95 | 0.04977 | 0.08 | 0.09 | 0.06 | SNP |
| chr6_31322767_G_A | 31322767 | 0.40 | 0.16 | 1.01 | 0.04988 | 0.02 | 0.01 | 0.03 | SNP |
| chr6_31323414_G_A | 31323414 | 0.40 | 0.16 | 1.01 | 0.04988 | 0.02 | 0.01 | 0.03 | SNP |
| chr6_31323707_G_A | 31323707 | 0.40 | 0.16 | 1.01 | 0.04988 | 0.02 | 0.01 | 0.03 | SNP |
| chr6_31326099_A_T | 31326099 | 0.40 | 0.16 | 1.01 | 0.04988 | 0.02 | 0.01 | 0.03 | SNP |

ANCA = anitneutrophil cytoplasmic antibody; AAV = ANCA-associated vasculitis; SNP = single nucleotide polymorphism; OR = Odds ratio; CI = confidence interval; MAF = minor allele frequency. SNPs and HLA alleles with P < 0.05 included in the table.
